# Supplementary material for: Familial Dilated Cardiomyopathy: A Novel MED9 Short Isoform Identification
Source: Int J Mol Sci. 2024 Mar 6;25(5):3057. doi: 10.3390/ijms25053057 (PMC10931922; doi:10.3390/ijms25053057)
Supplement: Supplementary file 1 [file ijms-25-03057-s001.zip › ijms-2865981-supplementary.pdf]

Supplementary Table S1. Forward and Reverse oligonucleotides list used in the validation experiments.

Primer name identifier	Forward (5'-3')	Reverse (5'-3')	Amplicon size (bp)	Tm (°C)
**Mediator complex subunit9 full isoform (MED9full)**	CTTTTGGCGACCCGACCTCT	GATGGTAGTGGGGAGACAGG	542	64
**Mediator complex subunit9 short isoform (MED9short)**	GAGGGAGGAAGAGAACTACT	AAACACTTCTGCCCCACCTA	209	60
**GATA binding protein 4 (GATA4)**	TGTGCAGAGTTTGCCTCACA	AGAACGAATGCCGAGTCCTG	238	60
**Fos proto-oncogene (FOS)**	GGGAGGACCTTATCTGTGCG	ACACACTCCATGCGTTTTGC	121	60
**Zinc finger protein, FOG family member 2 (ZFPM2/FOG2)**	ATGTCCCGGCGAAAGC	ATTCCACTTCTTCGCAGCTCA	184	50
**Inhibitor of DNA binding2 (ID2)**	GACTACATCTTGGACCTGCAG	TGTTGTTGTTGTTGTTGTGCA	250	62
**Ribosomal protein S18 (RPS18)**	CGATGGGCGGCGGAAAATA	CTGCTTTCCTCAACACCACA	86	60

Supplementary Table S2: Protein-coding genes.

Progression number	Ensembl Gene id	logFC	logCPM	F	p value
1	ENSG00000000419	-0,199256449	5,091509258	0,6485326	0,42884518
2	ENSG00000000457	-0,152836378	4,016656855	1,3369877	0,351924024
3	ENSG00000000971	1,633589327	8,704127502	15,812934	0,000591823
4	ENSG00000001036	-0,061573893	4,840865043	0,2862548	0,663501991
5	ENSG00000001084	0,016826466	4,786896831	0,023546	0,87937299
6	ENSG00000001167	0,206314616	4,276750435	2,9301117	0,165959165
7	ENSG00000001461	0,08971742	5,012484199	0,3442321	0,563083862
8	ENSG00000001497	0,07108952	4,205740051	0,3319027	0,679352241
9	ENSG00000001561	-0,506861347	5,921573349	2,5573986	0,123361083
10	ENSG00000001629	0,107576853	6,004538683	0,9080594	0,350455948
11	ENSG00000001631	0,038222171	3,671497014	0,1222985	0,832757785
12	ENSG00000002549	-0,427408447	6,133910747	8,5658016	0,007553658
13	ENSG00000002586	-0,078343964	6,551945226	0,1824541	0,673225587
14	ENSG00000002834	0,434114424	5,273803568	2,9716227	0,098089473
15	ENSG00000002919	-0,040356341	3,714066608	0,0836661	0,824613968
16	ENSG00000002933	-0,400116848	5,323048357	0,7111988	0,407689476
17	ENSG00000003056	0,008446388	5,646271881	0,0046346	0,946306826
18	ENSG00000003096	0,111820968	3,936074885	0,34154	0,564603098
19	ENSG00000003147	-0,100309783	5,667332947	0,256452	0,617364994
20	ENSG00000003393	-0,042726014	6,329453498	0,0558959	0,815187095
21	ENSG00000003400	-0,017481185	4,08152452	0,011791	0,914467727
22	ENSG00000003402	-0,164862005	8,256201439	0,8344068	0,370419
23	ENSG00000003436	-0,115329097	6,171541453	0,230116	0,635946103
24	ENSG00000003509	-0,312887584	4,619907347	3,9219444	0,059684271
25	ENSG00000003756	0,095872579	6,389577609	0,5181768	0,478812102
26	ENSG00000003989	1,375299692	6,746102678	7,2014851	0,013231522
27	ENSG00000004059	0,028847042	4,540060277	0,0547343	0,884206091
28	ENSG00000004142	-0,211200947	6,167486762	2,2627148	0,146017919
29	ENSG00000004399	0,280095159	5,67845056	1,3140498	0,263392416
30	ENSG00000004455	-0,290428831	6,275131248	9,9555579	0,00439706
31	ENSG00000004478	-0,246023164	5,334358696	1,791346	0,193780514
32	ENSG00000004487	0,097480374	5,650375893	1,3462319	0,322304043
33	ENSG00000004534	-0,224758685	6,552985574	1,850799	0,186811546
34	ENSG00000004700	0,177229964	4,998190658	0,7846368	0,384850323
35	ENSG00000004766	-0,27169866	5,474023266	1,3877528	0,250772302
36	ENSG00000004779	-0,95226538	7,517724606	17,056995	0,000403808
37	ENSG00000004799	0,017049381	8,733744428	0,0008698	0,97672519
38	ENSG00000004864	-0,149333943	4,137307368	1,6213599	0,316626649
39	ENSG00000004866	-0,289911506	4,841809309	7,7588352	0,014436307
40	ENSG00000004897	-0,087974353	6,47478882	0,3543029	0,557462891
41	ENSG00000004961	-0,470808192	4,943393863	8,05181	0,009300147
42	ENSG00000005007	0,347771199	4,916177529	3,0880823	0,092107977
43	ENSG00000005020	-0,805916526	4,646699432	25,172726	4,40952E-05
44	ENSG00000005022	0,078441294	5,968157235	0,4873541	0,492050242
45	ENSG00000005059	-0,528406499	4,718700642	5,4291732	0,028891338
46	ENSG00000005075	-0,171757179	4,750567924	1,5447228	0,226334573
47	ENSG00000005100	0,01744212	3,68883388	0,0189947	0,946657694
48	ENSG00000005102	-0,564110722	4,53612932	4,761504	0,039521787
49	ENSG00000005108	-0,060140858	3,963465577	0,0422425	0,838957755
50	ENSG00000005156	0,115959127	4,38970674	0,3956591	0,535507652
51	ENSG00000005175	-0,412342765	4,894860905	5,9420397	0,022884915
52	ENSG00000005187	-0,594328837	4,277866425	2,7947568	0,108058909
53	ENSG00000005194	-0,224895428	5,016480657	4,0253043	0,056619737
54	ENSG00000005243	0,516230236	4,484962277	15,50719	0,000648095
55	ENSG00000005249	0,226872236	6,728901227	0,2068115	0,653520695
56	ENSG00000005302	-0,189579994	4,204498594	2,7369609	0,235568351
57	ENSG00000005339	0,064194376	5,706524678	0,0694518	0,794471911

58	ENSG00000005436	-0,011524817	4,94413859	0,0089674	0,933614492
59	ENSG00000005469	0,139640863	3,985188835	1,1516893	0,390279749
60	ENSG00000005471	-0,089297797	4,167596301	0,1134574	0,739281152
61	ENSG00000005483	0,205020473	6,664616281	3,4925116	0,074328072
62	ENSG00000005700	-0,173968284	6,733535563	0,9746076	0,333756518
63	ENSG00000005801	-0,304920407	4,730843	7,6280811	0,015452148
64	ENSG00000005810	0,032106955	7,727148324	0,0441197	0,835465284
65	ENSG00000005812	-0,000916589	5,858793878	2,589E-05	0,995983625
66	ENSG00000005882	0,077241818	5,300138186	0,1407124	0,710994118
67	ENSG00000005884	0,420485472	4,710923939	5,7402753	0,025063789
68	ENSG00000005889	-0,138505216	6,181673606	0,721	0,404525412
69	ENSG00000005893	-0,142922469	6,575563591	0,9316415	0,344414091
70	ENSG00000006007	-0,220075088	6,187110725	3,4437554	0,076242894
71	ENSG00000006042	0,818549331	4,67473964	11,163145	0,002821611
72	ENSG00000006125	0,071416408	7,195354024	0,385007	0,540985263
73	ENSG00000006194	0,198266949	4,211068189	1,5133516	0,238844725
74	ENSG00000006282	0,290590691	5,304233236	1,3977267	0,249125004
75	ENSG00000006327	0,262988194	4,473417956	0,4126542	0,526946571
76	ENSG00000006451	0,14161386	4,784902187	1,1835236	0,287815828
77	ENSG00000006459	0,040766478	5,975460516	0,0647205	0,801434533
78	ENSG00000006468	0,025062391	6,215162365	0,0079908	0,929540907
79	ENSG00000006530	-0,103337502	5,34111081	0,4924769	0,489831354
80	ENSG00000006576	-0,108620444	5,164165099	0,6317701	0,434771323
81	ENSG00000006607	0,22183164	4,543794084	2,5401187	0,12915825
82	ENSG00000006652	0,212930644	5,740576668	1,2093455	0,282784539
83	ENSG00000006695	-0,188478923	4,796243104	0,9684256	0,335263237
84	ENSG00000006712	0,010863528	4,986318928	0,0066919	0,958140061
85	ENSG00000006715	0,13277102	6,041608834	1,4195181	0,245532284
86	ENSG00000006744	-0,183371305	5,634447751	1,5779001	0,22159504
87	ENSG00000006757	-0,090868338	4,224199498	0,2894229	0,595733612
88	ENSG00000006831	0,148024151	5,972941675	0,7907428	0,383037634
89	ENSG00000007062	-0,333873979	4,5166104	1,2585649	0,27344308
90	ENSG00000007168	-0,017738541	7,274777985	0,0322689	0,858998851
91	ENSG00000007202	-0,008166398	6,697615757	0,0028213	0,958096272
92	ENSG00000007237	-0,121166416	6,996775077	0,2692392	0,608775515
93	ENSG00000007341	-0,166007539	4,257366818	2,2143895	0,277329653
94	ENSG00000007392	-0,048286837	5,024941596	0,081021	0,778453258
95	ENSG00000007908	-1,525910618	4,80787733	5,2966891	0,030716041
96	ENSG00000007923	-0,311290676	4,930789565	2,4420885	0,131708423
97	ENSG00000007944	0,05573359	4,649173457	0,1234188	0,728538045
98	ENSG00000008018	-0,1901598	6,567987553	1,3800219	0,252058814
99	ENSG00000008083	-0,17328236	4,353162399	1,0691437	0,311837299
100	ENSG00000008086	0,01137384	5,048047124	0,0051874	0,943202728
101	ENSG00000008130	-0,071369807	4,833721863	0,0896883	0,767250354
102	ENSG00000008256	0,452885164	5,113454941	7,7143486	0,010680367
103	ENSG00000008277	0,162539038	3,762412573	0,841518	0,374505429
104	ENSG00000008282	-0,318567644	6,349202281	3,8385343	0,062257386
105	ENSG00000008294	0,162538783	7,263505546	1,8107177	0,191430065
106	ENSG00000008311	-0,506347536	6,122014934	8,1253016	0,009026409
107	ENSG00000008394	0,236591627	5,870681625	0,1824342	0,673242271
108	ENSG00000008405	-0,058466437	4,431722012	0,0391265	0,844927642
109	ENSG00000008441	0,608141764	5,444029529	4,2537298	0,050577878
110	ENSG00000008513	0,093828597	7,157428296	0,286932	0,597310712
111	ENSG00000008517	-0,025982547	4,642435408	0,0057379	0,940270803
112	ENSG00000008710	0,725889417	5,512456808	5,1209397	0,033338721
113	ENSG00000008838	0,049598894	4,941028667	0,0845333	0,7738384
114	ENSG00000008853	0,130432196	4,644880743	0,2484905	0,622850722
115	ENSG00000008869	-0,076622264	5,82735905	0,4775208	0,496405995
116	ENSG00000008952	0,167953812	6,984327701	0,9831641	0,331686611

117	ENSG00000008988	0,175565113	8,64379239	0,5217992	0,477321055
118	ENSG00000009307	-0,013174347	9,700785331	0,006339	0,937223933
119	ENSG00000009335	-0,059881315	6,402144712	0,2402114	0,628657622
120	ENSG00000009413	-0,145371237	6,589941398	1,6550854	0,210961768
121	ENSG00000009780	-0,028570326	4,097873321	0,0649938	0,83989676
122	ENSG00000009844	-0,332465979	5,322482529	2,2778041	0,144787633
123	ENSG00000009954	0,220621454	6,471675845	2,9182477	0,100934072
124	ENSG00000010017	0,051933064	5,336681483	0,1551324	0,697275848
125	ENSG00000010072	-0,347077142	3,761860775	6,6774684	0,060285918
126	ENSG00000010165	-0,211004106	4,020553674	2,0511628	0,182894456
127	ENSG00000010219	-0,070253303	3,842976771	0,1893819	0,726444385
128	ENSG00000010244	-0,106129403	7,142178007	0,4644922	0,502304515
129	ENSG00000010256	-0,482931023	7,947767579	4,6673537	0,04134744
130	ENSG00000010270	-0,134096919	5,013235455	0,6810916	0,417648102
131	ENSG00000010278	0,768914305	5,829252543	13,331264	0,001323954
132	ENSG00000010282	-0,328151999	7,710261215	2,7163515	0,112853561
133	ENSG00000010292	0,291342294	4,242886493	2,3085642	0,142225076
134	ENSG00000010319	0,045231076	4,211211179	0,0432063	0,837157967
135	ENSG00000010322	0,352681261	5,544931184	3,7470781	0,065224956
136	ENSG00000010327	-0,260926484	6,168571399	1,2019654	0,284221413
137	ENSG00000010404	0,338811135	3,649595588	3,8741627	0,084523272
138	ENSG00000010610	0,26702174	4,148538762	1,0221396	0,32247961
139	ENSG00000010803	-0,038874149	5,465973386	0,0790828	0,781039386
140	ENSG00000010810	0,043194064	5,632929539	0,0656222	0,800087181
141	ENSG00000010818	-0,08628089	6,008570505	0,2858071	0,598025821
142	ENSG00000011007	0,131481926	5,163246815	1,0459764	0,31698499
143	ENSG00000011021	0,040176933	4,940503998	0,0244407	0,877126428
144	ENSG00000011028	0,555664218	5,517290604	3,6819628	0,067436158
145	ENSG00000011105	-0,016928288	6,842144403	0,0047919	0,94540695
146	ENSG00000011114	0,082010731	5,449416539	0,4581073	0,505204733
147	ENSG00000011198	-0,020063571	5,587928616	0,0072854	0,932714612
148	ENSG00000011201	0,677011816	4,393606666	5,053924	0,034404068
149	ENSG00000011243	-0,16309278	4,514605198	0,9383023	0,34274274
150	ENSG00000011258	0,233847686	4,766544173	3,6696934	0,067818429
151	ENSG00000011260	0,021721329	3,964942358	0,0194323	0,890341892
152	ENSG00000011275	0,342119857	5,968479621	6,1252182	0,02106194
153	ENSG00000011295	-0,154996101	5,643531809	1,9738425	0,17329282
154	ENSG00000011304	0,114928762	5,126802396	0,3640762	0,552127098
155	ENSG00000011347	-0,649100256	4,630214127	5,5728177	0,027048499
156	ENSG00000011376	0,102065783	4,523414044	0,5350909	0,561201872
157	ENSG00000011405	-0,188558689	6,886994256	1,9463607	0,17619638
158	ENSG00000011454	0,129929796	6,355185205	1,913553	0,179740676
159	ENSG00000011465	-0,098564377	10,5828003	0,149895	0,702170433
160	ENSG00000011485	0,149765096	5,086862278	0,982887	0,331753352
161	ENSG00000011523	0,074437105	5,458432161	0,3118223	0,581915293
162	ENSG00000011566	-0,130108766	6,202604859	0,6084453	0,443288973
163	ENSG00000011638	0,19428148	5,596887517	1,5612215	0,223992072
164	ENSG00000012048	-0,184404496	3,986014168	0,8958974	0,353675525
165	ENSG00000012061	-0,157978336	5,363302071	0,6436293	0,430571829
166	ENSG00000012171	0,065264699	4,48705891	0,0811319	0,778305991
167	ENSG00000012174	-0,079898284	4,483572673	0,4054618	0,632889673
168	ENSG00000012232	0,075952683	5,949766427	0,1406124	0,711091914
169	ENSG00000012660	-0,122720676	6,07687164	0,2335928	0,633419531
170	ENSG00000012817	0,293159072	5,505948359	0,2884066	0,596376033
171	ENSG00000012822	0,042251247	6,788820244	0,0816481	0,777617908
172	ENSG00000012963	0,140502851	4,836276144	2,1822761	0,266020073
173	ENSG00000012983	0,139126674	5,145696939	0,6385681	0,43236554
174	ENSG00000013016	0,38299052	4,29839878	3,4256463	0,077013466
175	ENSG00000013275	-0,05097615	5,30405701	0,164246	0,688990977

176	ENSG00000013288	0,339577663	4,839091371	4,4519548	0,045892323
177	ENSG00000013306	0,080632203	4,815994387	0,3301177	0,663100466
178	ENSG00000013364	-0,032889916	5,703223634	0,0203476	0,887806848
179	ENSG00000013374	0,133426535	5,386996267	1,7945704	0,193349413
180	ENSG00000013375	0,086765989	5,490257727	0,1569671	0,695598266
181	ENSG00000013441	0,030202438	6,909077479	0,017716	0,895265208
182	ENSG00000013503	-0,012358331	4,141548994	0,0124269	0,973939302
183	ENSG00000013523	0,144551205	4,710086435	0,4849626	0,493127568
184	ENSG00000013561	-0,162618574	6,128387822	1,0092742	0,325479261
185	ENSG00000013583	-0,103945113	4,486701328	0,4066268	0,529953764
186	ENSG00000013588	0,158252453	4,459364556	0,0632874	0,803596897
187	ENSG00000014123	-0,054244257	6,027861653	0,044032	0,835632835
188	ENSG00000014216	0,084662192	5,250769562	0,2097789	0,651217785
189	ENSG00000014641	-0,547964899	9,68261639	3,6182035	0,06968459
190	ENSG00000014824	-0,276680211	6,906535476	1,259111	0,273341726
191	ENSG00000014919	0,117747945	4,707446189	1,0903373	0,35452513
192	ENSG00000015153	-0,192957229	6,098809393	1,0237749	0,322101052
193	ENSG00000015171	0,124248919	6,795436662	1,8071842	0,191848065
194	ENSG00000015475	-0,239179364	3,797705108	3,6940625	0,201065711
195	ENSG00000015479	-0,085024577	4,778749725	0,1502668	0,70182747
196	ENSG00000015568	-0,061707471	7,595288141	0,1953896	0,662562432
197	ENSG00000015676	0,065707944	6,16176621	0,1811696	0,674307306
198	ENSG00000016864	0,076522077	5,029715456	0,6704211	0,449354947
199	ENSG00000017260	0,063741217	6,89199524	0,1386678	0,713001764
200	ENSG00000017797	0,156081217	6,056116412	3,0221414	0,095394674
201	ENSG00000018189	-0,231570093	6,006580026	3,5377136	0,072601901
202	ENSG00000018408	0,2745314	6,31996647	2,4309927	0,132546354
203	ENSG00000018510	0,017460532	5,910293108	0,0081444	0,928868974
204	ENSG00000018610	0,142281847	4,127193841	0,9569895	0,343035846
205	ENSG00000018625	-0,391107289	7,371203876	2,2104318	0,150595628
206	ENSG00000018699	0,010568933	4,271736167	0,006596	0,935967042
207	ENSG00000019144	0,276490056	6,316839975	0,9197998	0,347453431
208	ENSG00000019485	0,44381741	4,119165276	3,9451696	0,05898947
209	ENSG00000019582	0,719503003	8,016204123	4,2041513	0,051832775
210	ENSG00000019991	-0,243992866	4,446909491	0,5173981	0,479164081
211	ENSG00000019995	-0,218998713	5,257528933	1,9610639	0,174681551
212	ENSG00000020426	-0,197958366	4,523297773	1,4123811	0,246729837
213	ENSG00000020577	0,24564107	7,356314124	1,0620439	0,313413686
214	ENSG00000020922	0,016933552	4,794933648	0,0283212	0,867814732
215	ENSG00000021355	-0,3076798	5,585834622	1,9177308	0,17933025
216	ENSG00000021574	0,125470127	4,465119696	0,6845899	0,416469302
217	ENSG00000021776	-0,069771895	5,902649081	0,3451982	0,562520863
218	ENSG00000022267	-0,210262063	9,628790897	0,394993	0,535848432
219	ENSG00000022277	-0,15171734	5,624585484	1,7669279	0,196690554
220	ENSG00000022567	0,141828728	4,113103277	0,74604	0,396608106
221	ENSG00000022840	-0,218416647	7,067392822	1,4265822	0,244436925
222	ENSG00000023041	-0,080509619	4,808186964	0,5132527	0,60169468
223	ENSG00000023191	0,252607408	6,184729428	1,2626174	0,272692161
224	ENSG00000023228	-0,250696146	8,618699905	1,0195045	0,323090873
225	ENSG00000023287	-0,153620853	7,255361849	0,6798998	0,418049946
226	ENSG00000023318	-0,092869903	5,697436363	0,6425663	0,430919328
227	ENSG00000023330	-0,41404445	6,258453132	4,7682984	0,039393586
228	ENSG00000023445	0,66854823	5,381249976	1,7121289	0,203572328
229	ENSG00000023516	0,208562307	7,036419805	3,6111629	0,069893913
230	ENSG00000023572	0,012695527	4,630419959	0,0071278	0,933444532
231	ENSG00000023608	0,102211043	3,615316314	0,3872077	0,606755623
232	ENSG00000023697	-0,096233122	4,148278904	0,4466773	0,636308423
233	ENSG00000023734	-0,265771508	6,385486655	5,5896456	0,026808923
234	ENSG00000023902	0,029118503	4,928451907	0,0232257	0,880194211

235	ENSG00000023909	-0,01881597	4,223985057	0,0097977	0,922004917
236	ENSG00000024048	-0,054662499	6,812255438	0,2231846	0,641039003
237	ENSG00000024422	0,039606768	5,223827045	0,021067	0,885854985
238	ENSG00000024862	0,268488028	3,720171154	4,0574114	0,147481633
239	ENSG00000025039	-0,330086326	6,864738485	2,0424477	0,166340564
240	ENSG00000025156	-0,806576454	5,621157828	14,304522	0,000958553
241	ENSG00000025293	-0,110058029	5,7664381	1,0696279	0,311693213
242	ENSG00000025434	-0,174799862	3,672342283	0,5367003	0,471168236
243	ENSG00000025796	0,15996815	6,336709093	1,6913422	0,206198433
244	ENSG00000025800	-0,158000649	6,523517266	1,2386588	0,27714038
245	ENSG00000026025	0,618249197	8,734513203	3,7622618	0,064721303
246	ENSG00000026103	0,402308347	4,099242042	1,8305735	0,1891472
247	ENSG00000026508	-0,028949323	6,230649077	0,0080225	0,929401834
248	ENSG00000026652	-0,195377277	3,805036453	0,8536289	0,365073615
249	ENSG00000026950	0,756081799	4,602196769	10,862424	0,003147774
250	ENSG00000027001	0,072205584	4,95235523	0,1292267	0,722498744
251	ENSG00000027075	0,259650413	4,855299961	4,4000879	0,047029313
252	ENSG00000027697	-0,315310896	7,19047805	4,2617315	0,050363511
253	ENSG00000028116	-0,331270315	4,456164885	5,3026275	0,030611104
254	ENSG00000028137	-0,269638367	4,766718867	1,408431	0,247372529
255	ENSG00000028203	-0,195424896	6,483769179	0,8852258	0,356504684
256	ENSG00000028310	0,070048258	4,656376885	0,2153237	0,646966559
257	ENSG00000028528	0,292882343	6,747624121	5,0257669	0,034827076
258	ENSG00000028839	0,014978896	3,744871404	0,0072528	0,932864931
259	ENSG00000029363	-0,045315963	7,476080753	0,0599944	0,808666419
260	ENSG00000029364	0,120781706	5,64823143	0,6855111	0,416151179
261	ENSG00000029534	-0,040021678	4,925118558	0,0115214	0,915447251
262	ENSG00000029725	-0,039347392	6,244146509	0,1054038	0,748348837
263	ENSG00000030066	0,075557277	5,352508759	0,4189737	0,523804911
264	ENSG00000030419	-0,045600606	4,576216184	0,0940254	0,868814881
265	ENSG00000030582	-0,081797173	6,22644595	0,1502055	0,701885199
266	ENSG00000031003	-0,172010193	6,40124564	1,224243	0,279913245
267	ENSG00000031081	-0,185146547	6,157284253	0,7620279	0,391673767
268	ENSG00000031698	-0,211206171	6,604697409	2,724003	0,112327814
269	ENSG00000031823	0,018341206	4,824155085	0,014672	0,904636645
270	ENSG00000032219	0,016772592	5,562004855	0,0171928	0,896809945
271	ENSG00000032742	0,306825451	4,449850516	2,7951868	0,108033275
272	ENSG00000033030	0,11887642	4,38464632	0,9784442	0,424587766
273	ENSG00000033100	0,423616218	4,416394221	5,152979	0,032842486
274	ENSG00000033122	-1,181041794	4,204292219	10,113754	0,004154337
275	ENSG00000033170	-0,09727992	4,442805481	0,684983	0,531432001
276	ENSG00000033178	-0,059807323	5,785630873	0,0715032	0,791532223
277	ENSG00000033327	0,548835176	5,118732867	8,1981026	0,008763975
278	ENSG00000033627	0,172464072	5,661404835	1,5336588	0,227962566
279	ENSG00000033800	-0,131321259	5,63681662	1,4807273	0,235898392
280	ENSG00000033867	-0,316611964	5,996520504	1,9355864	0,17739668
281	ENSG00000034152	-0,33534637	5,224204402	1,7115142	0,203650677
282	ENSG00000034510	-0,184555309	7,586244521	0,5994203	0,446647197
283	ENSG00000034677	-0,246716545	6,429975091	2,2002395	0,151498379
284	ENSG00000034693	-0,191126993	4,349340689	1,1975241	0,285090784
285	ENSG00000034713	-0,032167974	6,595692763	0,0774278	0,783279554
286	ENSG00000034971	-1,144676814	5,054250544	9,371856	0,005510844
287	ENSG00000035115	0,054833648	4,595494552	0,1567442	0,698257821
288	ENSG00000035141	-0,184534752	4,254838325	1,6516078	0,277640426
289	ENSG00000035403	0,032492158	8,09717349	0,0323431	0,858843806
290	ENSG00000035664	-0,703268107	6,612301783	7,8984442	0,009901371
291	ENSG00000035681	-0,074333508	5,195822498	0,7799908	0,501930573
292	ENSG00000035687	0,12447786	4,278994406	0,4145553	0,526004543
293	ENSG00000035862	0,348624908	7,031062505	2,5465728	0,12411765

294	ENSG00000035928	0,027696198	5,948209961	0,0419249	0,839549123
295	ENSG00000036054	-0,07112324	5,693818412	0,0909564	0,765661568
296	ENSG00000036257	-0,023553132	6,06005356	0,0416484	0,8400716
297	ENSG00000036448	0,310823201	9,584466893	0,8345994	0,370378004
298	ENSG00000036549	-0,066525402	6,110314445	0,1487952	0,703216993
299	ENSG00000036672	-0,544310794	4,969878572	3,0800563	0,092506376
300	ENSG00000037474	-0,050218306	5,114918224	0,1916841	0,665568768
301	ENSG00000037637	-0,145559844	4,919251123	1,2157469	0,281506789
302	ENSG00000037749	-0,127383609	5,248748542	0,6207922	0,438759367
303	ENSG00000038210	0,217411588	3,737739972	1,4845886	0,235349411
304	ENSG00000038219	0,128667534	6,627085866	1,3445064	0,25803888
305	ENSG00000038274	-0,292351824	6,154893403	3,8186021	0,062886919
306	ENSG00000038295	0,816455868	4,064100238	2,9284945	0,1004181
307	ENSG00000038358	0,251375405	3,844330729	1,1146994	0,301974271
308	ENSG00000038382	0,004448324	6,512651778	0,0007158	0,978884784
309	ENSG00000038427	0,02661029	8,386565577	0,0077699	0,93051907
310	ENSG00000038532	-0,007722982	4,583954304	0,0024212	0,961178373
311	ENSG00000038945	0,177580862	4,823553646	0,3697417	0,549074209
312	ENSG00000039123	-0,283821902	6,400918863	1,8530879	0,186549462
313	ENSG00000039319	-0,095067607	7,003484945	0,2092843	0,651600236
314	ENSG00000039523	0,165341934	4,819613378	0,5455497	0,467576462
315	ENSG00000039537	-0,938031088	6,806228217	11,783851	0,002259363
316	ENSG00000039560	0,489484824	4,561905587	4,0407906	0,056224028
317	ENSG00000040199	0,922630999	4,64953272	5,7771817	0,024648546
318	ENSG00000040341	-0,325337412	6,478245421	2,7410534	0,111316624
319	ENSG00000040933	0,083116399	5,109534863	0,2948745	0,592312445
320	ENSG00000041357	-0,288295574	6,011894892	1,8164627	0,190798002
321	ENSG00000041802	-0,272878176	4,879230157	7,1848602	0,021606674
322	ENSG00000041982	1,445359206	4,683279039	15,564909	0,000639728
323	ENSG00000042286	0,242925818	4,248120277	0,7069401	0,409075999
324	ENSG00000042317	0,160592383	4,145123954	1,0013796	0,327339004
325	ENSG00000042429	0,043537575	3,611158702	0,0549984	0,852486606
326	ENSG00000042445	0,231755488	6,033922737	0,4238116	0,521461511
327	ENSG00000042753	-0,227456182	4,398123217	1,4966074	0,233520664
328	ENSG00000042781	-0,047198045	4,325750274	0,0261235	0,873003623
329	ENSG00000043093	-0,143795398	5,96982987	0,3831185	0,541992225
330	ENSG00000043143	0,200688416	4,855027175	0,9032418	0,351746982
331	ENSG00000043462	-0,084869658	3,725391951	0,1278359	0,723930743
332	ENSG00000043514	-0,326347522	4,656901939	5,4928509	0,028030432
333	ENSG00000043591	-0,325645071	4,67912717	1,0708512	0,311459788
334	ENSG00000044090	0,485465247	3,870629735	5,9359353	0,022947657
335	ENSG00000044115	0,110676977	8,232350332	0,7631174	0,391309763
336	ENSG00000044446	0,050271546	4,708565041	0,079195	0,780895402
337	ENSG00000044459	-0,023228365	4,625392527	0,0272558	0,943906475
338	ENSG00000044524	1,173205762	5,027586953	13,296589	0,001339518
339	ENSG00000044574	-0,305835169	8,367223185	1,246542	0,275686929
340	ENSG00000046604	0,069790128	6,573178097	0,2696148	0,608510027
341	ENSG00000046651	0,17911479	4,694899136	1,8804137	0,183409612
342	ENSG00000046653	-0,133599879	4,48213222	0,3632829	0,55255715
343	ENSG00000046889	-0,155147095	6,029598877	0,9135046	0,349077024
344	ENSG00000047056	0,15633962	5,254106558	1,1678759	0,290970255
345	ENSG00000047188	-0,125793895	5,795483524	0,8714044	0,360183721
346	ENSG00000047230	0,198520049	4,078975798	2,5228671	0,208149294
347	ENSG00000047249	0,012838683	5,12116155	0,0182097	0,949664129
348	ENSG00000047315	-0,231455549	6,871811785	1,6129518	0,216714866
349	ENSG00000047346	0,187802599	6,129326049	1,291625	0,267395338
350	ENSG00000047365	0,786296495	4,794545446	3,6600727	0,068198625
351	ENSG00000047410	0,208395975	7,656086715	2,6633858	0,116186552
352	ENSG00000047457	2,357953407	4,458205859	39,425902	2,04199E-06

353	ENSG00000047578	0,487988372	3,955270633	7,1065542	0,013774034
354	ENSG00000047597	-0,289896615	3,63455884	1,3862419	0,251023067
355	ENSG00000047617	0,032041788	3,970592443	0,0359035	0,851369055
356	ENSG00000047621	-0,268116383	4,724934691	1,6406784	0,212936501
357	ENSG00000047634	-0,391262183	4,279287884	7,9187324	0,010661614
358	ENSG00000047644	0,267075764	5,060342759	2,9337678	0,10012996
359	ENSG00000047849	0,483090447	9,450377693	4,073441	0,055313675
360	ENSG00000047932	0,206713366	5,593329951	2,2378065	0,148164077
361	ENSG00000048028	-0,042961041	6,921003487	0,0294415	0,865257139
362	ENSG00000048052	0,176152786	5,614878871	0,7384382	0,398986897
363	ENSG00000048342	0,151335836	4,806032358	0,9705251	0,334750487
364	ENSG00000048392	-0,07532851	5,686971484	0,1324276	0,719235897
365	ENSG00000048405	-0,03035045	5,221454372	0,0389146	0,84534254
366	ENSG00000048471	0,192440303	4,660908975	0,7332168	0,400633222
367	ENSG00000048540	0,602720967	4,263515412	2,1312726	0,157780601
368	ENSG00000048544	-0,374779021	5,531241369	3,1901481	0,087210695
369	ENSG00000048649	0,090279027	6,432670973	0,64785	0,429056502
370	ENSG00000048707	-0,099259394	7,655315362	0,3898321	0,538486321
371	ENSG00000048740	0,027656352	6,888383846	0,0458514	0,832317403
372	ENSG00000048828	0,094664943	6,670343271	0,7890037	0,383520967
373	ENSG00000048991	-0,081221671	4,497180851	0,3433161	0,563580272
374	ENSG00000049130	-0,419002324	5,06859551	1,6455396	0,212282589
375	ENSG00000049167	0,098570061	3,855983544	0,3994662	0,541752461
376	ENSG00000049239	0,454770271	6,335413019	3,2362682	0,085096847
377	ENSG00000049245	0,016752928	6,346036275	0,0151129	0,903218016
378	ENSG00000049246	0,841977606	5,291812166	12,594722	0,001701764
379	ENSG00000049323	0,412149701	7,971290337	10,484066	0,003607276
380	ENSG00000049449	-0,124137317	4,722517351	0,4016932	0,532438805
381	ENSG00000049540	-0,408007318	6,764035142	1,1638351	0,291801911
382	ENSG00000049618	-0,068906484	6,75831161	0,1616673	0,691320255
383	ENSG00000049656	0,081581126	4,93696955	0,2469969	0,623892143
384	ENSG00000049759	0,029679696	6,216773421	0,0195789	0,889931663
385	ENSG00000049860	0,247723094	6,045415393	3,0038781	0,096353347
386	ENSG00000049883	-0,156504577	4,385358923	1,0316131	0,320295087
387	ENSG00000050130	-0,155433545	5,112200126	0,6189583	0,439427511
388	ENSG00000050165	0,536933635	10,68228189	3,7920002	0,063747654
389	ENSG00000050344	-0,310954842	4,741742779	2,5244578	0,12568025
390	ENSG00000050393	-0,293530466	4,532270007	8,9913401	0,039137402
391	ENSG00000050405	0,360710498	5,879349897	2,7164168	0,112849468
392	ENSG00000050426	0,286515009	4,927903683	5,1297116	0,033166739
393	ENSG00000050748	-0,218984926	5,479642912	6,7518579	0,024510245
394	ENSG00000051009	0,231032167	4,03014615	2,0350983	0,20370809
395	ENSG00000051108	0,321680575	6,33682504	3,885111	0,06080533
396	ENSG00000051382	0,101666643	5,391243095	1,0047244	0,326513402
397	ENSG00000051620	-0,377253769	6,036172121	4,013712	0,056991936
398	ENSG00000051825	-0,307109993	5,183109548	3,1611291	0,088571787
399	ENSG00000052126	-0,031805844	7,007401489	0,0376152	0,847912315
400	ENSG00000052723	0,041323129	5,239098587	0,0842874	0,774153265
401	ENSG00000052749	0,183158392	4,167684866	0,5513808	0,465234419
402	ENSG00000052795	0,135621323	6,396573905	1,0976724	0,305573115
403	ENSG00000052802	0,072664599	4,183752493	0,1023666	0,75188223
404	ENSG00000052841	-0,02302017	6,28924335	0,0344055	0,85445954
405	ENSG00000053254	-0,181649659	6,475526731	1,1610568	0,292364728
406	ENSG00000053371	0,226553877	4,340780564	3,1370562	0,157577113
407	ENSG00000053372	-0,295232566	3,98022818	3,5263683	0,088591033
408	ENSG00000053747	0,112624562	4,53044545	0,1357966	0,715849828
409	ENSG00000053770	-0,39123188	6,225113075	2,9107111	0,101396948
410	ENSG00000053900	0,046352262	5,098200403	0,1529827	0,699271069
411	ENSG00000054116	-0,168814428	5,16076971	3,0130615	0,110820865

412	ENSG00000054118	0,089916227	6,8278031	0,6503213	0,428189556
413	ENSG00000054148	-0,026542479	5,158044174	0,0245769	0,876787224
414	ENSG00000054267	0,189438551	5,734284136	2,9342232	0,100058463
415	ENSG00000054282	0,110141482	5,081954482	0,6979495	0,411997172
416	ENSG00000054523	-0,004785037	7,589450371	0,0006344	0,980122104
417	ENSG00000054611	0,161947751	3,950137346	0,7745097	0,404057769
418	ENSG00000054654	-0,293286681	8,855933584	3,625418	0,069381822
419	ENSG00000054793	0,673589008	6,043670655	5,4800882	0,028222566
420	ENSG00000054965	0,077689492	5,81707915	0,2106667	0,650532673
421	ENSG00000054983	-0,049650376	5,066124761	0,1898429	0,737426863
422	ENSG00000055044	-0,158416533	4,903156408	1,2432161	0,276290605
423	ENSG00000055070	-0,027104534	5,304735889	0,0261079	0,873041199
424	ENSG00000055118	-0,174701865	5,136242328	0,632163	0,434652375
425	ENSG00000055130	-0,109313449	6,165349582	1,5028596	0,23253384
426	ENSG00000055147	-0,031519021	5,329143778	0,0359261	0,851322853
427	ENSG00000055163	0,124178408	4,543853882	0,6500482	0,428313657
428	ENSG00000055208	0,011172801	6,264230801	0,0103408	0,919876953
429	ENSG00000055211	-0,033588887	5,601582031	0,1007496	0,884290281
430	ENSG00000055332	0,251253683	6,242601805	2,3448613	0,139270439
431	ENSG00000055483	0,014959881	4,654873413	0,0050172	0,944140806
432	ENSG00000055609	0,056937989	7,559127651	0,1335864	0,718056434
433	ENSG00000055917	0,004241403	6,971798188	0,0015962	0,968472868
434	ENSG00000055950	-0,039655349	4,115758972	0,1170437	0,803330411
435	ENSG00000056050	-0,292363788	4,126494419	3,864242	0,09061077
436	ENSG00000056097	-0,052558403	6,787269891	0,1696925	0,684167446
437	ENSG00000056586	-0,026968249	6,743439841	0,0470251	0,830219301
438	ENSG00000056972	0,015349411	3,959889643	0,0066772	0,93557739
439	ENSG00000057019	-0,348430654	7,232611753	3,2506445	0,084450049
440	ENSG00000057252	-0,098830066	5,049853812	0,2649211	0,611646512
441	ENSG00000057294	0,111435879	7,564989259	0,1814515	0,674069444
442	ENSG00000057608	-0,107418061	7,105401855	0,3747494	0,546402519
443	ENSG00000057663	-0,144640071	5,070599078	0,8978397	0,353164039
444	ENSG00000057757	0,100634139	4,81181021	0,8584638	0,421967286
445	ENSG00000057935	0,009964318	4,589818945	0,0110081	0,94028736
446	ENSG00000058056	-0,017606635	7,170489824	0,0059886	0,938982424
447	ENSG00000058063	-0,177267765	6,609044756	1,1481387	0,295000827
448	ENSG00000058091	0,186128961	5,883728948	1,334141	0,259872095
449	ENSG00000058262	0,101052237	6,647760489	0,4061587	0,530188595
450	ENSG00000058272	-0,092010146	6,909272897	0,4552552	0,506520433
451	ENSG00000058404	0,33704136	6,07963472	2,2309517	0,14879761
452	ENSG00000058600	-0,060315798	4,525785867	0,2205041	0,643040734
453	ENSG00000058668	0,294750257	8,010016755	2,6291753	0,118474861
454	ENSG00000058729	-0,122150363	4,816480714	0,3248719	0,574193542
455	ENSG00000058799	-0,189980839	4,067819446	3,4529631	0,262393105
456	ENSG00000058804	-0,089989607	4,395951118	0,2423534	0,627155235
457	ENSG00000058866	0,420478632	4,916770516	3,4121073	0,077561158
458	ENSG00000059378	0,382208975	4,098427441	7,806707	0,020501893
459	ENSG00000059573	0,469851688	4,712711736	8,2862692	0,00845735
460	ENSG00000059588	0,154156787	5,047753541	1,1373853	0,297210469
461	ENSG00000059691	-0,723788861	5,658860077	10,331861	0,003828789
462	ENSG00000059728	-0,017088694	4,40543538	0,0026119	0,959679287
463	ENSG00000059758	-0,112663995	5,609263162	0,3580185	0,555427629
464	ENSG00000059804	0,178302297	5,669080044	0,2154428	0,646876645
465	ENSG00000060138	-0,08043505	7,679113339	0,1996197	0,659186469
466	ENSG00000060237	-0,248390938	8,444570288	1,5472331	0,226012599
467	ENSG00000060339	-0,062819124	5,679210501	0,4193115	0,523639137
468	ENSG00000060688	0,039925533	4,466688705	0,1514365	0,82488787
469	ENSG00000060749	0,171952383	6,068840153	2,0869238	0,161939769
470	ENSG00000060762	-0,319074566	7,296394734	1,4687136	0,237792969

471	ENSG00000060971	-0,176231702	5,047678062	1,392038	0,250062848
472	ENSG00000060982	0,055571359	5,270429353	0,0241227	0,877921439
473	ENSG00000061273	0,007678494	5,089810014	0,0014124	0,97034362
474	ENSG00000061676	-0,100292247	8,051782928	0,4933971	0,489405991
475	ENSG00000061794	-0,246587772	5,728807284	1,5319873	0,228241038
476	ENSG00000061918	-0,064679655	4,530748158	0,176497	0,67828101
477	ENSG00000061936	-0,168518419	4,959258327	0,8000698	0,380292872
478	ENSG00000061987	0,094602461	6,593049844	0,3776923	0,544843971
479	ENSG00000062194	-0,077877945	6,670272721	0,3014413	0,588230913
480	ENSG00000062282	0,225995213	5,132838812	0,120494	0,731640148
481	ENSG00000062370	-0,178247603	3,640416349	1,1507948	0,379391277
482	ENSG00000062485	-0,246214128	8,026909605	1,6960292	0,205636815
483	ENSG00000062598	0,057104965	4,794678759	0,1397436	0,71194324
484	ENSG00000062650	-0,012650695	6,008201877	0,0141299	0,906402471
485	ENSG00000062716	-0,208874897	7,299052874	0,2586176	0,615891509
486	ENSG00000062725	0,106957525	5,578832215	0,6643013	0,423336144
487	ENSG00000063046	0,218101432	8,667517647	1,3073086	0,264557511
488	ENSG00000063177	0,284532619	7,129410116	1,8067829	0,191940698
489	ENSG00000063244	0,13247742	5,218505823	0,6555794	0,426382578
490	ENSG00000063245	-0,22932841	5,538433148	1,313791	0,263438159
491	ENSG00000063322	0,233881638	4,363717733	2,6975733	0,14922178
492	ENSG00000063587	0,278135674	3,968904717	2,520269	0,125978827
493	ENSG00000063601	0,164860805	4,502022653	1,7054255	0,240896162
494	ENSG00000063660	-0,128726325	5,805510507	0,4172283	0,524685252
495	ENSG00000063854	-0,048532406	5,501399149	0,0768693	0,784050445
496	ENSG00000063978	0,063300042	5,049798828	0,248999	0,699816609
497	ENSG00000064042	0,279981631	7,546323895	3,0753683	0,092708092
498	ENSG00000064102	-0,263435141	4,450948636	3,3032435	0,085177721
499	ENSG00000064115	0,35045708	5,081622851	6,2076567	0,0203114
500	ENSG00000064205	0,222255163	4,985985285	0,3589747	0,554904115
501	ENSG00000064225	0,063297282	5,776329316	0,0717902	0,791124496
502	ENSG00000064309	1,150899814	5,112646761	4,6505949	0,041682252
503	ENSG00000064313	-0,025159429	5,149033917	0,0329655	0,857503887
504	ENSG00000064393	0,125951008	7,632565462	0,1412306	0,710487904
505	ENSG00000064419	0,082495688	5,521152677	0,798062	0,381969167
506	ENSG00000064601	-0,393057006	5,745532695	7,8629846	0,010030045
507	ENSG00000064607	-0,132428846	6,219438303	0,6251613	0,437174079
508	ENSG00000064651	-0,250001117	5,593038318	1,4327039	0,243456931
509	ENSG00000064652	-0,092764354	3,697759505	0,4073245	0,6590235
510	ENSG00000064666	0,204695376	4,668405149	0,8691878	0,360817638
511	ENSG00000064703	-0,099534255	4,132161684	0,945172	0,542143545
512	ENSG00000064726	-0,23260898	7,420001065	0,83021	0,371617347
513	ENSG00000064763	-0,434906088	4,150948468	8,8643624	0,006715674
514	ENSG00000064933	-0,000206117	4,628699268	4,765E-07	0,999455128
515	ENSG00000064989	-0,409981383	6,552809028	1,2835291	0,268860002
516	ENSG00000064995	-0,269112605	4,218759634	8,8920266	0,088210765
517	ENSG00000064999	-0,204853581	5,35993302	0,9302686	0,34477696
518	ENSG00000065000	-0,134978162	5,930523614	0,6709936	0,421071928
519	ENSG00000065029	0,122106906	4,562965267	0,4374438	0,514898735
520	ENSG00000065060	-0,172230443	5,593452175	1,4270654	0,244342377
521	ENSG00000065135	-0,032292973	6,188789161	0,0680767	0,796460606
522	ENSG00000065150	0,211790417	7,484105088	1,8123383	0,191250425
523	ENSG00000065154	0,423585433	6,038908475	3,0273001	0,095175365
524	ENSG00000065183	-0,148100069	4,978700481	1,2669205	0,271857858
525	ENSG00000065243	-0,002503042	6,298068091	0,0002619	0,987226432
526	ENSG00000065308	0,051565211	4,974562374	0,0952054	0,760424944
527	ENSG00000065320	0,001533069	6,087206813	2,936E-05	0,995723504
528	ENSG00000065413	-0,009571907	5,033710071	0,0029966	0,95681527
529	ENSG00000065427	-0,328080617	6,594470301	4,764004	0,03945929

530	ENSG00000065457	0,031794432	3,635537336	0,0477342	0,885892909
531	ENSG00000065518	-0,555971863	6,614857814	9,6634279	0,004926994
532	ENSG00000065526	0,176178534	6,353437379	0,7532303	0,394377569
533	ENSG00000065534	0,005356018	5,856763893	0,0003141	0,986012644
534	ENSG00000065548	-0,16329845	5,912293885	0,5934708	0,448883383
535	ENSG00000065559	-0,014527237	5,592886748	0,0082948	0,928217013
536	ENSG00000065613	0,300634353	7,414270002	2,5277226	0,125448111
537	ENSG00000065615	-0,148029318	4,600162005	1,3144551	0,319511212
538	ENSG00000065665	0,007795746	3,72965153	0,0022212	0,998833411
539	ENSG00000065802	0,290582666	5,336988687	3,2858435	0,082890195
540	ENSG00000065809	1,818484107	5,301869085	8,5323918	0,007662176
541	ENSG00000065833	-0,839538169	5,528593732	26,545536	3,1547E-05
542	ENSG00000065882	-0,571605455	6,895678788	15,767895	0,000597336
543	ENSG00000065883	0,025208776	6,149399618	0,0379046	0,847330153
544	ENSG00000065911	-0,584555438	4,434094525	10,96122	0,003036275
545	ENSG00000065923	-0,108939748	4,009879657	0,2996321	0,589360359
546	ENSG00000065970	0,239811171	4,817363074	1,5382318	0,227324937
547	ENSG00000065978	-0,127843871	8,719642825	1,0282133	0,32104042
548	ENSG00000066027	-0,151339727	6,48855793	1,6359394	0,213532563
549	ENSG00000066044	0,014362484	5,045582076	0,0129388	0,940424864
550	ENSG00000066056	0,15499966	4,594957306	0,5178057	0,478992881
551	ENSG00000066084	0,127319706	5,576370363	1,1901837	0,286496572
552	ENSG00000066117	0,087287311	4,393494721	0,2897805	0,656397128
553	ENSG00000066135	0,213413575	3,740152121	1,7988634	0,27544196
554	ENSG00000066136	-0,056282736	5,365047287	0,1142463	0,73841147
555	ENSG00000066294	0,010087833	3,954297741	0,0011029	0,973792644
556	ENSG00000066322	0,073123177	4,547318204	0,2095163	0,651420766
557	ENSG00000066422	-0,007176173	4,993809279	0,0023144	0,962043014
558	ENSG00000066427	0,116944317	5,280974455	0,9182299	0,347823021
559	ENSG00000066455	-0,027240811	5,315108917	0,0434839	0,836637268
560	ENSG00000066557	-0,442654751	4,697746583	2,9286831	0,100407779
561	ENSG00000066583	-0,892583685	6,217198002	19,7038	0,000186656
562	ENSG00000066629	-0,024204053	5,054332815	0,0506028	0,823987728
563	ENSG00000066651	-0,131859836	4,788279527	0,7339786	0,400392372
564	ENSG00000066654	0,005296915	5,737886028	0,0016811	0,967645853
565	ENSG00000066697	0,002232688	3,87684432	0,0001857	0,989244129
566	ENSG00000066739	0,083157786	6,197277906	0,5031048	0,485208714
567	ENSG00000066777	0,005048043	6,519770959	0,001738	0,967102742
568	ENSG00000066855	-0,267969086	4,607606994	2,4963572	0,127699397
569	ENSG00000066926	0,171440184	5,07297883	1,1659825	0,291367877
570	ENSG00000066933	-0,009812789	6,890032931	0,0076624	0,930997552
571	ENSG00000067048	0,121130074	5,944242743	0,0575644	0,812502854
572	ENSG00000067057	0,132749275	6,080581297	0,5453022	0,467676304
573	ENSG00000067064	-0,16981845	5,163611975	0,4574581	0,505526972
574	ENSG00000067066	0,053486752	6,323727632	0,1694803	0,684353731
575	ENSG00000067082	0,5041493	6,394837063	2,2092732	0,150697914
576	ENSG00000067113	0,124672221	5,142978847	0,5334527	0,472497875
577	ENSG00000067141	0,687264823	5,183419496	3,8920621	0,060591948
578	ENSG00000067167	0,098252348	6,933231625	0,1873004	0,669184664
579	ENSG00000067177	-0,02820888	4,333874129	0,0259162	0,873504093
580	ENSG00000067182	-0,163703023	5,542288403	0,4130315	0,526759349
581	ENSG00000067208	0,279172842	6,158453385	2,9202048	0,100873014
582	ENSG00000067225	-0,243895882	9,204918904	1,9978385	0,170805019
583	ENSG00000067248	-0,038118766	5,721864145	0,0516938	0,822140921
584	ENSG00000067334	-0,242917965	5,786004067	1,9684616	0,173902686
585	ENSG00000067365	-0,29207527	3,733933704	2,6367932	0,117975405
586	ENSG00000067369	0,030947954	6,130548248	0,0612908	0,806645739
587	ENSG00000067533	-0,476149189	5,464034524	9,8743939	0,004546981
588	ENSG00000067560	0,111688937	7,740998406	0,8835262	0,356924611

589	ENSG00000067596	0,078270066	5,242133258	0,4180458	0,524260892
590	ENSG00000067646	-0,045467333	4,492446358	0,0112235	0,916543416
591	ENSG00000067704	-0,019175852	6,60290654	0,0339524	0,855409645
592	ENSG00000067798	-0,160091054	4,524689366	0,3097311	0,583194168
593	ENSG00000067829	-0,105718061	4,771045324	0,4614052	0,503714259
594	ENSG00000067900	0,096683126	6,865549619	0,7871806	0,38406197
595	ENSG00000067955	0,218580273	5,035122581	1,9957196	0,17106903
596	ENSG00000067992	0,258093936	6,230175789	3,4371539	0,07650648
597	ENSG00000068001	-0,14391277	4,483165523	0,3723973	0,547654334
598	ENSG00000068024	-0,060856324	4,233521835	0,0995101	0,755248967
599	ENSG00000068097	0,063680536	4,228780222	0,2428609	0,739692463
600	ENSG00000068305	0,109327266	7,341675057	0,3791326	0,544084273
601	ENSG00000068308	0,265404981	4,490590264	3,66988	0,08324355
602	ENSG00000068323	-0,005632894	4,562434276	0,0006903	0,979264764
603	ENSG00000068366	0,114028574	6,702893884	0,298874	0,589828703
604	ENSG00000068383	-0,089545965	5,893891253	0,2688306	0,609045875
605	ENSG00000068400	0,094818751	4,59541738	0,3653968	0,551412571
606	ENSG00000068615	0,599637178	5,194520927	4,6836921	0,041023935
607	ENSG00000068650	0,263315186	5,78576908	1,1561162	0,293369174
608	ENSG00000068654	-0,089623344	5,138662608	0,1646435	0,688648772
609	ENSG00000068697	-0,082817642	7,097930414	0,3503684	0,55963086
610	ENSG00000068724	0,104088506	3,734520796	0,2785417	0,622693654
611	ENSG00000068745	0,224765107	4,887022048	1,9523805	0,175601212
612	ENSG00000068784	0,061821551	4,747372634	0,1414579	0,710266197
613	ENSG00000068796	-0,000904766	4,457298772	3,298E-05	0,995467196
614	ENSG00000068878	-0,187983505	7,117300522	0,9828089	0,331772176
615	ENSG00000068885	0,291695682	4,027011701	1,7966344	0,193147779
616	ENSG00000068903	-0,507480634	5,799205169	6,0769105	0,021546257
617	ENSG00000068912	0,257131392	5,859135357	3,1923381	0,087081755
618	ENSG00000068976	-0,939305508	5,442641142	14,260573	0,000972433
619	ENSG00000069020	-0,017362881	6,732223631	0,0107699	0,918240724
620	ENSG00000069122	-0,406145863	6,78368019	8,7116917	0,007118226
621	ENSG00000069248	-0,057048209	5,960968172	0,2033506	0,656217473
622	ENSG00000069275	-0,017679121	8,626696561	0,0206331	0,887023657
623	ENSG00000069329	-0,00235834	6,871440601	0,0002352	0,987896334
624	ENSG00000069345	-0,099660438	6,469925547	0,6243126	0,437453492
625	ENSG00000069431	-0,233003715	6,946144065	2,1422907	0,156756231
626	ENSG00000069493	0,082903198	4,842016373	0,2133993	0,648434804
627	ENSG00000069509	-0,328986994	4,079822901	2,6242458	0,118807872
628	ENSG00000069535	0,124045703	6,094083526	0,4615434	0,503650977
629	ENSG00000069667	0,177329183	6,267718529	0,8683423	0,361047091
630	ENSG00000069702	-1,128086495	6,863262445	19,465995	0,000199625
631	ENSG00000069849	-0,654702381	5,817529889	6,9787867	0,014543448
632	ENSG00000069869	0,230053682	5,090558179	1,3574856	0,255858065
633	ENSG00000069956	0,12931052	5,468766407	0,2131081	0,648657536
634	ENSG00000069966	-0,542697456	4,933309427	11,529503	0,002473355
635	ENSG00000069974	-0,519389287	4,039256298	4,4038635	0,046982335
636	ENSG00000069998	-0,151457599	4,554170235	0,9045144	0,351414343
637	ENSG00000070010	-0,198325358	4,604637595	5,0469739	0,139505582
638	ENSG00000070018	0,064673883	5,97307278	0,4042356	0,531133859
639	ENSG00000070047	0,135112608	4,17314412	0,6021136	0,46940151
640	ENSG00000070061	0,081090823	5,959698005	0,5369268	0,47105021
641	ENSG00000070081	-0,211903748	6,447931298	0,7978127	0,380954423
642	ENSG00000070087	0,035029372	5,58840697	0,0583129	0,811309115
643	ENSG00000070159	0,169942459	4,595197848	0,9050778	0,351267206
644	ENSG00000070182	0,286149113	5,984571179	0,6652921	0,423024245
645	ENSG00000070214	0,142374747	5,826595753	0,5886226	0,450718975
646	ENSG00000070269	0,129690066	4,066038156	0,8952564	0,428273823
647	ENSG00000070366	0,204748369	4,536675037	0,7261599	0,402874579

648	ENSG00000070367	-0,123216493	6,385150081	0,475804	0,497197124
649	ENSG00000070371	-0,434099123	5,236860437	4,2275826	0,051235363
650	ENSG00000070404	0,159729875	5,555813577	0,2379639	0,630275986
651	ENSG00000070413	0,169723423	5,054346739	0,8065212	0,378411205
652	ENSG00000070476	0,030444827	4,396449508	0,0264178	0,872296768
653	ENSG00000070495	-0,416816371	5,317307322	3,9045371	0,060211125
654	ENSG00000070501	-0,213665876	4,251378413	2,087601	0,193398638
655	ENSG00000070540	-0,032785168	4,990882613	0,0340379	0,855235406
656	ENSG00000070610	-0,135702213	6,285936897	0,501818	0,485786083
657	ENSG00000070614	0,025952407	5,954684313	0,0093386	0,923848032
658	ENSG00000070718	0,40173142	4,525748917	6,6137266	0,01701707
659	ENSG00000070756	0,411331383	7,427682709	3,3488197	0,080181374
660	ENSG00000070761	-0,166043171	4,343175474	1,2793902	0,301426123
661	ENSG00000070770	0,185671997	4,38468522	1,2419474	0,27655081
662	ENSG00000070778	0,40360404	5,821676051	6,4070882	0,018621748
663	ENSG00000070785	-0,388441363	5,089180136	4,3076161	0,049252749
664	ENSG00000070808	-0,782658852	4,164785703	8,9075275	0,006603962
665	ENSG00000070814	-0,14321118	4,154376361	0,4857321	0,492788292
666	ENSG00000070831	-0,101178989	7,516088501	0,58157	0,453384088
667	ENSG00000070882	-0,14106679	4,073818961	0,273806	0,605770989
668	ENSG00000070961	0,658287395	6,11291737	4,6687936	0,041318815
669	ENSG00000071051	-0,067336497	4,101168066	0,2196428	0,643691071
670	ENSG00000071054	0,009908702	6,759323647	0,0065034	0,936416984
671	ENSG00000071073	0,057244784	5,892903797	0,1345854	0,717061673
672	ENSG00000071082	0,027709261	8,615205623	0,0100979	0,920823313
673	ENSG00000071127	-0,059623393	7,209778597	0,1562668	0,69623239
674	ENSG00000071189	-0,068114729	6,805514559	0,1279327	0,723830786
675	ENSG00000071205	-0,049110472	6,472182031	0,2383426	0,629989424
676	ENSG00000071242	0,182468015	6,273908621	0,6560364	0,426223666
677	ENSG00000071243	-0,206641141	4,391463618	1,5987149	0,218687616
678	ENSG00000071246	0,660054604	5,099763216	7,7050455	0,01072149
679	ENSG00000071282	0,301357348	5,195291095	1,0214928	0,322629508
680	ENSG00000071462	-0,319195799	5,100733162	4,6091357	0,042508066
681	ENSG00000071537	0,052365951	6,828138256	0,1927132	0,66473037
682	ENSG00000071553	-0,048590283	5,762730578	0,1117845	0,741130291
683	ENSG00000071575	0,234109466	5,062454441	3,1173048	0,090627923
684	ENSG00000071626	-0,268473949	5,332428366	2,8212255	0,106494328
685	ENSG00000071794	0,093536683	6,034426635	0,19526	0,662681252
686	ENSG00000071859	-0,074697585	4,874987454	0,1685019	0,685227148
687	ENSG00000071967	0,558803387	8,335964015	3,2015532	0,086682377
688	ENSG00000071991	-0,207213824	6,800466428	0,7236822	0,403666008
689	ENSG00000071994	-0,216863024	5,004028807	3,2244202	0,085588396
690	ENSG00000072042	-0,152961699	4,745059337	1,4700516	0,305475736
691	ENSG00000072062	-0,153372356	6,571864384	0,3668377	0,550634985
692	ENSG00000072110	0,069169984	6,981717118	0,0915141	0,764966782
693	ENSG00000072121	0,166381012	5,186291485	0,8974984	0,353253847
694	ENSG00000072134	-0,005989936	4,577188376	0,0005868	0,980882668
695	ENSG00000072135	0,089881685	4,282265858	0,2299674	0,636054557
696	ENSG00000072163	-0,325048486	6,120957267	1,6809396	0,207595477
697	ENSG00000072195	0,069188642	6,170095162	0,1163816	0,736074392
698	ENSG00000072201	-0,166039806	3,841514855	0,9618457	0,353721029
699	ENSG00000072210	0,420968259	6,317711772	4,1794982	0,052469866
700	ENSG00000072274	-0,079284229	7,208995977	0,0913285	0,765197737
701	ENSG00000072310	-0,501282716	4,739322099	2,4487725	0,131206696
702	ENSG00000072364	0,17985801	7,297063381	1,800518	0,1926397
703	ENSG00000072401	-0,271693575	5,463859913	1,2607837	0,273031608
704	ENSG00000072415	0,120991008	5,559776117	0,7599339	0,392289303
705	ENSG00000072422	0,242687116	5,064252055	5,0993279	0,033642711
706	ENSG00000072501	-0,060255019	5,850331807	0,1189732	0,733269907

707	ENSG00000072506	-0,216939938	5,013172002	1,4247166	0,244736568
708	ENSG00000072518	-0,167780694	4,473417435	0,5903251	0,450073015
709	ENSG00000072609	-0,081535538	4,009973456	0,1932019	0,664347323
710	ENSG00000072682	-0,343504294	5,420821029	3,4784621	0,07491891
711	ENSG00000072694	-0,554787305	4,520621585	5,9117967	0,023197638
712	ENSG00000072736	0,163506194	4,755804817	1,2070527	0,283229903
713	ENSG00000072756	-0,135752465	4,501661798	1,1533818	0,367165228
714	ENSG00000072778	-0,188891181	8,663294865	0,82648	0,372675273
715	ENSG00000072786	0,053545362	4,218293654	0,0677857	0,796893916
716	ENSG00000072803	0,20977674	6,340018459	4,3875324	0,047319206
717	ENSG00000072840	0,66199796	4,060986755	5,2744441	0,031034877
718	ENSG00000072849	0,062816041	4,593136593	0,2201367	0,643316282
719	ENSG00000072952	-0,555507942	4,985457933	6,581296	0,017258436
720	ENSG00000072958	0,094272797	4,507178949	0,33856	0,566294263
721	ENSG00000073008	-0,698988074	5,016685413	13,934332	0,001082603
722	ENSG00000073060	0,118703718	3,873690923	0,1885947	0,668116023
723	ENSG00000073331	0,040692872	4,621603171	0,043682	0,836277527
724	ENSG00000073417	-0,001444794	5,467096362	9,994E-05	0,992109469
725	ENSG00000073464	-0,308932086	5,300991878	2,303848	0,142614442
726	ENSG00000073578	-0,243323564	5,801537885	1,1105689	0,302850978
727	ENSG00000073584	-0,241327575	4,472141425	3,8639624	0,085332024
728	ENSG00000073614	0,096569385	6,745215303	0,8245235	0,373199497
729	ENSG00000073711	-0,386955372	6,584682815	3,1380516	0,089671729
730	ENSG00000073712	-0,264029743	7,132353112	3,2669846	0,083686021
731	ENSG00000073737	-1,617078724	7,121767955	14,292616	0,000962292
732	ENSG00000073792	0,013629528	3,789093211	0,0051718	0,980935785
733	ENSG00000073803	0,05676466	4,183856373	0,1870302	0,684658029
734	ENSG00000073849	0,385338542	6,051255248	3,714875	0,066308007
735	ENSG00000073905	-0,413433801	3,984873418	4,4575397	0,045767597
736	ENSG00000073910	0,022282023	7,58836123	0,0176534	0,895449365
737	ENSG00000073921	-0,011800357	7,676549256	0,0054478	0,941795512
738	ENSG00000073969	-0,082261824	5,253445737	0,422956	0,52185621
739	ENSG00000074054	0,046864263	6,914983046	0,1116484	0,741278337
740	ENSG00000074181	0,11991669	5,245087412	0,2084263	0,652265045
741	ENSG00000074201	0,130173581	5,702697973	1,3854761	0,251108905
742	ENSG00000074219	-0,419070557	4,622808728	5,0950598	0,033745691
743	ENSG00000074266	-0,059655831	4,171181617	0,2254005	0,762448266
744	ENSG00000074319	-0,104628944	5,396104843	1,3340527	0,314608642
745	ENSG00000074356	0,164013561	5,447776064	1,2142901	0,281827221
746	ENSG00000074416	0,455374587	6,414676079	3,8349859	0,062369616
747	ENSG00000074527	0,081062747	6,423297161	0,2364071	0,631391454
748	ENSG00000074582	-0,130854199	4,058993754	0,8662216	0,39980557
749	ENSG00000074590	0,305633373	5,58913219	2,6243277	0,118802411
750	ENSG00000074603	-0,052377916	6,112789747	0,2088016	0,651959259
751	ENSG00000074657	0,141469335	6,369161831	1,1892649	0,286678086
752	ENSG00000074695	-0,072974483	7,443737917	0,1369339	0,714717618
753	ENSG00000074696	0,275152347	4,993848201	2,7173478	0,112791093
754	ENSG00000074755	-0,029355684	6,16256084	0,021943	0,883523698
755	ENSG00000074800	0,018498723	7,634163332	0,0063432	0,937205927
756	ENSG00000074842	-0,031028191	4,233876765	0,0146947	0,90456314
757	ENSG00000074935	-0,267026026	5,373769443	1,3228889	0,261836061
758	ENSG00000074964	0,192155711	4,955803807	0,9538106	0,338863238
759	ENSG00000075089	-0,228712912	4,531879267	0,7586234	0,39271681
760	ENSG00000075142	0,100501163	5,293817372	0,5559251	0,463396543
761	ENSG00000075151	0,02373798	6,253788564	0,0412581	0,840811899
762	ENSG00000075223	0,707303599	6,876524938	2,6797426	0,115177304
763	ENSG00000075234	-0,081477993	4,134086019	0,2915741	0,594378656
764	ENSG00000075239	-0,251763206	7,345690365	0,9378769	0,342850034
765	ENSG00000075240	-0,049222052	5,064718645	0,0538049	0,81861139

766	ENSG00000075275	0,381434472	3,725105347	2,6994139	0,113921772
767	ENSG00000075292	-0,073615319	7,763396319	0,2628389	0,61302469
768	ENSG00000075303	-0,179393645	4,428281123	0,9448171	0,34110547
769	ENSG00000075336	-0,625552833	5,696711031	9,3911777	0,005469888
770	ENSG00000075391	0,205739552	6,444724226	1,9998197	0,170616811
771	ENSG00000075407	0,096298759	5,318668364	0,687561	0,415447672
772	ENSG00000075413	-0,246458353	6,466993636	3,0257572	0,095219306
773	ENSG00000075415	-0,472307852	9,145848162	7,5148903	0,011579862
774	ENSG00000075420	0,031777731	7,120858763	0,0689791	0,795147851
775	ENSG00000075426	-0,071270645	6,790690951	0,0555243	0,815790648
776	ENSG00000075539	-0,157057639	7,221795805	1,1834947	0,287825185
777	ENSG00000075568	-0,025968084	5,983241499	0,0431962	0,837170411
778	ENSG00000075624	-0,193061419	9,437500022	0,4389383	0,51418827
779	ENSG00000075651	-0,3364111	6,717734939	2,4425404	0,131674432
780	ENSG00000075711	0,111989716	6,310843093	0,6497224	0,428399382
781	ENSG00000075785	0,001086804	6,664370081	6,852E-05	0,993466127
782	ENSG00000075790	-0,355544477	6,482418541	2,4562042	0,130651487
783	ENSG00000075826	-0,144189501	4,011069239	0,4813478	0,494726885
784	ENSG00000075856	0,261300108	4,930684351	3,8851996	0,060759825
785	ENSG00000075884	0,102851482	3,777714894	0,1922504	0,66512125
786	ENSG00000075914	0,032174864	4,262996436	0,0546896	0,839541684
787	ENSG00000075945	0,077309603	6,491635814	0,1710284	0,683011547
788	ENSG00000075975	-0,043607577	5,346321688	0,1060162	0,747645251
789	ENSG00000076003	0,113630549	4,142855349	0,5579187	0,462631399
790	ENSG00000076043	-0,25248741	5,680287023	2,4148561	0,133776251
791	ENSG00000076053	-0,075152816	4,568107319	0,15298	0,699286005
792	ENSG00000076067	0,139225537	5,865308849	0,5175447	0,479102484
793	ENSG00000076108	0,186418973	5,494368061	0,5158112	0,479831568
794	ENSG00000076242	-0,233030863	5,714565967	2,144907	0,156514166
795	ENSG00000076248	0,339781989	3,989158814	6,5028699	0,056708516
796	ENSG00000076321	0,155421975	4,617807902	1,4471532	0,241121638
797	ENSG00000076356	0,146166985	4,876730297	0,2146205	0,647502539
798	ENSG00000076513	-0,116488958	4,941257874	1,1149106	0,301891807
799	ENSG00000076554	0,075690803	3,976918171	0,2150756	0,647155962
800	ENSG00000076555	-0,334741113	7,526515996	0,7035541	0,410183552
801	ENSG00000076641	-0,436916392	5,250196499	6,0337764	0,021964665
802	ENSG00000076685	0,11236906	5,122164227	0,5027333	0,485392798
803	ENSG00000076706	0,065625219	7,093883502	0,1000128	0,754652636
804	ENSG00000076716	0,28440946	4,035611205	0,9315173	0,344459668
805	ENSG00000076770	0,261179698	6,354022722	0,7433085	0,397460394
806	ENSG00000077009	0,03716744	7,685089902	0,0148667	0,90400902
807	ENSG00000077044	0,125658263	5,781664069	0,2503883	0,621533131
808	ENSG00000077063	-0,467271641	3,686966057	4,3176493	0,049010378
809	ENSG00000077092	-0,328836696	5,147535807	3,4790592	0,074895608
810	ENSG00000077097	-0,011308102	7,534287899	0,0078843	0,930008089
811	ENSG00000077147	0,014706853	7,3332652	0,0093176	0,923931209
812	ENSG00000077157	-0,104488494	10,36342108	0,0870346	0,770615253
813	ENSG00000077232	0,007744848	6,637498322	0,0022682	0,962423554
814	ENSG00000077235	0,131260733	6,1218075	0,5095899	0,482463586
815	ENSG00000077238	-0,324126084	4,832369474	1,0622247	0,313373412
816	ENSG00000077254	0,03495738	6,205665048	0,0472259	0,829869647
817	ENSG00000077380	-0,160491231	6,497415295	1,2384855	0,277180628
818	ENSG00000077458	-0,014900182	4,120592252	0,0078313	0,997462664
819	ENSG00000077514	0,003722308	3,869164631	0,0012802	0,983253516
820	ENSG00000077522	-0,016078293	10,22412069	0,0033303	0,954476353
821	ENSG00000077549	-0,067133565	6,431830027	0,209419	0,651485034
822	ENSG00000077585	-0,228594237	4,88200437	3,3946604	0,078228403
823	ENSG00000077684	-0,113131073	5,551353817	0,498664	0,487145561
824	ENSG00000077721	-0,106487254	5,53805403	0,7173268	0,405676774

825	ENSG00000077782	0,025157016	6,697317399	0,0146154	0,904819565
826	ENSG00000077942	-0,129924596	8,319922635	0,18335	0,672473816
827	ENSG00000077943	0,729467662	4,83562651	2,1674658	0,154445522
828	ENSG00000078018	0,306954379	4,065490652	1,6963423	0,205596424
829	ENSG00000078043	-0,096909585	5,865384316	0,5864607	0,451514517
830	ENSG00000078053	-0,672073707	4,238181952	7,1250421	0,013666479
831	ENSG00000078061	-0,163877007	4,952611346	0,9972248	0,328323675
832	ENSG00000078070	-0,179367406	5,377171079	1,4728685	0,237150308
833	ENSG00000078114	-0,324980366	11,36851076	0,593699	0,448797301
834	ENSG00000078124	-0,013525726	4,654261985	0,0044963	0,947114849
835	ENSG00000078140	-0,10855594	5,979231524	0,7671359	0,390085394
836	ENSG00000078142	0,004007854	5,926432028	0,0009578	0,975574923
837	ENSG00000078177	0,173515171	4,884959589	1,0808133	0,309269634
838	ENSG00000078246	0,408451603	4,185311352	3,9077673	0,060112961
839	ENSG00000078269	0,3989031	5,056377219	5,1622318	0,032700718
840	ENSG00000078304	-0,015025411	6,867737599	0,0216187	0,884376598
841	ENSG00000078328	-0,537782436	5,237738316	3,4413844	0,076382386
842	ENSG00000078369	0,095308989	7,004527475	0,5369787	0,47102902
843	ENSG00000078403	-0,006084824	5,762347613	0,0015688	0,968745991
844	ENSG00000078596	-0,151417236	6,325908821	0,4652839	0,501944046
845	ENSG00000078618	-0,297257457	7,061467328	7,3016141	0,012662042
846	ENSG00000078668	-0,542150697	7,782731491	4,6096164	0,042513863
847	ENSG00000078674	-0,078934529	8,160544697	0,2752098	0,604836536
848	ENSG00000078687	-0,211104357	5,215976065	0,8351706	0,370217159
849	ENSG00000078699	0,20295353	4,563900196	1,2554174	0,274028169
850	ENSG00000078747	-0,022654084	6,538993305	0,0381813	0,846781283
851	ENSG00000078804	0,26172113	6,18001316	1,0198311	0,323015031
852	ENSG00000078808	0,093431372	5,96617905	0,3667626	0,550675487
853	ENSG00000078814	0,332025525	6,553435958	1,5667252	0,223203349
854	ENSG00000078967	-0,252784239	4,513749163	2,6662453	0,116048162
855	ENSG00000079102	-0,048228646	5,350854976	0,0849947	0,773240007
856	ENSG00000079134	-0,12110847	5,09605241	0,9484391	0,340165068
857	ENSG00000079150	0,01473127	3,846399194	0,0066614	0,935653467
858	ENSG00000079156	-0,229375096	4,523750801	1,3182209	0,262656484
859	ENSG00000079215	-1,187023322	6,211877206	14,860255	0,000800522
860	ENSG00000079246	-0,100172675	7,663649167	0,3659123	0,551126557
861	ENSG00000079277	-0,187652042	4,886333296	1,7825949	0,194806484
862	ENSG00000079308	0,125510733	8,893418346	0,1541739	0,698175947
863	ENSG00000079332	0,038512383	7,008054715	0,0665575	0,798695511
864	ENSG00000079335	-0,184452933	3,472780066	1,3387004	0,350584
865	ENSG00000079337	-0,209150688	4,504944247	0,8911026	0,354942708
866	ENSG00000079387	0,104877514	3,994860666	1,0136514	0,517715244
867	ENSG00000079435	0,824491643	4,009009367	2,3594475	0,138103715
868	ENSG00000079459	-0,102436845	5,717185769	0,6895039	0,414798985
869	ENSG00000079482	-0,345980723	4,412240212	4,7791701	0,039189421
870	ENSG00000079691	0,674656844	4,917724199	7,1622058	0,013453075
871	ENSG00000079739	-0,27401109	6,825660414	3,7250704	0,065919302
872	ENSG00000079785	-0,284712468	7,21242056	2,2017413	0,151364954
873	ENSG00000079805	-0,20366416	6,221313324	1,0286105	0,320985254
874	ENSG00000079819	0,386006875	7,055126887	3,5830119	0,070962225
875	ENSG00000079950	-0,035560488	6,577974897	0,0571217	0,813208585
876	ENSG00000079999	-0,017747677	4,900420964	0,0121762	0,913087653
877	ENSG00000080189	0,157665844	3,978830929	1,1150968	0,370737917
878	ENSG00000080200	0,032018286	6,98283484	0,0500496	0,824936495
879	ENSG00000080298	-0,054194197	4,642051754	0,0662691	0,799126501
880	ENSG00000080345	-0,009834321	6,881952289	0,0041239	0,949349055
881	ENSG00000080371	0,022898339	6,839001017	0,0239919	0,878247394
882	ENSG00000080493	0,897788025	6,380766946	3,6913072	0,067113641
883	ENSG00000080503	0,200839421	7,030717608	3,0859119	0,092169265

884	ENSG00000080546	-0,361507145	5,901140996	4,9589968	0,035979038
885	ENSG00000080573	-0,325068671	4,736124966	1,5245733	0,229334761
886	ENSG00000080608	-0,156319776	4,863435963	0,9625761	0,336697647
887	ENSG00000080709	-1,272793502	4,414615135	27,686932	2,40568E-05
888	ENSG00000080802	-0,044458568	5,045746897	0,2586778	0,710588166
889	ENSG00000080815	0,016477777	5,529733934	0,0222401	0,882739229
890	ENSG00000080822	-0,294832502	5,626650931	3,3671188	0,079413451
891	ENSG00000080823	-0,06681253	3,599411945	0,1343089	0,717339183
892	ENSG00000080824	-0,335897249	9,55196361	1,7237158	0,202102438
893	ENSG00000080839	0,113006107	3,560047356	0,7118068	0,568441867
894	ENSG00000080845	0,338963198	4,684710557	2,2118557	0,15047002
895	ENSG00000080854	-0,166438685	5,581385093	0,2562107	0,617529682
896	ENSG00000081014	-0,068406629	4,785463346	0,2645476	0,640302063
897	ENSG00000081019	0,099188513	4,961014259	0,6852377	0,416225408
898	ENSG00000081026	0,196666858	4,675543196	1,5447339	0,226376002
899	ENSG00000081041	0,832138389	4,540083132	2,0655672	0,16405967
900	ENSG00000081052	0,171730028	5,253900678	0,2834811	0,599510377
901	ENSG00000081087	-0,325678478	5,742603646	2,5964346	0,120677754
902	ENSG00000081154	-0,039559348	6,765727793	0,0660683	0,799424111
903	ENSG00000081177	-0,007248183	4,469155985	0,0063648	0,937096687
904	ENSG00000081189	-0,076494469	6,697331942	0,1697666	0,684115696
905	ENSG00000081237	0,133408603	5,711637861	0,1748221	0,679720551
906	ENSG00000081307	-0,199443468	5,507881175	1,9201047	0,179053134
907	ENSG00000081320	0,518010314	5,051158438	2,1271454	0,158166387
908	ENSG00000081377	0,072615398	5,300807595	0,3705322	0,54863023
909	ENSG00000081386	0,043771292	4,748250349	0,1171466	0,735232168
910	ENSG00000081665	0,032516209	5,160905985	0,0472918	0,82975302
911	ENSG00000081760	-0,873447124	4,271655683	9,8805609	0,004536367
912	ENSG00000081791	0,18424314	6,005606976	1,2849972	0,268593633
913	ENSG00000081803	-1,006865483	5,659226192	20,184034	0,000163177
914	ENSG00000081870	-0,257445757	3,913415948	2,1686448	0,183797191
915	ENSG00000081913	0,035984374	3,690175121	0,0468665	0,88771171
916	ENSG00000081923	0,006042421	4,432246659	0,0013884	0,970596248
917	ENSG00000082014	0,301990379	5,292349432	3,7876999	0,06388741
918	ENSG00000082068	-0,018904627	4,640430106	0,0337421	0,927302382
919	ENSG00000082074	0,161499674	4,085585697	0,2768687	0,603773991
920	ENSG00000082146	0,004378525	5,279328817	0,0005133	0,982119271
921	ENSG00000082153	0,114605462	7,294485933	0,2089788	0,651836749
922	ENSG00000082175	0,152098509	5,387608919	1,0738944	0,310779721
923	ENSG00000082212	-0,463923302	6,407956778	8,1755969	0,008844195
924	ENSG00000082213	-0,14932892	4,600719549	0,9670149	0,335608383
925	ENSG00000082258	-0,095732758	5,94758019	0,3728465	0,547414845
926	ENSG00000082269	-0,172007536	5,439055459	1,0370009	0,319061746
927	ENSG00000082397	-0,696472324	5,264942219	8,5315678	0,007664697
928	ENSG00000082438	0,758901395	5,917771723	3,0863669	0,092192957
929	ENSG00000082458	-0,114956641	4,669520548	0,8127041	0,376588444
930	ENSG00000082497	0,71109811	3,688772901	10,408087	0,003721702
931	ENSG00000082512	0,344846168	4,093074986	1,1702977	0,290498319
932	ENSG00000082515	-0,384487062	4,188403726	11,060646	0,016934381
933	ENSG00000082516	0,006713968	4,915356333	0,0033017	0,954670563
934	ENSG00000082641	-0,072106681	8,854676805	0,1035408	0,750513332
935	ENSG00000082701	0,01206397	6,065543232	0,0119159	0,91401471
936	ENSG00000082781	0,707818349	6,043079811	12,492948	0,001762661
937	ENSG00000082805	0,058999455	6,579818992	0,1425052	0,709247352
938	ENSG00000082898	0,094541073	7,142803643	0,5676442	0,458776832
939	ENSG00000082996	-0,07575408	5,942653007	0,1559904	0,696496582
940	ENSG00000083093	-0,050512482	3,537799355	0,2182144	0,809162362
941	ENSG00000083097	0,100682335	6,146203119	0,5040145	0,484823886
942	ENSG00000083099	-0,250337675	5,406675543	4,2136645	0,051548344

943	ENSG00000083123	-0,015144238	4,655243588	0,0144214	0,970173433
944	ENSG00000083168	-0,00488109	6,199103085	0,0011095	0,973713617
945	ENSG00000083223	0,024229599	5,436878134	0,036264	0,85062821
946	ENSG00000083290	0,533618834	4,885672919	18,787161	0,000240756
947	ENSG00000083312	0,001942883	7,680079684	9,439E-05	0,992331658
948	ENSG00000083444	-0,085587631	5,68668746	0,1095207	0,743671978
949	ENSG00000083520	-0,07668615	5,81346149	0,2566254	0,617246757
950	ENSG00000083535	0,002462154	5,002249593	0,0002368	0,987855019
951	ENSG00000083544	-0,173880022	6,039579315	0,8304662	0,371544855
952	ENSG00000083642	-0,057659136	5,957561577	0,1741501	0,68028831
953	ENSG00000083720	0,184724713	7,815226876	0,4920407	0,490021674
954	ENSG00000083750	-0,132642182	4,20807342	1,0091605	0,43592815
955	ENSG00000083799	0,33846903	6,243513033	6,7229109	0,016205796
956	ENSG00000083814	-0,004372128	3,785810038	0,001177	0,976753966
957	ENSG00000083828	-0,00791678	4,211815878	0,0026788	0,95916721
958	ENSG00000083844	0,288833876	5,365400563	4,7911329	0,038928723
959	ENSG00000083845	0,180441505	7,326712382	0,4742243	0,4979049
960	ENSG00000083857	0,145100446	8,045597159	0,7606506	0,392064188
961	ENSG00000083896	0,149620142	6,024041263	3,2573958	0,084102584
962	ENSG00000083937	-0,026898151	5,470010976	0,0271034	0,870665596
963	ENSG00000084070	0,044656081	4,56106179	0,03491	0,85341474
964	ENSG00000084072	-0,194892247	4,626678848	2,6059095	0,168703863
965	ENSG00000084073	-0,340779687	5,749947451	5,1502372	0,032884626
966	ENSG00000084090	-0,338633487	7,153531035	6,040893	0,021865345
967	ENSG00000084092	-0,051399291	3,727664855	0,1505708	0,787699506
968	ENSG00000084093	0,24023626	6,109747191	3,5752731	0,071202424
969	ENSG00000084112	0,201647813	5,498562393	0,9649655	0,3361107
970	ENSG00000084207	0,068487342	6,428944619	0,1031949	0,750915682
971	ENSG00000084234	0,01221848	8,396885328	0,0078188	0,930298539
972	ENSG00000084463	0,1530541	5,585505836	1,5857509	0,220460237
973	ENSG00000084623	-0,121265745	6,12588069	0,9834864	0,331573467
974	ENSG00000084636	0,724591375	5,063998773	8,4168475	0,008024722
975	ENSG00000084652	0,1087263	5,373892539	0,3588252	0,554985914
976	ENSG00000084674	0,545836807	6,623352159	1,4906724	0,234421448
977	ENSG00000084676	-0,142509136	6,450775933	1,2643178	0,272338009
978	ENSG00000084693	0,134249427	3,75009437	0,8839501	0,495748203
979	ENSG00000084710	0,40906284	3,91091317	2,1178238	0,159041896
980	ENSG00000084733	-0,230127952	6,547752557	3,4776366	0,074906358
981	ENSG00000084754	-0,25799905	8,295809358	2,3230707	0,140988338
982	ENSG00000084764	-0,286732606	4,38436093	2,3690856	0,137339163
983	ENSG00000084774	0,398054578	3,828746682	2,3808433	0,136413265
984	ENSG00000085063	-0,633894692	8,658667619	18,242036	0,000282121
985	ENSG00000085224	-0,070921633	7,724555161	0,2067069	0,653587514
986	ENSG00000085231	-0,277242033	3,979047971	1,1189539	0,301074788
987	ENSG00000085274	-0,186082058	4,626348314	0,8321044	0,371081727
988	ENSG00000085276	0,359960501	5,319771195	5,0891608	0,033839231
989	ENSG00000085365	-0,232476451	6,142041165	0,8380703	0,369402232
990	ENSG00000085377	-0,058144505	4,83705698	0,2704925	0,607930587
991	ENSG00000085382	-0,055298144	4,71888435	0,1891797	0,682042976
992	ENSG00000085415	-0,060454922	4,517196102	0,1210499	0,731047307
993	ENSG00000085433	-0,009862894	4,696077832	0,00619	0,960957911
994	ENSG00000085449	0,226065439	5,04768954	1,5789972	0,221457259
995	ENSG00000085491	-0,201199013	5,263902251	1,514275	0,230865039
996	ENSG00000085511	-0,152579768	5,715828891	1,7319508	0,201021183
997	ENSG00000085662	-1,434372183	7,612139216	18,659144	0,000251499
998	ENSG00000085719	-0,240260011	7,263535156	1,3573731	0,255877208
999	ENSG00000085721	0,225981011	5,535826318	2,643006	0,117529567
1000	ENSG00000085733	0,414307601	6,333645071	8,2502407	0,00857005
1001	ENSG00000085760	-0,562577684	5,710466054	5,9886446	0,02241215

1002	ENSG00000085788	0,10212398	5,896518296	1,1127792	0,30234373
1003	ENSG00000085831	-0,490027325	3,866253339	4,3520174	0,048190306
1004	ENSG00000085832	0,016141456	7,175376881	0,0228894	0,881053179
1005	ENSG00000085871	-0,254067663	5,140897384	2,4934163	0,127912924
1006	ENSG00000085978	0,051394098	4,536515007	0,1908333	0,833300465
1007	ENSG00000085982	0,027997032	5,353900204	0,066257	0,799256446
1008	ENSG00000085998	-0,090021364	5,989201929	0,3132444	0,581080131
1009	ENSG00000086015	-0,315691689	6,508707093	1,6535665	0,211208299
1010	ENSG00000086061	0,068475523	6,646486602	0,1121018	0,740783392
1011	ENSG00000086062	0,138452021	6,021704004	0,3181359	0,578162734
1012	ENSG00000086065	-0,051587033	5,827508527	0,0553877	0,816012983
1013	ENSG00000086102	0,040037141	5,661381878	0,142025	0,709702079
1014	ENSG00000086189	-0,014565443	4,729073119	0,0107122	0,967134149
1015	ENSG00000086200	-0,006673149	5,279314597	0,0022378	0,962675754
1016	ENSG00000086232	0,080887329	5,640817277	0,4242895	0,521206605
1017	ENSG00000086289	-0,146972364	4,510630038	0,819938	0,374541316
1018	ENSG00000086475	-0,008546337	4,863384104	0,0100634	0,97747326
1019	ENSG00000086544	-0,339291049	4,153318795	1,6691999	0,209135413
1020	ENSG00000086589	0,21527988	4,837930488	4,2515245	0,091192284
1021	ENSG00000086598	-0,029036489	7,191081699	0,0315281	0,860614304
1022	ENSG00000086619	-0,07616785	4,499021375	0,2088393	0,651944826
1023	ENSG00000086666	-0,13862711	6,166193967	0,7636011	0,39119318
1024	ENSG00000086712	-0,057554685	4,36325451	0,1764162	0,711233015
1025	ENSG00000086758	0,170118199	8,125873721	0,642873	0,430839119
1026	ENSG00000086827	-0,173797966	4,238741239	2,1210447	0,280630649
1027	ENSG00000086848	-0,083516584	3,839351425	0,1559197	0,696561722
1028	ENSG00000086991	1,113328896	4,257035552	12,646349	0,001671757
1029	ENSG00000087008	-0,423215515	4,375077617	3,0786133	0,092578217
1030	ENSG00000087053	-0,106425498	5,287321916	0,6152977	0,440742464
1031	ENSG00000087074	0,318653951	6,003552728	2,6362962	0,118008252
1032	ENSG00000087086	0,369402973	9,474285628	1,4211172	0,245316051
1033	ENSG00000087087	0,05562859	5,012860069	0,1161916	0,736281384
1034	ENSG00000087095	-0,17853545	4,841537531	2,2479981	0,147276589
1035	ENSG00000087111	-0,255661516	4,682683497	2,4670609	0,129845404
1036	ENSG00000087116	0,571592537	4,128966061	2,8101671	0,107144756
1037	ENSG00000087152	0,117998152	4,441971868	0,4194095	0,523613149
1038	ENSG00000087157	-0,343855065	3,859127012	2,9317871	0,100238075
1039	ENSG00000087191	-0,224492845	5,872980701	1,571074	0,222582623
1040	ENSG00000087206	0,045873903	5,011916919	0,1898432	0,745940416
1041	ENSG00000087245	-0,353223748	7,080481066	2,6726347	0,115634951
1042	ENSG00000087253	-0,106021208	4,474434893	0,2458545	0,624691317
1043	ENSG00000087258	-0,325919653	5,219287802	0,8351547	0,370221644
1044	ENSG00000087263	0,077067449	4,895633848	0,4212316	0,534421995
1045	ENSG00000087266	-0,196415858	4,542079373	0,8283245	0,372151571
1046	ENSG00000087269	0,037238738	4,228914935	0,0459223	0,832196398
1047	ENSG00000087274	0,074320184	7,186383872	0,1704407	0,683525214
1048	ENSG00000087299	-0,30229869	3,686491612	3,2169241	0,104325245
1049	ENSG00000087301	0,426382889	4,351658875	3,8607946	0,061558543
1050	ENSG00000087302	-0,399075278	6,694065914	4,8693533	0,037540917
1051	ENSG00000087303	0,147031232	4,459637067	0,4340244	0,516530853
1052	ENSG00000087338	0,275922404	3,98825936	2,7342385	0,111738182
1053	ENSG00000087365	0,145789138	6,258611078	0,6780265	0,418682809
1054	ENSG00000087448	0,04258196	4,535007396	0,0841147	0,77437932
1055	ENSG00000087460	0,069235725	9,334585985	0,15454	0,69782405
1056	ENSG00000087470	-0,249717717	6,691581412	2,45795	0,130521469
1057	ENSG00000087502	-0,393456805	6,294691063	2,1575029	0,155355042
1058	ENSG00000087884	-0,399610567	3,941133041	7,2267288	0,023203763
1059	ENSG00000087995	-0,134528152	4,210009937	1,4877235	0,402511785
1060	ENSG00000088179	0,026923885	6,11530148	0,0254161	0,874719643

1061	ENSG00000088205	-0,026424492	5,89599252	0,0332413	0,856916744
1062	ENSG00000088247	0,265527623	4,815571634	1,6871365	0,206788308
1063	ENSG00000088256	0,008851951	4,932028291	0,0020952	0,963883974
1064	ENSG00000088280	-0,192241685	5,089049795	1,3524487	0,256717304
1065	ENSG00000088298	-0,077808294	3,866695922	0,1245184	0,727382536
1066	ENSG00000088367	0,348209101	4,397537185	2,3237578	0,140979301
1067	ENSG00000088387	-0,090933101	6,48722595	0,3019188	0,587952342
1068	ENSG00000088448	-0,018732243	6,455715616	0,0102931	0,92006433
1069	ENSG00000088538	-0,009818838	4,107609164	0,0015556	0,968877102
1070	ENSG00000088543	-0,486315437	4,785689397	6,8529653	0,015347899
1071	ENSG00000088682	-0,292365967	6,079800602	2,8442133	0,105156918
1072	ENSG00000088808	-0,316280496	5,572104638	2,3434949	0,139380338
1073	ENSG00000088812	0,110000905	5,990829586	1,0223168	0,322402374
1074	ENSG00000088827	-0,804057086	4,076593075	9,0195733	0,006319909
1075	ENSG00000088832	-0,162729332	6,414366464	1,7011103	0,204938098
1076	ENSG00000088833	-0,076376206	5,411838218	0,3878393	0,539512835
1077	ENSG00000088854	0,194859395	5,838944095	1,2444507	0,276079686
1078	ENSG00000088888	0,00953509	6,218380308	0,0017408	0,967078232
1079	ENSG00000088930	-0,222930595	6,347981362	2,5301372	0,125238327
1080	ENSG00000088970	0,225813907	4,689901668	2,3314664	0,140344011
1081	ENSG00000088986	0,089948188	6,097394287	0,1644918	0,688784228
1082	ENSG00000089006	0,215170523	5,8593355	3,1928776	0,087038013
1083	ENSG00000089009	0,063167749	8,754889363	0,0683588	0,796057285
1084	ENSG00000089022	-0,041636569	4,77935645	0,1557158	0,696737108
1085	ENSG00000089048	-0,038088764	4,837821957	0,0491949	0,826418274
1086	ENSG00000089050	0,167606847	3,983483155	0,8975473	0,353240976
1087	ENSG00000089053	-0,134372103	6,359536465	2,2281383	0,148995766
1088	ENSG00000089057	0,298537461	4,923232268	2,4256913	0,132948926
1089	ENSG00000089060	-0,277126944	3,881312568	2,5585297	0,123282344
1090	ENSG00000089063	-0,080107447	6,083221465	0,4741292	0,497923815
1091	ENSG00000089123	0,297037283	3,858132331	4,0998216	0,088019737
1092	ENSG00000089154	0,165532256	5,870098935	0,679867	0,418061011
1093	ENSG00000089157	0,050054427	9,026816937	0,0376471	0,847848694
1094	ENSG00000089159	-0,170066962	5,018588735	0,4931488	0,489538443
1095	ENSG00000089177	0,134134948	5,020376764	1,5184191	0,286033354
1096	ENSG00000089195	-0,183221541	3,936341064	2,3636961	0,304278521
1097	ENSG00000089199	0,616865005	6,338517317	1,6748291	0,208395246
1098	ENSG00000089220	-0,1577885	8,02038795	1,4592814	0,239217938
1099	ENSG00000089225	0,104425337	6,484366087	0,2263097	0,638739273
1100	ENSG00000089234	0,081348637	4,373190757	0,5900468	0,52137293
1101	ENSG00000089248	0,020696946	5,369887232	0,0241887	0,877751345
1102	ENSG00000089280	0,106813015	6,544774115	0,3498593	0,559934057
1103	ENSG00000089289	-0,02020112	5,444928803	0,0118001	0,914434976
1104	ENSG00000089335	-0,182547574	5,902369422	1,1162125	0,301653962
1105	ENSG00000089472	0,290549074	5,610390225	2,7034824	0,113664106
1106	ENSG00000089486	-0,212565341	4,614171081	1,0762556	0,310269032
1107	ENSG00000089597	0,212563198	6,880186127	1,3193854	0,262451511
1108	ENSG00000089682	-0,055186877	4,216821905	0,1806368	0,789642147
1109	ENSG00000089693	-0,176236949	5,809052348	1,6104783	0,217012361
1110	ENSG00000089737	-0,056608685	7,465245343	0,1573029	0,69527754
1111	ENSG00000089775	0,225564005	4,61445141	1,8852284	0,182916465
1112	ENSG00000089818	-0,014606126	4,715076464	0,0183864	0,911873574
1113	ENSG00000089876	-0,362203236	6,002072879	2,078639	0,162786782
1114	ENSG00000089902	0,000204143	4,539632676	1,468E-06	0,999043763
1115	ENSG00000089916	-0,016113843	6,670117712	0,0163295	0,899418932
1116	ENSG00000090006	0,504827823	6,714049214	5,7809829	0,024606215
1117	ENSG00000090013	-0,212388143	4,834120906	0,8002396	0,380243169
1118	ENSG00000090054	0,077241738	5,481348714	0,3178431	0,578320976
1119	ENSG00000090060	-0,140126822	7,454018499	1,1597063	0,292600444

1120	ENSG00000090097	-0,043081367	4,763465926	0,0497939	0,825382674
1121	ENSG00000090263	-0,642504003	5,329517233	10,009373	0,004320789
1122	ENSG00000090266	-0,583994952	6,624142318	9,4634369	0,005319659
1123	ENSG00000090273	-0,302057414	5,839857303	2,5420968	0,12443206
1124	ENSG00000090316	0,076328055	4,834180322	0,3797096	0,65220607
1125	ENSG00000090339	-0,142557014	4,416052068	0,2600676	0,614909224
1126	ENSG00000090372	0,106136605	4,463962599	0,4669649	0,58215899
1127	ENSG00000090376	0,086553863	5,697971115	0,0680649	0,796485856
1128	ENSG00000090382	0,150527771	5,110589681	0,1639629	0,689257177
1129	ENSG00000090432	-0,249534882	4,660121312	2,3118956	0,141950809
1130	ENSG00000090470	0,266847626	3,895460406	6,6661263	0,136116976
1131	ENSG00000090487	-0,056260999	5,286274039	0,3127461	0,582037814
1132	ENSG00000090520	-0,103904755	4,635687034	0,2839438	0,599214406
1133	ENSG00000090565	0,490578561	4,51303601	6,9931567	0,014454582
1134	ENSG00000090612	0,064027264	4,907935542	0,1123077	0,740554558
1135	ENSG00000090615	0,461662585	6,06323374	5,3143969	0,030464852
1136	ENSG00000090621	0,081629954	6,837507991	0,2484227	0,622897937
1137	ENSG00000090659	-1,265605233	4,008935103	22,298005	9,19837E-05
1138	ENSG00000090686	0,062309797	5,901057831	0,3174411	0,578556434
1139	ENSG00000090776	-0,020402474	3,903423091	0,0076317	0,931138367
1140	ENSG00000090857	0,295003336	5,570998494	1,4325953	0,243474273
1141	ENSG00000090861	-0,093813379	6,106784994	0,319395	0,5774166
1142	ENSG00000090863	0,221302317	7,270139537	1,4128289	0,246657128
1143	ENSG00000090905	0,002695449	6,453150584	0,0002434	0,987687208
1144	ENSG00000090989	-0,139779549	5,443589751	1,2993761	0,26596247
1145	ENSG00000091009	0,076680996	4,663262902	0,3855478	0,540703536
1146	ENSG00000091039	0,117108845	6,762037638	0,2723584	0,60671989
1147	ENSG00000091136	0,245441355	7,440537934	1,0468905	0,316814812
1148	ENSG00000091140	-0,380118942	7,432202881	2,7944286	0,108078478
1149	ENSG00000091157	-0,084469887	5,652687973	0,6923827	0,4138407
1150	ENSG00000091164	-0,354874003	6,083114076	6,655125	0,016688243
1151	ENSG00000091317	0,12109496	5,367183746	0,4579824	0,505285524
1152	ENSG00000091409	-0,374480944	6,619795938	3,9642213	0,058426379
1153	ENSG00000091428	-0,102576384	4,816114555	0,276985	0,603698396
1154	ENSG00000091436	-0,030498218	8,593280017	0,0191185	0,891224819
1155	ENSG00000091482	-0,908276911	7,816403045	7,1843488	0,013327678
1156	ENSG00000091483	-0,427559675	7,171883617	4,1129305	0,054234881
1157	ENSG00000091513	0,282053586	5,056509304	0,1660854	0,687364631
1158	ENSG00000091527	0,087924296	7,170883834	0,4149098	0,525807279
1159	ENSG00000091542	-0,123348782	6,433560376	0,6320768	0,434683267
1160	ENSG00000091592	0,590906861	4,488650616	5,1454959	0,032957642
1161	ENSG00000091640	-0,27965572	5,763653156	2,5458725	0,124166777
1162	ENSG00000091656	0,922867553	3,944171632	6,778426	0,015847647
1163	ENSG00000091732	-0,223984711	3,924238037	4,0403272	0,191929269
1164	ENSG00000091831	0,428993302	3,980296224	4,5825731	0,0430729
1165	ENSG00000091879	-0,192011749	3,991490835	0,6614167	0,424359287
1166	ENSG00000091972	1,202834389	4,56954564	4,0490044	0,055993423
1167	ENSG00000091986	0,573414757	9,122308702	2,0014858	0,170476808
1168	ENSG00000092010	0,168410843	5,546980754	1,4282236	0,2441406
1169	ENSG00000092020	-0,263005014	4,577340093	0,9382908	0,342745624
1170	ENSG00000092054	1,180522596	11,6605036	3,9267561	0,059539566
1171	ENSG00000092094	0,199651365	4,381437391	2,3195996	0,193493475
1172	ENSG00000092108	-0,109454011	5,997907661	0,3062998	0,585274229
1173	ENSG00000092140	-0,062598822	4,712924012	0,1508032	0,701322944
1174	ENSG00000092148	-0,030684565	8,07589203	0,0743578	0,787507395
1175	ENSG00000092199	-0,015218322	7,968339962	0,0135617	0,90829472
1176	ENSG00000092201	0,190079419	6,17201527	4,2268266	0,051213657
1177	ENSG00000092203	-0,047646816	5,902993303	0,1890248	0,667747909
1178	ENSG00000092330	-0,052296082	4,839603062	0,1356618	0,715975764

1179	ENSG00000092421	-0,413267251	4,592171448	4,3513707	0,048205593
1180	ENSG00000092439	-0,000678444	6,943422602	2,065E-05	0,996413503
1181	ENSG00000092531	-0,020366274	4,648131531	0,0225275	0,977001474
1182	ENSG00000092621	0,929238292	4,079989335	5,5373417	0,027491106
1183	ENSG00000092820	0,384011538	7,62799705	3,8610155	0,061551655
1184	ENSG00000092841	-0,154172445	8,283569544	0,4955426	0,488497409
1185	ENSG00000092847	0,117663408	5,817254078	0,3772306	0,545087953
1186	ENSG00000092871	0,261994626	3,596844743	2,7184101	0,194233118
1187	ENSG00000092931	-0,217844874	4,806977544	3,1032383	0,091314852
1188	ENSG00000092964	0,069304976	7,578338022	0,1419148	0,709821229
1189	ENSG00000092978	-0,070344831	4,730952713	0,3363234	0,636343164
1190	ENSG00000093000	0,191129025	5,019582931	2,4099782	0,134103579
1191	ENSG00000093010	0,034035719	4,553261387	0,0232411	0,880154749
1192	ENSG00000093072	0,206586003	5,035855251	0,4192869	0,523673314
1193	ENSG00000093144	-0,175140398	5,836754126	0,460257	0,504240387
1194	ENSG00000093167	-0,179220255	7,420139043	0,7734788	0,388195644
1195	ENSG00000093183	0,121424314	5,260631028	1,0749051	0,310528943
1196	ENSG00000094631	0,173325992	4,769829561	0,9687968	0,335172512
1197	ENSG00000094841	-0,088751077	3,874685758	0,6007274	0,628313705
1198	ENSG00000094880	-0,051103331	4,658135915	0,1712019	0,771211886
1199	ENSG00000094914	0,063255214	4,613293073	0,2316557	0,728572409
1200	ENSG00000094916	0,214116372	6,990933344	1,7522575	0,198537478
1201	ENSG00000094963	0,922876964	6,868477418	7,2838784	0,012779777
1202	ENSG00000094975	-0,021947533	5,936679707	0,019911	0,889008449
1203	ENSG00000095002	-0,033935967	4,649587741	0,0305958	0,862668502
1204	ENSG00000095015	0,068840379	5,359721859	0,1329282	0,718729728
1205	ENSG00000095066	0,209397268	4,294152653	0,7659145	0,390488075
1206	ENSG00000095139	0,186815619	6,814683528	3,9508196	0,058779415
1207	ENSG00000095203	-0,179755447	4,98751743	0,4392711	0,514030272
1208	ENSG00000095209	-0,833589402	5,583297127	7,3623669	0,012365254
1209	ENSG00000095261	0,033432002	4,573240622	0,1049471	0,748875008
1210	ENSG00000095303	1,982997281	4,752057881	13,53391	0,001236858
1211	ENSG00000095319	0,07751542	5,010260838	0,1852447	0,670891068
1212	ENSG00000095321	-0,127384622	5,123268844	0,4250801	0,520844466
1213	ENSG00000095380	-0,349121949	3,994047515	6,6296449	0,043623856
1214	ENSG00000095485	0,00343315	4,323264689	0,0010823	0,974036952
1215	ENSG00000095564	0,127799548	6,276035097	0,4571957	0,505647883
1216	ENSG00000095574	0,091530376	4,53937361	0,465237	0,501941917
1217	ENSG00000095637	0,029087594	9,390170399	0,0135334	0,908393571
1218	ENSG00000095739	0,413150553	4,812420827	6,0991856	0,021333661
1219	ENSG00000095787	0,003171957	6,872506841	0,0011101	0,973706465
1220	ENSG00000095794	-0,361902378	5,147550917	2,0622832	0,164381343
1221	ENSG00000095951	-0,02873904	5,407210605	0,0517211	0,82208777
1222	ENSG00000096060	-0,942089026	6,557474048	3,9042927	0,060218559
1223	ENSG00000096063	-0,220107917	4,43156145	2,9292859	0,138756263
1224	ENSG00000096070	0,007379586	4,300970127	0,0010342	0,974621168
1225	ENSG00000096093	0,385600704	4,430375508	4,7415699	0,039900634
1226	ENSG00000096384	0,170765718	9,397086502	1,0114032	0,324944211
1227	ENSG00000096401	-0,049924179	6,121739497	0,0729621	0,789468988
1228	ENSG00000096433	-0,015140616	4,234570087	0,0029148	0,957408288
1229	ENSG00000096696	-0,278207797	9,111969479	0,9537516	0,338877889
1230	ENSG00000096717	0,195274233	4,913996673	1,9378117	0,177129577
1231	ENSG00000096746	0,029950955	5,911786656	0,0486579	0,827345461
1232	ENSG00000096872	0,107546832	4,181391989	0,3938147	0,536452233
1233	ENSG00000096968	0,294027653	5,995312266	0,9780668	0,332917541
1234	ENSG00000097007	-0,320966062	5,486064278	1,7704442	0,196306463
1235	ENSG00000097033	0,02975316	7,394026602	0,04136	0,840618211
1236	ENSG00000097096	-0,127024886	4,906945274	0,4712415	0,499246172
1237	ENSG00000099139	-0,322258066	4,717215446	2,0383174	0,166752081

1238	ENSG00000099194	-0,23211631	9,07858745	0,116355	0,736103392
1239	ENSG00000099204	-0,206309759	9,41956903	0,7498246	0,395431708
1240	ENSG00000099219	0,186529249	5,592478202	0,8830846	0,357076218
1241	ENSG00000099246	-0,1810065	6,547529948	0,7316543	0,401127862
1242	ENSG00000099250	0,125819385	7,400227083	0,5538137	0,464262957
1243	ENSG00000099260	-0,441380909	6,966652086	7,5448372	0,011457818
1244	ENSG00000099290	-0,595988204	7,379312749	5,3830002	0,029513155
1245	ENSG00000099331	0,094953022	4,875104199	0,200665	0,658355385
1246	ENSG00000099341	0,060781592	6,667114843	0,0958453	0,759647539
1247	ENSG00000099622	0,10005559	7,470658991	0,5271015	0,475091091
1248	ENSG00000099725	0,493719688	3,619438145	0,7984985	0,380753244
1249	ENSG00000099783	0,031443457	6,366528723	0,0704099	0,793085035
1250	ENSG00000099785	-0,061243024	5,114573443	0,0780664	0,782420132
1251	ENSG00000099795	-0,195672691	5,641103843	0,5657163	0,459557934
1252	ENSG00000099797	-0,238257731	4,756447004	1,4714762	0,237365424
1253	ENSG00000099810	-0,009791089	4,867978202	0,0071844	0,960301702
1254	ENSG00000099817	-0,055818892	5,405877129	0,0885815	0,768647125
1255	ENSG00000099860	-0,098747492	5,931299487	0,1110213	0,741988078
1256	ENSG00000099875	-0,121868688	5,664693505	0,369006	0,549468791
1257	ENSG00000099901	0,106719358	4,258530897	0,5655473	0,533510965
1258	ENSG00000099910	0,072850677	3,841529287	0,177587	0,757164344
1259	ENSG00000099917	0,033407138	4,590352795	0,0316843	0,860273308
1260	ENSG00000099940	-0,071494469	4,668852612	0,2641953	0,612118003
1261	ENSG00000099942	-0,035567273	6,152554599	0,0881202	0,769222792
1262	ENSG00000099954	-0,18575323	3,913024749	0,4719524	0,498925947
1263	ENSG00000099956	-0,186731979	4,592197309	0,9783525	0,332848396
1264	ENSG00000099968	-0,175404036	6,074512829	1,7450712	0,199382996
1265	ENSG00000099991	-0,098573109	5,308881474	0,2399726	0,628843501
1266	ENSG00000099995	0,133182146	5,490817209	0,4201214	0,523264121
1267	ENSG00000100014	0,225498737	4,655363213	3,2160786	0,112008493
1268	ENSG00000100023	0,088508297	4,362464689	0,2563137	0,617459371
1269	ENSG00000100028	-0,053475609	4,949581343	0,1389612	0,712707844
1270	ENSG00000100029	0,005794677	4,284711859	0,0010564	0,974350015
1271	ENSG00000100030	0,153652593	6,911480415	3,0214772	0,095428947
1272	ENSG00000100034	0,237498479	4,579877899	1,8343075	0,188713307
1273	ENSG00000100055	-0,26914585	3,937140852	1,0634777	0,313094475
1274	ENSG00000100077	-0,566205167	5,435255811	4,1735294	0,052625443
1275	ENSG00000100083	0,113847474	4,327869044	0,5257307	0,560932306
1276	ENSG00000100097	-0,032905141	7,344871932	0,0137242	0,907752943
1277	ENSG00000100099	0,058763889	4,399149968	0,1468472	0,757006547
1278	ENSG00000100100	0,748925431	4,717118774	9,1679204	0,005964299
1279	ENSG00000100104	-0,237994266	3,798384603	3,5317091	0,19290799
1280	ENSG00000100105	0,505715242	3,698502069	6,2019112	0,020382947
1281	ENSG00000100106	0,021302154	5,800804772	0,0166631	0,898406596
1282	ENSG00000100109	0,107374183	4,234528209	0,9198172	0,550868495
1283	ENSG00000100129	0,315572757	6,708906347	2,5921049	0,120971963
1284	ENSG00000100138	-0,401920762	6,329955473	4,9778595	0,035659752
1285	ENSG00000100154	0,239269343	5,018558166	1,3152565	0,26317923
1286	ENSG00000100170	-0,797809628	6,022437748	4,0990455	0,054611456
1287	ENSG00000100196	0,670756268	3,998348099	14,808375	0,000813996
1288	ENSG00000100201	0,078268775	8,956849475	0,1862233	0,670063433
1289	ENSG00000100207	0,149863812	4,616008038	0,5347256	0,471975962
1290	ENSG00000100211	-0,075784811	3,898743324	0,3139112	0,673229778
1291	ENSG00000100216	-0,137399801	5,252168499	1,8621117	0,213403996
1292	ENSG00000100219	-0,08867254	6,391481065	0,2560355	0,617649302
1293	ENSG00000100220	-0,03546019	5,63488073	0,1178132	0,734510151
1294	ENSG00000100221	0,358018844	5,151961177	3,8048355	0,063332582
1295	ENSG00000100225	0,161293201	5,956465509	2,5532587	0,123602598
1296	ENSG00000100226	0,247797425	4,743371916	1,5957363	0,219103202

1297	ENSG00000100227	0,145645352	5,185975106	0,6911956	0,414264895
1298	ENSG00000100234	-0,007473513	9,434858165	0,000778	0,977986567
1299	ENSG00000100239	-0,147622069	5,427618188	0,9094025	0,35014077
1300	ENSG00000100241	0,2572342	4,905240361	1,4226266	0,24507283
1301	ENSG00000100242	0,013294046	5,546087967	0,0027773	0,958423852
1302	ENSG00000100243	-0,051530393	7,371185528	0,0820897	0,777037846
1303	ENSG00000100266	-0,067096413	5,697635768	0,2099703	0,651063895
1304	ENSG00000100280	-0,009187513	5,120493079	0,0020427	0,964339333
1305	ENSG00000100281	0,060696589	4,104171741	0,3794723	0,706027182
1306	ENSG00000100284	0,086115735	4,983127513	0,2357304	0,631877764
1307	ENSG00000100292	-1,116007984	4,509581283	5,5251623	0,027644918
1308	ENSG00000100296	-0,2802694	4,717144778	4,540052	0,043929742
1309	ENSG00000100300	0,001671487	4,563428679	5,382E-05	0,994209377
1310	ENSG00000100307	0,162842594	5,529843999	0,8553274	0,364605511
1311	ENSG00000100316	0,416606897	8,920237199	2,1188823	0,158942192
1312	ENSG00000100320	0,039933317	6,900763086	0,1377066	0,713939664
1313	ENSG00000100321	-0,224996835	4,711544317	1,3088398	0,264315409
1314	ENSG00000100325	0,101567757	4,783118012	0,3915071	0,537638443
1315	ENSG00000100330	0,339171086	3,83982552	5,4590892	0,066270437
1316	ENSG00000100335	-0,113465706	4,717452169	0,7382809	0,399005793
1317	ENSG00000100342	-0,022681455	4,857089603	0,0058065	0,939915216
1318	ENSG00000100345	0,140267663	8,086512254	0,355691	0,556705937
1319	ENSG00000100347	-0,298650958	5,338969474	6,5785667	0,017252358
1320	ENSG00000100348	-0,236154329	5,445440271	2,1312225	0,157785279
1321	ENSG00000100353	0,076621421	6,431083251	0,2659511	0,610952048
1322	ENSG00000100354	0,170533483	7,038561823	0,6261327	0,436822853
1323	ENSG00000100360	0,177823976	3,780845772	1,0188159	0,394617788
1324	ENSG00000100364	0,063007216	4,556753391	0,1222711	0,729750353
1325	ENSG00000100376	0,188039533	4,345746185	0,4952036	0,488644609
1326	ENSG00000100380	0,127005816	7,498828797	0,9500116	0,339772973
1327	ENSG00000100387	-0,222219109	5,462442732	1,9266571	0,178360429
1328	ENSG00000100393	0,20225515	5,89975363	0,7300544	0,401635312
1329	ENSG00000100395	0,046276132	3,96137956	0,0743744	0,857607185
1330	ENSG00000100401	-0,054835938	4,675876358	0,0767675	0,784189671
1331	ENSG00000100403	0,327311923	5,387009479	2,0883699	0,161846958
1332	ENSG00000100410	-0,243882318	3,808728706	3,2858254	0,19276724
1333	ENSG00000100412	0,080985505	7,650268476	0,1119063	0,741000929
1334	ENSG00000100416	-0,250240563	4,649375409	2,8272224	0,106124485
1335	ENSG00000100418	-0,747703338	6,118093114	12,973919	0,001494337
1336	ENSG00000100422	0,161303482	6,13177941	1,6775098	0,207999729
1337	ENSG00000100425	0,167691361	4,089626705	1,0642096	0,369624355
1338	ENSG00000100426	-0,08265478	3,725041436	0,2460304	0,624568109
1339	ENSG00000100439	-0,009054402	4,105515339	0,0031846	0,955482316
1340	ENSG00000100441	0,177777294	5,084559796	0,8596565	0,363416318
1341	ENSG00000100442	-0,607250397	5,454406328	13,94729	0,001077976
1342	ENSG00000100461	0,032573618	5,415749197	0,0694801	0,794422928
1343	ENSG00000100462	-0,002579929	4,25538676	0,0004702	0,982886254
1344	ENSG00000100478	-0,098347893	4,298332477	0,7657551	0,501025674
1345	ENSG00000100485	0,141920785	6,300188919	1,3174085	0,262759047
1346	ENSG00000100503	-0,014777319	7,532124829	0,0061015	0,938411403
1347	ENSG00000100504	0,36322442	5,072291202	0,7122395	0,407351757
1348	ENSG00000100519	-0,417097074	6,417275989	2,5921791	0,120966913
1349	ENSG00000100523	-0,087041589	5,206073001	0,4447499	0,511419196
1350	ENSG00000100528	-0,090165221	6,203384267	0,2160198	0,64643837
1351	ENSG00000100532	-0,200867237	4,331167846	1,2485832	0,275304261
1352	ENSG00000100554	-0,377715915	6,630919615	2,6121273	0,11961844
1353	ENSG00000100567	-0,524099351	5,936294333	13,320283	0,001324989
1354	ENSG00000100568	-0,100110048	5,76724676	0,6439563	0,430428048
1355	ENSG00000100578	-0,045677087	5,017389249	0,1284325	0,723304087

1356	ENSG00000100580	0,215386305	4,736487131	2,3527272	0,138603876
1357	ENSG00000100591	-0,340420719	6,190080752	3,8082126	0,063223882
1358	ENSG00000100592	-0,024744574	6,789169743	0,0301692	0,863614516
1359	ENSG00000100596	-0,007890753	5,653377767	0,0024705	0,960785428
1360	ENSG00000100600	-0,300183656	6,432810327	3,5054944	0,073872181
1361	ENSG00000100603	-0,068460601	5,464185181	0,2824241	0,600169988
1362	ENSG00000100605	-0,121047253	5,402733522	0,3016086	0,58814292
1363	ENSG00000100612	-0,23152368	4,957459745	3,2792968	0,083132192
1364	ENSG00000100614	-0,132299015	7,286927937	0,6209752	0,438692801
1365	ENSG00000100626	-0,597589237	5,512408788	4,1747421	0,052593794
1366	ENSG00000100628	-0,619969746	5,985474999	4,6674474	0,041345577
1367	ENSG00000100632	-0,128379397	5,211492455	0,7507894	0,395127553
1368	ENSG00000100644	0,011464201	6,783278117	0,0041925	0,948930491
1369	ENSG00000100647	0,140684564	4,954598985	0,538259	0,470532273
1370	ENSG00000100650	0,030857648	7,556450161	0,0592073	0,80989235
1371	ENSG00000100664	0,062320913	7,935183841	0,2335609	0,633426852
1372	ENSG00000100697	-0,079449419	6,827474657	0,4151753	0,525676023
1373	ENSG00000100711	0,293122583	4,123546636	3,1990619	0,093564639
1374	ENSG00000100714	0,19848416	5,580368716	1,6494	0,211765077
1375	ENSG00000100722	0,003330627	5,601580652	0,0008339	0,977210134
1376	ENSG00000100731	-0,067752471	7,308297324	0,3678057	0,550093291
1377	ENSG00000100744	-0,152825641	4,038065448	0,4559975	0,506200643
1378	ENSG00000100749	-0,01668323	3,778845156	0,0050459	0,943981507
1379	ENSG00000100764	-0,338215775	5,535403737	2,2909795	0,143683333
1380	ENSG00000100784	-0,271266622	4,563187858	1,7388268	0,200205224
1381	ENSG00000100796	0,026492833	5,905015654	0,0505139	0,824139674
1382	ENSG00000100804	-0,307978208	5,832442747	5,4908364	0,028050582
1383	ENSG00000100811	0,117171291	6,022311671	0,9088795	0,350242593
1384	ENSG00000100813	0,026851731	6,374610453	0,0264824	0,872142268
1385	ENSG00000100814	-0,326559995	5,712915167	4,8766788	0,037407142
1386	ENSG00000100815	0,032619598	6,880874265	0,0730837	0,789289762
1387	ENSG00000100823	-0,066713582	5,173457054	0,2135703	0,648295156
1388	ENSG00000100836	-0,004719739	5,037677273	0,0008555	0,976916683
1389	ENSG00000100852	-0,300095066	8,471055899	1,1063576	0,303748388
1390	ENSG00000100865	-0,203580123	4,146901999	2,0684293	0,230028871
1391	ENSG00000100883	-0,097164046	5,583481033	0,4372717	0,514978593
1392	ENSG00000100888	0,117186006	6,119686927	0,6688508	0,421782598
1393	ENSG00000100897	-0,060970784	5,603047127	0,2476948	0,623388748
1394	ENSG00000100906	0,49088426	6,091442364	3,0269136	0,095195247
1395	ENSG00000100911	-0,098286443	4,962931873	0,4774063	0,496466417
1396	ENSG00000100916	-0,222834025	4,015353599	2,5719279	0,20083652
1397	ENSG00000100934	-0,21280094	6,988498942	2,0287089	0,167695399
1398	ENSG00000100938	0,086718108	4,948756652	0,715732	0,435973549
1399	ENSG00000100941	-0,04276906	5,763150828	0,1731837	0,681123305
1400	ENSG00000100968	-0,297314351	4,118644849	1,8223732	0,19010439
1401	ENSG00000100979	-1,049723388	6,196956185	14,061094	0,001038255
1402	ENSG00000100983	-0,004655517	4,420008827	0,0018518	0,966043732
1403	ENSG00000100991	0,062717963	5,714312308	0,2594907	0,615282874
1404	ENSG00000100994	0,326925551	7,259352912	1,9348316	0,177477903
1405	ENSG00000100997	0,019941447	4,297666508	0,0258016	0,903501263
1406	ENSG00000101000	0,718424211	4,576358713	1,9948056	0,171163136
1407	ENSG00000101019	-0,252105077	5,88021505	2,7810052	0,108882361
1408	ENSG00000101040	0,229032481	5,248011913	1,8355454	0,188569725
1409	ENSG00000101052	0,485729197	3,901112658	5,439613	0,02875278
1410	ENSG00000101079	-0,068069947	5,308105119	0,253578	0,619316223
1411	ENSG00000101096	0,340555704	3,945418988	4,1644023	0,054368542
1412	ENSG00000101109	0,229465514	4,98125265	1,9800725	0,172688728
1413	ENSG00000101126	0,294001479	6,297713479	7,1608648	0,01343726
1414	ENSG00000101132	-0,392764731	4,255056064	2,5078108	0,126871818

1415	ENSG00000101134	0,717404565	3,652386178	8,8229363	0,006827719
1416	ENSG00000101138	-0,114740434	4,451537471	1,0515116	0,454776693
1417	ENSG00000101146	-0,040288137	3,975455241	0,0926636	0,763532335
1418	ENSG00000101150	0,2321069	5,939841037	3,9174107	0,059778378
1419	ENSG00000101152	-0,061310937	5,56727277	0,1279543	0,723808478
1420	ENSG00000101158	-0,090897361	4,936547992	0,365006	0,551623833
1421	ENSG00000101160	-0,139999345	6,63837501	1,10298	0,304433215
1422	ENSG00000101161	0,296943897	5,595669653	3,1093645	0,091061123
1423	ENSG00000101166	-0,221014116	5,57464596	0,973251	0,334086353
1424	ENSG00000101182	-0,316139334	6,389622114	4,2433137	0,05083497
1425	ENSG00000101191	0,140724417	5,662405774	0,9439734	0,341302749
1426	ENSG00000101193	0,241900699	4,927736263	4,8964125	0,04883425
1427	ENSG00000101210	-0,038371682	8,141454856	0,0185309	0,892898646
1428	ENSG00000101236	0,376159969	4,602200191	1,5465712	0,226108768
1429	ENSG00000101246	-0,131153018	4,407672282	0,4901678	0,490840219
1430	ENSG00000101247	-0,444659383	4,918173846	6,8213111	0,01555796
1431	ENSG00000101265	-0,182151568	4,829702271	0,3848934	0,54106549
1432	ENSG00000101266	-0,061622703	6,578111967	0,3528606	0,558248122
1433	ENSG00000101290	0,04829406	7,107040435	0,0893975	0,767616448
1434	ENSG00000101294	-0,086200671	5,19498191	0,2274075	0,63793069
1435	ENSG00000101310	0,081669201	4,447173802	0,1717901	0,682347338
1436	ENSG00000101333	-0,416294284	6,590189417	2,0923388	0,161465523
1437	ENSG00000101335	-0,246536536	9,506921048	0,8254464	0,372969174
1438	ENSG00000101337	0,08334372	5,32575029	0,3078719	0,584319319
1439	ENSG00000101343	0,014860139	5,321120378	0,0064927	0,93647173
1440	ENSG00000101346	0,173132836	5,326714763	1,4606147	0,239028333
1441	ENSG00000101347	-0,703389602	6,784615916	5,8591924	0,023752982
1442	ENSG00000101350	0,467119298	5,110491979	7,3593418	0,012380952
1443	ENSG00000101361	-0,166411133	5,390610861	1,5255832	0,22914252
1444	ENSG00000101365	-0,40964001	6,3082258	6,0717888	0,021595471
1445	ENSG00000101367	-0,199690387	6,345040098	1,7118666	0,20360576
1446	ENSG00000101384	-0,053260242	5,972581861	0,069089	0,794996651
1447	ENSG00000101391	-0,239220405	4,321824031	3,6997039	0,096079312
1448	ENSG00000101400	-0,400314105	6,660958578	2,5811318	0,121721393
1449	ENSG00000101407	-0,130738535	4,428757395	1,5828721	0,325110839
1450	ENSG00000101413	-0,240550406	5,90362807	2,8517964	0,104687102
1451	ENSG00000101417	0,085169121	3,751657196	0,2448807	0,693296474
1452	ENSG00000101421	-0,177506652	5,783820484	1,4015912	0,248473603
1453	ENSG00000101439	-0,209631257	7,847429895	1,0326759	0,320051283
1454	ENSG00000101444	0,03229941	5,013271458	0,0588478	0,906391238
1455	ENSG00000101445	0,47616256	4,079957624	5,7471888	0,024985414
1456	ENSG00000101452	0,007085989	4,377702492	0,0017236	0,967241267
1457	ENSG00000101463	-0,021378677	4,519801042	0,0135122	0,90846498
1458	ENSG00000101474	0,268787592	5,557018595	1,8150058	0,190969449
1459	ENSG00000101493	0,165204363	4,175636817	1,037638	0,364802661
1460	ENSG00000101544	0,214350029	4,652807967	2,4066219	0,134366331
1461	ENSG00000101557	-0,003156974	5,913174748	0,0009714	0,975402529
1462	ENSG00000101558	-0,067099347	7,393170918	0,2523608	0,620153836
1463	ENSG00000101574	0,032135207	3,820982268	0,0534472	0,828292028
1464	ENSG00000101577	0,15381123	4,762521645	0,6132268	0,441526058
1465	ENSG00000101596	-0,013691735	7,04732372	0,0065741	0,936075823
1466	ENSG00000101605	0,149614791	9,53138989	0,1813808	0,674129079
1467	ENSG00000101608	0,056126123	9,784166721	0,0191804	0,891050017
1468	ENSG00000101639	-0,087643157	6,666181627	0,2096495	0,651317794
1469	ENSG00000101654	0,088042319	6,026365564	0,419511	0,523541217
1470	ENSG00000101665	0,960324272	4,84554959	13,802791	0,001130823
1471	ENSG00000101745	0,038208632	6,918447294	0,0560912	0,81487064
1472	ENSG00000101751	0,164901074	5,313666665	1,3621114	0,255046486
1473	ENSG00000101752	0,157801388	6,598273218	0,7472079	0,39624452

1474	ENSG00000101773	0,057094118	4,069895832	0,0637757	0,80285725
1475	ENSG00000101782	0,144664635	6,230314506	0,5749882	0,455946551
1476	ENSG00000101825	1,307715161	5,215093695	7,9459193	0,009710799
1477	ENSG00000101843	-0,017296395	4,395972205	0,0055311	0,94135479
1478	ENSG00000101844	-0,524106523	4,453004121	5,3577728	0,029859204
1479	ENSG00000101846	0,263355505	4,501156837	3,2510254	0,084405866
1480	ENSG00000101849	0,1534759	4,191020315	0,5172593	0,479222388
1481	ENSG00000101856	0,133290567	5,39330075	0,278939	0,602432053
1482	ENSG00000101868	0,017601713	4,782082508	0,0395551	0,844086131
1483	ENSG00000101871	0,39558097	5,030657457	2,6870859	0,114706722
1484	ENSG00000101882	0,02402423	3,823282291	0,0420907	0,873857875
1485	ENSG00000101892	0,58514635	4,212266983	1,4173477	0,245924811
1486	ENSG00000101901	0,158947818	5,317470302	1,2471597	0,275550542
1487	ENSG00000101928	-0,17017041	4,284006064	0,4661277	0,501560411
1488	ENSG00000101935	0,250923599	3,568875848	2,0054505	0,188621415
1489	ENSG00000101938	0,75394214	5,72985665	2,9401616	0,099781881
1490	ENSG00000101940	-0,019178421	5,694163309	0,0112972	0,916270878
1491	ENSG00000101955	-0,43211333	5,60629244	5,7287581	0,025194964
1492	ENSG00000101966	0,060913713	5,962029294	0,3367197	0,567324154
1493	ENSG00000101972	-0,026538067	6,955819089	0,0173268	0,896414914
1494	ENSG00000101974	0,009645358	4,966229707	0,0015402	0,96903206
1495	ENSG00000102007	-0,176621746	4,419803124	1,0790587	0,309653867
1496	ENSG00000102024	0,288255363	6,92591022	1,5239527	0,229426613
1497	ENSG00000102038	-0,353179426	6,357359075	5,9682356	0,022601099
1498	ENSG00000102054	-0,034533313	6,321128272	0,1304011	0,72128493
1499	ENSG00000102081	0,26351195	5,733156339	3,3299459	0,080957145
1500	ENSG00000102125	-0,263955142	4,529795016	2,2608737	0,14622158
1501	ENSG00000102144	-0,381876394	8,550579212	5,0122432	0,03504986
1502	ENSG00000102158	-0,111297836	6,628537268	0,4487858	0,509550326
1503	ENSG00000102172	0,188231644	5,459210245	1,5543842	0,224976809
1504	ENSG00000102178	-0,186286536	5,244530377	1,3229973	0,261817036
1505	ENSG00000102181	-0,029040427	6,019885267	0,0317926	0,860037129
1506	ENSG00000102189	0,158379209	6,537339659	1,0985672	0,305402788
1507	ENSG00000102218	0,186621808	3,705239364	0,7464273	0,396487481
1508	ENSG00000102225	-0,230265274	5,809775472	1,8822324	0,183251429
1509	ENSG00000102226	0,591412704	5,450332629	9,4165622	0,005416588
1510	ENSG00000102241	0,060649544	6,432527981	0,2374432	0,630632686
1511	ENSG00000102265	0,983027782	7,192421151	3,7304472	0,065781745
1512	ENSG00000102287	-0,004169363	4,784170505	0,0001377	0,99073848
1513	ENSG00000102309	-0,267530285	3,939385038	3,616141	0,147734948
1514	ENSG00000102316	0,223331449	6,773651136	1,9748979	0,173228474
1515	ENSG00000102317	-0,178749195	6,94078167	1,2727707	0,270822644
1516	ENSG00000102349	0,013119596	4,023689005	0,0039008	0,950736302
1517	ENSG00000102359	-0,819303774	4,826289515	4,5299819	0,04418393
1518	ENSG00000102393	-0,437101991	3,639743537	7,6066501	0,026579292
1519	ENSG00000102401	0,287743835	6,326341337	4,7987706	0,038786963
1520	ENSG00000102409	0,070288628	5,536664117	0,1999546	0,658919922
1521	ENSG00000102452	-0,316233671	4,690254533	1,7757374	0,195662955
1522	ENSG00000102471	-0,006828376	5,287889883	0,0014761	0,969683009
1523	ENSG00000102531	0,239387164	6,685838032	1,4227394	0,245054675
1524	ENSG00000102547	-0,232143774	5,194064336	1,5216816	0,22976316
1525	ENSG00000102572	0,033483064	4,861268592	0,0335418	0,856282033
1526	ENSG00000102580	-0,103931839	6,741780991	0,3939255	0,536395415
1527	ENSG00000102595	-0,169997744	5,848500185	1,0128045	0,324652331
1528	ENSG00000102606	-0,119847651	5,888773937	0,6858405	0,416037035
1529	ENSG00000102678	0,615088686	3,661142041	7,7986249	0,010315664
1530	ENSG00000102683	-0,166691498	6,437850553	0,2932639	0,593318888
1531	ENSG00000102699	0,173033895	5,942714186	2,1641165	0,15470389
1532	ENSG00000102710	0,045250927	5,323921138	0,1651524	0,688181804

1533	ENSG00000102738	-0,398114219	4,625309711	5,1163152	0,033411037
1534	ENSG00000102753	-0,220943604	6,301623812	1,5908274	0,219790284
1535	ENSG00000102755	-0,397181767	7,113339198	2,7694303	0,109581159
1536	ENSG00000102760	-0,017736791	4,534344872	0,0059603	0,939126405
1537	ENSG00000102763	0,02281289	6,535462646	0,0171026	0,897083496
1538	ENSG00000102780	0,186556835	5,596812726	0,7801924	0,386177693
1539	ENSG00000102781	-0,055553192	6,256984014	0,132711	0,718949279
1540	ENSG00000102786	0,067552714	5,991278173	0,1138935	0,738800031
1541	ENSG00000102802	0,028271477	5,146712351	0,0032554	0,954990766
1542	ENSG00000102804	-0,215744444	7,337298101	2,8055694	0,107369635
1543	ENSG00000102805	-0,116015279	4,707153066	0,6187389	0,439507532
1544	ENSG00000102870	-0,042442164	4,91796932	0,0394663	0,844264847
1545	ENSG00000102882	0,139999967	4,732649814	0,6365359	0,433089045
1546	ENSG00000102893	0,044756087	6,737614987	0,1423028	0,709431972
1547	ENSG00000102897	-0,315237642	4,746816081	2,488463	0,128273511
1548	ENSG00000102898	-0,038555109	5,058821795	0,1017717	0,752569075
1549	ENSG00000102900	-0,291203737	4,529807798	6,3094626	0,024797112
1550	ENSG00000102908	0,097943447	6,602807225	0,3154527	0,579759333
1551	ENSG00000102910	0,018418927	6,870069493	0,028425	0,867575237
1552	ENSG00000102921	0,079341006	5,000928949	0,536649	0,575054308
1553	ENSG00000102967	0,240912577	4,175172215	2,389271	0,168965163
1554	ENSG00000102974	0,151492192	5,017313029	1,5795996	0,246383025
1555	ENSG00000102978	0,100366225	5,303980382	0,9245868	0,373324268
1556	ENSG00000102996	-0,438676286	5,121018345	1,8622185	0,185508462
1557	ENSG00000103018	-0,21616707	5,447829045	2,7047643	0,113536005
1558	ENSG00000103034	-0,231405665	7,239786322	0,576327	0,455428878
1559	ENSG00000103035	-0,524174014	5,602075453	9,9817037	0,004366129
1560	ENSG00000103042	-0,003019768	4,04409901	0,0002518	0,987476143
1561	ENSG00000103047	0,117446415	4,002233208	0,7123069	0,548402916
1562	ENSG00000103051	-0,110241054	5,378297399	0,7369842	0,399413789
1563	ENSG00000103064	-0,260921271	4,480614689	1,0938273	0,306439905
1564	ENSG00000103066	0,431733347	4,17484958	3,6893204	0,067182067
1565	ENSG00000103091	0,000285992	5,013651964	9,668E-07	0,999223926
1566	ENSG00000103111	0,030219009	4,129295321	0,0326366	0,933269792
1567	ENSG00000103121	-0,710109385	5,288824342	12,097419	0,002023
1568	ENSG00000103150	0,139134307	6,469202638	0,239074	0,62948342
1569	ENSG00000103160	-0,587998714	4,981343026	15,512351	0,000650414
1570	ENSG00000103194	-0,180113297	5,400722783	2,471587	0,129463945
1571	ENSG00000103196	-2,01932224	6,492132344	40,378824	1,70793E-06
1572	ENSG00000103197	0,316117253	5,004934989	2,1052548	0,160231686
1573	ENSG00000103222	0,263145229	4,626560272	1,2503246	0,274978359
1574	ENSG00000103226	0,191092597	7,646713892	0,6183288	0,439657219
1575	ENSG00000103264	0,109831283	4,94073609	0,3794055	0,543940526
1576	ENSG00000103275	-0,053614295	4,798239977	0,1388891	0,712775505
1577	ENSG00000103316	0,744433625	5,654538145	3,4501697	0,076032683
1578	ENSG00000103319	-0,070316061	5,553661949	0,0882986	0,769005647
1579	ENSG00000103335	-0,00329091	4,394545542	0,0001275	0,991087828
1580	ENSG00000103342	-0,098791089	6,770903499	1,1454947	0,295506095
1581	ENSG00000103351	0,293904517	4,313228182	5,2053997	0,045583556
1582	ENSG00000103353	-0,049364262	5,911818739	0,1049485	0,748883536
1583	ENSG00000103356	0,009357452	4,741641321	0,0030561	0,956388959
1584	ENSG00000103363	-0,112889424	5,811074733	0,1839802	0,671946221
1585	ENSG00000103365	-0,004923422	5,723492758	0,0010165	0,974839237
1586	ENSG00000103381	0,285100102	4,259898926	3,954612	0,060431985
1587	ENSG00000103404	-0,151991691	5,609943105	1,0095187	0,325421875
1588	ENSG00000103415	-0,612441935	5,218603223	15,401116	0,000673677
1589	ENSG00000103423	-0,155487576	5,197884955	0,8812975	0,357554235
1590	ENSG00000103429	0,036624664	5,006046797	0,1087656	0,74451393
1591	ENSG00000103479	0,163087667	6,224016105	2,3306244	0,140373528

1592	ENSG00000103485	-0,308563531	4,245098766	2,5704725	0,122454629
1593	ENSG00000103489	-0,27507128	4,357890094	1,4117862	0,246826498
1594	ENSG00000103496	-0,151771171	4,512941735	0,6582327	0,425461083
1595	ENSG00000103502	-0,023842901	5,378824257	0,0214907	0,884721368
1596	ENSG00000103507	-0,270309082	4,663897698	2,6768591	0,115362704
1597	ENSG00000103510	-0,050678183	4,978404514	0,1237381	0,728197391
1598	ENSG00000103512	0,104439966	7,655967888	0,2288442	0,636876161
1599	ENSG00000103540	0,193638776	4,66975849	1,5148046	0,230786034
1600	ENSG00000103544	0,132477885	5,45771702	1,6494579	0,265043701
1601	ENSG00000103549	0,279772244	4,254485987	1,7766786	0,195548811
1602	ENSG00000103569	0,685843732	4,338326985	2,0157541	0,16902202
1603	ENSG00000103591	-0,043534681	4,881353335	0,11255	0,80286785
1604	ENSG00000103642	-0,320658868	4,400807698	4,406718	0,046916826
1605	ENSG00000103657	0,01769466	8,036064475	0,01429	0,905876416
1606	ENSG00000103671	-0,077006016	4,224806899	0,2031121	0,656419953
1607	ENSG00000103707	-0,108752691	3,923918635	0,7437012	0,520142929
1608	ENSG00000103710	-0,709647688	5,161540132	9,8550136	0,004580511
1609	ENSG00000103769	-0,35205626	6,702178563	6,9477185	0,014713093
1610	ENSG00000103811	-0,206898554	5,373525782	1,7690583	0,196475383
1611	ENSG00000103852	-0,181104889	4,491704491	2,4667359	0,18300635
1612	ENSG00000103855	-0,191118961	4,685116511	0,7374172	0,399307999
1613	ENSG00000103876	0,270210002	4,269560005	0,6734319	0,420241246
1614	ENSG00000103942	-0,102586487	4,468853448	0,3201586	0,576965026
1615	ENSG00000103966	0,044260331	5,279835813	0,0606437	0,807655296
1616	ENSG00000103978	0,060040829	5,621042434	0,1638127	0,689391622
1617	ENSG00000103994	-0,00900814	10,2957247	0,0007653	0,978167558
1618	ENSG00000103995	-0,191779865	3,535737214	1,1148808	0,352621942
1619	ENSG00000104043	-0,294237006	4,404186471	1,0526717	0,315511319
1620	ENSG00000104047	-0,035854846	5,249191537	0,0777454	0,782847349
1621	ENSG00000104064	-0,010328812	3,971146838	0,0044712	0,978662092
1622	ENSG00000104067	0,221519409	7,718667243	3,555031	0,071952779
1623	ENSG00000104093	-0,039059878	5,919350693	0,0587176	0,810671731
1624	ENSG00000104131	-0,129741399	5,696700137	0,7984182	0,380759085
1625	ENSG00000104133	0,059177482	6,787282961	0,3579087	0,555467642
1626	ENSG00000104142	0,145535059	4,094173766	0,5703473	0,45774835
1627	ENSG00000104154	0,239331194	4,054601642	2,2556106	0,146670802
1628	ENSG00000104164	-0,099962837	5,813493989	0,3789989	0,544154707
1629	ENSG00000104177	0,131754526	4,277669905	0,550659	0,46552328
1630	ENSG00000104205	0,09502581	4,65187147	0,7060313	0,483419202
1631	ENSG00000104213	-0,668109632	3,812011298	9,8732223	0,004549
1632	ENSG00000104218	0,052462092	4,884344029	0,1457858	0,706074292
1633	ENSG00000104219	-0,096701863	5,944271909	0,2515157	0,620753443
1634	ENSG00000104231	-0,119892099	5,08661569	0,4198824	0,523381245
1635	ENSG00000104290	-0,01047656	3,924648432	0,0062136	0,976878368
1636	ENSG00000104299	-0,173746427	3,801546815	1,6606459	0,311125705
1637	ENSG00000104320	0,122683588	5,692137804	0,8054994	0,378678492
1638	ENSG00000104324	0,081708189	5,667002687	0,4075893	0,529449643
1639	ENSG00000104325	-0,530161478	7,254404239	7,8406343	0,010139021
1640	ENSG00000104331	-0,026251514	6,998588509	0,0456831	0,832620601
1641	ENSG00000104332	-0,408408318	8,518212935	2,1282702	0,158061135
1642	ENSG00000104341	-0,015284649	8,04815505	0,0043826	0,947787176
1643	ENSG00000104343	-0,061474049	5,821539404	0,058262	0,81139287
1644	ENSG00000104361	0,276783907	4,544067567	2,5840826	0,121519328
1645	ENSG00000104365	-0,044300194	4,983114855	0,0622341	0,805202793
1646	ENSG00000104368	-0,339498116	4,679004438	1,7185888	0,202751209
1647	ENSG00000104369	-0,241043291	3,630211721	1,1910508	0,286364249
1648	ENSG00000104375	-0,04066192	5,006436633	0,0799716	0,779850259
1649	ENSG00000104388	0,003560636	6,848261737	0,0004567	0,98313295
1650	ENSG00000104408	0,009690253	7,883591629	0,0011402	0,973352953

1651	ENSG00000104412	-0,331605295	5,924246499	2,1820388	0,153126638
1652	ENSG00000104419	-0,123674973	7,004477567	0,358953	0,554915957
1653	ENSG00000104427	-0,073053898	3,975564164	0,1441499	0,707656033
1654	ENSG00000104442	-0,182512175	5,227179374	1,4444901	0,241584273
1655	ENSG00000104447	0,295745414	5,811916114	1,5473008	0,22600275
1656	ENSG00000104472	-0,171100594	3,690259326	1,4093473	0,366493485
1657	ENSG00000104490	-0,594375346	4,578651839	8,5585098	0,007582749
1658	ENSG00000104497	-0,089994125	4,263372749	0,1330607	0,71859589
1659	ENSG00000104517	0,017109518	7,323886107	0,0304436	0,863001472
1660	ENSG00000104529	0,018173776	5,74645693	0,0088713	0,92577194
1661	ENSG00000104613	-0,041566826	5,500818429	0,097471	0,75767538
1662	ENSG00000104626	0,015989339	3,843177709	0,0079076	0,929907679
1663	ENSG00000104635	-0,629162082	7,044913148	8,5323012	0,007662453
1664	ENSG00000104643	-0,347289493	5,433429479	5,9701133	0,0225821
1665	ENSG00000104660	0,102811936	5,692073385	0,4652798	0,501945953
1666	ENSG00000104671	-0,418251353	4,920918017	2,3174035	0,141498722
1667	ENSG00000104679	-0,102568818	4,225475677	0,4214835	0,522597434
1668	ENSG00000104687	-0,001407153	5,596208482	0,0001007	0,992080844
1669	ENSG00000104695	0,026815666	6,18147547	0,0759874	0,785251662
1670	ENSG00000104723	-0,290551146	5,259275648	2,9349221	0,100067017
1671	ENSG00000104738	-0,2875748	4,567884235	2,9021733	0,101870837
1672	ENSG00000104756	-0,462885349	6,090293783	7,6152355	0,011127601
1673	ENSG00000104763	-0,145654097	9,59801761	0,2950656	0,592193278
1674	ENSG00000104765	0,43154688	7,084661842	2,916048	0,101102032
1675	ENSG00000104774	0,127129892	4,065713958	0,2933998	0,593233786
1676	ENSG00000104805	-0,030729389	6,855551172	0,0220728	0,88318216
1677	ENSG00000104812	-0,110260036	6,092838608	0,2170307	0,645672145
1678	ENSG00000104823	-0,206212097	6,279354155	0,6201048	0,439009607
1679	ENSG00000104824	0,062801275	5,934757463	0,18295	0,672803561
1680	ENSG00000104852	0,19819186	6,618428339	1,3717809	0,253439595
1681	ENSG00000104853	-0,206577991	5,369473666	1,5553281	0,224840532
1682	ENSG00000104870	0,170700439	5,810861984	0,5572718	0,462887885
1683	ENSG00000104872	-0,006311416	4,226492463	0,0012926	0,971628533
1684	ENSG00000104879	-0,587985362	10,61725694	4,3387252	0,048505624
1685	ENSG00000104881	-0,203216379	5,068279638	1,4074224	0,247536991
1686	ENSG00000104904	-0,201179628	7,731989998	1,1274248	0,299294647
1687	ENSG00000104936	-0,154324708	6,472364497	0,4867082	0,492358443
1688	ENSG00000104964	-0,276007277	7,365889526	2,2844575	0,144228716
1689	ENSG00000104969	-0,090033369	5,369765418	0,1151966	0,737368352
1690	ENSG00000104979	-0,189449252	4,945347263	1,4419084	0,241992887
1691	ENSG00000104980	-0,335705778	4,610653233	4,540864	0,043951414
1692	ENSG00000105053	0,037479808	3,893336455	0,0533504	0,904834439
1693	ENSG00000105058	-0,074171395	4,937436452	0,2357087	0,631893358
1694	ENSG00000105127	-0,117464695	4,338979865	0,6446849	0,430182426
1695	ENSG00000105135	-0,161818671	5,699873883	0,6910263	0,414321242
1696	ENSG00000105136	0,03653698	3,671627256	0,0841292	0,878439725
1697	ENSG00000105171	-0,257078244	4,369724872	2,9307818	0,103345091
1698	ENSG00000105176	-0,055294239	5,851044577	0,2364082	0,631374794
1699	ENSG00000105185	-0,540516281	5,490372318	8,2242918	0,008671635
1700	ENSG00000105186	-0,154060887	4,847884741	2,1598446	0,194506609
1701	ENSG00000105193	0,120846352	7,205094036	0,2918408	0,594211153
1702	ENSG00000105197	-0,225650763	4,852203517	1,5597818	0,224198974
1703	ENSG00000105202	0,155468197	4,719878213	0,4974317	0,48767848
1704	ENSG00000105220	-0,136876942	7,717127282	0,5542318	0,464096362
1705	ENSG00000105221	0,128303797	5,868725235	0,4699929	0,4998095
1706	ENSG00000105223	0,106080796	6,150555754	0,2961136	0,591540615
1707	ENSG00000105254	-0,075117497	4,775731367	0,4008521	0,53854509
1708	ENSG00000105281	-0,122673937	3,785003204	0,1236619	0,728282077
1709	ENSG00000105323	0,152900833	6,041562595	0,5342913	0,472153925

1710	ENSG00000105339	-0,30091488	4,923608128	1,6933611	0,205981466
1711	ENSG00000105341	-0,300807224	5,068148338	3,2795266	0,083167669
1712	ENSG00000105357	0,154097762	5,127811922	0,5014629	0,485938827
1713	ENSG00000105372	0,268629526	7,335263222	1,2424478	0,276456554
1714	ENSG00000105373	0,301652566	6,395472074	2,2540139	0,146807407
1715	ENSG00000105379	-0,211778834	5,093313846	0,639742	0,431948472
1716	ENSG00000105397	0,04915962	4,641700337	0,1091349	0,870634181
1717	ENSG00000105401	-0,213058303	6,451007214	1,8254586	0,189743545
1718	ENSG00000105402	-0,11898338	4,73639671	0,4136146	0,526470294
1719	ENSG00000105426	0,457172191	5,658563582	0,9856807	0,331081197
1720	ENSG00000105429	0,19096806	5,149032217	0,6632462	0,423728222
1721	ENSG00000105438	0,126126224	5,137452592	0,5434907	0,46840809
1722	ENSG00000105443	0,261013937	4,241152243	3,2136634	0,11265791
1723	ENSG00000105483	-0,008420457	5,392981603	0,0036534	0,952320956
1724	ENSG00000105497	0,140150406	4,03571694	1,5027152	0,395788017
1725	ENSG00000105499	0,298722211	4,598216458	2,7575995	0,110300828
1726	ENSG00000105552	0,032281038	4,208300047	0,0259063	0,873527954
1727	ENSG00000105568	0,132111438	7,374784039	0,4145411	0,52601157
1728	ENSG00000105576	0,354583808	5,289595968	2,7915821	0,108248366
1729	ENSG00000105609	-1,008349361	3,899573669	15,131783	0,000733858
1730	ENSG00000105640	0,258095294	7,283178889	1,352042	0,25678685
1731	ENSG00000105677	-0,402804143	4,865738755	5,0705762	0,034135833
1732	ENSG00000105698	0,119300534	5,964837708	0,7072513	0,408949921
1733	ENSG00000105700	-0,069409406	4,10292226	0,2207077	0,655623052
1734	ENSG00000105701	0,120709799	6,193120567	0,3296684	0,571400578
1735	ENSG00000105708	0,13114289	3,827323276	0,5437472	0,468304387
1736	ENSG00000105711	0,120218004	5,315539313	0,7787598	0,38657556
1737	ENSG00000105778	-0,023877131	5,430738387	0,0344	0,854470997
1738	ENSG00000105792	0,320147626	3,930101229	1,1681774	0,29092514
1739	ENSG00000105793	-0,089277509	4,711205421	0,3986538	0,580388565
1740	ENSG00000105808	0,414903291	4,00798113	1,9422048	0,176686449
1741	ENSG00000105810	0,346520623	5,818324194	1,8936421	0,181979768
1742	ENSG00000105819	-0,099197071	5,96338526	0,8977888	0,353143533
1743	ENSG00000105821	-0,162547056	4,497856622	1,8698725	0,263071293
1744	ENSG00000105829	-0,05080315	4,039137867	0,0686184	0,795679606
1745	ENSG00000105835	-0,485792237	8,259782852	1,646784	0,212115599
1746	ENSG00000105849	-0,190403384	4,619564346	0,7653352	0,39066447
1747	ENSG00000105851	0,310248072	3,591659527	1,9101386	0,180160197
1748	ENSG00000105854	-0,321197318	5,543877661	5,4837842	0,028147432
1749	ENSG00000105855	1,469166312	5,546255578	3,7464291	0,065246583
1750	ENSG00000105856	0,085171085	6,290960019	0,2588789	0,615714202
1751	ENSG00000105866	-0,046036804	4,655646023	0,1438325	0,764855812
1752	ENSG00000105877	0,038844308	3,641804654	0,0274201	0,869919437
1753	ENSG00000105879	-0,094085359	4,514540709	0,8969483	0,535741013
1754	ENSG00000105887	0,082479382	6,986666257	0,3490742	0,560351546
1755	ENSG00000105894	0,44830427	5,796126838	4,5537918	0,043676976
1756	ENSG00000105926	0,51712182	4,794158328	1,8119998	0,191323801
1757	ENSG00000105939	0,487658598	5,183963295	5,3188858	0,030401541
1758	ENSG00000105953	-0,220183595	7,805842338	0,7889067	0,383581377
1759	ENSG00000105968	-0,144113758	7,091855791	1,8287512	0,189314172
1760	ENSG00000105971	-0,145236344	7,224214309	0,5207423	0,477762533
1761	ENSG00000105974	-0,410102688	8,629712407	2,1865925	0,152717296
1762	ENSG00000105976	0,650207112	5,652948455	2,2703138	0,145419957
1763	ENSG00000105983	-0,261827518	5,716067804	5,6466061	0,026121075
1764	ENSG00000105993	0,024787853	7,087165246	0,0293877	0,865374244
1765	ENSG00000106012	0,105601859	3,775708453	0,3065474	0,619466608
1766	ENSG00000106025	-0,323855499	5,549990717	1,3459735	0,257827411
1767	ENSG00000106028	-0,51303341	5,789138654	8,3340641	0,008296085
1768	ENSG00000106034	0,579796125	6,265744584	6,6446439	0,016790447

1769	ENSG00000106049	-0,211403153	6,080951158	3,3327389	0,080817866
1770	ENSG00000106052	-0,336930554	7,601923768	2,5853721	0,12143115
1771	ENSG00000106066	0,101936764	4,544196641	0,099255	0,755552282
1772	ENSG00000106070	-0,000301402	5,787956346	3,017E-06	0,998629048
1773	ENSG00000106078	-0,328517549	5,524696029	3,0221009	0,095443197
1774	ENSG00000106080	-0,002837597	4,457295011	0,000304	0,986237667
1775	ENSG00000106086	0,162067669	4,12220042	0,9519199	0,33933291
1776	ENSG00000106100	-0,170976297	4,294495456	0,8950757	0,353892243
1777	ENSG00000106105	-0,212115086	5,55433888	4,5239919	0,044273438
1778	ENSG00000106144	0,026978593	3,825673237	0,0243137	0,940914359
1779	ENSG00000106153	-0,244181161	7,033169615	3,3899537	0,078421848
1780	ENSG00000106211	-0,39977458	8,846842053	0,5602213	0,461720313
1781	ENSG00000106244	-0,0725865	5,520995746	0,3166768	0,579011026
1782	ENSG00000106245	0,075081024	3,844903983	0,3872624	0,686432165
1783	ENSG00000106258	-0,094757105	3,847135379	0,2163387	0,646196396
1784	ENSG00000106261	0,243371798	6,331275427	3,5377182	0,07260173
1785	ENSG00000106263	-0,043064975	6,19631602	0,0960514	0,759388028
1786	ENSG00000106299	0,070261004	5,779106067	0,3552459	0,556930915
1787	ENSG00000106344	-0,289761688	5,389905929	3,4124686	0,077546485
1788	ENSG00000106355	-0,3776331	4,741894422	2,9489163	0,099307548
1789	ENSG00000106366	1,354201247	3,984809571	12,889578	0,001537959
1790	ENSG00000106392	0,203285847	5,101872935	1,4918951	0,23423551
1791	ENSG00000106397	-0,069223166	4,378922494	0,1098838	0,74326343
1792	ENSG00000106399	-0,128362559	3,757018892	0,3741108	0,546741836
1793	ENSG00000106400	-0,123487182	4,583317174	0,5353992	0,471700185
1794	ENSG00000106443	-0,007330123	5,586113803	0,0027511	0,958619103
1795	ENSG00000106459	-0,112395161	4,21587376	0,9356012	0,411000263
1796	ENSG00000106460	-0,302004563	7,02376323	1,2983103	0,266193728
1797	ENSG00000106479	0,309817314	3,647420175	2,7560234	0,131476392
1798	ENSG00000106484	0,718796077	4,779090213	1,4945505	0,233832349
1799	ENSG00000106511	0,359215433	4,153437648	3,0913743	0,091945136
1800	ENSG00000106524	-0,270466937	5,50143146	1,1621243	0,2921483
1801	ENSG00000106537	-0,405311811	4,23068171	5,1339128	0,033136785
1802	ENSG00000106538	0,62277112	4,790095017	2,8552127	0,104523914
1803	ENSG00000106546	0,131564045	5,487369186	0,2071466	0,653259628
1804	ENSG00000106554	-0,646256731	6,584924636	16,29875	0,000508944
1805	ENSG00000106565	-0,432914661	6,32986216	1,1184484	0,301181478
1806	ENSG00000106591	-0,395330278	5,197372123	5,0703547	0,034139385
1807	ENSG00000106603	-0,300784284	5,461520874	9,9356625	0,004430286
1808	ENSG00000106605	-0,262085116	4,6736833	3,6680537	0,067875605
1809	ENSG00000106608	0,212850464	5,072270027	2,289187	0,143800598
1810	ENSG00000106609	0,274863724	6,110352508	4,3562865	0,048049435
1811	ENSG00000106610	-0,303023896	4,417118334	3,0322636	0,094920491
1812	ENSG00000106615	-0,260959622	5,921502531	3,6816475	0,067403243
1813	ENSG00000106617	-0,089527195	7,449130901	0,1043062	0,749625605
1814	ENSG00000106624	0,638950422	6,827202234	5,8638986	0,0237027
1815	ENSG00000106628	-0,04214835	5,488302389	0,0440812	0,835542401
1816	ENSG00000106631	-0,539734464	12,53464469	2,0044411	0,170174235
1817	ENSG00000106635	-0,188496855	4,955236921	1,4729671	0,237135097
1818	ENSG00000106636	-0,065068918	5,363342953	0,3413649	0,564682547
1819	ENSG00000106638	0,330973814	3,85142182	4,3370311	0,076652825
1820	ENSG00000106682	0,047799839	7,358165029	0,2340207	0,633094384
1821	ENSG00000106692	0,018550688	5,000610839	0,0220034	0,883360232
1822	ENSG00000106701	0,017471865	4,148944356	0,00521	0,943079402
1823	ENSG00000106714	-0,95381867	5,357954068	5,0868544	0,033875883
1824	ENSG00000106723	0,152941643	6,325844832	2,6409601	0,117653353
1825	ENSG00000106733	0,32506116	3,905307179	2,5522422	0,123720756
1826	ENSG00000106771	-0,120277068	7,383575831	0,7078965	0,408734175
1827	ENSG00000106772	-0,305143456	5,665870643	0,8278622	0,372282728

1828	ENSG00000106780	-0,227328115	5,257281816	1,2301672	0,278782131
1829	ENSG00000106799	0,228940385	6,005217117	1,1582319	0,292938478
1830	ENSG00000106803	0,033535835	4,48384573	0,0223448	0,882470137
1831	ENSG00000106809	1,414912282	6,65149471	6,0421969	0,02188228
1832	ENSG00000106819	1,564429128	5,969827374	13,912959	0,001090283
1833	ENSG00000106823	1,318201457	5,640023479	11,630119	0,002386159
1834	ENSG00000106829	0,367394034	4,319972165	5,9897614	0,022398252
1835	ENSG00000106853	-0,26411926	4,571335537	3,1009383	0,091455178
1836	ENSG00000106868	0,047734492	3,924588977	0,0647791	0,801346634
1837	ENSG00000106948	0,263454151	3,800514995	0,8907838	0,355027189
1838	ENSG00000106991	0,568431178	7,051773343	3,3296601	0,080994567
1839	ENSG00000106992	-0,849245747	4,372266854	7,6231199	0,011091277
1840	ENSG00000106993	-0,111691077	5,649734057	0,2827081	0,600005435
1841	ENSG00000107020	-0,69636824	4,460159856	9,2058045	0,005877052
1842	ENSG00000107021	0,043250028	4,081964461	0,0572788	0,890842341
1843	ENSG00000107036	0,043452677	5,371640829	0,0771308	0,783693088
1844	ENSG00000107077	0,087284167	5,320749091	0,5497288	0,465870105
1845	ENSG00000107099	0,105205666	4,475935935	0,1482985	0,703687788
1846	ENSG00000107104	0,319174767	7,425086655	3,1263235	0,090236772
1847	ENSG00000107164	0,268150556	5,142042003	3,4811241	0,074796899
1848	ENSG00000107165	-0,284641507	5,281475712	0,7351631	0,400018335
1849	ENSG00000107175	0,039351033	4,338359145	0,0658554	0,817668721
1850	ENSG00000107185	0,131317918	4,706323287	0,4880761	0,491757202
1851	ENSG00000107186	0,173235668	7,364268363	1,7361668	0,200492965
1852	ENSG00000107201	0,123686271	4,712114158	0,5207704	0,47775078
1853	ENSG00000107223	-0,190869058	6,514609088	0,7243106	0,403465067
1854	ENSG00000107242	-0,685381562	5,596404248	7,6175381	0,011116979
1855	ENSG00000107249	-0,24615521	3,715622699	1,5853072	0,220566152
1856	ENSG00000107262	-0,19027014	6,3393804	3,0278388	0,095101249
1857	ENSG00000107263	-0,221836993	5,881533905	0,9838427	0,331523203
1858	ENSG00000107290	0,051147961	7,599398727	0,1978585	0,660578254
1859	ENSG00000107317	-0,26827606	5,5838016	0,5165443	0,479523022
1860	ENSG00000107331	0,243823744	4,509648648	1,2993942	0,265999564
1861	ENSG00000107341	-0,072816632	5,58860937	0,3822239	0,542439448
1862	ENSG00000107362	-0,117035515	3,660502189	0,9165432	0,536674312
1863	ENSG00000107371	-0,022543401	3,680406292	0,0277739	0,902090598
1864	ENSG00000107372	-0,109964674	7,420761948	0,5304984	0,473692999
1865	ENSG00000107438	-0,239302691	6,672937051	1,9033772	0,180903279
1866	ENSG00000107443	0,070942321	3,45185337	0,3297631	0,715286706
1867	ENSG00000107521	0,131815144	4,388154871	0,5767822	0,455253098
1868	ENSG00000107537	-0,088459294	5,865200855	0,3094027	0,583392577
1869	ENSG00000107551	-0,116542495	5,749199996	0,3663096	0,550919726
1870	ENSG00000107554	-0,03085962	4,284472723	0,0230126	0,880740577
1871	ENSG00000107560	0,040582128	5,191241915	0,0819349	0,777237306
1872	ENSG00000107562	0,294066673	7,120598827	3,1270648	0,090200937
1873	ENSG00000107566	0,287880168	4,248541694	2,381811	0,136337391
1874	ENSG00000107581	0,120372476	7,639716545	1,5150017	0,230713861
1875	ENSG00000107614	-0,196146393	3,863363259	1,8436961	0,269907286
1876	ENSG00000107625	-0,06622333	5,437628901	0,1349189	0,716727348
1877	ENSG00000107643	-0,028726508	5,435218696	0,0338702	0,855588278
1878	ENSG00000107651	0,050338985	5,284310694	0,2328239	0,633960516
1879	ENSG00000107669	-0,230981623	6,349120484	2,2600544	0,1462914
1880	ENSG00000107672	-0,0918789	4,62443957	0,6731097	0,507904466
1881	ENSG00000107679	-0,133725216	6,012060416	0,5271682	0,475088718
1882	ENSG00000107731	0,603832458	4,552608227	5,7285901	0,025196882
1883	ENSG00000107738	-0,061586808	5,107716094	0,0574813	0,812635534
1884	ENSG00000107742	0,861003437	4,134123009	7,0291669	0,014234519
1885	ENSG00000107745	-0,061342882	5,548036042	0,397307	0,534644833
1886	ENSG00000107758	-0,045129073	6,293510851	0,1735146	0,680836635

1887	ENSG00000107771	-0,025248654	7,358786873	0,0250367	0,87564922
1888	ENSG00000107779	0,011132992	6,451341929	0,0075089	0,931690173
1889	ENSG00000107796	-1,119685148	8,079589788	11,640289	0,002377534
1890	ENSG00000107798	0,360420104	5,791491484	1,3562288	0,256072103
1891	ENSG00000107816	0,267425742	4,762752239	2,7754072	0,109219673
1892	ENSG00000107819	0,317356324	4,622672774	3,1470872	0,089239197
1893	ENSG00000107829	-0,047918997	5,597972954	0,0617095	0,806007999
1894	ENSG00000107854	0,207588852	6,017931092	1,7652101	0,196945345
1895	ENSG00000107862	0,179423605	5,917309589	0,7789717	0,386543438
1896	ENSG00000107863	0,24919409	6,974763619	2,8643465	0,103968721
1897	ENSG00000107864	0,127144297	4,779438366	0,4366213	0,515290446
1898	ENSG00000107874	-0,065695166	4,843360774	0,1261394	0,7256895
1899	ENSG00000107890	-0,259859123	5,582450295	2,2527835	0,146912787
1900	ENSG00000107897	0,088482119	5,662951792	0,3133765	0,581000945
1901	ENSG00000107929	0,036104378	5,818344395	0,0456334	0,832716857
1902	ENSG00000107937	-0,233412283	5,169087914	2,9464267	0,099399879
1903	ENSG00000107938	-0,100078897	4,57295631	0,7508235	0,470191378
1904	ENSG00000107949	-0,247396802	5,194917689	2,5932408	0,120894698
1905	ENSG00000107951	-0,135833659	4,79198523	1,7279973	0,250234124
1906	ENSG00000107957	-0,039606728	6,013683876	0,0276547	0,869369407
1907	ENSG00000107959	0,08874805	5,913635904	0,4762695	0,496965063
1908	ENSG00000107960	-0,014901912	4,603359207	0,0249954	0,937992985
1909	ENSG00000107968	0,181407958	4,670158827	0,2588345	0,615744358
1910	ENSG00000108001	0,308792244	3,98362982	3,2059198	0,086481078
1911	ENSG00000108010	-0,350377723	5,380605536	6,5608942	0,017385783
1912	ENSG00000108021	-0,049290728	6,466234823	0,1519439	0,700241027
1913	ENSG00000108039	-0,152933803	5,430871524	1,6533647	0,211191235
1914	ENSG00000108055	0,032936629	5,807185701	0,0778569	0,782695756
1915	ENSG00000108061	-0,154005912	6,251465224	0,8707742	0,360387693
1916	ENSG00000108064	-0,093177936	5,263813847	0,279773	0,601893288
1917	ENSG00000108091	-0,010553534	5,575302348	0,0043472	0,947997832
1918	ENSG00000108094	-0,08601808	5,285544435	0,5657535	0,494660012
1919	ENSG00000108100	0,24841383	5,978915605	7,0625968	0,014009584
1920	ENSG00000108107	0,16511185	6,410117727	0,5562288	0,463301939
1921	ENSG00000108175	0,127406782	5,709373751	0,212258	0,649308998
1922	ENSG00000108179	-0,438902771	6,203506215	4,445224	0,046043149
1923	ENSG00000108219	-0,389046211	5,110204962	3,3520055	0,080047068
1924	ENSG00000108239	0,30656538	4,654072059	2,2526729	0,146922255
1925	ENSG00000108256	0,11451032	7,230076907	0,5874511	0,45114327
1926	ENSG00000108262	0,060514918	4,3525402	0,0977791	0,757315189
1927	ENSG00000108298	0,288501228	8,592416909	2,3396564	0,139653478
1928	ENSG00000108306	0,361441772	4,938709817	3,8772246	0,061048463
1929	ENSG00000108312	0,355586016	5,523271873	5,0106727	0,035111873
1930	ENSG00000108344	-0,070853634	5,58402019	0,2303221	0,635789307
1931	ENSG00000108349	0,174159428	5,551210861	1,2630692	0,272608615
1932	ENSG00000108384	-0,294977678	4,279998095	2,7924657	0,108195595
1933	ENSG00000108387	-0,013891043	4,453529912	0,0053928	0,942091121
1934	ENSG00000108389	0,110468673	4,980493425	0,5262746	0,475459053
1935	ENSG00000108395	-0,162961935	5,139006958	2,9246293	0,158722706
1936	ENSG00000108406	0,151024228	5,542069503	0,9301951	0,344795655
1937	ENSG00000108424	0,092167134	7,081749569	0,6633872	0,423650872
1938	ENSG00000108433	-0,107767072	5,225314165	0,8960363	0,353605063
1939	ENSG00000108439	0,038102462	3,730150262	0,0550847	0,868672437
1940	ENSG00000108443	0,05025743	5,37850029	0,2032255	0,65631598
1941	ENSG00000108468	0,106758798	5,127383387	1,2265434	0,358296229
1942	ENSG00000108506	0,166417378	4,70465042	2,7692001	0,169201661
1943	ENSG00000108510	0,224325135	7,065730969	2,438392	0,131939744
1944	ENSG00000108515	-1,030478722	7,589888071	8,948825	0,006497664
1945	ENSG00000108518	-0,077572432	6,261733697	0,195421	0,662551354

1946	ENSG00000108523	-0,22004665	5,247806925	2,0646671	0,164147761
1947	ENSG00000108528	-0,203249232	6,374778783	0,8535112	0,365106087
1948	ENSG00000108559	-0,144533119	4,728347	1,8274528	0,276280147
1949	ENSG00000108561	-0,306378767	5,609336298	6,201324	0,020359524
1950	ENSG00000108578	0,191052004	4,417232554	2,1497097	0,189977419
1951	ENSG00000108582	0,239689648	6,475549741	3,0111541	0,095963503
1952	ENSG00000108587	0,125655879	5,24032668	1,2155576	0,281543319
1953	ENSG00000108588	-0,302910249	6,627321402	6,5219151	0,017683403
1954	ENSG00000108591	0,027632199	3,694900088	0,0290984	0,941217575
1955	ENSG00000108592	-0,246905007	5,195546343	2,6213814	0,118998872
1956	ENSG00000108599	0,060951185	4,841912105	0,3178091	0,57833788
1957	ENSG00000108604	0,197317853	4,757476179	0,6268498	0,436563856
1958	ENSG00000108639	0,153779179	5,261350253	1,0905823	0,307127039
1959	ENSG00000108651	-0,211866894	4,851366812	3,2691379	0,105145477
1960	ENSG00000108654	-0,084229363	9,145770363	0,2843159	0,598959005
1961	ENSG00000108666	-0,179735166	4,274335276	1,2013036	0,284350739
1962	ENSG00000108669	-0,199913281	5,191267065	2,1777508	0,153470938
1963	ENSG00000108671	-0,210393403	5,719587045	3,9239413	0,059581599
1964	ENSG00000108679	-0,299222537	7,61695812	1,0232238	0,322228568
1965	ENSG00000108691	-0,230657961	6,211730664	0,1831127	0,672672673
1966	ENSG00000108773	0,061746294	4,649742867	0,0961932	0,759226025
1967	ENSG00000108788	-0,364223191	5,064327826	5,1564775	0,032788802
1968	ENSG00000108799	0,25025708	5,91583975	2,761662	0,110053088
1969	ENSG00000108821	0,510189482	6,866027268	1,7474247	0,199135598
1970	ENSG00000108823	0,029605401	5,43470048	0,0200722	0,888563186
1971	ENSG00000108826	-0,564324899	4,954375015	15,809396	0,00059007
1972	ENSG00000108828	-0,023987097	5,942223041	0,0133173	0,90912421
1973	ENSG00000108829	0,025848871	5,123625499	0,0250824	0,875537581
1974	ENSG00000108840	0,25956616	5,518417941	2,3638121	0,137756864
1975	ENSG00000108848	-0,038444187	6,816612309	0,0818336	0,777367298
1976	ENSG00000108854	0,324021349	5,154065409	4,7832483	0,039109762
1977	ENSG00000108861	-0,078139459	7,810441691	0,1479925	0,703978272
1978	ENSG00000108883	-0,069481839	5,506465055	0,2015459	0,657657054
1979	ENSG00000108924	0,484294107	5,379999369	8,4849394	0,007808838
1980	ENSG00000108946	0,254798603	9,139933054	1,0518527	0,315695529
1981	ENSG00000108950	-0,472045208	4,358699519	4,6716322	0,041262447
1982	ENSG00000108953	-0,139381588	7,777356806	1,8080058	0,191750769
1983	ENSG00000108960	0,669309303	4,725493098	3,0315702	0,09495605
1984	ENSG00000108984	0,061928515	4,256998557	0,0590297	0,810179389
1985	ENSG00000109016	-0,399808689	4,267167196	7,3174926	0,012586967
1986	ENSG00000109046	0,001259456	7,433149583	5,099E-05	0,994364065
1987	ENSG00000109072	-0,886059894	4,561334346	4,6699214	0,041296409
1988	ENSG00000109079	-0,10813195	5,214878208	0,4515685	0,508253167
1989	ENSG00000109099	-0,578185644	6,891413304	14,706989	0,000838637
1990	ENSG00000109107	-0,667530434	6,593090812	8,9070238	0,00660527
1991	ENSG00000109111	0,230962373	6,335361199	1,7658256	0,196870087
1992	ENSG00000109113	0,567747423	4,635305451	5,6158959	0,026521701
1993	ENSG00000109118	0,120149272	4,257695503	0,2561903	0,617543584
1994	ENSG00000109133	0,007587551	5,805853646	0,0024416	0,961014728
1995	ENSG00000109171	-0,122620935	6,222015703	0,9969011	0,328364808
1996	ENSG00000109180	-0,212609269	6,721475874	3,0304795	0,094965603
1997	ENSG00000109184	-0,062462631	5,949695339	0,1190462	0,733191437
1998	ENSG00000109189	0,114850987	6,085502229	0,2805195	0,601411959
1999	ENSG00000109270	-0,250891885	5,343051707	1,3235432	0,261721329
2000	ENSG00000109320	0,521720669	5,584355952	3,0529572	0,093866364
2001	ENSG00000109323	0,235011678	5,326747105	3,891372	0,060570349
2002	ENSG00000109332	-0,189181629	7,950329117	1,2953796	0,266686787
2003	ENSG00000109339	0,43433366	5,459817146	2,1176795	0,159055496
2004	ENSG00000109381	-0,019858679	6,062580608	0,0211073	0,885742243

2005	ENSG00000109390	-0,571649399	6,108036669	9,1242749	0,006066584
2006	ENSG00000109436	0,482562349	5,207966421	5,3206108	0,030377253
2007	ENSG00000109445	-0,344449179	6,126185621	3,1575756	0,088740137
2008	ENSG00000109452	0,093413636	6,197375712	0,0852329	0,772931809
2009	ENSG00000109458	0,044817881	6,631805098	0,0837868	0,774810465
2010	ENSG00000109466	-0,032654725	4,204494444	0,038979	0,845216275
2011	ENSG00000109472	0,429303759	6,577150247	3,1511885	0,089043666
2012	ENSG00000109475	0,163974883	8,003769257	0,4401982	0,513590691
2013	ENSG00000109501	-0,010870122	5,281024359	0,0026444	0,959429504
2014	ENSG00000109519	-0,27933221	5,035347541	5,3884579	0,029405217
2015	ENSG00000109536	-0,296411525	4,587010631	2,6993998	0,113922668
2016	ENSG00000109572	-0,342866547	6,981076151	5,9303393	0,022977833
2017	ENSG00000109586	0,059844418	3,967342424	0,1802753	0,675054947
2018	ENSG00000109606	-0,228775239	6,723516622	2,1510272	0,155949665
2019	ENSG00000109618	0,008382856	4,220290683	0,0022913	0,962233223
2020	ENSG00000109654	-0,029496841	5,953120994	0,0418257	0,839738868
2021	ENSG00000109670	-0,278171036	6,280245476	1,6214904	0,215542401
2022	ENSG00000109680	0,237468978	4,148784828	2,494377	0,127843126
2023	ENSG00000109685	0,120805115	5,538646566	0,7889514	0,383539406
2024	ENSG00000109686	0,248298924	7,156407452	3,0275392	0,095116651
2025	ENSG00000109689	0,178706218	4,357773653	0,8005621	0,380148798
2026	ENSG00000109738	0,211913693	3,770273452	1,4659043	0,244618104
2027	ENSG00000109756	0,025508822	6,502068175	0,051862	0,821850011
2028	ENSG00000109762	-0,025632517	4,908139824	0,0368918	0,849357262
2029	ENSG00000109771	-0,330792801	3,7665777	1,5615943	0,223938538
2030	ENSG00000109775	-0,157285161	5,161301094	0,830289	0,371594997
2031	ENSG00000109787	0,129898518	5,826486379	0,4217089	0,522487238
2032	ENSG00000109790	0,084220475	4,860583716	0,3446651	0,562832742
2033	ENSG00000109814	0,457810369	5,051308348	1,8251881	0,189775151
2034	ENSG00000109819	-0,338989645	7,081859814	0,9430555	0,341547094
2035	ENSG00000109846	-0,129486578	10,13278379	0,2472629	0,623706406
2036	ENSG00000109854	-0,390420234	3,768681221	8,4404934	0,039450503
2037	ENSG00000109861	-0,250164014	5,781022	1,041666	0,317999108
2038	ENSG00000109906	-0,164162632	6,35859898	0,1236366	0,72830868
2039	ENSG00000109911	-0,151796926	4,833123097	0,7196108	0,404971627
2040	ENSG00000109917	-0,002336219	4,580455103	0,0003398	0,995436344
2041	ENSG00000109919	-0,389930422	5,652228568	9,0908851	0,006130974
2042	ENSG00000109920	-0,290880333	6,260708737	1,5901884	0,219879921
2043	ENSG00000109929	-0,05357284	5,481890998	0,0873581	0,770202022
2044	ENSG00000109971	-0,068950725	9,925447863	0,0798215	0,780051231
2045	ENSG00000110002	0,420755206	3,953879982	3,0998923	0,091525343
2046	ENSG00000110013	-0,415614342	5,615700537	6,6099928	0,017044668
2047	ENSG00000110047	-0,038215145	4,576204875	0,0230015	0,880769324
2048	ENSG00000110048	0,23104854	5,926976574	3,5315551	0,07283436
2049	ENSG00000110066	0,09280904	5,726285877	1,2402261	0,280138131
2050	ENSG00000110074	-0,211738497	4,308084248	1,4495773	0,240781681
2051	ENSG00000110075	-0,159384205	7,014531413	1,7879348	0,194145028
2052	ENSG00000110077	-0,395439141	5,554893703	2,1819097	0,153138261
2053	ENSG00000110079	-0,948568671	4,630704811	13,153962	0,001405674
2054	ENSG00000110080	-0,291722342	5,000033944	4,4907455	0,044994773
2055	ENSG00000110090	0,231067305	5,922174509	1,4696348	0,237650294
2056	ENSG00000110092	0,72396287	6,167291262	13,334192	0,001322649
2057	ENSG00000110107	-0,014388	4,830839568	0,005287	0,942661276
2058	ENSG00000110108	-0,000920707	5,819123151	2,71E-05	0,995890921
2059	ENSG00000110172	-0,099976367	5,715496996	0,2402023	0,628680136
2060	ENSG00000110218	-0,224670628	4,278142208	1,8899122	0,182394294
2061	ENSG00000110237	0,210080167	5,67548246	1,0424383	0,317823662
2062	ENSG00000110274	0,352276377	3,887356862	2,8159861	0,106801919
2063	ENSG00000110315	-0,206862269	5,867550022	1,0102351	0,325253879

2064	ENSG00000110318	0,161655521	4,003822239	0,7674343	0,390025893
2065	ENSG00000110321	0,029396692	9,258653873	0,0447627	0,834289064
2066	ENSG00000110324	0,112379234	4,191294339	0,1482963	0,703689915
2067	ENSG00000110328	0,200436208	4,337819959	2,1376145	0,200857642
2068	ENSG00000110330	-0,06357968	6,03962505	0,1093287	0,743888465
2069	ENSG00000110344	-0,102793329	6,043578777	1,0701203	0,311584319
2070	ENSG00000110367	0,203322932	8,053942963	2,5027192	0,127191774
2071	ENSG00000110395	0,180952981	5,267379451	0,8827747	0,357159046
2072	ENSG00000110422	-0,236120464	8,669285724	2,3363482	0,139909775
2073	ENSG00000110429	-0,088972648	6,030322689	0,3177935	0,578365936
2074	ENSG00000110435	-0,443674966	6,29858493	4,2674496	0,050236709
2075	ENSG00000110442	-0,314805385	4,373679437	5,3964788	0,03428712
2076	ENSG00000110455	0,397448485	3,771673034	2,4243529	0,13305079
2077	ENSG00000110497	0,157446899	4,517896788	0,8502138	0,366017458
2078	ENSG00000110514	0,02279828	5,527209542	0,0106153	0,918827438
2079	ENSG00000110583	-0,290012448	4,720044212	1,8300114	0,18921262
2080	ENSG00000110619	-0,308839914	4,167610376	3,3842744	0,078701182
2081	ENSG00000110651	0,200466579	7,892194779	0,9625258	0,336710032
2082	ENSG00000110665	-0,791722938	4,045417061	8,9851653	0,006405682
2083	ENSG00000110693	0,246563977	5,71574349	2,5267393	0,125517972
2084	ENSG00000110696	-0,014669761	7,128567167	0,0147887	0,904256204
2085	ENSG00000110700	0,099162724	7,954450629	0,2473336	0,623657049
2086	ENSG00000110713	-0,039103731	6,643351358	0,1185649	0,733698655
2087	ENSG00000110717	-0,273128522	5,835516215	1,376387	0,252666642
2088	ENSG00000110721	-0,023950807	3,816490947	0,0140866	0,906548765
2089	ENSG00000110756	0,112053327	5,155324746	0,5498852	0,465833289
2090	ENSG00000110768	0,031077738	5,25095537	0,032366	0,858794766
2091	ENSG00000110799	0,501358192	8,6470907	3,2557388	0,084222213
2092	ENSG00000110841	0,279467189	6,913944422	2,8482961	0,104921434
2093	ENSG00000110851	0,073729079	4,480500024	0,5188966	0,621762069
2094	ENSG00000110852	0,034092607	3,932346884	0,0161319	0,900029972
2095	ENSG00000110871	-0,312899212	4,617920913	5,0110433	0,035075038
2096	ENSG00000110880	0,140555781	6,439907864	0,8357773	0,37004502
2097	ENSG00000110888	-0,006966105	5,199700659	0,0046305	0,9463306
2098	ENSG00000110906	-0,168040675	5,56165034	1,1059828	0,303828444
2099	ENSG00000110911	-0,316395947	5,761708888	6,9181068	0,014900711
2100	ENSG00000110917	-0,096103145	7,491234093	0,3348299	0,568425272
2101	ENSG00000110925	0,070724333	4,883643539	0,2907913	0,667061199
2102	ENSG00000110931	0,166073755	4,69531747	0,8291532	0,371916646
2103	ENSG00000110955	-0,315758581	10,15513024	2,5341674	0,124944193
2104	ENSG00000110958	0,016636047	6,757586609	0,0130277	0,910110869
2105	ENSG00000110987	0,556777783	3,926213618	7,1723925	0,013395226
2106	ENSG00000111011	-0,159588953	6,158869957	1,5954918	0,21909385
2107	ENSG00000111052	-0,202153081	4,563419884	0,8904312	0,355120671
2108	ENSG00000111058	-0,006955651	5,550998712	0,0022412	0,962647812
2109	ENSG00000111077	0,172012787	6,313775178	0,5147083	0,480296366
2110	ENSG00000111142	-0,352575135	6,316574001	2,9716836	0,098086229
2111	ENSG00000111144	-0,077099409	5,693359989	0,4079354	0,529276427
2112	ENSG00000111145	0,040602092	5,242170205	0,0869324	0,770738432
2113	ENSG00000111196	-0,105882877	3,714456037	0,3668891	0,630199543
2114	ENSG00000111203	0,108819039	3,803525866	0,4227922	0,612115252
2115	ENSG00000111224	0,009332171	3,676181972	0,0062501	0,944239513
2116	ENSG00000111229	-0,149607799	5,731518381	0,7270518	0,402590249
2117	ENSG00000111231	-0,224095188	4,370682421	1,5474471	0,225981507
2118	ENSG00000111237	-0,310809071	5,566640334	4,7950891	0,038866829
2119	ENSG00000111245	-0,30589485	7,612471627	0,5148426	0,480239753
2120	ENSG00000111252	0,073398956	5,329536528	0,1641346	0,689103577
2121	ENSG00000111266	0,320875256	5,025652813	5,9606115	0,022665054
2122	ENSG00000111269	0,341805304	5,7778235	3,5557184	0,071971578

2123	ENSG00000111271	0,093324702	4,506277752	0,239517	0,629167737
2124	ENSG00000111275	0,066121841	7,096851354	0,1164343	0,736017044
2125	ENSG00000111276	0,325725619	5,228681911	8,8859528	0,006644355
2126	ENSG00000111300	-0,261997308	5,496823838	4,1134109	0,054180392
2127	ENSG00000111321	-0,223608826	4,426098594	1,0444058	0,31737729
2128	ENSG00000111328	-0,266299394	5,280501929	3,8875064	0,060688933
2129	ENSG00000111331	0,660318015	4,09163719	5,8502024	0,023849362
2130	ENSG00000111335	0,264891147	3,893181748	2,1546195	0,155619471
2131	ENSG00000111341	0,631976439	9,025312612	5,3861006	0,029470936
2132	ENSG00000111348	0,086451852	5,8461501	0,2638416	0,612368875
2133	ENSG00000111358	-0,109906305	4,215000872	0,5864021	0,54528264
2134	ENSG00000111361	-0,053559449	4,762425912	0,216556	0,654967928
2135	ENSG00000111371	-0,395792746	7,754061724	1,564194	0,223565663
2136	ENSG00000111412	0,165582548	4,001118175	1,4772456	0,329129804
2137	ENSG00000111450	-0,072053538	4,967998496	0,260069	0,614898367
2138	ENSG00000111452	-0,148165768	6,666744278	0,3714303	0,548170561
2139	ENSG00000111481	-0,156406847	6,160337112	1,5827872	0,220878085
2140	ENSG00000111530	0,197268852	6,543345258	1,2951847	0,266754636
2141	ENSG00000111540	-0,059396905	6,71838205	0,244755	0,625446539
2142	ENSG00000111554	-0,261157539	3,853797201	2,8579709	0,152515312
2143	ENSG00000111581	-0,145241849	5,104309358	0,8421932	0,368247983
2144	ENSG00000111596	-0,125612711	6,046954406	0,551516	0,465180359
2145	ENSG00000111605	-0,051668885	6,254164373	0,1632468	0,689885984
2146	ENSG00000111615	-0,238618964	5,588562136	1,6466527	0,212133207
2147	ENSG00000111639	-0,537263645	5,895212599	9,3284608	0,005604063
2148	ENSG00000111640	-0,278050425	10,1219724	1,5232699	0,229484893
2149	ENSG00000111642	-0,039105474	4,239991624	0,0592907	0,809768788
2150	ENSG00000111647	0,1791418	5,463870684	1,3302246	0,260553485
2151	ENSG00000111652	-0,028515758	5,384236342	0,052074	0,821492682
2152	ENSG00000111653	0,161651188	4,10063874	1,6824885	0,318528115
2153	ENSG00000111666	-0,656721043	6,499199004	11,8321	0,002221104
2154	ENSG00000111667	0,188612852	5,277916431	1,0988292	0,305361635
2155	ENSG00000111669	-0,487929438	8,439210582	6,0973918	0,021348178
2156	ENSG00000111670	-0,184468199	6,069698116	1,1098167	0,303011018
2157	ENSG00000111674	0,414542063	4,483462541	2,8132522	0,106962835
2158	ENSG00000111676	0,153818758	5,456744444	0,3722689	0,547722799
2159	ENSG00000111707	0,16878439	5,422595831	3,4898911	0,090398962
2160	ENSG00000111711	-0,013512282	5,339888714	0,0038793	0,950872022
2161	ENSG00000111716	-0,723491759	6,776874875	3,3921572	0,078376362
2162	ENSG00000111725	0,069121406	3,901918324	0,264075	0,740289507
2163	ENSG00000111726	-0,110108095	5,025652755	1,081778	0,402437687
2164	ENSG00000111727	-0,044459381	5,203927712	0,0460146	0,83203051
2165	ENSG00000111731	-0,048417971	5,690885772	0,1346377	0,716997551
2166	ENSG00000111737	0,030447977	4,246572963	0,0453218	0,936406003
2167	ENSG00000111752	0,236233004	4,521764581	1,2321747	0,278400224
2168	ENSG00000111775	0,158344362	4,52744853	0,5932199	0,448978085
2169	ENSG00000111785	-0,053821275	4,987316751	0,1751118	0,679457556
2170	ENSG00000111786	0,114929641	4,061066906	0,6983725	0,520677702
2171	ENSG00000111790	0,097388904	5,818277102	0,254299	0,618837811
2172	ENSG00000111799	-0,373635841	6,951609896	2,2181693	0,149914602
2173	ENSG00000111801	0,552726313	4,429532383	4,4865291	0,045126301
2174	ENSG00000111802	-0,046773336	5,109785727	0,1233894	0,72855796
2175	ENSG00000111816	0,195401753	4,697333823	1,2550896	0,27408919
2176	ENSG00000111817	0,299189913	5,827262375	0,9814149	0,332108308
2177	ENSG00000111832	-0,424087923	5,990257066	5,7717293	0,024709406
2178	ENSG00000111843	0,012955068	6,059551851	0,0070499	0,933808306
2179	ENSG00000111845	-0,304021744	3,89825739	3,5189085	0,101191566
2180	ENSG00000111846	0,949061182	3,721134794	13,302678	0,00133677
2181	ENSG00000111850	-0,180806153	4,845211624	0,7313384	0,401227986

2182	ENSG00000111859	0,275476667	5,705899957	0,9699909	0,334880841
2183	ENSG00000111860	-0,544182351	5,256846162	7,7545188	0,010504787
2184	ENSG00000111875	-0,373206995	4,664173756	3,1539529	0,088912148
2185	ENSG00000111877	0,113995559	4,055236863	0,8327539	0,469995685
2186	ENSG00000111880	-0,093016955	4,64343952	0,4023238	0,539341592
2187	ENSG00000111885	-0,249804685	6,232120852	0,8433205	0,367933313
2188	ENSG00000111897	-0,237435404	8,556311532	1,1061463	0,30379352
2189	ENSG00000111906	-0,530437596	5,50267449	10,95232	0,00304614
2190	ENSG00000111907	-0,679691588	5,15429592	11,9233	0,00215071
2191	ENSG00000111911	-0,139487407	5,265420367	0,828639	0,372062395
2192	ENSG00000111912	0,474011829	5,365774297	3,0418978	0,094428029
2193	ENSG00000111961	0,057420595	6,246533843	0,0666024	0,798633592
2194	ENSG00000112031	-0,502194638	4,481284814	17,180184	0,000515688
2195	ENSG00000112062	-0,024976039	5,900978471	0,0349514	0,853323145
2196	ENSG00000112078	-0,013501866	6,190082208	0,0089371	0,925495882
2197	ENSG00000112079	-0,174641461	5,49375136	1,1759588	0,289362762
2198	ENSG00000112081	-0,11726384	7,168563552	0,6274228	0,436335781
2199	ENSG00000112096	0,01370476	8,81228492	0,0042167	0,948783032
2200	ENSG00000112110	-0,334244687	4,754932453	6,0935035	0,021358313
2201	ENSG00000112118	0,037360267	5,137011042	0,0861732	0,981294343
2202	ENSG00000112130	-0,133266552	4,550108943	1,7582325	0,316278255
2203	ENSG00000112137	-0,42244399	3,990158011	1,6811538	0,207567519
2204	ENSG00000112144	0,357701859	4,858877532	4,3330906	0,048639992
2205	ENSG00000112146	0,125441701	5,736398094	1,1326769	0,29816004
2206	ENSG00000112159	0,006378568	6,848674717	0,0017582	0,966913506
2207	ENSG00000112183	0,277402819	7,039718559	0,7920075	0,382663743
2208	ENSG00000112186	0,222398692	6,12098675	1,0449626	0,317251128
2209	ENSG00000112200	0,076612349	6,491060323	0,2068197	0,653514263
2210	ENSG00000112208	-0,352509975	5,176770369	3,3978777	0,07814161
2211	ENSG00000112210	0,497979182	4,176422159	4,9459212	0,036202247
2212	ENSG00000112234	0,019737357	5,800462063	0,0210253	0,885962824
2213	ENSG00000112237	-0,03412853	5,25385251	0,039038	0,845100737
2214	ENSG00000112242	0,054827863	3,593834574	0,1360919	0,800363469
2215	ENSG00000112249	-0,010570506	6,558744181	0,0045965	0,946529785
2216	ENSG00000112276	-0,202165405	5,882414572	0,5824735	0,45306454
2217	ENSG00000112282	0,001019945	5,204946435	6,752E-05	0,99351389
2218	ENSG00000112294	0,286894189	4,713917858	2,36637	0,137554068
2219	ENSG00000112297	-0,05555152	4,142128724	0,0376141	0,847914476
2220	ENSG00000112304	-0,507625251	4,998376229	13,58435	0,001211824
2221	ENSG00000112305	-0,102793654	3,720417638	0,6419423	0,57675898
2222	ENSG00000112306	0,203432062	8,261619715	0,6043872	0,444794
2223	ENSG00000112308	0,257396304	6,49218693	2,8564319	0,104449801
2224	ENSG00000112319	0,337863048	4,672237937	0,6403865	0,43171976
2225	ENSG00000112320	0,005386303	4,673426334	0,001111	0,973695864
2226	ENSG00000112335	-0,203003488	7,061932939	2,0894509	0,161696507
2227	ENSG00000112339	-0,339108983	6,196934075	2,7552391	0,110445074
2228	ENSG00000112343	0,208727029	4,853298269	0,8721594	0,360012906
2229	ENSG00000112367	-0,049529258	4,085360412	0,1955938	0,823365612
2230	ENSG00000112378	-0,246699158	6,39934602	1,9280419	0,178210553
2231	ENSG00000112379	0,774854668	4,326664238	3,8373793	0,062293892
2232	ENSG00000112406	-0,223575514	6,061627316	1,5690431	0,22287223
2233	ENSG00000112414	1,021444344	4,624210832	5,2270282	0,031726918
2234	ENSG00000112419	0,240474686	6,919684289	1,9523959	0,175599577
2235	ENSG00000112425	-0,154805089	5,33437476	0,8877546	0,355831348
2236	ENSG00000112473	0,015853047	5,970216851	0,0179741	0,894505732
2237	ENSG00000112511	0,056663178	5,331557245	0,161132	0,691793998
2238	ENSG00000112514	0,07133617	5,031159448	0,1948554	0,663007961
2239	ENSG00000112531	-0,109364804	8,775451663	0,3517319	0,558873522
2240	ENSG00000112541	-0,417132298	4,674062702	1,5761903	0,221855119

2241	ENSG00000112562	0,239213443	5,842488657	1,6861008	0,206922947
2242	ENSG00000112576	-0,128008114	4,932685887	0,5580391	0,462583686
2243	ENSG00000112584	0,06898117	5,439159505	0,4071045	0,529692529
2244	ENSG00000112624	0,275632929	4,906993776	3,0689433	0,093061313
2245	ENSG00000112640	0,062839357	4,090776861	0,1297501	0,765396426
2246	ENSG00000112658	-0,0987092	5,945230792	0,2729036	0,606362136
2247	ENSG00000112659	0,241478633	4,048576487	1,1828525	0,28798795
2248	ENSG00000112679	-0,417878583	5,34783026	11,933484	0,002135224
2249	ENSG00000112685	0,059916593	5,241183541	0,208482	0,652207
2250	ENSG00000112695	-0,435003629	7,02611445	3,7562199	0,064921184
2251	ENSG00000112697	0,069653535	7,105731579	0,1835382	0,672316163
2252	ENSG00000112701	0,055601346	6,905753688	0,1622796	0,690755408
2253	ENSG00000112715	-0,201129753	6,923754007	0,62712	0,436466315
2254	ENSG00000112739	-0,067670137	6,325700041	0,2486196	0,622744572
2255	ENSG00000112759	0,047657808	6,386729676	0,0759487	0,785313452
2256	ENSG00000112763	-0,120862148	4,536220182	1,1292543	0,349247458
2257	ENSG00000112769	0,517707177	7,43666227	4,0558366	0,055802425
2258	ENSG00000112773	-0,022247935	6,284713162	0,0255698	0,874340192
2259	ENSG00000112782	0,060124582	7,803831873	0,0591032	0,810063708
2260	ENSG00000112837	1,793704814	4,857795856	12,313388	0,001875997
2261	ENSG00000112851	0,017114077	7,159870422	0,0112311	0,916515036
2262	ENSG00000112855	0,005370256	4,722141228	0,0020771	0,99760122
2263	ENSG00000112874	-0,478238951	4,729589745	3,4728548	0,075138156
2264	ENSG00000112893	0,124057557	5,422135315	0,4483178	0,509769043
2265	ENSG00000112902	0,017523761	6,206309642	0,016077	0,900195305
2266	ENSG00000112936	0,601135732	9,569112652	3,6196102	0,069634067
2267	ENSG00000112941	0,171202416	4,267416532	1,1093696	0,303106193
2268	ENSG00000112964	0,07913739	5,760612918	0,0722382	0,790489989
2269	ENSG00000112972	-0,16551121	4,920815019	1,0883111	0,307635027
2270	ENSG00000112977	0,3317955	5,339302845	3,9726062	0,058180492
2271	ENSG00000112983	0,151856125	5,062763683	1,5528961	0,225148731
2272	ENSG00000112992	-0,262580041	8,487416557	0,9151112	0,348661635
2273	ENSG00000112996	-0,160251069	5,977416696	1,1024243	0,30458982
2274	ENSG00000113013	-0,19936178	8,203764501	1,3218231	0,261991264
2275	ENSG00000113048	-0,063528718	5,990358624	0,3061569	0,585342756
2276	ENSG00000113068	-0,179599895	5,239435608	1,7829988	0,194739472
2277	ENSG00000113070	0,579364824	4,778588441	3,40674	0,077779518
2278	ENSG00000113083	0,818375864	5,115752211	4,3979273	0,047118896
2279	ENSG00000113140	0,739228005	9,115339324	6,4519551	0,018259552
2280	ENSG00000113141	0,013929627	6,166330016	0,0099372	0,921451105
2281	ENSG00000113161	0,023206083	4,490335183	0,0229158	0,880989664
2282	ENSG00000113163	0,123367157	5,958234931	0,8962502	0,353548669
2283	ENSG00000113194	0,061100819	5,133927384	0,2850434	0,598494692
2284	ENSG00000113231	0,441889249	4,536592954	2,8895985	0,102573494
2285	ENSG00000113240	-0,193927596	5,47359642	1,1787196	0,288811137
2286	ENSG00000113269	-0,091624748	5,667411512	0,4205808	0,523021037
2287	ENSG00000113273	0,335434687	4,44087989	4,1824462	0,05239322
2288	ENSG00000113282	-0,204717465	6,368397999	3,0353502	0,094715995
2289	ENSG00000113296	0,257108662	5,647751858	1,0620535	0,313411552
2290	ENSG00000113300	0,031975756	4,994280468	0,0714668	0,791575741
2291	ENSG00000113312	-0,423758514	5,863381306	5,6951882	0,025581693
2292	ENSG00000113318	0,359107782	4,828119455	4,934528	0,036398003
2293	ENSG00000113319	-0,462371618	4,681654338	6,2782489	0,019706936
2294	ENSG00000113328	-0,221326347	6,739034998	0,8837374	0,35690182
2295	ENSG00000113360	0,097098036	5,207209902	0,8321641	0,439610246
2296	ENSG00000113369	-0,014418761	6,31398647	0,0026394	0,959468364
2297	ENSG00000113384	0,020769827	5,508623765	0,0290376	0,866169878
2298	ENSG00000113387	-0,23556039	7,280726254	0,7024317	0,410551674
2299	ENSG00000113389	2,475951894	5,635222488	30,250428	1,33872E-05

2300	ENSG00000113391	0,003074907	5,027310456	0,0005006	0,982340658
2301	ENSG00000113396	-1,423561261	5,865392766	31,817966	9,49025E-06
2302	ENSG00000113407	-0,147609247	6,145012285	0,8075767	0,378104643
2303	ENSG00000113441	0,011523201	8,044200991	0,0069478	0,93428583
2304	ENSG00000113448	-0,719156446	6,850856434	8,0244132	0,009404525
2305	ENSG00000113456	-0,65228949	5,079108138	25,793645	3,74789E-05
2306	ENSG00000113460	-0,267460838	4,205565903	2,4518184	0,130978806
2307	ENSG00000113504	0,008805999	5,520633678	0,0021155	0,963709636
2308	ENSG00000113532	0,04226716	4,043831356	0,023012	0,88074218
2309	ENSG00000113552	0,036927688	4,470454072	0,1167326	0,794757118
2310	ENSG00000113555	-0,022875313	4,125228658	0,019819	0,889263558
2311	ENSG00000113558	-0,134482574	7,693795983	0,6000777	0,446401232
2312	ENSG00000113569	-0,045102791	5,268895517	0,170429	0,822385736
2313	ENSG00000113575	0,000671695	6,814912791	2,104E-05	0,996379025
2314	ENSG00000113578	0,395991926	5,805841139	2,52296	0,125786916
2315	ENSG00000113580	0,229743438	6,936680576	2,8970609	0,102109092
2316	ENSG00000113583	-0,113306592	5,415802958	0,4949276	0,488764522
2317	ENSG00000113593	0,123351087	5,175315546	0,7332452	0,400613943
2318	ENSG00000113594	-0,1714307	7,799877928	0,7305737	0,401470491
2319	ENSG00000113595	-0,151196213	5,205524525	0,4982282	0,487333944
2320	ENSG00000113597	-0,410057383	4,901661161	2,929696	0,100352364
2321	ENSG00000113615	0,29895506	5,073034816	3,453004	0,075920255
2322	ENSG00000113621	-0,260675712	5,927626873	6,6638963	0,016624928
2323	ENSG00000113638	-0,131118855	5,075273456	0,3767442	0,545345138
2324	ENSG00000113643	-0,132689663	6,153108999	0,3118978	0,58188853
2325	ENSG00000113648	-0,262981783	6,332104577	4,299149	0,049417912
2326	ENSG00000113649	-0,225065399	5,62462369	2,8692609	0,103675106
2327	ENSG00000113657	0,207307554	7,199526101	0,4731262	0,498397964
2328	ENSG00000113658	-0,026749426	6,470174163	0,0242601	0,877577352
2329	ENSG00000113712	-0,148319358	6,782257984	1,5564446	0,224636321
2330	ENSG00000113716	0,006200227	4,851228813	0,0013742	0,970747645
2331	ENSG00000113719	0,029208149	6,153902377	0,0460011	0,832048299
2332	ENSG00000113721	-0,028688352	6,601374219	0,0117259	0,914703224
2333	ENSG00000113732	-0,187248214	6,42659258	1,7628747	0,197189017
2334	ENSG00000113739	-1,131222762	4,331531915	19,094574	0,00022189
2335	ENSG00000113742	0,068701506	7,462180106	0,081865	0,777334649
2336	ENSG00000113749	-0,811354771	4,077489701	7,1884273	0,013304723
2337	ENSG00000113758	-0,560806284	4,460187136	4,3544022	0,048133979
2338	ENSG00000113790	-0,137197755	4,259452454	0,433369	0,516844744
2339	ENSG00000113810	0,44439886	4,675363179	4,5589861	0,043567254
2340	ENSG00000113811	-0,424742508	5,942655012	2,9053429	0,101694606
2341	ENSG00000113812	-0,174708399	5,100533769	1,0763205	0,310254784
2342	ENSG00000113845	-0,50453204	6,457292991	6,8022213	0,015686179
2343	ENSG00000113851	-0,140502336	6,019961999	0,4211109	0,522779655
2344	ENSG00000113916	-0,834209546	5,93100484	9,5556175	0,005134556
2345	ENSG00000113971	0,117020391	3,850427022	0,4749853	0,497563733
2346	ENSG00000114019	0,544726793	5,334814105	3,3839301	0,078715403
2347	ENSG00000114021	-0,311464004	5,217576889	7,8309692	0,010159143
2348	ENSG00000114023	-0,483081677	6,226793828	6,3014091	0,019506793
2349	ENSG00000114030	-0,069769208	5,857866979	0,3644565	0,551900749
2350	ENSG00000114054	0,055024971	5,851920288	0,076155	0,785029778
2351	ENSG00000114062	-0,036493545	7,108447227	0,0809498	0,778539218
2352	ENSG00000114098	-0,289487341	5,791252435	4,7931362	0,038891484
2353	ENSG00000114107	-0,171859577	5,350919769	0,7993857	0,380493212
2354	ENSG00000114120	-0,245015901	7,065940343	1,3146625	0,263284138
2355	ENSG00000114125	-0,009930444	4,988685668	0,0077131	0,996319027
2356	ENSG00000114126	0,133245858	6,326378864	1,5556365	0,224752888
2357	ENSG00000114127	-0,00224073	6,655233766	0,000222	0,988239018
2358	ENSG00000114166	-0,236695129	6,484290839	1,95334	0,175499304

2359	ENSG00000114200	0,461446926	4,766291634	0,9255607	0,345976981
2360	ENSG00000114209	-0,317377462	5,10922128	2,3108933	0,142033261
2361	ENSG00000114279	-0,558039501	8,49357504	1,370244	0,253698195
2362	ENSG00000114302	-0,021268901	6,000843538	0,030986	0,861799565
2363	ENSG00000114315	1,096904232	4,303819532	12,998222	0,00148202
2364	ENSG00000114316	0,020557741	5,483059939	0,0300969	0,863775752
2365	ENSG00000114331	0,106778171	6,115536923	0,4195517	0,523543411
2366	ENSG00000114346	-0,348313143	3,610438585	2,7806996	0,108900744
2367	ENSG00000114353	0,064256835	6,59813378	0,1554088	0,697032973
2368	ENSG00000114354	-0,030948322	6,118237615	0,0740765	0,787899501
2369	ENSG00000114374	0,105438964	6,431634071	0,0464356	0,831276181
2370	ENSG00000114378	-0,633677781	4,364933376	7,7926099	0,010341233
2371	ENSG00000114383	-0,273112551	4,226676791	5,0387408	0,079958261
2372	ENSG00000114391	0,05385091	7,884832684	0,1093649	0,743837267
2373	ENSG00000114416	-0,198047546	7,333209755	1,4065078	0,24768622
2374	ENSG00000114423	-0,549450538	6,394333704	8,4369517	0,007960306
2375	ENSG00000114439	0,128538162	6,715241517	1,7819773	0,194862772
2376	ENSG00000114446	0,288282125	4,684860302	2,6987442	0,113964257
2377	ENSG00000114450	0,089425326	5,559683488	0,1925708	0,664860362
2378	ENSG00000114480	-0,061585176	6,225851429	0,1038694	0,750131842
2379	ENSG00000114491	0,130399594	3,86639552	1,1556854	0,453252523
2380	ENSG00000114503	0,093279774	5,30329722	0,8660602	0,361634154
2381	ENSG00000114520	-0,104415392	4,792193023	0,2320813	0,634515009
2382	ENSG00000114541	0,150616303	6,090724343	0,4888579	0,491414127
2383	ENSG00000114544	0,063719105	4,645052516	0,1412682	0,710451246
2384	ENSG00000114554	-0,202230823	4,662545756	0,7350568	0,400051879
2385	ENSG00000114573	0,027242396	5,505584276	0,0307943	0,862228324
2386	ENSG00000114648	-0,157506563	4,121806624	1,4262581	0,29846644
2387	ENSG00000114650	0,075301051	4,798989905	0,1374156	0,714239661
2388	ENSG00000114686	-0,332687426	5,476777443	2,5385284	0,124683382
2389	ENSG00000114698	0,732921215	5,763864809	5,5674763	0,027114629
2390	ENSG00000114735	0,039603928	4,132586483	0,0544423	0,882259503
2391	ENSG00000114738	-0,307855616	7,094459343	2,0697808	0,163648056
2392	ENSG00000114739	0,207887387	4,751607064	1,5524328	0,225258854
2393	ENSG00000114742	0,103533498	5,568448573	0,5067336	0,483674065
2394	ENSG00000114744	-0,019831723	4,660490313	0,0122875	0,912693201
2395	ENSG00000114745	0,062423404	4,885917046	0,2161744	0,740807642
2396	ENSG00000114770	-0,403052699	6,608652877	2,5238382	0,125724359
2397	ENSG00000114779	0,403721673	4,085743327	4,1448827	0,053379427
2398	ENSG00000114784	-0,245930715	6,686753881	1,4371878	0,242742329
2399	ENSG00000114790	0,465643507	3,767477385	1,9058928	0,180626376
2400	ENSG00000114796	0,003839396	7,946948709	0,0002358	0,987881227
2401	ENSG00000114841	0,226491793	3,770280641	0,6645317	0,423285696
2402	ENSG00000114850	-0,141869488	7,240927837	0,5675594	0,458836364
2403	ENSG00000114853	0,502472681	5,598581231	5,9662835	0,022637614
2404	ENSG00000114854	-0,21761668	9,96683425	0,4721307	0,498845668
2405	ENSG00000114857	-0,273408365	7,3229263	2,9304541	0,100310911
2406	ENSG00000114861	-0,077832769	6,031275562	0,2251647	0,639585207
2407	ENSG00000114867	-0,092274229	7,342200566	0,1899331	0,667015609
2408	ENSG00000114902	-0,113379855	5,19084922	0,578253	0,454683465
2409	ENSG00000114904	0,270097545	4,247824677	6,0570476	0,079509184
2410	ENSG00000114923	-0,147836093	7,891803182	0,3534529	0,557940502
2411	ENSG00000114933	0,028918103	5,983700657	0,0856573	0,772374652
2412	ENSG00000114942	0,191844237	7,346720551	0,8636315	0,362329285
2413	ENSG00000114948	-0,234300513	5,685186491	0,9630767	0,336574565
2414	ENSG00000114978	0,064014289	6,6546616	0,1723316	0,68187628
2415	ENSG00000114982	0,149735315	5,818240322	0,9373861	0,342973877
2416	ENSG00000114988	0,081590309	4,347703873	0,3190406	0,651659636
2417	ENSG00000114999	-0,248407107	5,697227172	4,3901153	0,047259406

2418	ENSG00000115020	-0,041406373	6,244676068	0,1004773	0,754093182
2419	ENSG00000115042	-0,087976821	4,504319583	0,3513311	0,559104074
2420	ENSG00000115053	0,161664943	7,550679209	1,8395294	0,188063274
2421	ENSG00000115073	0,148221824	5,333019967	0,9763161	0,333338592
2422	ENSG00000115084	0,301905287	7,039929641	2,0336126	0,167222334
2423	ENSG00000115091	0,013452149	7,04125255	0,0048171	0,945264031
2424	ENSG00000115109	0,066374745	5,344699584	0,4038541	0,531326086
2425	ENSG00000115128	-0,215007324	5,209997117	0,7583287	0,392807296
2426	ENSG00000115137	-0,405156956	4,343227622	9,4352535	0,006276898
2427	ENSG00000115145	-0,139192431	5,865216421	0,6510672	0,427956892
2428	ENSG00000115159	-0,124007192	5,193051081	0,6212799	0,438581952
2429	ENSG00000115170	0,100600625	4,961725109	0,4485872	0,509643139
2430	ENSG00000115183	0,151041806	7,047614942	1,1345346	0,297773418
2431	ENSG00000115204	0,036211231	4,601337588	0,1102653	0,791910812
2432	ENSG00000115207	0,078872444	4,248667647	0,2639028	0,70005839
2433	ENSG00000115211	-0,231802333	4,335481506	3,2549571	0,116135981
2434	ENSG00000115216	0,085382304	5,788341681	0,5806327	0,453743832
2435	ENSG00000115233	-0,486587408	5,733902511	4,8878391	0,037212707
2436	ENSG00000115234	0,194684215	5,625001072	2,0986049	0,160819025
2437	ENSG00000115239	-0,015380036	4,357682707	0,0116065	0,993524372
2438	ENSG00000115241	-0,185349716	5,120832123	1,7517396	0,198564586
2439	ENSG00000115252	0,764591337	4,554366527	7,1828747	0,013335986
2440	ENSG00000115267	0,325317429	3,924961078	2,3072227	0,142335701
2441	ENSG00000115268	0,111448522	6,498926817	0,3112693	0,582266664
2442	ENSG00000115271	-0,126653996	4,624008723	0,1710292	0,68301089
2443	ENSG00000115275	0,144192425	4,112050235	0,6158042	0,440580397
2444	ENSG00000115295	0,059202442	6,752611673	0,1511312	0,701013746
2445	ENSG00000115306	0,344732353	9,942150777	2,5252034	0,125580033
2446	ENSG00000115307	-0,170258034	5,357283988	1,0292125	0,32084671
2447	ENSG00000115310	-0,342138268	8,002162925	6,3161179	0,019352803
2448	ENSG00000115355	-0,061005479	6,582726457	0,3747182	0,546398358
2449	ENSG00000115364	-0,047628558	5,730889527	0,2075949	0,652895997
2450	ENSG00000115365	-0,140555395	6,396502701	0,5769137	0,455202358
2451	ENSG00000115368	-0,288835404	5,32029041	2,3556166	0,138409013
2452	ENSG00000115380	1,67968162	7,797983854	11,431818	0,002561361
2453	ENSG00000115392	-0,087932205	4,389507817	0,2252204	0,639544004
2454	ENSG00000115414	1,039457141	9,769688041	11,546364	0,002458503
2455	ENSG00000115415	0,472912958	6,720901561	5,6777091	0,025785671
2456	ENSG00000115419	0,515629041	7,272001654	3,5074215	0,073798203
2457	ENSG00000115421	0,003234075	4,587910935	0,0007086	0,978990668
2458	ENSG00000115446	0,06568892	4,570337305	0,2828788	0,599878417
2459	ENSG00000115457	0,674471644	5,965131756	4,0426353	0,056172143
2460	ENSG00000115459	0,419153098	4,241584045	3,3077581	0,081935803
2461	ENSG00000115461	-0,19194713	8,332696761	0,2740346	0,605621406
2462	ENSG00000115464	-0,065460017	7,709819291	0,1863889	0,669925963
2463	ENSG00000115484	-0,058908085	6,20586407	0,2548457	0,618446487
2464	ENSG00000115486	0,091098846	5,189834441	0,2568503	0,617093402
2465	ENSG00000115504	-0,012100125	5,713424304	0,0034582	0,953611944
2466	ENSG00000115514	-0,282522284	4,644328389	1,2825637	0,269035353
2467	ENSG00000115520	-0,137265365	5,134001393	0,5011718	0,486064116
2468	ENSG00000115524	-0,131662761	8,407089089	0,6349521	0,433626134
2469	ENSG00000115525	0,010597264	4,35539092	0,0011167	0,973628988
2470	ENSG00000115526	0,149839971	3,735976175	0,5982618	0,492750137
2471	ENSG00000115539	-0,223020887	3,963925874	2,2268853	0,223474713
2472	ENSG00000115540	-0,576391701	5,230255633	4,0683019	0,055455848
2473	ENSG00000115541	-0,424269922	4,76414609	4,3924494	0,047245312
2474	ENSG00000115548	-0,034620264	6,319890387	0,085221	0,77293811
2475	ENSG00000115561	-0,006043936	6,01173119	0,0028391	0,957962613
2476	ENSG00000115593	-0,622183881	7,182672859	5,2138205	0,031922748

2477	ENSG00000115594	0,428233766	6,654581629	1,6080737	0,217388277
2478	ENSG00000115641	0,965914587	6,545055458	7,027174	0,014246601
2479	ENSG00000115648	-0,084001353	5,99824416	0,0930207	0,763101066
2480	ENSG00000115649	-0,046641369	4,913246607	0,099476	0,755286276
2481	ENSG00000115652	-0,074155046	4,4967121	0,5083326	0,559582432
2482	ENSG00000115677	0,141158247	8,631184352	1,1633396	0,291863732
2483	ENSG00000115685	-0,043738606	5,355189465	0,1264226	0,725383724
2484	ENSG00000115694	-0,066752955	5,34923126	0,1999959	0,658882323
2485	ENSG00000115738	1,048613166	5,91965427	8,7031436	0,007158993
2486	ENSG00000115750	0,037270408	3,694037204	0,0492215	0,835980126
2487	ENSG00000115758	0,579312305	5,720686871	8,6494796	0,0073131
2488	ENSG00000115760	-0,012648088	8,087234733	0,0090514	0,925021555
2489	ENSG00000115761	-0,133028354	4,608497175	2,0953974	0,342671106
2490	ENSG00000115762	-0,196078455	5,88746351	4,0017788	0,057291973
2491	ENSG00000115806	0,077310436	5,397849408	0,5042337	0,553249262
2492	ENSG00000115808	0,286292794	6,720422452	2,4209143	0,133312926
2493	ENSG00000115816	-0,148367464	5,461011803	0,3716839	0,548035067
2494	ENSG00000115825	0,135803647	5,817580887	0,7829862	0,385342498
2495	ENSG00000115827	0,114265937	4,289446874	0,8222418	0,374003119
2496	ENSG00000115839	0,003042412	6,584504733	0,0006971	0,979161408
2497	ENSG00000115840	-0,42577293	6,988293992	3,7393959	0,06548148
2498	ENSG00000115866	-0,170135199	6,291267483	0,9456293	0,340902119
2499	ENSG00000115875	-0,152311514	6,10480569	0,9861455	0,330969566
2500	ENSG00000115896	0,218410779	5,875919017	2,6073393	0,119893342
2501	ENSG00000115904	0,148268798	5,997163185	0,923333	0,346546864
2502	ENSG00000115935	0,084043136	5,208436518	0,2901277	0,595288961
2503	ENSG00000115942	-0,06427019	5,1560023	0,2236123	0,640723694
2504	ENSG00000115944	-0,154922865	6,050136531	1,7592822	0,197627257
2505	ENSG00000115946	-0,24301229	3,768591159	3,4426407	0,201366723
2506	ENSG00000115947	-0,26983081	5,420026759	2,4583005	0,130495386
2507	ENSG00000115956	-0,544868764	4,535770578	3,501119	0,074040461
2508	ENSG00000115963	-0,157462933	5,54947067	0,3696915	0,549101132
2509	ENSG00000115966	-0,005123889	5,851646036	0,0006811	0,979403581
2510	ENSG00000115970	-0,053221883	5,830469562	0,1806696	0,674715878
2511	ENSG00000115977	0,106797002	6,770185241	0,2556415	0,617918529
2512	ENSG00000115993	0,160291628	7,826351627	0,6559927	0,426238854
2513	ENSG00000116001	-0,115824598	6,28262488	0,4591028	0,504770258
2514	ENSG00000116005	0,043902723	6,849746516	0,1225081	0,729488437
2515	ENSG00000116016	0,156835762	7,917400298	0,6317574	0,43479784
2516	ENSG00000116030	-0,246579112	5,962789237	2,2576138	0,146499626
2517	ENSG00000116044	0,340737763	6,7814364	4,2387423	0,050953571
2518	ENSG00000116062	0,008531499	5,312119187	0,0065897	0,935997852
2519	ENSG00000116095	0,144427327	5,816395674	1,6898953	0,206385948
2520	ENSG00000116096	-0,048640197	4,445773859	0,0603468	0,808116986
2521	ENSG00000116106	0,005695765	6,8990206	0,0005034	0,982291917
2522	ENSG00000116117	0,280609256	5,148629965	2,6702495	0,115789003
2523	ENSG00000116120	-0,287195514	5,176079705	6,5058667	0,017807672
2524	ENSG00000116127	0,135238782	6,192038236	1,3943995	0,249631482
2525	ENSG00000116128	0,15073167	4,018348944	0,4014934	0,532539902
2526	ENSG00000116132	-0,533996433	6,357958001	7,3987102	0,012178366
2527	ENSG00000116133	0,188894619	4,893436962	0,1743322	0,680143167
2528	ENSG00000116138	-0,089167187	4,826137985	0,729383	0,459207689
2529	ENSG00000116141	-0,140848413	4,808318997	0,975088	0,333625471
2530	ENSG00000116161	-0,438211666	5,413070854	8,2568536	0,008558317
2531	ENSG00000116171	-0,116009124	6,9190737	0,4416425	0,512907175
2532	ENSG00000116191	-0,568219689	4,841866143	13,064065	0,001449203
2533	ENSG00000116194	-0,36657179	4,641688866	2,2521649	0,146965797
2534	ENSG00000116198	0,251428818	4,383031008	2,839006	0,107491209
2535	ENSG00000116199	-0,020516245	6,568869241	0,0538993	0,81844826

2536	ENSG00000116205	0,217770522	3,664808083	3,3109422	0,259572242
2537	ENSG00000116209	-0,325866867	7,708961233	3,6944366	0,066997576
2538	ENSG00000116212	-0,184164752	3,966988062	1,9497069	0,280809412
2539	ENSG00000116221	-0,433859223	5,327099779	8,7785562	0,006938763
2540	ENSG00000116237	-0,399086616	6,551007911	7,5017852	0,011648468
2541	ENSG00000116251	0,15120685	7,131085835	0,4976715	0,487574718
2542	ENSG00000116260	-0,415567089	6,770512244	2,7598547	0,110163221
2543	ENSG00000116266	-0,137437555	5,551334027	0,490214	0,49081999
2544	ENSG00000116285	0,482892061	5,389499409	0,9706266	0,33472571
2545	ENSG00000116288	-0,309482603	6,934960954	3,6712093	0,067809498
2546	ENSG00000116350	-0,189051903	6,076277645	1,6684966	0,2092102
2547	ENSG00000116353	-0,172663868	3,942504124	1,0764406	0,310228373
2548	ENSG00000116396	0,474112593	3,794222642	2,6751212	0,115474611
2549	ENSG00000116406	0,085356404	5,885598715	0,3082131	0,584112504
2550	ENSG00000116455	-0,181859726	4,153055275	1,7269133	0,215954723
2551	ENSG00000116459	-0,625221752	8,236059631	6,6180763	0,016984983
2552	ENSG00000116473	-0,253698178	6,255968582	2,5659362	0,122768254
2553	ENSG00000116478	-0,005979738	5,066586214	0,0033182	0,954557655
2554	ENSG00000116489	-0,038195148	6,062192277	0,0441966	0,835330451
2555	ENSG00000116497	0,097296042	4,575964124	0,4380201	0,514615313
2556	ENSG00000116514	0,303156258	3,915927633	2,1826432	0,153072234
2557	ENSG00000116521	-0,171945818	4,78163101	1,1440941	0,295832708
2558	ENSG00000116539	0,133087303	7,515025206	1,6807557	0,207575278
2559	ENSG00000116560	0,094791572	7,491692526	0,3685724	0,549687291
2560	ENSG00000116574	0,07946486	4,914981962	0,0485813	0,827485841
2561	ENSG00000116580	0,113810333	5,966355483	0,8041331	0,379074165
2562	ENSG00000116584	0,238346933	5,404460948	1,7227206	0,202228166
2563	ENSG00000116604	0,162198032	5,686394961	0,552577	0,464756381
2564	ENSG00000116641	0,045912609	5,643238633	0,1301491	0,721542468
2565	ENSG00000116667	-0,113583213	6,669712037	0,4428643	0,512330204
2566	ENSG00000116668	0,188245623	4,407604271	0,7903428	0,383155975
2567	ENSG00000116675	0,172195373	4,569369365	0,6065897	0,443976147
2568	ENSG00000116678	1,275017437	5,430956589	8,6629074	0,0072742
2569	ENSG00000116679	0,109368134	6,809890255	0,3100543	0,582999029
2570	ENSG00000116685	0,021452243	4,782562051	0,0242279	0,974952205
2571	ENSG00000116688	0,208471996	8,393869945	0,7705057	0,389094295
2572	ENSG00000116698	-0,008669079	5,661077343	0,0029727	0,95698744
2573	ENSG00000116701	-0,331423332	4,30618045	1,1081329	0,303369642
2574	ENSG00000116704	0,294546523	4,227370541	4,5254489	0,046801124
2575	ENSG00000116717	0,537188084	4,123251831	7,4173638	0,012083657
2576	ENSG00000116729	-0,067599682	5,910871182	0,3334303	0,569209648
2577	ENSG00000116731	2,58028E-05	5,551532862	3,43E-08	0,999853812
2578	ENSG00000116741	0,626539135	5,751107258	2,6763802	0,115393526
2579	ENSG00000116747	0,087619885	6,715823295	0,2118291	0,649638252
2580	ENSG00000116750	-0,35187206	5,413733139	4,3486387	0,048270235
2581	ENSG00000116752	-0,308677	4,873812899	1,9908357	0,171572598
2582	ENSG00000116754	-0,123107177	6,971384779	0,6975566	0,412140543
2583	ENSG00000116774	0,445204371	4,814366809	2,3516884	0,138722896
2584	ENSG00000116786	0,036909893	5,572355799	0,0487354	0,82721704
2585	ENSG00000116791	-0,337397597	5,335411001	2,8448136	0,105122256
2586	ENSG00000116793	0,201970943	3,9319903	2,0569142	0,221380125
2587	ENSG00000116815	-0,246873839	4,175913055	1,8326632	0,188904223
2588	ENSG00000116830	0,128771777	4,082060969	0,6337911	0,457691768
2589	ENSG00000116857	0,381725438	4,636475186	5,8906934	0,02341867
2590	ENSG00000116871	-0,217259864	6,825349855	1,3373812	0,259310094
2591	ENSG00000116874	0,023799736	4,018876798	0,0374171	0,848302278
2592	ENSG00000116898	-0,465349531	5,44093184	12,051929	0,00204798
2593	ENSG00000116903	-0,098147564	4,790277672	0,6511495	0,444969293
2594	ENSG00000116906	-0,315763652	6,076550655	4,2745432	0,050061333

2595	ENSG00000116918	-0,252827426	5,105891396	2,0841099	0,162257586
2596	ENSG00000116954	0,074274251	4,009205332	0,2793569	0,609321269
2597	ENSG00000116962	-0,961168792	6,44206926	11,448171	0,002546392
2598	ENSG00000116977	0,280982801	6,391785077	2,4102839	0,134127193
2599	ENSG00000116984	0,115475891	6,881550221	0,7840631	0,384989718
2600	ENSG00000116991	-0,198495066	6,846753954	0,8886269	0,355599528
2601	ENSG00000117000	0,030524772	5,780087604	0,0298002	0,864447016
2602	ENSG00000117020	0,194611882	6,366926644	1,8968273	0,18158784
2603	ENSG00000117036	0,394725662	4,919644337	7,937272	0,009730579
2604	ENSG00000117054	-0,336601645	7,619435164	1,543965	0,226487954
2605	ENSG00000117114	-0,203715403	7,000538869	1,4817618	0,235782177
2606	ENSG00000117118	-0,645749229	7,063469737	9,9780506	0,004372154
2607	ENSG00000117133	-0,303657694	4,942986859	2,472465	0,129446358
2608	ENSG00000117139	0,301054461	5,526178251	3,5679175	0,071518423
2609	ENSG00000117143	-0,845560604	6,297827837	8,1039215	0,009105117
2610	ENSG00000117151	0,023501771	3,629558029	0,0176916	0,895337042
2611	ENSG00000117153	0,037956939	5,095252544	0,1324679	0,729790041
2612	ENSG00000117155	0,056385543	5,248087746	0,0566831	0,813915394
2613	ENSG00000117174	0,116235475	4,479793993	1,170323	0,374628692
2614	ENSG00000117222	-0,049240526	4,435129401	0,2686124	0,748044392
2615	ENSG00000117226	0,730106685	4,509836819	4,987399	0,035499491
2616	ENSG00000117228	0,328394176	4,879506481	1,2174919	0,281209632
2617	ENSG00000117262	-0,270214094	4,86670386	4,2209921	0,051361713
2618	ENSG00000117266	-0,176376509	6,038159974	0,4694753	0,500043362
2619	ENSG00000117280	0,106601602	4,364724951	0,5882584	0,487695846
2620	ENSG00000117298	0,226839889	6,293064594	1,0166366	0,323757991
2621	ENSG00000117305	0,124623322	4,558935533	0,8375447	0,372219795
2622	ENSG00000117318	0,494393739	5,532775284	8,4127161	0,00803803
2623	ENSG00000117335	-0,129731086	7,268955035	0,5329035	0,472723371
2624	ENSG00000117360	0,087360765	5,064511457	0,711074	0,443048978
2625	ENSG00000117362	0,202908638	5,580916321	2,5028728	0,127180681
2626	ENSG00000117394	-0,436659948	3,693784015	3,7747475	0,064310465
2627	ENSG00000117395	-0,143745269	4,434172459	1,099784	0,330312964
2628	ENSG00000117408	-0,205832908	4,753527273	1,2840689	0,268762016
2629	ENSG00000117410	-0,077940649	5,049853492	0,3236931	0,574865121
2630	ENSG00000117411	0,003507867	4,420965517	0,000428	0,983672432
2631	ENSG00000117419	-0,004032104	4,726516909	0,0012657	0,971924861
2632	ENSG00000117448	0,061445471	5,166193145	0,1216266	0,730433875
2633	ENSG00000117450	-0,788265563	7,502893281	12,734382	0,001621915
2634	ENSG00000117461	-0,299707505	5,813191324	2,2832528	0,144329727
2635	ENSG00000117475	-0,265913005	5,240285933	0,9729414	0,334161697
2636	ENSG00000117479	-0,341648258	5,355907558	1,5414044	0,226861303
2637	ENSG00000117481	-0,128474584	4,08933152	0,8095811	0,379369834
2638	ENSG00000117500	0,172183558	6,444411231	0,6594007	0,425056377
2639	ENSG00000117505	0,089665787	5,789017489	0,613533	0,441385981
2640	ENSG00000117519	0,069871353	6,826477784	0,1136067	0,739116251
2641	ENSG00000117523	0,090056612	7,268705684	0,3421128	0,564268225
2642	ENSG00000117525	0,488945926	5,19932966	5,9838886	0,022459894
2643	ENSG00000117528	-0,329980713	6,478608019	1,9985826	0,170774673
2644	ENSG00000117533	-0,039724733	4,580448692	0,0655067	0,800259182
2645	ENSG00000117543	-0,151429213	4,382694117	1,2699159	0,360203469
2646	ENSG00000117569	-0,450200193	5,344242818	7,1895598	0,013298357
2647	ENSG00000117592	-0,558830924	8,011972103	10,649535	0,003395523
2648	ENSG00000117593	-0,005034232	4,571757186	0,0024285	0,961118504
2649	ENSG00000117595	0,403522751	4,514464063	3,8028839	0,063395494
2650	ENSG00000117597	0,216339891	4,194542387	2,9921945	0,153836243
2651	ENSG00000117614	0,04171856	5,362552621	0,056533	0,814157179
2652	ENSG00000117616	0,451495673	5,907552623	5,3289102	0,030260691
2653	ENSG00000117620	0,02041175	4,732299279	0,021018	0,885984163

2654	ENSG00000117625	-0,072854972	5,919223102	0,2072097	0,653210512
2655	ENSG00000117632	0,418270245	4,223587	5,3254181	0,030309674
2656	ENSG00000117640	-0,210009895	6,116393791	1,4976103	0,233368877
2657	ENSG00000117643	0,468228406	3,885992571	4,0768973	0,055218288
2658	ENSG00000117682	0,135608408	4,626559529	0,7404112	0,398367438
2659	ENSG00000117691	-0,046729467	5,56133347	0,0767099	0,784268548
2660	ENSG00000117697	-0,16566292	5,148945209	1,8511792	0,190485792
2661	ENSG00000117707	-0,162803433	6,453392746	0,2668896	0,610334021
2662	ENSG00000117713	-0,022055729	5,493520684	0,0069228	0,934406025
2663	ENSG00000117748	0,15303736	4,798261163	1,49525	0,239907085
2664	ENSG00000117751	-0,015535503	4,644120436	0,0161808	0,953703544
2665	ENSG00000117758	-0,124912573	5,414381902	0,7550634	0,393796346
2666	ENSG00000117791	-0,382674159	4,71372415	13,574428	0,003559451
2667	ENSG00000117859	-0,092050226	6,682478172	0,6303262	0,435283715
2668	ENSG00000117868	-0,026105148	7,295236509	0,0434284	0,836739902
2669	ENSG00000117899	-0,125307546	5,178747271	1,2627235	0,274712687
2670	ENSG00000117906	-0,021232613	5,394951612	0,0191938	0,89101227
2671	ENSG00000117984	0,094561977	6,382588695	0,1397516	0,711935467
2672	ENSG00000118007	0,084109841	6,046003797	0,4107197	0,527886461
2673	ENSG00000118046	0,017217559	4,846919418	0,0147389	0,914303837
2674	ENSG00000118058	0,335494789	6,537150891	2,9926752	0,096975599
2675	ENSG00000118096	0,115906267	4,319347558	0,9196388	0,471196549
2676	ENSG00000118181	-0,01690293	8,45756883	0,0052774	0,942713143
2677	ENSG00000118194	-0,195382971	8,304066277	0,3566648	0,556170384
2678	ENSG00000118197	0,069233424	4,156113303	0,2979197	0,59769198
2679	ENSG00000118200	-0,076902027	6,536539643	0,2040968	0,655645101
2680	ENSG00000118217	-0,055531944	6,096666651	0,2927555	0,593619352
2681	ENSG00000118246	-0,343108911	6,325289984	2,8714833	0,103595684
2682	ENSG00000118257	0,509178039	4,559322343	4,3387396	0,048505281
2683	ENSG00000118260	-0,000624807	6,112414625	2,447E-05	0,99609553
2684	ENSG00000118263	-0,057877527	5,104517686	0,0830756	0,775740807
2685	ENSG00000118276	-0,540125453	3,571768314	9,2925956	0,006921433
2686	ENSG00000118363	-0,089815517	5,353158888	0,4581929	0,50516535
2687	ENSG00000118407	-0,151740336	7,328950985	0,2359714	0,631704444
2688	ENSG00000118412	-0,185024513	5,485683909	1,2517354	0,274714705
2689	ENSG00000118418	-0,447707101	6,354082253	4,8119916	0,038580229
2690	ENSG00000118454	0,099826361	5,024250995	0,4012708	0,532652579
2691	ENSG00000118473	0,355202979	4,013079325	4,1329614	0,053696795
2692	ENSG00000118482	0,07275958	7,287842386	0,2111235	0,650177766
2693	ENSG00000118495	0,23530559	4,746569674	1,4665108	0,238134584
2694	ENSG00000118496	-0,036004652	5,807865565	0,0533162	0,819421882
2695	ENSG00000118503	1,112021062	4,669013005	5,9754935	0,022544446
2696	ENSG00000118507	0,056272061	3,940445336	0,1243825	0,727525024
2697	ENSG00000118515	0,644331795	6,395482116	2,2505872	0,147101098
2698	ENSG00000118518	-0,074268392	5,92631094	0,2229638	0,641218677
2699	ENSG00000118523	1,134098069	7,316338266	5,5999862	0,026714914
2700	ENSG00000118564	-0,16903955	7,206399252	0,8360527	0,369968984
2701	ENSG00000118579	0,096995091	5,181753746	0,4378803	0,514688931
2702	ENSG00000118596	-0,119877219	8,447885453	0,086946	0,770728532
2703	ENSG00000118620	0,093270723	3,661378752	0,316209	0,605028215
2704	ENSG00000118680	0,100636056	7,355320108	0,2723939	0,606696545
2705	ENSG00000118689	0,12853152	6,427204763	0,5463757	0,467243538
2706	ENSG00000118690	0,018166941	3,681466243	0,01174	0,940450404
2707	ENSG00000118705	-0,188001871	6,975635319	2,2813602	0,144441639
2708	ENSG00000118729	-0,392668894	10,04924254	1,0967436	0,305810619
2709	ENSG00000118762	0,423905033	5,753005944	3,5032629	0,07395795
2710	ENSG00000118785	0,954088593	4,471397105	4,5351135	0,044074112
2711	ENSG00000118816	0,133796329	8,115111609	0,7953686	0,381640922
2712	ENSG00000118849	-0,260336493	4,985876269	0,247178	0,623765634

2713	ENSG00000118855	-0,107471519	5,734889371	0,3897319	0,538554279
2714	ENSG00000118873	0,003029361	6,007004117	0,0009519	0,9756502
2715	ENSG00000118898	-0,563092559	4,312859823	2,365648	0,137611278
2716	ENSG00000118900	-0,008242536	5,103001813	0,0017963	0,966557443
2717	ENSG00000118922	0,006931326	5,868306504	0,0029473	0,957169713
2718	ENSG00000118946	-0,258474515	3,816334089	1,4971828	0,233433555
2719	ENSG00000118960	0,182230956	4,546871851	0,8651376	0,361918641
2720	ENSG00000118961	0,002536602	3,867786217	0,0002851	0,986673543
2721	ENSG00000118965	0,248286401	4,763765073	2,1840151	0,152948823
2722	ENSG00000118971	0,270942094	8,24947848	1,8377661	0,188312503
2723	ENSG00000118985	-0,19699099	6,426326566	0,6895653	0,41480793
2724	ENSG00000119004	0,216627994	4,177581607	2,7690417	0,14436482
2725	ENSG00000119013	-0,898454576	6,884161158	9,1021545	0,006119159
2726	ENSG00000119041	-0,23792037	5,471101854	1,7250795	0,201930311
2727	ENSG00000119048	-0,156573243	6,27799531	0,8113479	0,377012312
2728	ENSG00000119138	0,108897141	6,658358117	0,8090495	0,377645399
2729	ENSG00000119185	0,155795793	6,067461218	0,3099584	0,583056904
2730	ENSG00000119203	-0,14676695	4,498064776	1,2200795	0,374743825
2731	ENSG00000119231	0,018868753	5,68857542	0,0359704	0,861439856
2732	ENSG00000119242	-0,308025058	4,951764647	3,1111611	0,090973383
2733	ENSG00000119280	0,386269926	5,880125495	5,0009323	0,035273516
2734	ENSG00000119285	-0,017215525	5,305682006	0,0120565	0,913514021
2735	ENSG00000119314	0,173816857	6,044500489	0,6297331	0,435524914
2736	ENSG00000119318	0,056577282	6,784071633	0,2449825	0,625286755
2737	ENSG00000119321	-0,153225512	5,370004905	2,5378728	0,124682469
2738	ENSG00000119326	-0,223067014	6,933080504	1,7698208	0,196382425
2739	ENSG00000119328	0,088094809	3,727585956	0,4183902	0,613674669
2740	ENSG00000119335	0,032636972	7,526674857	0,0610048	0,807087953
2741	ENSG00000119383	-0,195471689	6,148149377	1,1446961	0,295708687
2742	ENSG00000119392	0,074735698	4,573716947	0,4170374	0,590916667
2743	ENSG00000119396	0,150303998	6,181069307	1,5794841	0,221344973
2744	ENSG00000119397	0,315805619	5,052564027	2,8116339	0,107058218
2745	ENSG00000119401	-0,038463976	4,122161444	0,0714466	0,791609415
2746	ENSG00000119402	0,021287151	6,119463239	0,031471	0,860733852
2747	ENSG00000119403	0,119918988	3,868803657	0,3451147	0,562587519
2748	ENSG00000119408	0,389313112	3,994867478	2,9652207	0,098431145
2749	ENSG00000119414	0,058170443	5,779694981	0,2496562	0,622024263
2750	ENSG00000119421	-0,5142386	6,474004493	6,3667156	0,018954488
2751	ENSG00000119446	-0,252866216	5,454686469	1,4139986	0,246467294
2752	ENSG00000119471	0,056189392	7,074474993	0,0732325	0,789089059
2753	ENSG00000119487	-0,130830302	5,922088341	1,8142338	0,191015245
2754	ENSG00000119508	1,020842324	6,472870918	1,1161392	0,301669472
2755	ENSG00000119509	0,119551988	4,501382471	0,5642462	0,460134814
2756	ENSG00000119522	0,205267478	4,227410241	1,3478141	0,257511222
2757	ENSG00000119523	0,077601064	4,321444435	0,388348	0,573946121
2758	ENSG00000119537	0,102595743	5,610680338	0,8271683	0,372447243
2759	ENSG00000119541	0,156620062	5,200489014	1,3656345	0,254434655
2760	ENSG00000119596	0,129365455	6,017095095	0,7096744	0,408184982
2761	ENSG00000119616	0,025346077	4,578213717	0,0516559	0,822197953
2762	ENSG00000119638	0,139031607	6,712510928	0,9352503	0,343489184
2763	ENSG00000119650	0,262149853	4,147324792	2,9901055	0,115819777
2764	ENSG00000119655	0,116733565	5,974215654	0,456501	0,505968214
2765	ENSG00000119661	0,365537146	4,384083466	8,7852294	0,008231272
2766	ENSG00000119669	-0,059783759	4,556097734	0,1118391	0,741075669
2767	ENSG00000119673	-0,122080809	5,488649391	0,6101211	0,442669831
2768	ENSG00000119681	0,912506335	6,632947797	10,659125	0,003391608
2769	ENSG00000119682	-0,162311398	5,13340979	1,2931403	0,267106009
2770	ENSG00000119684	-0,242257736	5,631248361	4,4360144	0,046210818
2771	ENSG00000119685	0,01780197	4,469710574	0,0208471	0,962617413

2772	ENSG00000119688	-0,059256413	4,450278256	0,0904429	0,766303446
2773	ENSG00000119689	-0,215673177	6,738660275	1,8877907	0,182630575
2774	ENSG00000119699	0,212676545	4,999230014	1,4702831	0,237549943
2775	ENSG00000119705	-0,659177305	5,55161834	7,5988954	0,0112033
2776	ENSG00000119707	-0,078267371	6,552092992	0,3860355	0,540449692
2777	ENSG00000119711	0,3933014	5,997079667	4,375323	0,047643018
2778	ENSG00000119718	-0,246530922	4,018827792	7,7488085	0,136576303
2779	ENSG00000119720	-0,158314957	4,37730228	0,9664287	0,335751966
2780	ENSG00000119723	-0,299104383	3,642133075	3,552053	0,114161991
2781	ENSG00000119729	-0,143529686	7,304443209	0,808468	0,377843176
2782	ENSG00000119760	0,032775455	5,324946615	0,0840202	0,85458594
2783	ENSG00000119772	0,276269505	4,565417211	2,6195567	0,119120739
2784	ENSG00000119777	0,0517266	4,951264473	0,0988264	0,756062671
2785	ENSG00000119778	-0,031086209	5,533353634	0,0534751	0,81915095
2786	ENSG00000119787	0,075648434	5,581052611	0,3675583	0,550226386
2787	ENSG00000119801	0,121739391	6,010455276	0,5783878	0,45463393
2788	ENSG00000119812	-0,087588337	5,568212167	0,3670122	0,550532641
2789	ENSG00000119820	0,038024242	6,184738557	0,1197462	0,732429201
2790	ENSG00000119844	0,091218568	5,195099754	0,4410873	0,513151147
2791	ENSG00000119862	0,186819948	4,622900607	2,0495761	0,1707808
2792	ENSG00000119865	0,380556674	4,135954031	3,5659166	0,071592526
2793	ENSG00000119878	-0,239668157	4,705357626	2,9085847	0,103713989
2794	ENSG00000119899	0,296462984	4,447144097	3,980615	0,057929563
2795	ENSG00000119900	0,305530724	6,184074008	1,3067816	0,264681207
2796	ENSG00000119906	0,139964876	6,091896098	0,8111502	0,37706945
2797	ENSG00000119912	-0,280678301	6,221333561	2,3451715	0,139245505
2798	ENSG00000119917	0,315089263	4,699416948	1,5872003	0,220299685
2799	ENSG00000119922	0,648075643	4,250811834	5,4643845	0,028426973
2800	ENSG00000119927	0,099636718	8,405663456	0,0127675	0,911011367
2801	ENSG00000119929	-0,407094119	4,467565692	6,7811866	0,01582882
2802	ENSG00000119938	-0,646948238	7,802916241	5,3546016	0,029903023
2803	ENSG00000119950	0,017027264	5,945303879	0,0174237	0,896123715
2804	ENSG00000119953	0,003060116	4,810646947	0,0007526	0,978348659
2805	ENSG00000119977	0,308634257	4,543785602	6,6127318	0,027032494
2806	ENSG00000119979	0,153353674	4,549365103	1,5257613	0,229116197
2807	ENSG00000120008	-0,035660525	5,663594512	0,0680807	0,79645628
2808	ENSG00000120029	0,283259518	5,816831512	3,3192571	0,081440075
2809	ENSG00000120049	-1,059435252	7,450480757	12,838923	0,001564827
2810	ENSG00000120053	-0,548707079	8,247616378	4,0040967	0,057267468
2811	ENSG00000120063	-0,023252513	6,077309929	0,0207168	0,886800769
2812	ENSG00000120071	-0,278057949	6,157350587	1,9600949	0,174783889
2813	ENSG00000120129	1,233433934	6,504073058	8,771526	0,006967776
2814	ENSG00000120137	0,026023908	6,707127625	0,0237052	0,878973849
2815	ENSG00000120156	-0,514059873	5,628552888	13,07437	0,00144167
2816	ENSG00000120159	0,124419703	3,961686651	0,6546094	0,445827867
2817	ENSG00000120217	0,088751637	3,847422265	0,2535945	0,620730249
2818	ENSG00000120253	-0,178891559	4,981457508	2,6629474	0,144499709
2819	ENSG00000120265	-0,536447115	6,425092941	6,26691	0,019805754
2820	ENSG00000120278	-0,56225778	4,640137119	5,6752329	0,025814713
2821	ENSG00000120279	-0,292855646	4,766826312	3,2235539	0,085673629
2822	ENSG00000120306	0,335837684	5,193291903	2,7588362	0,110225344
2823	ENSG00000120314	-0,013766661	3,933874512	0,0076402	0,93110003
2824	ENSG00000120333	-0,157605608	4,709908517	1,190796	0,286392767
2825	ENSG00000120370	0,035576606	3,757305454	0,0914417	0,824896464
2826	ENSG00000120438	-0,282860538	6,848502698	5,4881175	0,028085645
2827	ENSG00000120451	0,101181907	6,203031267	0,5025271	0,485463132
2828	ENSG00000120457	-0,674542375	3,952209645	3,3736125	0,079142976
2829	ENSG00000120509	-0,212601529	4,279379649	3,0437274	0,181333736
2830	ENSG00000120519	0,04954361	3,483928966	0,110505	0,785155882

2831	ENSG00000120526	-0,138954373	4,670317262	0,3683272	0,549833406
2832	ENSG00000120533	-0,308803652	5,248025615	3,4750427	0,075052522
2833	ENSG00000120549	-0,49461076	5,490946289	4,3105617	0,049181452
2834	ENSG00000120594	1,062541529	7,087659427	10,394136	0,003741053
2835	ENSG00000120616	-0,079492232	5,541154976	0,6064985	0,443982609
2836	ENSG00000120656	-0,185871011	3,928704309	2,0946022	0,318544776
2837	ENSG00000120662	-0,245854542	3,658261244	2,2028957	0,209368832
2838	ENSG00000120675	-0,648020353	5,866710321	6,8017582	0,015689305
2839	ENSG00000120685	-0,005447614	4,163366708	0,0020722	0,964081692
2840	ENSG00000120686	0,125198529	5,891553546	0,3331553	0,569387212
2841	ENSG00000120688	-0,386995394	4,939368268	6,8807015	0,015166419
2842	ENSG00000120690	0,116548668	5,548237277	0,5185771	0,478669162
2843	ENSG00000120693	0,949336428	4,248267484	8,1038403	0,009105417
2844	ENSG00000120694	0,131813102	6,210169295	0,2475601	0,623499006
2845	ENSG00000120696	0,212220249	4,009531979	2,1256794	0,187956862
2846	ENSG00000120697	-0,091517188	3,994483167	0,3425653	0,655433687
2847	ENSG00000120699	-0,259849345	4,38109641	2,6236395	0,118848271
2848	ENSG00000120705	-0,096605586	6,844829129	0,6411615	0,431416742
2849	ENSG00000120708	-0,586571666	6,467125043	8,2230499	0,008675989
2850	ENSG00000120709	0,12272377	4,64296258	0,9744237	0,438061735
2851	ENSG00000120725	-0,089966326	4,417612581	0,2901119	0,595298937
2852	ENSG00000120727	-0,058228694	5,630841285	0,2290657	0,636698332
2853	ENSG00000120729	-0,124004347	4,752566287	0,0616928	0,806033694
2854	ENSG00000120733	0,389243037	6,410370487	4,5939792	0,04283611
2855	ENSG00000120738	0,872879304	6,60219873	2,7833995	0,108738463
2856	ENSG00000120742	0,521045734	6,093709529	2,9852134	0,097368708
2857	ENSG00000120784	0,025261705	4,104118461	0,0328637	0,876169611
2858	ENSG00000120798	-0,106370446	5,193406513	0,4986199	0,487164637
2859	ENSG00000120800	0,015164765	5,281986005	0,0248127	0,876197923
2860	ENSG00000120802	0,185103729	5,226349137	0,9546389	0,338657776
2861	ENSG00000120805	-0,125108185	6,599254542	0,3768337	0,545297838
2862	ENSG00000120820	0,666481738	4,146651533	7,0516342	0,014099099
2863	ENSG00000120832	-0,120149577	4,404337749	0,9307169	0,428981703
2864	ENSG00000120833	-0,034858235	4,421254003	0,0397719	0,843671302
2865	ENSG00000120837	0,029181538	5,419807202	0,0285666	0,867254065
2866	ENSG00000120860	-0,326564909	4,851822099	4,8985355	0,037022649
2867	ENSG00000120868	0,480231489	4,165750197	6,1005012	0,021321177
2868	ENSG00000120885	-0,221579104	7,732353083	0,4166992	0,524945913
2869	ENSG00000120889	0,284493465	4,619543194	1,7152423	0,203176068
2870	ENSG00000120899	0,232558725	4,050863581	0,7005789	0,411160452
2871	ENSG00000120910	-0,146855301	4,984047797	1,8716432	0,19330995
2872	ENSG00000120915	0,113621232	4,736177454	0,3994946	0,533553191
2873	ENSG00000120925	0,085079137	4,667140281	0,5654076	0,459652686
2874	ENSG00000120937	0,94317667	9,467380194	5,5258252	0,027636521
2875	ENSG00000120948	-0,050982656	5,632248133	0,1800201	0,675265578
2876	ENSG00000120963	-0,053017856	5,219583711	0,1834284	0,690572727
2877	ENSG00000120992	-0,351071589	5,686583835	2,2330682	0,148613624
2878	ENSG00000121022	-0,531061397	6,210978442	9,2302971	0,005821393
2879	ENSG00000121039	-0,085012246	4,949578487	0,0371065	0,84893093
2880	ENSG00000121057	-0,125827406	6,792137722	0,3597106	0,55450187
2881	ENSG00000121058	-0,12591136	3,793910714	0,9402839	0,515096452
2882	ENSG00000121060	0,043853252	4,910628756	0,0577241	0,812248076
2883	ENSG00000121064	-0,062641792	5,669595953	0,1434218	0,708359165
2884	ENSG00000121067	0,056377516	5,651238683	0,3463524	0,613658314
2885	ENSG00000121073	-0,360179443	5,333454289	7,3013246	0,012663585
2886	ENSG00000121210	0,186498409	4,973713999	1,9284639	0,178119129
2887	ENSG00000121274	0,193619477	4,102972375	1,5872219	0,220296652
2888	ENSG00000121289	0,291575196	3,740388002	4,0417156	0,136243631
2889	ENSG00000121310	0,100924161	6,238677299	0,3538308	0,557731708

2890	ENSG00000121316	0,154752582	4,96840501	1,0164662	0,323797675
2891	ENSG00000121350	-0,157983694	4,466448772	0,8716446	0,360152126
2892	ENSG00000121361	-0,056227985	4,573289984	0,1645656	0,688706528
2893	ENSG00000121390	-0,179904478	5,140243005	3,2871797	0,082786073
2894	ENSG00000121406	0,086833577	3,716221323	0,4114884	0,676256366
2895	ENSG00000121413	0,284869952	4,512813772	2,7944338	0,108078164
2896	ENSG00000121417	0,056185597	4,322039388	0,1561168	0,732297147
2897	ENSG00000121440	-0,316087272	5,429095537	2,6013074	0,120347645
2898	ENSG00000121481	-0,018675785	4,159657505	0,0119017	0,914068671
2899	ENSG00000121486	-0,018227791	4,983560764	0,020029	0,990192425
2900	ENSG00000121542	-0,075669546	3,886633441	0,3346241	0,722527736
2901	ENSG00000121577	-0,19514329	7,871432673	0,7825351	0,385477156
2902	ENSG00000121578	0,255513582	4,375029635	2,334108	0,14013809
2903	ENSG00000121579	-0,074250066	6,261174058	0,2420919	0,627338736
2904	ENSG00000121644	0,041073003	5,493087057	0,0691526	0,79490458
2905	ENSG00000121671	0,176814592	6,393527283	0,7755824	0,387561672
2906	ENSG00000121691	0,315696257	6,666552759	1,9552201	0,175299828
2907	ENSG00000121741	-0,109508921	7,194495379	0,5581888	0,462504452
2908	ENSG00000121743	-0,385253651	3,673697747	1,700054	0,205118274
2909	ENSG00000121749	-0,034292323	5,613504472	0,0468799	0,830483852
2910	ENSG00000121766	-0,09916889	4,815535899	0,3602461	0,554209519
2911	ENSG00000121769	-0,307044289	10,0522566	1,0664837	0,312426648
2912	ENSG00000121774	0,105191399	6,159471043	0,9799177	0,332434396
2913	ENSG00000121851	-0,105769982	4,512632303	0,4548621	0,551305288
2914	ENSG00000121858	-0,116405307	5,257490656	0,2024818	0,656917089
2915	ENSG00000121864	-0,152746022	5,034687369	1,4442111	0,241586353
2916	ENSG00000121879	-0,014112807	6,139479632	0,0083751	0,927871696
2917	ENSG00000121892	-0,015008411	6,736820137	0,0107284	0,918395622
2918	ENSG00000121897	-0,24858827	4,2244687	2,1052055	0,160236378
2919	ENSG00000121898	0,658529895	4,494021931	5,6466165	0,026153012
2920	ENSG00000121931	-0,26939838	4,075969202	2,2676862	0,145642558
2921	ENSG00000121940	0,065466994	4,810100309	0,5150225	0,592489079
2922	ENSG00000121957	-0,791927633	4,321052524	12,546537	0,001730304
2923	ENSG00000121964	0,011542351	5,582287236	0,0116483	0,91498163
2924	ENSG00000121988	-0,05531667	3,479243227	0,1034035	0,79484405
2925	ENSG00000121989	0,090345695	4,733892163	0,4441586	0,511697527
2926	ENSG00000122008	-0,129950007	5,626990434	0,5461508	0,467334127
2927	ENSG00000122026	-0,022642222	8,043597851	0,0118735	0,914170204
2928	ENSG00000122033	-0,774091659	5,734744899	24,145435	5,7032E-05
2929	ENSG00000122034	-0,605088748	6,512661395	26,565744	3,10747E-05
2930	ENSG00000122042	0,085647463	5,769543397	0,3671802	0,550435016
2931	ENSG00000122068	-0,062190689	6,587638853	0,1017414	0,752614564
2932	ENSG00000122085	-0,094283965	4,197877047	0,7693994	0,516366199
2933	ENSG00000122126	-0,2806288	4,94439678	5,0532627	0,034379
2934	ENSG00000122176	1,489766592	6,243191547	23,815964	6,20154E-05
2935	ENSG00000122203	-0,001876735	5,937176864	0,0003144	0,986005302
2936	ENSG00000122218	0,283119987	7,429942702	8,5703327	0,007529996
2937	ENSG00000122257	0,095020673	5,867070837	0,5082232	0,483020322
2938	ENSG00000122299	0,076304621	5,573337807	0,4417379	0,512839291
2939	ENSG00000122335	0,090714448	3,645003038	0,3608805	0,615308901
2940	ENSG00000122359	-0,187531421	7,642241803	0,8848759	0,356597985
2941	ENSG00000122367	-0,283405195	9,530638985	0,7176149	0,405613983
2942	ENSG00000122376	-0,283276113	5,329296581	1,9991083	0,170720681
2943	ENSG00000122378	-0,468660728	6,514030846	5,7559869	0,024886068
2944	ENSG00000122406	0,178971049	8,910800019	0,6288849	0,435830149
2945	ENSG00000122417	0,179620068	4,952161114	0,85899	0,363599026
2946	ENSG00000122420	-1,103623532	4,324797221	11,428589	0,002564327
2947	ENSG00000122435	-0,07918792	4,418092419	0,3035104	0,636587467
2948	ENSG00000122477	-0,642914686	6,581178301	2,7814719	0,108854293

2949	ENSG00000122482	-0,235365406	6,472620762	1,2562563	0,27387207
2950	ENSG00000122483	0,182179503	3,764472564	1,3000126	0,294005032
2951	ENSG00000122484	0,028708328	5,39287038	0,0843997	0,774003022
2952	ENSG00000122507	0,231026424	4,921773472	3,8872829	0,060695799
2953	ENSG00000122512	0,100376761	4,027022516	1,0299632	0,568475389
2954	ENSG00000122545	-0,002896032	7,834999909	0,0001741	0,989584397
2955	ENSG00000122550	-0,487935617	6,711541459	3,8687498	0,061310965
2956	ENSG00000122557	0,26501704	4,588271321	5,7603622	0,04676339
2957	ENSG00000122565	-0,18115051	6,337549853	1,0151738	0,32409896
2958	ENSG00000122566	-0,253965925	8,382252066	4,4767543	0,045302037
2959	ENSG00000122591	-0,100918274	6,179359305	0,2653545	0,611357006
2960	ENSG00000122642	0,122173815	6,555456083	0,5534744	0,464398255
2961	ENSG00000122643	-0,116265983	4,412492499	0,4412703	0,513083153
2962	ENSG00000122644	-0,154459048	4,110664544	0,4833584	0,493836211
2963	ENSG00000122674	0,174597474	5,58732477	1,2210914	0,280517433
2964	ENSG00000122679	-0,246848437	4,563545373	0,9339583	0,343840585
2965	ENSG00000122692	-0,063001642	6,018604401	0,3770868	0,545143177
2966	ENSG00000122696	-0,059430348	3,595830427	0,2029257	0,749100737
2967	ENSG00000122705	0,056356945	5,236620687	0,1247015	0,727190666
2968	ENSG00000122707	0,222673645	4,438684284	1,6422947	0,212718804
2969	ENSG00000122729	-0,025169252	6,294435205	0,0200289	0,888682782
2970	ENSG00000122741	-0,060079054	5,270941214	0,1887689	0,667961147
2971	ENSG00000122779	-0,088302589	4,80359695	0,6686723	0,478670399
2972	ENSG00000122786	0,204402893	8,463636553	1,1420032	0,296240765
2973	ENSG00000122861	-1,174100971	4,466614747	10,445928	0,003669764
2974	ENSG00000122862	-0,366963816	6,372030833	1,0801484	0,309415166
2975	ENSG00000122863	0,234037411	5,027114778	1,4307069	0,243776059
2976	ENSG00000122870	0,412669683	5,710630834	2,0353524	0,167048246
2977	ENSG00000122873	-0,655303588	6,282198483	5,7351232	0,025122374
2978	ENSG00000122882	-0,097391004	4,733767714	0,7555461	0,489814033
2979	ENSG00000122884	-0,233748871	6,252895897	1,0934866	0,306513531
2980	ENSG00000122912	0,23553632	5,050554132	2,1276454	0,158119586
2981	ENSG00000122958	-0,000516815	5,498190109	1,086E-05	0,997398577
2982	ENSG00000122965	-0,097935869	4,330763694	0,3358006	0,567869186
2983	ENSG00000122970	0,062183544	4,337891994	0,2131504	0,648610168
2984	ENSG00000123066	0,008371034	7,038689181	0,0025293	0,960322004
2985	ENSG00000123080	0,114491125	3,845058823	0,3568752	0,556054807
2986	ENSG00000123091	-0,041774548	6,651659467	0,0750336	0,786576526
2987	ENSG00000123094	0,072622957	6,511168357	0,1196435	0,732550176
2988	ENSG00000123095	0,722370579	4,405991598	24,581225	5,0747E-05
2989	ENSG00000123096	0,075936024	6,619345784	0,1235723	0,72837637
2990	ENSG00000123104	0,094514674	6,87819258	0,2628377	0,613042338
2991	ENSG00000123106	-0,018093595	5,562118558	0,0136498	0,908002222
2992	ENSG00000123119	-0,302140647	5,091475144	1,2653876	0,27218041
2993	ENSG00000123124	-0,218014833	6,614634127	3,7336111	0,065631836
2994	ENSG00000123130	-0,136421159	6,029511656	1,1056918	0,303853036
2995	ENSG00000123131	0,210577461	5,307344607	1,5239877	0,22942144
2996	ENSG00000123143	-0,087333707	5,81670109	0,1556249	0,696833533
2997	ENSG00000123144	-0,079780369	5,238697377	0,3548488	0,557149793
2998	ENSG00000123146	-0,032206749	4,416994053	0,0193002	0,890712643
2999	ENSG00000123159	0,022617505	4,65878209	0,015734	0,90126357
3000	ENSG00000123178	-0,193944069	4,000142087	1,9485647	0,26460592
3001	ENSG00000123191	0,62511986	4,014164152	12,602762	0,001697053
3002	ENSG00000123200	0,182333711	6,225882902	4,2064315	0,051733317
3003	ENSG00000123213	-0,10036866	4,236825874	0,8858936	0,555961091
3004	ENSG00000123240	-0,076098492	7,035329108	0,4629336	0,50299199
3005	ENSG00000123243	0,446187106	5,059811594	3,1601574	0,088617787
3006	ENSG00000123268	0,21129665	4,199997194	1,8678979	0,184864531
3007	ENSG00000123338	-0,205806045	4,569982072	0,6567633	0,425971028

3008	ENSG00000123349	0,11051735	7,295704955	0,3627915	0,552823916
3009	ENSG00000123352	0,228611483	3,616953483	1,8563609	0,250543962
3010	ENSG00000123358	0,941408666	6,301310729	4,1300027	0,053775891
3011	ENSG00000123384	0,103138662	8,325230097	0,0763104	0,784816268
3012	ENSG00000123416	-0,144850473	6,156318198	0,4814449	0,494683781
3013	ENSG00000123472	-0,104677938	7,03469489	0,5097167	0,482394024
3014	ENSG00000123505	-0,378783461	6,633104653	5,5270246	0,027621336
3015	ENSG00000123545	-0,520344726	5,726423736	3,4273355	0,076945446
3016	ENSG00000123552	-0,160216891	5,039160968	0,9022446	0,352007949
3017	ENSG00000123560	-0,983033255	4,479372339	14,474763	0,000906794
3018	ENSG00000123562	-0,12486102	7,331369259	0,3239849	0,574713078
3019	ENSG00000123570	-0,215175067	5,407535121	0,4823569	0,494279513
3020	ENSG00000123575	-0,027490985	5,426712378	0,0476014	0,829199103
3021	ENSG00000123595	-0,016186881	4,147650583	0,0110417	0,962256014
3022	ENSG00000123600	0,174614378	4,003030027	1,2509165	0,279124428
3023	ENSG00000123607	-0,156631034	5,378433367	1,2665171	0,27194404
3024	ENSG00000123636	-0,036059819	6,81913043	0,0908029	0,765843911
3025	ENSG00000123684	0,060691755	6,421997018	0,1846107	0,671408192
3026	ENSG00000123689	0,356613944	5,950127834	0,5051964	0,484337052
3027	ENSG00000123728	-0,120401115	4,825098176	0,7989172	0,384786105
3028	ENSG00000123737	-0,177904977	4,144591686	2,4612935	0,283908735
3029	ENSG00000123739	0,094045885	5,260780491	0,505731	0,484083781
3030	ENSG00000123836	-0,184617291	5,97476085	0,2629977	0,612934942
3031	ENSG00000123908	0,011680469	6,037857099	0,0031057	0,95603649
3032	ENSG00000123933	0,490782882	4,732134569	5,2638147	0,031188534
3033	ENSG00000123983	-0,058846228	6,199859569	0,1863073	0,669993679
3034	ENSG00000123989	0,528345138	5,70678558	5,1054531	0,033581588
3035	ENSG00000123992	0,077869728	4,943457741	0,2730578	0,606259434
3036	ENSG00000124006	0,46294316	6,24591569	4,5342452	0,044092672
3037	ENSG00000124067	0,278641869	5,43117597	1,9103121	0,180141183
3038	ENSG00000124098	0,024148808	5,605990012	0,0474595	0,829449691
3039	ENSG00000124120	-0,319817856	4,054201772	1,9138483	0,179754089
3040	ENSG00000124126	-0,254169294	4,409212046	0,7910613	0,382943426
3041	ENSG00000124145	0,502769244	6,233320831	4,1924058	0,052135207
3042	ENSG00000124151	-0,117154405	5,986176987	0,8648159	0,36197306
3043	ENSG00000124155	0,03968317	6,325047739	0,0713406	0,79176021
3044	ENSG00000124160	-0,032498377	4,317368083	0,033019	0,857393442
3045	ENSG00000124164	-0,000643152	6,096899885	2,139E-05	0,996349793
3046	ENSG00000124172	-0,59246934	7,970147806	5,8396752	0,023962776
3047	ENSG00000124177	0,102734259	6,459776325	0,8866634	0,356087989
3048	ENSG00000124181	0,372466617	5,516130396	2,6506947	0,117061098
3049	ENSG00000124193	0,019340343	6,331029398	0,017558	0,895728026
3050	ENSG00000124198	-0,003103333	6,926405731	0,0005736	0,981096917
3051	ENSG00000124201	0,313923464	5,983900384	3,4629445	0,075527451
3052	ENSG00000124207	-0,297961223	6,125002772	2,1838493	0,152963733
3053	ENSG00000124209	0,24536191	5,279442965	1,922868	0,17877132
3054	ENSG00000124212	0,89115702	7,035859305	2,9976548	0,096714279
3055	ENSG00000124214	0,144528623	6,07483853	1,678134	0,207918025
3056	ENSG00000124222	0,209954655	5,158644025	1,9449165	0,176396448
3057	ENSG00000124225	-0,043997515	4,934155757	0,0275876	0,869526441
3058	ENSG00000124226	0,047457188	5,242716051	0,1606949	0,753762968
3059	ENSG00000124228	-0,018190982	4,379067607	0,0153709	0,902401235
3060	ENSG00000124275	-0,166278898	4,870393484	1,9956971	0,179038576
3061	ENSG00000124299	-0,21966046	4,433423731	1,6081954	0,217371445
3062	ENSG00000124333	0,029893105	4,937108458	0,027482	0,869774016
3063	ENSG00000124356	-0,144888245	4,883209843	1,9746624	0,300313863
3064	ENSG00000124357	0,031553807	4,756615897	0,0525186	0,895767976
3065	ENSG00000124370	-0,210029833	4,247525252	1,0174827	0,323560985
3066	ENSG00000124374	0,229187656	5,9493142	0,5808063	0,453703859

3067	ENSG00000124380	-0,187982372	4,369839043	1,9746423	0,238049582
3068	ENSG00000124383	0,025302244	4,727701	0,0616951	0,806022515
3069	ENSG00000124406	0,04538118	6,734507363	0,1190217	0,733206972
3070	ENSG00000124422	0,379742454	6,895320367	3,6090621	0,070013922
3071	ENSG00000124429	0,147493523	4,637296938	0,2324299	0,634261991
3072	ENSG00000124440	-0,033311154	5,2585394	0,0082761	0,928297652
3073	ENSG00000124459	0,023963868	3,918963842	0,0590251	0,891139359
3074	ENSG00000124486	0,04177066	8,075618971	0,1161368	0,736330406
3075	ENSG00000124491	-1,672558448	7,824733855	44,405814	8,26724E-07
3076	ENSG00000124523	-0,231850582	5,430280931	1,6464815	0,212156168
3077	ENSG00000124532	-0,226165114	5,579647544	1,7090912	0,203959882
3078	ENSG00000124535	-0,067292518	4,897214042	0,3467905	0,561627669
3079	ENSG00000124541	-0,283874989	4,313210055	4,8780705	0,067340965
3080	ENSG00000124562	-0,426566856	5,328479034	7,42808	0,012029617
3081	ENSG00000124570	-0,56265	7,093052908	14,641737	0,000855125
3082	ENSG00000124571	0,081250271	4,77878813	0,3996669	0,609906277
3083	ENSG00000124588	-0,377300014	5,089392271	4,8734406	0,037468068
3084	ENSG00000124596	-0,061618346	4,379890609	0,234438	0,777522031
3085	ENSG00000124614	0,061233509	4,039774086	0,0761334	0,785059372
3086	ENSG00000124615	-0,308176191	4,758348189	1,7491016	0,198927799
3087	ENSG00000124641	-0,341208054	3,790385799	13,697032	0,062825388
3088	ENSG00000124688	-0,291173253	4,037458834	4,1380756	0,0877439
3089	ENSG00000124701	-0,596378564	6,008473995	5,2833511	0,030906772
3090	ENSG00000124702	-0,033590347	5,934700852	0,0466231	0,83094132
3091	ENSG00000124733	0,035530096	4,101627712	0,0439917	0,835706781
3092	ENSG00000124743	-0,445874561	8,04879413	1,7617721	0,197366398
3093	ENSG00000124749	0,849367072	5,834346872	13,409908	0,001289381
3094	ENSG00000124762	0,072827113	5,906981148	0,0573424	0,81285759
3095	ENSG00000124766	0,056390978	4,180083721	0,0489436	0,826854635
3096	ENSG00000124767	-0,394687437	6,602974149	3,3642287	0,079534169
3097	ENSG00000124772	0,322563104	5,328101428	0,8745452	0,359368658
3098	ENSG00000124782	0,023076132	5,449848708	0,0169031	0,897681821
3099	ENSG00000124783	0,138239966	6,77714009	0,901968	0,352054455
3100	ENSG00000124784	0,006049909	4,051378836	0,001743	0,967056753
3101	ENSG00000124785	-0,173855404	3,784310345	0,9531088	0,339037451
3102	ENSG00000124786	0,112591285	4,074914413	0,5698581	0,45793896
3103	ENSG00000124788	0,288652434	6,209729576	3,3300889	0,080976262
3104	ENSG00000124789	0,074923096	5,745814927	0,3754216	0,546025068
3105	ENSG00000124795	-0,277440705	6,777140061	3,2373528	0,085047849
3106	ENSG00000124831	0,219912996	6,828156924	3,3745242	0,079059868
3107	ENSG00000124942	0,495655058	11,41561939	2,7641968	0,10988105
3108	ENSG00000125037	-0,546051961	6,040962695	8,9796239	0,006419615
3109	ENSG00000125107	0,087784347	7,009243026	0,8485339	0,36645011
3110	ENSG00000125124	0,058952252	5,806093932	0,2024421	0,656933929
3111	ENSG00000125148	-0,647028494	5,905961982	1,5664909	0,223236866
3112	ENSG00000125166	-0,279801476	7,666025136	1,4339593	0,243256586
3113	ENSG00000125249	0,042262749	5,467715061	0,0439463	0,83579043
3114	ENSG00000125257	0,02033306	3,96740237	0,0086315	0,926778997
3115	ENSG00000125266	0,587765727	3,979963358	4,2166079	0,051514201
3116	ENSG00000125304	-0,075065333	6,92330993	0,2764276	0,604046537
3117	ENSG00000125347	0,114236717	4,364049598	0,1971196	0,661184851
3118	ENSG00000125354	0,269279163	4,404857544	2,6198784	0,119099245
3119	ENSG00000125356	-0,811598671	7,802669909	10,476808	0,003627969
3120	ENSG00000125375	-0,118643915	3,869466874	0,9327628	0,516058557
3121	ENSG00000125386	-0,014010072	4,263619199	0,0053568	0,9422845
3122	ENSG00000125398	0,723916063	4,024529091	6,0619256	0,021690599
3123	ENSG00000125445	-0,466933298	5,841172074	6,5239859	0,017694308
3124	ENSG00000125447	-0,051683221	4,350489797	0,0810825	0,778371533
3125	ENSG00000125450	-0,177429302	4,475854366	1,6638247	0,20980118

3126	ENSG00000125459	0,028794078	4,140117164	0,0391424	0,930916821
3127	ENSG00000125482	-0,244709999	4,47657679	3,0148473	0,095780023
3128	ENSG00000125484	0,127105141	4,916645878	0,9192033	0,358139683
3129	ENSG00000125503	0,063075577	6,197868962	0,0698679	0,793871891
3130	ENSG00000125629	0,024098505	4,677844945	0,0232406	0,880155991
3131	ENSG00000125630	-0,040813681	4,165631939	0,1154147	0,820378293
3132	ENSG00000125633	0,069659398	5,888615169	0,1958735	0,662186622
3133	ENSG00000125648	0,098622897	5,335215022	0,1795982	0,6756368
3134	ENSG00000125651	0,126843157	4,610286623	0,5022601	0,485596075
3135	ENSG00000125676	-0,136211469	6,115282603	0,9213553	0,34702846
3136	ENSG00000125686	0,044128997	5,295313332	0,1555189	0,74728886
3137	ENSG00000125691	0,056216087	8,571270403	0,0597699	0,809017362
3138	ENSG00000125703	0,033699288	3,899149204	0,0350198	0,853187207
3139	ENSG00000125730	0,934202195	9,821660973	2,2597034	0,146321326
3140	ENSG00000125733	-0,171568342	5,358468187	1,102125	0,30465396
3141	ENSG00000125734	0,284906028	4,028171284	2,8511764	0,111213897
3142	ENSG00000125741	-0,261384938	4,830909005	1,4653804	0,238310131
3143	ENSG00000125743	-0,173679669	5,768933768	0,7401981	0,398434286
3144	ENSG00000125753	-0,390184156	4,861837759	4,0888448	0,054889999
3145	ENSG00000125755	0,097819806	4,722462851	0,2407802	0,628269625
3146	ENSG00000125772	-0,015050858	6,162763884	0,0080471	0,929293877
3147	ENSG00000125779	-0,034288175	4,353738191	0,0720423	0,834937439
3148	ENSG00000125810	-0,089719581	6,650502844	0,3209968	0,576470199
3149	ENSG00000125812	0,041919754	4,840447748	0,0914421	0,765049411
3150	ENSG00000125814	0,021027243	4,21309436	0,0259511	0,928685513
3151	ENSG00000125817	0,079730525	5,15422173	0,1827383	0,672986829
3152	ENSG00000125818	-0,11744319	5,755451136	1,225881	0,279560527
3153	ENSG00000125826	0,146907035	4,655785074	0,8162417	0,375601681
3154	ENSG00000125827	-0,375353463	7,745545939	6,3562755	0,01901378
3155	ENSG00000125834	0,12260511	4,494191819	0,3295735	0,571455588
3156	ENSG00000125835	-0,01940115	4,168757841	0,0131635	0,909648221
3157	ENSG00000125844	0,06434797	6,228911111	0,0678965	0,796731908
3158	ENSG00000125846	0,103394966	3,928836596	0,633821	0,565212618
3159	ENSG00000125863	-0,36813242	4,836167244	9,6816719	0,004879576
3160	ENSG00000125868	-0,225830666	8,643740232	2,1398031	0,156940197
3161	ENSG00000125870	-0,223811888	4,926987916	1,7305648	0,201239788
3162	ENSG00000125871	-0,104033641	3,789966186	0,6936918	0,582417862
3163	ENSG00000125875	0,169733069	5,05176427	1,3835139	0,251459854
3164	ENSG00000125944	0,035705115	6,716958032	0,0858373	0,772142573
3165	ENSG00000125945	-0,331211798	5,335241264	2,3399198	0,13966836
3166	ENSG00000125952	0,071368923	4,853681761	0,4554356	0,585524424
3167	ENSG00000125968	0,368872818	5,940298243	4,1304725	0,053763324
3168	ENSG00000125970	-0,004620037	5,857916183	0,0010643	0,974254574
3169	ENSG00000125977	-0,301588785	6,250523206	3,5632954	0,071689731
3170	ENSG00000125991	0,085771689	6,007025929	0,2547407	0,618534973
3171	ENSG00000125995	-0,148987746	4,698856361	0,3506156	0,559513332
3172	ENSG00000126001	0,390049969	4,161745308	3,6483451	0,068611163
3173	ENSG00000126012	-0,018467831	5,549082812	0,0086555	0,926677745
3174	ENSG00000126016	0,573584226	4,227526572	4,5057962	0,044705683
3175	ENSG00000126067	-0,186476712	5,152373561	3,0192931	0,095541758
3176	ENSG00000126070	0,041535098	6,098037416	0,0568677	0,813618575
3177	ENSG00000126088	-0,067528593	5,310685855	0,1608418	0,69206623
3178	ENSG00000126107	0,112452511	4,11444656	0,333573	0,569146967
3179	ENSG00000126214	-0,005871078	4,987411578	0,001317	0,971362748
3180	ENSG00000126216	0,021150202	4,617222728	0,0289175	0,965085489
3181	ENSG00000126217	-0,249453414	4,975924512	1,1288679	0,298992787
3182	ENSG00000126226	-0,126815826	5,448572226	1,4188096	0,245646721
3183	ENSG00000126247	-0,100459388	6,933748198	0,5105364	0,482036776
3184	ENSG00000126261	0,090129078	6,238006099	0,3224176	0,575633349

3185	ENSG00000126267	-0,626846637	7,709321692	8,806453	0,006872284
3186	ENSG00000126351	0,345754401	5,566392167	3,3915686	0,078400565
3187	ENSG00000126432	-0,283117349	6,913241348	1,9714005	0,173594432
3188	ENSG00000126457	-0,159945188	6,224195115	0,7526385	0,394560438
3189	ENSG00000126458	0,098416933	4,916174849	0,2131671	0,648612441
3190	ENSG00000126524	0,031648526	6,980240246	0,047881	0,828707583
3191	ENSG00000126561	-0,044455448	4,684560986	0,0225134	0,882031161
3192	ENSG00000126581	-0,164855396	5,209683208	1,9007391	0,181148591
3193	ENSG00000126602	-0,042960356	4,918566933	0,0494268	0,826016497
3194	ENSG00000126653	-0,028464485	5,017146893	0,0648936	0,839973661
3195	ENSG00000126698	-0,16837846	5,87809658	1,1422697	0,296208964
3196	ENSG00000126709	0,798705462	4,394776996	12,003842	0,00209057
3197	ENSG00000126749	-0,237256375	4,329290326	3,8078158	0,118108717
3198	ENSG00000126756	-0,163030815	4,535022262	1,2377222	0,312126319
3199	ENSG00000126773	0,109652107	6,658607029	0,4781002	0,496171365
3200	ENSG00000126775	0,150487486	5,133831998	1,1055524	0,303905118
3201	ENSG00000126777	-0,266866563	8,664426661	1,8644852	0,185234286
3202	ENSG00000126785	-0,123073597	4,309112576	0,4952917	0,48860632
3203	ENSG00000126790	0,084341016	3,781749154	0,3495193	0,639030079
3204	ENSG00000126803	1,164939499	4,667033206	10,873607	0,003134932
3205	ENSG00000126804	0,200346202	5,441591313	1,986352	0,172009199
3206	ENSG00000126814	-0,00311212	4,188658679	0,0005229	0,981951306
3207	ENSG00000126821	-0,06965148	3,872985625	0,1225187	0,729488267
3208	ENSG00000126822	0,371455672	4,303853305	2,3967599	0,135171641
3209	ENSG00000126858	-0,175537543	5,815935608	1,2715581	0,271045031
3210	ENSG00000126870	-0,191768681	5,098835036	1,6582687	0,21058214
3211	ENSG00000126878	-0,552671489	4,757405747	12,388377	0,001827723
3212	ENSG00000126882	-0,321290812	5,000214858	1,7884182	0,1941319
3213	ENSG00000126883	-0,054215738	5,321869242	0,0992714	0,755532701
3214	ENSG00000126934	-0,171039502	5,34785832	0,7373036	0,399343773
3215	ENSG00000126945	-0,097287649	5,914345712	0,5407411	0,469496868
3216	ENSG00000126947	0,158074453	5,116015967	1,7086418	0,203972868
3217	ENSG00000127022	0,151935814	9,008841647	0,9651863	0,33602136
3218	ENSG00000127054	-0,050509919	4,923054341	0,1135144	0,739217258
3219	ENSG00000127081	-0,177695482	4,607226426	1,4466488	0,241239404
3220	ENSG00000127083	1,743712767	5,034551863	12,900412	0,001532279
3221	ENSG00000127125	0,058780065	4,805542203	0,1044302	0,749482188
3222	ENSG00000127184	-0,610544521	8,906910384	7,447696	0,011931386
3223	ENSG00000127241	0,731279197	4,866308111	4,2602971	0,050414242
3224	ENSG00000127311	0,077168329	3,759901449	0,1229268	0,729056931
3225	ENSG00000127314	0,100656463	6,442395344	0,2931868	0,593367162
3226	ENSG00000127328	-0,69319151	5,219800193	6,6605573	0,016675109
3227	ENSG00000127329	-0,341065866	6,817338098	3,0498777	0,094022372
3228	ENSG00000127334	-0,046070135	6,452539956	0,0583417	0,811266518
3229	ENSG00000127445	-0,108955186	4,768692944	0,3789324	0,544189726
3230	ENSG00000127463	-0,024470323	5,447846144	0,0226108	0,881778167
3231	ENSG00000127472	-0,257442966	6,049967498	2,2760172	0,144938185
3232	ENSG00000127481	-0,015767418	7,656478265	0,0043512	0,947974136
3233	ENSG00000127483	0,210622424	7,039860465	2,5202301	0,125934451
3234	ENSG00000127511	0,211521649	4,672455848	1,1927142	0,286036296
3235	ENSG00000127526	0,055882082	4,750493186	0,1711924	0,739620078
3236	ENSG00000127527	-0,10563379	5,204574894	0,2054487	0,654585067
3237	ENSG00000127540	-0,474134676	5,377944047	3,8067856	0,063269788
3238	ENSG00000127603	-0,031068642	10,15424064	0,0410926	0,841127072
3239	ENSG00000127616	-0,029785529	4,944161943	0,0203687	0,887749066
3240	ENSG00000127663	0,001553536	4,123390444	6,839E-05	0,99347287
3241	ENSG00000127720	-0,19938536	4,367148116	1,023708	0,322116523
3242	ENSG00000127804	0,136693114	4,774718629	0,892658	0,354517207
3243	ENSG00000127824	-0,683927605	5,425014475	3,7121663	0,066400037

3244	ENSG00000127837	0,254645618	5,332972744	2,553926	0,123603174
3245	ENSG00000127838	-0,210717611	4,741553512	1,4110811	0,246941114
3246	ENSG00000127863	-1,094201282	6,295452971	23,060944	7,53194E-05
3247	ENSG00000127870	0,033714187	5,085667816	0,0512303	0,822925926
3248	ENSG00000127884	-0,064347927	6,344448609	0,0951963	0,76043602
3249	ENSG00000127914	-0,124012193	7,96220174	0,5135806	0,480762326
3250	ENSG00000127920	-0,408663901	6,036793554	4,1375154	0,053575307
3251	ENSG00000127922	-0,326408315	5,649205781	6,4310258	0,018400129
3252	ENSG00000127946	-0,039389679	5,086131108	0,0346101	0,854038212
3253	ENSG00000127947	-0,001675255	6,063428062	8,874E-05	0,992564826
3254	ENSG00000127948	0,046097693	4,566533743	0,0438715	0,835928109
3255	ENSG00000127951	0,582719675	6,01082305	3,3943129	0,078287805
3256	ENSG00000127954	-0,849587148	6,895019157	11,882686	0,002181752
3257	ENSG00000127955	0,186824428	4,577654505	0,5536831	0,464315035
3258	ENSG00000127980	-0,160899272	5,279343375	0,8844964	0,356699235
3259	ENSG00000127989	-0,126936029	3,781858108	0,8288249	0,527244784
3260	ENSG00000127990	-0,103628291	5,801357065	0,4032619	0,531646312
3261	ENSG00000127993	0,014969735	4,013911753	0,0153491	0,939361385
3262	ENSG00000127995	-0,22675664	5,770267589	1,1186089	0,301147595
3263	ENSG00000128000	-0,153296742	6,005279584	0,6158995	0,44054549
3264	ENSG00000128016	-0,005007387	6,406175891	0,0001849	0,989268894
3265	ENSG00000128050	-0,153939958	5,195342632	2,1813694	0,185527089
3266	ENSG00000128052	0,993937732	6,564238421	3,5656983	0,071600615
3267	ENSG00000128159	0,280294594	4,479020425	1,8140085	0,191086925
3268	ENSG00000128185	0,050773333	4,42669247	0,0545138	0,817442809
3269	ENSG00000128191	0,014877028	4,178056277	0,0073613	0,999383636
3270	ENSG00000128245	0,081859802	5,560918417	0,2863513	0,597679623
3271	ENSG00000128272	-0,27720176	7,216004005	3,4494154	0,076022425
3272	ENSG00000128284	-0,248066999	5,190828177	1,9684563	0,173903245
3273	ENSG00000128294	-0,338745376	4,368978248	5,0039761	0,035211045
3274	ENSG00000128309	-0,359483096	4,538532315	3,1778224	0,087785844
3275	ENSG00000128335	-0,267908436	4,937251044	2,7510673	0,11070055
3276	ENSG00000128463	-0,201351947	4,418396852	2,9797371	0,160410504
3277	ENSG00000128487	0,378606978	4,723333083	5,3756052	0,029614127
3278	ENSG00000128512	-0,083927741	5,745480852	0,3207318	0,576626551
3279	ENSG00000128513	-0,232476533	4,773668254	1,61362	0,216622825
3280	ENSG00000128524	-0,266559983	4,459169608	3,3445795	0,080323148
3281	ENSG00000128534	-0,273888535	5,526090913	3,4253895	0,077023812
3282	ENSG00000128567	-0,327685741	6,651535068	2,4007624	0,134861532
3283	ENSG00000128578	-0,185872455	4,699929246	0,4914737	0,490269226
3284	ENSG00000128581	0,054039675	3,783518904	0,1316583	0,757989281
3285	ENSG00000128585	-0,184909123	7,087416005	1,7803368	0,195060997
3286	ENSG00000128590	-0,121050218	6,078049037	0,2802643	0,601576393
3287	ENSG00000128591	0,188107372	8,580873448	0,1765148	0,678265736
3288	ENSG00000128595	-0,362141425	8,401417865	2,3627263	0,137843052
3289	ENSG00000128607	0,146267098	5,688826428	1,7350385	0,200634156
3290	ENSG00000128609	-0,994892755	7,810264448	6,1641206	0,020727107
3291	ENSG00000128641	-0,041400539	5,802409926	0,0804775	0,779174618
3292	ENSG00000128654	-0,406328703	5,159949359	8,4151388	0,008015788
3293	ENSG00000128692	-0,223900749	3,925911941	1,9017283	0,238409494
3294	ENSG00000128694	-0,193832771	4,040104843	2,2426589	0,247883445
3295	ENSG00000128699	-0,041600429	5,215140661	0,0774108	0,783311329
3296	ENSG00000128708	-0,409106637	5,252470296	5,0116205	0,035096188
3297	ENSG00000128731	-0,155317612	7,026846901	0,473231	0,498350853
3298	ENSG00000128739	0,112787207	5,47391675	0,2021804	0,657155192
3299	ENSG00000128789	0,023524589	4,834531487	0,029228	0,865737356
3300	ENSG00000128791	0,296219033	4,967346819	2,4370922	0,132084951
3301	ENSG00000128829	0,061969743	5,683224451	0,4725537	0,498631664
3302	ENSG00000128833	-0,765826173	4,41069895	10,847069	0,003165504

3303	ENSG00000128849	1,039511084	6,148928883	5,8056916	0,024333019
3304	ENSG00000128872	0,153929405	5,626223532	0,4926185	0,489769598
3305	ENSG00000128881	-0,08805359	5,8856275	0,5148886	0,480195427
3306	ENSG00000128908	0,046073176	5,186930572	0,1010048	0,75347711
3307	ENSG00000128915	0,035053096	5,640755866	0,1049227	0,748903211
3308	ENSG00000128918	1,664092506	5,993988637	20,71029	0,000141085
3309	ENSG00000128923	-0,033607183	6,672194031	0,0376437	0,847855437
3310	ENSG00000128928	0,169976827	5,909742952	0,9462631	0,340743538
3311	ENSG00000128951	0,005020628	4,962605491	0,0016435	0,968009179
3312	ENSG00000128989	-0,070816727	7,479996118	0,1659168	0,687514454
3313	ENSG00000129003	-0,099635782	8,149374642	0,3771898	0,545088698
3314	ENSG00000129009	0,72679362	4,367269969	5,0125361	0,035081044
3315	ENSG00000129055	-0,182479703	5,76934724	0,6663763	0,422651924
3316	ENSG00000129071	-0,027940673	4,991105485	0,0691891	0,839347655
3317	ENSG00000129083	0,007174308	6,7056402	0,0017764	0,966742825
3318	ENSG00000129084	-0,282416952	4,621310431	1,6069453	0,217544433
3319	ENSG00000129103	0,285648752	4,904900789	2,1566878	0,155429737
3320	ENSG00000129116	-0,263091045	8,826196975	1,8457368	0,187359212
3321	ENSG00000129128	0,069132275	7,214884101	0,1278771	0,723888212
3322	ENSG00000129170	-0,82967235	10,03743232	5,8380973	0,023979828
3323	ENSG00000129187	0,437341488	4,924011935	11,156839	0,002818716
3324	ENSG00000129197	-0,147633871	4,444211738	2,3824899	0,300584557
3325	ENSG00000129235	-0,300320672	4,013725666	3,0309478	0,098377075
3326	ENSG00000129245	-0,194221781	5,689107589	1,3282599	0,260896187
3327	ENSG00000129250	-0,133984141	8,221504256	0,3984872	0,534065225
3328	ENSG00000129255	-0,389023463	4,179038156	5,5053526	0,027897148
3329	ENSG00000129292	-0,122227338	6,59332444	0,4779072	0,49625743
3330	ENSG00000129315	0,066777214	5,662498304	0,2814359	0,60080458
3331	ENSG00000129317	-0,00708044	5,241391593	0,0012653	0,97193019
3332	ENSG00000129351	0,026921978	6,597385382	0,0273036	0,87019344
3333	ENSG00000129353	-0,00244316	6,225520064	0,0001589	0,990050145
3334	ENSG00000129422	-0,157376862	7,81375682	0,9465332	0,340648729
3335	ENSG00000129460	-0,210230912	4,318060182	2,5710756	0,194448822
3336	ENSG00000129467	-0,294236487	4,739743906	2,4808266	0,128831773
3337	ENSG00000129472	0,15938363	4,40305792	2,6669505	0,247610342
3338	ENSG00000129473	0,228727083	5,598134272	3,3692142	0,079280815
3339	ENSG00000129484	-0,033605606	3,989739637	0,0587572	0,810602535
3340	ENSG00000129493	0,003512909	5,245865523	0,0012266	0,972361046
3341	ENSG00000129515	-0,156316734	6,163310189	0,5854503	0,451926559
3342	ENSG00000129518	0,108966095	4,413650334	0,3771914	0,545108673
3343	ENSG00000129521	0,05077139	6,103725083	0,024541	0,876876459
3344	ENSG00000129534	0,107379252	4,626471562	0,3403974	0,565250319
3345	ENSG00000129538	-0,54052614	7,368551616	6,7885589	0,015778664
3346	ENSG00000129559	-0,280256858	5,074454937	3,3356003	0,080741435
3347	ENSG00000129562	-0,247828736	5,891789804	1,7171915	0,202928465
3348	ENSG00000129566	0,01923196	4,878833594	0,0083253	0,92808564
3349	ENSG00000129595	0,215207414	4,713484892	2,2492259	0,147191919
3350	ENSG00000129596	-0,268296446	4,454126873	0,459057	0,504791287
3351	ENSG00000129625	-0,222735549	7,297565541	2,5837071	0,121497868
3352	ENSG00000129636	-0,118753287	6,219180122	0,5538714	0,46423996
3353	ENSG00000129657	0,08272645	5,98017355	0,2197132	0,643649504
3354	ENSG00000129675	0,094338643	6,270996359	0,5039946	0,484827079
3355	ENSG00000129680	0,097283756	4,873189349	1,0125253	0,332621699
3356	ENSG00000129691	0,077048713	4,973883643	0,5100809	0,514116955
3357	ENSG00000129757	-0,091672824	4,030969926	0,163324	0,689829624
3358	ENSG00000129824	0,569005677	6,306629174	0,6693186	0,421644024
3359	ENSG00000129933	0,266734942	4,981162091	2,0008279	0,17054425
3360	ENSG00000129991	-0,001430354	9,165923937	1,311E-05	0,997142328
3361	ENSG00000130021	-0,453283206	3,897284351	8,5462145	0,011372768

3362	ENSG00000130023	-0,008255497	4,204231171	0,0062416	0,953409238
3363	ENSG00000130024	0,076550686	6,560307837	0,3109327	0,582450738
3364	ENSG00000130037	-0,333019352	5,346139125	1,7055179	0,20441693
3365	ENSG00000130052	-0,265269059	3,848792215	1,5512984	0,225423029
3366	ENSG00000130054	-0,670065666	5,12902428	5,7498534	0,024955279
3367	ENSG00000130066	0,197409337	7,709174324	0,6118217	0,442042952
3368	ENSG00000130119	0,200035667	5,210070228	1,0214724	0,322634228
3369	ENSG00000130147	0,233104074	4,574217568	2,3718387	0,137117448
3370	ENSG00000130150	0,224590797	4,204043562	1,79395	0,193468638
3371	ENSG00000130158	0,199001149	4,542743254	0,6641171	0,423428329
3372	ENSG00000130159	-0,360984804	5,253109168	2,4174627	0,133576667
3373	ENSG00000130164	-0,42851312	4,633017461	1,4076815	0,24749472
3374	ENSG00000130175	0,15416608	5,458383497	0,5565331	0,463181078
3375	ENSG00000130176	-0,312709418	6,524283267	0,8662648	0,361611735
3376	ENSG00000130177	-0,077149232	5,697888546	0,526468	0,475353609
3377	ENSG00000130202	-0,158948545	4,644642517	0,5081157	0,483090843
3378	ENSG00000130203	0,480356528	5,158316211	1,8586796	0,185911087
3379	ENSG00000130224	-0,199553706	4,233118806	0,9499353	0,339826883
3380	ENSG00000130227	0,150199313	5,86521413	2,0763731	0,162960159
3381	ENSG00000130254	0,144964007	5,402771165	1,0121214	0,324797463
3382	ENSG00000130255	0,300283386	6,479034439	1,5061961	0,232074593
3383	ENSG00000130294	0,055640766	5,090370499	0,0354345	0,85233113
3384	ENSG00000130300	0,038933563	5,515961797	0,0174862	0,895942451
3385	ENSG00000130304	0,208633463	4,686001497	0,9111348	0,349690996
3386	ENSG00000130309	-0,206638695	5,150653516	1,6751641	0,208351297
3387	ENSG00000130312	-0,432016615	4,842437177	5,1369876	0,033089124
3388	ENSG00000130338	-0,052021227	6,698621174	0,1523649	0,699847488
3389	ENSG00000130340	0,059910574	5,902301871	0,2259345	0,639000693
3390	ENSG00000130347	-0,661372133	3,821555351	10,508799	0,003585221
3391	ENSG00000130348	-0,564237204	4,83869153	9,7509996	0,004765174
3392	ENSG00000130349	-0,387483071	4,140886159	5,888899	0,023437572
3393	ENSG00000130363	0,319037777	4,190448734	4,4563041	0,045795159
3394	ENSG00000130396	0,08285971	6,968745861	0,2870535	0,597233596
3395	ENSG00000130402	0,094138284	6,433267535	0,229816	0,63616519
3396	ENSG00000130413	-0,166343969	4,899634099	0,371424	0,548173895
3397	ENSG00000130414	-0,219442039	7,210177471	2,1848217	0,152841251
3398	ENSG00000130449	-0,180045579	4,145471557	0,9563567	0,338232191
3399	ENSG00000130508	0,143653292	5,379325629	0,2402187	0,628668505
3400	ENSG00000130517	0,299911986	4,906620341	3,2372631	0,085051903
3401	ENSG00000130520	-0,10037521	4,751289513	0,4574794	0,505507702
3402	ENSG00000130522	0,179867428	5,584885775	0,9824147	0,33186719
3403	ENSG00000130528	-0,546612327	9,008405758	3,9123857	0,059972926
3404	ENSG00000130544	0,145687575	3,757055953	1,0699939	0,420654477
3405	ENSG00000130559	0,306041592	4,822127648	5,4592163	0,028461382
3406	ENSG00000130560	-0,291636137	5,284655379	4,2917723	0,049603768
3407	ENSG00000130600	-0,536269119	7,004004983	4,9103435	0,036817486
3408	ENSG00000130635	0,280042209	5,424861041	1,0318912	0,320231263
3409	ENSG00000130638	0,043190571	6,472736248	0,1201139	0,732035536
3410	ENSG00000130640	0,030428293	5,027117296	0,0408449	0,841606009
3411	ENSG00000130695	0,013410902	5,145385615	0,0022008	0,962985672
3412	ENSG00000130702	0,018577444	5,362976987	0,0091056	0,924801337
3413	ENSG00000130703	0,225210676	5,383461575	2,036861	0,16689748
3414	ENSG00000130707	-0,043426301	5,323828083	0,0215358	0,884601343
3415	ENSG00000130713	-0,089284714	3,975224466	0,5462479	0,577969873
3416	ENSG00000130714	0,202480067	4,221599105	3,168335	0,229511648
3417	ENSG00000130723	0,286452513	6,029128835	0,7511344	0,39502579
3418	ENSG00000130724	-0,184160418	5,633645455	1,032595	0,320069826
3419	ENSG00000130725	-0,323228547	5,001298354	3,5817258	0,071009421
3420	ENSG00000130726	0,191625245	5,640924927	0,7655316	0,390604653

3421	ENSG00000130741	0,1576359	6,771916721	1,1831409	0,287901047
3422	ENSG00000130749	0,33825145	3,81611898	2,9142524	0,101201143
3423	ENSG00000130764	0,000120656	5,037840457	4,156E-07	0,999491121
3424	ENSG00000130770	0,201749971	6,38229659	1,4063665	0,247709285
3425	ENSG00000130779	-0,055873452	7,290507701	0,1904078	0,66661235
3426	ENSG00000130787	-0,087853815	4,922042571	0,1527618	0,699489414
3427	ENSG00000130803	-0,001411045	4,217983055	9,789E-05	0,992190995
3428	ENSG00000130811	0,117890037	5,644339908	0,4773859	0,496490038
3429	ENSG00000130816	-0,325777326	4,387920749	2,2054663	0,151034622
3430	ENSG00000130818	0,178020423	5,129751747	3,0142254	0,095804102
3431	ENSG00000130821	-0,060941196	5,886118151	0,0718484	0,79104203
3432	ENSG00000130826	-0,154787414	4,757270849	2,0835591	0,236585504
3433	ENSG00000130830	-0,490916055	4,899032921	12,142912	0,001985361
3434	ENSG00000130844	1,232666434	5,749491851	7,300349	0,012691533
3435	ENSG00000130856	0,007738158	4,423246837	0,0050289	0,979283871
3436	ENSG00000130935	-0,304621295	5,241289212	2,6285702	0,11852019
3437	ENSG00000130939	0,165110841	5,730635904	0,6954985	0,412836852
3438	ENSG00000130940	-0,110686844	3,9241776	0,1587537	0,693963451
3439	ENSG00000130956	0,005696298	4,469123322	0,0021461	0,965896648
3440	ENSG00000130962	-0,190339797	4,253480537	1,7767875	0,241695584
3441	ENSG00000130985	0,125979032	6,753318615	0,561973	0,461029187
3442	ENSG00000130988	-0,240235347	3,77523863	2,9914842	0,196119726
3443	ENSG00000131002	-0,004346038	5,295293287	8,829E-05	0,992583601
3444	ENSG00000131013	0,122663619	5,228592357	0,7077727	0,408795033
3445	ENSG00000131016	0,145596961	7,461496513	0,2700771	0,608221791
3446	ENSG00000131018	0,334751556	9,37908917	2,3644766	0,137667994
3447	ENSG00000131023	0,104972678	5,271163268	0,5298037	0,473989027
3448	ENSG00000131043	-0,069201986	4,324105552	0,2118845	0,649589631
3449	ENSG00000131051	-0,080353754	7,937311424	0,2782815	0,602840101
3450	ENSG00000131069	-0,356291732	6,245148936	3,6658335	0,067997017
3451	ENSG00000131089	0,338514497	6,014978581	3,0972226	0,091656679
3452	ENSG00000131100	-0,427800486	6,93624547	5,2682183	0,031124774
3453	ENSG00000131115	-0,037779981	3,95216431	0,1414707	0,845108481
3454	ENSG00000131127	-0,166674709	5,511921021	1,0013992	0,327334386
3455	ENSG00000131143	-0,405926642	8,655255035	4,4325199	0,046297062
3456	ENSG00000131148	-0,230690996	3,877488015	3,3051673	0,194040177
3457	ENSG00000131149	-0,18146893	5,630686033	0,5964212	0,447772196
3458	ENSG00000131165	0,080533133	4,619770894	0,2059678	0,654179131
3459	ENSG00000131171	0,086438796	6,145375592	0,116597	0,735839964
3460	ENSG00000131174	-0,788275759	8,21293038	11,481926	0,002515796
3461	ENSG00000131236	0,129681405	5,674562437	0,3587737	0,555014083
3462	ENSG00000131238	0,045608051	6,015320996	0,1008212	0,753690825
3463	ENSG00000131263	0,027957266	6,663062103	0,0559504	0,815091402
3464	ENSG00000131269	-0,217584134	5,082945645	2,6329765	0,118185166
3465	ENSG00000131323	0,339759069	4,250761573	5,8001676	0,035741719
3466	ENSG00000131368	-0,813445953	5,750384721	20,850401	0,000135768
3467	ENSG00000131373	-0,109250371	4,040795862	0,5184334	0,579678037
3468	ENSG00000131374	0,172408033	6,058126337	1,4400685	0,242253225
3469	ENSG00000131375	-0,046946862	5,922283874	0,0929431	0,76319679
3470	ENSG00000131378	-0,159319026	5,299132276	0,6542513	0,426845043
3471	ENSG00000131381	0,151476798	5,478672553	2,6197755	0,139661802
3472	ENSG00000131386	-1,191625194	5,332584509	6,9193239	0,014917656
3473	ENSG00000131389	1,090323236	6,558361371	7,3037368	0,012673466
3474	ENSG00000131408	-0,002222246	5,15961164	0,0001999	0,988841909
3475	ENSG00000131437	0,002536636	4,49812005	0,0004686	0,982915381
3476	ENSG00000131446	-0,089954532	6,090336361	0,2617286	0,613788355
3477	ENSG00000131459	-0,543507058	5,884354391	1,0949172	0,306204529
3478	ENSG00000131467	-0,195627831	5,268156371	2,8093473	0,107146254
3479	ENSG00000131469	0,009207108	7,755357569	0,0026064	0,959722083

3480	ENSG00000131471	0,588795356	5,720160952	1,4775519	0,236428574
3481	ENSG00000131473	-0,57574908	5,635651661	5,9671433	0,022628898
3482	ENSG00000131475	-0,181109339	4,292636784	1,3685286	0,253987215
3483	ENSG00000131495	-0,264160868	4,844289324	1,5990708	0,218638029
3484	ENSG00000131504	-0,040131669	5,58117523	0,0296664	0,864748654
3485	ENSG00000131507	0,11865804	6,605991493	1,1610096	0,292335891
3486	ENSG00000131508	-0,150878836	5,684384508	1,5711352	0,222530603
3487	ENSG00000131558	0,134266245	5,961631702	1,6743825	0,208409685
3488	ENSG00000131620	0,482206709	4,012458916	4,1864237	0,052290007
3489	ENSG00000131626	0,126272354	5,279487588	0,8970656	0,353333881
3490	ENSG00000131711	-0,391690985	8,843213731	3,3726025	0,079184976
3491	ENSG00000131724	0,340141149	5,811074155	2,4907855	0,128104289
3492	ENSG00000131725	0,195163827	4,645366279	2,3556837	0,138356603
3493	ENSG00000131730	-0,489610116	8,824229236	3,4602508	0,075633663
3494	ENSG00000131732	0,078966218	3,885616462	0,2546267	0,623150413
3495	ENSG00000131779	-0,025730642	3,958686927	0,0391175	0,886357162
3496	ENSG00000131791	0,113397144	5,684914048	0,3759134	0,545785074
3497	ENSG00000131828	-0,554790101	7,489820787	8,7073698	0,007147009
3498	ENSG00000131844	-0,18740203	5,939914574	1,6139307	0,216566885
3499	ENSG00000131845	0,147547172	4,284730348	1,3428574	0,371882994
3500	ENSG00000131849	0,049326338	3,619306879	0,0985511	0,859963567
3501	ENSG00000131871	-0,134502786	5,05593163	1,456145	0,278475612
3502	ENSG00000131873	-0,198465462	5,372688426	1,1617952	0,292215009
3503	ENSG00000131876	-0,356192104	4,161609231	7,0977526	0,030454355
3504	ENSG00000131931	-0,194671849	3,636398128	0,9180732	0,359134387
3505	ENSG00000131943	-0,274052168	4,922365	4,3796068	0,047503238
3506	ENSG00000131966	-0,299051298	5,879729376	3,2977782	0,082368854
3507	ENSG00000131981	0,084074563	6,515501849	0,2022607	0,657091715
3508	ENSG00000132002	0,677912733	5,342839161	16,421099	0,000490129
3509	ENSG00000132122	-0,024973948	4,5186255	0,0186548	0,892543573
3510	ENSG00000132128	0,135715283	5,27943129	0,7034185	0,410227979
3511	ENSG00000132153	-0,019727258	5,21838435	0,0161541	0,89996162
3512	ENSG00000132155	-0,338924951	7,280820416	5,0331738	0,034716875
3513	ENSG00000132170	0,23320366	5,081509163	0,2681646	0,609487238
3514	ENSG00000132199	-0,340695797	5,527993288	2,0487842	0,16571163
3515	ENSG00000132205	-0,691644181	4,406079751	6,3975485	0,018699783
3516	ENSG00000132254	-0,117731355	4,170817564	0,6490194	0,428658426
3517	ENSG00000132274	0,235611105	5,601660862	1,0523198	0,315590461
3518	ENSG00000132275	-0,087798327	4,389662147	0,522825	0,477762514
3519	ENSG00000132286	-0,026429092	4,461683555	0,0637912	0,857163116
3520	ENSG00000132294	0,066484076	6,620291877	0,2293583	0,636484157
3521	ENSG00000132300	-0,062360745	6,931070778	0,1033664	0,750716108
3522	ENSG00000132305	-0,226869498	7,278413892	1,7354191	0,200631131
3523	ENSG00000132313	-0,345519067	6,354148514	1,9638681	0,174385816
3524	ENSG00000132323	-0,014787088	4,494457522	0,0105126	0,919217276
3525	ENSG00000132326	1,024856947	4,029095183	17,370312	0,000367502
3526	ENSG00000132329	-0,490315801	5,784856243	2,8002694	0,107730857
3527	ENSG00000132334	-0,250695936	5,07130753	1,83135	0,189056865
3528	ENSG00000132341	-0,208322897	6,948168669	1,6102826	0,21708301
3529	ENSG00000132356	-0,167308241	5,984298392	1,378218	0,252335552
3530	ENSG00000132357	0,056824178	4,376112387	0,0843387	0,774091442
3531	ENSG00000132359	-0,982815732	5,619747377	10,098873	0,004177632
3532	ENSG00000132361	0,035810329	5,738125015	0,0229762	0,880834301
3533	ENSG00000132376	0,073771143	4,485687854	0,2767169	0,637168869
3534	ENSG00000132383	-0,041595529	5,720565355	0,0941544	0,761698575
3535	ENSG00000132386	-0,297184521	7,562633654	2,0796083	0,162692873
3536	ENSG00000132388	0,019695914	5,41003891	0,0281424	0,868228681
3537	ENSG00000132405	-0,02583059	4,655968216	0,0402413	0,842758134
3538	ENSG00000132406	0,06898213	3,709369602	0,2923053	0,70427588

3539	ENSG00000132423	-0,695733084	4,197764592	10,311796	0,003857537
3540	ENSG00000132424	-0,036452074	7,371159701	0,072542	0,790052497
3541	ENSG00000132429	-0,645936518	4,670490421	7,2957783	0,012715954
3542	ENSG00000132432	-0,470277568	5,309812066	3,0153138	0,09579414
3543	ENSG00000132434	0,025728389	4,014346418	0,0435054	0,912297185
3544	ENSG00000132436	0,134646487	3,616677935	0,8039007	0,467499311
3545	ENSG00000132463	-0,320087256	6,719079259	6,7112888	0,016287403
3546	ENSG00000132466	-0,036247032	7,272829933	0,116169	0,736295335
3547	ENSG00000132467	-0,064470101	4,308578973	0,2498077	0,703922333
3548	ENSG00000132471	0,161960409	5,590279604	1,0639412	0,312991377
3549	ENSG00000132475	0,041406397	7,728202572	0,0611365	0,806887607
3550	ENSG00000132478	-0,096279123	3,924622994	0,6943874	0,547032645
3551	ENSG00000132485	-0,148573371	6,342848903	0,7743078	0,387945614
3552	ENSG00000132507	-0,193485139	6,078178282	1,5724913	0,222370383
3553	ENSG00000132530	0,394602088	3,812544547	2,4873524	0,128354525
3554	ENSG00000132541	-0,345044771	4,300351243	1,7107677	0,203745873
3555	ENSG00000132549	0,068945084	6,782793496	0,3626823	0,552862868
3556	ENSG00000132561	0,774275505	6,686238856	4,0258844	0,056645284
3557	ENSG00000132570	0,048745211	4,120044886	0,0884734	0,797416705
3558	ENSG00000132581	-0,214947148	4,544195194	2,5551156	0,123473019
3559	ENSG00000132589	-0,16893611	5,512740535	0,8647524	0,362023603
3560	ENSG00000132600	0,165424885	4,157203031	0,7724784	0,388497672
3561	ENSG00000132604	0,15650599	4,17138911	1,2183118	0,368036719
3562	ENSG00000132612	-0,103645615	4,822399914	0,332067	0,570014062
3563	ENSG00000132640	-0,137690817	5,216769319	0,8063569	0,378458952
3564	ENSG00000132646	-0,187018992	4,250913091	2,052867	0,249073863
3565	ENSG00000132664	-0,026065351	3,715814437	0,0309766	0,94019431
3566	ENSG00000132669	0,021085892	6,019660016	0,034202	0,854885482
3567	ENSG00000132670	0,364932636	5,481951356	8,9461417	0,006488834
3568	ENSG00000132676	-0,202523403	6,185818602	2,0351974	0,167028434
3569	ENSG00000132680	-0,090650835	5,110253835	0,3303575	0,57100154
3570	ENSG00000132688	0,42534241	8,609844167	2,4104143	0,134117165
3571	ENSG00000132694	0,088624854	5,42276253	0,1574449	0,695159975
3572	ENSG00000132716	0,097590353	5,592051064	0,4597941	0,504443307
3573	ENSG00000132718	0,130489345	5,12377335	0,4605781	0,504093155
3574	ENSG00000132763	-0,250255928	3,891847653	2,0338935	0,167194214
3575	ENSG00000132780	-0,10634754	5,856482014	0,6163127	0,440366578
3576	ENSG00000132792	-0,185240684	4,999477282	2,4576817	0,1304943
3577	ENSG00000132819	-0,649307766	5,244564539	4,0003535	0,057375142
3578	ENSG00000132821	-0,531921178	5,055961774	2,8482284	0,104925335
3579	ENSG00000132823	-0,016379529	4,944454527	0,016527	0,994590446
3580	ENSG00000132824	0,082093388	7,171450767	0,6515066	0,427774695
3581	ENSG00000132840	-0,566431318	4,044837871	7,4981103	0,01168297
3582	ENSG00000132842	0,112339857	5,721993393	1,2497615	0,275044002
3583	ENSG00000132849	-0,374825685	7,125080109	3,6040096	0,070196711
3584	ENSG00000132912	-0,055133821	6,148478843	0,3896548	0,583836103
3585	ENSG00000132938	-0,285796499	5,877929331	1,1508586	0,294443164
3586	ENSG00000132950	0,036027529	4,168084607	0,0668872	0,798207373
3587	ENSG00000132952	-0,04966456	4,590757394	0,1681192	0,775064796
3588	ENSG00000132953	0,034227719	5,838478235	0,0611356	0,806886843
3589	ENSG00000132963	-0,629167304	6,36119404	7,6085906	0,011158317
3590	ENSG00000132964	0,253391117	4,131155287	2,3513119	0,138753026
3591	ENSG00000132967	-0,059600029	5,995780847	0,0848259	0,773458701
3592	ENSG00000132970	-0,19934642	3,788727958	1,0862009	0,308093887
3593	ENSG00000133026	1,341257731	7,228184893	17,257228	0,000380178
3594	ENSG00000133028	-0,126229084	5,178307377	1,3582794	0,285597993
3595	ENSG00000133030	0,202423135	7,419954281	1,1738325	0,289788586
3596	ENSG00000133056	0,055997257	4,757042997	0,0810085	0,778469944
3597	ENSG00000133059	0,09588801	4,822337612	0,3813235	0,542932446

3598	ENSG00000133065	-0,333508149	7,549712874	1,4718864	0,237302016
3599	ENSG00000133083	-1,057235683	5,831060689	10,279573	0,003904202
3600	ENSG00000133103	0,006634365	5,529296047	0,0008778	0,976618541
3601	ENSG00000133104	-0,098983189	6,267008855	0,424802	0,520979673
3602	ENSG00000133110	1,80015415	6,498933148	9,1427076	0,006023153
3603	ENSG00000133112	0,086440467	10,54868795	0,1090725	0,74417173
3604	ENSG00000133114	0,066229066	4,70394134	0,3984634	0,59772969
3605	ENSG00000133121	-0,357309296	5,669098922	9,5830096	0,005067402
3606	ENSG00000133131	0,57737093	4,18936609	6,369361	0,018932485
3607	ENSG00000133138	0,26234065	4,076617009	2,959395	0,098743252
3608	ENSG00000133142	-0,13533596	5,882626149	0,929813	0,344861542
3609	ENSG00000133226	0,132148688	6,108993279	0,8993881	0,352729142
3610	ENSG00000133302	-0,174845031	4,246921603	0,9129811	0,349212501
3611	ENSG00000133313	-0,188016736	5,385218305	1,3579574	0,255777768
3612	ENSG00000133318	0,010298831	6,470964465	0,0030146	0,956685919
3613	ENSG00000133392	-0,00616584	7,261314206	0,0003514	0,985204561
3614	ENSG00000133393	-0,100102724	4,830963075	0,6128492	0,531684109
3615	ENSG00000133401	0,018426142	6,768799355	0,0072668	0,932800455
3616	ENSG00000133422	-0,068873258	4,584011466	0,1077926	0,745627298
3617	ENSG00000133424	-0,053802409	6,306411045	0,0545341	0,817409335
3618	ENSG00000133454	0,311953778	7,536852182	0,7985587	0,380735599
3619	ENSG00000133460	-0,226528307	3,922408347	1,1897942	0,286612331
3620	ENSG00000133561	-0,174833838	5,45039599	1,2971598	0,266400017
3621	ENSG00000133574	-0,168131205	5,205115568	0,6636192	0,423599749
3622	ENSG00000133606	0,126898031	6,367450243	1,0968699	0,305745968
3623	ENSG00000133624	-0,203456558	4,026093704	1,30944	0,264208871
3624	ENSG00000133639	0,204322542	6,996540863	2,1604913	0,155034889
3625	ENSG00000133641	0,067100142	4,198359857	0,0848947	0,773369632
3626	ENSG00000133657	0,32493601	8,175847675	1,9334796	0,177623499
3627	ENSG00000133687	-0,594090666	6,761332893	10,873357	0,003135218
3628	ENSG00000133703	-0,127010734	4,984752282	0,7312077	0,401269418
3629	ENSG00000133704	0,112633815	5,751185739	1,5054502	0,232144009
3630	ENSG00000133706	-0,128412603	6,953183909	0,6999217	0,411376734
3631	ENSG00000133731	-0,583179376	5,366443061	3,1485712	0,089168388
3632	ENSG00000133739	0,227088993	4,008989097	1,401117	0,248568239
3633	ENSG00000133773	-0,097746389	4,296074403	0,4360048	0,619931696
3634	ENSG00000133789	0,095022703	5,752419113	0,5787292	0,454475812
3635	ENSG00000133794	-1,057971337	4,019907233	30,683422	1,21609E-05
3636	ENSG00000133800	-1,356269027	6,556875381	22,413385	8,92244E-05
3637	ENSG00000133812	-0,049215388	6,312553379	0,2480331	0,623152891
3638	ENSG00000133816	0,870888758	6,580950443	19,707415	0,000186466
3639	ENSG00000133818	0,216034602	5,275313285	0,9131561	0,349167197
3640	ENSG00000133835	-0,238367099	7,016470857	2,774155	0,109257022
3641	ENSG00000133858	0,166579184	6,118153852	0,7626717	0,391477018
3642	ENSG00000133872	0,205021897	7,319973984	2,3978175	0,135042513
3643	ENSG00000133878	0,079703379	4,79043958	0,1254096	0,726450183
3644	ENSG00000133884	0,09318521	4,832894002	0,2070789	0,653312387
3645	ENSG00000133943	0,064048418	6,758310735	0,0852453	0,772915732
3646	ENSG00000133961	0,042840657	5,369188245	0,0879206	0,769481879
3647	ENSG00000133997	-0,022515618	4,217453844	0,043512	0,916892479
3648	ENSG00000134001	-0,290016447	6,168538344	3,3059069	0,08201593
3649	ENSG00000134014	0,024314562	5,041944509	0,0652854	0,800581352
3650	ENSG00000134020	-0,44462721	5,631871826	2,1524949	0,155814659
3651	ENSG00000134030	0,195457115	5,031023228	0,817867	0,375134889
3652	ENSG00000134046	0,04867374	5,622061382	0,103656	0,750379454
3653	ENSG00000134049	-0,54071421	4,863990144	4,0848254	0,055000195
3654	ENSG00000134056	-0,719380354	5,655575297	6,0272746	0,022028513
3655	ENSG00000134077	-0,208207235	5,000093583	3,9268798	0,08311977
3656	ENSG00000134107	0,279277627	5,854949442	0,8815492	0,35748686

3657	ENSG00000134108	0,004968168	6,314075535	0,0013339	0,9711781
3658	ENSG00000134109	0,340717142	4,85618381	4,1583659	0,053023035
3659	ENSG00000134121	-1,188822437	6,02299841	17,18936	0,000388014
3660	ENSG00000134138	0,463828146	4,983574009	3,1722023	0,08804954
3661	ENSG00000134146	-0,269736133	4,419611331	1,4113704	0,246894083
3662	ENSG00000134152	-0,026463129	4,673130785	0,0218223	0,883842065
3663	ENSG00000134153	-0,328096044	5,084451551	10,133077	0,005022286
3664	ENSG00000134186	-0,020505206	5,189990764	0,021266	0,885318591
3665	ENSG00000134202	-0,32464659	5,658601316	2,806135	0,10738307
3666	ENSG00000134243	0,037443555	7,911317025	0,0507847	0,823678972
3667	ENSG00000134245	-0,09398206	4,033054185	0,283239	0,599665333
3668	ENSG00000134247	-0,142909856	7,222672922	0,6473272	0,429268661
3669	ENSG00000134248	-0,40876795	5,266453187	7,4451389	0,011940636
3670	ENSG00000134250	0,439950067	7,251499075	3,8312501	0,062488023
3671	ENSG00000134253	0,185455606	4,013925849	0,6975776	0,41214954
3672	ENSG00000134255	-0,066014875	4,876145312	0,164077	0,689155018
3673	ENSG00000134262	0,070435637	3,898417099	0,320343	0,722582069
3674	ENSG00000134265	-0,106257166	5,814696274	0,3379315	0,566652213
3675	ENSG00000134278	0,276107712	5,29208678	3,0343773	0,094812193
3676	ENSG00000134283	-0,088851568	5,202866336	0,5642927	0,49666697
3677	ENSG00000134287	0,294471081	5,103630568	2,3471075	0,139090011
3678	ENSG00000134291	-0,151665406	5,458902008	1,3702762	0,253651553
3679	ENSG00000134294	0,256218358	8,077649447	0,4906945	0,49060977
3680	ENSG00000134308	0,400540471	7,187272978	11,282276	0,002693734
3681	ENSG00000134313	0,210807074	7,155824918	4,3443488	0,048331776
3682	ENSG00000134318	-0,044695413	7,12108444	0,0956632	0,75986054
3683	ENSG00000134321	0,087527249	3,808634307	0,0787181	0,781538267
3684	ENSG00000134324	-0,230931572	5,86194569	1,2534835	0,274388477
3685	ENSG00000134330	-0,09809595	5,115631841	0,7418085	0,411452237
3686	ENSG00000134333	-0,467550158	8,249644372	6,5733319	0,017302503
3687	ENSG00000134352	0,056140441	8,87582266	0,0453359	0,83325441
3688	ENSG00000134369	0,009582805	5,770059965	0,001107	0,973743917
3689	ENSG00000134371	0,04752051	6,171291252	0,1720835	0,682078679
3690	ENSG00000134375	-0,859914298	6,048680697	16,827222	0,000432918
3691	ENSG00000134419	0,211504895	7,130390915	0,7665459	0,390295951
3692	ENSG00000134440	-0,058284279	7,193268139	0,0985719	0,756366458
3693	ENSG00000134444	-0,075213359	5,543767461	0,4084619	0,529013071
3694	ENSG00000134452	-0,059200109	5,894977009	0,1438508	0,707944661
3695	ENSG00000134453	-0,289769954	5,705056092	6,5845386	0,017207603
3696	ENSG00000134463	0,013983756	4,426976715	0,002326	0,961948352
3697	ENSG00000134480	-0,226066925	5,431795582	2,3166053	0,141564135
3698	ENSG00000134504	-0,108412223	4,225668463	0,3491673	0,560319549
3699	ENSG00000134516	0,13704622	4,51897464	0,2560751	0,617622288
3700	ENSG00000134531	-0,031696344	7,443422918	0,0160183	0,900380542
3701	ENSG00000134532	-0,256136129	4,301677626	2,1886388	0,152533782
3702	ENSG00000134569	0,087282674	4,706733679	0,0695575	0,794319289
3703	ENSG00000134571	-0,029577836	10,26850995	0,0108088	0,918093549
3704	ENSG00000134575	-0,118144686	4,012720981	0,5316515	0,47323802
3705	ENSG00000134590	-0,070465557	6,137366817	0,1589704	0,693765906
3706	ENSG00000134597	-0,018890853	3,822662357	0,0280794	0,943271162
3707	ENSG00000134644	0,154889701	6,251695672	1,8779217	0,183689195
3708	ENSG00000134684	-0,535512469	5,245684076	8,388388	0,008116893
3709	ENSG00000134686	-0,347848701	5,987674275	3,7384544	0,065512994
3710	ENSG00000134697	-0,134587864	5,194130465	0,9433835	0,341436466
3711	ENSG00000134698	0,002055449	5,83033213	0,000214	0,988453895
3712	ENSG00000134709	-0,14771089	4,357647406	0,279388	0,602141895
3713	ENSG00000134716	0,170052396	6,693931639	0,2503934	0,621529629
3714	ENSG00000134717	-0,082675027	5,457824698	0,3267944	0,573063014
3715	ENSG00000134744	0,030498625	6,077820733	0,0597337	0,809066514

3716	ENSG00000134748	0,044159819	4,671339031	0,1520246	0,705845414
3717	ENSG00000134755	-0,247906464	5,510318621	3,4239491	0,077036867
3718	ENSG00000134758	0,07484715	4,389320535	0,1168695	0,735543791
3719	ENSG00000134759	0,13812256	6,245247943	1,4417255	0,241979856
3720	ENSG00000134765	-0,136218473	6,340327518	0,1384446	0,713221961
3721	ENSG00000134769	-0,508244552	7,121049422	5,7727858	0,0246976
3722	ENSG00000134775	-0,042073584	6,995543144	0,0179853	0,894477116
3723	ENSG00000134779	-0,042299998	5,304518032	0,1024986	0,751717919
3724	ENSG00000134802	-0,483534483	4,138822683	5,1337638	0,033139096
3725	ENSG00000134817	0,23657018	4,641540673	0,2942859	0,592679857
3726	ENSG00000134824	-0,61683766	4,789920335	3,8681851	0,061328503
3727	ENSG00000134825	-0,113487293	4,951990687	0,5123605	0,481288433
3728	ENSG00000134851	-0,179355731	6,068869299	0,9673848	0,335517848
3729	ENSG00000134852	-0,103617075	6,621130365	0,6195815	0,439172583
3730	ENSG00000134853	-0,279206414	6,946265726	1,3374619	0,259296118
3731	ENSG00000134871	-0,110480696	7,545426153	0,2071078	0,65328984
3732	ENSG00000134874	0,36176603	3,918610112	2,7477937	0,110901509
3733	ENSG00000134882	-0,070234691	5,120209805	0,4788935	0,522712591
3734	ENSG00000134884	0,121876786	6,835577467	0,5729102	0,456751884
3735	ENSG00000134897	-0,148065911	4,910577485	1,0515865	0,315740532
3736	ENSG00000134900	-0,140008739	6,543634932	1,1281309	0,299108981
3737	ENSG00000134905	0,094579941	4,862000012	0,645919	0,479993644
3738	ENSG00000134909	0,283648919	4,656924018	1,6427802	0,212653473
3739	ENSG00000134910	-0,045031972	6,436151546	0,1568198	0,695720878
3740	ENSG00000134954	-0,113627486	6,211915302	0,4854331	0,492920061
3741	ENSG00000134970	-0,191690386	6,746045924	0,5144367	0,480410952
3742	ENSG00000134982	-0,062864775	6,863875212	0,1584376	0,694251908
3743	ENSG00000134986	0,276601366	3,626717823	0,8981173	0,353091031
3744	ENSG00000134987	-0,10357133	5,564280378	0,3391684	0,565948146
3745	ENSG00000134996	-0,063686031	4,592919444	0,2792978	0,68508225
3746	ENSG00000135002	-0,198234141	4,733928013	1,3418428	0,258538816
3747	ENSG00000135018	0,069995254	6,819441369	0,4000904	0,533229244
3748	ENSG00000135040	-0,047407093	5,206063238	0,199977	0,664278087
3749	ENSG00000135046	0,288910193	7,386425282	0,7357566	0,399831135
3750	ENSG00000135047	-0,670063862	6,84231435	19,669476	0,00018773
3751	ENSG00000135048	-0,32935768	5,460740618	0,8488835	0,366386059
3752	ENSG00000135049	-0,581550025	6,803085136	4,5170787	0,044461429
3753	ENSG00000135052	0,502067557	4,367503224	4,9103119	0,036818038
3754	ENSG00000135063	0,050545444	4,894398196	0,0387852	0,845596406
3755	ENSG00000135070	-0,191281537	5,969744672	1,6661053	0,209527368
3756	ENSG00000135074	-0,280256763	5,957743182	0,8331619	0,370783221
3757	ENSG00000135090	0,074200246	5,197035983	0,5725057	0,456882445
3758	ENSG00000135093	0,248837443	3,890983555	6,046247	0,16167369
3759	ENSG00000135108	-0,049612947	6,153052051	0,1185183	0,733752192
3760	ENSG00000135111	-0,886902476	4,055987055	17,127763	0,000395278
3761	ENSG00000135148	0,238742453	4,844118783	3,3604906	0,079645344
3762	ENSG00000135164	0,081720661	6,309592419	0,4127021	0,526900891
3763	ENSG00000135205	0,102982965	4,30110043	0,2420377	0,627378537
3764	ENSG00000135218	-0,353179539	9,660313006	1,3739863	0,253069132
3765	ENSG00000135241	-0,316273198	6,134592668	1,536264	0,227613119
3766	ENSG00000135249	-0,090328176	3,891225255	0,697102	0,612165547
3767	ENSG00000135250	0,143836081	5,342196963	2,0417079	0,166367903
3768	ENSG00000135269	-0,404074272	4,60185821	5,4891872	0,02810488
3769	ENSG00000135272	0,451180307	5,706948644	3,5191417	0,073350104
3770	ENSG00000135297	-0,075953447	4,676409057	0,678652	0,551916305
3771	ENSG00000135299	0,101633163	3,85316997	0,583828	0,572184519
3772	ENSG00000135315	0,23153696	4,239877279	1,8180889	0,190606859
3773	ENSG00000135316	0,062053282	7,066662077	0,2259649	0,638978287
3774	ENSG00000135317	-0,09160695	5,64013466	0,4700787	0,499747132

3775	ENSG00000135318	0,485552539	4,23639367	5,2945134	0,030747063
3776	ENSG00000135333	0,078530888	4,87499507	0,0925124	0,763728681
3777	ENSG00000135334	0,02192272	4,396193723	0,0369693	0,901931319
3778	ENSG00000135336	-0,209472532	5,429299023	0,7372299	0,399366966
3779	ENSG00000135338	0,174063815	3,711871083	0,7215116	0,404361266
3780	ENSG00000135341	0,017365347	5,884425217	0,0158849	0,900790476
3781	ENSG00000135365	-0,050079848	5,377561833	0,1130566	0,739724312
3782	ENSG00000135372	0,08337992	4,594765304	0,1901677	0,666823216
3783	ENSG00000135387	0,110043249	7,327643318	1,309937	0,264080212
3784	ENSG00000135390	-0,357494245	7,09148083	4,3484056	0,048275756
3785	ENSG00000135404	-0,149459997	8,770598227	1,1273402	0,299274442
3786	ENSG00000135414	-0,505227787	4,643415062	5,5575117	0,027238478
3787	ENSG00000135424	-0,159027259	8,124567864	0,3974261	0,534605602
3788	ENSG00000135441	-0,171529246	3,73934895	0,8281191	0,372209831
3789	ENSG00000135446	0,265295666	4,200395948	4,003432	0,104107443
3790	ENSG00000135447	-0,820057425	6,204352115	6,2243947	0,020181204
3791	ENSG00000135452	-0,044965716	4,606307864	0,206209	0,680510211
3792	ENSG00000135457	0,110954988	4,674158819	0,9850395	0,436005956
3793	ENSG00000135469	-0,715295142	5,760569824	8,6347755	0,00735596
3794	ENSG00000135473	0,051293608	4,964003572	0,064876	0,801201518
3795	ENSG00000135486	0,286075861	8,145953564	4,8380407	0,03806718
3796	ENSG00000135503	-0,16014198	4,171933263	0,6267282	0,436607763
3797	ENSG00000135506	0,14522154	6,602303156	1,0053701	0,326370537
3798	ENSG00000135521	-0,307727962	3,719890909	4,2740424	0,114017525
3799	ENSG00000135535	0,015078172	7,53064814	0,004901	0,944790095
3800	ENSG00000135537	-0,19664175	3,69151599	0,9536347	0,338906885
3801	ENSG00000135540	0,434224821	5,537772529	5,8932334	0,023391942
3802	ENSG00000135541	0,103355366	5,817583385	0,3142305	0,580489547
3803	ENSG00000135596	-0,308661821	4,011731585	2,0058978	0,170025344
3804	ENSG00000135597	0,114959155	5,535293444	1,4216401	0,248722375
3805	ENSG00000135617	-0,447821306	4,99649439	5,2191171	0,031844054
3806	ENSG00000135624	-0,275751442	7,025767078	4,2926687	0,049575878
3807	ENSG00000135631	0,000348093	4,53746823	1,799E-06	0,998941424
3808	ENSG00000135632	0,009128491	4,022246883	0,0035142	0,993174825
3809	ENSG00000135636	-0,281311033	5,909058156	1,0107545	0,325132159
3810	ENSG00000135655	-0,220524777	6,882799294	1,0313612	0,320352916
3811	ENSG00000135677	-0,16796084	7,360890473	2,0577622	0,164779094
3812	ENSG00000135678	-0,588741848	4,705647545	1,2220894	0,28032593
3813	ENSG00000135679	0,077664635	5,969634621	0,1658111	0,687608467
3814	ENSG00000135686	0,168680713	5,223086658	0,9705479	0,33474491
3815	ENSG00000135709	-0,262774711	4,006794403	1,1732079	0,289913825
3816	ENSG00000135720	0,285579202	6,607134593	7,100549	0,01378541
3817	ENSG00000135744	-0,010539408	6,415514631	0,0036395	0,952412698
3818	ENSG00000135750	-0,460722619	5,553539989	4,7012463	0,040679527
3819	ENSG00000135775	-0,164724389	5,152785376	1,5926444	0,219492157
3820	ENSG00000135776	0,178571279	4,257080974	2,7551341	0,235545544
3821	ENSG00000135778	-0,253920846	5,181620965	3,9751065	0,058065109
3822	ENSG00000135801	0,009826429	4,058777325	0,0069298	0,978346714
3823	ENSG00000135821	-0,915897603	8,65691816	9,0952971	0,006135559
3824	ENSG00000135823	0,294472907	4,563130199	7,6509681	0,024518351
3825	ENSG00000135828	0,108518747	3,950807849	0,3429514	0,56380558
3826	ENSG00000135829	0,041402108	6,806490905	0,1438183	0,707963965
3827	ENSG00000135837	0,041619668	7,156591312	0,1672182	0,6863473
3828	ENSG00000135842	-0,118319839	8,896415273	0,2104735	0,650681563
3829	ENSG00000135845	-0,044964674	4,337787648	0,1227963	0,841955479
3830	ENSG00000135862	-0,032437537	8,168426061	0,0350908	0,85303594
3831	ENSG00000135870	0,086531783	6,229147113	0,3413014	0,564730124
3832	ENSG00000135899	0,064522378	3,794265376	0,094619	0,761139953
3833	ENSG00000135900	-0,167022155	4,492506135	1,8802998	0,201949147

3834	ENSG00000135905	0,588316443	4,172297342	3,9432187	0,059047479
3835	ENSG00000135913	0,034285332	4,299811846	0,0606877	0,807580837
3836	ENSG00000135916	0,051038486	5,039240218	0,1071806	0,7463239
3837	ENSG00000135919	0,542649588	5,837261399	3,3306185	0,080953664
3838	ENSG00000135924	0,199931438	5,34783486	1,3655363	0,254492398
3839	ENSG00000135926	-0,055912883	6,664327066	0,0747599	0,786956782
3840	ENSG00000135929	0,783912213	3,965093181	13,700226	0,00117005
3841	ENSG00000135930	-0,049232109	4,982753212	0,2507479	0,621267757
3842	ENSG00000135932	-0,179706117	6,858853457	1,7423005	0,199727557
3843	ENSG00000135940	-0,499099117	7,894560311	4,3580033	0,048049065
3844	ENSG00000135945	0,016618943	5,916985568	0,0186755	0,89248014
3845	ENSG00000135956	0,06570085	6,24791843	0,1439676	0,707831853
3846	ENSG00000135966	0,258473138	4,237105056	2,649463	0,117141765
3847	ENSG00000135968	0,132231549	6,251327391	1,1110891	0,302702727
3848	ENSG00000135972	-0,466177275	4,823206337	7,9803845	0,009574986
3849	ENSG00000135974	0,126914591	4,721865626	0,9169028	0,348164999
3850	ENSG00000135976	0,149119516	6,775693175	0,7873847	0,384032977
3851	ENSG00000135999	0,121763243	5,364181672	1,6213567	0,215516969
3852	ENSG00000136003	-0,156898886	7,425500623	0,6662402	0,422698631
3853	ENSG00000136010	-0,013759911	3,94728188	0,0041177	0,949387261
3854	ENSG00000136021	0,157829647	5,92956869	0,7848472	0,384787651
3855	ENSG00000136026	0,200969587	5,569112087	2,0391178	0,166636902
3856	ENSG00000136040	-0,09078939	4,701996894	0,1956147	0,6623952
3857	ENSG00000136044	-0,065000906	5,180890879	0,1533115	0,698977264
3858	ENSG00000136045	-0,023170883	4,710458407	0,0207207	0,886790164
3859	ENSG00000136048	-0,336745289	4,041089958	2,343754	0,139359487
3860	ENSG00000136051	-0,033606145	6,579053648	0,0290463	0,866155229
3861	ENSG00000136052	1,444248641	4,50585509	6,9905352	0,014470748
3862	ENSG00000136068	0,06421863	6,440134209	0,0672376	0,797697661
3863	ENSG00000136098	0,167606964	3,928082542	1,0861131	0,34365375
3864	ENSG00000136100	-0,197139087	6,10711079	2,2985109	0,143009622
3865	ENSG00000136104	-0,24190926	5,60948987	3,2404259	0,084863468
3866	ENSG00000136108	-0,003848526	3,893254171	0,000404	0,984135903
3867	ENSG00000136111	0,033691068	6,922136163	0,0270425	0,870809606
3868	ENSG00000136114	-0,073869394	3,76325401	0,2783127	0,636571802
3869	ENSG00000136141	0,053941803	4,819656079	0,2043118	0,659263608
3870	ENSG00000136143	-0,518568386	6,781239079	4,023396	0,056715954
3871	ENSG00000136144	0,00372053	5,077452819	0,000555	0,981407163
3872	ENSG00000136146	-0,154514475	4,827362804	1,0415397	0,318027812
3873	ENSG00000136147	-0,034575933	4,362762697	0,0730672	0,865835379
3874	ENSG00000136149	0,254755152	5,714560097	1,4877382	0,23486842
3875	ENSG00000136152	0,122958684	5,416998744	1,0180488	0,32339313
3876	ENSG00000136153	0,159853729	8,041298277	0,4456544	0,511017045
3877	ENSG00000136156	0,141694624	8,601472889	0,6857731	0,416045988
3878	ENSG00000136158	0,249208824	4,841100494	3,3099372	0,081804541
3879	ENSG00000136159	-0,332860268	3,651184511	7,4503285	0,097722974
3880	ENSG00000136160	-0,712327454	5,350384312	4,9500382	0,036131799
3881	ENSG00000136161	0,181475711	4,955561484	1,3840287	0,251390983
3882	ENSG00000136167	-0,253695005	5,895738851	0,6143827	0,441101526
3883	ENSG00000136169	0,103439798	5,150821356	0,507091	0,483522943
3884	ENSG00000136193	0,36739672	5,769672302	6,6040165	0,017068764
3885	ENSG00000136205	0,351189344	6,425841126	3,049127	0,094060447
3886	ENSG00000136235	0,098220078	7,290462079	0,2368485	0,631074718
3887	ENSG00000136237	0,178764513	5,615634326	0,7995788	0,38043664
3888	ENSG00000136238	-0,041017344	6,760658512	0,093348	0,762688459
3889	ENSG00000136240	0,071302306	6,575914013	0,2622361	0,613429863
3890	ENSG00000136243	-0,162339478	3,791155028	1,8698448	0,384230497
3891	ENSG00000136247	-0,174625597	4,404331388	1,324335	0,261570322
3892	ENSG00000136261	-0,480746941	7,250522406	3,4464385	0,076180981

3893	ENSG00000136270	-0,163610442	4,984147546	0,7085271	0,408558487
3894	ENSG00000136271	-0,052688577	4,803666657	0,1226317	0,729367768
3895	ENSG00000136273	0,018191478	4,169013827	0,0161883	0,987270772
3896	ENSG00000136279	-0,018616251	5,377552573	0,0127363	0,91111982
3897	ENSG00000136280	-0,077905766	4,224915582	0,229413	0,636459819
3898	ENSG00000136319	0,307442193	3,831714115	6,3654016	0,085844232
3899	ENSG00000136378	0,287807124	5,173073443	1,9414729	0,176764822
3900	ENSG00000136381	-0,055405332	5,970632596	0,2659791	0,610923294
3901	ENSG00000136383	0,529585378	7,305711708	3,0146116	0,095830536
3902	ENSG00000136436	-0,485730703	7,334747571	18,138996	0,000290779
3903	ENSG00000136444	-0,179237533	4,784566751	1,8048605	0,192123556
3904	ENSG00000136448	0,015868956	5,829870294	0,013175	0,909605872
3905	ENSG00000136450	-0,026175558	6,160070339	0,0178968	0,894735553
3906	ENSG00000136451	-0,041683535	5,937980253	0,1864711	0,669857756
3907	ENSG00000136463	-0,3929157	4,252005506	5,3502295	0,029963556
3908	ENSG00000136478	-0,125494172	6,01326686	1,0501329	0,316046163
3909	ENSG00000136485	0,031360689	5,567956534	0,0316105	0,86043423
3910	ENSG00000136504	0,025401101	5,94585245	0,0289396	0,866398306
3911	ENSG00000136518	-0,108840523	4,166654714	0,6435058	0,538115639
3912	ENSG00000136521	-0,345519935	7,206477246	2,0988005	0,16084682
3913	ENSG00000136522	-0,562215373	5,147084504	5,3648871	0,029761158
3914	ENSG00000136527	-0,013303782	6,704376467	0,0091095	0,924782512
3915	ENSG00000136536	-0,172590265	6,891261014	0,6520086	0,427627678
3916	ENSG00000136546	-0,356068735	7,290856067	2,0656983	0,164046838
3917	ENSG00000136560	-0,096938392	5,351742234	0,345924	0,562133144
3918	ENSG00000136574	-0,031888787	5,782042401	0,0272539	0,870310575
3919	ENSG00000136603	0,80448256	6,717029955	5,0253356	0,034870103
3920	ENSG00000136628	-0,136214711	7,026544601	0,7518231	0,394812605
3921	ENSG00000136631	-0,040307204	5,904196978	0,0579587	0,81187453
3922	ENSG00000136636	-0,052932015	5,512830464	0,1192124	0,733012817
3923	ENSG00000136643	-0,005878747	5,163082452	0,0019792	0,964896088
3924	ENSG00000136682	-0,321374915	6,049205598	4,4381725	0,046201761
3925	ENSG00000136699	0,060552006	4,678514544	0,1169815	0,735422149
3926	ENSG00000136709	-0,093502563	6,038626716	0,9013889	0,352198161
3927	ENSG00000136710	0,326086841	3,882440532	7,3603387	0,063788632
3928	ENSG00000136715	0,070179071	3,923898737	0,1529586	0,739770867
3929	ENSG00000136718	-0,094545059	4,55956355	0,5504817	0,465568388
3930	ENSG00000136731	-0,01431311	6,410904671	0,0160229	0,900362593
3931	ENSG00000136732	0,4299407	5,554262736	4,9181864	0,03668086
3932	ENSG00000136738	0,286072738	5,1267148	5,7244467	0,025212683
3933	ENSG00000136754	0,26185198	5,169526696	4,1632261	0,052854002
3934	ENSG00000136758	-0,04076413	7,101400799	0,0885568	0,768669086
3935	ENSG00000136770	-0,024425126	4,439632724	0,0317804	0,893698247
3936	ENSG00000136783	-0,206830481	4,730704783	0,6230836	0,437926826
3937	ENSG00000136802	-0,123439853	4,548888904	0,3544907	0,557367364
3938	ENSG00000136807	-0,189311808	4,99945996	0,9757514	0,333478787
3939	ENSG00000136810	-0,184884717	5,243526144	0,8175247	0,375233119
3940	ENSG00000136811	0,30945684	4,464936581	4,7603989	0,039580972
3941	ENSG00000136813	-0,238492212	7,550820938	4,6128507	0,042409026
3942	ENSG00000136816	-0,022671655	3,721999062	0,0311303	0,895219155
3943	ENSG00000136819	0,11865396	5,005708632	0,8388772	0,369143213
3944	ENSG00000136824	-0,246095693	5,066596257	1,6980412	0,205377397
3945	ENSG00000136826	0,620015159	5,269828238	4,1370889	0,05358667
3946	ENSG00000136827	0,061115813	4,234531919	0,3171546	0,677389683
3947	ENSG00000136828	-0,041830972	4,296400579	0,0340498	0,855210356
3948	ENSG00000136830	0,005837214	5,214662501	0,0007781	0,977985667
3949	ENSG00000136842	-0,022896003	6,582470777	0,0107458	0,918331747
3950	ENSG00000136848	0,054039751	5,198882306	0,0651515	0,800789224
3951	ENSG00000136854	0,059007755	4,579120195	0,0787473	0,781498785

3952	ENSG00000136859	0,648901629	5,69115304	6,3349119	0,019221256
3953	ENSG00000136861	0,121046895	6,153111692	0,883808	0,356849344
3954	ENSG00000136868	-0,184044944	4,456011432	2,4939214	0,19461927
3955	ENSG00000136869	-0,120185774	6,244338412	0,304538	0,586348162
3956	ENSG00000136870	-0,409620532	5,515636132	4,4095414	0,046852135
3957	ENSG00000136874	0,048493864	5,613729815	0,2515884	0,620686723
3958	ENSG00000136875	-0,074519588	4,142578866	0,5112346	0,627617504
3959	ENSG00000136877	-0,382498195	4,836549624	3,0100676	0,096066433
3960	ENSG00000136888	-0,224022468	5,989987464	1,4335336	0,24332449
3961	ENSG00000136891	0,12027196	4,890946045	0,5453265	0,467666512
3962	ENSG00000136895	0,220697469	4,414064347	1,2374907	0,277392192
3963	ENSG00000136897	-0,447119566	5,464200439	3,1034779	0,091349296
3964	ENSG00000136925	-0,067336159	4,455377354	0,1891306	0,66767487
3965	ENSG00000136930	-0,413367724	6,044771486	9,6936322	0,004857332
3966	ENSG00000136935	0,046276611	4,851441701	0,1445588	0,785072302
3967	ENSG00000136936	0,008906857	4,098122039	0,0048847	0,944879799
3968	ENSG00000136937	0,02327326	5,81074239	0,0506362	0,82393074
3969	ENSG00000136938	0,148328157	5,075625362	0,7888899	0,383586358
3970	ENSG00000136940	0,072802281	4,175937924	0,2629048	0,612986741
3971	ENSG00000136942	0,153602714	7,107790113	0,3788424	0,544237177
3972	ENSG00000136950	-0,104281674	4,481726705	0,8428176	0,462879094
3973	ENSG00000136960	0,013434471	4,307908717	0,0020066	0,964655516
3974	ENSG00000136986	0,088590541	5,065553695	0,5195097	0,47825329
3975	ENSG00000136997	0,112266013	4,67855038	0,1214284	0,730644552
3976	ENSG00000137033	0,109777032	6,158098064	0,1415925	0,710135056
3977	ENSG00000137038	-0,094221077	5,652663741	0,244053	0,625956373
3978	ENSG00000137040	-0,561257561	5,919522492	3,6907612	0,067132441
3979	ENSG00000137054	0,289755224	3,970839783	5,4472638	0,094000541
3980	ENSG00000137055	-0,145507147	5,303654134	1,3634312	0,254807457
3981	ENSG00000137070	0,047343976	4,744657734	0,0875694	0,769932658
3982	ENSG00000137073	0,059228726	5,108132455	0,3232089	0,66402876
3983	ENSG00000137074	-0,267588684	4,46559994	5,6699316	0,060163151
3984	ENSG00000137075	0,309774876	5,486893323	5,7482725	0,024941716
3985	ENSG00000137076	0,261317804	8,307635145	1,152747	0,294056809
3986	ENSG00000137094	-0,260578105	5,359304675	1,3734008	0,253167416
3987	ENSG00000137100	-0,052165517	4,787948852	0,1412243	0,710483154
3988	ENSG00000137103	0,467637249	4,188683943	4,1161298	0,054148529
3989	ENSG00000137106	-0,085187678	5,560404336	0,3096138	0,583265023
3990	ENSG00000137124	-0,1530452	4,278674853	0,5922875	0,449330284
3991	ENSG00000137145	-0,009412757	6,895673173	0,0038171	0,951266961
3992	ENSG00000137154	0,278932949	9,248183182	1,6366114	0,213467711
3993	ENSG00000137168	-0,92234235	5,388556916	22,873354	7,90874E-05
3994	ENSG00000137171	0,190912925	4,362094879	1,1174375	0,301394964
3995	ENSG00000137177	0,46696805	7,257545305	11,896621	0,002163201
3996	ENSG00000137185	-0,323750562	3,716952306	9,2440835	0,086300855
3997	ENSG00000137193	-0,833701345	5,175230974	5,6607555	0,025985249
3998	ENSG00000137198	-0,469273124	5,200759425	4,3603486	0,047993852
3999	ENSG00000137200	0,06372625	5,560510794	0,1500779	0,702005329
4000	ENSG00000137207	0,092795313	5,160155246	0,4901881	0,490807143
4001	ENSG00000137210	-0,267983535	5,628245899	1,6517074	0,211456511
4002	ENSG00000137216	-0,305131883	4,550031149	3,5465897	0,072312832
4003	ENSG00000137267	-0,461819838	4,637955586	2,8949939	0,102271319
4004	ENSG00000137275	0,092364829	4,745926111	0,2311428	0,635197445
4005	ENSG00000137288	-0,330516973	5,65860007	2,627901	0,118564651
4006	ENSG00000137312	-0,283153604	6,873370271	3,5724652	0,071317469
4007	ENSG00000137337	0,148270508	4,332595674	0,3284541	0,572105085
4008	ENSG00000137364	-0,266096058	4,580718738	2,2357906	0,148377363
4009	ENSG00000137393	-0,663715144	4,772392849	11,955157	0,002126699
4010	ENSG00000137409	-0,184703966	6,938800032	1,4545037	0,239990455

4011	ENSG00000137413	-0,257428453	4,740006941	1,8994156	0,181340407
4012	ENSG00000137414	0,202652087	5,570997413	1,3661621	0,254386636
4013	ENSG00000137449	0,056222437	5,147256131	0,1045217	0,749376337
4014	ENSG00000137462	-0,227180382	4,400850163	0,4092556	0,528638336
4015	ENSG00000137478	0,140099474	5,425230559	1,2021158	0,284153016
4016	ENSG00000137486	0,223729241	4,822254334	0,8326676	0,37092271
4017	ENSG00000137491	-0,304332185	4,99249935	1,491614	0,234278247
4018	ENSG00000137492	0,307965001	5,56602974	3,1094976	0,091054622
4019	ENSG00000137497	0,390908075	6,182082039	3,6277598	0,0693422
4020	ENSG00000137500	-0,002803551	5,098556546	0,0002897	0,986566024
4021	ENSG00000137501	0,380591962	4,703210084	2,8731158	0,103503083
4022	ENSG00000137502	0,098367479	5,345987217	0,5230168	0,476788201
4023	ENSG00000137504	-0,136959272	5,545091564	0,5561427	0,463336132
4024	ENSG00000137507	-0,151670072	5,102942355	0,3491496	0,560329408
4025	ENSG00000137509	-0,077559432	6,430359904	0,3161805	0,579306616
4026	ENSG00000137513	-0,03123001	4,058666714	0,0406342	0,883072175
4027	ENSG00000137522	-0,130531832	4,005816675	1,3179331	0,437675759
4028	ENSG00000137547	-0,514909096	5,709434723	13,516399	0,001239762
4029	ENSG00000137571	-0,566576655	4,547917057	3,0646374	0,093277366
4030	ENSG00000137573	3,14703767	7,24266234	8,2340692	0,008637435
4031	ENSG00000137574	-0,028777734	4,558905414	0,0926138	0,892949995
4032	ENSG00000137575	-0,508247766	7,300488637	6,252531	0,019931858
4033	ENSG00000137601	0,101996931	5,553104955	0,500915	0,48616454
4034	ENSG00000137628	0,465806928	5,274533521	4,9005174	0,036989469
4035	ENSG00000137642	0,182854283	5,713960893	0,5587742	0,462292551
4036	ENSG00000137692	-0,216077525	4,585339304	2,1077737	0,159973348
4037	ENSG00000137693	0,411269077	6,340467084	5,3417355	0,030081546
4038	ENSG00000137710	-0,08968329	6,76650607	0,3682185	0,549883461
4039	ENSG00000137713	0,208935333	5,29047762	0,7881464	0,383806887
4040	ENSG00000137714	-0,36280417	4,792420789	6,7985414	0,01569117
4041	ENSG00000137752	0,326237958	3,955760608	1,809774	0,1915867
4042	ENSG00000137760	0,047399192	4,060081094	0,0773926	0,783336024
4043	ENSG00000137764	0,052307861	4,011726335	0,1360206	0,761245048
4044	ENSG00000137770	-0,133003715	5,417277596	0,6819891	0,41734587
4045	ENSG00000137776	0,050668549	6,440455492	0,1436203	0,708155252
4046	ENSG00000137801	-1,263719383	8,132507759	7,5344903	0,011507258
4047	ENSG00000137802	0,197960076	3,984199944	0,6263118	0,436758149
4048	ENSG00000137806	-0,553510132	4,717053551	7,0631521	0,014030229
4049	ENSG00000137809	-0,405887537	4,895381603	3,1081392	0,091121022
4050	ENSG00000137814	-0,00627318	3,960918712	0,0030361	0,99697393
4051	ENSG00000137815	0,106935748	5,337853782	0,5880651	0,450910287
4052	ENSG00000137817	-0,157248487	4,941494792	1,1036458	0,304328173
4053	ENSG00000137818	0,101996956	7,983973393	0,2283208	0,637259912
4054	ENSG00000137821	-0,422724867	4,946354027	1,7447045	0,199473243
4055	ENSG00000137822	-0,151766846	3,612988641	0,9706249	0,404709889
4056	ENSG00000137824	0,090312417	4,101195454	0,5151726	0,62842527
4057	ENSG00000137831	0,23823971	7,087634006	1,5119655	0,231209998
4058	ENSG00000137834	1,049018917	4,455808738	11,386518	0,002603331
4059	ENSG00000137842	-0,298889442	3,735390901	8,2119578	0,107713251
4060	ENSG00000137845	-0,066108359	6,807918712	0,2294547	0,636413698
4061	ENSG00000137871	-0,075474803	5,303885413	0,2095945	0,65136034
4062	ENSG00000137872	0,195728021	4,852767708	1,8251878	0,189761569
4063	ENSG00000137876	-0,155022974	6,28765903	0,3252605	0,573966233
4064	ENSG00000137878	-1,049250619	5,705904885	9,0074298	0,006350036
4065	ENSG00000137936	0,053143593	4,087569312	0,0340654	0,855177627
4066	ENSG00000137941	0,300367904	5,50327321	3,2443383	0,084733068
4067	ENSG00000137942	-0,002337382	5,451308239	0,0002257	0,988142812
4068	ENSG00000137944	-0,130154341	4,200550523	0,3728457	0,547415264
4069	ENSG00000137947	0,043091103	4,355879324	0,0448454	0,834144819

4070	ENSG00000137955	-0,2486207	6,133971267	1,4224638	0,245099061
4071	ENSG00000137959	0,042853439	5,444197036	0,0157097	0,901339536
4072	ENSG00000137962	-0,250652412	6,907286893	4,0327241	0,056409576
4073	ENSG00000137965	0,061118217	4,421216071	0,0703838	0,793130612
4074	ENSG00000137970	-0,008816981	8,062738086	0,0008274	0,977298836
4075	ENSG00000137992	-0,132515619	5,930487755	0,6454946	0,429913702
4076	ENSG00000137996	-0,172435019	4,89096484	2,4784632	0,181490314
4077	ENSG00000138002	0,158860229	4,871042649	0,9889359	0,330300397
4078	ENSG00000138018	-0,2612323	5,252338449	2,4983702	0,127553488
4079	ENSG00000138029	-0,512490512	8,863025962	4,8223249	0,038390641
4080	ENSG00000138031	-0,702421163	3,948709853	5,6957174	0,025575545
4081	ENSG00000138032	-0,318455081	5,104545099	4,3439911	0,04838043
4082	ENSG00000138035	-0,322419478	5,039057822	6,0783346	0,021503148
4083	ENSG00000138036	0,263421929	4,494458083	2,9425977	0,099649628
4084	ENSG00000138050	-0,229289258	4,018706577	3,8406751	0,183696984
4085	ENSG00000138061	-0,202794825	5,789620971	0,4655501	0,501822959
4086	ENSG00000138069	-0,140178897	6,765773917	1,5591745	0,224243131
4087	ENSG00000138071	0,020665443	6,903411791	0,0276712	0,869325744
4088	ENSG00000138073	-0,000777076	5,053376689	3,104E-05	0,995602315
4089	ENSG00000138078	-0,030723201	7,13749038	0,0197874	0,889351192
4090	ENSG00000138081	0,108408683	5,294064642	0,4683303	0,50056133
4091	ENSG00000138085	-0,126967464	6,169845968	0,7604328	0,392143565
4092	ENSG00000138095	-0,092385768	7,553613692	0,354628	0,557275919
4093	ENSG00000138100	-0,251134379	6,253902538	1,060408	0,313778446
4094	ENSG00000138107	-0,233769249	6,381350012	2,7983282	0,107799312
4095	ENSG00000138119	0,362268085	5,987031869	2,0742796	0,163209954
4096	ENSG00000138134	-0,358580268	4,490973017	2,6118025	0,119640248
4097	ENSG00000138138	-0,531134495	7,233785488	3,3237628	0,081246771
4098	ENSG00000138162	0,25508045	7,624680981	0,5341869	0,472196731
4099	ENSG00000138166	0,408082862	3,896409613	1,4899658	0,234528986
4100	ENSG00000138175	-0,642240016	4,840466902	14,699951	0,000842965
4101	ENSG00000138182	0,079177432	3,677376515	0,1488424	0,703172281
4102	ENSG00000138185	-0,232100315	5,019981241	2,2828075	0,144367086
4103	ENSG00000138190	-0,200887638	4,846682325	1,0897529	0,307322045
4104	ENSG00000138193	0,594077783	7,106959845	6,5997476	0,017120647
4105	ENSG00000138246	-0,072355686	6,754578427	0,5289945	0,474308057
4106	ENSG00000138279	-0,040598461	6,664106018	0,1231487	0,72881169
4107	ENSG00000138286	0,054048531	4,467039089	0,3078274	0,687587658
4108	ENSG00000138303	-0,350141871	4,891042812	6,5258522	0,017658038
4109	ENSG00000138326	-0,083012143	9,001426797	0,1130018	0,739784957
4110	ENSG00000138336	-0,085711848	4,315724058	0,2350706	0,6323527
4111	ENSG00000138347	0,480075571	7,507371527	2,1554912	0,155539472
4112	ENSG00000138356	-0,437921123	5,940488837	0,6154656	0,440704431
4113	ENSG00000138363	-0,275792551	4,393496374	1,8423544	0,187782396
4114	ENSG00000138375	-0,122829366	3,783626341	0,8624195	0,489209648
4115	ENSG00000138376	0,14132794	4,550618099	0,3987463	0,533933452
4116	ENSG00000138378	0,612405784	6,397922924	1,9549786	0,175325437
4117	ENSG00000138380	-0,015752109	4,436130121	0,0137097	0,969013785
4118	ENSG00000138381	-0,422685695	4,609215691	4,1970443	0,052015533
4119	ENSG00000138382	-0,373892377	4,592329354	3,2786728	0,083205257
4120	ENSG00000138385	-0,090817574	5,659246077	0,3722402	0,547736235
4121	ENSG00000138386	0,256110229	6,377565851	1,7641419	0,197076052
4122	ENSG00000138398	-0,101600548	6,466589499	0,256222	0,617521968
4123	ENSG00000138399	-0,531836065	5,610991589	3,3294071	0,081005369
4124	ENSG00000138411	-0,075818473	5,713739423	0,181778	0,673794373
4125	ENSG00000138413	0,098837402	5,776508297	0,0765035	0,784551346
4126	ENSG00000138430	-0,309928147	5,638711463	3,4745046	0,075073572
4127	ENSG00000138433	0,015916507	4,853112122	0,012278	0,912725206
4128	ENSG00000138434	0,066391786	6,781810114	0,2524957	0,620060919

4129	ENSG00000138442	-0,499552544	4,871997245	14,706095	0,000837229
4130	ENSG00000138443	0,069703164	6,598075411	0,2230534	0,641145733
4131	ENSG00000138448	-0,259619558	8,339911794	1,2019878	0,28421704
4132	ENSG00000138449	0,401158583	7,023789221	1,9898347	0,171676031
4133	ENSG00000138459	0,020985027	4,783152159	0,0148796	0,903967555
4134	ENSG00000138463	0,042822101	4,424121397	0,0992531	0,80881247
4135	ENSG00000138468	-0,142361296	5,575761902	0,7215051	0,40436338
4136	ENSG00000138495	-0,870722061	5,711226668	17,330014	0,000371966
4137	ENSG00000138496	0,174897536	5,306072299	0,9192398	0,347597429
4138	ENSG00000138592	-0,16959105	5,96405752	1,8166354	0,190732547
4139	ENSG00000138593	0,11006653	6,508305518	1,2167729	0,281308937
4140	ENSG00000138594	0,319281552	5,669028419	2,1861211	0,15275961
4141	ENSG00000138600	-0,126264209	6,459612471	0,8160903	0,37561633
4142	ENSG00000138604	-0,092274354	4,528425651	0,3203663	0,576842295
4143	ENSG00000138613	0,142520365	4,229915443	1,6121582	0,353073629
4144	ENSG00000138614	0,055302485	4,47067808	0,4141057	0,679653742
4145	ENSG00000138615	-1,111178881	5,893376182	9,8765403	0,004543284
4146	ENSG00000138629	-0,145143311	4,464949303	0,8421769	0,368252551
4147	ENSG00000138639	-0,064672044	5,582606483	0,1993881	0,659368043
4148	ENSG00000138640	0,220054352	5,7533601	1,1026764	0,304535783
4149	ENSG00000138642	-0,09446354	4,364777166	0,3318145	0,570159717
4150	ENSG00000138658	-0,357459178	3,523750726	4,494345	0,075431587
4151	ENSG00000138660	-0,180905392	4,169425364	1,0784095	0,309796188
4152	ENSG00000138663	-0,457623877	5,978578352	3,0189342	0,095606756
4153	ENSG00000138668	0,006506935	6,334387471	0,0034957	0,953359183
4154	ENSG00000138669	-0,377800518	4,247197087	0,7635367	0,391212846
4155	ENSG00000138670	0,352986814	4,425470463	2,5354108	0,124903451
4156	ENSG00000138674	0,415714262	7,391192335	19,387911	0,000202681
4157	ENSG00000138678	-0,074492417	5,703816522	0,1520345	0,700168698
4158	ENSG00000138685	0,437186765	4,883758762	1,9621467	0,174567289
4159	ENSG00000138686	0,220286458	3,797583774	2,8343876	0,214825745
4160	ENSG00000138688	-0,012999743	8,10600297	0,0107019	0,918495071
4161	ENSG00000138698	0,085088103	4,512193169	0,3580945	0,555370576
4162	ENSG00000138709	0,049525852	4,889553582	0,1017024	0,752660396
4163	ENSG00000138735	0,305338048	6,12863812	1,0498406	0,316148725
4164	ENSG00000138738	0,510136094	4,764666483	5,8836107	0,023493378
4165	ENSG00000138744	0,272231388	3,760534894	3,9918171	0,147461389
4166	ENSG00000138750	0,006708849	4,862363325	0,0020596	0,964191764
4167	ENSG00000138756	-0,117281094	5,37251884	0,5540646	0,464162963
4168	ENSG00000138757	-0,036967626	6,615908437	0,1117284	0,741188465
4169	ENSG00000138758	0,493401001	6,953504293	3,9215241	0,05969693
4170	ENSG00000138759	2,21990901	5,433674962	11,219519	0,002764691
4171	ENSG00000138760	0,024466194	7,47610832	0,0474775	0,829417822
4172	ENSG00000138764	0,461350076	4,677225765	2,6496054	0,117132441
4173	ENSG00000138767	0,055680094	5,872141919	0,1530853	0,699179305
4174	ENSG00000138768	-0,077140507	7,462138754	0,2539814	0,619043051
4175	ENSG00000138771	0,377282552	4,05553107	2,6164163	0,119330818
4176	ENSG00000138772	-0,511438693	5,165116722	4,8413317	0,038044646
4177	ENSG00000138777	-0,420088673	6,161336578	3,5170369	0,073430348
4178	ENSG00000138785	-0,30744752	3,788265939	5,1297215	0,102493987
4179	ENSG00000138792	-0,814589782	4,510912979	13,037885	0,001462155
4180	ENSG00000138796	-0,175138134	6,773993184	1,2868541	0,268257203
4181	ENSG00000138798	-0,967709673	4,539816407	8,0669332	0,009243078
4182	ENSG00000138801	0,423097701	4,533774146	3,5778395	0,071152257
4183	ENSG00000138802	-0,06223137	5,823823784	0,2923834	0,59385266
4184	ENSG00000138814	0,097281731	5,328311803	0,242078	0,62735002
4185	ENSG00000138821	1,575722222	6,695116037	8,3050755	0,008393487
4186	ENSG00000138829	-1,473761381	4,658411329	13,087141	0,00143789
4187	ENSG00000138834	0,250175565	5,290678492	1,4194603	0,245583393

4188	ENSG00000138835	-0,05602516	6,34139033	0,0889787	0,768144718
4189	ENSG00000138867	-0,201818769	6,007314612	1,6347768	0,213733773
4190	ENSG00000138942	0,030376124	4,67926164	0,0596175	0,872038161
4191	ENSG00000139044	0,648724596	5,010226549	5,6375033	0,026261784
4192	ENSG00000139083	-0,302636994	4,431211005	2,8768211	0,10329326
4193	ENSG00000139112	-0,110609158	7,870945746	0,5405939	0,469556684
4194	ENSG00000139116	-0,399039717	6,826688511	2,9753384	0,097891807
4195	ENSG00000139117	-0,068984726	5,235579022	0,1230297	0,728948297
4196	ENSG00000139132	-0,182601818	5,998497008	1,4836259	0,235496683
4197	ENSG00000139146	-0,07303196	3,882933233	0,2810126	0,691381857
4198	ENSG00000139154	0,08765466	5,169473576	0,3362173	0,567630805
4199	ENSG00000139163	0,003353649	5,990758175	0,0002483	0,987563774
4200	ENSG00000139168	-0,282029111	5,182605886	2,1542727	0,155651309
4201	ENSG00000139173	-0,141471218	3,882176351	0,6049582	0,444581743
4202	ENSG00000139174	0,684553299	3,79929132	17,433594	0,00036061
4203	ENSG00000139178	0,041110245	4,474013093	0,0192763	0,890779867
4204	ENSG00000139180	-0,409095487	5,043070539	3,860721	0,061560839
4205	ENSG00000139197	-0,093790324	4,577740893	0,3160995	0,579373648
4206	ENSG00000139218	0,042376627	7,503201038	0,1430607	0,708696765
4207	ENSG00000139233	0,02028536	4,155229089	0,0187434	0,892289872
4208	ENSG00000139239	0,107145076	5,241305015	0,2390713	0,629485354
4209	ENSG00000139263	0,623256397	4,337557441	6,1978668	0,020419474
4210	ENSG00000139278	-0,022533784	4,550904306	0,0085653	0,927059611
4211	ENSG00000139289	0,867107259	6,1616802	8,8557001	0,006740079
4212	ENSG00000139291	-0,254546476	5,027319385	3,1171242	0,090668959
4213	ENSG00000139304	1,013073919	4,893146921	3,2502574	0,08446739
4214	ENSG00000139318	0,970895914	5,352380522	14,322533	0,000952927
4215	ENSG00000139323	0,076284744	4,096092916	0,3542193	0,578507933
4216	ENSG00000139324	-0,04350945	5,350942288	0,0449454	0,833962976
4217	ENSG00000139329	0,972277212	8,484772749	10,495648	0,003602727
4218	ENSG00000139350	-0,148239491	4,7185788	0,5448687	0,467851242
4219	ENSG00000139370	-0,008623289	4,429291714	0,0096598	0,95918775
4220	ENSG00000139372	-0,186351384	4,535502564	2,6473353	0,1998418
4221	ENSG00000139428	-0,198683462	4,276933768	1,7902941	0,210621378
4222	ENSG00000139433	-0,055726351	4,512432775	0,2694895	0,694587435
4223	ENSG00000139436	-0,037861788	4,683614268	0,0621161	0,805383575
4224	ENSG00000139437	-0,065466034	3,633870258	0,1519029	0,704934824
4225	ENSG00000139496	-0,149934373	5,862484954	0,7503193	0,395278326
4226	ENSG00000139505	-0,220647597	5,673247065	1,3254846	0,26138129
4227	ENSG00000139508	-0,062669339	4,78945252	0,2408467	0,679084547
4228	ENSG00000139514	-0,26475529	4,516825427	1,227492	0,279292166
4229	ENSG00000139517	0,318574519	4,506118283	6,3535215	0,019036818
4230	ENSG00000139531	-0,101832923	4,571993623	0,3913899	0,537698796
4231	ENSG00000139567	-0,156140935	4,518365274	0,6227507	0,438047637
4232	ENSG00000139613	0,319341054	5,791344563	2,5667966	0,122708694
4233	ENSG00000139620	-0,255004717	4,680647079	7,842296	0,049519706
4234	ENSG00000139624	0,059408683	4,602597982	0,1384305	0,713235893
4235	ENSG00000139631	0,156685998	3,819523213	0,523539	0,476595742
4236	ENSG00000139636	-0,011559805	4,344080841	0,0039824	0,950224531
4237	ENSG00000139641	0,436849446	5,973701623	2,8608026	0,104203924
4238	ENSG00000139644	0,106151128	8,069479494	0,5648689	0,459864067
4239	ENSG00000139645	0,198294425	4,719268923	0,6066386	0,44395803
4240	ENSG00000139651	0,282921365	4,507872038	3,2025226	0,086637643
4241	ENSG00000139668	-0,188103112	4,375215469	1,2727254	0,27083095
4242	ENSG00000139675	0,230439972	5,621830977	3,0780602	0,092559497
4243	ENSG00000139679	-0,275532393	5,358865756	0,9907869	0,329857555
4244	ENSG00000139684	-0,276976386	6,649438266	2,6200138	0,119090197
4245	ENSG00000139687	-0,089553269	5,904396951	0,4315739	0,517685882
4246	ENSG00000139697	0,042549007	6,312316961	0,1535585	0,698735028

4247	ENSG00000139726	0,008892109	5,080199347	0,0065175	0,936348606
4248	ENSG00000139746	-0,067250021	6,154707568	0,3429073	0,563810754
4249	ENSG00000139793	-0,17953107	6,936452324	1,2219766	0,280347558
4250	ENSG00000139826	-0,052026155	4,66113003	0,0813995	0,77795083
4251	ENSG00000139842	0,072686268	6,849437776	0,2529994	0,619718373
4252	ENSG00000139914	-0,536413544	4,921062242	3,2442	0,084739288
4253	ENSG00000139921	0,0116689	4,819028272	0,0033578	0,954289105
4254	ENSG00000139926	0,307210631	4,931706645	1,1139046	0,302142695
4255	ENSG00000139946	0,104004124	4,018385196	0,2214099	0,642377957
4256	ENSG00000139971	0,104522545	4,891656101	0,2290854	0,636699507
4257	ENSG00000139974	-0,005178735	3,728801028	0,0009286	0,981838126
4258	ENSG00000139977	-0,118335597	5,450782279	0,6464511	0,429574278
4259	ENSG00000139990	-0,000819128	5,923669273	3,831E-05	0,99511417
4260	ENSG00000139998	-0,642143253	4,931044114	4,8198967	0,038435097
4261	ENSG00000140022	-0,087301071	4,206310383	0,2019686	0,657322585
4262	ENSG00000140044	0,070960071	4,024367928	0,1398073	0,711880791
4263	ENSG00000140092	0,224343155	6,603053613	0,6665526	0,422591407
4264	ENSG00000140105	-0,460831998	6,569724242	6,679519	0,016538821
4265	ENSG00000140153	-0,034867829	4,457750928	0,1897739	0,805268784
4266	ENSG00000140157	-0,229640228	4,423486473	2,9399425	0,122938024
4267	ENSG00000140199	-0,04249602	5,295859687	0,091285	0,76524635
4268	ENSG00000140259	0,019341879	4,5951798	0,0184971	0,892992663
4269	ENSG00000140262	0,181780716	6,091377532	2,2139822	0,150235864
4270	ENSG00000140264	0,02213813	6,957948049	0,0109053	0,917730269
4271	ENSG00000140265	0,096046429	4,080155166	0,5425339	0,584775275
4272	ENSG00000140285	-0,830290405	6,038779894	5,9909806	0,022388741
4273	ENSG00000140299	0,003491602	6,906073597	0,0005371	0,981709603
4274	ENSG00000140307	-0,214830649	4,7235737	2,0173803	0,168855131
4275	ENSG00000140319	-0,217518066	6,962818932	2,7400252	0,111333096
4276	ENSG00000140332	-0,169736041	3,941140786	0,5047959	0,484508451
4277	ENSG00000140350	-0,017829586	6,15322999	0,0147905	0,904252186
4278	ENSG00000140367	0,383553902	5,624879969	5,711477	0,025393224
4279	ENSG00000140374	-0,174297748	7,259008618	0,7946143	0,381894789
4280	ENSG00000140382	0,076754346	5,00527864	0,4230448	0,583286594
4281	ENSG00000140386	-0,323199848	5,903543177	4,2448429	0,050800266
4282	ENSG00000140391	0,215241143	6,736005152	1,6809582	0,207593053
4283	ENSG00000140395	-0,010355772	4,888729907	0,0111316	0,945140285
4284	ENSG00000140396	0,049371928	6,332586722	0,1967282	0,661484807
4285	ENSG00000140400	0,164488841	5,309411537	0,5966829	0,447673874
4286	ENSG00000140403	0,132805535	6,971101359	0,2694756	0,60861918
4287	ENSG00000140406	-0,348703821	3,808869933	3,6154261	0,069784461
4288	ENSG00000140416	-0,297812795	11,77694269	1,1485778	0,294872452
4289	ENSG00000140443	0,133685735	6,201159059	0,2603665	0,614707173
4290	ENSG00000140450	0,530636278	5,414998504	4,3869918	0,04737164
4291	ENSG00000140455	0,084317716	4,578839706	0,4714257	0,499139495
4292	ENSG00000140463	0,333040507	4,16405648	3,9883859	0,057720932
4293	ENSG00000140464	0,174917889	4,242814182	0,7877384	0,383927956
4294	ENSG00000140471	0,134872424	3,981662052	0,6508106	0,428046705
4295	ENSG00000140474	-0,205497706	4,498816777	1,8574667	0,186024113
4296	ENSG00000140479	-0,399131768	3,959397374	2,9361822	0,099998351
4297	ENSG00000140497	0,074552146	4,853024024	0,2111916	0,650128366
4298	ENSG00000140521	-0,101077742	4,492726891	0,2510712	0,621060557
4299	ENSG00000140526	0,0082875	7,366933648	0,0020339	0,964416168
4300	ENSG00000140545	-0,015494073	7,526083255	0,0055895	0,94104652
4301	ENSG00000140553	0,219050058	4,465194789	1,2906328	0,267574281
4302	ENSG00000140564	-0,084694903	5,371707649	0,2095436	0,651399651
4303	ENSG00000140575	0,116855395	7,509225358	0,327522	0,572647061
4304	ENSG00000140577	-0,03820986	5,024954808	0,0340256	0,855261224
4305	ENSG00000140598	-0,060491023	4,598523601	0,2870866	0,692242499

4306	ENSG00000140612	0,075045584	6,057657901	0,1767363	0,678075975
4307	ENSG00000140632	0,082721423	5,68667572	0,2372855	0,630761522
4308	ENSG00000140650	-0,137231182	3,557547476	0,4711727	0,499277176
4309	ENSG00000140694	0,098760637	5,127759586	1,0495196	0,329926502
4310	ENSG00000140718	0,133508758	6,01494441	2,0095108	0,169610576
4311	ENSG00000140740	-0,429307538	8,7017134	3,1954261	0,086965743
4312	ENSG00000140743	0,507845789	4,019214105	10,894843	0,0031107
4313	ENSG00000140750	0,060459164	5,230213942	0,0767958	0,784150889
4314	ENSG00000140795	-0,219673073	8,799592726	0,5419773	0,469020949
4315	ENSG00000140829	0,226360777	5,023059315	1,3910028	0,250233995
4316	ENSG00000140836	-0,414383398	5,455075694	2,2836112	0,144299671
4317	ENSG00000140848	0,306062622	3,81973483	2,6804543	0,115131598
4318	ENSG00000140853	0,05296273	4,191976573	0,0396926	0,843825202
4319	ENSG00000140859	0,284822012	4,809251489	2,2344193	0,148496315
4320	ENSG00000140905	-0,140456418	3,94678249	0,5632258	0,460535956
4321	ENSG00000140937	2,339738732	5,225297224	10,695715	0,003346237
4322	ENSG00000140939	-0,092875335	4,634671005	0,3470205	0,561518711
4323	ENSG00000140941	0,054507856	6,53484093	0,2504559	0,621469881
4324	ENSG00000140943	-0,171147554	6,675315155	1,2071899	0,283203232
4325	ENSG00000140945	0,223652091	5,35379411	1,4036897	0,248146802
4326	ENSG00000140948	0,31490764	4,87609106	3,3806696	0,078850235
4327	ENSG00000140986	-0,634531364	5,737970059	4,4627111	0,045652448
4328	ENSG00000140988	0,08391482	8,234260845	0,2363768	0,631397385
4329	ENSG00000140990	-0,584027584	6,731358213	9,812568	0,004654901
4330	ENSG00000140992	0,056596228	5,364068805	0,1016899	0,752674996
4331	ENSG00000140995	0,041839435	4,741870979	0,0870718	0,830948371
4332	ENSG00000141002	0,238180001	5,379818939	1,8118102	0,191346184
4333	ENSG00000141026	-0,333459565	4,510951892	4,6718152	0,041255375
4334	ENSG00000141027	0,034380948	6,92110115	0,0720183	0,790792957
4335	ENSG00000141030	-0,424373828	5,571163299	7,4803645	0,011769755
4336	ENSG00000141034	-0,026682809	4,217285503	0,0448404	0,834147518
4337	ENSG00000141040	0,107161137	3,750481664	0,4879344	0,544687935
4338	ENSG00000141052	0,095660376	6,442213994	0,1644043	0,688862407
4339	ENSG00000141076	-0,094666531	4,480188506	0,4081929	0,529156481
4340	ENSG00000141084	0,443815458	4,084434948	4,9166664	0,036707294
4341	ENSG00000141127	-0,062785041	4,304348445	0,3253847	0,693269291
4342	ENSG00000141161	0,055798	7,608109291	0,042692	0,838115642
4343	ENSG00000141179	-0,102969686	3,9967332	0,9804588	0,537731874
4344	ENSG00000141198	0,081089179	3,609720015	0,3530146	0,653016003
4345	ENSG00000141219	-0,075602108	4,684095738	0,3617089	0,621615642
4346	ENSG00000141232	0,396028923	5,128660503	5,2397389	0,031539726
4347	ENSG00000141252	0,062289507	5,280850335	0,0701695	0,793438274
4348	ENSG00000141258	0,222338965	4,833210559	1,4044763	0,248018127
4349	ENSG00000141279	-0,03727617	6,782699835	0,0748535	0,786818451
4350	ENSG00000141295	0,315592461	4,133170466	1,6042095	0,217923594
4351	ENSG00000141298	-0,230608902	6,286739508	3,1890735	0,087214746
4352	ENSG00000141338	-0,288399521	8,015338228	2,4385011	0,131978644
4353	ENSG00000141367	-0,032927295	8,463085269	0,0822074	0,776873634
4354	ENSG00000141376	0,178451354	4,719158439	3,0165835	0,197563158
4355	ENSG00000141378	-0,488824896	3,797685136	12,306567	0,007476288
4356	ENSG00000141380	0,000372934	6,029590738	1,062E-05	0,997428077
4357	ENSG00000141385	0,011170659	6,416610451	0,0049305	0,944624641
4358	ENSG00000141424	-0,01170382	5,04474381	0,0084091	0,927722989
4359	ENSG00000141425	-0,321080624	6,102119183	3,044281	0,094306667
4360	ENSG00000141429	-0,045920331	6,268046048	0,0600902	0,808516905
4361	ENSG00000141431	-0,126654776	3,860517992	0,4636569	0,502685299
4362	ENSG00000141441	-0,201423469	4,76327368	1,0127781	0,324658505
4363	ENSG00000141446	-0,207459438	5,173834846	1,1137815	0,302168787
4364	ENSG00000141447	-0,345507096	6,1770962	7,732495	0,010579982

4365	ENSG00000141448	0,262941596	5,616910082	4,3227366	0,048847709
4366	ENSG00000141449	-0,398430683	5,330331791	2,3626378	0,137850085
4367	ENSG00000141452	-0,039573665	3,862701698	0,1305185	0,814755232
4368	ENSG00000141458	-0,067739438	4,601496978	0,2161502	0,646333227
4369	ENSG00000141469	0,039662191	4,2330909	0,0178893	0,894757296
4370	ENSG00000141480	-0,275225935	4,546572486	1,0990369	0,305316991
4371	ENSG00000141503	0,155893784	4,677675714	0,547638	0,466735477
4372	ENSG00000141504	-0,245709645	5,040669482	2,7728321	0,109356167
4373	ENSG00000141540	-0,301555848	4,175947078	1,5818666	0,221051479
4374	ENSG00000141543	0,014463568	4,860779197	0,013306	0,98864167
4375	ENSG00000141551	-0,072118001	5,304884098	0,1338027	0,71784803
4376	ENSG00000141552	-0,220186227	4,942614454	1,1092133	0,303139458
4377	ENSG00000141556	-0,083591363	5,208808885	0,2472954	0,623683732
4378	ENSG00000141562	-0,241094371	5,188449777	2,8744037	0,103395431
4379	ENSG00000141564	0,202590608	4,107978613	0,9644939	0,336226428
4380	ENSG00000141568	-0,234971231	4,497167787	1,4592135	0,239270747
4381	ENSG00000141580	0,056562779	5,308071702	0,2647006	0,705432709
4382	ENSG00000141627	0,028856549	5,549605125	0,1015471	0,752832783
4383	ENSG00000141639	0,255326764	5,289235935	0,6143636	0,441108543
4384	ENSG00000141644	0,178430214	5,086612608	1,6636077	0,209839447
4385	ENSG00000141646	0,12131088	6,395936654	1,0306412	0,320481929
4386	ENSG00000141664	-0,168141615	4,698739949	0,9892014	0,330236817
4387	ENSG00000141699	0,353056616	4,980437023	4,0598127	0,055691608
4388	ENSG00000141736	-0,142986676	6,200486309	0,3338677	0,568977605
4389	ENSG00000141753	-0,486283452	7,886305199	1,8401777	0,18803366
4390	ENSG00000141756	0,54800326	4,667989337	4,8571055	0,037760162
4391	ENSG00000141759	-0,230665132	4,889201953	3,2217414	0,085710416
4392	ENSG00000141867	-0,087758978	4,931191296	0,1063849	0,747232905
4393	ENSG00000141905	-0,030058491	6,005506349	0,0121726	0,913100375
4394	ENSG00000141959	0,264162773	5,138004544	1,6350061	0,213702721
4395	ENSG00000142002	-0,086159154	4,302790231	0,183746	0,672142182
4396	ENSG00000142065	0,219182387	4,015712703	1,9279386	0,178221724
4397	ENSG00000142082	0,299780489	4,338655092	2,3944961	0,135347412
4398	ENSG00000142089	-0,231458018	7,189725865	0,6171788	0,440077355
4399	ENSG00000142156	-0,169761733	7,410641096	0,7721844	0,388586503
4400	ENSG00000142166	0,19717021	5,85159003	1,1231754	0,30018587
4401	ENSG00000142168	-0,497493334	7,469974462	5,6112181	0,026578347
4402	ENSG00000142173	-0,098403152	7,886251231	0,154567	0,697811566
4403	ENSG00000142178	1,339696476	4,833836387	12,635997	0,001677727
4404	ENSG00000142186	0,085972961	5,029917879	0,3287325	0,571942539
4405	ENSG00000142188	-0,26989624	5,705948168	4,6001971	0,042669042
4406	ENSG00000142192	-0,119667002	8,547154746	0,6462328	0,429625331
4407	ENSG00000142197	0,086248646	3,795477364	0,2478484	0,694901283
4408	ENSG00000142207	0,235364148	4,847388624	1,4164013	0,246077948
4409	ENSG00000142208	0,12815028	5,497011712	0,359419	0,554661172
4410	ENSG00000142227	-0,409144509	4,647578743	3,0018079	0,096496962
4411	ENSG00000142230	0,062937532	5,483730347	0,38285	0,572230499
4412	ENSG00000142327	-0,04329926	4,815450086	0,0319322	0,859733686
4413	ENSG00000142507	-0,358772673	5,797736966	5,3032902	0,030622134
4414	ENSG00000142534	0,178315213	8,595290277	0,6587341	0,425287257
4415	ENSG00000142541	0,27142005	9,26214037	1,3764974	0,252648158
4416	ENSG00000142556	-0,030686873	4,243909805	0,0441174	0,931074145
4417	ENSG00000142599	0,094386152	6,785286155	0,2568677	0,617081535
4418	ENSG00000142657	-0,31496434	5,133945746	1,1955798	0,285472491
4419	ENSG00000142661	-0,038732311	7,605682798	0,015453	0,902144451
4420	ENSG00000142676	0,087274787	8,653153121	0,1355879	0,716058204
4421	ENSG00000142686	0,042739216	4,044528648	0,1305475	0,837849593
4422	ENSG00000142687	-0,034536447	6,259620759	0,0387543	0,845656977
4423	ENSG00000142733	-0,460646916	4,811861141	3,1359241	0,089773924

4424	ENSG00000142751	0,147031696	3,861126019	1,0193805	0,460591079
4425	ENSG00000142784	0,167533054	5,368502848	0,4854465	0,492914156
4426	ENSG00000142798	0,695132147	7,15420829	3,0253063	0,095277973
4427	ENSG00000142856	0,394858604	3,597377161	4,1560872	0,053083076
4428	ENSG00000142864	0,03353136	7,346680116	0,0464832	0,831187156
4429	ENSG00000142867	0,073732232	3,835216301	0,2243645	0,646798075
4430	ENSG00000142871	0,414003496	6,610440294	0,8298478	0,371719903
4431	ENSG00000142875	-0,032934061	6,044100505	0,0631815	0,80374998
4432	ENSG00000142892	-0,538352644	5,885818078	3,4100064	0,077646547
4433	ENSG00000142910	-0,434893379	6,027728532	4,5384767	0,044002305
4434	ENSG00000142937	0,237981953	8,446447092	1,0398226	0,318418438
4435	ENSG00000142949	1,180706894	5,319880765	2,9916752	0,097028174
4436	ENSG00000142961	0,00524141	4,416931814	0,0011207	0,973582125
4437	ENSG00000143013	0,1938352	5,414488169	1,0061213	0,326220237
4438	ENSG00000143033	-0,000493034	4,51719041	1,318E-05	0,997134767
4439	ENSG00000143061	-0,245817551	4,668003274	1,4429506	0,241827828
4440	ENSG00000143067	0,335382541	4,48480981	5,2783088	0,030953768
4441	ENSG00000143079	-0,024684105	4,75609185	0,0404316	0,898812389
4442	ENSG00000143093	0,108265688	4,539315378	0,5110252	0,538005693
4443	ENSG00000143106	-0,241428655	5,552901745	1,9782157	0,172882175
4444	ENSG00000143119	-0,279383446	4,343786018	1,0672284	0,312261496
4445	ENSG00000143126	0,19968153	4,577413476	0,2614325	0,613987818
4446	ENSG00000143147	0,126792717	3,574243048	0,3676137	0,550217067
4447	ENSG00000143149	0,261242739	6,180437928	2,9322568	0,100212423
4448	ENSG00000143153	-0,472886455	7,131792809	7,0471022	0,0141263
4449	ENSG00000143155	-0,137256844	5,344404698	0,4648069	0,502161186
4450	ENSG00000143157	0,053733939	5,325831973	0,158192	0,694464721
4451	ENSG00000143158	-0,527435825	6,112514668	7,1813561	0,01334455
4452	ENSG00000143162	-0,132028065	6,586664504	0,6386084	0,432351198
4453	ENSG00000143164	-0,229233229	7,742967961	2,20443	0,151079643
4454	ENSG00000143183	-0,16103118	5,663995248	0,7620321	0,391672481
4455	ENSG00000143190	0,054773037	5,497882168	0,1564117	0,696098428
4456	ENSG00000143195	0,369975502	4,422398397	2,5269139	0,125505563
4457	ENSG00000143196	0,92776801	5,871465671	12,498834	0,001759074
4458	ENSG00000143198	-0,12351598	7,572614802	0,5316185	0,473251622
4459	ENSG00000143207	-0,037127263	5,272622141	0,0869775	0,7706791
4460	ENSG00000143222	-0,079432971	5,496818259	0,2204853	0,643070092
4461	ENSG00000143226	-0,444049934	5,403595107	3,1220271	0,090444795
4462	ENSG00000143248	-0,415459144	8,589568598	2,8647264	0,103979992
4463	ENSG00000143252	-0,18489958	6,668957677	2,2073241	0,150823398
4464	ENSG00000143294	-0,161189708	4,161282411	0,7343216	0,400284022
4465	ENSG00000143314	-0,244539528	4,926369934	3,0319838	0,094896581
4466	ENSG00000143315	0,069635333	3,656864569	0,2474639	0,734494422
4467	ENSG00000143318	-0,891984646	4,631539491	3,1440023	0,0893866
4468	ENSG00000143321	-0,238148436	6,361896027	2,420352	0,133343463
4469	ENSG00000143322	0,033954951	5,699413999	0,0181219	0,894079594
4470	ENSG00000143324	0,521666235	6,668056764	3,0139332	0,095865712
4471	ENSG00000143337	0,240849782	6,379912731	5,8522376	0,023796702
4472	ENSG00000143341	0,602241603	6,751141858	2,9134338	0,101246369
4473	ENSG00000143344	0,033395424	5,811110142	0,0406709	0,841938968
4474	ENSG00000143353	-0,024875352	5,706876912	0,0074012	0,932183386
4475	ENSG00000143363	0,100765203	5,12348264	0,3498598	0,559933794
4476	ENSG00000143369	-0,104061638	4,698519731	0,1704033	0,683557942
4477	ENSG00000143374	0,054560381	4,289955413	0,0934064	0,762626203
4478	ENSG00000143376	-0,093549182	5,73164044	0,3274995	0,572660133
4479	ENSG00000143379	0,04245866	4,361160725	0,0656768	0,800005879
4480	ENSG00000143382	-0,98207936	5,013664269	7,7192664	0,010658699
4481	ENSG00000143384	0,412168186	7,823998148	3,2748469	0,08337392
4482	ENSG00000143387	0,468452162	4,913727666	2,8969853	0,102160053

4483	ENSG00000143390	0,080732689	4,664244574	0,4597553	0,644345701
4484	ENSG00000143393	0,179123707	5,244051642	1,0321463	0,320172737
4485	ENSG00000143398	0,102132701	5,490616787	0,3928231	0,536961358
4486	ENSG00000143401	0,112059245	5,736990695	0,2310612	0,63525687
4487	ENSG00000143409	0,227495166	4,934732659	1,199451	0,284713178
4488	ENSG00000143416	0,101896005	5,500251465	0,0746408	0,78712218
4489	ENSG00000143418	0,245803519	4,644478497	1,3979257	0,249092284
4490	ENSG00000143420	-0,009477041	6,781464655	0,0072802	0,932735814
4491	ENSG00000143429	-0,077158157	3,877049221	0,1194789	0,732726741
4492	ENSG00000143434	0,080777924	4,680365824	0,1051931	0,748601609
4493	ENSG00000143436	-0,164876824	3,634160144	2,1369515	0,400553093
4494	ENSG00000143437	0,149806731	5,758890109	1,233015	0,278201109
4495	ENSG00000143442	0,295930634	5,932122701	2,4701801	0,129614903
4496	ENSG00000143457	0,171937255	4,310188066	1,5310331	0,228342509
4497	ENSG00000143458	0,247522108	5,008243543	4,1159284	0,054112461
4498	ENSG00000143479	0,05130804	4,970877221	0,0511093	0,823131372
4499	ENSG00000143486	0,202349395	5,052491265	3,9413705	0,078169352
4500	ENSG00000143493	-0,266302226	4,073480405	6,6884448	0,103110033
4501	ENSG00000143499	1,634845956	4,304712523	30,20047	1,35372E-05
4502	ENSG00000143514	0,035508922	5,298795753	0,0714894	0,791543598
4503	ENSG00000143515	0,37690257	5,072240737	3,1703377	0,088137231
4504	ENSG00000143537	-0,364318705	5,66396402	2,3210444	0,141200826
4505	ENSG00000143543	-0,018151625	3,893156483	0,0147518	0,913619076
4506	ENSG00000143546	-0,73810636	6,048003591	1,5614888	0,223953686
4507	ENSG00000143549	-0,439907141	8,248088365	2,2672	0,145683789
4508	ENSG00000143553	-0,177162003	4,743682293	1,9090643	0,188513543
4509	ENSG00000143569	0,083687651	6,020747913	0,2343095	0,63290163
4510	ENSG00000143575	-0,365126595	6,272363965	5,4485349	0,028634956
4511	ENSG00000143612	-0,358362322	7,200866918	8,6867736	0,007188975
4512	ENSG00000143614	0,183437495	5,17779493	0,572529	0,456899863
4513	ENSG00000143621	-0,001021154	6,141608824	8,682E-05	0,992645197
4514	ENSG00000143622	-0,098501665	4,871266622	0,5051681	0,484331816
4515	ENSG00000143624	0,29591478	5,828236391	2,664063	0,11618969
4516	ENSG00000143632	0,085900123	9,725192976	0,0366654	0,84982015
4517	ENSG00000143641	-0,283266938	5,014077576	2,4464169	0,131383258
4518	ENSG00000143643	-0,056981111	3,915931897	0,2113748	0,757465764
4519	ENSG00000143653	-0,110339211	5,407244665	0,3758989	0,545792758
4520	ENSG00000143669	0,158789829	6,485344629	0,7696768	0,389345367
4521	ENSG00000143702	0,082516268	6,146346786	0,5945538	0,448447947
4522	ENSG00000143727	-0,130232372	5,560576992	0,7212069	0,404459011
4523	ENSG00000143740	0,366823465	4,512338956	2,6518869	0,116983072
4524	ENSG00000143742	-0,366272406	7,382345155	2,2013717	0,15139777
4525	ENSG00000143748	0,013569824	3,981983295	0,0143827	0,92651223
4526	ENSG00000143751	-0,012731564	4,646603585	0,0121384	0,968906779
4527	ENSG00000143753	-0,094364921	5,400959379	0,53518	0,471764411
4528	ENSG00000143756	-0,030280058	5,394645895	0,0343906	0,854496241
4529	ENSG00000143761	0,083322535	7,404250971	0,3905346	0,538118574
4530	ENSG00000143771	-0,087169675	5,245101978	0,3877186	0,539580387
4531	ENSG00000143772	-0,209912435	4,484167825	0,910248	0,349921141
4532	ENSG00000143774	0,144934864	5,933595598	0,5510414	0,465370208
4533	ENSG00000143776	-0,197074299	7,131460379	2,2522739	0,14690956
4534	ENSG00000143797	0,074071186	4,682453674	0,0906369	0,7660607
4535	ENSG00000143799	0,009353213	6,187879749	0,0061133	0,938349697
4536	ENSG00000143801	-0,124060366	3,874023394	0,750361	0,456134294
4537	ENSG00000143811	-0,006103539	4,374688153	0,0021414	0,963486758
4538	ENSG00000143815	-0,103830485	4,994284025	0,3938143	0,536452457
4539	ENSG00000143819	0,118944448	5,212090612	0,1083323	0,745014756
4540	ENSG00000143847	0,337994854	4,855786202	1,3627353	0,254966468
4541	ENSG00000143850	1,132702163	5,051454224	14,555977	0,000883185

4542	ENSG00000143862	0,005332445	4,164569652	0,0018063	0,985889832
4543	ENSG00000143867	-1,098616083	4,694290377	20,757093	0,000139284
4544	ENSG00000143870	-0,055047776	6,248913511	0,1636913	0,689487413
4545	ENSG00000143878	0,231935662	6,927572604	1,3632091	0,254886193
4546	ENSG00000143889	0,115906151	5,248438526	0,5748778	0,455989266
4547	ENSG00000143891	-0,098717701	4,14763016	0,576605	0,516861086
4548	ENSG00000143924	0,190779429	5,240195284	0,8990525	0,352845202
4549	ENSG00000143933	-0,296931259	8,650458691	1,5351154	0,227781541
4550	ENSG00000143947	0,216544522	8,591944929	0,7939362	0,382094602
4551	ENSG00000143951	0,167828684	4,7321235	1,5378746	0,227334241
4552	ENSG00000143952	0,038653207	5,540774017	0,0738884	0,788165936
4553	ENSG00000143970	0,131449382	5,742875062	0,9097172	0,350028036
4554	ENSG00000143971	-0,061218711	4,447283817	0,1065541	0,747039327
4555	ENSG00000143977	-0,363756816	4,777997597	3,8515425	0,061847914
4556	ENSG00000143995	-0,703178572	5,563737675	13,891708	0,001097978
4557	ENSG00000144021	-0,012962466	5,793120996	0,0195925	0,889889491
4558	ENSG00000144026	0,225141964	4,541267086	3,0260511	0,095193204
4559	ENSG00000144028	0,22098498	6,759257347	1,1740455	0,28974588
4560	ENSG00000144029	-0,440356051	5,680964883	11,233675	0,002741423
4561	ENSG00000144034	-0,584576613	4,041253231	7,56756	0,011350064
4562	ENSG00000144036	0,134088066	7,601272454	0,5529041	0,464625769
4563	ENSG00000144043	0,065440627	5,463980182	0,2144089	0,647652331
4564	ENSG00000144048	0,067370431	3,909439496	0,3318249	0,670157908
4565	ENSG00000144061	0,037545505	3,609173528	0,0568783	0,820618
4566	ENSG00000144063	-0,409097683	4,186009488	3,6251439	0,069435731
4567	ENSG00000144115	-0,236003139	4,411216743	1,1361766	0,297470291
4568	ENSG00000144118	-0,077208725	5,673426826	0,7964048	0,416185616
4569	ENSG00000144136	0,849310365	5,807065245	4,2096498	0,051691879
4570	ENSG00000144161	-0,038910449	4,050366543	0,1052886	0,811051215
4571	ENSG00000144199	-0,113421549	4,27279463	0,4990057	0,486997998
4572	ENSG00000144218	-0,010956547	4,530271441	0,0052033	0,943115902
4573	ENSG00000144224	0,006369035	6,720815253	0,0015399	0,969034873
4574	ENSG00000144228	0,02468669	5,163561732	0,0171527	0,896933745
4575	ENSG00000144231	0,090877498	4,447769089	0,5565628	0,556860582
4576	ENSG00000144233	0,151032946	4,287555402	1,3382284	0,350611367
4577	ENSG00000144283	-0,067848837	6,246272473	0,248565	0,622782567
4578	ENSG00000144306	-0,199704761	4,801112183	1,1399571	0,29668686
4579	ENSG00000144320	-0,206556212	5,753124715	1,8894944	0,182440792
4580	ENSG00000144331	1,297066913	4,656767986	12,667649	0,001659546
4581	ENSG00000144357	-0,224739974	7,827357607	1,3076611	0,264524821
4582	ENSG00000144366	-0,287505027	3,907202452	1,2096036	0,282734465
4583	ENSG00000144369	0,291211315	3,941115309	1,3141605	0,263372849
4584	ENSG00000144381	-0,124978129	7,456918312	0,681061	0,417631902
4585	ENSG00000144426	0,073265493	6,64005682	0,2129028	0,648814737
4586	ENSG00000144445	-0,019430217	5,605044951	0,0227368	0,881447146
4587	ENSG00000144451	0,007023877	4,455703388	0,0013286	0,971236466
4588	ENSG00000144455	0,206493528	4,510589554	1,8725806	0,184328738
4589	ENSG00000144468	-0,080560174	5,037238239	0,6186541	0,479481915
4590	ENSG00000144476	-0,673949376	4,941222654	7,3940302	0,012202256
4591	ENSG00000144524	-0,009740671	4,009287737	0,0041838	0,948983366
4592	ENSG00000144535	-0,022423938	4,175132096	0,0286241	0,867116737
4593	ENSG00000144560	0,472175492	4,309082842	6,5460861	0,017524795
4594	ENSG00000144566	0,00669858	5,530404012	0,0035425	0,953048608
4595	ENSG00000144567	0,35201628	5,132637903	4,5752095	0,04322655
4596	ENSG00000144579	0,185410954	5,623097043	1,0754936	0,310436555
4597	ENSG00000144580	-0,088440651	4,86207767	0,7650828	0,439482589
4598	ENSG00000144596	0,59178493	4,489503605	3,0589484	0,093563709
4599	ENSG00000144597	0,247362029	3,5879469	3,5854119	0,205271979
4600	ENSG00000144635	-0,161891856	5,637502479	1,8318132	0,188957766

4601	ENSG00000144642	0,45733228	6,748097194	5,5690622	0,027094975
4602	ENSG00000144655	1,111178955	4,883319438	9,2786676	0,005713167
4603	ENSG00000144659	-0,009589075	3,949396341	0,0049894	0,964226205
4604	ENSG00000144668	0,105802769	5,540340774	0,2783973	0,602782564
4605	ENSG00000144674	-0,112126543	8,633234314	0,2601346	0,614863934
4606	ENSG00000144677	-0,164563052	5,973457738	2,5977177	0,120543582
4607	ENSG00000144711	0,022484761	5,834670734	0,0099242	0,921504809
4608	ENSG00000144712	0,074768538	5,314229826	0,0683989	0,795998844
4609	ENSG00000144713	0,09544222	8,49035779	0,1693397	0,684490276
4610	ENSG00000144724	-0,504659714	6,655216729	7,9077204	0,009863813
4611	ENSG00000144730	-0,07766376	3,608437695	0,2420336	0,666140299
4612	ENSG00000144736	0,197009886	4,441494922	3,1113283	0,150566846
4613	ENSG00000144741	-0,664499253	4,850108768	21,072038	0,000127214
4614	ENSG00000144744	-0,458266863	5,948379747	3,5632202	0,071692524
4615	ENSG00000144746	-0,231176005	7,606203811	2,5352334	0,12486883
4616	ENSG00000144747	0,185083425	5,838708841	1,7175692	0,202846564
4617	ENSG00000144749	-0,130679303	6,406891182	0,3804051	0,543414734
4618	ENSG00000144791	0,167937622	4,277291714	0,8322895	0,371029448
4619	ENSG00000144792	0,004708897	3,404945109	0,0007865	0,977866813
4620	ENSG00000144802	0,535903324	5,762603171	1,5066263	0,232009993
4621	ENSG00000144810	1,126449981	6,098380449	20,767227	0,000138897
4622	ENSG00000144815	-0,205776851	5,722916609	1,0849067	0,308375769
4623	ENSG00000144824	0,462162512	7,060450079	5,4291847	0,028891184
4624	ENSG00000144827	-0,127329445	5,082744329	0,4255733	0,520604946
4625	ENSG00000144840	-0,046973143	4,535761555	0,0860626	0,771861754
4626	ENSG00000144848	-0,123275021	5,149801451	0,8382538	0,369321096
4627	ENSG00000144857	-0,028855822	3,915492733	0,0139457	0,907015143
4628	ENSG00000144867	-0,04462314	5,084563399	0,0882289	0,769094084
4629	ENSG00000144895	-0,245192041	6,693726095	1,1719471	0,290166847
4630	ENSG00000144908	-0,108115586	4,671134126	0,0569427	0,813498096
4631	ENSG00000144909	-0,284833428	4,436273802	3,3961532	0,078212291
4632	ENSG00000144935	-0,125575336	5,479737214	0,550739	0,465491274
4633	ENSG00000144959	-0,358367824	4,626941475	3,2484305	0,084549286
4634	ENSG00000145012	-0,046799629	8,846947028	0,1069202	0,746610768
4635	ENSG00000145022	0,293642533	3,965701849	5,0773448	0,101835238
4636	ENSG00000145041	0,018836412	4,770719344	0,0213534	0,987423991
4637	ENSG00000145050	-0,283151232	4,658710242	1,2290527	0,278994474
4638	ENSG00000145147	0,538166563	5,383175127	4,2298265	0,051178561
4639	ENSG00000145191	-0,097895255	5,30365358	0,7572378	0,39311153
4640	ENSG00000145216	0,0940186	3,897016322	0,5136049	0,584015879
4641	ENSG00000145220	-0,248531836	4,012236823	2,1459508	0,162090801
4642	ENSG00000145241	0,142637141	5,723290617	0,7015509	0,4108409
4643	ENSG00000145244	0,118525387	7,709627449	0,1441374	0,707668054
4644	ENSG00000145246	0,33094475	5,209931663	5,5114198	0,027796607
4645	ENSG00000145293	-0,051247533	4,277098584	0,1811691	0,792431941
4646	ENSG00000145332	0,031687127	4,834448417	0,0436635	0,836311516
4647	ENSG00000145337	-0,1032379	5,230425541	0,3737749	0,546920512
4648	ENSG00000145348	0,177345842	5,424775567	1,6062095	0,217613028
4649	ENSG00000145349	0,040286759	7,807151241	0,0383894	0,846375649
4650	ENSG00000145354	-0,260564046	4,245870095	1,7006806	0,205037694
4651	ENSG00000145362	-0,125335452	8,43948105	0,2891324	0,595917109
4652	ENSG00000145365	-0,072656302	3,94761873	0,2061944	0,673211639
4653	ENSG00000145375	0,023455514	4,421473761	0,0461187	0,893483811
4654	ENSG00000145388	0,001281998	4,952553977	9,52E-05	0,992298729
4655	ENSG00000145390	-0,140959695	7,12126072	0,5360759	0,471423387
4656	ENSG00000145391	0,283418966	7,224404097	7,4777778	0,011760589
4657	ENSG00000145414	0,345550121	3,626689547	1,8476723	0,1871703
4658	ENSG00000145416	-0,233819324	4,515282203	0,6605605	0,424655128
4659	ENSG00000145425	0,25153678	8,971468727	1,0244921	0,32193522

4660	ENSG00000145431	0,752512168	4,89657714	7,2517953	0,012953622
4661	ENSG00000145439	-0,217085432	5,128282661	3,214281	0,086051311
4662	ENSG00000145476	-0,131322102	5,401214305	0,3985711	0,534022548
4663	ENSG00000145494	-0,508277138	5,768962196	5,4292135	0,028890801
4664	ENSG00000145495	-0,105320015	7,628339264	0,6195544	0,439182445
4665	ENSG00000145555	0,300382983	4,156119355	1,3271859	0,261083783
4666	ENSG00000145592	0,196608343	8,110200535	0,7409741	0,398190958
4667	ENSG00000145623	-0,6732602	6,087823363	6,00398	0,022258971
4668	ENSG00000145632	-0,398620881	6,930029879	2,193553	0,152094136
4669	ENSG00000145675	-0,05016892	7,983286445	0,0335153	0,856338089
4670	ENSG00000145685	-0,005605039	5,174424078	0,0005775	0,981033324
4671	ENSG00000145687	0,167444781	5,849990578	2,3685042	0,137338064
4672	ENSG00000145703	-0,431065894	4,70440667	2,2988931	0,143024883
4673	ENSG00000145715	0,029934246	5,696816575	0,0542362	0,817892288
4674	ENSG00000145723	-0,316903599	4,302023161	2,2414739	0,147885598
4675	ENSG00000145725	-0,205801184	6,295948445	1,3751999	0,252865561
4676	ENSG00000145730	-0,151726707	11,26613065	0,3242626	0,574535755
4677	ENSG00000145734	-0,052457594	6,648417461	0,1733619	0,680968892
4678	ENSG00000145736	-0,062007293	5,154415631	0,1009733	0,753517835
4679	ENSG00000145740	0,252487014	5,368515164	3,1922057	0,087087291
4680	ENSG00000145741	0,038159909	7,445463025	0,0919069	0,764469283
4681	ENSG00000145743	-0,189071063	5,835219505	1,2824254	0,269060491
4682	ENSG00000145780	-0,273094967	6,249440585	1,9062365	0,180588589
4683	ENSG00000145781	-0,349447242	5,039969871	2,536422	0,124832024
4684	ENSG00000145782	-0,07279284	5,363096361	0,319319	0,577442661
4685	ENSG00000145817	0,096953748	5,239566337	0,2711821	0,607493309
4686	ENSG00000145819	-0,384891299	6,425745066	3,8804022	0,060950366
4687	ENSG00000145833	-0,103268335	5,650954233	0,9496625	0,339859969
4688	ENSG00000145860	0,038794378	5,410648205	0,0563996	0,814372254
4689	ENSG00000145868	0,12191071	5,912775096	1,4085004	0,247319586
4690	ENSG00000145901	0,324683792	5,928633006	3,3948489	0,078265804
4691	ENSG00000145907	0,098366592	7,265330575	0,7425339	0,397671968
4692	ENSG00000145916	-0,144593219	4,563548794	0,7442881	0,39715443
4693	ENSG00000145919	-0,015010821	5,04319666	0,0182301	0,952555128
4694	ENSG00000145934	0,76085863	5,29570032	3,4782326	0,074927871
4695	ENSG00000145979	-0,592957905	4,274620445	12,702931	0,001639533
4696	ENSG00000145982	-0,461693918	4,147768894	8,0446942	0,009327134
4697	ENSG00000145990	-0,677230823	6,853008449	8,9374985	0,00652663
4698	ENSG00000145996	-0,177346703	4,740574366	2,032888	0,183140656
4699	ENSG00000146006	-0,181632412	3,494840491	0,5292452	0,474229839
4700	ENSG00000146007	-0,130035862	5,316463483	0,5326791	0,472815542
4701	ENSG00000146021	0,500423548	6,080818492	4,6184542	0,042332941
4702	ENSG00000146066	-0,21662585	4,802312197	2,1937432	0,152049437
4703	ENSG00000146067	-0,020375045	4,301424917	0,0144957	0,905208343
4704	ENSG00000146072	0,382720855	4,187930312	1,250037	0,275032157
4705	ENSG00000146085	-0,270215309	5,927410214	2,2770129	0,144854276
4706	ENSG00000146112	-0,130567801	4,571482098	0,4062728	0,530131325
4707	ENSG00000146122	-0,227398606	4,347014458	0,5527355	0,464693102
4708	ENSG00000146147	-0,452496498	7,828427065	2,3034482	0,142647506
4709	ENSG00000146223	-0,12213428	6,452459703	1,4979179	0,233279756
4710	ENSG00000146243	0,093037352	3,508752438	0,5113691	0,63528246
4711	ENSG00000146247	-0,020636462	7,11999468	0,0168055	0,89797604
4712	ENSG00000146263	-0,037891554	3,759955303	0,0675171	0,855459759
4713	ENSG00000146278	0,216695902	7,048417787	2,0314283	0,16742242
4714	ENSG00000146281	0,019316201	4,59467045	0,0128348	0,910778456
4715	ENSG00000146282	-0,001804521	5,233145702	0,0002202	0,988288521
4716	ENSG00000146350	0,138850998	3,768646125	0,51737	0,47917589
4717	ENSG00000146373	0,181364466	5,527181394	0,702913	0,410393731
4718	ENSG00000146376	0,689345672	5,355693063	2,1261385	0,158260678

4719	ENSG00000146411	0,248540161	4,212240868	0,6598882	0,424887655
4720	ENSG00000146414	-0,11250108	6,009160087	0,6960235	0,412633584
4721	ENSG00000146416	-0,137646545	5,370673007	1,0302133	0,320582726
4722	ENSG00000146425	0,161265645	5,189928111	1,4310734	0,243675555
4723	ENSG00000146426	0,204838648	3,783958636	1,4695965	0,297449741
4724	ENSG00000146433	0,068204591	5,389333911	0,2953141	0,592020305
4725	ENSG00000146457	0,075880376	6,028721588	0,1574812	0,695126694
4726	ENSG00000146463	0,251964443	6,060994184	6,1978917	0,020390507
4727	ENSG00000146476	-0,302096706	4,755301628	1,8550112	0,186329584
4728	ENSG00000146530	-0,625308237	4,587658036	3,3778111	0,078968659
4729	ENSG00000146535	-0,165069968	5,309755024	0,5792901	0,454286585
4730	ENSG00000146556	0,109783068	4,388875799	0,3435861	0,563447664
4731	ENSG00000146574	0,089084521	5,596848826	0,2931688	0,593378406
4732	ENSG00000146587	-0,079451017	4,919759874	0,2710807	0,607560106
4733	ENSG00000146592	0,152788262	5,047272254	0,2610894	0,614219178
4734	ENSG00000146648	-0,470186316	5,876164159	2,4569386	0,130596773
4735	ENSG00000146674	1,24291824	6,524892052	9,7966108	0,00468321
4736	ENSG00000146676	0,163304254	6,002269528	1,5410083	0,226876096
4737	ENSG00000146701	-0,440524548	7,571171152	4,8758545	0,037425121
4738	ENSG00000146729	-0,304863299	7,640294329	1,9142002	0,179715627
4739	ENSG00000146731	-0,220837952	6,571011028	1,9734274	0,173382232
4740	ENSG00000146757	0,019925658	3,631950353	0,0132666	0,90929655
4741	ENSG00000146802	-0,109059705	5,752438477	0,5079365	0,483167171
4742	ENSG00000146809	-0,448619215	4,981708289	1,0921924	0,306793458
4743	ENSG00000146830	0,375530995	5,000400531	3,6149383	0,069802021
4744	ENSG00000146833	0,512796091	4,378621436	6,8740027	0,015210031
4745	ENSG00000146834	0,403239754	4,105412351	5,3997207	0,029286267
4746	ENSG00000146842	0,219080388	3,926819689	3,1497026	0,19878936
4747	ENSG00000146859	0,507926355	3,77998134	6,3785621	0,018856174
4748	ENSG00000146872	-0,201965423	5,332706604	5,0950045	0,048788423
4749	ENSG00000146909	-0,085748516	3,992494592	0,3912599	0,577858413
4750	ENSG00000146963	-0,09498417	5,042044484	0,352645	0,558387498
4751	ENSG00000146966	-0,140061392	3,9505915	0,2384785	0,629908281
4752	ENSG00000147010	-0,519532587	5,39475846	11,295148	0,002690297
4753	ENSG00000147027	0,322502574	6,041041052	3,7482346	0,065186438
4754	ENSG00000147044	0,376469721	4,747215342	5,7077605	0,025436089
4755	ENSG00000147050	-0,056174334	5,142601527	0,0903634	0,766403078
4756	ENSG00000147065	0,190555237	7,575051814	1,3026386	0,265411716
4757	ENSG00000147099	-0,063872598	4,497647735	0,205091	0,654853827
4758	ENSG00000147100	0,565354466	4,192907525	4,1226788	0,053972253
4759	ENSG00000147113	-0,655320422	4,952124649	11,247802	0,002736609
4760	ENSG00000147121	0,132357188	3,657085948	1,4463809	0,489319373
4761	ENSG00000147123	-0,530618856	5,912858118	6,9741678	0,01457214
4762	ENSG00000147124	-0,154566425	4,740236895	0,8789842	0,358174368
4763	ENSG00000147130	0,440763657	4,362827838	3,7219982	0,066066685
4764	ENSG00000147133	0,219260248	5,913483574	2,5204981	0,125919706
4765	ENSG00000147140	0,187248364	6,777430182	1,8269097	0,189528918
4766	ENSG00000147162	0,263154311	7,727272478	3,9121471	0,059937518
4767	ENSG00000147164	0,066624667	4,983321258	0,4002303	0,652091782
4768	ENSG00000147166	-0,815945818	5,912088423	8,3234528	0,008331594
4769	ENSG00000147180	-0,012471959	3,8494975	0,0026369	0,959487004
4770	ENSG00000147202	-0,037958589	5,558684728	0,0383829	0,846388578
4771	ENSG00000147224	-0,098077015	4,46653059	0,7556036	0,531052374
4772	ENSG00000147251	0,310005562	5,846499787	0,4705063	0,499577737
4773	ENSG00000147274	0,133279003	6,995191205	1,0912807	0,306953525
4774	ENSG00000147316	-0,125484372	4,75966294	1,9220301	0,340602213
4775	ENSG00000147324	-0,298743491	4,149314021	5,0758693	0,059613169
4776	ENSG00000147364	0,106573805	5,452693776	0,701982	0,410669598
4777	ENSG00000147400	0,08574609	4,792032217	0,1503696	0,701730675

4778	ENSG00000147403	0,38581953	9,074481004	3,7084191	0,066491644
4779	ENSG00000147408	-0,034182238	6,018913467	0,0395591	0,844084271
4780	ENSG00000147416	-0,123367359	6,164019209	1,3661207	0,25435247
4781	ENSG00000147419	-0,239397065	5,135139322	1,6737274	0,208539851
4782	ENSG00000147421	0,075158363	5,678869507	0,3392442	0,565885501
4783	ENSG00000147454	0,217128617	5,566406613	0,6366799	0,433037731
4784	ENSG00000147457	-0,138280829	4,926595017	1,1327585	0,29814303
4785	ENSG00000147459	-0,157198556	5,583051228	1,1183142	0,301209811
4786	ENSG00000147471	0,139715406	5,498468164	2,8146249	0,163423343
4787	ENSG00000147475	0,193330213	5,427481847	2,0197954	0,168570822
4788	ENSG00000147485	0,175565561	6,591482936	0,3609464	0,553827624
4789	ENSG00000147526	-0,129163085	7,69177699	0,4654286	0,501878247
4790	ENSG00000147535	0,155934813	3,994851808	0,858418	0,363755942
4791	ENSG00000147548	0,176114043	6,291856796	1,8352945	0,188553553
4792	ENSG00000147573	-0,206059423	6,462189856	0,5156537	0,479897883
4793	ENSG00000147592	-0,235893322	3,796996741	1,2997684	0,26593258
4794	ENSG00000147601	0,031589903	4,704913013	0,0795631	0,784657663
4795	ENSG00000147604	-0,016847744	9,206885098	0,0033579	0,954288873
4796	ENSG00000147649	0,064462874	6,746839278	0,286038	0,597861185
4797	ENSG00000147650	-0,212663441	5,010611986	2,5338818	0,124968779
4798	ENSG00000147654	-0,192325925	4,471282036	1,6119197	0,24254939
4799	ENSG00000147669	-0,351883973	5,139568597	2,274732	0,145046585
4800	ENSG00000147677	-0,033943684	6,952853939	0,0856556	0,772376788
4801	ENSG00000147679	-0,031698253	4,411174525	0,0650454	0,93029187
4802	ENSG00000147684	-0,64877475	7,074290226	14,244884	0,000977441
4803	ENSG00000147687	-0,213841655	4,328567134	1,2731895	0,270745886
4804	ENSG00000147789	-0,045090738	4,087489357	0,1428595	0,757093783
4805	ENSG00000147852	-0,299626502	6,696999561	2,7643507	0,109889476
4806	ENSG00000147853	-0,242887893	6,931918196	1,8828216	0,183185493
4807	ENSG00000147854	0,018665762	5,415546906	0,0265692	0,871929814
4808	ENSG00000147862	0,034540832	8,014848595	0,0923523	0,763917251
4809	ENSG00000147872	-0,335297005	5,930223301	0,8350284	0,37025719
4810	ENSG00000147874	0,024121975	4,719871471	0,0358379	0,851497395
4811	ENSG00000147894	-0,37482698	5,248378456	2,2040658	0,151158714
4812	ENSG00000147905	0,188192922	4,784754137	1,1936945	0,285843261
4813	ENSG00000147996	-0,233824963	5,965582711	3,803353	0,063337154
4814	ENSG00000148019	0,230816233	4,037237344	3,0357425	0,163287739
4815	ENSG00000148053	0,005311583	6,088910672	0,0004845	0,982627821
4816	ENSG00000148090	-0,138465248	4,709852099	1,0344853	0,319603782
4817	ENSG00000148110	0,115429544	5,103380674	0,833783	0,370575448
4818	ENSG00000148120	-0,228060769	6,594092004	1,5619502	0,223887443
4819	ENSG00000148143	0,2039092	5,548064674	1,1163009	0,301635257
4820	ENSG00000148153	0,020615541	4,400865169	0,0349778	0,887192054
4821	ENSG00000148154	-0,534837832	5,485901429	2,9620016	0,098603464
4822	ENSG00000148158	0,388546248	4,43319188	4,3561746	0,048092165
4823	ENSG00000148175	-0,345114607	7,605565492	7,7775504	0,010385093
4824	ENSG00000148180	0,453739946	10,4836083	2,1444886	0,156552844
4825	ENSG00000148187	-0,174828115	5,013305021	3,0756863	0,114906926
4826	ENSG00000148218	0,222525453	5,254067065	1,2777938	0,269903956
4827	ENSG00000148229	-0,229037777	4,420879394	3,5664987	0,1340446
4828	ENSG00000148248	0,143685967	6,40037109	1,1736323	0,289790109
4829	ENSG00000148290	-0,3815601	4,561966929	4,8609702	0,037690826
4830	ENSG00000148296	0,066064309	4,14821255	0,1514598	0,742409496
4831	ENSG00000148297	0,107134596	4,002003633	0,4538168	0,60760291
4832	ENSG00000148303	0,286848671	8,752343871	2,1187574	0,15892637
4833	ENSG00000148308	-0,287193222	4,395040143	2,4598417	0,130380756
4834	ENSG00000148334	-0,434212387	5,393157605	3,5279026	0,073017187
4835	ENSG00000148337	0,11025272	4,835310666	0,4360845	0,515546434
4836	ENSG00000148339	-0,316648938	4,180224797	1,5581412	0,224435039

4837	ENSG00000148341	0,136643466	4,474107827	0,6893711	0,414872697
4838	ENSG00000148343	-0,105850715	4,602465184	0,3081678	0,58413994
4839	ENSG00000148358	0,24300414	5,875156589	2,6650446	0,116121759
4840	ENSG00000148396	0,243344716	5,352020035	1,0347963	0,319565625
4841	ENSG00000148399	0,110542706	3,996579551	0,4028007	0,570958476
4842	ENSG00000148400	0,399257453	4,313035325	2,4494454	0,131156305
4843	ENSG00000148411	-0,137433308	5,010515986	0,8538942	0,364995193
4844	ENSG00000148429	0,147889932	4,543250674	2,4929201	0,254847912
4845	ENSG00000148444	-0,376472445	4,051281919	3,4689184	0,07529251
4846	ENSG00000148450	-0,25677435	5,826400693	2,1856644	0,152800618
4847	ENSG00000148468	-0,068725477	3,878740718	0,0932194	0,762856338
4848	ENSG00000148481	0,122869899	5,276529656	0,3962809	0,535189871
4849	ENSG00000148484	-0,126445893	5,592067562	0,9685366	0,335200871
4850	ENSG00000148488	0,399884194	4,131754755	4,6326898	0,042043342
4851	ENSG00000148498	0,034229273	5,702523055	0,1706078	0,709761759
4852	ENSG00000148516	-0,059282548	7,124279822	0,1072373	0,746259258
4853	ENSG00000148572	0,015707067	3,920387295	0,0099929	0,921234386
4854	ENSG00000148606	-0,021610014	4,444509781	0,0269845	0,870942136
4855	ENSG00000148634	0,097335652	6,566121398	0,34723	0,561401454
4856	ENSG00000148655	-1,070668923	4,946920219	30,684806	1,21572E-05
4857	ENSG00000148660	0,099067587	4,195611674	0,4235669	0,570380909
4858	ENSG00000148672	0,048144509	6,746059545	0,2740538	0,605591598
4859	ENSG00000148677	0,203392916	12,12992938	0,2081107	0,652509972
4860	ENSG00000148688	-0,239512256	4,514027087	2,3994465	0,134963387
4861	ENSG00000148690	-0,202499324	5,010896715	1,2411291	0,276705056
4862	ENSG00000148700	-0,17149328	7,474015851	1,0966283	0,305835468
4863	ENSG00000148719	0,007266286	4,837978628	0,0029445	0,957190351
4864	ENSG00000148730	-0,370242841	7,879368524	6,1317699	0,020997731
4865	ENSG00000148737	0,30735348	4,095786448	2,5679062	0,122631936
4866	ENSG00000148834	-0,053109185	6,491698112	0,11063	0,742425981
4867	ENSG00000148840	-0,359543697	4,410843621	1,9887976	0,171783267
4868	ENSG00000148841	0,033941899	5,493219305	0,0164566	0,899034374
4869	ENSG00000148842	0,396939262	3,981271627	5,7723024	0,024703002
4870	ENSG00000148843	-0,082933458	4,784650363	0,2754891	0,604671785
4871	ENSG00000148925	-0,043886622	5,134549343	0,0670831	0,797924943
4872	ENSG00000148926	0,932309014	4,502616336	11,572018	0,002436092
4873	ENSG00000148943	0,153552141	5,808133258	0,6383255	0,432451798
4874	ENSG00000149084	-0,279407554	6,166510521	2,1473036	0,15629282
4875	ENSG00000149089	-0,459153536	4,429656058	7,8748694	0,009997534
4876	ENSG00000149090	0,090187806	3,835663728	0,1368074	0,714843285
4877	ENSG00000149091	0,17349807	5,644470315	0,8258605	0,372851402
4878	ENSG00000149100	-0,158769572	6,453922136	0,8238063	0,373436283
4879	ENSG00000149115	0,1252763	5,331127551	0,2326072	0,634133338
4880	ENSG00000149131	0,252863137	7,482624177	0,9032675	0,351740271
4881	ENSG00000149136	0,058299874	5,260299607	0,1452148	0,706631167
4882	ENSG00000149177	0,335541453	4,543806786	2,1568849	0,155411671
4883	ENSG00000149182	0,197124659	5,68646232	1,7563675	0,19803056
4884	ENSG00000149187	0,14600094	6,443185351	0,8688457	0,360910436
4885	ENSG00000149196	-0,37897576	4,846644781	4,4279227	0,046433413
4886	ENSG00000149212	0,949246386	6,578638043	4,867917	0,037566554
4887	ENSG00000149218	0,248202788	5,395475516	2,649385	0,117146882
4888	ENSG00000149231	-0,163853189	5,509116754	1,254312	0,27423045
4889	ENSG00000149257	-0,080242345	5,893184491	0,0956611	0,759871023
4890	ENSG00000149262	-0,119670915	4,501356972	1,4118995	0,35933769
4891	ENSG00000149269	0,100521755	4,224182455	0,3028829	0,587360825
4892	ENSG00000149273	0,261493214	8,150963902	1,6076547	0,217446249
4893	ENSG00000149289	-0,102819062	5,536625016	0,8311911	0,371307257
4894	ENSG00000149292	0,180410463	3,618131766	0,9860512	0,340249994
4895	ENSG00000149294	-0,003119273	7,487735486	0,0001898	0,989125153

4896	ENSG00000149308	-0,041771592	5,202258744	0,0784662	0,781878586
4897	ENSG00000149311	0,066046128	7,491291964	0,1574213	0,695181616
4898	ENSG00000149313	-0,127929526	5,178677772	0,5032755	0,485160064
4899	ENSG00000149346	0,217951602	3,564161645	1,7842603	0,257937963
4900	ENSG00000149357	-0,084655639	5,323875689	0,2352255	0,632241151
4901	ENSG00000149428	-0,25395487	5,921972677	1,2038613	0,283851364
4902	ENSG00000149474	0,147618306	3,86286092	1,7124268	0,421908696
4903	ENSG00000149480	-0,011367542	5,040598098	0,0044076	0,94763837
4904	ENSG00000149483	-0,072022189	3,849452812	0,3168892	0,665599018
4905	ENSG00000149485	-0,274022379	5,732963833	2,2665987	0,145734804
4906	ENSG00000149499	0,143050503	4,156227234	0,3549732	0,55710131
4907	ENSG00000149532	0,105063293	5,308355608	0,39287	0,536937248
4908	ENSG00000149547	-0,35807108	5,979519196	8,8779395	0,006665367
4909	ENSG00000149557	-0,055132239	5,012914834	0,092844	0,763319099
4910	ENSG00000149564	-0,328279732	5,138733292	3,5498709	0,072189957
4911	ENSG00000149573	-0,217104217	3,868694034	0,8359177	0,370006944
4912	ENSG00000149577	0,037478068	4,94821349	0,020173	0,888285764
4913	ENSG00000149591	-0,341283749	7,489294907	1,1819012	0,288177153
4914	ENSG00000149596	-0,252506767	6,298652171	0,7535216	0,394287581
4915	ENSG00000149600	-0,375871719	4,034922952	6,0471321	0,02931859
4916	ENSG00000149639	0,098385737	4,987407867	0,0985274	0,756419539
4917	ENSG00000149657	-0,016119238	4,556038975	0,0127176	0,911181945
4918	ENSG00000149658	0,037450355	4,614315681	0,0766786	0,857133013
4919	ENSG00000149743	-0,393308375	4,235418867	4,3987715	0,047099448
4920	ENSG00000149792	0,112088429	4,581721977	0,6683133	0,495538137
4921	ENSG00000149806	0,283094923	6,700993993	2,0819462	0,162466625
4922	ENSG00000149809	0,658276062	4,665720667	7,1929966	0,013279057
4923	ENSG00000149823	0,333936469	4,731609214	2,9308001	0,100292001
4924	ENSG00000149930	0,21759136	5,143253814	1,7581962	0,19780553
4925	ENSG00000149932	0,118557877	5,060430949	0,4522831	0,507921016
4926	ENSG00000150054	0,146938632	4,404050451	0,8203087	0,374435223
4927	ENSG00000150093	-0,143146366	9,459381819	0,517914	0,478922427
4928	ENSG00000150316	-0,457496876	5,646787752	5,1414118	0,033020681
4929	ENSG00000150347	0,200482713	6,854602004	0,3965202	0,535067673
4930	ENSG00000150401	0,174336198	5,801369501	0,4104234	0,528055889
4931	ENSG00000150403	0,545336251	5,139032169	26,887747	2,87648E-05
4932	ENSG00000150457	0,221921787	4,857649577	1,7824309	0,194852945
4933	ENSG00000150459	-0,138544438	6,570115378	0,8617876	0,362819456
4934	ENSG00000150471	0,143879053	4,244029138	1,2474073	0,348737699
4935	ENSG00000150477	0,205214833	4,005441723	3,2231355	0,234858656
4936	ENSG00000150527	-0,045067693	5,507206077	0,1134911	0,73923337
4937	ENSG00000150540	0,594736068	5,173994372	2,7071787	0,113430603
4938	ENSG00000150593	0,430627211	5,669520297	4,2974957	0,0494986
4939	ENSG00000150672	-0,161432685	4,467355484	1,0159868	0,323909389
4940	ENSG00000150687	0,863559181	5,392766191	8,7841701	0,006933039
4941	ENSG00000150712	0,252181191	4,934955181	4,1358473	0,053578384
4942	ENSG00000150722	-0,353923021	3,890916551	0,8042542	0,379070875
4943	ENSG00000150753	-0,374516058	6,181263702	5,6303113	0,02634798
4944	ENSG00000150756	-0,278778284	3,937310475	4,0827357	0,119540677
4945	ENSG00000150760	0,371351062	6,027883682	9,05406	0,006219914
4946	ENSG00000150764	0,380178124	6,275058291	7,4728256	0,011784939
4947	ENSG00000150768	-0,512965815	6,70095785	4,7878262	0,039027714
4948	ENSG00000150776	-0,282811847	4,942102729	3,5015523	0,074023774
4949	ENSG00000150779	-0,412237583	5,320806733	3,9251489	0,059587856
4950	ENSG00000150787	-0,25194029	4,096159428	1,4826295	0,235649227
4951	ENSG00000150867	0,317745417	5,050220444	2,1392257	0,157040382
4952	ENSG00000150907	0,043624959	5,570262749	0,0401727	0,842896425
4953	ENSG00000150938	1,076574444	6,833773762	6,6538149	0,016723869
4954	ENSG00000150961	0,159916692	5,345564505	0,6044821	0,444758722

4955	ENSG00000150991	-0,255085434	11,5289559	1,1734469	0,289827282
4956	ENSG00000150995	0,054533298	6,469336908	0,0918863	0,764504343
4957	ENSG00000151065	-0,223874716	4,256622491	0,4516653	0,508208177
4958	ENSG00000151067	-0,138615412	6,67380692	0,2068803	0,653467069
4959	ENSG00000151090	0,202010365	4,635694229	1,7082603	0,20406604
4960	ENSG00000151092	-0,306723452	5,235920543	5,3195528	0,030358051
4961	ENSG00000151093	-0,416098586	3,708108347	5,2810857	0,033199278
4962	ENSG00000151116	-0,243401461	4,714318032	2,0663098	0,163987035
4963	ENSG00000151131	0,149689962	4,982846933	0,9534186	0,338960545
4964	ENSG00000151135	0,186044791	6,029388795	1,1489116	0,294842197
4965	ENSG00000151148	-0,052161977	5,78681429	0,0790495	0,781091324
4966	ENSG00000151150	-0,478701675	8,075410964	5,8736219	0,02359919
4967	ENSG00000151151	-0,035955118	3,769496636	0,02666	0,871718129
4968	ENSG00000151176	-0,075949109	4,914852274	0,118907	0,733341101
4969	ENSG00000151208	0,405950776	3,845651845	1,9140364	0,179733529
4970	ENSG00000151229	0,024107537	5,008779519	0,0150357	0,903467872
4971	ENSG00000151233	0,234802083	4,614809776	2,0831251	0,162352689
4972	ENSG00000151239	-0,046956877	5,445706587	0,0271867	0,870468925
4973	ENSG00000151240	-0,030438885	5,422873892	0,0270188	0,870865887
4974	ENSG00000151247	-0,202632658	5,870041436	0,7073513	0,408941842
4975	ENSG00000151276	0,188500408	5,867558979	1,4938966	0,23393154
4976	ENSG00000151292	0,369732645	5,625725253	2,4017208	0,134787395
4977	ENSG00000151304	-0,033090146	4,149002016	0,077019	0,883390286
4978	ENSG00000151320	0,051504032	8,246083803	0,0258061	0,873770593
4979	ENSG00000151327	0,336736675	5,714065885	3,6694	0,067872544
4980	ENSG00000151332	0,122775523	3,824715546	0,7826678	0,441505991
4981	ENSG00000151338	-0,1565472	4,855997812	0,6589199	0,425222883
4982	ENSG00000151348	0,07244145	5,867491998	0,4387696	0,514245668
4983	ENSG00000151353	0,210631982	4,029799729	2,4227652	0,209690325
4984	ENSG00000151366	-0,429355576	4,89097954	7,7968341	0,01032327
4985	ENSG00000151376	-0,20797532	4,685614976	2,1301088	0,157854435
4986	ENSG00000151413	-0,006267752	4,80616948	0,0011544	0,973188203
4987	ENSG00000151414	-0,293708019	8,49401076	0,7127205	0,40719579
4988	ENSG00000151422	0,100388366	6,205109086	0,2792619	0,60222337
4989	ENSG00000151445	0,08925506	4,24243612	0,695848	0,551313564
4990	ENSG00000151458	0,730422586	5,678528849	6,9142006	0,014950394
4991	ENSG00000151461	0,00073316	5,53187475	4,106E-05	0,994942191
4992	ENSG00000151465	-0,305676503	5,304437514	2,9624223	0,098580922
4993	ENSG00000151466	0,171930892	5,026146399	0,6028624	0,445361629
4994	ENSG00000151468	0,418389491	4,474446028	1,9444286	0,176448584
4995	ENSG00000151474	-0,012518972	4,632787391	0,0021597	0,963332396
4996	ENSG00000151491	0,09782667	6,591034907	0,3426774	0,563960202
4997	ENSG00000151498	0,163710607	4,890737556	1,0256144	0,321675959
4998	ENSG00000151500	-0,225895955	4,869305104	1,7098783	0,203859371
4999	ENSG00000151502	0,051002481	4,925526687	0,1385	0,796101943
5000	ENSG00000151503	0,138091688	3,922092096	0,7472301	0,454066006
5001	ENSG00000151532	-0,109436668	4,65556855	1,4536101	0,376465484
5002	ENSG00000151552	0,013300958	4,506409508	0,0133869	0,939972183
5003	ENSG00000151553	0,178643639	5,716157463	1,3528542	0,256647997
5004	ENSG00000151576	0,137211916	3,848874842	0,6387615	0,442529874
5005	ENSG00000151612	0,214293558	4,682046465	1,4833415	0,23554022
5006	ENSG00000151617	0,300429504	5,853170396	1,756218	0,198048966
5007	ENSG00000151623	-0,171537544	5,625872904	1,6993178	0,20516865
5008	ENSG00000151632	0,133095538	6,054797571	0,0751917	0,786358287
5009	ENSG00000151657	-0,20729941	3,932984486	3,7170259	0,237418419
5010	ENSG00000151665	-0,096716313	3,525272835	0,4086095	0,66114282
5011	ENSG00000151687	0,293125828	4,461483299	3,7439287	0,065321576
5012	ENSG00000151689	-0,22837356	4,710904962	2,5598667	0,123153344
5013	ENSG00000151690	-0,361184172	5,098100193	4,6261712	0,042175674

5014	ENSG00000151692	-0,043479677	4,350578402	0,0460823	0,831909022
5015	ENSG00000151693	0,273367874	4,479542633	1,1603817	0,29250171
5016	ENSG00000151694	0,047432013	5,189576946	0,0849336	0,773319137
5017	ENSG00000151702	-0,065235827	4,878159626	0,2197727	0,643594123
5018	ENSG00000151718	0,100965022	5,72773537	0,4316612	0,517664324
5019	ENSG00000151726	-0,060660651	8,364540239	0,0242033	0,877719557
5020	ENSG00000151729	-0,612600304	9,933287192	6,3234742	0,019303133
5021	ENSG00000151746	0,468632662	4,324696461	7,5130797	0,011610313
5022	ENSG00000151748	0,02917141	5,164236376	0,0597565	0,80903079
5023	ENSG00000151779	-0,081353991	6,986403746	0,3684293	0,549758009
5024	ENSG00000151789	-0,657705406	4,041971388	5,5614321	0,027189676
5025	ENSG00000151806	-0,155343933	5,174149671	1,328795	0,260765872
5026	ENSG00000151835	0,101009237	7,292502173	0,1792034	0,675971875
5027	ENSG00000151849	-0,04942093	3,760928234	0,1263576	0,811042515
5028	ENSG00000151883	0,343234418	5,045120412	3,5288561	0,072981057
5029	ENSG00000151892	-0,44853528	5,437833367	2,1249943	0,158367907
5030	ENSG00000151893	0,223296853	5,917173105	3,7722991	0,064347424
5031	ENSG00000151914	0,07676191	10,77699666	0,1227615	0,729220543
5032	ENSG00000151923	-0,091478524	5,918548396	0,5961319	0,447853874
5033	ENSG00000151929	-0,237205896	6,275394024	1,0524324	0,315565132
5034	ENSG00000151967	-0,244868465	4,390219609	0,803298	0,379349596
5035	ENSG00000152061	-0,082138248	8,453950072	0,0962568	0,759149025
5036	ENSG00000152078	-0,161952205	6,183622791	0,5348632	0,471919626
5037	ENSG00000152102	0,08607043	6,49647626	0,4896903	0,491025077
5038	ENSG00000152104	0,790530139	5,788551489	7,0997059	0,013814112
5039	ENSG00000152117	0,037322549	4,213959338	0,0318037	0,860013068
5040	ENSG00000152127	-0,093279095	7,281290014	0,1681285	0,685556247
5041	ENSG00000152133	0,037415514	4,171670908	0,0459181	0,832204051
5042	ENSG00000152137	-0,17928808	7,878338545	0,9584581	0,337712612
5043	ENSG00000152193	-0,245880134	3,853539714	1,4102063	0,247083422
5044	ENSG00000152217	0,544636441	4,846640495	2,8929519	0,102385561
5045	ENSG00000152219	-0,177101037	5,307196679	0,7611814	0,391932727
5046	ENSG00000152223	-0,15314182	6,368611359	0,8527875	0,365305833
5047	ENSG00000152234	-0,413860939	10,11720619	3,1133359	0,090831145
5048	ENSG00000152242	-0,261026748	5,96295566	2,1699485	0,154219878
5049	ENSG00000152256	-0,095962739	5,730629592	0,249056	0,62245746
5050	ENSG00000152270	0,313616352	5,977249101	0,8490003	0,366353682
5051	ENSG00000152291	0,092560418	7,061112697	0,3857767	0,5405937
5052	ENSG00000152332	0,218084351	6,734238446	1,3346548	0,259782869
5053	ENSG00000152377	-0,481363329	6,726011498	4,1774812	0,052522382
5054	ENSG00000152382	-0,332507024	3,964490161	6,8060979	0,055765282
5055	ENSG00000152402	-0,201487074	6,041519835	0,6610488	0,424486378
5056	ENSG00000152404	-0,032772402	5,067327768	0,0505948	0,824007729
5057	ENSG00000152409	0,250971622	5,683002515	1,6605252	0,210282466
5058	ENSG00000152413	0,167342802	6,23985243	0,1408478	0,710861684
5059	ENSG00000152443	0,020369773	4,540235414	0,0365927	0,883212717
5060	ENSG00000152454	-0,069040066	3,657616316	0,2530815	0,689123199
5061	ENSG00000152457	0,145868434	4,019602593	1,2544462	0,372535148
5062	ENSG00000152465	0,10300609	4,408039782	0,7909764	0,416861275
5063	ENSG00000152484	0,435343842	5,48911319	3,5737579	0,071302625
5064	ENSG00000152492	0,233136025	6,792020101	2,3841818	0,136132584
5065	ENSG00000152518	0,338861356	4,917651948	1,7222942	0,202282065
5066	ENSG00000152520	0,137955459	5,351216351	0,9886312	0,330346181
5067	ENSG00000152527	0,628519761	4,675148669	4,6266799	0,04216533
5068	ENSG00000152556	-0,175849837	7,944196268	0,6900565	0,414644205
5069	ENSG00000152558	0,089159443	6,759597545	0,2455645	0,624894615
5070	ENSG00000152580	0,496512288	5,46820874	1,5110271	0,231350352
5071	ENSG00000152582	0,38759394	3,512365551	4,4434158	0,057784011
5072	ENSG00000152583	-0,737120845	9,899873294	11,663371	0,002349797

5073	ENSG00000152601	-0,109473268	8,06988707	0,504482	0,484618218
5074	ENSG00000152620	-0,284113888	5,370053911	4,5495082	0,043728781
5075	ENSG00000152642	-0,273003044	8,181850387	0,9775532	0,33304192
5076	ENSG00000152661	-0,185633036	8,055382084	0,4305997	0,518174881
5077	ENSG00000152683	-0,118286963	5,049085967	0,7713383	0,388813954
5078	ENSG00000152684	-0,131928615	4,619366892	1,2343802	0,366432651
5079	ENSG00000152689	-0,240810897	6,057578587	1,0144807	0,324260709
5080	ENSG00000152700	-0,19089908	6,516004905	2,0832416	0,162295008
5081	ENSG00000152749	0,027092281	4,61969615	0,0231045	0,880504662
5082	ENSG00000152763	0,430826477	3,732668586	3,2146094	0,086082098
5083	ENSG00000152767	-0,037935542	4,886224351	0,0369228	0,849300591
5084	ENSG00000152778	-0,063432245	5,104317447	0,0879812	0,769408639
5085	ENSG00000152779	0,541554678	3,993703599	8,3088603	0,008380699
5086	ENSG00000152782	-0,074610882	3,888291289	0,102362	0,751887563
5087	ENSG00000152795	-0,009237717	7,288811494	0,0046679	0,946114691
5088	ENSG00000152818	0,037821226	8,343749977	0,0792492	0,78081381
5089	ENSG00000152894	-0,180760671	6,127712489	2,3108534	0,141989546
5090	ENSG00000152904	-0,23839588	4,048389667	1,8481037	0,195843332
5091	ENSG00000152926	0,1228778	5,460032494	0,164737	0,688565248
5092	ENSG00000152942	-0,190904065	4,983801117	1,3461401	0,257798768
5093	ENSG00000152944	-0,215505452	5,603046581	1,347648	0,257539743
5094	ENSG00000152952	0,13933725	6,347888921	0,1729107	0,681373343
5095	ENSG00000152990	0,193498481	5,681522994	3,188704	0,087231938
5096	ENSG00000153006	0,093504765	4,959128224	0,2884626	0,596340599
5097	ENSG00000153015	-0,145175068	4,780227685	1,2235022	0,330480647
5098	ENSG00000153029	0,467068371	4,306228702	9,2320546	0,005817422
5099	ENSG00000153037	-0,204565652	4,097868856	2,5187889	0,212049326
5100	ENSG00000153046	0,023300316	4,538091337	0,0494686	0,896221984
5101	ENSG00000153048	-0,015500946	4,048309457	0,0035691	0,952874822
5102	ENSG00000153066	-0,137747296	4,551260229	1,4888647	0,303077887
5103	ENSG00000153071	-0,115903544	6,306201197	0,2056109	0,654458172
5104	ENSG00000153094	0,564682753	3,652426824	11,455889	0,004007651
5105	ENSG00000153107	0,049704529	5,714190542	0,2838772	0,599239364
5106	ENSG00000153113	-0,488231929	9,011780734	5,1562755	0,0327919
5107	ENSG00000153130	-0,386532467	6,469966648	1,6749117	0,208384404
5108	ENSG00000153132	-0,766660414	6,940935934	3,2321999	0,085280918
5109	ENSG00000153140	-0,169189651	4,201928582	0,6414266	0,431351022
5110	ENSG00000153147	0,14417244	6,987390451	1,0775823	0,309940554
5111	ENSG00000153165	-0,014342648	5,812951823	0,0091933	0,924441191
5112	ENSG00000153179	0,052792417	6,474903877	0,1204749	0,731657295
5113	ENSG00000153187	0,050947872	8,427961265	0,1345357	0,717099883
5114	ENSG00000153201	-0,122841481	8,485611847	0,5200749	0,47801663
5115	ENSG00000153207	0,019137662	6,307524844	0,0263761	0,872391693
5116	ENSG00000153208	-0,421795282	4,226749743	2,1943978	0,152018714
5117	ENSG00000153214	0,233120603	4,673363342	1,12396	0,300021043
5118	ENSG00000153234	1,611869046	4,649105632	10,000282	0,004335628
5119	ENSG00000153246	0,639032088	4,773657381	4,8258358	0,038326466
5120	ENSG00000153250	-0,162072376	6,867473186	1,1744119	0,289656774
5121	ENSG00000153291	0,253356781	4,747729806	2,0306546	0,167518808
5122	ENSG00000153310	-0,168639113	5,129000569	0,8808581	0,357671898
5123	ENSG00000153317	0,045690418	5,795018352	0,074177	0,78776776
5124	ENSG00000153339	-0,091831109	6,149418361	0,3598082	0,554448532
5125	ENSG00000153395	-0,141323055	4,156286487	0,6816514	0,41745953
5126	ENSG00000153531	0,441605665	7,497487858	2,2015053	0,15138591
5127	ENSG00000153560	0,025113213	5,692990559	0,0550458	0,816563707
5128	ENSG00000153561	-0,181861913	7,29153777	1,1399742	0,296683321
5129	ENSG00000153707	0,539336866	4,348240448	1,7168099	0,202976914
5130	ENSG00000153721	-0,283688657	4,669614374	1,269576	0,271409057
5131	ENSG00000153774	-0,18467173	4,509828604	1,7680281	0,231003839

5132	ENSG00000153786	-0,102014472	5,267497479	0,5640281	0,460194248
5133	ENSG00000153814	-0,030401729	4,553637803	0,0423319	0,944995183
5134	ENSG00000153815	-0,297787589	4,000800801	1,1000654	0,305095948
5135	ENSG00000153820	-0,257791662	6,812885618	0,4150787	0,52574574
5136	ENSG00000153823	-0,329568323	4,268109372	1,4729518	0,237137459
5137	ENSG00000153827	0,045363375	7,879888387	0,22663	0,638487621
5138	ENSG00000153879	-0,261492695	5,118456004	4,2723715	0,05007436
5139	ENSG00000153904	0,409331577	4,661129208	4,5340569	0,044096699
5140	ENSG00000153914	-0,140005712	6,802400771	0,8414245	0,368462793
5141	ENSG00000153922	-0,039986885	6,405755941	0,0248857	0,876022407
5142	ENSG00000153933	0,077807007	4,392447304	0,2599424	0,614993938
5143	ENSG00000153936	0,202457506	4,907350274	1,1682902	0,290902415
5144	ENSG00000153944	0,160437893	6,318029852	0,9001514	0,352556672
5145	ENSG00000153956	0,108096241	6,817450969	0,3202308	0,576922348
5146	ENSG00000153989	0,033105757	5,28439311	0,0485321	0,827571855
5147	ENSG00000154001	-0,065032563	5,598302431	0,307304	0,584645394
5148	ENSG00000154059	0,158689395	5,046959299	1,0322127	0,320157514
5149	ENSG00000154114	-0,085717885	5,170348219	0,3012922	0,588337485
5150	ENSG00000154122	0,221570272	6,624054915	2,3669734	0,137473827
5151	ENSG00000154124	-0,040186829	5,015361181	0,0676179	0,797139746
5152	ENSG00000154133	-0,032215985	4,281246101	0,0189508	0,891699779
5153	ENSG00000154144	-0,019648938	4,632095103	0,0316145	0,860420196
5154	ENSG00000154153	-1,225853261	7,789797297	9,3173824	0,005628138
5155	ENSG00000154174	-0,190000229	6,595299252	2,0201513	0,168530533
5156	ENSG00000154175	0,121013082	6,484307842	0,2772382	0,603534027
5157	ENSG00000154188	0,423004577	4,398043226	0,9723659	0,334301794
5158	ENSG00000154217	-0,400764887	4,466815155	3,2479169	0,084572327
5159	ENSG00000154222	0,123651938	4,337305311	0,5592895	0,4921215
5160	ENSG00000154229	0,885453773	4,564906178	8,3781158	0,008150447
5161	ENSG00000154240	0,043960021	4,944518906	0,1383351	0,713318258
5162	ENSG00000154258	-0,611425217	6,953535225	6,8569522	0,015321665
5163	ENSG00000154262	-0,239653177	7,274926485	1,0685537	0,311967896
5164	ENSG00000154263	-0,71653237	6,245276892	6,4283841	0,018448848
5165	ENSG00000154265	-0,208117937	6,139436265	1,302271	0,265485156
5166	ENSG00000154277	1,344594151	4,281173488	16,903506	0,000423007
5167	ENSG00000154305	0,079326629	6,574337078	0,6109973	0,442319176
5168	ENSG00000154309	0,261986513	3,792156067	1,9150192	0,179626139
5169	ENSG00000154310	-0,115224987	5,387259822	0,1924463	0,66496171
5170	ENSG00000154328	-0,236553622	4,214662027	2,8857646	0,130332265
5171	ENSG00000154330	-0,1864809	7,805327381	0,7689609	0,389562432
5172	ENSG00000154358	-0,022259125	8,114462044	0,0038005	0,951372755
5173	ENSG00000154359	-0,096913681	4,576114052	0,5429578	0,517213652
5174	ENSG00000154380	0,464941415	7,105530117	11,730616	0,002294257
5175	ENSG00000154415	-0,841554281	7,370934538	2,8456027	0,105076709
5176	ENSG00000154429	0,052918182	3,892441277	0,2131981	0,71163486
5177	ENSG00000154447	0,240292802	4,26871577	1,9449364	0,176394324
5178	ENSG00000154473	-0,017749533	5,325777278	0,0219404	0,956181876
5179	ENSG00000154511	0,206401276	3,798173829	0,723374	0,403764632
5180	ENSG00000154518	-0,593627243	8,313219783	12,424913	0,00179777
5181	ENSG00000154529	-1,036141933	5,876587347	5,677109	0,025792705
5182	ENSG00000154553	-0,086094914	7,982637315	0,1090539	0,744198438
5183	ENSG00000154556	0,366744188	8,992814678	1,5053455	0,232202413
5184	ENSG00000154582	-0,464904158	5,382119909	7,4527133	0,011906404
5185	ENSG00000154608	0,064090288	4,414015927	0,4610645	0,646925446
5186	ENSG00000154639	0,856916747	5,435693267	4,7331768	0,040061364
5187	ENSG00000154642	0,018707774	4,744550442	0,0061825	0,93800459
5188	ENSG00000154655	-0,207364046	4,480183463	1,4481988	0,24099883
5189	ENSG00000154678	0,23911461	7,303193706	0,5760201	0,455547455
5190	ENSG00000154719	-0,581230876	4,786569982	6,526623	0,017673986

5191	ENSG00000154721	-0,165622082	5,564448264	1,0883786	0,307620362
5192	ENSG00000154723	-0,667367911	7,393564206	10,724036	0,003311576
5193	ENSG00000154727	0,037993835	5,571063737	0,0382871	0,846577772
5194	ENSG00000154734	-0,415452045	7,472873373	2,0928144	0,16141988
5195	ENSG00000154736	-0,26393372	5,000657805	0,7289508	0,401985892
5196	ENSG00000154743	-0,30168913	3,9213631	10,942266	0,079278533
5197	ENSG00000154767	0,628892373	4,202331902	15,886348	0,000578406
5198	ENSG00000154781	0,07343357	4,063812058	0,4034626	0,664999373
5199	ENSG00000154783	-0,014948059	4,624659961	0,0045289	0,946924124
5200	ENSG00000154803	-0,026238537	4,848096404	0,017856	0,894854756
5201	ENSG00000154813	-0,38544115	5,066806683	4,3036853	0,049348074
5202	ENSG00000154814	-0,215515935	4,644542558	3,4198212	0,095979496
5203	ENSG00000154822	-0,446292456	6,114386326	3,6523896	0,068468572
5204	ENSG00000154845	0,150594516	6,176829103	1,4503266	0,240621656
5205	ENSG00000154856	-0,45029966	4,724861818	2,2215454	0,149618606
5206	ENSG00000154874	-0,075094126	5,642917429	0,0583236	0,81129521
5207	ENSG00000154889	0,244878718	3,89520506	2,3433742	0,18501378
5208	ENSG00000154898	0,153674432	4,312512294	0,1843526	0,67163503
5209	ENSG00000154917	-0,348435663	4,876743881	2,5997408	0,120453655
5210	ENSG00000154930	-0,215442839	5,824769805	0,8964088	0,353540771
5211	ENSG00000154945	-0,045148418	5,585276141	0,0821513	0,77695651
5212	ENSG00000154978	0,293307346	4,942640551	4,0725596	0,05532107
5213	ENSG00000155008	-0,17084096	5,161220946	1,000666	0,327507832
5214	ENSG00000155016	0,33791391	4,715879179	7,821909	0,010197081
5215	ENSG00000155085	-0,32038779	3,999457547	5,3132625	0,056284333
5216	ENSG00000155090	0,291167268	5,982140702	0,6564602	0,426076332
5217	ENSG00000155096	0,196327664	7,059405966	0,9961223	0,328585654
5218	ENSG00000155097	-0,004440192	5,943478492	0,0009832	0,975253627
5219	ENSG00000155099	-0,327216437	4,528509493	3,7328619	0,065700566
5220	ENSG00000155100	-0,202786641	4,27643974	0,7018434	0,41074481
5221	ENSG00000155111	0,246236918	4,369532572	2,3533806	0,138587577
5222	ENSG00000155115	-0,560372694	5,256045244	11,476821	0,002520397
5223	ENSG00000155158	0,477013154	4,165334449	4,5651525	0,0434374
5224	ENSG00000155189	0,048634279	4,009259043	0,0903837	0,766377609
5225	ENSG00000155229	0,041339624	5,420805307	0,1006493	0,753890047
5226	ENSG00000155252	0,131341968	3,923124716	0,7739845	0,465334464
5227	ENSG00000155256	0,218948524	4,199486307	2,1553708	0,20121304
5228	ENSG00000155287	-0,209055972	4,414686328	1,9810075	0,172563917
5229	ENSG00000155304	-0,191165409	5,605144949	0,4224774	0,522111962
5230	ENSG00000155313	0,022761062	6,601556932	0,0254642	0,874598395
5231	ENSG00000155324	-0,127437247	4,284655917	0,6951174	0,412963024
5232	ENSG00000155329	-0,24584022	4,073805444	1,1000381	0,30510181
5233	ENSG00000155330	-0,266252842	3,867242316	2,5222631	0,162019119
5234	ENSG00000155363	-0,143373663	4,094077659	0,5295118	0,474119782
5235	ENSG00000155366	-0,145079738	5,803271649	0,6295111	0,435604762
5236	ENSG00000155368	-0,171352828	6,674310494	0,687069	0,415641549
5237	ENSG00000155380	0,065571153	7,061110733	0,0638343	0,802768699
5238	ENSG00000155393	-0,297759354	4,149163488	6,1813053	0,057470403
5239	ENSG00000155438	0,016486049	4,010493543	0,0062511	0,93766234
5240	ENSG00000155463	-0,075938522	6,258882384	0,3952263	0,535707656
5241	ENSG00000155506	0,126801849	6,769514323	0,2896743	0,595574948
5242	ENSG00000155508	-0,069987178	5,019749303	0,3452681	0,594275361
5243	ENSG00000155545	-0,011817398	5,051771803	0,0039525	0,950411436
5244	ENSG00000155561	-0,195109836	5,472663076	3,0517421	0,093881532
5245	ENSG00000155592	-0,106539273	3,692507997	0,4091573	0,538040046
5246	ENSG00000155629	-0,351145393	3,953325104	2,3888406	0,135787723
5247	ENSG00000155636	-0,142949647	3,701359429	1,0422114	0,463860454
5248	ENSG00000155657	-0,39118869	15,66723424	0,640484	0,431685143
5249	ENSG00000155659	-1,444545331	5,445675459	21,10256	0,000126741

5250	ENSG00000155660	-0,343873598	5,702655633	1,3936036	0,249804293
5251	ENSG00000155729	0,22855222	3,977931707	3,236297	0,179139754
5252	ENSG00000155744	-0,090390463	5,289263939	0,3138627	0,580709684
5253	ENSG00000155754	-0,121803179	3,938080354	0,2065055	0,653759318
5254	ENSG00000155755	0,16721629	4,734393827	1,3735959	0,253110216
5255	ENSG00000155760	0,963932598	4,573220482	48,486043	4,06211E-07
5256	ENSG00000155827	0,047228369	5,715778169	0,180512	0,674849133
5257	ENSG00000155846	-0,151256477	4,884129556	0,3931203	0,536808659
5258	ENSG00000155849	0,070896063	4,771156897	0,1609819	0,691939476
5259	ENSG00000155850	0,030130633	4,773667023	0,0421037	0,839218154
5260	ENSG00000155868	-0,254295018	3,998755254	2,3495664	0,16118812
5261	ENSG00000155876	-0,042117357	5,718455282	0,1589019	0,693815585
5262	ENSG00000155893	0,173332405	3,915137712	0,7507304	0,395150919
5263	ENSG00000155903	0,078461121	4,775235148	0,210505	0,650657317
5264	ENSG00000155906	-0,233625576	4,295625754	4,1295514	0,13974229
5265	ENSG00000155926	-0,791929059	4,084113245	4,9870398	0,03550551
5266	ENSG00000155957	0,066773147	4,248531738	0,1660187	0,687423902
5267	ENSG00000155959	-0,540882783	5,947564032	4,0113782	0,057058675
5268	ENSG00000155962	-0,352640186	4,566616155	2,2064599	0,150946654
5269	ENSG00000155970	0,076727768	4,612504054	0,1316151	0,720059888
5270	ENSG00000155975	-0,159392673	5,659416576	0,9739269	0,333921961
5271	ENSG00000156011	-0,380560282	8,007229101	1,5582307	0,224422143
5272	ENSG00000156017	0,14562655	4,390119404	0,5785544	0,454569768
5273	ENSG00000156026	-0,031364694	4,378210391	0,0744239	0,80003459
5274	ENSG00000156030	0,561134491	4,906867224	4,4386391	0,046191246
5275	ENSG00000156042	0,306001559	3,57271901	2,1690535	0,154301176
5276	ENSG00000156052	0,253476251	5,784419328	1,3840071	0,251394571
5277	ENSG00000156096	-0,980772094	4,113417463	5,1894788	0,032287215
5278	ENSG00000156103	0,569831439	4,008935	3,3771549	0,078995876
5279	ENSG00000156110	-0,576951121	4,793439652	13,610962	0,001205402
5280	ENSG00000156136	0,129810332	3,665291762	0,4514916	0,508288928
5281	ENSG00000156162	0,024944307	5,67927405	0,0107584	0,918283989
5282	ENSG00000156170	-0,253652369	4,073141261	2,7215301	0,130328259
5283	ENSG00000156171	-0,324529433	5,251044318	2,7884211	0,108437385
5284	ENSG00000156218	0,194659933	5,645149455	0,730311	0,401553859
5285	ENSG00000156219	-0,636956901	4,992546504	4,2453923	0,050786486
5286	ENSG00000156232	0,139295578	3,775298261	0,7156077	0,464564047
5287	ENSG00000156239	-0,142345684	4,337392104	0,8491063	0,366324276
5288	ENSG00000156253	-0,459828263	4,219603229	6,6987061	0,016402169
5289	ENSG00000156256	-0,271372619	6,290098695	1,5192622	0,230122357
5290	ENSG00000156261	-0,375452221	6,813139828	3,1410903	0,089525999
5291	ENSG00000156273	0,099176642	6,209439357	0,2362774	0,631484579
5292	ENSG00000156298	-0,230062373	5,017208068	3,4129808	0,077480626
5293	ENSG00000156304	0,001478539	4,35088765	3,912E-05	0,99506323
5294	ENSG00000156313	-0,316078147	4,006028512	3,0545059	0,093788021
5295	ENSG00000156381	-0,187250628	5,488153154	1,2324353	0,278350685
5296	ENSG00000156411	-0,727135442	6,978377194	10,093827	0,004185562
5297	ENSG00000156453	-0,499095378	4,235694328	2,9960745	0,096797124
5298	ENSG00000156463	-0,003103777	6,295883429	0,0002481	0,987567628
5299	ENSG00000156467	-0,904853695	9,534667783	8,6690338	0,007256527
5300	ENSG00000156469	-0,688127123	4,346674958	21,21181	0,000122504
5301	ENSG00000156471	-1,150346111	7,003948681	33,533535	6,58832E-06
5302	ENSG00000156482	0,230155763	7,259390742	1,2207138	0,280589942
5303	ENSG00000156502	-0,212640795	4,836761136	3,1986912	0,086768721
5304	ENSG00000156504	0,132642583	4,30147915	1,2769788	0,347462408
5305	ENSG00000156508	0,445718769	12,56262311	2,5668648	0,122656823
5306	ENSG00000156515	-0,133888915	7,581724352	0,5521618	0,464922208
5307	ENSG00000156531	-0,03519086	4,748153556	0,0518587	0,821862391
5308	ENSG00000156535	-0,173518069	5,793683025	0,6544731	0,426767731

5309	ENSG00000156587	0,367321814	4,213117	3,0139262	0,095866074
5310	ENSG00000156599	-0,027215847	5,876752977	0,0415806	0,840199956
5311	ENSG00000156639	-0,013949681	5,231532407	0,0085292	0,927212895
5312	ENSG00000156642	-0,238971607	6,87371216	3,9660948	0,058328997
5313	ENSG00000156650	-0,035954169	6,060629459	0,0950787	0,760569643
5314	ENSG00000156671	-0,023523378	6,22128162	0,024733	0,876399154
5315	ENSG00000156675	0,666989128	4,997661265	13,790207	0,001135558
5316	ENSG00000156697	-0,137094308	3,872500722	0,8199999	0,478801771
5317	ENSG00000156709	-0,391516792	6,195621357	5,1414807	0,033019618
5318	ENSG00000156735	0,013731895	4,509391817	0,0067532	0,9352126
5319	ENSG00000156787	0,057084106	4,038678776	0,0604387	0,807973829
5320	ENSG00000156795	-0,469509538	3,912427681	4,3735941	0,047683378
5321	ENSG00000156802	-0,029285223	5,002766711	0,0252253	0,875186658
5322	ENSG00000156804	-0,013097448	8,354043862	0,0016629	0,967822947
5323	ENSG00000156831	-0,239217858	4,339926826	2,081496	0,162510164
5324	ENSG00000156885	-0,160249905	6,085802762	0,3033498	0,587074762
5325	ENSG00000156928	-0,427455151	4,414226218	9,5025732	0,005226412
5326	ENSG00000156931	0,476867208	6,368780232	3,7384912	0,065511763
5327	ENSG00000156958	0,15285853	4,818402671	1,2072292	0,283163744
5328	ENSG00000156976	-0,028706074	8,63770801	0,0205795	0,887173683
5329	ENSG00000156990	-0,368018487	3,973440424	5,6288755	0,032256686
5330	ENSG00000157020	0,044493632	5,438642052	0,1481732	0,707837712
5331	ENSG00000157036	0,032739036	4,912365277	0,0174447	0,896065264
5332	ENSG00000157045	-0,102933667	4,015255904	0,5487214	0,604387432
5333	ENSG00000157064	0,418566309	5,118678292	1,2548354	0,274136538
5334	ENSG00000157077	0,083124476	5,281642924	0,6294535	0,435597555
5335	ENSG00000157106	0,010910706	7,826303064	0,0046015	0,946499072
5336	ENSG00000157107	0,2139238	5,563903223	1,1832567	0,287907613
5337	ENSG00000157110	-0,156131026	6,416782992	0,7209677	0,40453577
5338	ENSG00000157150	0,074072061	4,564800108	0,0198827	0,889086805
5339	ENSG00000157181	-0,141923398	5,257164062	0,6403485	0,431733222
5340	ENSG00000157184	0,28626076	3,888800727	2,8215631	0,129313621
5341	ENSG00000157191	0,072334254	4,334871912	0,1343703	0,717277533
5342	ENSG00000157212	-0,080040344	3,600566469	0,3336133	0,664350141
5343	ENSG00000157214	0,059826974	6,059336621	0,0785486	0,781767185
5344	ENSG00000157216	-0,113531918	4,287813958	0,3744132	0,546581152
5345	ENSG00000157224	-0,389561467	5,43381366	4,7321827	0,040080449
5346	ENSG00000157227	0,005572411	5,197722853	0,0004557	0,983152255
5347	ENSG00000157240	0,286692484	4,678225296	2,8581532	0,104355445
5348	ENSG00000157259	0,171850033	5,003229045	1,5053126	0,23218207
5349	ENSG00000157326	-0,155596512	3,950405219	0,7769888	0,387138687
5350	ENSG00000157349	0,002525134	4,084342696	0,0004226	0,994371717
5351	ENSG00000157350	-0,280361437	4,013745995	2,1751352	0,153749735
5352	ENSG00000157426	-0,070248038	4,24750977	0,2608368	0,704965036
5353	ENSG00000157445	-0,327221362	4,535622988	2,7068056	0,113454145
5354	ENSG00000157450	0,064794388	4,834495343	0,4081156	0,625173746
5355	ENSG00000157483	0,048848172	4,686800015	0,161645	0,739160009
5356	ENSG00000157500	-0,079542324	6,584808233	0,223103	0,641115074
5357	ENSG00000157510	0,272290047	5,625903102	3,4584335	0,075698301
5358	ENSG00000157514	0,747907498	6,235799566	3,2241209	0,085647814
5359	ENSG00000157538	-0,060291098	5,396146535	0,4335472	0,525853256
5360	ENSG00000157540	-0,008597766	6,925251888	0,0043844	0,947774288
5361	ENSG00000157554	-0,106816983	4,641490445	0,6130545	0,441561857
5362	ENSG00000157557	-0,346198629	6,21746231	3,1674575	0,088272885
5363	ENSG00000157570	-0,054816842	6,337076921	0,0349163	0,853401579
5364	ENSG00000157593	-0,04831902	4,30757595	0,1615593	0,69606206
5365	ENSG00000157600	0,047588441	5,328165062	0,0654121	0,800400185
5366	ENSG00000157601	0,540666143	4,575332476	6,096539	0,021358798
5367	ENSG00000157617	0,699665746	4,891933773	3,1109748	0,090982478

5368	ENSG00000157625	0,065140878	4,894986104	0,4612339	0,555360915
5369	ENSG00000157637	0,368954474	5,017433461	4,0412448	0,056211249
5370	ENSG00000157654	0,011547682	8,08255614	0,0058843	0,939512896
5371	ENSG00000157680	1,463510543	5,586138491	16,879946	0,000426041
5372	ENSG00000157734	-1,691382486	4,532956843	17,92614	0,000311548
5373	ENSG00000157741	0,282650337	5,961960825	3,5070535	0,073803203
5374	ENSG00000157764	0,103602329	6,115101224	0,9684631	0,335218844
5375	ENSG00000157796	0,150019779	4,766061674	0,5542905	0,464072969
5376	ENSG00000157800	0,098736395	4,59096702	0,5245878	0,476134222
5377	ENSG00000157823	-0,069883377	4,29078288	0,2272826	0,638014588
5378	ENSG00000157827	-0,100185286	4,919287	0,180421	0,674939811
5379	ENSG00000157837	0,274981254	4,757466833	4,0306782	0,056471344
5380	ENSG00000157869	0,015892045	4,832853072	0,0117921	0,914463787
5381	ENSG00000157881	-0,099952778	4,195733507	0,3204359	0,576801192
5382	ENSG00000157916	-0,191848973	5,573877153	4,2131552	0,051561346
5383	ENSG00000157933	0,243698569	5,065030094	0,7778669	0,386874921
5384	ENSG00000157954	-0,015576948	5,418954902	0,0191935	0,891008902
5385	ENSG00000157985	-0,147579726	4,869831253	0,4295725	0,518669842
5386	ENSG00000158019	-0,088825742	5,056989946	0,4289603	0,518942805
5387	ENSG00000158022	-0,409423969	6,694251216	2,9209767	0,100830554
5388	ENSG00000158042	-0,271071545	4,573674721	4,2577129	0,056175218
5389	ENSG00000158079	0,246351057	4,897912703	1,9556184	0,175257598
5390	ENSG00000158092	0,001506735	4,790253377	7,789E-05	0,993034272
5391	ENSG00000158109	0,173789228	5,404906383	1,517473	0,230356448
5392	ENSG00000158122	-0,355012945	4,365562993	5,0910553	0,033809158
5393	ENSG00000158158	-0,095931532	4,528453031	0,1403114	0,711386515
5394	ENSG00000158161	0,101418581	4,893127848	0,6211143	0,481137935
5395	ENSG00000158169	-0,227420705	4,441255363	0,6082682	0,443354499
5396	ENSG00000158186	-0,224209337	7,264361319	1,5776156	0,221652983
5397	ENSG00000158195	0,454983717	6,083676586	3,1952321	0,08697473
5398	ENSG00000158258	0,099324608	6,170545458	0,0769282	0,783969865
5399	ENSG00000158270	0,2892757	5,452488224	1,0901496	0,307236
5400	ENSG00000158286	0,346794963	5,217529766	1,4239383	0,244861727
5401	ENSG00000158290	0,263897459	6,015461111	2,5149926	0,126356124
5402	ENSG00000158301	0,302732585	4,818609015	3,7370878	0,065558775
5403	ENSG00000158321	-0,317095128	4,683713871	3,889053	0,060684217
5404	ENSG00000158352	-0,178776745	4,92365856	0,7341793	0,400328953
5405	ENSG00000158411	-0,017975668	3,831953544	0,0148075	0,96138474
5406	ENSG00000158417	-0,047874857	6,922457134	0,0723062	0,79039388
5407	ENSG00000158435	-0,036798921	4,992418213	0,0925336	0,763692953
5408	ENSG00000158467	0,352041719	3,912948701	4,2295625	0,051185239
5409	ENSG00000158470	-0,315211505	5,339090267	3,4642859	0,075474622
5410	ENSG00000158526	0,240693257	4,77618382	3,4452879	0,07779463
5411	ENSG00000158528	-0,118194414	6,415130248	0,2265555	0,63855803
5412	ENSG00000158560	-0,414581351	4,774383451	3,5287281	0,072985905
5413	ENSG00000158604	0,176171396	5,929592568	2,8124549	0,10696291
5414	ENSG00000158615	0,469960959	6,057121152	2,8745053	0,10342434
5415	ENSG00000158623	-0,043431237	4,663986465	0,0882325	0,772042067
5416	ENSG00000158636	-0,088169341	4,940476702	0,5785012	0,454563605
5417	ENSG00000158669	-0,079156133	5,317314302	0,4180529	0,524257405
5418	ENSG00000158691	0,060432234	3,602759644	0,368364	0,751488525
5419	ENSG00000158710	0,031475243	6,750052426	0,0174058	0,896180505
5420	ENSG00000158711	-0,039920105	6,345955779	0,0719321	0,7909204
5421	ENSG00000158747	0,252691406	5,097732883	1,0648713	0,31278461
5422	ENSG00000158769	0,189081611	3,98157367	0,6828341	0,417061611
5423	ENSG00000158773	-0,019880346	4,604912639	0,0200364	0,888658103
5424	ENSG00000158793	-0,19356925	4,04664564	2,1503315	0,225513856
5425	ENSG00000158796	-0,156396561	4,100782081	1,5844543	0,330458472
5426	ENSG00000158813	-0,647560057	3,882283566	8,9827372	0,006411783

5427	ENSG00000158828	-0,104305526	4,906029455	0,2652149	0,611450244
5428	ENSG00000158856	-0,224072949	4,990974562	0,8407341	0,368655874
5429	ENSG00000158859	-1,563836068	5,610137944	18,871684	0,000236541
5430	ENSG00000158863	0,100805115	4,79666328	0,3584281	0,555203259
5431	ENSG00000158864	-0,434621043	7,298109338	6,2860726	0,019639072
5432	ENSG00000158869	-0,853787338	4,350232397	9,4125179	0,005425042
5433	ENSG00000158882	-0,612678083	4,32963655	10,242265	0,003959004
5434	ENSG00000158941	0,087265558	4,744515759	0,2300354	0,636004955
5435	ENSG00000158966	0,639182546	4,481432679	2,7329904	0,11181559
5436	ENSG00000158985	0,04754265	5,030962972	0,0910865	0,765499368
5437	ENSG00000158987	0,144251284	3,601994674	0,975203	0,432047428
5438	ENSG00000159023	0,212844636	5,874392161	1,8564607	0,186164084
5439	ENSG00000159063	-0,126255689	4,217834378	1,1111847	0,454133585
5440	ENSG00000159069	0,075229253	5,890254865	0,1654904	0,687893707
5441	ENSG00000159082	0,063263018	4,554299013	0,1758014	0,678877923
5442	ENSG00000159086	-0,051368606	5,361509359	0,1307858	0,720899114
5443	ENSG00000159111	0,019345187	4,760219563	0,0219278	0,956118114
5444	ENSG00000159128	-0,000934318	5,003587078	4,67E-05	0,994605927
5445	ENSG00000159131	-0,17905044	5,241741724	3,3094963	0,092896734
5446	ENSG00000159140	0,008283242	8,089067326	0,0046152	0,946419323
5447	ENSG00000159176	0,416758627	6,655007258	2,3131381	0,141848669
5448	ENSG00000159189	-0,2017033	5,271331523	0,4851575	0,493041607
5449	ENSG00000159199	-0,769970299	6,66525418	12,277506	0,001899588
5450	ENSG00000159200	0,437789909	5,65386449	5,2813328	0,030935748
5451	ENSG00000159202	0,239876675	5,528771022	3,1822975	0,08753058
5452	ENSG00000159210	-0,275168196	4,61548878	3,0205125	0,095525196
5453	ENSG00000159216	-0,106649548	4,372584975	0,0811611	0,778267208
5454	ENSG00000159251	-0,742558408	12,10949611	5,998371	0,022287722
5455	ENSG00000159256	0,102976987	6,105051095	0,1530481	0,699222539
5456	ENSG00000159267	0,050381626	4,043573187	0,1623507	0,768837202
5457	ENSG00000159314	-0,13886262	4,098250852	0,4602138	0,504260198
5458	ENSG00000159322	-0,023457359	4,718368467	0,0264741	0,87216004
5459	ENSG00000159335	-0,110395348	5,427051343	0,1737025	0,680687419
5460	ENSG00000159346	0,039992853	6,327436443	0,0793248	0,780714005
5461	ENSG00000159348	0,290096164	5,974958962	3,1108489	0,090988625
5462	ENSG00000159352	-0,273889366	6,426052935	3,0384374	0,094604571
5463	ENSG00000159377	-0,110465649	6,409883508	0,5810193	0,45360206
5464	ENSG00000159388	0,156658907	7,113421358	0,1654796	0,687903321
5465	ENSG00000159399	-0,430877266	5,233321917	1,6817262	0,207492806
5466	ENSG00000159403	-0,035657405	7,994820627	0,0154012	0,902307979
5467	ENSG00000159423	0,270691386	4,479051024	1,2533639	0,274410775
5468	ENSG00000159433	-0,208720273	6,060768993	0,6280036	0,436147628
5469	ENSG00000159445	-0,13766681	4,496435466	1,8546112	0,352334981
5470	ENSG00000159459	-0,072665195	6,375665125	0,2666416	0,610485134
5471	ENSG00000159461	0,014247402	6,062611152	0,0109436	0,917583517
5472	ENSG00000159479	-0,164063112	4,142211383	1,9482227	0,349076132
5473	ENSG00000159579	0,099424644	4,794536332	0,7281352	0,402214984
5474	ENSG00000159592	0,06091024	6,625836162	0,5832248	0,452750106
5475	ENSG00000159593	-0,227937744	5,209569457	1,9372902	0,177213513
5476	ENSG00000159596	-0,132388907	4,105458354	1,2332796	0,434108824
5477	ENSG00000159658	0,173922473	6,714786876	2,7405869	0,111298408
5478	ENSG00000159692	0,011261606	5,188632771	0,0060083	0,938880957
5479	ENSG00000159720	-0,077581545	5,198194883	0,2040803	0,655658086
5480	ENSG00000159788	0,156661026	3,684640726	0,7763267	0,466664946
5481	ENSG00000159792	0,066016408	4,031846584	0,1160557	0,7364295
5482	ENSG00000159840	-0,242104558	5,357100856	0,813508	0,37638871
5483	ENSG00000159842	0,24175009	6,324528687	0,9883148	0,330449188
5484	ENSG00000159873	0,355143328	4,351558648	5,5705107	0,027070695
5485	ENSG00000159899	0,225417911	4,089649917	1,0947222	0,306246607

5486	ENSG00000159917	0,032752187	3,499613117	0,0610554	0,88665235
5487	ENSG00000159921	-0,011814288	4,778115088	0,0145388	0,905064473
5488	ENSG00000160007	-0,058690968	6,836018401	0,0969592	0,758300977
5489	ENSG00000160014	0,274766255	6,952563244	1,8499176	0,186912594
5490	ENSG00000160049	0,046832532	4,86955462	0,130049	0,78325584
5491	ENSG00000160058	0,223991755	5,493512497	1,6221174	0,215456617
5492	ENSG00000160075	-0,292851465	5,721260284	3,1377789	0,089684823
5493	ENSG00000160094	0,191989259	4,184638769	1,0747398	0,31060239
5494	ENSG00000160097	-0,711841065	5,855847414	5,4049038	0,029216331
5495	ENSG00000160111	-0,328572277	3,983222352	0,6229292	0,437982858
5496	ENSG00000160131	0,093117236	5,351150958	0,3190727	0,577607401
5497	ENSG00000160145	0,151536673	5,645299003	0,9256331	0,345958475
5498	ENSG00000160179	-0,601057418	4,467964388	4,3565491	0,048083335
5499	ENSG00000160194	-0,409399655	6,134376626	7,7818435	0,010372027
5500	ENSG00000160201	-0,214999656	5,495438893	1,9814738	0,172542921
5501	ENSG00000160208	0,056835578	4,373166615	0,1695574	0,735146101
5502	ENSG00000160209	0,225035372	5,821896978	0,7921923	0,382609168
5503	ENSG00000160213	-0,127905509	5,171100658	0,8279891	0,372220258
5504	ENSG00000160216	-0,139970405	5,644904825	0,6502126	0,428256091
5505	ENSG00000160218	0,025416971	5,208740889	0,0331759	0,857054859
5506	ENSG00000160219	-0,093805509	5,692003819	0,121174	0,730915227
5507	ENSG00000160221	-0,1131936	6,23053346	0,4012029	0,532686955
5508	ENSG00000160255	-0,257194739	4,61987602	0,80028	0,380231339
5509	ENSG00000160285	-0,015920124	5,271131317	0,0038953	0,950770837
5510	ENSG00000160294	0,053949069	5,19317669	0,0714035	0,791674013
5511	ENSG00000160299	-0,726443292	5,928206814	6,4140634	0,018564919
5512	ENSG00000160305	0,150780549	5,296904312	0,7141502	0,406732761
5513	ENSG00000160310	0,161295715	5,933159027	2,0921761	0,161434674
5514	ENSG00000160321	0,785957618	5,32542764	7,8485552	0,01010609
5515	ENSG00000160336	-0,027620812	3,683770814	0,0414213	0,902025847
5516	ENSG00000160392	-0,212098588	4,555654604	1,092008	0,306833368
5517	ENSG00000160439	0,107272944	3,739102414	0,2013263	0,657830965
5518	ENSG00000160445	0,312796982	5,135232952	2,5694283	0,122526739
5519	ENSG00000160539	-0,260264253	5,618681745	1,8006327	0,1926711
5520	ENSG00000160551	0,069201935	7,582775897	0,3664091	0,550845605
5521	ENSG00000160584	0,078598962	5,44852936	0,1572745	0,695316201
5522	ENSG00000160593	0,572186632	3,690628235	2,6954666	0,11417242
5523	ENSG00000160613	-0,058982806	4,517140468	0,0509041	0,823480607
5524	ENSG00000160633	0,12993704	5,534072349	0,8012283	0,379942125
5525	ENSG00000160678	-0,334427736	4,417197615	2,166278	0,154553623
5526	ENSG00000160679	-0,109730885	5,854972067	0,99911	0,327840602
5527	ENSG00000160685	-0,146236782	4,160717144	0,5972918	0,447445165
5528	ENSG00000160688	-0,303240881	4,011452418	2,9419176	0,099686529
5529	ENSG00000160691	0,089888301	5,146735048	0,2032778	0,656289376
5530	ENSG00000160695	0,130998848	4,806776363	0,997315	0,357549614
5531	ENSG00000160710	0,264775019	6,957739285	2,6469108	0,117309137
5532	ENSG00000160712	0,421084127	4,415815571	1,6129447	0,216715839
5533	ENSG00000160714	0,053850052	4,99312024	0,147127	0,704792536
5534	ENSG00000160746	0,020018146	4,157457253	0,0293065	0,901933779
5535	ENSG00000160752	-0,23783458	4,523123342	3,487386	0,082899299
5536	ENSG00000160785	0,175278219	4,515155273	0,7227221	0,403973332
5537	ENSG00000160789	0,111568797	7,077420091	0,2087164	0,652040057
5538	ENSG00000160796	-0,080117439	4,180452935	0,1250221	0,72685505
5539	ENSG00000160803	0,053718609	4,60530006	0,1111789	0,74181199
5540	ENSG00000160808	0,012301831	6,967126111	0,00056	0,981323229
5541	ENSG00000160818	-0,370736434	4,278730062	4,7654434	0,039447398
5542	ENSG00000160862	0,57644708	5,076530412	3,5917694	0,070641797
5543	ENSG00000160908	0,033125393	4,419039208	0,0762512	0,889439983
5544	ENSG00000160948	-0,026632965	5,143587285	0,0236546	0,879102072

5545	ENSG00000160953	0,252495082	4,25944202	2,3730988	0,137022292
5546	ENSG00000160961	0,014465195	4,041050938	0,0085374	0,990940727
5547	ENSG00000161010	0,41590131	3,714399818	5,445035	0,034785928
5548	ENSG00000161011	0,435359201	6,851488905	4,4609074	0,045692573
5549	ENSG00000161013	0,313294402	5,181509835	4,4163464	0,046694875
5550	ENSG00000161016	0,095059294	8,313116357	0,2387642	0,629704409
5551	ENSG00000161021	0,005455415	4,382655313	0,0007327	0,978638238
5552	ENSG00000161048	0,209694947	4,474550987	1,4527845	0,240277448
5553	ENSG00000161057	-0,444669812	6,207679496	4,1932177	0,052114239
5554	ENSG00000161202	0,217700882	5,423542397	1,4702671	0,237552426
5555	ENSG00000161203	-0,047834498	7,233901573	0,1320018	0,719655915
5556	ENSG00000161204	0,040595593	5,040980853	0,079155	0,780945708
5557	ENSG00000161217	-0,004027515	6,657094647	0,0006756	0,9794872
5558	ENSG00000161267	-0,691523604	4,690321953	4,9994832	0,035297635
5559	ENSG00000161281	-0,481785452	8,173885849	3,1717129	0,08807255
5560	ENSG00000161298	0,054177233	4,418874012	0,1166572	0,735774479
5561	ENSG00000161381	-0,568977367	4,239614088	4,2912889	0,049650064
5562	ENSG00000161526	-0,196985907	4,941439491	2,7923277	0,108156897
5563	ENSG00000161533	0,078658756	6,713201854	0,5889554	0,450565652
5564	ENSG00000161542	-0,236806516	4,538113481	4,7884067	0,077470621
5565	ENSG00000161547	0,028542613	5,99247252	0,0348356	0,853569113
5566	ENSG00000161551	0,343813573	4,278254901	11,036708	0,024185846
5567	ENSG00000161638	0,078021113	5,997700316	0,1307396	0,720950917
5568	ENSG00000161647	-0,88147099	5,06827869	7,1445657	0,013553906
5569	ENSG00000161654	-0,291369905	4,651359636	8,8194095	0,025611069
5570	ENSG00000161671	0,399969685	5,356891492	3,781007	0,064105618
5571	ENSG00000161791	0,713285015	4,877990855	9,756193	0,004755762
5572	ENSG00000161813	-0,193366907	6,696986698	1,0267713	0,321409022
5573	ENSG00000161896	-0,466562511	3,737336308	1,6213596	0,215560306
5574	ENSG00000161904	0,101142462	4,541415183	0,542892	0,557809678
5575	ENSG00000161940	0,09574712	3,85362039	0,2891214	0,653193163
5576	ENSG00000161970	-0,104371459	7,042979448	0,1176529	0,734694334
5577	ENSG00000161981	-0,543869022	4,686020529	8,1306658	0,009006779
5578	ENSG00000162086	-0,253156069	4,454857325	4,6915527	0,070254595
5579	ENSG00000162104	-0,082672405	5,012290682	0,1353411	0,716304866
5580	ENSG00000162129	-0,128999904	4,151593624	0,5167744	0,479426233
5581	ENSG00000162139	0,22352893	4,256341553	1,7480386	0,199059495
5582	ENSG00000162191	-0,00820632	5,069732125	0,0027813	0,958393733
5583	ENSG00000162231	-0,007642804	5,469975162	0,0019204	0,96542274
5584	ENSG00000162236	0,184419408	4,180061982	1,7396157	0,286154274
5585	ENSG00000162244	0,172389304	7,618702177	0,7775631	0,386966149
5586	ENSG00000162298	0,22410524	4,270795826	1,1191904	0,301024906
5587	ENSG00000162337	0,98625955	4,834723492	11,714541	0,002315591
5588	ENSG00000162368	0,246927363	6,793276178	1,8583781	0,185945444
5589	ENSG00000162377	-0,152828444	3,98407843	1,8379949	0,384303387
5590	ENSG00000162378	-0,041033411	6,986544827	0,0683396	0,796085206
5591	ENSG00000162384	-0,179361163	4,946266276	2,3625738	0,153747609
5592	ENSG00000162390	0,096076451	5,071529532	0,070857	0,79245328
5593	ENSG00000162402	-0,084198466	7,288406313	0,4688548	0,500300329
5594	ENSG00000162407	-0,444180283	6,638906032	13,064058	0,001443245
5595	ENSG00000162408	-0,012658876	4,655407748	0,006267	0,937583427
5596	ENSG00000162409	0,032694737	8,61093525	0,0059879	0,938985994
5597	ENSG00000162413	-0,186111386	5,926276646	0,5904352	0,450031293
5598	ENSG00000162430	-0,097332855	4,620175443	0,1843289	0,671654828
5599	ENSG00000162433	0,106119918	6,707924135	0,4633715	0,502815506
5600	ENSG00000162434	-0,028102782	7,441757794	0,0550093	0,81662338
5601	ENSG00000162437	-0,228314398	5,189227551	3,970685	0,058194413
5602	ENSG00000162441	-0,13922771	5,377154106	1,2570587	0,273683068
5603	ENSG00000162458	-0,552186984	5,776066134	6,8119853	0,015620452

5604	ENSG00000162496	-0,382835928	5,639436093	2,4574633	0,130557698
5605	ENSG00000162511	-0,14077553	4,586405241	0,2608188	0,614401771
5606	ENSG00000162512	0,704786628	4,998507238	5,0183403	0,034985211
5607	ENSG00000162517	-0,001310415	4,874176881	6,536E-05	0,993618692
5608	ENSG00000162521	0,152361217	5,956400902	1,8117708	0,191305704
5609	ENSG00000162576	0,807664764	4,714523554	9,9598968	0,004402231
5610	ENSG00000162599	-0,047402593	8,762897984	0,1185167	0,733750647
5611	ENSG00000162600	-0,015615421	5,078108121	0,0050261	0,944091124
5612	ENSG00000162601	-0,172182701	6,264724625	1,5559729	0,224708373
5613	ENSG00000162604	-0,079438629	4,841901253	0,3083296	0,584034384
5614	ENSG00000162607	0,076154857	5,206342621	0,2227608	0,641369829
5615	ENSG00000162613	-0,062945589	6,715584185	0,1583756	0,694308527
5616	ENSG00000162614	0,11153246	8,268962586	0,1471773	0,704753898
5617	ENSG00000162616	-0,04243707	6,655455973	0,0202617	0,888042117
5618	ENSG00000162618	-0,409761447	5,595675834	3,7165437	0,066251384
5619	ENSG00000162623	-0,03902973	5,130147938	0,0389616	0,845250409
5620	ENSG00000162627	-0,233939286	4,580678456	1,9967855	0,170959363
5621	ENSG00000162630	-0,909623323	6,17924069	4,7199746	0,040315664
5622	ENSG00000162636	0,649096898	5,05269876	5,09508	0,033745371
5623	ENSG00000162642	-0,125990037	3,881323682	0,9607523	0,494427882
5624	ENSG00000162645	0,003398541	5,632166213	0,0001507	0,990309868
5625	ENSG00000162654	0,11269243	5,135092641	0,1129396	0,739853728
5626	ENSG00000162664	-0,061098108	5,196151215	0,1971378	0,661159532
5627	ENSG00000162669	0,1667287	3,822324582	0,2469375	0,623933667
5628	ENSG00000162687	2,56328796	5,20901039	8,9032623	0,006615049
5629	ENSG00000162688	-0,299117916	7,096328047	1,6202662	0,215710018
5630	ENSG00000162692	0,121991116	5,482032216	0,1091234	0,744120079
5631	ENSG00000162694	0,056098661	4,381836362	0,0828516	0,776034749
5632	ENSG00000162695	0,256650103	5,112227919	1,3002377	0,265848612
5633	ENSG00000162702	0,034673689	4,383493225	0,0546155	0,817273936
5634	ENSG00000162704	0,142266258	6,108844875	0,4118972	0,527322525
5635	ENSG00000162714	0,300291915	4,311571597	2,9378644	0,099906777
5636	ENSG00000162733	-0,058727913	7,909212474	0,0932214	0,762853846
5637	ENSG00000162734	0,046837345	7,063062993	0,0724877	0,790137409
5638	ENSG00000162735	-0,021305523	5,83594661	0,0175997	0,895607379
5639	ENSG00000162736	0,084172086	5,8092867	0,341609	0,564553039
5640	ENSG00000162772	0,877072055	5,18596136	1,7040379	0,204606605
5641	ENSG00000162775	0,007468643	4,70470026	0,003683	0,962652572
5642	ENSG00000162783	0,299001471	4,952962125	2,3015928	0,142801076
5643	ENSG00000162804	0,18337302	4,077673854	0,2805987	0,601360964
5644	ENSG00000162813	-0,04865503	4,197503107	0,1278243	0,75560883
5645	ENSG00000162817	-0,474265907	5,053414859	4,7866174	0,039050252
5646	ENSG00000162819	0,116793887	5,499184033	0,6764296	0,419206037
5647	ENSG00000162825	0,25184846	5,295797841	2,0953048	0,161181177
5648	ENSG00000162836	-0,391102355	4,092396016	3,9480201	0,05890483
5649	ENSG00000162851	-0,138208746	3,638658339	1,0948966	0,49519676
5650	ENSG00000162852	-0,125382843	5,232766314	0,9250363	0,346076592
5651	ENSG00000162869	0,306793589	4,819937547	8,7376532	0,013939417
5652	ENSG00000162878	-0,54142005	5,726212399	5,4776173	0,02825462
5653	ENSG00000162885	-0,325894443	4,607485575	7,0108536	0,014321741
5654	ENSG00000162889	-0,197246238	6,991860695	1,0534088	0,315345652
5655	ENSG00000162909	0,148568873	7,046960204	1,0758763	0,310318769
5656	ENSG00000162923	-0,116459961	6,934859556	0,7655032	0,390582148
5657	ENSG00000162924	0,543058968	4,503640877	1,9569055	0,175121245
5658	ENSG00000162927	0,125072329	3,729876846	0,6873782	0,4799941
5659	ENSG00000162928	-0,108832086	5,057445751	0,8186113	0,384735
5660	ENSG00000162929	-0,031401794	3,698194237	0,0633986	0,866892744
5661	ENSG00000162944	0,417841242	3,957699379	3,0744592	0,092785391
5662	ENSG00000162946	-0,006134068	3,650995056	0,0009016	0,976304223

5663	ENSG00000162961	-0,193168395	3,980440889	1,4175054	0,300224631
5664	ENSG00000162971	-0,044302465	3,988553015	0,1053141	0,80752969
5665	ENSG00000162976	0,463087647	4,227183465	5,1361076	0,033102757
5666	ENSG00000162980	-0,019174004	6,358831899	0,0077014	0,930825259
5667	ENSG00000162989	-0,758836958	6,060696374	6,8925324	0,015089733
5668	ENSG00000162999	-0,054534471	3,877359726	0,1393575	0,771278053
5669	ENSG00000163001	0,14378383	4,804290193	0,8652332	0,361892593
5670	ENSG00000163002	-0,062345834	3,629027907	0,1816512	0,763901101
5671	ENSG00000163029	-0,063949991	5,305651726	0,2264097	0,638651512
5672	ENSG00000163032	-0,210121988	5,066740146	0,4091114	0,528710309
5673	ENSG00000163041	-0,095427118	7,437169899	0,5390876	0,470169193
5674	ENSG00000163050	-0,534265807	6,989158764	5,4509696	0,028602897
5675	ENSG00000163069	-0,101729171	6,807516624	0,5283	0,474597445
5676	ENSG00000163072	0,129930109	4,640391612	0,8214716	0,374083472
5677	ENSG00000163092	0,270951449	8,385466042	0,3319604	0,570075521
5678	ENSG00000163104	-0,034841731	5,482603701	0,0449625	0,833931715
5679	ENSG00000163110	-0,208536086	9,889781623	0,5855607	0,451884442
5680	ENSG00000163125	0,199690592	5,370173274	1,6570015	0,210750652
5681	ENSG00000163131	0,003234828	5,769214532	0,0001183	0,991416383
5682	ENSG00000163138	0,060369534	4,13860848	0,2300567	0,718815418
5683	ENSG00000163159	-0,062617633	4,0985037	0,2226168	0,684606091
5684	ENSG00000163161	0,033012938	4,909666054	0,0641246	0,938796235
5685	ENSG00000163162	-0,376386041	5,397320009	2,5819058	0,121668352
5686	ENSG00000163166	-0,261069665	5,580034642	5,5734254	0,027008473
5687	ENSG00000163171	-0,080031145	6,360244026	0,2083825	0,652298972
5688	ENSG00000163191	-0,157774613	5,337292423	0,2782239	0,602894823
5689	ENSG00000163214	0,106750361	4,555107356	1,0123486	0,423741239
5690	ENSG00000163220	-0,84508471	6,799886891	2,3531304	0,13860758
5691	ENSG00000163249	0,695113243	4,481534275	4,4464355	0,04601596
5692	ENSG00000163257	0,152541002	4,957855664	1,4321314	0,243506452
5693	ENSG00000163281	-0,233869519	5,067555981	1,0005505	0,327535188
5694	ENSG00000163291	-0,023776678	4,410416282	0,0164919	0,898926797
5695	ENSG00000163297	0,091416029	5,913571126	0,4010015	0,532776949
5696	ENSG00000163312	-0,042753025	4,546378317	0,1087742	0,856065548
5697	ENSG00000163319	-0,454912955	3,739092411	6,7574434	0,019722558
5698	ENSG00000163320	-0,034521866	6,467890011	0,0577057	0,812272403
5699	ENSG00000163328	-0,288276034	5,040364042	3,6547317	0,068386154
5700	ENSG00000163344	-0,379057069	4,683900005	2,9975324	0,096720695
5701	ENSG00000163346	-0,049756181	6,447487545	0,0548013	0,81697098
5702	ENSG00000163348	0,211694209	3,909926442	1,4888197	0,28740806
5703	ENSG00000163349	-0,062104419	6,666645909	0,2105411	0,650614576
5704	ENSG00000163359	-0,154879027	8,270871499	0,3128559	0,581313104
5705	ENSG00000163374	0,029116942	5,815224131	0,0560648	0,814906199
5706	ENSG00000163376	-0,271759579	4,086403508	0,4565845	0,505929707
5707	ENSG00000163378	0,28342547	5,634392774	2,1809695	0,15322295
5708	ENSG00000163380	-0,43173774	6,838390194	1,4670298	0,23805403
5709	ENSG00000163382	-0,182530335	5,163008589	2,0316928	0,167368443
5710	ENSG00000163389	0,060503736	4,102951792	0,1193594	0,732854957
5711	ENSG00000163399	-0,320144103	7,178608245	1,7494091	0,198889729
5712	ENSG00000163412	-0,148584233	5,824916735	1,4594816	0,239186675
5713	ENSG00000163428	0,107748691	5,630916401	0,42745	0,519695213
5714	ENSG00000163430	-0,136461559	8,834394369	0,7169325	0,405803921
5715	ENSG00000163431	-0,600470155	5,274959832	3,3479806	0,080216788
5716	ENSG00000163444	-0,126622044	5,040623186	0,7792389	0,386450556
5717	ENSG00000163453	0,692562887	8,162655002	9,8047419	0,004668761
5718	ENSG00000163466	-0,093907536	6,895491167	0,6143377	0,441090487
5719	ENSG00000163468	-0,384142087	6,895647071	6,5786634	0,017263436
5720	ENSG00000163479	0,138949545	5,757606371	0,5338374	0,472340047
5721	ENSG00000163491	-0,355808383	4,422620998	1,1680119	0,290958505

5722	ENSG00000163492	-0,197223191	8,095297654	0,1485603	0,703439502
5723	ENSG00000163510	0,029272805	4,982239056	0,03275	0,857968971
5724	ENSG00000163512	-0,206892577	4,507198097	1,8004511	0,192692722
5725	ENSG00000163513	-0,345924022	7,946753922	4,9684751	0,035781896
5726	ENSG00000163516	0,134094314	4,543209898	0,8058544	0,389745583
5727	ENSG00000163517	-0,080911062	4,443020388	0,1692351	0,684582209
5728	ENSG00000163520	0,429765551	7,401517475	1,5590428	0,224305273
5729	ENSG00000163527	-0,011225898	7,10503715	0,0047881	0,945428785
5730	ENSG00000163528	-0,536214395	4,030075357	23,605992	0,001658302
5731	ENSG00000163531	0,853231139	5,607581225	6,6670114	0,01662858
5732	ENSG00000163539	-0,107989031	6,857566245	0,4228409	0,521934578
5733	ENSG00000163541	-0,47855015	7,231499964	7,9656553	0,009632769
5734	ENSG00000163558	0,477599334	5,231551249	3,5039168	0,073932806
5735	ENSG00000163563	0,364485306	3,876139954	0,6502879	0,428229719
5736	ENSG00000163565	-0,417705508	6,342624602	3,5537851	0,072043695
5737	ENSG00000163577	0,063168348	3,868890439	0,1369063	0,71474503
5738	ENSG00000163590	-0,781036728	5,877843417	19,710322	0,000186314
5739	ENSG00000163596	-0,002028115	4,347169516	0,0001874	0,989194117
5740	ENSG00000163597	-0,115767507	5,750609561	0,9056951	0,351072052
5741	ENSG00000163602	0,197409813	5,095252895	1,5918177	0,219651455
5742	ENSG00000163605	-0,141971026	6,409316368	0,5153645	0,480019743
5743	ENSG00000163607	-0,407671731	4,015197791	6,0543694	0,021763793
5744	ENSG00000163608	-0,085168166	5,538000408	0,6644703	0,476720005
5745	ENSG00000163618	-0,483454497	6,473836343	1,4687704	0,237784162
5746	ENSG00000163625	0,039186697	6,967443322	0,0806696	0,778912158
5747	ENSG00000163626	-0,108928128	4,288676841	0,5960411	0,467512204
5748	ENSG00000163629	0,87996711	5,730330852	5,2104955	0,031972261
5749	ENSG00000163634	-0,39150085	5,256350025	3,5606245	0,07178894
5750	ENSG00000163636	-0,303943418	6,101531129	3,9445863	0,059006809
5751	ENSG00000163637	0,028311538	5,232184929	0,018686	0,892454099
5752	ENSG00000163638	-1,290784327	6,375228105	6,7412453	0,016103643
5753	ENSG00000163644	-0,216421552	8,44421308	0,2599446	0,61499242
5754	ENSG00000163655	0,132972745	5,569897229	1,4639192	0,238495036
5755	ENSG00000163659	0,08417805	5,97165411	0,1176453	0,734702569
5756	ENSG00000163660	-0,156147406	6,753635674	0,3969127	0,534867382
5757	ENSG00000163681	-0,052954853	8,434443639	0,0291548	0,865908096
5758	ENSG00000163682	0,348562841	8,877404114	2,4646364	0,130024906
5759	ENSG00000163683	0,232078502	5,607986859	1,5656224	0,223361114
5760	ENSG00000163684	-0,030825114	4,734460398	0,043599	0,836430155
5761	ENSG00000163697	0,116472522	6,334061459	0,8472474	0,366807229
5762	ENSG00000163703	-0,068618735	5,133323592	0,1207098	0,731409849
5763	ENSG00000163710	0,213447927	6,734237348	1,2618951	0,272825803
5764	ENSG00000163714	-0,139029194	6,827241383	0,8457962	0,367240605
5765	ENSG00000163719	-0,017578794	5,130234168	0,0140416	0,906697557
5766	ENSG00000163728	-0,015382117	5,626165017	0,0096007	0,922790313
5767	ENSG00000163738	0,061125265	4,386221892	0,1059308	0,747753437
5768	ENSG00000163743	-0,113948822	5,275551463	0,4788597	0,495832899
5769	ENSG00000163754	-0,287883696	6,588664856	3,1288545	0,090114487
5770	ENSG00000163755	0,040704504	3,972657066	0,0971727	0,758034167
5771	ENSG00000163762	-0,531151665	4,059176894	3,2120364	0,086200016
5772	ENSG00000163781	-0,092520362	5,029460081	0,5672255	0,510615446
5773	ENSG00000163785	0,019265762	6,102345915	0,0306171	0,862615781
5774	ENSG00000163788	0,206071412	5,610780345	2,6092489	0,119764779
5775	ENSG00000163798	0,016961597	4,817355021	0,0235344	0,879402553
5776	ENSG00000163807	-0,015525993	5,814122468	0,0133931	0,908863792
5777	ENSG00000163811	-0,042570885	5,309624467	0,0693151	0,794669497
5778	ENSG00000163812	0,134710186	5,501908033	1,4908046	0,234358808
5779	ENSG00000163818	0,442989351	4,161059863	4,3083395	0,049235229
5780	ENSG00000163820	0,131335794	7,38719873	0,2734885	0,605978821

5781	ENSG00000163827	-0,177705555	5,607748879	0,1400447	0,711647862
5782	ENSG00000163833	0,019182661	8,106942425	0,0034116	0,953924915
5783	ENSG00000163840	0,216807036	5,242379209	0,8022424	0,379657662
5784	ENSG00000163848	-0,08312353	6,720548881	0,2148714	0,647311432
5785	ENSG00000163864	-0,249628761	4,037502865	2,2092226	0,150702387
5786	ENSG00000163866	-0,578953103	4,928536223	23,815402	6,14699E-05
5787	ENSG00000163867	-0,101641694	4,389895515	0,4113135	0,549673807
5788	ENSG00000163872	0,145936548	4,809831989	1,2218049	0,28091421
5789	ENSG00000163875	0,146281096	4,863269424	1,8645159	0,187087025
5790	ENSG00000163877	0,129965176	3,86518424	0,9488244	0,460013483
5791	ENSG00000163900	-0,004816381	4,113924678	0,0019326	0,965311865
5792	ENSG00000163902	-0,062099417	6,930265131	0,184961	0,671115606
5793	ENSG00000163904	0,064333616	5,220474597	0,5716829	0,561395572
5794	ENSG00000163909	0,300738326	5,178894818	1,1817731	0,288202643
5795	ENSG00000163913	0,316548438	4,78864927	2,7870134	0,108521687
5796	ENSG00000163930	0,088903624	5,035580562	0,323368	0,575074914
5797	ENSG00000163931	0,290293966	5,192944569	0,5849978	0,452099265
5798	ENSG00000163932	-0,096620601	4,265437005	0,1590264	0,69371485
5799	ENSG00000163935	-0,016470621	4,334040263	0,0133024	0,90917287
5800	ENSG00000163938	-0,258194337	5,584927078	2,2740225	0,145106466
5801	ENSG00000163939	-0,016269136	6,492890683	0,0198298	0,889229353
5802	ENSG00000163946	0,122763236	6,534363332	0,6828595	0,417037326
5803	ENSG00000163947	-0,094198456	4,849507785	0,2167647	0,645873555
5804	ENSG00000163950	0,141002426	3,942005159	0,9683407	0,395670981
5805	ENSG00000163956	-0,060453258	6,962469333	0,1168829	0,735529168
5806	ENSG00000163960	0,2039474	5,793228624	2,0180131	0,168765472
5807	ENSG00000163961	-0,058850508	5,048560668	0,262012	0,619176328
5808	ENSG00000163964	-0,184933391	3,874532961	2,6235459	0,314065077
5809	ENSG00000164022	-0,162725861	5,648734276	0,8946396	0,354007312
5810	ENSG00000164023	0,439442818	5,81058106	1,6947097	0,205807169
5811	ENSG00000164024	-0,249757915	5,205181178	4,8350884	0,038120768
5812	ENSG00000164031	-0,029794637	6,066895084	0,015807	0,901036263
5813	ENSG00000164032	-0,542535281	5,380497917	7,9443396	0,009717075
5814	ENSG00000164035	-0,38781777	5,976930242	2,5367142	0,124811392
5815	ENSG00000164038	-0,023311754	3,996574225	0,0160779	0,900196369
5816	ENSG00000164039	0,261829046	5,129781164	1,8267491	0,189592868
5817	ENSG00000164040	0,039881444	5,677412357	0,0725507	0,790047008
5818	ENSG00000164048	-0,275806589	3,925420106	2,4064239	0,134424315
5819	ENSG00000164050	0,142776458	5,026878847	0,4777434	0,496330524
5820	ENSG00000164054	0,156451637	5,281515393	0,5837736	0,452566967
5821	ENSG00000164056	-0,041691367	5,423702268	0,0325152	0,858473258
5822	ENSG00000164062	-0,137669739	5,438140316	0,5746378	0,456082194
5823	ENSG00000164066	0,06251918	5,19457931	0,1546328	0,697750568
5824	ENSG00000164068	0,050161939	4,271262003	0,0415211	0,840318739
5825	ENSG00000164070	0,145970183	4,881826972	0,4589586	0,50483652
5826	ENSG00000164073	-0,052223648	4,384313375	0,1720705	0,792962902
5827	ENSG00000164074	0,241905673	4,521065563	1,1515245	0,294306846
5828	ENSG00000164080	0,122908684	4,877862771	0,3724795	0,547610492
5829	ENSG00000164081	-0,222078856	4,389048564	1,5473876	0,225990151
5830	ENSG00000164091	0,067255253	6,431234298	0,3047845	0,586179298
5831	ENSG00000164093	-0,96491033	3,995726357	15,414118	0,000670912
5832	ENSG00000164096	-0,046587006	6,591238974	0,0655094	0,800255222
5833	ENSG00000164104	-0,051914231	5,309340989	0,0486057	0,827443239
5834	ENSG00000164107	-0,068455807	4,957317965	0,2244019	0,640142184
5835	ENSG00000164111	0,222558538	7,545465293	0,9511516	0,339524024
5836	ENSG00000164114	0,016596998	4,46337247	0,0061033	0,938401992
5837	ENSG00000164116	0,420787302	6,302956005	3,4893158	0,07449661
5838	ENSG00000164117	0,082704838	4,796553527	0,2482966	0,62298569
5839	ENSG00000164118	-0,09895225	4,966283476	0,3567807	0,556106725

5840	ENSG00000164124	0,095428375	4,623045297	0,1631418	0,689993127
5841	ENSG00000164125	-0,18537348	6,918620765	1,7086615	0,203978577
5842	ENSG00000164134	-0,246439376	5,995036283	2,655531	0,11674496
5843	ENSG00000164142	-0,447327864	4,927545522	4,5225094	0,044344397
5844	ENSG00000164144	0,062817971	4,728974227	0,1499973	0,702081315
5845	ENSG00000164151	0,035460023	6,147412856	0,1408734	0,710824777
5846	ENSG00000164163	-0,152658524	5,887735514	0,4950155	0,488726299
5847	ENSG00000164164	0,090063432	5,975163587	0,2280485	0,637459698
5848	ENSG00000164168	-0,073689231	5,239422473	0,5225758	0,565503214
5849	ENSG00000164169	0,241126585	4,232829126	1,5923088	0,219582652
5850	ENSG00000164172	-0,290391118	5,640451701	3,9423406	0,059056355
5851	ENSG00000164176	0,199819475	5,299832492	0,191288	0,665906295
5852	ENSG00000164180	-0,188376737	4,900543598	1,0657279	0,312594375
5853	ENSG00000164182	-0,35103923	4,091249232	3,3811959	0,078828451
5854	ENSG00000164187	-0,293007088	5,97359962	1,3814052	0,251827986
5855	ENSG00000164190	0,102630379	7,012500455	1,0582285	0,314228529
5856	ENSG00000164197	0,205298617	5,150486809	1,4282181	0,244174542
5857	ENSG00000164209	-0,216946479	6,265497846	1,110923	0,302775697
5858	ENSG00000164211	-0,044596375	4,682272374	0,0231758	0,880322009
5859	ENSG00000164219	-0,138174578	5,43094485	0,6309568	0,435085171
5860	ENSG00000164236	0,142786132	3,859255701	0,2127266	0,648949689
5861	ENSG00000164237	-0,1459318	4,866147312	0,7624413	0,391547416
5862	ENSG00000164244	0,192124782	5,589181033	1,3057976	0,264856322
5863	ENSG00000164252	-0,000422847	4,150178927	4,732E-06	0,99828308
5864	ENSG00000164253	-0,066979674	5,373435799	0,2566831	0,617190725
5865	ENSG00000164258	-0,527123618	6,144661099	4,321766	0,048911322
5866	ENSG00000164270	-1,058073004	4,091177908	29,519874	1,57726E-05
5867	ENSG00000164284	-0,05492702	4,206537051	0,1896738	0,787341742
5868	ENSG00000164292	0,062229887	7,386888626	0,1787372	0,676354648
5869	ENSG00000164294	0,611235744	4,756873829	9,3045384	0,005656192
5870	ENSG00000164300	0,116812582	4,861125323	0,5944492	0,448514398
5871	ENSG00000164305	-0,001230646	4,643052985	2,071E-05	0,99640813
5872	ENSG00000164306	0,066691491	3,867789202	0,185398	0,670763413
5873	ENSG00000164307	0,179894403	6,208977899	1,2321605	0,278402911
5874	ENSG00000164309	-0,068965466	11,8174728	0,0250447	0,875630321
5875	ENSG00000164318	-0,730915709	3,850598318	10,086309	0,004197409
5876	ENSG00000164323	0,082473183	5,475568688	0,1883163	0,668345538
5877	ENSG00000164327	0,070538585	7,220641544	0,0774386	0,783273349
5878	ENSG00000164329	0,022097171	5,765136392	0,0260401	0,873200367
5879	ENSG00000164330	0,141535112	5,377656921	0,2068392	0,653499127
5880	ENSG00000164331	-0,006297698	3,938123879	0,0014648	0,99029627
5881	ENSG00000164332	-0,102791334	4,32937693	0,5371821	0,609427311
5882	ENSG00000164338	-0,347809239	4,124019797	5,9507669	0,034151184
5883	ENSG00000164346	-0,05482679	5,794290675	0,0656422	0,800057321
5884	ENSG00000164347	-0,355615602	5,516638373	1,8830536	0,183159538
5885	ENSG00000164366	-0,033138175	4,499174632	0,0697437	0,794042692
5886	ENSG00000164402	0,30700641	5,016359802	4,5171247	0,044444545
5887	ENSG00000164405	-0,343918112	7,223793847	2,0087523	0,169734022
5888	ENSG00000164418	0,041353548	4,403253833	0,0165444	0,89876698
5889	ENSG00000164440	-0,018775584	6,926571637	0,003726	0,951851475
5890	ENSG00000164442	0,081848483	5,257454219	0,3902023	0,538290093
5891	ENSG00000164463	-0,004945699	6,566775473	0,0005063	0,982240887
5892	ENSG00000164466	-0,135159945	4,898590519	1,0322448	0,320113954
5893	ENSG00000164494	-0,118744962	4,358937686	1,2859705	0,412175272
5894	ENSG00000164506	0,04935363	5,490987739	0,0844782	0,773910044
5895	ENSG00000164530	0,084542497	5,634250327	0,0504735	0,82421565
5896	ENSG00000164532	0,367043251	5,185598839	1,4052136	0,247897615
5897	ENSG00000164543	0,168876809	4,902751096	1,2886952	0,267924185
5898	ENSG00000164548	0,114638207	5,357232613	0,7633667	0,391233641

5899	ENSG00000164574	0,153076776	4,933377101	0,7146137	0,406582832
5900	ENSG00000164576	0,16787516	5,148486212	1,9446652	0,176377447
5901	ENSG00000164587	0,265858251	8,189291154	1,6443534	0,212441921
5902	ENSG00000164588	-0,265691236	3,913126111	0,5099982	0,482290089
5903	ENSG00000164597	-0,364723029	6,128609459	5,1613831	0,032713693
5904	ENSG00000164603	-0,05986215	5,139730777	0,0473328	0,829680401
5905	ENSG00000164609	0,081281688	5,792554885	0,3959336	0,535345939
5906	ENSG00000164610	-0,190053948	3,826182576	1,8760831	0,307063392
5907	ENSG00000164615	0,209391024	4,590702196	1,4935261	0,233987779
5908	ENSG00000164631	0,040934052	5,509970637	0,1287493	0,722977959
5909	ENSG00000164649	-0,028761827	4,858059134	0,0238777	0,878538005
5910	ENSG00000164654	-0,139790795	4,993915643	1,0807627	0,309247005
5911	ENSG00000164659	0,218947106	4,150976587	1,4141925	0,246435843
5912	ENSG00000164684	0,598857485	6,632273928	9,5514496	0,005142771
5913	ENSG00000164687	-1,085663951	5,649631047	21,572261	0,000111621
5914	ENSG00000164692	0,598006615	8,758110277	2,5972768	0,120620621
5915	ENSG00000164713	-0,160801625	5,122705275	0,9750704	0,333644122
5916	ENSG00000164715	0,295797362	5,983631708	1,7045204	0,204544744
5917	ENSG00000164733	-0,535950374	9,133541075	8,5931508	0,00746179
5918	ENSG00000164741	-0,361199663	6,59846982	5,325674	0,030306082
5919	ENSG00000164742	-0,434914964	4,181833962	2,5263059	0,125548781
5920	ENSG00000164751	-0,149898056	5,293784949	0,7106991	0,407851797
5921	ENSG00000164754	-0,020628749	7,122184893	0,0325178	0,8584622
5922	ENSG00000164808	0,014557828	5,267203313	0,00812	0,928975149
5923	ENSG00000164815	0,089376387	3,795845736	0,3506236	0,587568702
5924	ENSG00000164823	0,434736869	4,627528208	7,2049556	0,013212142
5925	ENSG00000164828	0,050899183	6,782139366	0,0982399	0,756763234
5926	ENSG00000164830	-0,133150509	6,211213478	0,3839745	0,541544906
5927	ENSG00000164880	0,23238925	4,749593249	1,4798694	0,236072458
5928	ENSG00000164889	0,261041111	4,450579201	1,1594595	0,292688976
5929	ENSG00000164896	-0,089474871	5,046574731	0,2055582	0,654499379
5930	ENSG00000164902	-0,116772599	4,913830394	0,8628928	0,442796853
5931	ENSG00000164904	0,015753579	5,294317185	0,0155863	0,901722005
5932	ENSG00000164916	0,336806146	4,918318091	1,5727954	0,222337509
5933	ENSG00000164919	-0,906237628	7,964690747	9,600439	0,005047119
5934	ENSG00000164924	0,031446801	8,071593702	0,0457532	0,832494363
5935	ENSG00000164930	0,089805766	4,812597573	0,3799248	0,543665366
5936	ENSG00000164934	-0,318729482	5,000633721	2,14781	0,156246106
5937	ENSG00000164938	0,207699634	5,652701587	1,0062544	0,326188905
5938	ENSG00000164941	-0,099108465	5,187060991	0,5469508	0,466986162
5939	ENSG00000164944	0,14701908	6,142606311	0,7486678	0,395790738
5940	ENSG00000164946	0,337671219	4,768309631	2,138741	0,157085377
5941	ENSG00000164949	0,638122045	5,499715268	2,9751572	0,097901435
5942	ENSG00000164951	-0,076512957	6,5831389	0,0812281	0,778178218
5943	ENSG00000164953	-0,583437301	5,532283734	5,4910734	0,028080553
5944	ENSG00000164961	0,077239395	5,293952097	0,677304	0,418898153
5945	ENSG00000164970	-0,04695457	4,355720569	0,0732675	0,789039872
5946	ENSG00000164975	-0,154079723	5,417719007	1,5249813	0,229231535
5947	ENSG00000164978	-0,168938703	3,839027457	0,5247348	0,476098297
5948	ENSG00000164983	-0,141858014	5,907181704	0,4842752	0,493431008
5949	ENSG00000164985	0,132105378	6,04748861	1,3300269	0,260547223
5950	ENSG00000164989	0,329861612	3,870931978	3,1840105	0,087496547
5951	ENSG00000165006	-0,189332492	5,399301425	2,289003	0,143801389
5952	ENSG00000165025	0,129999674	3,909970415	0,2697029	0,608468969
5953	ENSG00000165028	-0,550019867	4,76309653	5,991922	0,022379315
5954	ENSG00000165029	-0,399188076	6,716836663	2,2533465	0,146864553
5955	ENSG00000165030	-0,580131706	5,233855122	4,624637	0,042206887
5956	ENSG00000165055	-0,063050225	4,256146784	0,2456118	0,624845205
5957	ENSG00000165060	-0,411513869	3,855027982	4,95607	0,036028866

5958	ENSG00000165071	0,673322089	5,39893906	3,8617569	0,061528537
5959	ENSG00000165072	-0,626662784	5,100380172	6,1523892	0,020835255
5960	ENSG00000165092	0,118735292	5,992046565	0,1118174	0,741099887
5961	ENSG00000165097	0,218647069	4,212173424	2,4978371	0,150759159
5962	ENSG00000165102	0,261758255	5,638937064	2,3100472	0,142102909
5963	ENSG00000165113	-0,194128041	4,021152226	1,8280421	0,256391656
5964	ENSG00000165115	-0,137307245	3,812228399	1,2880821	0,448117913
5965	ENSG00000165119	0,104545114	8,137749553	0,9960848	0,328558837
5966	ENSG00000165121	-0,03638213	3,782287265	0,0835258	0,857122564
5967	ENSG00000165124	0,488633854	6,887874564	1,9527709	0,175559744
5968	ENSG00000165152	-0,071006716	5,510406277	0,1028491	0,751318657
5969	ENSG00000165156	0,06409667	5,988220149	0,1106743	0,742376383
5970	ENSG00000165168	-0,345601088	5,973735527	1,1901474	0,286542579
5971	ENSG00000165169	0,004758347	5,05607503	0,000293	0,986490053
5972	ENSG00000165181	0,050814895	3,695542402	0,0411354	0,841051536
5973	ENSG00000165192	-0,495985544	5,48176341	1,4210584	0,24532553
5974	ENSG00000165195	-0,400602526	3,707887526	4,3734233	0,047687366
5975	ENSG00000165209	-0,177591166	4,839937217	1,9437105	0,176479501
5976	ENSG00000165219	0,033831207	6,127986594	0,0731756	0,789160647
5977	ENSG00000165238	-0,025914219	6,195151087	0,0062498	0,937668782
5978	ENSG00000165240	0,20347076	4,908708127	2,7339788	0,111707263
5979	ENSG00000165264	-0,821157815	5,87231361	10,935741	0,00306461
5980	ENSG00000165269	0,210853463	5,894167519	0,435801	0,515681684
5981	ENSG00000165271	-0,081774444	4,455257995	0,1832516	0,672556262
5982	ENSG00000165280	0,025058552	7,833959503	0,032523	0,858451046
5983	ENSG00000165282	-0,13350016	3,662747177	0,460458	0,504148211
5984	ENSG00000165283	-0,326097645	5,531131003	4,9135977	0,036740366
5985	ENSG00000165288	0,074559406	5,098560198	0,2766192	0,603926456
5986	ENSG00000165312	-0,025357786	5,267508582	0,0044529	0,947370336
5987	ENSG00000165322	0,057779486	6,055398941	0,1159964	0,736494182
5988	ENSG00000165338	0,192053	5,144841568	1,1732056	0,28991429
5989	ENSG00000165355	0,283098923	4,302362299	1,6572113	0,210722735
5990	ENSG00000165359	-0,227341109	5,382478062	1,5333604	0,2280392
5991	ENSG00000165389	-0,44559635	4,512511003	8,5773081	0,007524603
5992	ENSG00000165392	0,057069271	5,010152912	0,2572659	0,616793636
5993	ENSG00000165406	-0,004868881	5,374025634	0,0013009	0,971536946
5994	ENSG00000165410	-0,330026043	8,251075127	0,8710837	0,360303918
5995	ENSG00000165416	0,044240223	6,785485085	0,1232217	0,728734763
5996	ENSG00000165417	0,13901046	5,517287787	1,0705517	0,311488961
5997	ENSG00000165424	0,302960526	5,732478183	2,9221505	0,100766029
5998	ENSG00000165434	-0,269494425	3,63936462	0,9631176	0,336564518
5999	ENSG00000165457	-0,274660297	4,261219394	1,4963556	0,23355878
6000	ENSG00000165458	0,123035682	5,746599587	0,3470811	0,561484813
6001	ENSG00000165475	0,149611416	4,071470447	1,1939938	0,391539952
6002	ENSG00000165476	0,131033687	5,45109316	0,4518855	0,508105806
6003	ENSG00000165487	-0,434657157	5,902144018	7,4020173	0,012161515
6004	ENSG00000165494	0,194098899	6,107643797	2,076833	0,162915525
6005	ENSG00000165502	-0,193940808	6,774259921	0,5373316	0,470910509
6006	ENSG00000165507	-0,968259581	6,026242764	7,6628377	0,010910274
6007	ENSG00000165512	-0,408835101	4,303934951	3,1314368	0,089989916
6008	ENSG00000165516	-0,169379876	5,705953749	2,0934948	0,161308158
6009	ENSG00000165525	-0,04785041	5,977837208	0,1297618	0,721938753
6010	ENSG00000165526	-0,220182327	4,469781202	4,0468432	0,13180094
6011	ENSG00000165527	0,046549583	5,352921997	0,0929296	0,763213462
6012	ENSG00000165533	0,30909693	3,761499461	5,2126546	0,088832213
6013	ENSG00000165572	-0,044106037	4,401973643	0,0745721	0,787217683
6014	ENSG00000165609	0,018964333	4,26271434	0,0175415	0,895776772
6015	ENSG00000165626	-0,019811636	3,772644384	0,0123765	0,912378777
6016	ENSG00000165629	-0,770250536	8,52381778	9,5271444	0,005190965

6017	ENSG00000165633	0,260209727	5,353368451	1,4439054	0,24167674
6018	ENSG00000165637	-0,404656814	7,650803153	6,8943005	0,015053468
6019	ENSG00000165650	0,007410879	5,613981681	0,0039519	0,950413436
6020	ENSG00000165660	-0,258184589	4,432556559	2,157507	0,155354673
6021	ENSG00000165669	-0,083296615	5,560070189	0,3901424	0,538321036
6022	ENSG00000165671	0,028085003	6,601258372	0,0705011	0,792954291
6023	ENSG00000165672	-0,359331309	7,357814299	2,7504027	0,110741318
6024	ENSG00000165675	0,075609919	4,233540077	0,5408566	0,564542679
6025	ENSG00000165678	-0,394146326	8,35714807	2,6903537	0,114498039
6026	ENSG00000165688	-0,038992121	5,105269149	0,0628261	0,804298368
6027	ENSG00000165699	0,074616513	6,025440868	0,187054	0,669388637
6028	ENSG00000165704	-0,06882783	3,867716037	0,1819727	0,741558326
6029	ENSG00000165714	-0,073790324	3,683341223	0,2289423	0,672342721
6030	ENSG00000165732	-0,332325328	6,166283365	2,4674734	0,129814896
6031	ENSG00000165733	-0,132243881	5,657189986	1,4154141	0,246196092
6032	ENSG00000165757	0,137113654	6,499634426	0,6055693	0,44435474
6033	ENSG00000165775	-0,521240012	7,261039831	9,945757	0,004425816
6034	ENSG00000165792	-0,185401903	4,270102532	2,4058043	0,202524372
6035	ENSG00000165795	-0,180823085	7,038754775	1,5858675	0,220447857
6036	ENSG00000165801	0,38851406	4,70832527	2,6150268	0,119423911
6037	ENSG00000165806	0,241762132	4,292746714	1,3097495	0,264153951
6038	ENSG00000165810	-0,211018077	5,708554587	0,3632925	0,552551955
6039	ENSG00000165813	-0,027263132	5,950577018	0,029009	0,866240406
6040	ENSG00000165819	-0,034763767	4,959977371	0,1551347	0,697273708
6041	ENSG00000165832	0,06832012	4,991945392	0,0611794	0,806825455
6042	ENSG00000165861	0,211483592	4,033491878	1,7108527	0,231400396
6043	ENSG00000165868	0,127340987	4,8943551	0,4126665	0,526940455
6044	ENSG00000165874	-0,342061566	3,759650454	3,6859223	0,080985861
6045	ENSG00000165895	-0,105148008	5,019877459	0,383407	0,541841374
6046	ENSG00000165898	-0,041928571	4,235228405	0,0535112	0,819098053
6047	ENSG00000165899	-0,135972589	5,417302016	0,0775432	0,783130899
6048	ENSG00000165912	0,179806495	5,938094633	0,4759928	0,497112644
6049	ENSG00000165914	0,019786787	4,714010555	0,0122922	0,912676431
6050	ENSG00000165916	-0,238045725	6,357166787	1,6602409	0,210320196
6051	ENSG00000165929	-0,342355765	5,246963637	1,1807941	0,28839755
6052	ENSG00000165934	-0,051022512	5,767713354	0,1669345	0,686598464
6053	ENSG00000165943	0,408469959	5,745160962	5,7016837	0,02550635
6054	ENSG00000165949	-0,154506659	5,571129648	0,7045614	0,409853586
6055	ENSG00000165959	-0,550841638	4,775623515	2,6006533	0,120391891
6056	ENSG00000165983	-0,410012383	4,497358723	4,4763125	0,045351147
6057	ENSG00000165995	-0,478341674	5,586678488	4,6311545	0,042074468
6058	ENSG00000165996	-0,423534896	5,702793837	3,2359752	0,085110088
6059	ENSG00000165997	0,105428143	5,52507914	0,1671696	0,686403356
6060	ENSG00000166004	-0,12520436	4,69262795	0,8094724	0,417094043
6061	ENSG00000166012	0,066717707	5,754547423	0,1083413	0,745004541
6062	ENSG00000166016	0,109555284	4,511830234	0,2234323	0,640870116
6063	ENSG00000166024	-0,028814866	4,186698641	0,0538343	0,924064804
6064	ENSG00000166025	0,121737159	6,990967994	0,6761718	0,419289277
6065	ENSG00000166033	0,673718398	6,388869646	15,801031	0,000594031
6066	ENSG00000166037	-0,130805368	5,553611815	0,431838	0,517579358
6067	ENSG00000166046	-0,103742925	5,473435603	0,1975096	0,660872094
6068	ENSG00000166068	0,165181421	5,798101887	0,801248	0,379948218
6069	ENSG00000166086	0,153725645	5,660078339	1,1002194	0,305047653
6070	ENSG00000166091	-1,082353587	4,899197686	10,652219	0,003400247
6071	ENSG00000166106	-0,623915861	4,703840635	3,1109371	0,090984319
6072	ENSG00000166123	-0,834859389	4,483198505	4,3407639	0,048457111
6073	ENSG00000166128	0,094673221	5,413889385	0,1278681	0,723897509
6074	ENSG00000166135	-0,057964104	5,788245109	0,1679133	0,685736108
6075	ENSG00000166136	-0,363445247	4,759501295	4,1278645	0,053833134

6076	ENSG00000166147	-0,067073388	8,60719059	0,1028337	0,751336575
6077	ENSG00000166164	0,041233505	5,517221917	0,2565876	0,617255877
6078	ENSG00000166165	-0,444758526	7,062202985	1,6592316	0,210454191
6079	ENSG00000166167	0,166185627	5,650441942	1,7343478	0,200720654
6080	ENSG00000166170	0,050074062	5,234856994	0,2049854	0,654933213
6081	ENSG00000166173	-0,46844701	4,879785817	6,9977013	0,014426603
6082	ENSG00000166181	0,039200054	5,770421942	0,1101574	0,742945568
6083	ENSG00000166197	-0,038193187	5,420656874	0,0889953	0,768114566
6084	ENSG00000166199	-0,20794014	3,708069485	2,6675531	0,280724586
6085	ENSG00000166200	-0,306701655	6,795050811	1,2037142	0,28388006
6086	ENSG00000166206	-1,099231876	5,601274737	12,809891	0,001580458
6087	ENSG00000166224	0,027762016	5,025732811	0,0180795	0,89420277
6088	ENSG00000166225	-0,071401942	5,619442494	0,231108	0,635222813
6089	ENSG00000166226	-0,332532413	6,332145366	2,9970304	0,096747001
6090	ENSG00000166228	-0,160471866	5,208570097	1,0695402	0,311749591
6091	ENSG00000166233	0,014686246	6,683393955	0,0162243	0,899741653
6092	ENSG00000166250	0,408369788	5,246116649	0,9273929	0,345509264
6093	ENSG00000166260	-0,124363129	5,167582294	0,6539794	0,426939785
6094	ENSG00000166263	0,104049903	4,37001262	0,7264003	0,474327632
6095	ENSG00000166265	-0,49079114	4,815391727	7,6991562	0,010747614
6096	ENSG00000166266	-0,250181878	6,370368611	1,1842323	0,287713823
6097	ENSG00000166272	0,21208137	5,488930785	1,0569137	0,314559526
6098	ENSG00000166275	-0,049207084	4,706908506	0,0546021	0,817297642
6099	ENSG00000166295	-0,300399249	7,254985851	3,8854066	0,060770674
6100	ENSG00000166311	-0,19805783	4,872698148	1,0425428	0,317799932
6101	ENSG00000166313	-0,169485244	5,416745638	0,8316851	0,371200181
6102	ENSG00000166317	0,264866539	7,76896772	0,6850069	0,4163321
6103	ENSG00000166326	0,111297312	6,297454782	1,0336208	0,319798341
6104	ENSG00000166340	-0,207045497	5,623139933	1,561012	0,224022165
6105	ENSG00000166341	0,50929599	4,537927366	1,8495134	0,186958946
6106	ENSG00000166347	-0,243471502	5,406169701	0,8660276	0,361676296
6107	ENSG00000166348	0,144560726	5,237014771	0,3603431	0,554156562
6108	ENSG00000166352	-0,633104093	4,543766279	5,4513282	0,02859818
6109	ENSG00000166377	0,130004348	5,046348134	1,5128802	0,261316968
6110	ENSG00000166387	0,077614089	4,383708505	0,2575019	0,62580222
6111	ENSG00000166398	0,052652173	5,248585046	0,110868	0,742159474
6112	ENSG00000166402	0,110735433	4,825494845	0,2696673	0,608492446
6113	ENSG00000166405	-0,170347263	4,558908614	0,9279443	0,345368696
6114	ENSG00000166411	-0,499639318	6,442712749	2,8307216	0,105939459
6115	ENSG00000166432	0,408822584	4,903367497	4,8514121	0,037862568
6116	ENSG00000166435	0,278313974	3,880408853	1,7111464	0,203697576
6117	ENSG00000166439	0,14759324	5,121594272	1,177007	0,289118416
6118	ENSG00000166441	0,138795257	8,248828623	0,5787974	0,454461396
6119	ENSG00000166444	-0,162652919	4,808314519	0,4401301	0,513622967
6120	ENSG00000166454	0,15383216	5,690255177	2,7391896	0,111384714
6121	ENSG00000166471	-0,033022382	4,80402027	0,0486919	0,827292943
6122	ENSG00000166473	0,267854526	4,093384028	0,620489	0,438869726
6123	ENSG00000166477	-0,097978311	3,986011026	0,857462	0,58053949
6124	ENSG00000166478	-0,312242544	4,367923538	3,1887828	0,087274188
6125	ENSG00000166479	-0,13219115	6,13567703	0,5153768	0,480014536
6126	ENSG00000166482	0,410218947	6,2636569	2,1583892	0,155273873
6127	ENSG00000166483	0,208271996	5,994777736	0,8076954	0,378070201
6128	ENSG00000166503	-0,020599199	5,867405598	0,0295847	0,864927905
6129	ENSG00000166508	0,199448186	4,421435679	1,0325749	0,320074433
6130	ENSG00000166510	-0,106089041	3,717057816	0,4979861	0,55758215
6131	ENSG00000166526	-0,066420773	4,50301431	0,3259358	0,57355259
6132	ENSG00000166532	-0,088582642	5,40159771	0,4368016	0,515181876
6133	ENSG00000166548	0,447773431	4,692138329	6,1138511	0,021194965
6134	ENSG00000166557	-0,065635424	4,768166604	0,135234	0,716412018

6135	ENSG00000166562	-0,215944162	4,121636557	1,0187339	0,323269935
6136	ENSG00000166575	0,187906964	5,522332903	0,7031906	0,410302708
6137	ENSG00000166579	0,014038852	5,141544793	0,0060908	0,938464961
6138	ENSG00000166592	-0,170403229	6,495745561	0,1968552	0,661397141
6139	ENSG00000166595	-0,29517041	5,063550727	2,264558	0,145908097
6140	ENSG00000166598	-0,193467016	8,781036203	0,6849724	0,416343666
6141	ENSG00000166619	-0,199396034	6,010935585	2,0445158	0,166093001
6142	ENSG00000166669	0,074457313	5,095832851	0,1793694	0,67583092
6143	ENSG00000166681	-0,349583194	6,322711916	4,1053443	0,05444026
6144	ENSG00000166685	-0,099993215	4,530770877	0,3867166	0,540116672
6145	ENSG00000166689	-0,218964215	5,232555212	0,5121602	0,48137321
6146	ENSG00000166704	-0,022185268	4,205907215	0,0251248	0,875429801
6147	ENSG00000166710	0,078296683	10,21122167	0,1052631	0,748513894
6148	ENSG00000166716	0,193641914	4,829741007	0,8877275	0,355838557
6149	ENSG00000166734	0,463066834	6,275450713	4,4572807	0,045773373
6150	ENSG00000166741	-0,286646598	6,082692046	0,7294923	0,401813818
6151	ENSG00000166747	0,003739453	6,274926626	0,0014386	0,970069097
6152	ENSG00000166750	0,243460464	6,082818557	1,2922012	0,267291489
6153	ENSG00000166770	-0,151300235	4,187204471	1,3560804	0,353408804
6154	ENSG00000166780	0,114020209	5,065983555	0,5995444	0,44659826
6155	ENSG00000166783	0,193347865	5,943492385	1,5976668	0,218833746
6156	ENSG00000166794	0,097347011	5,034302853	0,2320351	0,63454855
6157	ENSG00000166797	-0,438601588	4,789139064	5,4896299	0,028099169
6158	ENSG00000166801	0,050391189	4,533591468	0,068696	0,795566818
6159	ENSG00000166816	-0,093068817	4,552066966	0,1075905	0,745857046
6160	ENSG00000166819	1,231246828	6,757347317	2,1135499	0,159445271
6161	ENSG00000166822	0,242330743	4,051394783	2,2446955	0,147607705
6162	ENSG00000166831	-0,454654313	6,725430255	2,7838723	0,108710076
6163	ENSG00000166833	0,36018159	5,312587561	2,433911	0,132325358
6164	ENSG00000166847	-0,018962286	5,65385978	0,0370543	0,875693614
6165	ENSG00000166848	0,384533241	5,708653607	14,523145	0,000888407
6166	ENSG00000166855	-0,089681082	5,767487915	0,5941368	0,448605124
6167	ENSG00000166881	0,360391322	3,710813073	6,0906241	0,052798828
6168	ENSG00000166887	0,190029593	5,926539159	1,4901826	0,234495988
6169	ENSG00000166888	0,205027809	6,0399454	0,9833561	0,331640372
6170	ENSG00000166889	-0,058450608	4,52462811	0,1143913	0,738252045
6171	ENSG00000166900	-0,135730559	4,594952415	0,8004236	0,380189329
6172	ENSG00000166902	-0,310916467	5,108872442	4,2539703	0,050570084
6173	ENSG00000166908	0,091963791	4,112172886	0,4127659	0,654484922
6174	ENSG00000166912	-0,174010659	5,478541676	1,4690266	0,237727288
6175	ENSG00000166913	0,058063506	7,244204963	0,3084583	0,583945457
6176	ENSG00000166927	0,088806377	4,859470173	0,0686319	0,795659864
6177	ENSG00000166938	-0,00377289	5,330026099	0,0010226	0,974762898
6178	ENSG00000166946	0,192148068	5,785485977	3,3307959	0,080900702
6179	ENSG00000166949	-0,382318252	5,695751891	2,7564187	0,110372959
6180	ENSG00000166963	-0,070000119	6,632932058	0,0481256	0,828283466
6181	ENSG00000166971	-0,590146636	5,670735622	18,51252	0,000260862
6182	ENSG00000166974	0,143847641	6,792967645	0,8267353	0,372602707
6183	ENSG00000166979	-0,400592263	4,31780146	3,8786865	0,061003308
6184	ENSG00000166986	-0,174721999	5,689034739	1,4462938	0,241276684
6185	ENSG00000167004	-0,0295681	7,409410835	0,0272458	0,870329571
6186	ENSG00000167005	0,095582816	6,410350924	0,2883423	0,596416672
6187	ENSG00000167037	0,20223758	5,110368458	0,3671321	0,550476362
6188	ENSG00000167074	1,23415272	4,897620102	18,641909	0,000252757
6189	ENSG00000167081	0,0088689	5,856953256	0,0050055	0,944203381
6190	ENSG00000167085	-0,359244237	5,58199936	4,6074964	0,042557389
6191	ENSG00000167088	-0,338612101	4,366009284	5,5268848	0,027597414
6192	ENSG00000167107	0,151279184	4,88764731	0,410729	0,527903695
6193	ENSG00000167110	0,271493415	5,907384492	2,9795258	0,097669594

6194	ENSG00000167112	-0,082163854	4,542433019	0,2925655	0,59374995
6195	ENSG00000167113	0,141318557	4,201626326	0,8632841	0,450380362
6196	ENSG00000167118	-0,127903846	4,652748388	0,6707207	0,42116504
6197	ENSG00000167186	-0,100828997	4,610074579	0,4836257	0,493701437
6198	ENSG00000167191	-0,082844456	5,35454638	0,1275051	0,724272611
6199	ENSG00000167193	0,142363929	5,803075967	1,4130335	0,246582204
6200	ENSG00000167196	-0,143225024	4,814939952	1,2161499	0,29779352
6201	ENSG00000167202	0,663866863	5,427564401	4,2937683	0,049589496
6202	ENSG00000167232	0,017943952	8,182771815	0,0071669	0,933262539
6203	ENSG00000167257	0,144382249	3,691722403	0,9326847	0,458768482
6204	ENSG00000167258	0,030391664	5,310373516	0,0357573	0,851668224
6205	ENSG00000167272	0,000232761	4,058725728	2,749E-06	0,998691271
6206	ENSG00000167283	-0,469109535	6,008531393	3,1037476	0,091336069
6207	ENSG00000167291	0,118720634	4,835190617	0,1484061	0,70358574
6208	ENSG00000167306	0,788987268	4,056350768	6,7974357	0,015718506
6209	ENSG00000167315	-0,167815489	6,628942753	1,075255	0,310489036
6210	ENSG00000167323	0,014769595	4,220757066	0,0048579	0,94503304
6211	ENSG00000167325	-0,122088747	5,274952534	0,4599108	0,504399191
6212	ENSG00000167378	0,093782205	5,361365245	0,2204792	0,6430747
6213	ENSG00000167380	0,031455797	4,805298052	0,0611757	0,806823617
6214	ENSG00000167384	0,02118883	3,611813082	0,0283767	0,902517776
6215	ENSG00000167393	0,165705072	5,299296849	0,607741	0,443549589
6216	ENSG00000167447	0,044612597	3,762468105	0,1762417	0,800516136
6217	ENSG00000167460	-0,197589735	7,498314887	0,9673884	0,335516958
6218	ENSG00000167468	0,116626972	6,569507178	0,2305953	0,635596381
6219	ENSG00000167470	-0,073080752	5,011723477	0,1066325	0,746949678
6220	ENSG00000167491	-0,01556235	4,470295603	0,0068645	0,934682199
6221	ENSG00000167515	0,173023133	4,322820414	1,2627724	0,272663499
6222	ENSG00000167522	0,10939581	5,85598051	0,2132133	0,648577068
6223	ENSG00000167526	0,338708334	8,50684378	2,3390532	0,139738278
6224	ENSG00000167528	0,085044652	4,928031299	0,664911	0,442630605
6225	ENSG00000167548	0,214990738	5,544165267	0,3413272	0,564723485
6226	ENSG00000167549	0,019965101	5,696459226	0,014106	0,906484909
6227	ENSG00000167552	-0,182061014	7,365037579	1,0715922	0,311296167
6228	ENSG00000167553	-0,265099232	5,910278813	2,5074227	0,126899759
6229	ENSG00000167555	0,043242172	4,759466963	0,0815426	0,777761261
6230	ENSG00000167562	-0,019132792	3,768104479	0,0231594	0,950082907
6231	ENSG00000167565	0,154691229	3,969181005	0,9695591	0,418805216
6232	ENSG00000167588	0,897930177	6,275343894	1,2800919	0,269485015
6233	ENSG00000167601	0,003471791	5,197231113	0,0001797	0,989418715
6234	ENSG00000167613	-0,056283172	3,823688471	0,035946	0,851282161
6235	ENSG00000167615	0,472952629	6,291264246	2,9521429	0,099133394
6236	ENSG00000167632	-0,014538353	4,538239113	0,0055273	0,941374882
6237	ENSG00000167635	-0,118976365	6,100841745	0,5655916	0,459606825
6238	ENSG00000167637	-0,049565733	4,613104765	0,0839329	0,774619837
6239	ENSG00000167657	0,268747055	5,208122034	1,3065573	0,264721104
6240	ENSG00000167658	0,507089215	9,757485846	6,0789141	0,021497595
6241	ENSG00000167671	0,111503758	5,664664055	0,4511674	0,508439775
6242	ENSG00000167676	0,844033327	8,142363328	2,744641	0,111095451
6243	ENSG00000167699	0,047187726	4,832081587	0,1306451	0,72103593
6244	ENSG00000167721	-0,035236138	4,825911193	0,1115324	0,777755843
6245	ENSG00000167766	0,281263248	5,379983967	4,341965	0,048390532
6246	ENSG00000167770	-0,092826182	4,663196086	0,3253653	0,573904996
6247	ENSG00000167778	0,289744521	5,125498122	2,5521824	0,12372494
6248	ENSG00000167779	-0,59958587	5,546262812	1,7369497	0,200439696
6249	ENSG00000167785	0,001392225	4,020201278	0,0001489	0,995797004
6250	ENSG00000167792	-0,124769455	7,341503455	0,2835046	0,599495324
6251	ENSG00000167815	-0,241459998	6,883533093	1,1543706	0,293725183
6252	ENSG00000167842	0,126026957	4,113828841	0,6720984	0,420695209

6253	ENSG00000167862	-0,454768071	4,029076168	12,621387	0,00817143
6254	ENSG00000167863	-0,573724218	7,848095626	4,3603108	0,047994742
6255	ENSG00000167881	-0,074407548	5,48253668	0,2927308	0,593636459
6256	ENSG00000167904	0,115493534	3,645310154	0,8456656	0,513639927
6257	ENSG00000167930	-0,345212525	5,92708122	2,2564819	0,146596317
6258	ENSG00000167969	-0,244477714	4,944858916	1,6120925	0,216833302
6259	ENSG00000167978	0,096297541	8,265398017	0,1060329	0,747636206
6260	ENSG00000167986	0,171008993	7,235181855	2,0541855	0,165131466
6261	ENSG00000167996	0,07891646	6,794967751	0,2452266	0,625131626
6262	ENSG00000168002	0,137256632	4,273585672	1,1874963	0,360417894
6263	ENSG00000168003	0,000338999	4,9052709	4,228E-06	0,998376996
6264	ENSG00000168014	0,050878253	4,66472991	0,1343622	0,86308079
6265	ENSG00000168016	0,697799594	5,212372485	8,0496621	0,009308284
6266	ENSG00000168028	0,174205876	8,217985372	0,6065549	0,443989033
6267	ENSG00000168036	0,076695607	8,034055969	0,5610592	0,461363351
6268	ENSG00000168038	-0,704545732	4,648711937	17,820272	0,000321445
6269	ENSG00000168056	0,544563592	5,90872609	6,3193179	0,019353579
6270	ENSG00000168066	-0,036029783	6,446212256	0,0441045	0,835499583
6271	ENSG00000168079	-0,87010161	6,795563203	6,6629006	0,016658199
6272	ENSG00000168090	-0,220921566	5,58000317	2,2867078	0,144012435
6273	ENSG00000168092	0,045375346	6,247130184	0,1880162	0,668579162
6274	ENSG00000168116	-0,07225516	4,225499972	0,1016767	0,752690551
6275	ENSG00000168118	-0,280619685	5,756869125	4,1185663	0,054045247
6276	ENSG00000168137	-0,00784028	6,417934923	0,0015701	0,968732713
6277	ENSG00000168152	-0,159000393	3,538536456	0,4389206	0,514196666
6278	ENSG00000168159	0,001303488	5,342985668	5,461E-05	0,994167341
6279	ENSG00000168172	0,331102894	6,689877041	4,8573517	0,037733777
6280	ENSG00000168175	-0,04931377	6,269898434	0,1249165	0,72695445
6281	ENSG00000168214	-0,264082404	6,45573197	4,2238525	0,051289068
6282	ENSG00000168216	-0,047929147	5,920348644	0,133335	0,718307639
6283	ENSG00000168228	-0,099897053	4,003944578	0,5839773	0,556334505
6284	ENSG00000168234	0,468856955	3,654183784	3,0112956	0,09600262
6285	ENSG00000168246	0,227914847	4,313906487	1,7139756	0,203337169
6286	ENSG00000168256	-0,186057135	4,646802089	1,214185	0,281847534
6287	ENSG00000168259	-0,185911047	5,702728946	4,2268414	0,051213283
6288	ENSG00000168264	0,250917688	6,215644343	4,7744796	0,039239839
6289	ENSG00000168268	-0,888643371	5,714517113	8,1004516	0,009117962
6290	ENSG00000168273	-0,333846669	3,973412724	3,9030737	0,060255656
6291	ENSG00000168283	0,142725137	5,017442718	0,6958716	0,412713396
6292	ENSG00000168286	-0,151187187	4,021999972	1,2777063	0,359348179
6293	ENSG00000168288	-0,406871038	6,434808918	2,5608172	0,123123295
6294	ENSG00000168291	-0,490722665	6,645479054	5,3632407	0,029783816
6295	ENSG00000168297	0,31794795	4,603472512	5,7684767	0,024714479
6296	ENSG00000168298	-0,133235644	4,802641671	0,1032492	0,7508525
6297	ENSG00000168300	-0,098871608	7,221931063	0,2097963	0,651204298
6298	ENSG00000168303	-0,081442607	3,82650972	0,2535786	0,621025
6299	ENSG00000168306	0,017366901	4,197534618	0,0127189	0,974914492
6300	ENSG00000168309	-1,010479441	5,442415869	10,317467	0,003849389
6301	ENSG00000168310	0,129295464	4,315429073	1,1091808	0,420174211
6302	ENSG00000168334	-0,175691265	8,320825684	0,1396538	0,712031415
6303	ENSG00000168374	0,266623252	6,526283432	2,8110697	0,107091496
6304	ENSG00000168385	0,251987658	7,921892347	4,8049823	0,038672098
6305	ENSG00000168386	-0,401061691	7,10957232	4,5080975	0,04465574
6306	ENSG00000168394	0,136412997	4,128117376	0,406014	0,530261254
6307	ENSG00000168395	-0,066088837	4,503832491	0,2096945	0,65127165
6308	ENSG00000168397	0,05053749	4,553707961	0,1238488	0,820646273
6309	ENSG00000168405	0,084683009	5,090566962	0,1361733	0,715474217
6310	ENSG00000168411	0,315590799	3,67978976	3,1715071	0,106620674
6311	ENSG00000168434	0,314454035	3,851213244	6,0269818	0,097089438

6312	ENSG00000168438	-0,096766956	5,061036248	0,3830165	0,54204556
6313	ENSG00000168439	-0,393132315	6,108021586	5,298157	0,03069513
6314	ENSG00000168461	0,669776736	5,668680457	5,7461264	0,02499744
6315	ENSG00000168477	0,291742308	7,177842713	0,5645657	0,460009339
6316	ENSG00000168488	0,133215507	4,728067113	0,2759981	0,604340207
6317	ENSG00000168495	0,079762742	4,122477058	0,2767377	0,661187661
6318	ENSG00000168497	0,018200461	7,264042238	0,0082785	0,9282873
6319	ENSG00000168509	-0,467946474	5,605432067	2,8177983	0,106695414
6320	ENSG00000168522	-0,141088461	5,610710959	1,2011038	0,284350792
6321	ENSG00000168538	0,007083272	5,607447464	0,0039725	0,95028456
6322	ENSG00000168542	1,159180645	8,977966774	5,7533447	0,024915857
6323	ENSG00000168564	0,091014815	4,147584099	0,4401754	0,513582709
6324	ENSG00000168566	-0,106644428	4,679094965	0,5498021	0,465855902
6325	ENSG00000168575	-0,103259436	6,692904898	0,2565696	0,617284765
6326	ENSG00000168591	0,030370611	4,32878907	0,0634533	0,907012234
6327	ENSG00000168610	-0,29514307	7,432420342	2,0059775	0,170017197
6328	ENSG00000168615	-0,108726617	6,742770929	0,3352797	0,568167464
6329	ENSG00000168653	-0,712238971	7,691045138	8,1767512	0,00884006
6330	ENSG00000168672	0,55578711	4,151978365	4,2179182	0,05148082
6331	ENSG00000168675	-0,007806361	5,575043053	0,0021761	0,963194252
6332	ENSG00000168679	0,283845771	3,564056967	2,2810492	0,147028432
6333	ENSG00000168702	-1,287682625	3,811506447	9,6816421	0,004892846
6334	ENSG00000168710	0,189560475	6,094659862	1,4172688	0,245937561
6335	ENSG00000168724	-0,424155467	6,291394409	11,469143	0,002518657
6336	ENSG00000168734	-0,140716423	6,494831915	0,6214149	0,43853286
6337	ENSG00000168758	0,351342347	4,481908682	3,0252082	0,095283021
6338	ENSG00000168763	0,279374222	3,957245343	1,9419681	0,176711783
6339	ENSG00000168765	-0,449619544	4,293023207	7,2068633	0,013201502
6340	ENSG00000168769	-0,2240545	6,05459878	3,5385784	0,072569328
6341	ENSG00000168781	0,201968585	4,823830349	2,0037677	0,170215794
6342	ENSG00000168795	0,240410714	3,814610106	3,3440185	0,199910097
6343	ENSG00000168803	-0,317599781	5,066032095	2,1714127	0,154086985
6344	ENSG00000168807	0,490844996	5,74652113	2,7699088	0,109552165
6345	ENSG00000168813	0,088071001	5,367896932	0,8995125	0,352690457
6346	ENSG00000168818	-0,102789704	4,179360393	1,3209548	0,504103592
6347	ENSG00000168827	-0,228331637	6,135199883	1,9908124	0,171575002
6348	ENSG00000168874	0,627179559	4,492553774	3,1794905	0,08770775
6349	ENSG00000168883	-0,028659486	4,632855781	0,1123813	0,809904821
6350	ENSG00000168887	0,040179071	4,539459623	0,1748899	0,820916614
6351	ENSG00000168894	-0,100390723	4,858473132	0,2623033	0,613401513
6352	ENSG00000168904	-0,138071409	4,242978552	3,0113602	0,356969089
6353	ENSG00000168906	-0,24725388	7,305340779	1,0314246	0,320338345
6354	ENSG00000168916	0,168347925	5,647055719	0,8258154	0,372864216
6355	ENSG00000168918	0,258435367	3,743423848	1,4171515	0,245956535
6356	ENSG00000168924	-0,076138041	4,996140604	0,1351887	0,716457299
6357	ENSG00000168936	0,107670717	4,89026111	0,4967588	0,48796992
6358	ENSG00000168938	-0,357018524	4,68127038	10,185612	0,00776901
6359	ENSG00000168944	0,168122711	4,983708036	2,3934244	0,135383625
6360	ENSG00000168952	-0,657845397	4,233777388	9,5403197	0,005164779
6361	ENSG00000168958	-0,262707751	6,004779967	6,0559132	0,021719236
6362	ENSG00000168994	-0,084891963	4,546370609	0,153816	0,698508151
6363	ENSG00000169016	0,038714071	4,511399128	0,085611	0,793114525
6364	ENSG00000169018	-0,218540033	6,917607282	2,2619257	0,146098996
6365	ENSG00000169019	-0,05425571	4,207118742	0,0384704	0,846215838
6366	ENSG00000169020	-0,568104059	6,230531678	6,1304413	0,021039275
6367	ENSG00000169021	-0,59951296	7,790943975	10,199305	0,004023157
6368	ENSG00000169031	-0,096590767	4,540920433	0,1118605	0,74105189
6369	ENSG00000169032	-0,076177296	4,672235094	0,3406634	0,5650799
6370	ENSG00000169045	0,072650116	7,859925415	0,1694802	0,684366971

6371	ENSG00000169047	0,186256383	4,41800891	1,1326364	0,298206463
6372	ENSG00000169057	0,100925618	5,80735136	0,2298327	0,63615299
6373	ENSG00000169062	0,172574705	4,939720724	2,026432	0,170039102
6374	ENSG00000169083	-0,084832478	5,303614978	0,28518	0,598425315
6375	ENSG00000169084	-0,259517246	4,135768573	3,6685132	0,105525286
6376	ENSG00000169087	0,088513027	3,60704205	0,4835111	0,668235185
6377	ENSG00000169100	0,319483299	6,518361318	3,1609847	0,088578619
6378	ENSG00000169116	0,197602089	7,57375559	0,6919788	0,414004407
6379	ENSG00000169118	0,239719152	3,945939195	3,1281192	0,155242526
6380	ENSG00000169122	0,138836042	4,473526885	0,7164282	0,405996662
6381	ENSG00000169139	-0,123011423	5,009078263	0,4932692	0,489486023
6382	ENSG00000169155	-0,135411252	5,248527574	0,7540045	0,394138503
6383	ENSG00000169180	0,069004267	5,218341503	0,1420294	0,709709737
6384	ENSG00000169184	0,063933321	4,132409593	0,0596033	0,809278155
6385	ENSG00000169189	0,267925892	4,255497814	2,2281065	0,149045372
6386	ENSG00000169193	0,05352796	3,859066197	0,0373472	0,848448044
6387	ENSG00000169203	0,043073938	5,619704049	0,0315875	0,860484566
6388	ENSG00000169217	0,170144616	5,08833913	0,9631708	0,336551431
6389	ENSG00000169223	0,000402058	5,217118482	7,242E-06	0,997875973
6390	ENSG00000169231	0,572669502	3,871945289	4,9996896	0,035294198
6391	ENSG00000169239	0,095711855	3,690147213	0,2188492	0,644299295
6392	ENSG00000169242	0,400530108	4,29671567	5,8850569	0,023478102
6393	ENSG00000169246	0,031291829	5,905469127	0,0147098	0,904514308
6394	ENSG00000169247	0,227171415	3,977773499	0,8260145	0,372807594
6395	ENSG00000169251	-0,180339139	6,23366751	0,6927656	0,413742982
6396	ENSG00000169255	-0,365262821	6,249744376	1,7645584	0,197025073
6397	ENSG00000169271	-0,644571872	6,9016055	4,9602379	0,03595793
6398	ENSG00000169282	0,058791849	3,965671768	0,1397453	0,711941571
6399	ENSG00000169288	-0,595220403	4,870237535	4,533031	0,044118641
6400	ENSG00000169291	-0,13607958	5,068741092	0,7069822	0,409062281
6401	ENSG00000169299	0,24795347	4,594626397	0,9820432	0,331956751
6402	ENSG00000169359	0,086655134	4,651754238	0,2657504	0,611092833
6403	ENSG00000169372	-0,257728293	4,597479438	2,7581342	0,110268187
6404	ENSG00000169375	0,194322471	5,386051473	1,3390671	0,259018294
6405	ENSG00000169379	0,214324798	3,78240241	1,4092424	0,247240351
6406	ENSG00000169398	0,039081199	6,803099526	0,1627507	0,690331569
6407	ENSG00000169410	0,18160938	4,258245261	1,3409028	0,258701064
6408	ENSG00000169418	0,245848037	4,847841756	0,4734331	0,498260072
6409	ENSG00000169432	-0,912739551	3,85933498	10,154365	0,00409149
6410	ENSG00000169439	0,0742612	6,28887695	0,1809056	0,674530171
6411	ENSG00000169446	-0,070874899	5,357912876	0,2361474	0,631571537
6412	ENSG00000169490	-0,194944434	4,564563315	1,958073	0,192020058
6413	ENSG00000169499	-0,031323138	6,072729754	0,0395654	0,844072141
6414	ENSG00000169504	-0,400203882	8,656448802	3,0307701	0,0949971
6415	ENSG00000169508	-0,191946193	3,688981674	0,4210899	0,522789899
6416	ENSG00000169515	0,304071579	4,859031555	2,8908582	0,102502851
6417	ENSG00000169519	-0,064521464	4,6954978	0,1815908	0,673952107
6418	ENSG00000169554	0,038111529	7,715461448	0,1040462	0,749916711
6419	ENSG00000169567	-0,440310747	6,541355169	8,7166185	0,007111674
6420	ENSG00000169570	-0,086564177	4,352701862	0,1860807	0,670195736
6421	ENSG00000169599	-0,456777333	5,109360629	6,0800683	0,021515975
6422	ENSG00000169604	0,744887515	6,57802109	8,9725816	0,00643737
6423	ENSG00000169609	-0,0323753	3,673117964	0,0623742	0,83941354
6424	ENSG00000169612	-0,267743168	3,697084544	1,8970953	0,190901467
6425	ENSG00000169621	0,410840405	4,145770424	3,1192129	0,090581353
6426	ENSG00000169629	-0,055829572	7,388524142	0,1707905	0,683206124
6427	ENSG00000169641	-0,172538385	6,00856638	1,9104752	0,180077619
6428	ENSG00000169692	0,025371608	5,059923063	0,0096215	0,922707002
6429	ENSG00000169710	-0,909874373	6,594813515	1,8794664	0,183561346

6430	ENSG00000169714	-0,178021403	8,2369579	1,4044475	0,24798124
6431	ENSG00000169715	0,002069337	4,727840938	3,139E-05	0,995578115
6432	ENSG00000169727	0,016833813	5,15860535	0,0093922	0,923630579
6433	ENSG00000169738	-0,211461324	4,897829998	0,985006	0,331243358
6434	ENSG00000169740	-0,316384225	4,619715244	5,2059441	0,032005364
6435	ENSG00000169744	0,070386025	5,539256828	0,2480901	0,623119855
6436	ENSG00000169756	0,153485455	6,231105409	0,9626842	0,336671076
6437	ENSG00000169762	-0,121863137	5,123103251	0,8381507	0,369346956
6438	ENSG00000169764	-0,411056542	8,115657113	3,0384208	0,094605419
6439	ENSG00000169813	-0,066308957	6,042555659	0,2312992	0,63506795
6440	ENSG00000169814	0,210651369	4,368449922	1,3099704	0,264114757
6441	ENSG00000169826	0,091940772	4,843434653	0,4928284	0,489653814
6442	ENSG00000169855	0,924625877	5,006102724	12,298136	0,001885986
6443	ENSG00000169857	-0,426356361	4,076793352	6,7004428	0,016389862
6444	ENSG00000169862	0,021689808	3,811144314	0,0041737	0,949044827
6445	ENSG00000169871	0,2831364	5,128220159	1,4583838	0,239400361
6446	ENSG00000169891	-1,18642772	4,38041083	15,977615	0,000562186
6447	ENSG00000169895	0,004816164	5,418923715	0,0013957	0,970518444
6448	ENSG00000169896	-0,531827154	4,038067848	4,6842263	0,041013406
6449	ENSG00000169902	-0,209531889	3,923695441	2,1254125	0,223857699
6450	ENSG00000169905	0,272291656	6,623178672	1,7228413	0,20221292
6451	ENSG00000169908	0,978760981	7,558988761	3,1372087	0,089712203
6452	ENSG00000169914	0,037350505	3,965998051	0,0841844	0,846050918
6453	ENSG00000169919	0,099862441	4,260994393	0,4099243	0,536672784
6454	ENSG00000169925	0,371261841	3,755459099	2,2579207	0,146473422
6455	ENSG00000169926	0,083471258	6,088482151	0,1338421	0,717808409
6456	ENSG00000169946	0,299979826	5,154175455	4,6711584	0,041240755
6457	ENSG00000169967	0,210158974	6,445156952	1,2776498	0,269930234
6458	ENSG00000169976	-0,291318308	4,916499604	2,0603185	0,164574152
6459	ENSG00000169981	-0,294648264	3,673357938	10,404818	0,121317183
6460	ENSG00000170004	0,152782105	5,649753197	0,3670241	0,550534528
6461	ENSG00000170017	-0,045368575	4,431043672	0,0297064	0,864658462
6462	ENSG00000170027	-0,005463531	7,676061623	0,0013064	0,971476955
6463	ENSG00000170035	-0,245663529	4,897960015	5,6152685	0,049945862
6464	ENSG00000170088	0,059672035	4,669869422	0,3356201	0,613395322
6465	ENSG00000170089	-0,225318556	3,717262839	1,4272266	0,244333519
6466	ENSG00000170100	-0,063276698	3,804708529	0,2278405	0,680791723
6467	ENSG00000170113	0,11423963	3,964459443	0,729353	0,496466718
6468	ENSG00000170142	-0,195331209	5,411216933	0,9804416	0,332343262
6469	ENSG00000170144	0,030052993	7,194419798	0,064754	0,801376553
6470	ENSG00000170145	0,113860184	6,579659255	0,2937125	0,59303816
6471	ENSG00000170153	-0,155849483	6,816203845	0,6339583	0,434009489
6472	ENSG00000170185	-0,229958198	5,465591021	3,084829	0,092222972
6473	ENSG00000170234	0,116135477	4,398554394	0,93939	0,406838735
6474	ENSG00000170242	-0,107637505	7,06158389	0,3232078	0,57516898
6475	ENSG00000170248	0,007434098	6,94649604	0,004396	0,947705589
6476	ENSG00000170266	0,020398261	4,637396833	0,0287964	0,933854931
6477	ENSG00000170271	0,557961615	5,428344772	7,70111	0,010738939
6478	ENSG00000170275	0,462505872	6,641921094	7,627442	0,01107142
6479	ENSG00000170290	0,141115327	5,830521143	0,1226807	0,729316891
6480	ENSG00000170291	-0,101495245	4,130551721	0,6262403	0,506989477
6481	ENSG00000170310	-0,233882548	4,572190474	2,1390153	0,157059906
6482	ENSG00000170315	-0,008664758	7,37417181	0,0027185	0,958865813
6483	ENSG00000170322	0,295829167	4,055848775	2,9553144	0,098962555
6484	ENSG00000170323	-0,021455662	7,965448082	0,0012518	0,972080354
6485	ENSG00000170340	0,131462324	4,630853817	0,6055931	0,444345902
6486	ENSG00000170345	1,243312083	7,049789808	5,0137111	0,035061619
6487	ENSG00000170348	0,008318208	7,904784128	0,004053	0,949784051
6488	ENSG00000170364	-0,151425762	4,052090277	2,0873196	0,363870255

6489	ENSG00000170365	-0,000806652	3,811480748	2,979E-05	0,998813928
6490	ENSG00000170385	0,49336177	5,868810715	3,790612	0,063792729
6491	ENSG00000170390	0,079199444	3,766923695	0,2267239	0,714559629
6492	ENSG00000170417	-0,441254063	7,731153358	0,8511459	0,365759491
6493	ENSG00000170421	-0,014899154	6,300400222	0,0019436	0,965214582
6494	ENSG00000170439	-1,656665006	6,789002273	14,829814	0,000808398
6495	ENSG00000170445	-0,085986764	5,138487457	0,6202919	0,438913786
6496	ENSG00000170448	-2,032541968	5,719664372	18,295252	0,00027956
6497	ENSG00000170456	-0,029856686	6,158392712	0,0335207	0,856326574
6498	ENSG00000170458	-0,549801839	5,58431029	2,0014575	0,170479706
6499	ENSG00000170464	-0,17375639	4,316303925	1,4486973	0,240895302
6500	ENSG00000170471	0,00058156	6,199179329	2,85E-05	0,995785954
6501	ENSG00000170485	-0,521130225	4,725045428	3,6839767	0,067366501
6502	ENSG00000170500	0,294756214	6,075423788	1,2351732	0,277831038
6503	ENSG00000170502	-0,174047013	4,508569074	2,5391165	0,202189658
6504	ENSG00000170515	-0,122323021	5,22307233	1,2423686	0,27643189
6505	ENSG00000170525	-0,046668612	5,605031404	0,0098312	0,921872402
6506	ENSG00000170540	0,349024013	5,625175269	1,6242819	0,215160824
6507	ENSG00000170542	0,562899543	4,180837586	3,5421394	0,072479864
6508	ENSG00000170558	0,343121265	7,876285844	1,8580111	0,18598727
6509	ENSG00000170571	0,43725362	5,329036682	1,0016052	0,327285662
6510	ENSG00000170581	0,444938603	5,942109078	3,9287917	0,059478467
6511	ENSG00000170584	0,033674232	4,221630352	0,0524678	0,820835261
6512	ENSG00000170606	0,011729684	6,936322902	0,0051946	0,94316322
6513	ENSG00000170624	0,077640154	7,249987252	0,097478	0,757676696
6514	ENSG00000170629	-0,215908203	4,135721123	0,5266455	0,475305278
6515	ENSG00000170632	0,260502312	4,713974563	3,7714853	0,064374145
6516	ENSG00000170633	-0,113630913	4,49083892	1,4087342	0,412507271
6517	ENSG00000170634	-0,123536093	4,958909949	0,4372306	0,515000208
6518	ENSG00000170653	0,082342506	4,479006294	0,2096566	0,651312282
6519	ENSG00000170667	0,395199284	4,113730316	1,86468	0,185229037
6520	ENSG00000170677	-0,235292934	5,498039812	1,7555469	0,198131634
6521	ENSG00000170681	0,191439354	5,647506381	0,4088224	0,528854675
6522	ENSG00000170759	0,039748673	8,313224914	0,0514006	0,822630671
6523	ENSG00000170776	-0,053939044	8,806365819	0,0569807	0,813437197
6524	ENSG00000170791	-0,323792874	5,172551553	1,979012	0,172799178
6525	ENSG00000170801	-0,081608282	5,695690167	0,0728332	0,789650422
6526	ENSG00000170802	-0,349611165	5,730725284	1,6078887	0,217413865
6527	ENSG00000170807	-0,14582522	9,100986992	0,2126851	0,648981503
6528	ENSG00000170832	-0,094399385	5,91197191	0,5165127	0,479511325
6529	ENSG00000170836	0,078549294	3,825237808	0,2534527	0,663016593
6530	ENSG00000170852	0,103322063	5,71024465	0,4564376	0,505997466
6531	ENSG00000170854	-0,24764995	4,282642282	3,4386941	0,118173682
6532	ENSG00000170860	-0,38496075	5,507630006	2,3319754	0,140310926
6533	ENSG00000170871	-0,197691239	7,272155301	1,5562479	0,224707856
6534	ENSG00000170873	-0,277376327	5,442324706	2,3767395	0,136735586
6535	ENSG00000170876	0,283882805	6,198209132	3,5492814	0,07221006
6536	ENSG00000170881	-0,033276856	5,120752842	0,0425197	0,838437883
6537	ENSG00000170889	0,309979119	7,572770605	1,6323044	0,214068889
6538	ENSG00000170899	0,155931422	4,121367905	0,7367158	0,399528829
6539	ENSG00000170903	-0,23020227	5,032642868	1,8044507	0,192217269
6540	ENSG00000170906	-0,223917516	5,509974277	1,0414453	0,318049279
6541	ENSG00000170919	0,166565718	5,050870594	0,8726625	0,359876906
6542	ENSG00000170921	0,210246256	4,622970696	1,4316964	0,243617874
6543	ENSG00000170946	-0,051649334	4,594890453	0,0614389	0,806424824
6544	ENSG00000170949	0,119597673	5,422746161	0,7468163	0,396342893
6545	ENSG00000170954	-0,234184235	4,641018226	1,1904518	0,286482472
6546	ENSG00000170961	-1,041954601	4,328737785	6,1548972	0,020812082
6547	ENSG00000170962	1,076717823	5,683943856	10,81296	0,003205279

6548	ENSG00000170989	-0,230532538	5,792773579	2,364573	0,137696499
6549	ENSG00000171016	0,119239107	4,242509556	0,4685631	0,500455951
6550	ENSG00000171033	0,032099445	6,578752008	0,0103235	0,91994681
6551	ENSG00000171055	-0,235768761	6,187239504	2,5377106	0,124715683
6552	ENSG00000171100	0,066968652	4,255454312	0,3817799	0,600749641
6553	ENSG00000171105	0,081214909	6,814117948	0,1472412	0,704693012
6554	ENSG00000171109	-0,436029358	6,696844406	4,0457241	0,056085389
6555	ENSG00000171115	0,075593319	4,900219178	0,1293592	0,722362822
6556	ENSG00000171130	0,02383202	4,435824397	0,0103584	0,91981214
6557	ENSG00000171132	-0,264695475	4,509962055	2,6742754	0,115529122
6558	ENSG00000171135	-0,184740067	4,007180047	1,4964832	0,270295174
6559	ENSG00000171148	0,07562656	5,263568701	0,1618403	0,691164161
6560	ENSG00000171150	0,150794513	5,639793786	1,5554775	0,224775831
6561	ENSG00000171155	-0,345441276	4,661370168	2,7813565	0,108861234
6562	ENSG00000171161	-0,213941006	4,514054945	1,8178348	0,190636713
6563	ENSG00000171202	-0,703323252	5,069907041	7,7682759	0,01044539
6564	ENSG00000171204	-0,49619585	5,23398815	4,5456554	0,043849474
6565	ENSG00000171206	-0,08998391	5,418012411	0,1443717	0,707442183
6566	ENSG00000171223	0,382559976	6,254745544	1,2745476	0,27049719
6567	ENSG00000171262	-0,200969845	5,070374544	1,9958305	0,171032009
6568	ENSG00000171291	-0,138069685	3,623778476	1,0642196	0,484626922
6569	ENSG00000171295	0,043137982	3,879658638	0,1246934	0,806983695
6570	ENSG00000171302	-0,010264093	4,195708791	0,0044319	0,947494425
6571	ENSG00000171303	-0,629250974	4,839071349	7,545997	0,011452291
6572	ENSG00000171307	-0,266275484	4,178900918	4,1392891	0,091677068
6573	ENSG00000171310	0,029479759	4,179413782	0,0540321	0,833723205
6574	ENSG00000171311	0,146917391	3,822543719	1,2435863	0,39936911
6575	ENSG00000171314	-0,010025898	7,510848377	0,0069487	0,934281495
6576	ENSG00000171316	-0,228586068	5,40089214	1,5460097	0,226190392
6577	ENSG00000171365	0,083456596	3,578137793	0,2564664	0,670325796
6578	ENSG00000171385	-0,343303349	5,041220553	2,8669971	0,103850656
6579	ENSG00000171408	0,266252106	4,503635262	1,4869505	0,234988593
6580	ENSG00000171444	0,248502015	6,14482122	1,7719861	0,196118734
6581	ENSG00000171448	-0,14071701	3,514972241	1,2354335	0,497308576
6582	ENSG00000171451	0,27777168	4,378673315	0,7503749	0,395261092
6583	ENSG00000171456	0,015284986	5,899341446	0,0066177	0,935864569
6584	ENSG00000171466	0,010410867	5,4366301	0,011704	0,914779197
6585	ENSG00000171467	0,136167897	4,650174666	0,5228892	0,476866434
6586	ENSG00000171469	0,100127892	4,663070974	0,7344675	0,400207467
6587	ENSG00000171475	0,259120069	4,461225636	2,0308758	0,167496615
6588	ENSG00000171488	-0,113506013	5,020061676	0,6425124	0,430954972
6589	ENSG00000171490	0,075836799	6,529811779	0,5203512	0,477901016
6590	ENSG00000171492	0,213107853	3,773351564	2,3539893	0,234818419
6591	ENSG00000171497	-0,185834065	4,981258442	1,6063968	0,217612102
6592	ENSG00000171503	-0,377125206	6,88554089	3,174399	0,087946364
6593	ENSG00000171517	-0,499149266	4,685416228	4,5812886	0,043099658
6594	ENSG00000171530	-0,169335991	5,442422226	0,8433665	0,367920464
6595	ENSG00000171552	-0,030937168	4,288233346	0,0235536	0,879358327
6596	ENSG00000171566	-0,151885734	5,517036706	0,555405	0,463629365
6597	ENSG00000171603	0,202162691	6,039752344	0,8326652	0,3709234
6598	ENSG00000171604	-0,155547453	4,516554827	1,5420892	0,25327818
6599	ENSG00000171606	0,078293711	4,795069298	0,3903996	0,618977296
6600	ENSG00000171612	-0,173419133	4,046391424	0,5787002	0,454513639
6601	ENSG00000171617	0,08409016	4,733350303	0,070186	0,793414546
6602	ENSG00000171621	-0,81349845	4,056692353	11,88119	0,002182905
6603	ENSG00000171634	0,119533535	6,946650443	0,8262609	0,372705072
6604	ENSG00000171659	0,399840204	4,228985519	0,8901139	0,355204814
6605	ENSG00000171681	0,139849849	6,999136193	1,6618253	0,210065993
6606	ENSG00000171714	-0,046804925	7,328849987	0,0226479	0,881682018

6607	ENSG00000171720	0,120495174	4,53509462	0,9009012	0,493734181
6608	ENSG00000171723	-0,086770307	4,579841114	0,6278972	0,516972626
6609	ENSG00000171724	0,865287505	4,733223006	11,157508	0,002827373
6610	ENSG00000171735	-0,255068964	5,201500745	2,4928979	0,127950607
6611	ENSG00000171766	0,730024384	5,378816689	8,9012187	0,006620368
6612	ENSG00000171791	-0,07930499	5,308427421	0,1532629	0,699022553
6613	ENSG00000171793	-0,188728609	3,980108537	0,654493	0,426760799
6614	ENSG00000171817	0,022306036	3,980011592	0,0222846	0,887918231
6615	ENSG00000171824	0,000860139	4,899521884	5,799E-05	0,993989126
6616	ENSG00000171827	0,13110768	4,179791602	1,2312944	0,375154417
6617	ENSG00000171843	-0,122016118	4,138210479	1,0557588	0,461974009
6618	ENSG00000171853	-0,198675424	5,039365002	1,7145191	0,203268023
6619	ENSG00000171858	0,299125319	6,398787851	2,9293727	0,100370046
6620	ENSG00000171860	-0,334853086	3,770282568	1,6782834	0,207942659
6621	ENSG00000171862	-0,030461226	7,660270408	0,0213858	0,885000784
6622	ENSG00000171863	0,00626833	7,869289098	0,0008344	0,977203577
6623	ENSG00000171865	0,132531376	4,520010303	1,3324071	0,36553483
6624	ENSG00000171867	0,19538325	7,636827324	1,4862422	0,235067437
6625	ENSG00000171877	-0,178597672	4,770566708	0,4055055	0,530516655
6626	ENSG00000171914	0,116504131	6,191602693	0,1865321	0,669821102
6627	ENSG00000171928	-0,079979221	5,26026177	0,1356266	0,716019558
6628	ENSG00000171940	0,182621489	4,679030369	0,5692122	0,458190826
6629	ENSG00000171943	0,332970452	5,641959394	1,7751625	0,195732719
6630	ENSG00000171988	-0,061248535	7,355656541	0,1080796	0,745301396
6631	ENSG00000171992	0,042126202	8,903790046	0,0347366	0,853774944
6632	ENSG00000172007	0,154493582	4,242529939	1,3012632	0,265624856
6633	ENSG00000172014	-0,284114065	4,198206913	0,9330579	0,344068773
6634	ENSG00000172037	0,394853985	8,52649434	2,4529876	0,130891448
6635	ENSG00000172046	0,25968408	5,276024693	1,6988174	0,205277423
6636	ENSG00000172053	-0,141216482	6,392952923	1,232414	0,27831528
6637	ENSG00000172057	0,512566222	5,276117973	7,7978997	0,010318743
6638	ENSG00000172058	0,273763308	4,056358448	3,1578009	0,117352361
6639	ENSG00000172059	0,40198883	4,797488722	5,0882927	0,033853021
6640	ENSG00000172062	0,281246467	4,895311966	2,8261411	0,106206681
6641	ENSG00000172071	0,291687362	4,606979191	1,2541332	0,274267369
6642	ENSG00000172086	-0,184650215	5,018268674	0,8617282	0,3628492
6643	ENSG00000172113	-0,182594698	3,994334227	3,2770636	0,274142285
6644	ENSG00000172115	-0,745128083	9,026049829	4,3977992	0,047121847
6645	ENSG00000172159	-0,225764459	6,023500724	0,6552585	0,42649427
6646	ENSG00000172164	-0,20475907	3,857419018	0,6689519	0,421769432
6647	ENSG00000172172	-0,523058569	4,93344687	5,7702211	0,02472627
6648	ENSG00000172175	-0,075383721	5,350372981	0,2391496	0,629429518
6649	ENSG00000172197	0,071382652	3,729772494	0,1831944	0,728231621
6650	ENSG00000172201	2,945856109	6,155479361	31,425504	1,03339E-05
6651	ENSG00000172209	-0,044710801	5,582594764	0,0123311	0,912538958
6652	ENSG00000172216	0,297098298	4,585671227	1,4966857	0,2335088
6653	ENSG00000172239	-0,118702617	6,134373042	0,9516583	0,339363016
6654	ENSG00000172260	-0,257205727	5,347587123	1,0053249	0,326407762
6655	ENSG00000172262	0,021863868	4,646896178	0,0385757	0,84600233
6656	ENSG00000172270	-0,039767271	8,703453421	0,0315496	0,860567229
6657	ENSG00000172292	-0,267000228	6,364105715	1,3208801	0,262188711
6658	ENSG00000172296	0,050969369	4,021128515	0,0823594	0,776682221
6659	ENSG00000172301	0,111969961	4,784569692	0,6927573	0,413716248
6660	ENSG00000172315	-0,127237346	3,697254678	0,9305244	0,508545374
6661	ENSG00000172331	-0,473628011	4,271943761	3,6633815	0,068082745
6662	ENSG00000172340	-0,115248089	6,612171829	0,3771179	0,545147514
6663	ENSG00000172346	-0,403724451	6,44637639	2,4065706	0,134413008
6664	ENSG00000172348	-0,50408413	7,586379686	7,7844564	0,010376005
6665	ENSG00000172349	0,514264152	3,917975791	3,5446199	0,072386709

6666	ENSG00000172354	-0,03196208	5,514591455	0,0236512	0,879110634
6667	ENSG00000172380	0,477805912	6,52517169	3,2145485	0,086084887
6668	ENSG00000172399	-0,748437589	9,972446762	3,8054824	0,063311743
6669	ENSG00000172403	-0,317943316	7,846227543	4,4107138	0,046785539
6670	ENSG00000172428	-0,206998199	4,778848315	1,6395764	0,213085091
6671	ENSG00000172432	0,013411876	4,514615372	0,0064934	0,93646844
6672	ENSG00000172456	0,188268169	4,179402731	0,9045861	0,351395601
6673	ENSG00000172465	-0,318898887	4,153475964	2,8597994	0,104261268
6674	ENSG00000172466	-0,088340717	6,952812134	0,3799542	0,543635434
6675	ENSG00000172469	-0,147248857	4,389893977	0,4176318	0,52448662
6676	ENSG00000172493	-0,129553865	7,176321849	0,8529227	0,365235485
6677	ENSG00000172500	0,022503887	4,449606818	0,0223198	0,941580812
6678	ENSG00000172531	-0,017904476	5,175952299	0,0132866	0,909228673
6679	ENSG00000172534	0,085817163	4,746605633	0,0992334	0,755577981
6680	ENSG00000172572	-0,292293356	8,210185508	0,9654735	0,335986077
6681	ENSG00000172575	-0,045553734	3,774225692	0,0465955	0,830990583
6682	ENSG00000172586	-0,461550838	4,158311824	9,7276621	0,006037114
6683	ENSG00000172590	-0,1800941	4,333030929	1,0550518	0,314976804
6684	ENSG00000172638	0,253229391	5,499198424	2,6467946	0,117316767
6685	ENSG00000172661	-0,309234966	6,941021534	2,0089785	0,169710963
6686	ENSG00000172667	0,365602301	5,724598968	2,6215448	0,118987972
6687	ENSG00000172671	-0,19906789	4,072913165	2,3655774	0,220266598
6688	ENSG00000172716	0,105259923	5,656191301	0,3066451	0,585064179
6689	ENSG00000172728	0,480230325	3,736543264	4,887778	0,037213787
6690	ENSG00000172731	-0,508918497	4,912459244	3,6223053	0,069537391
6691	ENSG00000172757	-0,083793294	6,847179871	0,2551155	0,618278359
6692	ENSG00000172765	0,077504681	5,422516918	0,4511961	0,508403338
6693	ENSG00000172766	-0,023195473	4,453729482	0,0268639	0,888515084
6694	ENSG00000172775	-0,186795053	5,510667426	3,8588597	0,071433562
6695	ENSG00000172785	-0,347968078	6,110950285	4,4977215	0,04488142
6696	ENSG00000172795	0,160539269	5,929169952	0,9189296	0,347677218
6697	ENSG00000172809	0,120828652	7,428550292	0,578185	0,4546963
6698	ENSG00000172817	-0,228313263	4,80475125	2,5622099	0,123007428
6699	ENSG00000172831	0,353385496	5,07755573	3,5149512	0,07350996
6700	ENSG00000172845	0,059023277	6,614181295	0,1180876	0,734224394
6701	ENSG00000172869	-0,058833135	6,882346838	0,1995404	0,659235185
6702	ENSG00000172888	0,110795868	4,68511949	0,6558875	0,426249502
6703	ENSG00000172890	0,117917308	4,587001103	0,5459593	0,467411305
6704	ENSG00000172893	-0,05327982	4,342127803	0,0566734	0,813931056
6705	ENSG00000172915	-0,117779723	6,810204237	0,3079029	0,584300525
6706	ENSG00000172939	0,091549564	5,567829631	0,7878497	0,383863291
6707	ENSG00000172943	0,236209083	4,323681984	1,4278113	0,244239748
6708	ENSG00000172954	-0,163237598	4,867383825	0,7164079	0,406003212
6709	ENSG00000172965	0,404794281	4,618198028	5,6138281	0,026546724
6710	ENSG00000172974	-0,402023727	6,256454868	7,0584486	0,014056096
6711	ENSG00000172986	0,511761437	5,187649942	5,8599293	0,023745101
6712	ENSG00000172992	0,266340993	4,007593055	3,4606383	0,142092531
6713	ENSG00000173011	-0,108024397	4,529870885	0,3650224	0,551614983
6714	ENSG00000173020	0,043338079	5,103984719	0,0722037	0,790538778
6715	ENSG00000173039	-0,025864798	4,744578026	0,0219797	0,883427008
6716	ENSG00000173041	-0,141415433	5,887271716	0,6816212	0,417469716
6717	ENSG00000173064	0,092596217	6,000800637	0,1839368	0,671982565
6718	ENSG00000173068	0,160504172	5,844979727	0,1742057	0,680252448
6719	ENSG00000173085	-0,332056122	4,078734728	3,3142499	0,08165551
6720	ENSG00000173113	0,289415404	4,878432042	2,6676171	0,115959297
6721	ENSG00000173114	-1,831260658	5,442526076	18,744552	0,000245368
6722	ENSG00000173120	0,123307398	5,892337022	0,4436128	0,511977366
6723	ENSG00000173141	-0,214785291	4,657001648	2,5636226	0,122881417
6724	ENSG00000173145	-0,052876223	4,900784633	0,0670397	0,797988796

6725	ENSG00000173153	-0,241039163	5,614098218	1,2289251	0,279018793
6726	ENSG00000173163	-0,22671264	5,162098775	1,6798155	0,207742325
6727	ENSG00000173166	0,00247752	4,232597139	0,0001231	0,991242906
6728	ENSG00000173175	0,138237741	6,992042518	0,23767	0,630486264
6729	ENSG00000173193	0,019672958	6,595734084	0,0083915	0,927801065
6730	ENSG00000173207	-0,055951008	3,666181905	0,0772565	0,795158988
6731	ENSG00000173208	-0,174459439	5,024439673	0,385992	0,540493417
6732	ENSG00000173209	0,166729428	5,348676947	1,166863	0,291190179
6733	ENSG00000173210	-0,114619664	6,988036895	0,2829144	0,599873217
6734	ENSG00000173214	-0,001823875	5,092354371	6,396E-05	0,993687839
6735	ENSG00000173218	0,187456435	3,801749557	1,8608482	0,325684607
6736	ENSG00000173221	-0,356445641	5,642520106	4,1994389	0,051953876
6737	ENSG00000173226	0,201504796	4,095550013	1,9474039	0,18803006
6738	ENSG00000173230	0,068898671	8,330276187	0,3024487	0,587608754
6739	ENSG00000173258	0,453263588	6,189515176	6,0320886	0,021981219
6740	ENSG00000173269	-0,107727735	5,709294353	0,5011735	0,486063399
6741	ENSG00000173273	0,113787948	6,070517315	0,932883	0,344078535
6742	ENSG00000173275	-0,446409516	4,210068681	4,3699603	0,047768327
6743	ENSG00000173276	0,0816751	4,633522875	0,1262569	0,725567233
6744	ENSG00000173281	-0,247148234	5,949642794	3,4880361	0,074501524
6745	ENSG00000173320	-0,466315171	4,947171458	14,602273	0,000865864
6746	ENSG00000173327	0,073018198	4,47860882	0,1992909	0,764990088
6747	ENSG00000173334	0,443422602	5,400477699	1,4824046	0,235683682
6748	ENSG00000173369	-0,270913902	5,992979027	0,6153953	0,440730211
6749	ENSG00000173372	-0,10360787	5,701411801	0,1120656	0,740823673
6750	ENSG00000173402	-0,064704109	6,420597354	0,0861035	0,771809047
6751	ENSG00000173409	-0,369953048	4,486909517	3,4865704	0,074603172
6752	ENSG00000173418	-0,067974877	5,093028603	0,2866333	0,72131403
6753	ENSG00000173436	-0,579240938	4,486684363	18,975597	0,000228035
6754	ENSG00000173442	0,223205754	5,957389613	1,6370739	0,213423002
6755	ENSG00000173451	-0,229463377	3,817000685	0,9396732	0,342397279
6756	ENSG00000173457	-0,395130236	4,799602128	2,4936165	0,127898375
6757	ENSG00000173473	0,301409197	5,568033195	5,1403017	0,033002617
6758	ENSG00000173480	0,17321542	4,814669995	2,2243581	0,161053961
6759	ENSG00000173482	0,011396015	7,107173308	0,0040667	0,949701008
6760	ENSG00000173511	-0,046756577	6,291761055	0,0559225	0,815143867
6761	ENSG00000173517	0,251805566	7,071373051	2,4385265	0,131976734
6762	ENSG00000173542	0,044942053	5,5909181	0,0796588	0,780272256
6763	ENSG00000173545	-0,126561028	4,619993838	0,4534074	0,507399188
6764	ENSG00000173546	0,131214437	4,475573742	0,1382029	0,71346057
6765	ENSG00000173548	0,550104319	4,72051558	5,6533107	0,026073434
6766	ENSG00000173559	-0,303432157	5,943591604	0,2842588	0,599013107
6767	ENSG00000173575	0,141756539	6,753726427	1,0922377	0,306746302
6768	ENSG00000173588	-0,446809299	5,235316304	3,9366204	0,059244153
6769	ENSG00000173598	-0,211051265	7,930920025	1,6847796	0,207050607
6770	ENSG00000173611	0,264237445	4,468651884	3,874734	0,061082638
6771	ENSG00000173614	-0,124091063	4,37915014	0,6820833	0,417311457
6772	ENSG00000173641	-0,223332873	9,769560722	0,7383851	0,399003584
6773	ENSG00000173660	-0,691902389	8,051182899	7,5133924	0,011608801
6774	ENSG00000173674	0,008120329	6,444638634	0,0022353	0,962697122
6775	ENSG00000173681	-0,07790314	4,606216093	0,4159298	0,600033833
6776	ENSG00000173692	-0,236671409	6,785238375	2,3086003	0,142222104
6777	ENSG00000173706	0,188325958	7,959436654	0,7313066	0,401238071
6778	ENSG00000173726	0,123991217	6,942892878	1,6347737	0,213690343
6779	ENSG00000173744	-0,277433636	6,482190194	3,0591685	0,093552612
6780	ENSG00000173757	0,28897727	5,862720034	2,7313415	0,11191795
6781	ENSG00000173786	0,078663961	4,759992513	0,1742579	0,68020736
6782	ENSG00000173801	0,176495173	6,4384313	0,6975379	0,412162675
6783	ENSG00000173812	-0,016583125	8,357022872	0,0138609	0,907293463

6784	ENSG00000173821	0,176137813	7,108084436	0,4862361	0,492566276
6785	ENSG00000173848	0,123768731	5,355025801	0,2263841	0,638684401
6786	ENSG00000173852	0,182791769	5,433725886	1,0999611	0,305118346
6787	ENSG00000173875	0,098873723	5,173622819	0,9017788	0,352095967
6788	ENSG00000173889	0,126740196	6,932940886	0,9146739	0,348740395
6789	ENSG00000173905	0,150948404	6,418735126	1,3332988	0,259977668
6790	ENSG00000173914	-0,100980102	4,256025302	0,7909266	0,477250589
6791	ENSG00000173915	-0,690767995	7,271615705	5,204574	0,03206065
6792	ENSG00000173918	-0,405161891	6,922383359	1,334716	0,259772252
6793	ENSG00000173926	0,060193231	3,917134598	0,1321888	0,767135529
6794	ENSG00000173933	-0,346786705	4,290813104	3,760612	0,064775815
6795	ENSG00000173960	0,101139685	5,168833133	1,0344872	0,349592641
6796	ENSG00000173991	-0,662800884	10,41707015	3,1244958	0,090325199
6797	ENSG00000174010	0,128901045	4,616822295	0,500998	0,486138935
6798	ENSG00000174013	-0,214529408	4,01197262	3,1265815	0,205397884
6799	ENSG00000174032	-0,045627361	5,870385403	0,0346933	0,853864885
6800	ENSG00000174059	-0,304927055	6,777027707	1,6351636	0,213681413
6801	ENSG00000174080	0,319389763	5,959266435	3,5517235	0,07212069
6802	ENSG00000174099	0,141863072	7,308332483	1,7632538	0,19713999
6803	ENSG00000174106	0,067112409	4,947904011	0,2545839	0,618625833
6804	ENSG00000174125	0,251131914	3,942100884	0,8417365	0,368375581
6805	ENSG00000174132	-0,008786227	3,69514507	0,0022157	0,994884975
6806	ENSG00000174173	-0,459993064	4,663752584	3,830007	0,062527482
6807	ENSG00000174175	0,86044581	4,364243609	7,595581	0,011218724
6808	ENSG00000174197	0,069138867	7,042364631	0,439604	0,513849638
6809	ENSG00000174227	-0,086555853	4,566710574	0,2877581	0,596786667
6810	ENSG00000174231	0,136508271	7,525548863	0,437609	0,514820073
6811	ENSG00000174233	0,846018995	6,632725519	7,8041375	0,010292292
6812	ENSG00000174238	0,019171538	5,269630389	0,0162094	0,899791526
6813	ENSG00000174243	0,128523204	5,396684044	0,7748408	0,387772278
6814	ENSG00000174282	0,267599202	6,343192527	2,0585487	0,164748068
6815	ENSG00000174306	0,061619182	5,617886453	0,0716373	0,79134162
6816	ENSG00000174348	-0,24958928	5,460149476	1,0135399	0,324480424
6817	ENSG00000174353	-0,012702824	4,444911601	0,0031301	0,955864754
6818	ENSG00000174373	-0,003953586	6,930154476	0,0007632	0,978196164
6819	ENSG00000174405	-0,201986699	4,5675924	1,189757	0,28661968
6820	ENSG00000174428	0,007112739	5,590820123	0,0022786	0,962337583
6821	ENSG00000174429	-0,574696847	5,042199527	2,5374026	0,124762798
6822	ENSG00000174437	-0,867945334	11,3319032	8,3322325	0,008302203
6823	ENSG00000174444	0,153074807	9,616138546	0,4705466	0,499542026
6824	ENSG00000174446	-0,322515727	4,38689944	3,2681718	0,083669135
6825	ENSG00000174485	0,066888516	6,149975138	0,1727483	0,68151429
6826	ENSG00000174501	0,040994997	6,635690794	0,0698059	0,793961264
6827	ENSG00000174502	0,199744127	4,337878959	0,5340053	0,472271177
6828	ENSG00000174547	-0,29114379	4,441480095	2,9457982	0,099476188
6829	ENSG00000174574	-0,201434444	5,532878923	2,0515666	0,165400863
6830	ENSG00000174579	0,137575293	4,805408707	0,94537	0,340947672
6831	ENSG00000174600	0,262060224	4,237761046	1,6787154	0,207886147
6832	ENSG00000174606	0,003799559	5,011092312	0,0006062	0,980568885
6833	ENSG00000174640	-0,542966158	4,480640031	2,7487973	0,110839858
6834	ENSG00000174652	0,152272316	4,699251368	0,8342958	0,37046352
6835	ENSG00000174684	0,140420809	4,95781138	0,6376051	0,432708136
6836	ENSG00000174695	-0,070552834	6,429533771	0,1399584	0,711732531
6837	ENSG00000174705	-0,824976166	4,400146001	6,216946	0,020247794
6838	ENSG00000174718	0,108294125	6,388240769	0,2405085	0,628462589
6839	ENSG00000174720	-0,307827288	5,863592914	2,4038539	0,134622574
6840	ENSG00000174738	0,343588527	6,651701881	2,5664753	0,122730931
6841	ENSG00000174748	0,368183647	8,617583167	3,2775033	0,083256772
6842	ENSG00000174749	0,033552841	4,640674844	0,0334034	0,856575436

6843	ENSG00000174780	0,095416242	5,799959984	0,39436	0,536172655
6844	ENSG00000174796	-0,069434979	4,563887941	0,1660432	0,687402138
6845	ENSG00000174804	0,395433033	6,602712041	2,0216716	0,168423096
6846	ENSG00000174839	0,045128372	5,017249507	0,0920294	0,764326709
6847	ENSG00000174840	0,015054138	4,73923815	0,0116907	0,914828142
6848	ENSG00000174842	-0,177618167	3,756478038	0,8021009	0,37969899
6849	ENSG00000174891	-0,714404109	5,052949209	17,920172	0,000312097
6850	ENSG00000174903	-0,003882115	4,646355414	0,0004169	0,983884486
6851	ENSG00000174917	-0,271255893	5,160567152	1,3792718	0,252184099
6852	ENSG00000174953	0,005189866	6,161144783	0,0023121	0,962060601
6853	ENSG00000174977	-0,140888312	3,635642264	0,8094784	0,512854661
6854	ENSG00000175029	-0,169265307	4,875059136	1,7990667	0,192812582
6855	ENSG00000175048	0,382510829	3,739873854	3,2108019	0,086256657
6856	ENSG00000175054	-0,167648293	6,005612428	1,5342715	0,22786244
6857	ENSG00000175061	0,558020392	6,230749575	8,071077	0,009227507
6858	ENSG00000175066	0,151105766	4,873841978	0,6776134	0,418822536
6859	ENSG00000175073	0,004519056	5,97311433	0,0013343	0,971174282
6860	ENSG00000175084	-0,17142428	11,7091031	0,3606328	0,553982037
6861	ENSG00000175087	-0,116881692	3,687543744	0,4011091	0,594838369
6862	ENSG00000175104	0,029391294	5,073542442	0,0602449	0,825229295
6863	ENSG00000175105	-0,035500883	5,150978634	0,032696	0,858084807
6864	ENSG00000175106	-0,219498661	4,045542069	1,5170274	0,23326026
6865	ENSG00000175110	-0,563155192	5,2015385	6,0649914	0,02166098
6866	ENSG00000175115	0,420250389	5,659630206	3,0692936	0,093043761
6867	ENSG00000175137	0,118876184	4,153601142	0,4853444	0,531627845
6868	ENSG00000175155	0,260038749	5,908217871	3,6209966	0,069540194
6869	ENSG00000175161	-0,724800565	4,591214068	4,5974771	0,042763787
6870	ENSG00000175166	-0,09868813	7,001394704	0,5412427	0,469293258
6871	ENSG00000175183	-0,57948109	3,932050697	5,2650238	0,031171013
6872	ENSG00000175197	-0,149518296	3,896290126	0,673755	0,449257487
6873	ENSG00000175198	-0,144752404	5,490461739	1,4423519	0,241880613
6874	ENSG00000175203	-0,064700246	6,231926744	0,2806743	0,601294718
6875	ENSG00000175206	0,687269266	13,21677929	1,9126686	0,179883115
6876	ENSG00000175215	0,192396899	6,654477613	0,9285678	0,345209819
6877	ENSG00000175216	-0,078414058	6,76700327	0,3534772	0,55790704
6878	ENSG00000175220	0,63873985	6,4875845	6,5102508	0,017800575
6879	ENSG00000175224	0,225422395	5,611009844	2,302992	0,142685246
6880	ENSG00000175265	0,056325233	6,023483045	0,0684186	0,795970218
6881	ENSG00000175274	-0,029748382	5,317212998	0,0116727	0,914896149
6882	ENSG00000175309	-0,19303588	5,028060072	1,0467956	0,31683627
6883	ENSG00000175322	0,035402839	3,677517311	0,0360196	0,86966666
6884	ENSG00000175324	-0,302474305	3,812739232	2,2736871	0,145134777
6885	ENSG00000175334	-0,243883817	5,292436451	1,7793217	0,195228693
6886	ENSG00000175348	-0,175115479	5,251476527	1,7115752	0,20359846
6887	ENSG00000175354	-0,159779354	4,724323628	0,8991071	0,352830868
6888	ENSG00000175387	-0,066081803	7,225065907	0,2981259	0,590273467
6889	ENSG00000175390	0,218241899	6,452323066	3,632264	0,069137419
6890	ENSG00000175395	-0,354313639	5,495995588	3,3017298	0,082197068
6891	ENSG00000175414	0,053998355	4,313419317	0,0659321	0,799626333
6892	ENSG00000175416	-0,241923129	6,08699566	1,2955084	0,266696484
6893	ENSG00000175445	0,312251166	8,82974873	0,7194803	0,405013585
6894	ENSG00000175455	0,086377679	5,447713185	0,3179707	0,578260777
6895	ENSG00000175467	0,031027524	4,444995045	0,0245359	0,876889245
6896	ENSG00000175470	-0,088079231	4,880277611	0,6445852	0,430206066
6897	ENSG00000175471	-0,649993235	5,014412424	5,1778697	0,032462677
6898	ENSG00000175548	0,069663779	4,656109264	0,1954801	0,662503673
6899	ENSG00000175575	-0,057829766	4,436788799	0,15341	0,698879302
6900	ENSG00000175581	-0,45018068	4,074428668	6,7133065	0,016299022
6901	ENSG00000175582	-0,001311005	6,754079944	0,0001491	0,990362682

6902	ENSG00000175595	-0,005664067	3,895692156	0,0016591	0,995508701
6903	ENSG00000175606	-0,5318896	4,422891549	7,1313212	0,013630161
6904	ENSG00000175662	0,302822242	6,976400439	1,4197569	0,245535508
6905	ENSG00000175701	-0,478295489	4,101720062	3,9860348	0,057789144
6906	ENSG00000175727	0,150983197	5,547559976	0,3564748	0,556274793
6907	ENSG00000175756	-0,353030777	5,324310319	2,1675877	0,154434436
6908	ENSG00000175782	0,157409718	3,663488806	0,6836542	0,435451719
6909	ENSG00000175792	-0,337723627	4,630014777	6,0296706	0,021975224
6910	ENSG00000175806	-0,459707701	4,022457564	9,7707046	0,006589615
6911	ENSG00000175826	-0,177714745	4,488575631	0,8148315	0,376007365
6912	ENSG00000175886	0,362087785	4,672596528	4,7115592	0,040478707
6913	ENSG00000175893	-0,310166374	6,226544203	1,9151861	0,179607912
6914	ENSG00000175895	-0,1661772	4,211199991	1,0777374	0,31020044
6915	ENSG00000175899	-0,274113794	9,98433847	2,5044656	0,127065713
6916	ENSG00000175931	0,115330469	4,583772749	0,2745661	0,605274017
6917	ENSG00000175946	-0,200586083	4,278551365	0,266193	0,610797793
6918	ENSG00000175970	0,207247773	4,460155396	1,7314989	0,201122483
6919	ENSG00000175984	0,035992499	3,742092401	0,0480933	0,835414997
6920	ENSG00000176014	-0,506997773	6,690851128	9,8834076	0,004531477
6921	ENSG00000176018	-0,101479508	5,560918186	0,1703277	0,683624112
6922	ENSG00000176046	0,059825422	5,173963988	0,0712613	0,79187653
6923	ENSG00000176087	-0,139503649	5,142783913	0,6532737	0,427185906
6924	ENSG00000176095	0,230406074	4,455655519	1,5851661	0,220586032
6925	ENSG00000176102	0,032254304	4,050063294	0,0795334	0,788881971
6926	ENSG00000176105	-0,112602292	5,557213389	0,536732	0,471155311
6927	ENSG00000176124	-0,220363318	3,52314764	1,7475988	0,263601611
6928	ENSG00000176142	-0,051073736	5,080411221	0,2348275	0,649091243
6929	ENSG00000176148	0,067197311	3,748904055	0,1843994	0,748150973
6930	ENSG00000176171	-0,201021964	6,492646862	1,1257156	0,299652693
6931	ENSG00000176209	-0,111237316	4,810742388	0,4219073	0,522390314
6932	ENSG00000176225	-0,060921287	5,12615133	0,25881	0,615744218
6933	ENSG00000176261	-0,333182299	4,196566136	3,6312027	0,069219315
6934	ENSG00000176340	-0,540923158	6,698301771	6,1562745	0,020799369
6935	ENSG00000176386	-0,201278152	4,099610763	1,0319166	0,320225442
6936	ENSG00000176390	-0,061419729	3,982901847	0,1439677	0,707831775
6937	ENSG00000176393	-0,183966558	3,900113195	0,801548	0,37986051
6938	ENSG00000176407	-0,207836401	5,668230868	5,6794926	0,029024189
6939	ENSG00000176422	-0,129881675	3,88168831	0,99097	0,433936539
6940	ENSG00000176435	0,217535737	4,956833348	3,2113934	0,086200297
6941	ENSG00000176438	0,044522124	5,948279631	0,0146798	0,904611157
6942	ENSG00000176444	0,038092048	4,212319608	0,0657875	0,860846654
6943	ENSG00000176463	-0,350362037	5,448343646	4,7106883	0,040495623
6944	ENSG00000176485	-0,258864348	6,116418692	1,2555291	0,274007372
6945	ENSG00000176490	-0,372427647	6,162655553	2,467115	0,129841406
6946	ENSG00000176533	0,188447778	4,640999784	0,5950023	0,448306042
6947	ENSG00000176542	-0,021967083	5,754956403	0,0245485	0,876854409
6948	ENSG00000176623	-0,209458333	6,14069857	2,1033844	0,160382098
6949	ENSG00000176624	0,159890433	4,820434732	1,1627573	0,292020074
6950	ENSG00000176641	0,486175007	5,4093785	3,7989182	0,063523552
6951	ENSG00000176658	0,867348667	5,365197566	12,935671	0,001513949
6952	ENSG00000176681	-0,365496179	4,954380893	2,3517609	0,138717096
6953	ENSG00000176728	0,252062932	4,373060551	0,2938465	0,592954406
6954	ENSG00000176731	-0,414324976	3,939652121	4,5859408	0,043002835
6955	ENSG00000176771	0,149222492	4,111154097	0,4287483	0,519067589
6956	ENSG00000176783	-0,058771093	4,813797006	0,173367	0,680968649
6957	ENSG00000176809	-0,420271769	4,295659127	3,0049916	0,096330748
6958	ENSG00000176826	0,125381762	4,280011498	0,7348027	0,477954973
6959	ENSG00000176834	-0,124314476	3,99109588	0,4389347	0,514189961
6960	ENSG00000176853	-0,026436587	5,297346443	0,019156	0,891118856

6961	ENSG00000176871	-0,14088079	5,890209348	1,2305751	0,278665053
6962	ENSG00000176903	0,23818757	4,601047461	2,4860704	0,128436437
6963	ENSG00000176907	-0,147144369	5,122718717	0,4366283	0,515287108
6964	ENSG00000176915	0,121650295	5,410504775	0,5619186	0,461050643
6965	ENSG00000176946	-0,281343847	5,029416439	1,7541184	0,198307753
6966	ENSG00000176953	0,106784062	4,259958426	0,4106927	0,572925818
6967	ENSG00000176971	0,481547252	4,243495735	6,176164	0,020616732
6968	ENSG00000176978	-0,13566927	5,693405342	0,279183	0,602274369
6969	ENSG00000176986	0,137780022	4,697130881	0,4193634	0,523635782
6970	ENSG00000176994	0,137005652	4,604875268	0,3689601	0,549493468
6971	ENSG00000177000	-0,462995303	5,877506818	3,4498675	0,076044682
6972	ENSG00000177034	0,032225405	5,277572794	0,0636013	0,803113258
6973	ENSG00000177054	-0,332368451	4,539819919	4,5774603	0,043179518
6974	ENSG00000177058	0,080627165	4,077200237	0,3559032	0,583326316
6975	ENSG00000177084	0,188551978	4,086358355	0,5636847	0,46035548
6976	ENSG00000177098	0,24567501	4,11903939	1,1688911	0,290781389
6977	ENSG00000177119	0,200558806	7,027583628	1,0012496	0,327369766
6978	ENSG00000177125	0,246337944	4,195685099	3,0508239	0,101889923
6979	ENSG00000177144	-0,072567809	5,449538479	0,1402238	0,711472317
6980	ENSG00000177150	-0,348431365	5,394034822	3,3009347	0,082231601
6981	ENSG00000177156	-0,166248282	5,618104882	0,8289312	0,371979566
6982	ENSG00000177181	-0,514362989	4,313572235	3,0909148	0,091967847
6983	ENSG00000177189	0,124485247	6,438643632	0,3182943	0,578068709
6984	ENSG00000177200	0,002890651	7,572060914	0,0003464	0,985310429
6985	ENSG00000177225	0,210858688	4,293221194	1,4371433	0,242749395
6986	ENSG00000177239	0,38294038	4,886470495	4,7101475	0,040506131
6987	ENSG00000177302	0,177288708	3,964724908	1,1670393	0,356641864
6988	ENSG00000177311	0,023577913	6,905046576	0,0567101	0,813864692
6989	ENSG00000177354	0,625555552	5,692873597	2,5602885	0,123160039
6990	ENSG00000177370	-0,150155505	4,179447641	1,0200185	0,322956845
6991	ENSG00000177383	-0,009714179	4,032852177	0,0076335	0,934767659
6992	ENSG00000177409	0,278304563	5,163848694	1,2404226	0,276838304
6993	ENSG00000177425	0,382839166	5,134901708	1,2189195	0,280934822
6994	ENSG00000177426	0,432025126	3,921237695	3,0220772	0,095444418
6995	ENSG00000177463	-0,106056883	6,122441554	0,4836175	0,493721638
6996	ENSG00000177469	0,34507254	7,957916413	2,6673425	0,115977082
6997	ENSG00000177479	0,277070676	6,612517786	2,323951	0,140963545
6998	ENSG00000177485	0,06297137	4,379364377	0,1459171	0,705957737
6999	ENSG00000177556	-0,024495152	4,032876735	0,0227714	0,881362461
7000	ENSG00000177565	0,07909374	7,917262016	0,1614494	0,691516948
7001	ENSG00000177570	0,265734256	5,182267509	2,4741853	0,12931963
7002	ENSG00000177575	-2,146995291	6,942339677	18,266086	0,000281952
7003	ENSG00000177600	0,170162083	7,591811842	0,4375242	0,514860432
7004	ENSG00000177606	0,351613336	5,985742274	2,689126	0,11457639
7005	ENSG00000177613	0,016673273	5,25212975	0,0167532	0,89813003
7006	ENSG00000177628	0,066426346	4,237857542	0,1329926	0,718664662
7007	ENSG00000177646	0,018356215	4,808052716	0,0131016	0,909859912
7008	ENSG00000177663	-0,464941817	4,373843643	3,3799622	0,078879523
7009	ENSG00000177666	0,044838237	5,998369775	0,0303672	0,863177139
7010	ENSG00000177683	-0,05781622	5,089359409	0,1351216	0,71652443
7011	ENSG00000177694	0,447970259	4,353211966	4,1325547	0,053707658
7012	ENSG00000177697	-0,033909365	7,153692302	0,0283071	0,867852538
7013	ENSG00000177700	-0,20572577	5,602169463	0,9820058	0,331965764
7014	ENSG00000177707	0,306157073	4,580947691	1,8077388	0,191827475
7015	ENSG00000177728	0,275828205	4,675233849	1,3440013	0,258166754
7016	ENSG00000177731	-0,097404063	6,325637581	0,3036407	0,586896739
7017	ENSG00000177733	0,115164786	5,864284704	1,0487246	0,316363842
7018	ENSG00000177830	0,148590292	4,7116613	0,6332674	0,434256714
7019	ENSG00000177853	-0,089652129	6,162353133	0,2490857	0,622436794

7020	ENSG00000177879	-0,178255471	5,436720327	1,0237927	0,322096951
7021	ENSG00000177885	0,167507175	6,096724607	1,5550114	0,224843104
7022	ENSG00000177888	-0,021454167	5,884638819	0,006206	0,937887281
7023	ENSG00000177889	-0,321957049	5,932470521	3,0268995	0,095195972
7024	ENSG00000177932	0,029709682	4,115883096	0,0369655	0,849214506
7025	ENSG00000177951	0,266061336	4,3249284	1,9310485	0,177885668
7026	ENSG00000177954	0,367033512	8,634004113	3,2299136	0,085384563
7027	ENSG00000177963	0,228579693	5,244357619	2,5266661	0,125497793
7028	ENSG00000177971	-0,12313426	3,824400566	0,738593	0,517976268
7029	ENSG00000177981	-0,373235848	5,438204355	8,2797595	0,00846131
7030	ENSG00000177990	-0,040919313	7,119315499	0,0116284	0,915057282
7031	ENSG00000178028	-0,093994999	4,246247827	0,3018448	0,587997805
7032	ENSG00000178033	0,229129297	4,333792049	1,3737886	0,253102317
7033	ENSG00000178035	0,209377025	5,699576156	1,3064769	0,264735408
7034	ENSG00000178038	0,075106248	4,480943173	0,0818096	0,777407829
7035	ENSG00000178053	-0,839403853	7,136973307	6,8824559	0,01515502
7036	ENSG00000178057	-0,075977382	5,180858477	0,1975799	0,660815803
7037	ENSG00000178074	-0,139922508	4,182357466	1,0361494	0,416326489
7038	ENSG00000178104	-0,138679499	10,65208127	0,2580801	0,616256468
7039	ENSG00000178105	-0,119450382	4,582770838	0,9012434	0,372715261
7040	ENSG00000178115	0,065476554	3,581223385	0,1045073	0,778623332
7041	ENSG00000178163	-0,20623355	5,870987765	2,2411402	0,147872869
7042	ENSG00000178177	-0,132024331	4,669478728	0,4550495	0,50663872
7043	ENSG00000178188	0,25230765	4,590782845	1,7463827	0,199264851
7044	ENSG00000178202	0,145727252	5,309969127	0,7226262	0,404004031
7045	ENSG00000178209	0,315695682	6,848181786	1,6962306	0,205610828
7046	ENSG00000178229	0,145619953	3,458184454	0,7616245	0,501507191
7047	ENSG00000178234	0,054428716	5,281205204	0,3238074	0,574798115
7048	ENSG00000178252	0,232118819	5,686615811	1,5559344	0,224753061
7049	ENSG00000178295	-0,304802862	4,025974043	4,0471024	0,059647354
7050	ENSG00000178338	-0,062924278	3,719687742	0,2412343	0,746987687
7051	ENSG00000178385	0,211729851	4,97817223	2,0088571	0,169723342
7052	ENSG00000178425	-0,193831796	5,472243737	1,4511861	0,240528585
7053	ENSG00000178429	0,309888248	5,799980603	1,4314131	0,243663148
7054	ENSG00000178449	-0,547606944	5,362562221	11,383679	0,002605986
7055	ENSG00000178458	-0,046901894	4,131343497	0,1147629	0,77783774
7056	ENSG00000178464	0,386249134	6,192427059	3,2092799	0,086326551
7057	ENSG00000178467	0,315706659	4,282073209	4,5983034	0,059483656
7058	ENSG00000178498	0,057358375	4,580739186	0,1284331	0,723314768
7059	ENSG00000178502	0,404506517	3,610690648	3,698824	0,066855485
7060	ENSG00000178537	0,307727478	5,142125519	2,3004045	0,14289954
7061	ENSG00000178538	-0,269662534	4,075849304	0,8470563	0,366893219
7062	ENSG00000178567	-0,009547769	6,164041506	0,0036043	0,952643281
7063	ENSG00000178568	-0,051686308	6,197701303	0,030491	0,862901351
7064	ENSG00000178573	0,59935843	5,189241642	4,7302711	0,040117178
7065	ENSG00000178585	-0,101576683	4,240619103	0,2716955	0,607155448
7066	ENSG00000178607	0,586824906	4,019508424	5,3982116	0,029306664
7067	ENSG00000178662	-0,276063865	4,887819448	1,1782011	0,288914636
7068	ENSG00000178691	0,019723814	5,286702456	0,0228009	0,881281622
7069	ENSG00000178694	-0,077036945	4,141042547	0,4391846	0,667877013
7070	ENSG00000178695	-0,020774572	6,933900725	0,0106547	0,91867749
7071	ENSG00000178719	-0,075385234	5,385976582	0,1189003	0,733348377
7072	ENSG00000178726	-0,361735009	5,140361985	2,4169505	0,133615854
7073	ENSG00000178741	-0,592416672	7,636113141	7,5731701	0,011323633
7074	ENSG00000178761	-0,091505157	4,495989837	0,291331	0,59453142
7075	ENSG00000178764	0,052141812	4,453316952	0,072764	0,789747896
7076	ENSG00000178802	-0,323271672	5,024214964	3,6697945	0,067858793
7077	ENSG00000178852	0,14002272	4,53260492	0,5697575	0,457978179
7078	ENSG00000178878	0,051950871	5,545580857	0,0163403	0,899389723

7079	ENSG00000178904	0,316600749	4,67338415	1,7438748	0,199576359
7080	ENSG00000178913	-0,067238167	6,802056792	0,2525071	0,62005307
7081	ENSG00000178927	-0,281951868	4,711757916	1,8055808	0,192083195
7082	ENSG00000178950	0,169617251	4,567633937	0,7714625	0,388804749
7083	ENSG00000178952	-0,197497802	6,523006557	1,1806035	0,288435517
7084	ENSG00000178971	0,066859196	4,036068261	0,087077	0,770561079
7085	ENSG00000178974	-0,112182016	4,859536273	1,0144783	0,348529772
7086	ENSG00000178980	-0,586692597	8,112028794	8,6857658	0,007208502
7087	ENSG00000178982	-0,320571667	7,289135188	3,60208	0,070266662
7088	ENSG00000178988	-0,040064658	5,587894548	0,0962301	0,759171672
7089	ENSG00000178996	0,383732437	4,73268688	4,0698671	0,055412502
7090	ENSG00000179010	-0,075881721	7,240749055	0,4798352	0,495374819
7091	ENSG00000179021	-0,028068868	4,436647792	0,0296088	0,864878633
7092	ENSG00000179051	0,278400302	4,066370868	1,5605269	0,224091861
7093	ENSG00000179091	-0,434217645	7,129590696	2,6938431	0,114275699
7094	ENSG00000179094	0,738476079	5,775695248	3,6518441	0,068487784
7095	ENSG00000179104	0,440757683	3,876608839	2,4598933	0,130376921
7096	ENSG00000179119	0,199340488	4,488623499	2,0174387	0,168809368
7097	ENSG00000179134	0,229388449	4,849567851	0,9413092	0,341985672
7098	ENSG00000179144	-0,437425483	4,235057731	5,3257231	0,030305393
7099	ENSG00000179152	-0,113459515	5,393596947	0,4861292	0,49261332
7100	ENSG00000179163	0,321206251	4,617745696	2,7318824	0,111884361
7101	ENSG00000179195	-0,152233427	6,659718181	2,1632958	0,154778747
7102	ENSG00000179218	-0,06748103	7,721507673	0,1356536	0,715992575
7103	ENSG00000179222	0,298037471	6,211259493	4,2386983	0,050929831
7104	ENSG00000179262	-0,158223242	5,172907357	0,9028787	0,351841994
7105	ENSG00000179295	0,15870479	7,481763704	1,0348041	0,319536862
7106	ENSG00000179304	0,257500848	4,730342435	2,7831748	0,108751956
7107	ENSG00000179335	0,086567787	4,946242914	0,2564292	0,617380589
7108	ENSG00000179364	0,126613206	5,050686364	0,436665	0,515269636
7109	ENSG00000179387	-0,029456973	5,447408141	0,015342	0,90249483
7110	ENSG00000179406	0,279561918	3,529029815	2,3465294	0,173716404
7111	ENSG00000179409	0,183935841	3,796711034	0,865382	0,368685572
7112	ENSG00000179454	-0,151980083	5,104001829	0,5944032	0,448531746
7113	ENSG00000179456	-0,080687041	4,762281626	0,2957045	0,591795234
7114	ENSG00000179476	-0,043355111	3,534228916	0,0602454	0,848345156
7115	ENSG00000179532	-0,10650451	4,365402007	0,1827518	0,672975513
7116	ENSG00000179562	0,316952503	3,770504121	5,7481842	0,095427058
7117	ENSG00000179583	0,405172831	4,237494142	1,613256	0,216672954
7118	ENSG00000179604	-0,524569842	5,119373575	4,3036306	0,049349401
7119	ENSG00000179630	-0,05284458	3,99491048	0,0524399	0,820884943
7120	ENSG00000179632	-0,179732	5,97580494	1,1298098	0,298795984
7121	ENSG00000179776	0,124682758	5,837597607	0,3840693	0,541495413
7122	ENSG00000179813	-0,730508758	4,686525902	4,0614435	0,055646231
7123	ENSG00000179818	-0,019534893	4,765421381	0,007312	0,932592333
7124	ENSG00000179820	0,082218393	5,627466883	0,1127728	0,74003853
7125	ENSG00000179833	0,235700054	4,825796566	3,3855185	0,078604639
7126	ENSG00000179889	0,070890208	6,504980402	0,3510091	0,559274731
7127	ENSG00000179909	0,093145891	4,261475979	0,2600748	0,61490437
7128	ENSG00000179912	0,153971797	4,888635093	0,6127414	0,441704506
7129	ENSG00000179914	3,049926016	9,619595443	7,8712151	0,010012532
7130	ENSG00000179915	-0,658905287	4,544244338	5,0763818	0,034042866
7131	ENSG00000179918	-0,342899201	4,663509722	5,9964614	0,02230399
7132	ENSG00000179933	-0,282862532	4,694899326	4,3452626	0,048310099
7133	ENSG00000179941	0,006226651	4,570637829	0,0007136	0,978917199
7134	ENSG00000179950	-0,058865622	5,382278401	0,1101993	0,742908971
7135	ENSG00000179978	0,184143347	5,138001347	1,0190198	0,323203505
7136	ENSG00000179981	-0,195511356	4,395005066	1,3180793	0,262681424
7137	ENSG00000180008	0,011349533	5,723232682	0,0032055	0,955336494

7138	ENSG00000180011	-0,458279034	5,944376396	6,6196178	0,016973627
7139	ENSG00000180098	-0,134510807	3,725893538	1,5017089	0,478554636
7140	ENSG00000180104	0,002614716	5,109144337	0,0003293	0,985676606
7141	ENSG00000180155	-0,074992843	5,809359969	0,0927363	0,763452039
7142	ENSG00000180182	0,102583514	5,339729573	0,8680385	0,361096262
7143	ENSG00000180185	-0,418071293	4,242482939	6,9086743	0,014985795
7144	ENSG00000180228	-0,148481891	4,412876648	1,2819878	0,309817193
7145	ENSG00000180229	-0,532414526	6,058121487	3,5034531	0,073950636
7146	ENSG00000180263	0,117116217	5,389504209	0,4754814	0,497341491
7147	ENSG00000180304	0,209068627	5,748572044	1,7017901	0,204895109
7148	ENSG00000180329	-0,36730795	4,511221677	2,8364436	0,105606746
7149	ENSG00000180353	-0,313477748	4,613295075	1,3012196	0,265673003
7150	ENSG00000180354	0,973414927	6,33688828	21,545777	0,000112419
7151	ENSG00000180357	0,09498447	5,376963921	0,2632444	0,612769337
7152	ENSG00000180370	-0,037566064	6,347851689	0,0576656	0,812341356
7153	ENSG00000180376	-0,032325132	5,164383721	0,0461466	0,831793728
7154	ENSG00000180398	0,175312898	6,440232115	1,3188177	0,26255141
7155	ENSG00000180447	1,029535428	4,892448946	6,9696485	0,014600274
7156	ENSG00000180488	0,065614597	5,253768964	0,2069836	0,65338063
7157	ENSG00000180530	-0,036421448	6,36298419	0,027645	0,869392084
7158	ENSG00000180543	-0,130496432	5,161249817	0,5898821	0,450240945
7159	ENSG00000180573	0,434809358	4,733027872	3,1040237	0,091322535
7160	ENSG00000180581	-0,315498673	4,704983035	2,1570859	0,155393251
7161	ENSG00000180626	0,056711219	4,357335527	0,213539	0,74795932
7162	ENSG00000180628	0,071797998	7,225020625	0,1599731	0,692853789
7163	ENSG00000180667	0,309127262	4,718466375	0,9686239	0,335214748
7164	ENSG00000180694	0,132470456	4,783521051	0,5523776	0,464835993
7165	ENSG00000180747	-0,219452665	7,520428246	0,9837927	0,331535242
7166	ENSG00000180758	-0,103696772	3,837692872	0,2154951	0,64683686
7167	ENSG00000180773	-0,468025157	4,476864139	9,6285504	0,004993115
7168	ENSG00000180776	-0,009617271	5,372195129	0,0026671	0,959256123
7169	ENSG00000180787	-0,075687609	4,557188118	0,2352835	0,632199316
7170	ENSG00000180817	-0,557598316	6,286949512	6,2959609	0,019553671
7171	ENSG00000180867	0,047338678	4,950711332	0,1103862	0,742693093
7172	ENSG00000180879	0,325937765	5,72378163	2,3727876	0,137046831
7173	ENSG00000180881	-0,304659006	4,345632446	2,767088	0,109723207
7174	ENSG00000180891	-0,125951297	4,755498917	0,5943246	0,448561369
7175	ENSG00000180901	0,06282852	5,243521039	0,1130382	0,739744654
7176	ENSG00000180917	-0,058242913	4,646990147	0,1348961	0,716750219
7177	ENSG00000180957	0,069832295	6,041525121	0,2429235	0,626739404
7178	ENSG00000180964	-0,294715173	5,83809617	1,2152285	0,281646025
7179	ENSG00000180979	0,14998189	3,758584	1,7662015	0,433536931
7180	ENSG00000180992	-0,448954632	4,186831765	5,3110406	0,030512285
7181	ENSG00000181019	0,045501723	4,206176471	0,0149637	0,903697996
7182	ENSG00000181061	-0,814609682	6,790985537	4,5764319	0,043201001
7183	ENSG00000181072	-0,337279121	7,489378091	1,2187047	0,280976143
7184	ENSG00000181090	0,016110295	4,668218558	0,0080688	0,929198817
7185	ENSG00000181092	0,607410917	6,64860387	0,5720198	0,457097695
7186	ENSG00000181104	1,169836739	4,613987286	23,638429	6,48957E-05
7187	ENSG00000181163	0,090618399	8,072533806	0,1344535	0,71719399
7188	ENSG00000181191	0,358171398	4,484841421	2,6580373	0,116581528
7189	ENSG00000181192	0,224539421	4,993086017	1,2736249	0,270666137
7190	ENSG00000181222	0,423868556	5,892211491	2,588926	0,12118851
7191	ENSG00000181264	-0,039035502	4,840382704	0,0370642	0,84901604
7192	ENSG00000181315	0,028363645	5,073611078	0,0281156	0,868295846
7193	ENSG00000181381	-0,120046665	5,364220576	0,5017803	0,485802324
7194	ENSG00000181450	0,014553262	4,439310841	0,0063773	0,937037862
7195	ENSG00000181458	0,197462446	3,775931233	0,61491	0,440908125
7196	ENSG00000181467	0,28559321	4,594228452	1,355835	0,256139229

7197	ENSG00000181524	0,025904957	4,676212767	0,0215087	0,884673417
7198	ENSG00000181555	0,128235766	6,681763921	1,6374313	0,213330836
7199	ENSG00000181610	-0,359243678	5,548980445	4,6257357	0,042184532
7200	ENSG00000181619	0,088321542	3,401975352	0,3195327	0,695043765
7201	ENSG00000181666	-0,029569528	5,489756629	0,0299941	0,864011308
7202	ENSG00000181690	-0,343629686	4,624188671	1,2230271	0,28014613
7203	ENSG00000181704	-0,120252799	5,74967381	0,6479422	0,429052504
7204	ENSG00000181722	0,086061243	7,865736865	0,3821958	0,542454182
7205	ENSG00000181744	0,223817743	4,813132689	1,1444466	0,295760072
7206	ENSG00000181754	0,006838091	4,269420049	0,002095	0,963885174
7207	ENSG00000181788	0,009850645	4,15735344	0,0035221	0,953185924
7208	ENSG00000181789	0,298825408	6,506375875	5,7714654	0,024681061
7209	ENSG00000181804	-0,072800447	4,558254436	0,1275738	0,724201564
7210	ENSG00000181826	0,075232902	5,038023981	0,1060209	0,74765008
7211	ENSG00000181827	0,302882714	4,4509859	4,8289576	0,038232321
7212	ENSG00000181852	0,065430546	5,36447117	0,272766	0,60643515
7213	ENSG00000181856	-0,578122472	6,244254937	3,8115251	0,063117471
7214	ENSG00000181873	0,207560652	3,752799183	0,7868127	0,384202907
7215	ENSG00000181894	-0,026365711	3,917478436	0,0620344	0,895834766
7216	ENSG00000181904	-0,035991972	5,473079138	0,0584237	0,811136526
7217	ENSG00000181915	0,22690001	4,717046689	2,6311493	0,118311791
7218	ENSG00000181924	-0,279088599	4,822826646	3,5324237	0,072830152
7219	ENSG00000181929	-0,215879271	5,899983106	1,9987136	0,170761213
7220	ENSG00000181982	-0,114759677	4,39070093	0,6649361	0,42312048
7221	ENSG00000181991	-0,327220954	3,939046808	7,7951434	0,060892743
7222	ENSG00000182004	-0,296077661	4,655824081	1,3379915	0,259204417
7223	ENSG00000182013	0,775522655	4,406493817	10,676216	0,003370332
7224	ENSG00000182022	0,185403984	4,054342382	0,3925949	0,537078628
7225	ENSG00000182054	-0,868770073	7,325035659	11,731567	0,002301639
7226	ENSG00000182093	-0,31586054	4,236376827	2,397228	0,135135328
7227	ENSG00000182095	0,457916732	4,538040154	3,0952952	0,09175163
7228	ENSG00000182117	-0,480778614	4,92298812	5,7299848	0,025180956
7229	ENSG00000182141	-0,178962591	4,461117374	1,7417291	0,19979871
7230	ENSG00000182149	-0,039170229	6,495230297	0,2084383	0,652240908
7231	ENSG00000182150	-0,032102403	5,864341497	0,0929581	0,763168843
7232	ENSG00000182154	-0,256204058	5,781653731	1,4435578	0,241731734
7233	ENSG00000182158	0,025024458	6,17732014	0,0168288	0,897905776
7234	ENSG00000182168	0,292280859	4,384770094	1,0033129	0,326882221
7235	ENSG00000182179	0,775699214	4,337123568	11,898011	0,002169981
7236	ENSG00000182180	-0,293920436	5,610370161	6,0366154	0,021907154
7237	ENSG00000182197	0,53376298	5,499686792	13,369845	0,001304595
7238	ENSG00000182199	-0,077408618	4,316634301	0,1608392	0,692068637
7239	ENSG00000182220	0,171938644	5,96192817	1,1654813	0,291469115
7240	ENSG00000182240	0,23369921	4,979844544	1,4335571	0,243320748
7241	ENSG00000182247	0,131841856	4,160602415	1,5201429	0,402488969
7242	ENSG00000182253	-0,564734716	8,201266742	5,4006925	0,02927314
7243	ENSG00000182263	0,033813735	4,960782807	0,0789999	0,781149442
7244	ENSG00000182287	-0,513648388	6,508984117	4,2221238	0,051373843
7245	ENSG00000182307	0,145784643	4,680935892	1,5422373	0,297178254
7246	ENSG00000182326	0,110034384	8,168953989	0,1646937	0,688603934
7247	ENSG00000182359	-0,175879561	3,693985656	0,5390845	0,470196046
7248	ENSG00000182372	0,03217739	4,145098952	0,0322227	0,859104093
7249	ENSG00000182446	0,034242475	6,117697691	0,0450047	0,833855042
7250	ENSG00000182463	-0,247578808	4,728759105	1,4517371	0,24044198
7251	ENSG00000182473	0,052669559	5,379653652	0,0999077	0,754777142
7252	ENSG00000182481	-0,549358657	4,468712382	10,024235	0,004296648
7253	ENSG00000182492	1,022873618	6,876048714	8,6499156	0,007311833
7254	ENSG00000182504	-0,021024021	4,4802118	0,0248461	0,972663373
7255	ENSG00000182512	-0,428720596	5,229499467	5,7535373	0,024913684

7256	ENSG00000182534	0,149606587	8,307863086	1,0551761	0,314912173
7257	ENSG00000182541	-0,29484414	4,331707674	1,9511069	0,175736604
7258	ENSG00000182551	-0,004461732	7,003051213	0,0009228	0,976025812
7259	ENSG00000182552	-0,180795614	4,618336792	1,0151722	0,324099347
7260	ENSG00000182568	0,103790193	5,812856223	0,4567935	0,505833295
7261	ENSG00000182578	0,569191744	5,006662967	3,2846494	0,082942566
7262	ENSG00000182606	-0,087743571	7,371573002	0,1425529	0,709201025
7263	ENSG00000182621	-0,292488413	6,107421576	2,2221106	0,149569123
7264	ENSG00000182628	0,151333702	4,777396997	0,8484102	0,36651734
7265	ENSG00000182636	0,195714879	4,886996146	1,8687021	0,184745477
7266	ENSG00000182670	0,291335632	7,864479855	5,9543677	0,022728601
7267	ENSG00000182700	0,416040541	4,957035644	11,100851	0,00287654
7268	ENSG00000182704	0,159559532	4,192839735	0,1225975	0,72940488
7269	ENSG00000182718	0,160947868	8,056082887	0,3281586	0,572276774
7270	ENSG00000182732	-0,859725237	4,269580411	14,125833	0,001016374
7271	ENSG00000182752	0,090202036	4,224054669	0,0783307	0,782062043
7272	ENSG00000182768	0,176051144	4,870163204	2,3508505	0,163238545
7273	ENSG00000182809	-0,211292039	8,266600911	0,5783179	0,454660875
7274	ENSG00000182827	0,166172298	5,539966964	1,3997926	0,248754772
7275	ENSG00000182831	0,084572369	6,128864964	0,4187749	0,523902553
7276	ENSG00000182836	-0,350125251	7,463301105	2,2252985	0,149290381
7277	ENSG00000182871	0,395690296	5,378275547	4,8891607	0,037189366
7278	ENSG00000182872	0,110423728	4,467477292	0,371888	0,547926068
7279	ENSG00000182899	0,257620045	8,008137463	1,4982757	0,233268245
7280	ENSG00000182903	-0,079795681	5,314548734	0,4409224	0,513224951
7281	ENSG00000182919	-0,007973577	4,224771417	0,0042785	0,971883685
7282	ENSG00000182923	-0,284610212	5,34320653	6,3235027	0,019289958
7283	ENSG00000182934	0,04679464	6,1093715	0,127194	0,724583437
7284	ENSG00000182944	-0,031870916	6,978474808	0,066118	0,799342499
7285	ENSG00000182952	-0,084351102	5,374451376	0,4305703	0,518166583
7286	ENSG00000182957	-0,190692888	4,274163229	1,6141476	0,216550181
7287	ENSG00000182963	-0,117175042	5,50819502	0,2946954	0,592424163
7288	ENSG00000182973	-0,113813594	4,282184531	1,2087532	0,492740856
7289	ENSG00000182983	0,056235746	3,951806773	0,119953	0,77401073
7290	ENSG00000182985	-0,122798598	5,201622841	0,324147	0,574618077
7291	ENSG00000183011	-0,315568896	4,291049748	2,0235551	0,168233005
7292	ENSG00000183020	-0,184649038	5,00510779	0,9547921	0,338619779
7293	ENSG00000183023	-0,097841902	9,883895206	0,0657503	0,799896503
7294	ENSG00000183044	0,854979107	4,227593203	8,1639764	0,008885931
7295	ENSG00000183049	-0,192639044	5,181676879	0,6408943	0,431539678
7296	ENSG00000183054	-0,056592469	7,586715929	0,1696493	0,6842054
7297	ENSG00000183060	-0,231294392	3,972337644	1,9407185	0,176845645
7298	ENSG00000183087	0,049704902	6,570282756	0,0885976	0,768626739
7299	ENSG00000183091	0,673946658	8,798908319	1,3784472	0,252321897
7300	ENSG00000183098	-0,489872377	4,005241938	2,4150468	0,133761635
7301	ENSG00000183111	0,091971919	4,709035907	0,3091319	0,583556291
7302	ENSG00000183137	-0,134839395	4,077311013	0,7023755	0,431968321
7303	ENSG00000183172	-0,200827626	4,748269647	1,4109851	0,246956727
7304	ENSG00000183199	0,188567044	4,513047996	1,7582902	0,197754137
7305	ENSG00000183207	-0,083440981	4,491078604	0,174639	0,67987846
7306	ENSG00000183230	-0,094864473	7,118375939	0,0742379	0,787682841
7307	ENSG00000183255	-0,082006716	7,373123924	0,701783	0,410734946
7308	ENSG00000183258	0,003481491	4,261321619	0,0007723	0,982252914
7309	ENSG00000183283	0,086847599	6,941275357	0,7751042	0,387674286
7310	ENSG00000183291	0,131625632	6,048162714	0,4335114	0,516776527
7311	ENSG00000183298	0,148634742	4,604366837	0,3471399	0,561451893
7312	ENSG00000183309	0,229399284	4,615953347	3,4412094	0,093560107
7313	ENSG00000183337	0,218111198	4,569549895	1,2216262	0,280414784
7314	ENSG00000183340	0,212131589	3,783121556	2,5923731	0,218151415

7315	ENSG00000183354	-0,215958618	6,083328205	4,3727205	0,047663793
7316	ENSG00000183426	-0,158116627	5,138713332	0,3927026	0,537023308
7317	ENSG00000183431	-0,040275346	5,405186662	0,0907802	0,765872349
7318	ENSG00000183454	1,219586643	5,307445292	8,0962926	0,009133384
7319	ENSG00000183474	-0,028121943	5,145459794	0,0292644	0,865658688
7320	ENSG00000183475	0,150876046	3,73031439	2,0921843	0,41201121
7321	ENSG00000183486	0,079656759	4,03952086	0,1469583	0,704962615
7322	ENSG00000183495	0,07843482	4,976516816	0,133332	0,718322231
7323	ENSG00000183506	-0,014364521	4,306294007	0,0049019	0,944785387
7324	ENSG00000183508	0,403825873	5,536023119	2,8263838	0,106192502
7325	ENSG00000183513	0,024443275	4,773827799	0,0286806	0,866990036
7326	ENSG00000183520	-0,630981382	4,744741328	11,16426	0,002820473
7327	ENSG00000183527	-0,172868167	3,847897745	1,5205947	0,375303831
7328	ENSG00000183530	0,132866695	5,767165202	1,4096772	0,247127879
7329	ENSG00000183576	-0,039873844	6,769926938	0,0982352	0,756759063
7330	ENSG00000183580	-0,131003254	4,513126298	0,5328924	0,472727905
7331	ENSG00000183597	0,211965795	4,516996349	0,9793753	0,332600944
7332	ENSG00000183604	-0,19070751	6,385159601	0,7999326	0,380333052
7333	ENSG00000183605	-0,34131881	4,621587128	5,8731647	0,023576478
7334	ENSG00000183621	-0,376341287	4,474987841	8,5529119	0,007582531
7335	ENSG00000183624	-0,382968452	5,455322694	5,5821671	0,026933181
7336	ENSG00000183648	-0,695652055	6,776128782	9,4521741	0,005342773
7337	ENSG00000183666	0,163785327	4,363241399	1,1878961	0,315487117
7338	ENSG00000183688	-0,136087096	4,350541933	0,7303486	0,401541907
7339	ENSG00000183696	0,020981982	4,627546489	0,0103602	0,919805056
7340	ENSG00000183718	-0,016475023	3,617024241	0,0100007	0,921203966
7341	ENSG00000183722	-0,094524025	6,146969258	0,4522	0,507940566
7342	ENSG00000183723	0,441822876	4,407058113	3,4991973	0,074114509
7343	ENSG00000183726	0,174667474	5,84076171	1,752875	0,19841647
7344	ENSG00000183735	-0,222731058	4,999174428	1,5954078	0,219149107
7345	ENSG00000183741	0,203051997	5,316615769	0,8681491	0,36109955
7346	ENSG00000183762	0,080031391	5,397540459	0,0923026	0,763988196
7347	ENSG00000183793	0,051572568	4,216530446	0,0599756	0,808695819
7348	ENSG00000183801	0,567559133	4,063822019	4,4206992	0,046597457
7349	ENSG00000183808	0,021587911	5,140998995	0,0239321	0,878398009
7350	ENSG00000183814	0,396607386	3,720364171	2,9039169	0,101773848
7351	ENSG00000183826	-0,135758518	5,179034234	0,7077163	0,408822761
7352	ENSG00000183853	0,374957571	4,904900421	1,7846598	0,194584142
7353	ENSG00000183864	0,3215881	5,444911852	2,7624964	0,110002279
7354	ENSG00000183873	-0,400054238	7,228132888	1,6560635	0,210875505
7355	ENSG00000183878	0,131958404	5,562685299	0,0876097	0,769881323
7356	ENSG00000183889	0,037417243	5,06629893	0,0337316	0,855880625
7357	ENSG00000183955	0,045823861	4,153298455	0,1226145	0,79636958
7358	ENSG00000183963	-0,268947695	6,265413044	1,2879853	0,268052528
7359	ENSG00000183978	-0,274568089	4,527729316	3,4615626	0,07554869
7360	ENSG00000184007	-0,253646732	7,763513836	2,9466262	0,099384744
7361	ENSG00000184009	0,217113683	8,587368218	0,6266723	0,436627918
7362	ENSG00000184014	0,078835452	6,777191837	0,2778347	0,603141799
7363	ENSG00000184076	-0,608213657	5,818231683	5,6059303	0,026642543
7364	ENSG00000184083	0,171643865	4,155254601	1,4880685	0,299179261
7365	ENSG00000184110	0,203462225	7,617761786	1,0782608	0,30982881
7366	ENSG00000184117	0,265274508	4,068456562	2,3302039	0,140454693
7367	ENSG00000184178	-0,026064727	3,885011179	0,0607743	0,867134656
7368	ENSG00000184182	-0,53397377	3,922067134	16,168392	0,002688822
7369	ENSG00000184203	-0,142855521	5,936677124	0,6045642	0,444728191
7370	ENSG00000184205	0,26170628	5,54284862	1,9271941	0,178302298
7371	ENSG00000184206	0,000191688	4,163871348	9,375E-08	0,999758328
7372	ENSG00000184208	0,339483334	3,823665358	2,8344153	0,105724548
7373	ENSG00000184216	-0,141197499	4,650778472	0,5092216	0,482620138

7374	ENSG00000184220	-0,378052751	4,317925867	7,8230878	0,013608383
7375	ENSG00000184226	0,431911763	8,091259961	3,7277881	0,065871272
7376	ENSG00000184227	-0,200136104	4,779044208	1,3198812	0,262364292
7377	ENSG00000184232	0,092848771	4,493839024	0,1342743	0,717373927
7378	ENSG00000184260	0,141077974	4,458637688	0,3184828	0,577956948
7379	ENSG00000184277	-0,134166842	5,055457242	1,7700867	0,287677441
7380	ENSG00000184304	0,667956559	3,923921806	4,003972	0,057271054
7381	ENSG00000184305	-0,212320129	5,097585726	0,541365	0,469269261
7382	ENSG00000184319	0,044411802	4,203899715	0,0709195	0,792364059
7383	ENSG00000184347	0,980804699	6,329015565	7,3621559	0,012366348
7384	ENSG00000184349	0,304639348	4,609323596	3,2359009	0,085113448
7385	ENSG00000184371	0,148340633	4,305822155	0,2711975	0,607483174
7386	ENSG00000184384	0,313737171	4,751462196	1,986779	0,171992235
7387	ENSG00000184402	-0,029902802	3,828801634	0,0423144	0,848378095
7388	ENSG00000184432	0,003002006	6,947749144	0,000574	0,981090938
7389	ENSG00000184445	-0,085497162	4,066317103	0,297446	0,645662659
7390	ENSG00000184465	0,186245345	3,895039319	1,3182427	0,30739072
7391	ENSG00000184481	0,114869187	4,227234305	0,5617216	0,565368691
7392	ENSG00000184489	-0,611044401	7,138069212	3,3411578	0,080505438
7393	ENSG00000184500	1,009318972	6,876281778	45,072299	7,28961E-07
7394	ENSG00000184517	0,11063368	3,718423909	0,3769655	0,545228124
7395	ENSG00000184544	-0,379353084	5,934314749	1,4302356	0,243851454
7396	ENSG00000184557	-0,570270998	6,174127826	2,1401871	0,156951188
7397	ENSG00000184575	0,015631438	5,77991842	0,009388	0,923647573
7398	ENSG00000184584	-0,069647806	5,758662917	0,1331182	0,718537839
7399	ENSG00000184588	0,101087094	6,06235114	0,1225457	0,729459707
7400	ENSG00000184601	0,14164965	4,938196774	0,3005715	0,588781069
7401	ENSG00000184602	-0,341607251	4,954532413	3,6899129	0,067161653
7402	ENSG00000184634	0,356554706	4,878385352	2,4865777	0,128411072
7403	ENSG00000184640	-0,275215896	5,450641731	1,5764537	0,221817743
7404	ENSG00000184677	0,050380784	5,103895674	0,0667341	0,798439124
7405	ENSG00000184678	0,01074675	5,213940948	0,0033836	0,9541142
7406	ENSG00000184708	-0,075774746	4,333643061	0,2582225	0,616154297
7407	ENSG00000184719	-0,072549102	3,786817433	0,2445138	0,699353628
7408	ENSG00000184743	0,321767733	6,677529725	1,1477008	0,295090727
7409	ENSG00000184752	-0,959703963	6,842148977	13,673386	0,001180559
7410	ENSG00000184787	-0,137816631	6,156888376	1,550868	0,225442246
7411	ENSG00000184831	-0,664678096	4,454371049	13,065518	0,001448488
7412	ENSG00000184840	-0,038552713	5,624912288	0,0526386	0,820552108
7413	ENSG00000184863	0,011500385	5,759591242	0,0083004	0,928190053
7414	ENSG00000184867	0,40379548	5,099883746	10,477386	0,003616229
7415	ENSG00000184887	-0,166362497	4,700577723	0,6829992	0,417006124
7416	ENSG00000184900	-0,03785925	5,636838564	0,1458593	0,706000952
7417	ENSG00000184903	0,073786792	3,675049098	0,5418373	0,694002922
7418	ENSG00000184939	0,236704843	4,689884179	3,593007	0,070552393
7419	ENSG00000184983	-0,652832517	6,921789356	8,7272285	0,007090993
7420	ENSG00000185009	0,123306496	5,065991461	1,6019677	0,235481008
7421	ENSG00000185010	-0,755650566	7,290182761	7,1333072	0,013618697
7422	ENSG00000185015	0,148224391	3,780817154	0,7129987	0,409538894
7423	ENSG00000185043	0,040031559	4,904592812	0,0591682	0,809961308
7424	ENSG00000185070	1,549725338	6,054999094	7,2187679	0,01313532
7425	ENSG00000185088	-0,033316389	6,009627516	0,0279821	0,86860577
7426	ENSG00000185100	-0,422726731	5,702354663	4,5650221	0,043440142
7427	ENSG00000185104	-0,122020697	5,921363174	1,2970437	0,266380534
7428	ENSG00000185115	-0,035503817	3,861306705	0,1208017	0,813122927
7429	ENSG00000185122	-0,013240271	5,410667872	0,0053808	0,942155497
7430	ENSG00000185127	-0,055919962	4,182780669	0,1210659	0,808966823
7431	ENSG00000185129	0,204095854	6,786532955	2,1876511	0,152575583
7432	ENSG00000185164	0,135826442	8,579906961	0,4256003	0,520591838

7433	ENSG00000185201	-0,270690246	6,257600837	0,8957745	0,353707922
7434	ENSG00000185215	-0,134602552	5,37787829	0,4126751	0,526936211
7435	ENSG00000185219	0,09594725	5,332193147	0,2297352	0,636224253
7436	ENSG00000185222	-0,038474972	5,269525828	0,03394	0,85544133
7437	ENSG00000185238	-0,024468826	4,421767414	0,024596	0,895816395
7438	ENSG00000185246	-0,173811823	4,932975937	1,1561252	0,293367341
7439	ENSG00000185261	0,182051915	3,747216549	1,0732143	0,310938363
7440	ENSG00000185278	0,16935403	6,23728552	1,1073084	0,303545468
7441	ENSG00000185304	0,473750931	5,703476433	1,5746619	0,222072131
7442	ENSG00000185305	0,019632028	4,643186354	0,012627	0,911500398
7443	ENSG00000185339	-0,603377239	4,015221108	4,5044834	0,044734202
7444	ENSG00000185344	-0,136173113	4,54139023	1,1590759	0,292728509
7445	ENSG00000185345	-0,055396979	4,800325185	0,0812305	0,778175046
7446	ENSG00000185359	0,233111371	4,279000107	1,7381201	0,200293464
7447	ENSG00000185414	-0,175229102	4,952056694	1,3345297	0,259804581
7448	ENSG00000185418	-0,017699235	5,370318262	0,0099487	0,92140827
7449	ENSG00000185432	0,328189802	6,230220529	0,7038684	0,410080529
7450	ENSG00000185477	0,172998961	5,638383303	0,8747825	0,359304666
7451	ENSG00000185483	0,863367913	4,453946784	23,695196	6,39592E-05
7452	ENSG00000185485	-0,157281919	5,223976171	0,6574393	0,425736301
7453	ENSG00000185495	-0,076393449	3,873239243	0,173451	0,680905037
7454	ENSG00000185515	0,006430242	4,147448543	0,0021952	0,963032124
7455	ENSG00000185532	-0,292023495	6,472617503	3,4484074	0,076102685
7456	ENSG00000185551	0,168375501	5,207997632	0,7746219	0,387850958
7457	ENSG00000185567	0,149839572	4,489604164	0,1769219	0,677917162
7458	ENSG00000185585	0,042569765	4,719723066	0,029893	0,86423842
7459	ENSG00000185591	0,165280776	6,269847685	0,9255175	0,345988012
7460	ENSG00000185596	-0,039908921	4,582290404	0,0497011	0,825542636
7461	ENSG00000185608	-0,274776574	4,821898598	1,7792041	0,19524292
7462	ENSG00000185619	-0,087312732	4,785128362	0,5088772	0,482741895
7463	ENSG00000185624	0,211376907	7,113000145	1,6991286	0,205237361
7464	ENSG00000185627	-0,27391608	5,807669513	5,2340102	0,031589306
7465	ENSG00000185630	0,241429776	6,95162467	1,5149445	0,230765167
7466	ENSG00000185641	0,007358269	5,200139931	0,0008426	0,977092109
7467	ENSG00000185650	0,346973227	6,283508028	1,9475674	0,176113518
7468	ENSG00000185651	-0,363763104	5,816817101	8,8915451	0,006629734
7469	ENSG00000185658	-0,029992173	7,639866246	0,0317096	0,860217531
7470	ENSG00000185684	0,114793186	3,738937082	0,234507	0,632759083
7471	ENSG00000185710	-0,230625433	6,984446977	1,0336802	0,319821119
7472	ENSG00000185716	0,012554866	4,309830354	0,0112857	0,916310033
7473	ENSG00000185721	-0,306902653	5,406048919	3,3897831	0,078474026
7474	ENSG00000185722	0,196067425	5,330252702	1,5479544	0,225907837
7475	ENSG00000185728	-0,032225429	6,577503958	0,0554239	0,815946807
7476	ENSG00000185739	-0,443444315	8,839590745	2,0729664	0,163337689
7477	ENSG00000185745	-0,016485222	5,130391398	0,0043527	0,947965144
7478	ENSG00000185787	-0,12907217	7,882638018	0,7997592	0,380351918
7479	ENSG00000185808	-0,244632415	4,382505981	2,6977725	0,128940452
7480	ENSG00000185825	-0,130188653	5,942534479	0,4655352	0,50182977
7481	ENSG00000185829	-0,505207241	4,675614112	3,0795846	0,092529854
7482	ENSG00000185834	0,223551936	3,981978635	0,9132675	0,349138362
7483	ENSG00000185842	-0,136655879	3,745780367	0,4195384	0,523549921
7484	ENSG00000185864	-0,045014899	6,303111597	0,0244358	0,877138557
7485	ENSG00000185880	-0,512870746	6,171649545	11,600717	0,002408165
7486	ENSG00000185885	-0,191027457	4,993590879	1,4716498	0,237338586
7487	ENSG00000185896	-0,013289549	6,349204068	0,0087874	0,92612028
7488	ENSG00000185909	0,047890073	4,731321116	0,0775307	0,783147991
7489	ENSG00000185917	0,033043938	3,775287633	0,0658435	0,884496343
7490	ENSG00000185946	-0,188244846	5,07755325	1,4906286	0,234428108
7491	ENSG00000185947	0,028997673	4,261168605	0,0191383	0,891168829

7492	ENSG00000185950	-0,141673114	5,143124376	0,3768041	0,545313476
7493	ENSG00000185963	0,016238755	5,372047806	0,0261951	0,945425312
7494	ENSG00000185973	-0,286830176	5,45938878	3,5058949	0,073856798
7495	ENSG00000185986	-0,174118845	4,621138763	1,0782245	0,309836774
7496	ENSG00000185989	0,13640148	4,418059473	0,3567911	0,556101041
7497	ENSG00000186001	0,145742056	5,689880953	1,4600385	0,239099734
7498	ENSG00000186017	0,005458587	4,158666678	0,0015204	0,96923096
7499	ENSG00000186020	0,030826583	4,802470354	0,0428796	0,837765677
7500	ENSG00000186063	0,027746039	6,185842534	0,0371682	0,848802662
7501	ENSG00000186073	0,047867907	4,480583786	0,0480616	0,828395748
7502	ENSG00000186076	0,092550057	5,663768426	0,3068967	0,584911303
7503	ENSG00000186088	0,035995567	4,722016554	0,0774522	0,783246247
7504	ENSG00000186104	-0,310475865	3,858031699	6,1726189	0,082060129
7505	ENSG00000186106	-0,304461736	4,899433563	1,5728204	0,22233396
7506	ENSG00000186130	-0,126179176	4,252913972	0,2922587	0,593948786
7507	ENSG00000186141	0,193683986	4,232526047	3,5613351	0,228115601
7508	ENSG00000186166	-0,093258945	4,461366595	0,2700594	0,608233501
7509	ENSG00000186184	0,027875846	5,09854912	0,0830076	0,823623867
7510	ENSG00000186260	-0,075762475	6,218544502	0,2371403	0,630865584
7511	ENSG00000186272	-0,050201778	3,660559423	0,2147575	0,795800681
7512	ENSG00000186283	0,197689703	3,815486661	1,4838752	0,322569488
7513	ENSG00000186298	-0,872701131	7,373106006	46,200186	5,95737E-07
7514	ENSG00000186300	-0,084091304	3,984952531	0,5166252	0,631185488
7515	ENSG00000186310	0,6847218	3,938141046	6,2788011	0,019702138
7516	ENSG00000186314	-0,484748425	4,622194435	5,2672606	0,031138627
7517	ENSG00000186318	0,467836293	4,180354337	7,2224436	0,01311496
7518	ENSG00000186340	0,670006499	4,823301869	2,9076626	0,101565859
7519	ENSG00000186350	0,128619062	5,126767584	0,3073282	0,584649177
7520	ENSG00000186376	0,081389773	4,256035507	0,40946	0,619487047
7521	ENSG00000186399	0,097746363	3,459870548	0,1930802	0,664446187
7522	ENSG00000186432	0,098831506	7,382243694	0,4267683	0,520006908
7523	ENSG00000186439	-0,561229597	8,462312641	3,2537038	0,084313142
7524	ENSG00000186448	0,113734312	4,988352594	1,6435073	0,278653273
7525	ENSG00000186462	0,059022019	3,836425382	0,0579046	0,811960581
7526	ENSG00000186468	0,152875707	7,530425951	0,363799	0,552277274
7527	ENSG00000186469	0,290789806	4,345537164	1,5329605	0,228097954
7528	ENSG00000186470	0,713625383	4,859708197	6,9867128	0,014494357
7529	ENSG00000186472	0,2096199	4,334023334	1,1113109	0,302693245
7530	ENSG00000186480	-0,142170278	5,169744291	0,3243698	0,574487537
7531	ENSG00000186522	0,16475322	5,886415461	1,1443235	0,295785444
7532	ENSG00000186532	-0,139749926	3,940964242	0,9511749	0,393169282
7533	ENSG00000186566	0,08910605	5,689058328	0,2346214	0,632676519
7534	ENSG00000186575	-0,048924888	4,998918764	0,0639178	0,802642476
7535	ENSG00000186591	0,343167372	6,914921993	6,267053	0,019776166
7536	ENSG00000186594	-0,256355784	5,878110027	1,8406363	0,187980685
7537	ENSG00000186625	-0,082330686	4,071373559	0,8790778	0,639897528
7538	ENSG00000186628	-0,154185168	5,871146711	0,2212426	0,642503051
7539	ENSG00000186635	0,250741169	5,177748452	1,671211	0,208870605
7540	ENSG00000186642	0,26811879	4,772442872	1,1860524	0,287352747
7541	ENSG00000186660	-0,143590619	6,036601531	1,8914079	0,182182348
7542	ENSG00000186687	-0,293429099	5,608984952	2,9603965	0,098689516
7543	ENSG00000186716	0,27882931	4,682472488	0,9880521	0,330512132
7544	ENSG00000186812	0,117519023	5,210616961	1,1502018	0,294539429
7545	ENSG00000186814	0,196542384	5,220804299	1,0787034	0,309731748
7546	ENSG00000186815	0,179136263	6,277822284	0,4420337	0,512722324
7547	ENSG00000186834	-0,043481634	4,643024983	0,0759197	0,785353442
7548	ENSG00000186868	0,31051382	3,901672911	0,6064599	0,444024295
7549	ENSG00000186908	-0,233439822	6,042049742	1,4325553	0,243480665
7550	ENSG00000186918	0,493742392	4,82555988	5,0376319	0,034668793

7551	ENSG00000186951	0,031008895	6,373582481	0,0195506	0,890010771
7552	ENSG00000187051	-0,261293375	4,675234126	1,6673785	0,209375609
7553	ENSG00000187079	-0,272304686	7,225135584	3,7346526	0,065596877
7554	ENSG00000187097	0,077371877	3,937359879	0,3629999	0,670991975
7555	ENSG00000187098	-0,105548746	6,358077537	0,2163001	0,646225687
7556	ENSG00000187109	-0,017358486	8,48399218	0,0102068	0,920396025
7557	ENSG00000187118	-0,384643895	4,208215783	8,3924586	0,015841341
7558	ENSG00000187134	0,095956576	6,232866765	0,0592977	0,809757706
7559	ENSG00000187147	-0,145135424	4,646743898	1,0121706	0,324773082
7560	ENSG00000187164	0,438088893	4,386659932	3,773623	0,064347347
7561	ENSG00000187187	0,025661547	4,403936335	0,0374364	0,848263663
7562	ENSG00000187189	-0,146219707	5,21552898	0,6530703	0,427256887
7563	ENSG00000187229	-0,308095997	3,599746527	0,6573566	0,425765006
7564	ENSG00000187231	-0,154559169	6,170648179	1,2706354	0,271174457
7565	ENSG00000187239	0,298431288	5,060859361	2,3193381	0,141340339
7566	ENSG00000187240	0,350285264	6,064515013	2,2065538	0,150938339
7567	ENSG00000187257	0,047612746	4,751225222	0,1395055	0,712165331
7568	ENSG00000187325	-0,297844861	5,337581365	1,2802625	0,269453955
7569	ENSG00000187391	-0,007984987	5,151051746	0,0040338	0,949902593
7570	ENSG00000187446	0,158487116	6,554043599	1,3730337	0,253187832
7571	ENSG00000187474	0,478505736	3,867571189	2,4817591	0,128763453
7572	ENSG00000187498	-0,131956998	8,128067305	0,3276663	0,572563079
7573	ENSG00000187514	0,332745788	7,539840862	1,5378067	0,227387162
7574	ENSG00000187522	-0,18041836	3,770020582	1,3721499	0,349665226
7575	ENSG00000187555	0,00764906	6,416986584	0,0041453	0,949216264
7576	ENSG00000187601	-0,131511203	4,28334515	0,6690391	0,440182976
7577	ENSG00000187627	0,424297158	5,638275339	1,3806239	0,251958327
7578	ENSG00000187642	-0,441504282	4,785098929	2,2582291	0,1464471
7579	ENSG00000187653	0,6420163	6,208612766	4,4112005	0,046814166
7580	ENSG00000187672	-0,368385531	3,490336773	2,0688842	0,163735532
7581	ENSG00000187676	-0,255535572	5,453164394	1,3176632	0,262754733
7582	ENSG00000187678	0,871959632	4,637149358	8,2171805	0,008696602
7583	ENSG00000187699	-0,523781336	5,100640032	10,364725	0,003782211
7584	ENSG00000187713	-0,14804131	4,303936253	1,2910305	0,302545162
7585	ENSG00000187720	0,045338347	6,423618277	0,0170697	0,897181841
7586	ENSG00000187735	0,048902715	6,125592446	0,1278601	0,723894534
7587	ENSG00000187742	-0,058313627	5,677582788	0,1498998	0,702173196
7588	ENSG00000187764	0,26807105	4,547117909	2,0796197	0,162691761
7589	ENSG00000187778	0,081907948	5,323469251	0,2796475	0,601974308
7590	ENSG00000187790	-0,120543884	4,174621435	0,5685597	0,458445503
7591	ENSG00000187837	-0,167361872	5,946695145	0,26083	0,614394183
7592	ENSG00000187866	-0,160114714	4,01315405	1,2271814	0,340703407
7593	ENSG00000187955	0,90310544	7,308565127	7,282552	0,012786913
7594	ENSG00000188021	0,120889613	5,133565626	0,553784	0,464274818
7595	ENSG00000188026	-0,188756566	5,292891028	0,9877095	0,330594254
7596	ENSG00000188070	0,280686628	4,07154907	2,6232635	0,118873331
7597	ENSG00000188092	-0,277659872	4,780545739	5,2622496	0,033739871
7598	ENSG00000188153	0,010097608	5,88878833	0,0025468	0,960184923
7599	ENSG00000188171	0,188106708	3,682500139	1,8130191	0,310732998
7600	ENSG00000188177	-0,039504104	5,996212648	0,0628554	0,804253866
7601	ENSG00000188186	0,029743503	4,728281904	0,0233065	0,879987761
7602	ENSG00000188215	0,291780501	4,671319681	1,0753644	0,31046496
7603	ENSG00000188227	0,25072966	3,630893715	1,9998078	0,217063487
7604	ENSG00000188229	-0,547887276	6,926569478	3,7205234	0,066116567
7605	ENSG00000188234	0,050374088	4,080397639	0,1082903	0,796913102
7606	ENSG00000188243	0,231441205	5,554680763	0,9592305	0,33752191
7607	ENSG00000188257	0,385231349	7,445110603	0,5373326	0,470910083
7608	ENSG00000188283	0,039832663	4,273908341	0,081464	0,782063251
7609	ENSG00000188313	-0,515993483	5,497408988	5,8497464	0,023854262

7610	ENSG00000188321	0,02199288	4,666146743	0,0208243	0,88650952
7611	ENSG00000188342	-0,415609178	4,979365696	4,7683592	0,03939244
7612	ENSG00000188343	0,011388239	5,080335006	0,0049009	0,944790563
7613	ENSG00000188352	0,007186025	6,437061005	0,002048	0,964292688
7614	ENSG00000188419	-0,026425459	4,889325015	0,0275889	0,869523367
7615	ENSG00000188428	-0,026404349	3,66937347	0,0398738	0,943062252
7616	ENSG00000188529	-0,061443506	6,458599862	0,1696171	0,684244077
7617	ENSG00000188536	2,088463784	6,031125856	15,375851	0,000679087
7618	ENSG00000188549	-0,261577054	5,540267212	1,1249543	0,299812359
7619	ENSG00000188554	0,211097806	7,236009523	3,6884555	0,067168084
7620	ENSG00000188559	-0,035955752	6,142550871	0,0386553	0,845851663
7621	ENSG00000188612	0,006543757	6,270621483	0,002601	0,959762051
7622	ENSG00000188636	-0,020167432	5,052549896	0,0160334	0,900333713
7623	ENSG00000188641	0,222658453	5,954824591	0,7558138	0,393580703
7624	ENSG00000188643	-0,2343633	4,876287343	1,4987575	0,233195416
7625	ENSG00000188647	-0,133797972	6,799090561	0,3763816	0,54553706
7626	ENSG00000188677	-0,539794983	5,462706017	5,2571373	0,031285496
7627	ENSG00000188690	-0,195977629	5,271361272	2,0156488	0,168991202
7628	ENSG00000188706	-0,393116794	4,086967332	4,9219744	0,036615074
7629	ENSG00000188725	0,041886024	4,575390354	0,0510073	0,823304798
7630	ENSG00000188730	-0,557883612	4,687512917	3,4684041	0,075312701
7631	ENSG00000188738	-0,081969414	4,603101738	0,1012354	0,753209153
7632	ENSG00000188783	0,431389339	6,938391652	1,4716844	0,237333239
7633	ENSG00000188785	-0,056061142	4,283686815	0,2253183	0,728561487
7634	ENSG00000188786	0,049946019	4,060865697	0,0898116	0,793427104
7635	ENSG00000188801	0,013397168	3,40627721	0,0054648	0,941706853
7636	ENSG00000188811	0,029617669	5,112334	0,0314481	0,860789332
7637	ENSG00000188818	-0,110510343	3,715598678	0,1549761	0,697432858
7638	ENSG00000188846	0,040490506	7,992878093	0,0445936	0,834604
7639	ENSG00000188856	-0,335519698	4,453857283	1,6132947	0,216667624
7640	ENSG00000188895	0,06719083	5,613351739	0,1825629	0,673134122
7641	ENSG00000188906	0,108913944	5,826974505	0,4430964	0,512220732
7642	ENSG00000188917	-0,083439157	4,391973788	0,4027226	0,531896897
7643	ENSG00000188921	0,119619932	4,080962828	0,5039277	0,484880355
7644	ENSG00000188938	0,095123522	5,471907452	1,1113363	0,302650186
7645	ENSG00000188976	-0,024974047	4,700363456	0,022358	0,882435871
7646	ENSG00000188994	-0,090804935	6,94410048	0,3451178	0,562585761
7647	ENSG00000189014	-0,310684249	3,729001063	3,3209353	0,109738245
7648	ENSG00000189042	-0,107847704	3,468783578	0,3928357	0,624245585
7649	ENSG00000189043	-0,793349586	9,322748449	8,8304604	0,006807482
7650	ENSG00000189056	0,213814993	5,899572789	0,1737412	0,680653923
7651	ENSG00000189058	-1,629290576	6,934080915	26,503739	3,18657E-05
7652	ENSG00000189060	-0,300236829	5,779901976	3,0007221	0,096553725
7653	ENSG00000189067	-0,390898019	6,122712148	3,7816192	0,064085623
7654	ENSG00000189077	-0,216018909	5,293614247	1,008395	0,325685646
7655	ENSG00000189079	0,0972523	5,896595902	0,8701585	0,360521114
7656	ENSG00000189091	0,149904841	6,530582229	1,3984347	0,248967072
7657	ENSG00000189136	0,002135782	3,448963364	0,0002045	0,988712617
7658	ENSG00000189144	0,099714161	3,53114606	0,5559486	0,617313284
7659	ENSG00000189164	0,069052663	3,82227288	0,1986153	0,701491181
7660	ENSG00000189171	-0,312795585	5,059300956	2,3432531	0,139399793
7661	ENSG00000189180	-0,059889615	6,118552171	0,243421	0,626385396
7662	ENSG00000189184	0,38037429	5,261589078	0,7860835	0,3844197
7663	ENSG00000189195	0,169788016	4,26638927	1,0453227	0,317169568
7664	ENSG00000189212	-0,222943278	6,966793219	0,2887036	0,596188117
7665	ENSG00000189221	-0,192940077	7,195787274	0,4849162	0,493148033
7666	ENSG00000189223	0,137038557	4,985908613	0,152378	0,699847627
7667	ENSG00000189241	0,201736691	6,449819111	2,9682731	0,098221494
7668	ENSG00000189266	-0,003087548	6,756147044	0,0002092	0,988583165

7669	ENSG00000189306	-0,10113327	4,268170176	0,1508549	0,701274378
7670	ENSG00000189308	0,120450526	4,039546256	0,9847014	0,434670387
7671	ENSG00000189319	0,194290882	4,450464791	0,7932897	0,382285242
7672	ENSG00000189339	0,285920017	5,124038672	1,9979077	0,170844
7673	ENSG00000189343	0,105831646	6,259317336	0,3409407	0,564942385
7674	ENSG00000189350	-0,873817392	4,5509864	10,204707	0,004015028
7675	ENSG00000189362	-0,074466841	3,689738008	0,2462012	0,674560089
7676	ENSG00000189369	0,021785617	3,64466406	0,0340993	0,917853628
7677	ENSG00000189403	-0,151147231	7,784438567	0,5106936	0,481994859
7678	ENSG00000189423	0,200653492	4,06522339	0,364061	0,552135312
7679	ENSG00000196072	0,06744139	4,447987587	0,1036866	0,750344001
7680	ENSG00000196083	0,180439016	4,086912016	0,7400168	0,39849115
7681	ENSG00000196109	0,919078525	3,73403589	11,401857	0,002589035
7682	ENSG00000196110	0,370250796	3,480750541	6,4644055	0,06734304
7683	ENSG00000196116	0,081102128	4,331595976	0,7657289	0,541483362
7684	ENSG00000196126	0,834499386	6,32811655	10,083471	0,004201892
7685	ENSG00000196139	0,280255116	4,939165737	0,6838695	0,416713726
7686	ENSG00000196141	-0,029704931	6,24542523	0,0825656	0,776401885
7687	ENSG00000196150	-0,008306709	3,855824523	0,0036129	0,95258537
7688	ENSG00000196151	-0,149931394	3,647583469	1,5057938	0,436242433
7689	ENSG00000196154	-0,315287777	6,196392277	1,1812303	0,288310692
7690	ENSG00000196159	0,264985405	6,818552189	2,9938023	0,096900019
7691	ENSG00000196177	-0,101433883	6,206699451	0,2536469	0,619285424
7692	ENSG00000196182	-0,280431996	5,517437424	2,7413954	0,111295518
7693	ENSG00000196187	-0,153714578	4,777758972	0,3932557	0,53673912
7694	ENSG00000196199	0,268355901	6,126414374	5,5084778	0,02782427
7695	ENSG00000196205	0,445770888	11,17475632	2,8026928	0,107548326
7696	ENSG00000196214	0,020320035	4,450985491	0,0245409	0,876871982
7697	ENSG00000196227	0,004716198	4,785603079	0,0007907	0,977808595
7698	ENSG00000196230	-0,222629694	7,44204451	1,3165906	0,26294381
7699	ENSG00000196233	-0,034418713	6,19972942	0,1181202	0,73417841
7700	ENSG00000196235	0,268931255	5,568318803	1,8679249	0,184861469
7701	ENSG00000196236	0,038903419	4,938657808	0,0570119	0,813387107
7702	ENSG00000196247	0,122986537	3,795948456	0,6890109	0,461344681
7703	ENSG00000196262	-0,10398634	7,92294781	0,417338	0,524609162
7704	ENSG00000196263	0,264870061	4,133480902	8,0876883	0,095199607
7705	ENSG00000196268	-0,11285365	5,152913372	0,3598718	0,554413819
7706	ENSG00000196275	-0,034698201	5,536643355	0,0528333	0,820226632
7707	ENSG00000196290	-0,235080463	4,063881813	3,0111733	0,161146559
7708	ENSG00000196295	-0,100203231	5,870830667	0,271118	0,607535495
7709	ENSG00000196305	-0,023920751	6,82302516	0,0582974	0,811329382
7710	ENSG00000196312	-0,030586619	4,51185496	0,0273677	0,870042664
7711	ENSG00000196313	0,037830223	5,132687991	0,0231033	0,880507783
7712	ENSG00000196323	-0,044892257	6,695381723	0,1268659	0,724923481
7713	ENSG00000196352	0,055253934	6,406511775	0,0256521	0,874144457
7714	ENSG00000196363	0,118981456	4,140468474	0,7101693	0,523561341
7715	ENSG00000196365	0,066804882	5,30093125	0,1197916	0,732391429
7716	ENSG00000196367	0,155106537	5,847455542	0,4581577	0,505204834
7717	ENSG00000196369	0,291036796	5,643639525	1,4011213	0,248567537
7718	ENSG00000196387	0,095057771	3,965476492	0,8800017	0,583017601
7719	ENSG00000196396	0,064899943	4,950681572	0,2505184	0,683899916
7720	ENSG00000196405	0,375739694	4,900838647	5,3512198	0,029949833
7721	ENSG00000196411	-0,011269102	4,516320903	0,0032923	0,954736554
7722	ENSG00000196417	-0,064687923	4,08458649	0,2372717	0,708104895
7723	ENSG00000196418	0,361824821	4,464358603	1,774035	0,195869637
7724	ENSG00000196419	-0,224738043	7,546589175	1,7045729	0,204538014
7725	ENSG00000196428	0,27338545	5,534128155	1,8268221	0,189584353
7726	ENSG00000196437	0,008472136	3,656313281	0,0019571	0,965094178
7727	ENSG00000196440	0,137399597	4,248039651	0,6355208	0,433451165

7728	ENSG00000196455	0,014645478	5,224452705	0,0252809	0,875045485
7729	ENSG00000196458	-0,103047619	4,799998576	0,6424877	0,474687301
7730	ENSG00000196459	-0,001694796	3,632765421	0,0001659	0,997961944
7731	ENSG00000196470	-0,136797474	3,590906163	0,661354	0,50533905
7732	ENSG00000196482	-0,323242664	5,654758258	1,0631209	0,31317387
7733	ENSG00000196498	-0,034831519	5,487015082	0,0254826	0,874557263
7734	ENSG00000196502	0,094731431	4,040891709	0,1992658	0,659468419
7735	ENSG00000196504	-0,094657074	6,371405247	0,4138876	0,526326136
7736	ENSG00000196505	0,044696497	5,52519037	0,0931778	0,762904598
7737	ENSG00000196507	-0,030448338	4,404270087	0,0528924	0,868252067
7738	ENSG00000196510	-0,302853837	4,805747733	10,424875	0,017199042
7739	ENSG00000196511	-0,257773548	3,506118494	4,0606298	0,220768038
7740	ENSG00000196526	0,231027683	4,990153671	1,122869	0,300250282
7741	ENSG00000196531	0,233138319	8,183789538	1,4118175	0,24682141
7742	ENSG00000196535	0,377733376	6,536621596	1,7446188	0,199483882
7743	ENSG00000196547	-0,327718541	5,424361662	1,4527665	0,240280272
7744	ENSG00000196549	0,00061566	5,228292054	1,198E-06	0,999136118
7745	ENSG00000196562	0,244287712	5,08009229	2,1189328	0,158937437
7746	ENSG00000196569	-0,095537929	9,019271817	0,2300296	0,635997562
7747	ENSG00000196576	0,625262831	5,713379281	9,6272918	0,004995519
7748	ENSG00000196586	0,049155443	5,266941456	0,13794	0,713708751
7749	ENSG00000196588	-0,096689297	4,135730298	0,1263616	0,725458325
7750	ENSG00000196591	-0,158409052	5,55362585	2,5975118	0,142931824
7751	ENSG00000196597	-0,013169062	3,69950503	0,0100985	0,933627946
7752	ENSG00000196605	0,147906542	3,672281341	1,2675778	0,456031872
7753	ENSG00000196616	0,244924327	8,923697896	0,1760265	0,678684574
7754	ENSG00000196628	0,098067267	7,599696642	0,3558098	0,556632208
7755	ENSG00000196642	-0,170047199	5,180879702	0,6882447	0,415248613
7756	ENSG00000196646	0,060744751	3,993639023	0,3488779	0,695791505
7757	ENSG00000196652	-0,139378924	4,049330054	2,0768478	0,400370426
7758	ENSG00000196655	-0,048695021	4,35205538	0,1191915	0,784282308
7759	ENSG00000196656	-0,1036208	5,533559883	0,097047	0,758195116
7760	ENSG00000196663	0,20785823	4,540502648	1,0347824	0,319568801
7761	ENSG00000196670	-0,025549905	4,594421417	0,0445857	0,925531502
7762	ENSG00000196678	-0,118523845	3,767214017	0,6270323	0,551138511
7763	ENSG00000196683	0,03524962	7,212137732	0,035366	0,852472104
7764	ENSG00000196693	-0,182468959	5,037646797	1,1755863	0,289437311
7765	ENSG00000196704	-0,034085655	5,532318234	0,0469305	0,83039395
7766	ENSG00000196705	-0,150453847	4,926601952	0,8460941	0,367160692
7767	ENSG00000196712	0,045073727	7,441446336	0,1275448	0,724220303
7768	ENSG00000196715	0,288746408	5,545209251	1,4936496	0,233969025
7769	ENSG00000196724	-0,038627938	4,121341168	0,0842654	0,839225723
7770	ENSG00000196730	1,220166259	4,737941747	5,7471087	0,02498632
7771	ENSG00000196735	1,473700596	4,624315605	18,408029	0,000270515
7772	ENSG00000196743	0,144895777	5,544510223	1,3399115	0,258831475
7773	ENSG00000196757	-0,025199969	3,945394607	0,0367563	0,926180428
7774	ENSG00000196776	0,007883852	6,891332967	0,0024384	0,96104035
7775	ENSG00000196781	0,419132081	3,860124332	2,5164174	0,126254115
7776	ENSG00000196792	-0,088747765	6,039701167	0,2767387	0,603858418
7777	ENSG00000196821	-0,450979731	6,963103561	6,6855667	0,016495613
7778	ENSG00000196850	-0,591639014	6,272394498	5,8981776	0,023340014
7779	ENSG00000196862	0,084176031	5,442921269	0,2083594	0,652316898
7780	ENSG00000196865	0,024526799	7,456306424	0,0157346	0,901261668
7781	ENSG00000196867	-0,142206201	3,93760298	1,989185	0,401957931
7782	ENSG00000196873	-0,361271945	5,887874819	8,1656643	0,008861105
7783	ENSG00000196911	-0,102661892	5,317058172	0,2524139	0,620133746
7784	ENSG00000196912	0,083767437	5,927177192	0,3021526	0,587808753
7785	ENSG00000196914	0,082094685	8,310039584	0,4104212	0,528035127
7786	ENSG00000196922	-0,454487228	5,372677617	11,696356	0,002322372

7787	ENSG00000196924	0,116826463	8,19449621	0,1867501	0,669640377
7788	ENSG00000196935	-0,102265457	4,142007084	0,2539241	0,619095025
7789	ENSG00000196937	0,223756137	6,034118512	2,1907423	0,152345409
7790	ENSG00000196943	0,263700013	4,230438571	3,1153878	0,120741415
7791	ENSG00000196950	0,454506628	5,005676263	3,5123235	0,073610405
7792	ENSG00000196954	-0,203933091	5,291543687	0,7044416	0,409892808
7793	ENSG00000196961	0,080505256	5,091652344	0,1925862	0,664847822
7794	ENSG00000196967	0,106711675	4,320530206	0,5629211	0,524169608
7795	ENSG00000196968	0,151032275	4,325672323	0,8996088	0,352699097
7796	ENSG00000196975	0,455841883	5,019975801	4,2825365	0,049864543
7797	ENSG00000196998	-0,047271816	4,425049653	0,130286	0,72260531
7798	ENSG00000197006	-0,084174157	5,787190368	0,3409222	0,564941212
7799	ENSG00000197008	-0,03688066	3,827548363	0,0657886	0,853019653
7800	ENSG00000197013	0,196604227	3,745874111	1,1051428	0,304007923
7801	ENSG00000197016	0,144176781	4,088612543	1,5734373	0,327166406
7802	ENSG00000197020	-0,050282431	4,062983024	0,0787591	0,781482865
7803	ENSG00000197024	0,072755575	4,082303882	0,3625755	0,675961482
7804	ENSG00000197037	0,065555625	3,604475414	0,1610863	0,774375473
7805	ENSG00000197043	-0,110300794	7,793329702	0,3431198	0,563710574
7806	ENSG00000197044	0,028488866	4,570028735	0,0132631	0,909308492
7807	ENSG00000197045	-0,155328292	6,298787324	0,2961281	0,591531591
7808	ENSG00000197050	-0,004613023	3,754028178	0,0005808	0,980979957
7809	ENSG00000197056	0,131797474	4,034369944	0,9699471	0,395142187
7810	ENSG00000197061	0,177343391	4,819574718	0,4579924	0,505280911
7811	ENSG00000197062	-0,086023308	4,242713326	0,2965832	0,653215634
7812	ENSG00000197063	0,057560622	4,375633236	0,1146235	0,737996916
7813	ENSG00000197077	0,106245031	5,196467332	0,180778	0,674637985
7814	ENSG00000197081	-0,171404899	7,015319563	0,6384876	0,432394167
7815	ENSG00000197102	0,176311446	8,956953224	0,7975972	0,38099174
7816	ENSG00000197111	0,124495402	6,644494191	0,8833296	0,356977144
7817	ENSG00000197121	-0,54482709	6,088986247	3,2983038	0,082345983
7818	ENSG00000197128	-0,138742297	4,755744943	1,0167359	0,323706586
7819	ENSG00000197140	0,342820235	3,845982232	1,9268932	0,178334873
7820	ENSG00000197142	0,158525415	4,194002941	0,5690213	0,458265296
7821	ENSG00000197147	0,026065508	4,098395213	0,019896	0,889050053
7822	ENSG00000197157	0,1741733	6,456468123	1,1845945	0,287641929
7823	ENSG00000197170	-0,160329295	6,009719521	1,37034	0,253640825
7824	ENSG00000197183	0,047293896	3,709699266	0,0542339	0,855835921
7825	ENSG00000197217	-0,227813267	6,081995854	2,2987787	0,143034373
7826	ENSG00000197223	-0,284322912	4,111615711	2,8170348	0,106740269
7827	ENSG00000197226	0,327868907	5,588689829	1,7339453	0,200815679
7828	ENSG00000197256	0,298991542	7,222619429	2,7686601	0,109627843
7829	ENSG00000197258	0,317969543	5,721501692	2,9448394	0,099528112
7830	ENSG00000197265	-0,05400829	4,02733192	0,1612083	0,753155417
7831	ENSG00000197296	-0,137001026	7,122653946	0,2689057	0,60899617
7832	ENSG00000197302	-0,091980881	4,763621105	0,5581345	0,462519776
7833	ENSG00000197312	0,063183374	5,851280783	0,1229325	0,729050871
7834	ENSG00000197321	0,350796116	8,734422845	2,4240291	0,133075451
7835	ENSG00000197323	0,066518316	5,859522987	0,3350494	0,568279996
7836	ENSG00000197324	0,128767358	5,850288606	0,4661665	0,50154277
7837	ENSG00000197329	0,089266008	4,434722397	0,2468805	0,62397346
7838	ENSG00000197343	-0,03013833	6,318109281	0,0513182	0,822769794
7839	ENSG00000197345	-0,488951297	4,793101195	7,2936013	0,012727603
7840	ENSG00000197375	0,173555552	4,010400266	0,7083149	0,40862763
7841	ENSG00000197381	-0,143529412	4,799838767	0,9776384	0,333006901
7842	ENSG00000197386	-0,015148344	6,040548613	0,0058369	0,939758845
7843	ENSG00000197429	0,320038521	4,133077168	5,3774841	0,047094856
7844	ENSG00000197442	0,324828053	5,01065489	2,1397654	0,156990303
7845	ENSG00000197448	-0,132279504	6,574452892	0,8649654	0,361938466

7846	ENSG00000197451	-0,286104252	5,549064201	3,234152	0,085192538
7847	ENSG00000197461	0,159727737	4,150934134	1,3042809	0,321571819
7848	ENSG00000197498	-0,41925703	4,838780386	5,2562473	0,031298445
7849	ENSG00000197535	-0,039049327	5,848472179	0,0871043	0,770517013
7850	ENSG00000197536	0,001564171	3,756875686	4,381E-05	0,994775904
7851	ENSG00000197548	0,00725672	4,523140191	0,003331	0,998178681
7852	ENSG00000197555	0,492478325	3,904021679	5,360997	0,029814725
7853	ENSG00000197557	-0,064611569	3,820979446	0,1121957	0,740679027
7854	ENSG00000197563	-0,163621992	5,6471471	0,8005507	0,380152136
7855	ENSG00000197565	-0,318509318	4,864509936	1,865014	0,185191162
7856	ENSG00000197579	0,041156083	4,894456923	0,0576854	0,812309751
7857	ENSG00000197580	-0,276600181	6,240663539	1,4843587	0,235384568
7858	ENSG00000197586	-0,094371041	5,842855916	0,3073304	0,584647868
7859	ENSG00000197594	0,139629561	4,121213015	0,6458725	0,429780587
7860	ENSG00000197601	-0,140977662	6,424483239	0,5324847	0,472895394
7861	ENSG00000197603	0,052418081	6,275070935	0,1150801	0,737496062
7862	ENSG00000197608	0,169321129	4,373911257	1,5261733	0,233472742
7863	ENSG00000197614	-1,543801592	6,461768493	13,095515	0,001433809
7864	ENSG00000197616	-0,856923093	13,41545519	3,0129802	0,095915151
7865	ENSG00000197619	-0,188143731	4,552037257	1,1066173	0,30369295
7866	ENSG00000197620	-0,154178924	4,019650529	1,63737	0,358545522
7867	ENSG00000197622	0,122499884	5,68680121	0,4998557	0,486631142
7868	ENSG00000197629	0,078431648	5,067160695	0,1031325	0,750988335
7869	ENSG00000197694	0,342161312	8,668203897	2,5275301	0,125461788
7870	ENSG00000197702	0,180951133	6,764843117	0,9887925	0,330334743
7871	ENSG00000197712	0,398371907	5,434310116	8,5378519	0,007628272
7872	ENSG00000197713	-0,341765229	5,168120842	5,1084214	0,033534885
7873	ENSG00000197714	0,183974682	5,752307086	1,4583008	0,239413331
7874	ENSG00000197724	0,143393025	4,883859847	0,4939739	0,489179195
7875	ENSG00000197728	-0,238901856	6,391266604	0,642932	0,430818258
7876	ENSG00000197746	0,119352099	9,859520463	0,5017027	0,485811162
7877	ENSG00000197747	0,119702677	5,98957571	0,1726916	0,681563552
7878	ENSG00000197756	-0,044177449	8,794389553	0,0408222	0,841649402
7879	ENSG00000197763	0,090808024	3,519624012	0,6091396	0,625835978
7880	ENSG00000197766	-0,411134257	7,016412258	1,1944513	0,285694341
7881	ENSG00000197771	-0,001124792	5,546456027	0,0001031	0,991986851
7882	ENSG00000197776	0,141749932	4,064468334	1,0090769	0,350858045
7883	ENSG00000197779	0,112748815	4,270697981	0,6794532	0,446033697
7884	ENSG00000197780	-0,282471913	4,571283912	2,1277624	0,158108636
7885	ENSG00000197782	0,068334105	4,783969858	0,2214332	0,64235586
7886	ENSG00000197798	-0,207050409	4,162549913	4,2963257	0,204708216
7887	ENSG00000197818	-0,157982713	4,127409191	0,890773	0,355030054
7888	ENSG00000197841	-0,238407365	5,200411023	1,5317452	0,228276639
7889	ENSG00000197852	-0,174186385	4,791729659	0,5381817	0,470563784
7890	ENSG00000197857	-0,119156712	4,646980689	0,8019523	0,379710452
7891	ENSG00000197860	0,26817037	4,012823964	2,5407014	0,124530264
7892	ENSG00000197863	-0,074251064	3,961300197	0,3444821	0,689225286
7893	ENSG00000197872	-0,061715526	4,101556955	0,138865	0,739168846
7894	ENSG00000197879	0,2678221	6,718678387	1,3605732	0,255333189
7895	ENSG00000197885	-0,307011176	4,139573999	5,7049069	0,062885025
7896	ENSG00000197892	0,286145901	4,922760516	4,22655	0,051231255
7897	ENSG00000197893	0,314580386	9,384009827	0,9555315	0,338436534
7898	ENSG00000197894	-0,067526463	6,950497016	0,1022566	0,752010896
7899	ENSG00000197912	-0,001903124	6,128086787	0,0001058	0,991882732
7900	ENSG00000197915	0,056654271	4,239515681	0,0190315	0,891471121
7901	ENSG00000197928	0,098804225	3,956496803	0,8143302	0,569981543
7902	ENSG00000197930	0,034037295	4,959128909	0,0694981	0,794396989
7903	ENSG00000197937	-0,040100928	4,541063612	0,1039338	0,782434341
7904	ENSG00000197943	-0,249644561	4,480457288	2,4838454	0,12861074

7905	ENSG00000197956	0,073243441	7,701314974	0,0783492	0,782036964
7906	ENSG00000197958	0,331099378	8,042074555	2,2147492	0,150215171
7907	ENSG00000197959	-0,198335532	3,704747599	1,434024	0,257527126
7908	ENSG00000197961	-0,021092253	5,514524134	0,0345982	0,854057365
7909	ENSG00000197965	-0,216496644	6,26729883	0,8532843	0,365168689
7910	ENSG00000197969	-0,187798026	7,827312995	1,8592013	0,185806261
7911	ENSG00000197971	0,212787152	5,386022898	1,6874405	0,206748816
7912	ENSG00000197978	0,110682556	3,640525229	0,3737431	0,600791562
7913	ENSG00000197982	-0,309428671	4,534529555	2,8614467	0,104167125
7914	ENSG00000198000	0,000718752	5,003422559	4,028E-05	0,994990709
7915	ENSG00000198001	-0,107890461	4,27015104	0,6836271	0,507083875
7916	ENSG00000198015	-0,198401165	5,841932125	1,5109545	0,231361215
7917	ENSG00000198018	0,204975234	4,405123615	0,603834	0,444999784
7918	ENSG00000198034	0,179392578	8,801225234	0,8506017	0,365883205
7919	ENSG00000198040	0,066829543	5,59514745	0,2652883	0,611384254
7920	ENSG00000198042	-0,083737906	4,444307103	0,1910785	0,666077561
7921	ENSG00000198046	0,211504985	3,545119025	1,9199155	0,298414644
7922	ENSG00000198053	0,124331202	5,26439925	0,3364079	0,567521833
7923	ENSG00000198060	-0,159183395	4,794670144	1,8387798	0,243063975
7924	ENSG00000198064	-0,061283456	5,390233276	0,0554247	0,815952689
7925	ENSG00000198087	0,019613347	5,323398736	0,0114817	0,91559264
7926	ENSG00000198093	-0,108430085	3,644667893	0,7161554	0,574573002
7927	ENSG00000198105	-0,179346023	5,056461953	1,6805712	0,207612656
7928	ENSG00000198121	-0,13691851	5,379481821	0,271851	0,607053203
7929	ENSG00000198125	-0,300846516	11,92216958	1,5387595	0,227204744
7930	ENSG00000198130	-0,134550639	5,388370139	1,7007423	0,215256606
7931	ENSG00000198131	0,017388856	4,903456846	0,0129553	0,910362439
7932	ENSG00000198146	-0,114952633	5,728509853	0,4465471	0,510598115
7933	ENSG00000198160	0,047686629	5,876921173	0,0958434	0,759649831
7934	ENSG00000198162	0,123871699	6,753586448	0,5206296	0,477809644
7935	ENSG00000198168	-0,208851655	5,020681832	0,8386118	0,369250338
7936	ENSG00000198169	0,166181908	3,989976245	1,2562043	0,369044515
7937	ENSG00000198171	-0,063493771	4,205146332	0,1315032	0,720173598
7938	ENSG00000198176	-0,051014727	5,200682575	0,1267994	0,724997004
7939	ENSG00000198189	0,038280337	5,047730442	0,0235634	0,879333355
7940	ENSG00000198198	-0,082445682	5,2900059	0,0932093	0,762868726
7941	ENSG00000198218	0,046240358	5,457363846	0,1255994	0,726240921
7942	ENSG00000198225	-0,085175066	3,965362793	0,4121222	0,615115203
7943	ENSG00000198231	0,24218777	6,4648511	5,0664074	0,034167076
7944	ENSG00000198237	0,408791146	3,933624089	4,6914024	0,040872258
7945	ENSG00000198242	0,126893838	7,376141402	0,2780431	0,603011997
7946	ENSG00000198252	0,11230868	5,031246007	0,4924979	0,489822213
7947	ENSG00000198258	-0,251105431	6,326783673	2,2769301	0,144861249
7948	ENSG00000198265	0,139437475	6,787224831	1,7127922	0,203443371
7949	ENSG00000198270	-0,545285015	4,011968803	6,0104753	0,022194443
7950	ENSG00000198300	0,217094286	4,100648691	0,8439323	0,36776267
7951	ENSG00000198301	0,015090309	5,146373503	0,018266	0,893657874
7952	ENSG00000198315	0,1103655	5,474602712	1,2365894	0,277523261
7953	ENSG00000198336	-1,187554388	10,77642741	10,065457	0,004230463
7954	ENSG00000198356	-0,092118287	4,656263172	0,4682368	0,500580099
7955	ENSG00000198363	0,024334089	7,896541224	0,0132072	0,909498916
7956	ENSG00000198369	0,25263875	4,304109214	2,1714578	0,154082893
7957	ENSG00000198373	0,38228297	4,300886645	4,7904326	0,038979171
7958	ENSG00000198380	0,396917235	6,683162559	7,1294162	0,013627151
7959	ENSG00000198382	0,183039484	4,962589988	1,7002079	0,205072157
7960	ENSG00000198393	-0,053639748	4,38028675	0,1025946	0,751615726
7961	ENSG00000198399	0,05608513	6,366771508	0,2731776	0,606165219
7962	ENSG00000198406	0,184315423	4,167198121	0,7593018	0,392508628
7963	ENSG00000198408	0,245691431	7,680584677	3,5609419	0,071732734

7964	ENSG00000198416	-0,311824446	4,442090695	1,9987676	0,17075567
7965	ENSG00000198420	0,076061083	5,777322485	0,1970045	0,661277256
7966	ENSG00000198431	0,027448908	7,301311715	0,017381	0,896254165
7967	ENSG00000198453	0,164085631	4,014639989	2,4743056	0,303713224
7968	ENSG00000198455	0,299003939	3,879141047	5,3216539	0,09133888
7969	ENSG00000198464	-0,305606133	4,98263356	2,8851215	0,102825023
7970	ENSG00000198466	0,087157251	4,43366407	0,1817644	0,673805817
7971	ENSG00000198467	-0,375407731	8,2390523	2,2553357	0,146694311
7972	ENSG00000198478	0,158966641	4,784031199	0,6707033	0,421170996
7973	ENSG00000198482	0,009009884	4,99943668	0,0036093	0,952610233
7974	ENSG00000198492	0,002634107	5,417902121	0,000778	0,977986698
7975	ENSG00000198498	-0,106640759	3,730530013	0,5350295	0,608953044
7976	ENSG00000198502	0,721392749	5,037142669	7,2420438	0,013006979
7977	ENSG00000198521	-0,09657389	4,699814396	0,5397673	0,494261512
7978	ENSG00000198522	-0,154072572	4,290752476	2,2586899	0,339934099
7979	ENSG00000198523	-0,59238863	10,89508723	1,1082717	0,303340055
7980	ENSG00000198538	0,128842191	3,939093393	1,3162829	0,423566335
7981	ENSG00000198551	0,215828401	3,84471907	1,7557774	0,211430692
7982	ENSG00000198561	0,192632344	7,072467685	1,3912388	0,250194964
7983	ENSG00000198563	-0,180322092	5,492521671	0,7347219	0,400157617
7984	ENSG00000198585	-0,070936074	5,815132462	0,1861115	0,670170139
7985	ENSG00000198586	0,107114851	5,556597722	0,8248133	0,373116957
7986	ENSG00000198589	-0,050288204	6,868596928	0,1711346	0,682905602
7987	ENSG00000198604	0,046033793	4,933099339	0,0437081	0,836229287
7988	ENSG00000198612	-0,268753533	6,061500468	1,72443	0,202012269
7989	ENSG00000198618	-0,125397458	5,530869407	0,4781037	0,496169799
7990	ENSG00000198624	-0,4243451	7,352363618	6,6551158	0,01671378
7991	ENSG00000198625	0,007235436	6,402556583	0,0017668	0,966833185
7992	ENSG00000198626	-0,323175488	11,56537484	0,3625127	0,552975319
7993	ENSG00000198642	0,067677634	5,408894393	0,2246014	0,639994053
7994	ENSG00000198646	-0,073559191	4,777841642	0,1220629	0,729970878
7995	ENSG00000198648	-0,127627324	4,946596788	1,3013884	0,301284225
7996	ENSG00000198663	-0,200562333	6,838797356	2,706787	0,113408267
7997	ENSG00000198668	-0,217409983	8,923918819	1,6238825	0,215171608
7998	ENSG00000198677	-0,22774436	7,370631439	0,6846052	0,416466803
7999	ENSG00000198682	0,709319489	4,797515971	5,0085668	0,035146751
8000	ENSG00000198689	-0,209184124	4,891704709	4,1611322	0,07452722
8001	ENSG00000198690	-0,154512245	5,139905247	1,0931223	0,306592297
8002	ENSG00000198692	-0,313502892	6,333420703	0,2031176	0,656415642
8003	ENSG00000198695	0,063230959	11,13319072	0,0891545	0,767913591
8004	ENSG00000198700	-0,007937577	5,711317805	0,0044718	0,947257129
8005	ENSG00000198707	0,151562212	5,314207453	0,7336642	0,400491738
8006	ENSG00000198712	-0,227134647	13,0560767	0,7857252	0,384494679
8007	ENSG00000198718	-0,064547803	5,707289182	0,2090524	0,651770729
8008	ENSG00000198721	-0,355169337	5,932728713	3,2873547	0,082823973
8009	ENSG00000198722	0,069900407	6,138661894	0,3395556	0,565708547
8010	ENSG00000198727	-0,152911698	12,87312221	0,2816267	0,600681974
8011	ENSG00000198728	0,560655433	5,015802099	4,6550928	0,041592094
8012	ENSG00000198729	-0,41029089	6,171352958	3,6025294	0,070250363
8013	ENSG00000198730	0,171473166	5,469397583	2,6185802	0,119138887
8014	ENSG00000198734	-0,716028733	5,211480184	2,3844643	0,136129611
8015	ENSG00000198739	-0,361394761	4,532700126	1,3775877	0,252465657
8016	ENSG00000198740	0,154793057	5,236149553	1,0073933	0,325921019
8017	ENSG00000198742	0,097679648	4,409777555	0,3358729	0,56782781
8018	ENSG00000198743	-0,301095765	6,147917538	0,618429	0,439620627
8019	ENSG00000198744	-0,171561884	9,396958603	0,6487835	0,428728704
8020	ENSG00000198752	0,275406082	5,946052431	1,3671695	0,254216509
8021	ENSG00000198755	0,276912624	7,999940658	1,2068503	0,283269267
8022	ENSG00000198756	0,6063831	4,072177021	4,8428216	0,038017674

8023	ENSG00000198763	0,063112682	12,66781638	0,0647498	0,801382842
8024	ENSG00000198771	-0,222736696	5,458211358	2,813534	0,10689933
8025	ENSG00000198780	-0,245025542	3,649010905	0,8267997	0,372584405
8026	ENSG00000198786	0,283577952	12,30857654	0,4062437	0,530145949
8027	ENSG00000198791	-0,0885525	6,444353715	0,2820167	0,600448989
8028	ENSG00000198792	-0,000473963	4,87981504	7,874E-06	0,997785194
8029	ENSG00000198793	-0,038369072	6,115903996	0,0522001	0,821287542
8030	ENSG00000198795	0,013666823	5,301065012	0,0193028	0,999817701
8031	ENSG00000198796	-0,646729452	8,10132583	3,4665745	0,075384591
8032	ENSG00000198799	0,027352092	5,219387286	0,037135	0,848870732
8033	ENSG00000198804	-0,166186814	15,32083584	0,3765491	0,545427616
8034	ENSG00000198812	-0,942935605	5,282632504	9,5395913	0,005166222
8035	ENSG00000198814	-0,08943058	4,180779052	0,1483466	0,703642109
8036	ENSG00000198815	0,092901925	5,877459834	0,7034148	0,410199449
8037	ENSG00000198818	-0,050048332	4,187194848	0,2148806	0,779750767
8038	ENSG00000198824	0,156386068	3,600891803	0,547381	0,46683884
8039	ENSG00000198825	0,366769938	4,174948416	6,6890243	0,017356122
8040	ENSG00000198830	0,583863145	6,645153138	11,99619	0,002096204
8041	ENSG00000198832	0,058078739	4,939100468	0,0989933	0,755863778
8042	ENSG00000198833	-0,167535466	5,531093794	0,9687212	0,335190989
8043	ENSG00000198836	-0,241039271	7,443805885	1,0604526	0,313768508
8044	ENSG00000198837	-0,146264323	4,706882657	0,3907206	0,53804387
8045	ENSG00000198839	0,051940958	4,700332469	0,0802304	0,779506986
8046	ENSG00000198840	-0,414735904	11,26947753	1,9007749	0,18116323
8047	ENSG00000198842	-0,107237724	7,134239735	0,1243164	0,727594334
8048	ENSG00000198843	-0,253336668	6,368116031	1,3572764	0,255893668
8049	ENSG00000198848	0,381069959	5,76541556	1,3810208	0,251892111
8050	ENSG00000198853	-0,089215974	5,08313577	0,1803938	0,674962792
8051	ENSG00000198856	0,060905729	5,062130492	0,0557852	0,815366622
8052	ENSG00000198860	-0,303986409	4,6042545	3,6267436	0,069378516
8053	ENSG00000198862	-0,306068611	6,534794443	1,6137631	0,216603114
8054	ENSG00000198863	-0,023805114	3,811878602	0,0274653	0,869809621
8055	ENSG00000198873	0,041321036	5,256657866	0,0351656	0,852885641
8056	ENSG00000198874	-0,246047417	4,590576053	4,4452471	0,053357782
8057	ENSG00000198876	0,468389756	4,765141558	4,6532898	0,041628207
8058	ENSG00000198879	0,137368123	4,907710035	1,0544737	0,315069776
8059	ENSG00000198886	0,034877057	13,55370708	0,0178211	0,894953018
8060	ENSG00000198887	0,033932767	6,054506093	0,0412452	0,840842642
8061	ENSG00000198888	-0,080995205	12,41296355	0,0810731	0,778375267
8062	ENSG00000198892	-0,514382199	4,499898795	5,0101783	0,035120058
8063	ENSG00000198894	-0,18348704	6,003210843	1,040604	0,318240594
8064	ENSG00000198898	-0,508856947	8,040086102	2,3239525	0,14096342
8065	ENSG00000198899	-0,076072251	12,87623577	0,1099512	0,743177227
8066	ENSG00000198900	0,009896419	5,454153127	0,0043596	0,947924212
8067	ENSG00000198909	0,308220958	5,42668908	2,902896	0,101830625
8068	ENSG00000198911	0,392501331	5,955613447	2,4643758	0,13004422
8069	ENSG00000198918	0,201028413	8,096506445	0,9240912	0,346352755
8070	ENSG00000198919	-0,10772247	5,865512345	0,3320175	0,570042589
8071	ENSG00000198920	0,340753088	3,727020211	5,2735363	0,071687281
8072	ENSG00000198924	-0,135468451	4,010932512	0,7038826	0,447511307
8073	ENSG00000198925	-0,083873609	4,526143561	0,1651086	0,688233862
8074	ENSG00000198932	0,438911839	5,810836193	7,0505792	0,014105426
8075	ENSG00000198938	-0,201030073	13,64581542	0,7713574	0,388805261
8076	ENSG00000198947	-0,20432548	9,59989218	0,6240038	0,437593178
8077	ENSG00000198951	-0,259598656	4,266450773	2,1518178	0,155876928
8078	ENSG00000198952	-0,018033948	6,262337092	0,0084248	0,927658504
8079	ENSG00000198954	-0,132726107	5,275186119	0,952806	0,339081262
8080	ENSG00000198959	-0,047245923	8,564924146	0,0412105	0,840908608
8081	ENSG00000198960	0,173705923	4,21768339	1,3312844	0,26895195

8082	ENSG00000198961	-0,120853088	7,904709329	0,3544502	0,557389714
8083	ENSG00000198964	0,024021224	5,339528613	0,0302701	0,863389679
8084	ENSG00000199415	0,610654314	5,339241168	1,2572348	0,273690127
8085	ENSG00000199480	0,613167012	5,346319415	1,2927619	0,267190498
8086	ENSG00000199523	0,632254537	5,366571142	1,3634948	0,254837818
8087	ENSG00000199568	1,382870751	6,957849771	3,6809773	0,067470273
8088	ENSG00000199677	0,622500081	3,448134474	2,5046156	0,127102047
8089	ENSG00000199994	0,424618288	4,631684406	0,7399571	0,398509875
8090	ENSG00000200087	0,634243509	7,464444254	2,560237	0,123163618
8091	ENSG00000200156	0,589262885	5,649855545	1,3934667	0,249826886
8092	ENSG00000200312	0,969200471	8,755105205	2,0517153	0,165421671
8093	ENSG00000200314	0,765493531	6,711392028	2,3784536	0,136600845
8094	ENSG00000200488	0,812473586	10,39141546	1,890689	0,182307863
8095	ENSG00000200494	0,872978858	9,358473198	2,6167178	0,119310628
8096	ENSG00000200558	0,087700173	3,861243444	0,0439242	0,83583104
8097	ENSG00000200795	-0,080527448	6,175118506	0,0302613	0,863413257
8098	ENSG00000200959	1,134846218	5,25972417	3,5451964	0,072365079
8099	ENSG00000201098	0,910949263	8,364221835	2,2383309	0,148157314
8100	ENSG00000201185	0,696323314	5,776488178	2,0807328	0,162583998
8101	ENSG00000201428	0,478290002	10,75839423	0,8690795	0,360847016
8102	ENSG00000201654	0,423837338	3,646831305	1,7187222	0,202734293
8103	ENSG00000201955	0,878773766	9,32634905	2,7023987	0,113732674
8104	ENSG00000201998	0,6882391	5,0165848	2,033899	0,167193661
8105	ENSG00000202058	0,269146769	10,1100878	0,4906528	0,490628026
8106	ENSG00000202354	0,866729351	9,406009329	2,5416647	0,124462458
8107	ENSG00000202512	0,283316574	5,39335216	0,6196417	0,439178327
8108	ENSG00000202538	0,447940548	9,573759418	0,6073584	0,44369129
8109	ENSG00000203326	0,202335932	3,975238779	0,9344711	0,34371074
8110	ENSG00000203485	0,35105141	4,722849141	2,5146072	0,126383741
8111	ENSG00000203506	-0,015713253	3,55939303	0,0067876	0,938800279
8112	ENSG00000203666	-0,366844719	4,428624707	2,2505508	0,147104221
8113	ENSG00000203668	-0,108505492	4,489469004	0,3167846	0,578965654
8114	ENSG00000203705	-0,197507916	4,497252631	0,8417013	0,368385436
8115	ENSG00000203709	-0,227306978	6,418695856	0,527179	0,475084271
8116	ENSG00000203710	-1,586686306	4,723649134	15,300933	0,000695409
8117	ENSG00000203747	-0,193046934	4,919690161	0,3739203	0,546843156
8118	ENSG00000203778	-0,331953893	4,912183951	3,2043802	0,086551993
8119	ENSG00000203791	0,034123912	3,768313176	0,0583316	0,838662147
8120	ENSG00000203797	-0,383593213	3,928399534	2,7251077	0,11230593
8121	ENSG00000203867	0,138926226	7,006845632	0,1739828	0,680445009
8122	ENSG00000203875	0,095004317	5,626691883	0,2938215	0,592970056
8123	ENSG00000203879	-0,020360312	5,894616705	0,0149646	0,903695184
8124	ENSG00000203880	0,069872896	6,474258335	0,2686384	0,60915608
8125	ENSG00000203930	0,226805953	6,123592823	0,1413077	0,710412657
8126	ENSG00000203965	0,075273477	4,148881265	0,1207423	0,731375173
8127	ENSG00000204054	0,25266085	4,572261835	1,38217	0,2517005
8128	ENSG00000204070	0,07940411	4,571674398	0,2951634	0,635887298
8129	ENSG00000204084	-0,006479204	4,640201405	0,0016048	0,968389684
8130	ENSG00000204103	-0,230878044	4,262394443	0,6956667	0,412781205
8131	ENSG00000204104	0,300221172	3,800617562	6,1150713	0,096147627
8132	ENSG00000204116	-0,256048688	5,306641687	1,4603915	0,239086863
8133	ENSG00000204120	0,051744446	6,527311009	0,3350718	0,568267166
8134	ENSG00000204130	0,102248803	4,847680059	0,4943233	0,489023192
8135	ENSG00000204131	0,140321749	4,590706166	0,3787912	0,544264137
8136	ENSG00000204136	0,461855118	4,427879455	4,1671093	0,052793368
8137	ENSG00000204138	0,069404861	4,61993653	0,2061212	0,654059321
8138	ENSG00000204147	-0,252875665	3,788358579	1,0890311	0,307478687
8139	ENSG00000204161	-0,545133439	4,312779835	14,752006	0,000825588
8140	ENSG00000204177	-0,062963093	4,58457929	0,1569608	0,695604048

8141	ENSG00000204178	0,058756194	4,716281623	0,2763863	0,626815177
8142	ENSG00000204179	-0,132115712	4,301422693	0,1960918	0,662010869
8143	ENSG00000204186	0,224849191	5,491581114	1,8390545	0,188163463
8144	ENSG00000204209	0,258805105	3,981885749	1,8501096	0,186890575
8145	ENSG00000204217	0,107277172	7,53024341	0,6289277	0,435786804
8146	ENSG00000204219	-0,087107736	5,182439197	0,27656	0,603974591
8147	ENSG00000204231	0,323234615	4,751172348	4,1450774	0,05337426
8148	ENSG00000204253	-0,008177288	4,504311392	0,0027914	0,958318458
8149	ENSG00000204256	0,134938541	6,388701794	0,7647444	0,390844477
8150	ENSG00000204257	0,405101015	4,508081685	3,2466933	0,084627247
8151	ENSG00000204262	0,512480444	6,068019198	3,3526864	0,080018398
8152	ENSG00000204264	0,171609406	4,140551172	0,4355606	0,515796454
8153	ENSG00000204271	0,045651955	3,757573932	0,1219649	0,811614426
8154	ENSG00000204287	0,756514741	7,548839536	5,9718446	0,022581307
8155	ENSG00000204291	0,58840184	6,82660992	3,5611356	0,071769944
8156	ENSG00000204301	0,226513322	4,829096856	0,8404081	0,368747096
8157	ENSG00000204304	0,18581479	4,789973188	1,0567479	0,314596653
8158	ENSG00000204308	-0,408937516	4,916029567	8,8798451	0,006660364
8159	ENSG00000204351	-0,004564503	4,463689355	0,0006009	0,9806532
8160	ENSG00000204356	0,062632656	4,8390733	0,2073129	0,685242149
8161	ENSG00000204370	-0,422607349	6,807373589	2,4235346	0,13311312
8162	ENSG00000204386	0,08677754	4,522687174	0,5119375	0,611652974
8163	ENSG00000204388	-0,085682048	6,228218621	0,0612121	0,806774927
8164	ENSG00000204389	-0,119821713	7,512694681	0,0981	0,75693062
8165	ENSG00000204394	0,025382588	4,201070472	0,0134185	0,908781416
8166	ENSG00000204406	0,01065925	5,535121142	0,0095868	0,922843072
8167	ENSG00000204439	-0,019978385	3,779487942	0,0110471	0,917199211
8168	ENSG00000204463	0,030063685	6,332382563	0,0227852	0,881326628
8169	ENSG00000204469	0,167980398	6,174631597	0,3909635	0,537918581
8170	ENSG00000204472	0,124957262	4,176298741	0,2577412	0,616486862
8171	ENSG00000204514	0,025249137	4,662473568	0,0158388	0,900937128
8172	ENSG00000204519	0,092869077	3,727263633	0,4144677	0,64381706
8173	ENSG00000204524	0,203238675	4,91316953	3,1541916	0,088854769
8174	ENSG00000204525	0,328890647	7,28731107	3,0191849	0,095593795
8175	ENSG00000204560	0,153216946	4,47320273	0,998195	0,353047555
8176	ENSG00000204568	-0,334443314	5,731550672	6,8156482	0,015570611
8177	ENSG00000204569	0,51244276	4,864708811	4,6527524	0,041638978
8178	ENSG00000204574	-0,019754443	5,328930123	0,0161567	0,899953633
8179	ENSG00000204580	0,634059072	4,978692339	9,3450007	0,00556833
8180	ENSG00000204590	0,26626275	4,772177022	2,5156797	0,126306922
8181	ENSG00000204592	0,428171817	8,124190508	7,0414482	0,01413625
8182	ENSG00000204604	0,037943112	3,568880156	0,0970819	0,828504777
8183	ENSG00000204611	0,275740318	3,592125652	3,7398846	0,142560407
8184	ENSG00000204619	-0,296880974	4,736850522	4,5156496	0,044456802
8185	ENSG00000204623	0,045132207	3,927899001	0,0452031	0,8334949
8186	ENSG00000204628	0,131594892	8,551556965	0,321272	0,576307889
8187	ENSG00000204634	0,288632988	5,778504994	1,8993656	0,18134593
8188	ENSG00000204642	0,379781231	4,305106212	5,8763789	0,023569932
8189	ENSG00000204650	-0,235703174	3,72204687	1,268444	0,271617252
8190	ENSG00000204652	-0,092362721	4,694201128	0,0816252	0,777651877
8191	ENSG00000204681	0,213784711	4,85465051	0,9284168	0,345248279
8192	ENSG00000204713	0,283661287	4,915290229	6,0096156	0,02244406
8193	ENSG00000204745	0,048143415	4,091753087	0,1951931	0,783382224
8194	ENSG00000204764	0,46486591	3,909350702	4,4152617	0,046721373
8195	ENSG00000204778	-0,323124121	4,270465018	1,9902446	0,171633661
8196	ENSG00000204789	-0,295457755	4,322159556	1,548581	0,225816891
8197	ENSG00000204842	0,078448948	5,489239768	0,2137494	0,648167187
8198	ENSG00000204843	0,163867203	6,401973718	0,8081792	0,377929835
8199	ENSG00000204852	0,043759449	4,655030635	0,1402954	0,72446039

8200	ENSG00000204899	-0,040785486	3,817839707	0,0296145	0,86486581
8201	ENSG00000204977	-0,141377686	5,362361046	1,1367101	0,297321513
8202	ENSG00000205038	2,720434004	7,833211303	4,7953025	0,038888651
8203	ENSG00000205060	0,072533459	4,020749387	0,2094127	0,651500933
8204	ENSG00000205133	0,161828081	5,281045173	0,4141964	0,526182141
8205	ENSG00000205189	0,007019581	6,146825526	0,0017666	0,966834665
8206	ENSG00000205213	-0,106228374	5,94562469	0,4980584	0,487407372
8207	ENSG00000205221	-1,212992872	4,188207333	10,844821	0,003168109
8208	ENSG00000205250	-0,122682379	4,649793522	0,6660581	0,42274942
8209	ENSG00000205268	-0,482821859	6,630393503	2,6416352	0,117655995
8210	ENSG00000205269	0,09168248	4,744933194	0,1923328	0,665054087
8211	ENSG00000205302	-0,075574512	5,868159394	0,1678298	0,685819882
8212	ENSG00000205339	0,045763954	7,583379711	0,0691429	0,794918703
8213	ENSG00000205352	-0,125653828	4,217484838	0,5276322	0,474896649
8214	ENSG00000205363	-0,949847841	4,724812855	9,1703069	0,005958761
8215	ENSG00000205413	0,366816669	4,964674122	1,6652089	0,209662176
8216	ENSG00000205423	-0,223910517	4,014736686	2,2835283	0,196902476
8217	ENSG00000205476	-0,257640136	3,904569834	1,5580687	0,22444548
8218	ENSG00000205531	-0,009983042	6,708982527	0,0083677	0,927900422
8219	ENSG00000205534	-0,195004704	6,485460829	0,948082	0,340289068
8220	ENSG00000205542	0,528370054	9,229532722	3,1194764	0,090568557
8221	ENSG00000205571	0,252703786	4,855391698	1,8681821	0,184832373
8222	ENSG00000205572	0,275153098	4,055248212	3,2294876	0,114168993
8223	ENSG00000205581	0,358232296	6,07338677	6,9805393	0,014508192
8224	ENSG00000205583	0,025409612	4,083888957	0,0115772	0,91524361
8225	ENSG00000205609	0,207407285	7,562359343	1,1420822	0,296247677
8226	ENSG00000205629	0,024442075	4,296695031	0,0292016	0,947499022
8227	ENSG00000205659	-0,037977955	3,706226486	0,0925266	0,84463184
8228	ENSG00000205670	-0,200435963	4,325376683	2,6879344	0,176625299
8229	ENSG00000205678	-0,700243306	9,889195927	2,4755951	0,129215888
8230	ENSG00000205683	-0,242610978	4,577165092	0,6060407	0,444179785
8231	ENSG00000205707	-0,558556236	5,184105527	3,7028097	0,066719058
8232	ENSG00000205726	0,088766786	6,50895541	0,1978541	0,660596206
8233	ENSG00000205730	0,45711668	5,245357563	5,8526736	0,023822825
8234	ENSG00000205758	-0,171670884	4,695086448	1,9474812	0,232181919
8235	ENSG00000205765	-0,074565135	5,147280501	0,2205214	0,643043084
8236	ENSG00000205871	0,374887593	4,984810582	2,4080162	0,134301653
8237	ENSG00000205937	-0,040639903	5,517318622	0,0717841	0,791133232
8238	ENSG00000205978	0,597409951	4,071897553	5,513109	0,027798084
8239	ENSG00000205981	-0,509660816	5,371488255	5,9710517	0,022589325
8240	ENSG00000206052	-0,07447348	4,267914917	0,2630151	0,612914167
8241	ENSG00000206053	0,136690565	4,690916677	0,9284378	0,345214837
8242	ENSG00000206077	-0,282697292	4,049700211	1,1640471	0,291759027
8243	ENSG00000206127	0,18727566	5,161040364	1,0796506	0,309524183
8244	ENSG00000206149	-0,418268009	6,695623199	2,6695358	0,115835144
8245	ENSG00000206172	2,104189646	5,921295489	14,597692	0,00087132
8246	ENSG00000206337	0,346851995	4,020981082	3,350579	0,080107171
8247	ENSG00000206341	0,423554696	5,037286796	2,9668334	0,098344946
8248	ENSG00000206384	0,381363632	4,965645063	0,9265845	0,345715524
8249	ENSG00000206418	-0,281776674	5,946178395	2,8258833	0,106221742
8250	ENSG00000206503	0,430385229	7,573744771	7,1191326	0,013688852
8251	ENSG00000206527	0,492564894	5,587701426	1,9763203	0,173079909
8252	ENSG00000206530	0,173529688	3,608969735	1,2581371	0,384490695
8253	ENSG00000206538	0,614795412	5,563087572	1,7880334	0,194178142
8254	ENSG00000206560	0,02924502	5,473569754	0,0354627	0,852273117
8255	ENSG00000206561	0,878572993	4,246915208	6,9735325	0,01457609
8256	ENSG00000206573	-0,119145477	4,83570313	0,7166944	0,405894694
8257	ENSG00000206585	0,718550773	12,4766925	1,3953028	0,249524063
8258	ENSG00000206588	0,554738964	7,896571509	0,884328	0,356744161

8259	ENSG00000206596	0,628251668	9,812339614	1,172987	0,289958131
8260	ENSG00000206625	0,575964963	3,890616107	2,7900633	0,108339138
8261	ENSG00000206651	0,753989274	4,405610116	1,7956789	0,193261921
8262	ENSG00000206652	0,712408269	12,50201105	1,3740304	0,253061731
8263	ENSG00000206737	0,50234788	8,438606149	0,7431926	0,397496626
8264	ENSG00000206899	0,408526409	4,036216353	1,4475572	0,241099981
8265	ENSG00000206932	0,415302852	3,683940257	1,54807	0,225891067
8266	ENSG00000206965	0,529669755	3,910642244	2,3766716	0,136740927
8267	ENSG00000207005	0,650638657	9,910460984	1,2441823	0,276130154
8268	ENSG00000207008	0,290824787	5,579700465	0,5638516	0,460289888
8269	ENSG00000207041	0,480382308	3,971939254	2,1416779	0,156812993
8270	ENSG00000207142	0,868717626	9,407425013	2,5829896	0,121594126
8271	ENSG00000207357	0,436790949	3,993194821	1,642805	0,212650129
8272	ENSG00000207389	0,623804504	10,21455256	1,1739365	0,289767732
8273	ENSG00000207445	0,848634632	4,32970471	2,8658377	0,10391667
8274	ENSG00000207507	0,415169448	3,713111633	1,763903	0,1971053
8275	ENSG00000207513	0,760870736	12,05780372	1,4701649	0,237568242
8276	ENSG00000207524	0,494034846	3,844202401	2,181249	0,153197768
8277	ENSG00000208772	0,608389722	5,236961085	1,2984088	0,266176079
8278	ENSG00000208892	0,002415664	4,050020417	6,766E-05	0,993507432
8279	ENSG00000210082	0,48059829	13,83595048	1,3795785	0,25213286
8280	ENSG00000210127	0,133925272	4,500348431	0,0908806	0,765756281
8281	ENSG00000210135	0,12738753	7,687410743	0,1090962	0,744150696
8282	ENSG00000210140	-0,007823776	9,274053695	0,0004632	0,983013755
8283	ENSG00000210144	-0,286838949	6,056908916	0,607757	0,443543657
8284	ENSG00000210196	-0,50892453	8,20900013	1,4264985	0,244450356
8285	ENSG00000211445	-0,446686345	9,616564914	3,8323777	0,062428326
8286	ENSG00000211448	-0,161317485	4,007831568	0,2535036	0,619383838
8287	ENSG00000211455	0,223338438	7,523418361	0,435691	0,515734215
8288	ENSG00000211456	0,094533643	5,682690988	0,3437098	0,563377938
8289	ENSG00000211459	1,036057952	12,4300346	1,2735119	0,270686823
8290	ENSG00000211460	0,034134748	5,643025475	0,0901228	0,766695342
8291	ENSG00000211584	-0,225477229	4,277728213	1,1502558	0,294566634
8292	ENSG00000212232	1,471946569	9,12053437	4,1340678	0,053667251
8293	ENSG00000212402	2,979885327	6,337745809	5,1262541	0,033255832
8294	ENSG00000212443	1,032772313	7,899954237	2,5700537	0,122483548
8295	ENSG00000212464	0,462967678	4,71128022	1,2308279	0,278656366
8296	ENSG00000212664	0,027973791	6,208591557	0,0114222	0,915810572
8297	ENSG00000212719	-0,024687368	4,100391883	0,0196386	0,889765122
8298	ENSG00000212802	0,391050971	6,647230769	3,7530946	0,065024853
8299	ENSG00000212907	0,019910133	10,04651843	0,0023153	0,962036113
8300	ENSG00000212994	-0,166157104	4,763131239	0,2607495	0,614448549
8301	ENSG00000213020	0,048932212	3,947051521	0,2336001	0,788300083
8302	ENSG00000213024	-0,004908062	4,042091778	0,0009344	0,975876796
8303	ENSG00000213047	0,032087565	5,885197746	0,0194393	0,890322197
8304	ENSG00000213064	0,181150907	5,97529465	0,9633343	0,336511248
8305	ENSG00000213066	0,151473579	3,59546902	1,5194471	0,44000237
8306	ENSG00000213079	0,052957996	6,238199387	0,1812083	0,674260915
8307	ENSG00000213088	-0,995439828	4,926264928	6,5570802	0,01744114
8308	ENSG00000213096	-0,086288638	5,417866356	0,2449312	0,625339003
8309	ENSG00000213160	-0,580581287	4,704649074	7,9066305	0,009868218
8310	ENSG00000213178	0,132239832	5,934455287	0,4545761	0,506857759
8311	ENSG00000213190	-0,277668279	4,026224449	0,769071	0,389529039
8312	ENSG00000213281	0,000466258	4,910167409	9,39E-06	0,997581269
8313	ENSG00000213300	-0,022031754	3,831666257	0,0185122	0,892952314
8314	ENSG00000213326	-0,015790066	4,650231432	0,0052085	0,94308739
8315	ENSG00000213341	-0,135845774	4,868465078	0,85304	0,365236116
8316	ENSG00000213366	-0,208479928	4,443977317	1,0320033	0,320205552
8317	ENSG00000213430	-0,160523332	3,995532329	1,2886448	0,372415647

8318	ENSG00000213442	0,290106722	5,660194557	1,7294933	0,201374438
8319	ENSG00000213462	0,087680455	4,083770733	0,098287	0,756706935
8320	ENSG00000213463	-0,087379394	6,224422971	0,4434525	0,512030004
8321	ENSG00000213516	0,011235733	4,782170294	0,0116488	0,914979927
8322	ENSG00000213523	0,108671376	4,539369445	0,8113973	0,461935993
8323	ENSG00000213553	0,049986814	6,584246961	0,0366556	0,849839895
8324	ENSG00000213585	-0,352940651	8,236192727	2,8690523	0,103733756
8325	ENSG00000213593	-0,407883814	5,690136318	14,046772	0,001038427
8326	ENSG00000213614	-0,028999373	4,755098583	0,0616425	0,806103518
8327	ENSG00000213619	-0,425934854	5,864691418	6,6720791	0,016592148
8328	ENSG00000213621	-0,004296227	4,671999946	0,0004252	0,983726139
8329	ENSG00000213625	-0,002413745	6,457624691	0,0003432	0,985378862
8330	ENSG00000213626	1,066339184	5,311741623	21,005577	0,000130133
8331	ENSG00000213639	-0,044849649	8,591990755	0,0450113	0,833843101
8332	ENSG00000213672	0,7965399	4,350270238	6,2100626	0,020309547
8333	ENSG00000213676	0,293017274	4,476073363	2,4885056	0,1282704
8334	ENSG00000213694	-1,282282363	4,825867989	29,343457	1,64155E-05
8335	ENSG00000213699	-0,07360054	4,758458199	0,1362114	0,715436274
8336	ENSG00000213707	-0,013637492	4,042654097	0,0036234	0,952517873
8337	ENSG00000213719	-0,010705462	6,369476835	0,0029394	0,957228609
8338	ENSG00000213722	0,380833691	4,835712584	3,6460891	0,068690851
8339	ENSG00000213741	0,239081303	7,237477761	0,9254174	0,346013604
8340	ENSG00000213753	0,017278663	3,909447375	0,0173922	0,979960215
8341	ENSG00000213757	0,226449949	4,006664416	0,7324034	0,400890614
8342	ENSG00000213762	0,039852617	4,66263389	0,1998411	0,788438169
8343	ENSG00000213799	0,079004347	4,196780254	0,2276325	0,637765265
8344	ENSG00000213853	-0,157453128	6,09762403	0,78255	0,385472721
8345	ENSG00000213860	-0,033907878	7,227724307	0,0221167	0,883067045
8346	ENSG00000213866	-0,106872913	4,083689155	0,6969011	0,503917283
8347	ENSG00000213885	0,216633863	5,081783782	0,9773316	0,333095606
8348	ENSG00000213923	0,081875509	4,534456986	0,2502626	0,621620239
8349	ENSG00000213930	-0,00422618	3,968583318	0,0009366	0,975847858
8350	ENSG00000213949	0,178720728	6,860459873	0,5731598	0,456655024
8351	ENSG00000213965	-0,477993229	3,666622761	12,586892	0,013361607
8352	ENSG00000213995	0,07394417	4,773583252	0,3631128	0,707129356
8353	ENSG00000214013	-0,142238454	4,714770789	1,8413827	0,240865404
8354	ENSG00000214021	0,373291007	3,81242339	2,3330973	0,140219972
8355	ENSG00000214022	-0,013270864	5,306372549	0,0072706	0,93278274
8356	ENSG00000214029	0,153699956	3,957709874	0,876413	0,375591111
8357	ENSG00000214046	0,054877658	4,679982785	0,1399727	0,711713647
8358	ENSG00000214078	0,1036933	4,593615822	0,2709419	0,607651495
8359	ENSG00000214097	-0,932759281	4,597482182	7,0868379	0,013889769
8360	ENSG00000214106	0,205814162	3,585912519	1,4733478	0,298899172
8361	ENSG00000214113	-0,106248574	4,080069034	0,6958376	0,537701644
8362	ENSG00000214135	-0,328982898	4,873412102	1,9995298	0,170677414
8363	ENSG00000214174	-0,000322027	4,150132941	3,38E-06	0,998548837
8364	ENSG00000214194	-0,388319809	6,398889533	1,542686	0,226674339
8365	ENSG00000214199	0,382974519	5,801532673	1,6538223	0,211174178
8366	ENSG00000214223	0,387645564	5,93863651	5,1805932	0,032421418
8367	ENSG00000214253	-0,056476538	6,037934594	0,0708012	0,792533021
8368	ENSG00000214331	0,0252359	3,795763247	0,0116333	0,915039264
8369	ENSG00000214357	-0,244162473	5,404675872	1,3848243	0,251258646
8370	ENSG00000214367	0,070153099	3,970816063	0,2683614	0,660041239
8371	ENSG00000214389	0,262232062	6,928130789	1,0075364	0,325887371
8372	ENSG00000214391	-0,186407854	4,136690587	1,2661366	0,272042268
8373	ENSG00000214413	-0,14654678	4,530624731	1,4234627	0,302820087
8374	ENSG00000214425	0,316834075	5,507525373	0,9980463	0,328128662
8375	ENSG00000214456	-0,19641083	4,597641784	0,5529127	0,464622353
8376	ENSG00000214485	-0,006531904	7,549848153	0,0004817	0,982678957

8377	ENSG00000214517	-0,005147041	4,653917325	0,002301	0,962151662
8378	ENSG00000214544	0,039142762	4,539848881	0,0641823	0,953383067
8379	ENSG00000214548	-0,477062692	6,00181615	3,7245978	0,065978866
8380	ENSG00000214652	0,415566862	4,156647487	2,4233705	0,133125619
8381	ENSG00000214655	0,233816424	4,660494031	1,1811584	0,288325001
8382	ENSG00000214717	0,061902895	5,193595427	0,1047509	0,749111598
8383	ENSG00000214753	0,035804234	4,711599641	0,0554257	0,815951096
8384	ENSG00000214765	-0,063042486	4,104519304	0,2366562	0,685814569
8385	ENSG00000214784	0,300478777	5,603668991	1,4880678	0,234818167
8386	ENSG00000214810	-0,853929185	4,237398167	4,6508691	0,041676751
8387	ENSG00000214941	0,05544413	3,949497469	0,2137964	0,75807951
8388	ENSG00000214960	0,161639128	4,196745271	0,4559771	0,506210041
8389	ENSG00000214967	-0,120556103	4,520716216	0,3371289	0,56710998
8390	ENSG00000214975	-0,092094455	5,198388536	0,2716891	0,607159717
8391	ENSG00000215021	-0,006536572	6,196781657	0,0025632	0,960055121
8392	ENSG00000215030	0,431628293	5,904367547	3,80892	0,063201143
8393	ENSG00000215093	0,280471545	4,375484186	1,2663895	0,271995634
8394	ENSG00000215105	0,30481404	4,26615113	5,4611711	0,041182658
8395	ENSG00000215114	-0,232042936	5,150603732	2,6024414	0,120253497
8396	ENSG00000215126	-0,349450467	5,581111864	7,8368925	0,010134425
8397	ENSG00000215158	0,130139677	4,033815913	0,6846886	0,452880308
8398	ENSG00000215193	0,089287791	4,791885084	0,2722976	0,606759779
8399	ENSG00000215246	-0,166846075	3,855264223	0,2889778	0,596014791
8400	ENSG00000215251	-0,464900572	4,611931602	11,290614	0,002686485
8401	ENSG00000215252	0,134655153	6,060689871	0,4844192	0,493367435
8402	ENSG00000215301	-0,168000342	8,154863461	0,8527162	0,365322487
8403	ENSG00000215302	0,264247961	4,162076395	0,7530751	0,394425517
8404	ENSG00000215305	0,013383008	3,815232021	0,0049933	0,996824514
8405	ENSG00000215386	-0,265490134	6,807019139	3,1150316	0,090780528
8406	ENSG00000215421	-0,246835786	5,58369691	5,9858743	0,022409949
8407	ENSG00000215492	0,366848784	6,036302156	3,9174203	0,059820691
8408	ENSG00000215712	-0,32983908	4,550173113	6,7618294	0,018430415
8409	ENSG00000215717	0,124390162	5,705382879	0,6699496	0,42142838
8410	ENSG00000215790	0,123653735	4,796929061	0,4354468	0,515850815
8411	ENSG00000215817	-0,017490945	4,232579454	0,0178	0,913488992
8412	ENSG00000215861	-0,450574855	8,54234621	2,371146	0,137176371
8413	ENSG00000216285	0,069174393	4,822277193	0,1744646	0,680028865
8414	ENSG00000216866	0,004819831	4,369723743	0,0005815	0,98096876
8415	ENSG00000216937	0,35770325	5,258439856	2,076222	0,163021234
8416	ENSG00000217128	0,080772883	6,255112936	0,4888365	0,491399347
8417	ENSG00000217716	0,272415334	4,695787392	2,1758703	0,153683248
8418	ENSG00000218283	-0,151136354	5,693258477	1,0394941	0,318489939
8419	ENSG00000218336	0,489544083	4,128427465	4,3242924	0,048850643
8420	ENSG00000218418	0,257489166	4,14677835	2,5649808	0,122834427
8421	ENSG00000218426	0,148644511	6,08100961	0,854579	0,364811656
8422	ENSG00000218739	-0,263691418	5,011630524	4,1313967	0,053697198
8423	ENSG00000219023	0,262426704	4,728950866	0,6328921	0,434391106
8424	ENSG00000219133	-0,052749082	5,430194075	0,0623931	0,804959487
8425	ENSG00000219201	0,051638122	3,656575058	0,1251071	0,766634057
8426	ENSG00000219481	0,367703589	6,008123241	1,9322315	0,177758038
8427	ENSG00000219507	-0,076977623	4,065505371	0,1045837	0,749304758
8428	ENSG00000219545	-0,010028317	3,733990891	0,0036965	0,976714544
8429	ENSG00000219626	-0,002563688	3,827571123	0,0003179	0,994383477
8430	ENSG00000220205	0,177032266	4,83764649	0,9351418	0,343540982
8431	ENSG00000220749	-0,016043739	6,651078863	0,0043139	0,948197402
8432	ENSG00000220793	-0,037758483	7,648698961	0,0255874	0,874301915
8433	ENSG00000220842	-0,034712702	8,343448395	0,0204933	0,887408679
8434	ENSG00000221818	-0,771882726	5,075675356	12,885207	0,001540258
8435	ENSG00000221823	0,112155211	4,503626359	0,629203	0,435690306

8436	ENSG00000221866	0,756825309	5,615890735	3,0620649	0,093406719
8437	ENSG00000221869	-0,134519027	5,638515107	0,207649	0,652868678
8438	ENSG00000221890	0,322701857	4,447029007	1,0370692	0,319046162
8439	ENSG00000221914	-0,097059426	5,634568037	0,6741441	0,419970035
8440	ENSG00000221923	0,113549071	4,12541251	0,9312902	0,42265857
8441	ENSG00000221962	-0,160602161	3,804850861	0,7777838	0,386899863
8442	ENSG00000221963	0,384499765	6,594826595	0,8204417	0,374397167
8443	ENSG00000221968	-0,247634365	5,084781611	2,0480811	0,16578127
8444	ENSG00000221978	0,100572535	6,094886079	0,5152873	0,480031948
8445	ENSG00000221983	0,155462589	7,471559956	0,6920164	0,413991902
8446	ENSG00000221986	-1,022362328	6,388645042	17,077788	0,000401282
8447	ENSG00000222011	-0,085144572	3,63181884	0,3064189	0,670335635
8448	ENSG00000222041	0,432850167	4,445220524	1,9232376	0,178731195
8449	ENSG00000222489	0,092795618	4,332712928	0,0995024	0,755258137
8450	ENSG00000223247	0,87719187	5,554993697	2,2539363	0,146814057
8451	ENSG00000223361	-0,150059716	6,157002248	0,365677	0,55126119
8452	ENSG00000223416	-0,024513343	4,145502664	0,0050607	0,943899605
8453	ENSG00000223459	0,216867924	4,067946293	0,933702	0,343905518
8454	ENSG00000223482	-0,220861638	5,883415689	1,2856473	0,268475781
8455	ENSG00000223496	0,086933787	4,846232443	0,2376825	0,630477331
8456	ENSG00000223501	0,17711301	4,471968781	1,3308278	0,272696113
8457	ENSG00000223509	0,104736669	4,546950939	0,1386199	0,713048948
8458	ENSG00000223529	0,368706152	3,891356832	1,6212752	0,215571854
8459	ENSG00000223546	0,101861064	3,676984995	0,9090583	0,583528797
8460	ENSG00000223547	0,230658189	3,826613977	1,5221925	0,229687396
8461	ENSG00000223551	0,607821933	4,144385057	2,4506171	0,131068626
8462	ENSG00000223705	0,040524416	4,618867928	0,0386048	0,845951066
8463	ENSG00000223745	0,058427408	4,08391003	0,0970156	0,758232999
8464	ENSG00000223865	1,20201528	5,596065907	20,19269	0,000162785
8465	ENSG00000223891	-0,146543207	3,804880019	0,891325	0,39664639
8466	ENSG00000223930	-0,803636138	4,776886024	10,063503	0,004233574
8467	ENSG00000223959	-0,301225626	3,749265424	2,9933531	0,10385289
8468	ENSG00000223960	-0,024894569	4,018956364	0,0199156	0,888995931
8469	ENSG00000224078	0,172997434	9,609359062	0,3096794	0,583225412
8470	ENSG00000224094	-0,028989319	4,912002565	0,0125243	0,911859324
8471	ENSG00000224389	0,952011991	6,88474476	2,7082777	0,113361286
8472	ENSG00000224411	-0,100365251	4,717587706	0,1850412	0,671060561
8473	ENSG00000224470	0,263937763	5,218322114	2,1802803	0,153285071
8474	ENSG00000224531	-0,105321787	4,614485095	0,4242772	0,521234887
8475	ENSG00000224546	0,328906552	5,185270683	3,2866616	0,082854336
8476	ENSG00000224578	0,377601162	5,724588649	7,0653685	0,014000508
8477	ENSG00000224597	-0,204935085	4,462203723	2,567048	0,133718192
8478	ENSG00000224631	0,219983896	5,233588596	0,8591325	0,363559949
8479	ENSG00000224660	-0,040902531	4,123418534	0,0467521	0,830711334
8480	ENSG00000224712	0,029806682	3,731965606	0,0212463	0,931334616
8481	ENSG00000224819	-0,433094953	4,368246563	1,0648408	0,312791386
8482	ENSG00000224858	0,200935605	4,261533846	1,0797899	0,309493654
8483	ENSG00000224861	-0,089866882	5,669736803	0,3930937	0,536809667
8484	ENSG00000224877	-0,129979924	4,344060623	0,5670521	0,459034763
8485	ENSG00000224914	-0,071066036	4,142811465	0,1803055	0,675037504
8486	ENSG00000224957	-0,136338241	3,553613733	0,3024573	0,587621792
8487	ENSG00000225031	0,294836188	5,822745095	2,9255037	0,100581953
8488	ENSG00000225071	-0,076989958	4,470075987	0,0467981	0,830629488
8489	ENSG00000225137	-0,103169069	3,844607527	0,445889	0,606101611
8490	ENSG00000225151	0,201457185	4,342829525	1,1447815	0,295691097
8491	ENSG00000225178	0,115889435	6,198438587	0,2067337	0,653581318
8492	ENSG00000225190	0,038172679	4,156154127	0,0314382	0,860810897
8493	ENSG00000225405	0,27891637	3,678526059	0,7380588	0,399106179
8494	ENSG00000225470	-0,234010458	4,465224271	0,6107697	0,442430559

8495	ENSG00000225484	-0,092589349	5,629539792	0,188243	0,668405959
8496	ENSG00000225630	0,083540476	11,07023934	0,0655765	0,800155167
8497	ENSG00000225648	0,177188133	4,365894783	1,4667988	0,23806482
8498	ENSG00000225695	0,284500857	3,783427973	4,8053219	0,116009988
8499	ENSG00000225733	0,292953153	6,568164903	6,0191314	0,022078973
8500	ENSG00000225746	-1,398965992	5,010454721	8,1370102	0,008983623
8501	ENSG00000225793	-0,148205086	4,011017356	0,120101	0,732060223
8502	ENSG00000225830	0,152947836	5,458095919	1,5229663	0,229529865
8503	ENSG00000225921	-0,2149767	4,990235148	4,0281697	0,089802271
8504	ENSG00000225972	-0,050135672	9,046140735	0,0345855	0,854089351
8505	ENSG00000226084	0,05043552	7,026050151	0,0361208	0,850925478
8506	ENSG00000226085	-0,526508275	5,055132919	9,1585269	0,005986152
8507	ENSG00000226121	0,075282908	4,704528279	0,3857123	0,547674819
8508	ENSG00000226149	0,198655793	3,868287487	0,9410947	0,34203962
8509	ENSG00000226221	0,073789749	5,353723462	0,0726432	0,78991814
8510	ENSG00000226259	-0,011582824	4,541953637	0,0035327	0,953115566
8511	ENSG00000226306	-0,378543626	6,475851767	1,4100909	0,247102206
8512	ENSG00000226312	-0,460369033	4,166812563	1,9612456	0,174662379
8513	ENSG00000226380	0,049423961	3,92990965	0,0222629	0,882684199
8514	ENSG00000226415	-0,44388753	4,914395122	5,4888879	0,028108744
8515	ENSG00000226419	0,228214576	3,629290024	0,8026199	0,379547448
8516	ENSG00000226479	-0,09213422	4,799235929	0,4226721	0,522001293
8517	ENSG00000226564	-0,080755671	4,325576258	0,105407	0,748355304
8518	ENSG00000226688	-0,197278779	3,64273374	1,0183127	0,323367867
8519	ENSG00000226752	0,713680852	4,528347819	6,2391371	0,020050127
8520	ENSG00000226784	-0,018939315	4,937000489	0,0214715	0,884768026
8521	ENSG00000226891	-0,08166489	3,77911801	0,0714334	0,791631462
8522	ENSG00000226950	-0,191219261	4,535242888	1,3011799	0,26568011
8523	ENSG00000226964	-0,316418874	3,663297832	5,715802	0,111297321
8524	ENSG00000227008	-0,041188869	4,884729225	0,0240171	0,878186845
8525	ENSG00000227028	0,006499337	4,657525333	0,0003733	0,984750471
8526	ENSG00000227057	0,051481556	4,09063831	0,0809417	0,815132079
8527	ENSG00000227063	0,121549605	7,624756006	0,3336229	0,569118291
8528	ENSG00000227081	0,350827692	5,832425296	2,5734315	0,122250563
8529	ENSG00000227097	0,405975907	4,401980357	2,9537925	0,099044495
8530	ENSG00000227124	0,113322333	4,100278195	0,5964855	0,476616604
8531	ENSG00000227232	0,06042111	4,379133256	0,088517	0,768728811
8532	ENSG00000227345	-0,069464161	5,450671766	0,1587854	0,693934555
8533	ENSG00000227372	0,056081602	4,445491552	0,137453	0,817159287
8534	ENSG00000227615	0,172325426	4,0442653	0,6898815	0,41470251
8535	ENSG00000227671	-0,137820729	4,05155725	0,5077425	0,483249829
8536	ENSG00000227729	-0,869492722	4,202064999	4,3850992	0,047415536
8537	ENSG00000227733	0,4797204	3,769465386	5,0412602	0,034609641
8538	ENSG00000227827	0,499340564	4,285079973	4,37643	0,047617199
8539	ENSG00000228223	0,33475964	4,898853649	7,0997596	0,013790032
8540	ENSG00000228253	0,251069713	9,11012344	0,4478469	0,509989302
8541	ENSG00000228327	-0,054525843	4,828050573	0,0544481	0,817550662
8542	ENSG00000228463	-0,035446138	3,83624297	0,0341741	0,854949507
8543	ENSG00000228474	-0,118870724	5,976257808	0,4148305	0,525868431
8544	ENSG00000228502	0,435825103	8,780561303	2,3770469	0,136711408
8545	ENSG00000228532	-0,004984025	3,738114012	0,0014485	0,993983509
8546	ENSG00000228589	-0,121490097	4,045332684	0,8029727	0,476048053
8547	ENSG00000228695	0,292761386	4,088934888	0,8327773	0,370891749
8548	ENSG00000228696	-0,512511308	4,528013184	3,4651321	0,075441318
8549	ENSG00000228716	0,111473528	3,964858141	0,2015512	0,657652826
8550	ENSG00000228794	-0,230686653	6,102649969	0,9825457	0,331835613
8551	ENSG00000228929	0,038933984	4,225498273	0,032514	0,858475823
8552	ENSG00000229117	0,161197855	9,253647501	0,5377993	0,470711981
8553	ENSG00000229119	-0,027171769	6,049737905	0,0095616	0,922947445

8554	ENSG00000229180	-0,119760594	4,079262457	0,3972462	0,534697289
8555	ENSG00000229236	0,02677164	4,217879795	0,0033164	0,954571783
8556	ENSG00000229240	-0,188317316	4,269965487	0,3489462	0,5604428
8557	ENSG00000229344	-0,277233033	7,817917762	1,1341588	0,297889583
8558	ENSG00000229358	-0,136939112	4,606765035	0,2142182	0,647809281
8559	ENSG00000229419	0,096243237	5,120813976	0,522965	0,476809699
8560	ENSG00000229503	-0,005262164	4,626324982	0,0003153	0,985985872
8561	ENSG00000229605	0,007033861	6,140762516	0,0008111	0,977523531
8562	ENSG00000229619	-0,028952231	3,711860499	0,0467922	0,897044322
8563	ENSG00000229638	0,087758468	6,356506753	0,1797488	0,675509061
8564	ENSG00000229644	-0,458120065	6,063831041	1,6214681	0,215545457
8565	ENSG00000229939	0,205781622	3,378813414	1,14673	0,308471279
8566	ENSG00000229944	-0,204272827	3,821244513	0,5917783	0,449522811
8567	ENSG00000230076	0,287533117	5,191188963	1,3178886	0,262715012
8568	ENSG00000230084	-0,134284013	4,541877133	0,3195893	0,5773016
8569	ENSG00000230124	-0,157856345	4,389370326	2,4022422	0,277947065
8570	ENSG00000230202	0,03907859	5,375739415	0,0374899	0,848162474
8571	ENSG00000230204	-0,025452426	4,957480032	0,0122862	0,912697589
8572	ENSG00000230207	0,131839659	4,646687479	0,3675585	0,550246801
8573	ENSG00000230291	0,135234002	4,456915829	0,5126493	0,481166198
8574	ENSG00000230373	0,160550789	3,889696184	0,7398912	0,398530563
8575	ENSG00000230383	0,04252547	4,750830071	0,0294425	0,865254704
8576	ENSG00000230409	-0,023999806	3,843873478	0,0367661	0,931179008
8577	ENSG00000230551	0,025065393	5,081928562	0,0056575	0,940689918
8578	ENSG00000230590	-0,039909175	7,496602706	0,0289929	0,866277163
8579	ENSG00000230606	0,658978828	4,169543973	6,2657295	0,019816074
8580	ENSG00000230629	0,199842934	5,668107691	0,6079624	0,443467641
8581	ENSG00000230630	-0,044476485	4,188225151	0,039817	0,843583846
8582	ENSG00000230943	-1,630150547	4,117202781	27,581992	2,46573E-05
8583	ENSG00000230989	-0,396509652	6,249793575	4,8948448	0,037089165
8584	ENSG00000231006	0,007571345	5,627328653	0,0006326	0,980149316
8585	ENSG00000231074	-0,034719431	5,685828557	0,034105	0,855094443
8586	ENSG00000231341	-0,441391856	3,978555299	4,9297994	0,036479598
8587	ENSG00000231365	-0,062145505	4,061566475	0,115906	0,736592888
8588	ENSG00000231389	1,143144613	6,477707115	14,068509	0,001035722
8589	ENSG00000231500	0,180436422	8,708609298	0,5008016	0,486223514
8590	ENSG00000231607	-0,142516664	3,728871645	0,43074	0,518107366
8591	ENSG00000231721	-0,216249658	5,54514798	1,0613139	0,3135764
8592	ENSG00000231747	0,195491551	3,65456568	0,5069015	0,483608485
8593	ENSG00000231811	-0,538241112	4,421561514	0,8077088	0,378066308
8594	ENSG00000231925	0,173041083	5,583823345	0,6977535	0,41209149
8595	ENSG00000231991	0,246492818	4,583415754	0,7417624	0,397944036
8596	ENSG00000232112	-0,322121081	5,204286606	1,3369351	0,259387365
8597	ENSG00000232119	-0,287247342	5,210437982	4,915442	0,036691965
8598	ENSG00000232187	0,000942554	5,564188901	2,014E-05	0,996458109
8599	ENSG00000232310	0,774542922	3,977892997	5,1975551	0,032165776
8600	ENSG00000232385	0,291631708	4,312971016	0,8934164	0,354330403
8601	ENSG00000232388	-0,352302872	4,259561531	2,9617083	0,098619183
8602	ENSG00000232573	0,495703909	5,86307851	2,1509197	0,155959562
8603	ENSG00000232593	0,155660114	4,150258457	0,6207215	0,438785113
8604	ENSG00000232653	0,224557119	4,956917064	1,6088024	0,217287509
8605	ENSG00000232931	0,324501386	4,495600189	1,9187052	0,179224078
8606	ENSG00000233024	0,110380857	4,766957642	0,3584692	0,555180758
8607	ENSG00000233045	0,361601875	4,097070931	2,8714017	0,103600315
8608	ENSG00000233057	0,416934134	4,403022924	1,727453	0,201631158
8609	ENSG00000233117	0,531673353	5,819198213	3,3113984	0,081778493
8610	ENSG00000233178	-0,39690787	5,094713498	1,5354878	0,227726931
8611	ENSG00000233237	-0,215608128	6,097602525	0,849645	0,366174987
8612	ENSG00000233254	0,030454918	4,163732577	0,0111288	0,916894661

8613	ENSG00000233276	0,162743514	5,739271199	0,6156917	0,440621585
8614	ENSG00000233369	0,247636953	7,500700404	2,6933346	0,114260997
8615	ENSG00000233396	-0,103133086	4,615327129	0,1888818	0,667879591
8616	ENSG00000233426	0,302627668	3,936929504	5,5868452	0,094021415
8617	ENSG00000233476	0,441812847	10,69961457	2,9072949	0,101557017
8618	ENSG00000233559	-0,381094287	3,457992602	1,4148241	0,246333429
8619	ENSG00000233766	0,159475802	3,355566306	0,4447934	0,511421641
8620	ENSG00000233913	0,281604635	6,356942678	1,7244052	0,202015402
8621	ENSG00000233927	0,008641554	6,536679572	0,0010022	0,975016739
8622	ENSG00000233954	-0,536491146	4,611168374	4,8138853	0,038545406
8623	ENSG00000234127	0,117940701	4,236158248	0,5713417	0,512817731
8624	ENSG00000234268	0,327772325	3,529185336	1,6387476	0,213196936
8625	ENSG00000234287	0,334280984	5,373226124	2,077095	0,162936504
8626	ENSG00000234352	-0,453161772	5,455442389	0,8845668	0,356680437
8627	ENSG00000234354	0,079157333	4,27889277	0,0575988	0,812448005
8628	ENSG00000234420	0,150924278	4,493273369	0,5445843	0,467966102
8629	ENSG00000234444	0,046031825	4,429802969	0,1369181	0,742003541
8630	ENSG00000234456	0,047517186	6,705504022	0,0999781	0,754689815
8631	ENSG00000234498	0,177605309	4,756199677	0,527777	0,474836726
8632	ENSG00000234520	-0,848282802	6,744421776	5,4721601	0,028325556
8633	ENSG00000234545	-0,105049993	4,556944018	0,3919846	0,537392584
8634	ENSG00000234585	-0,024777401	3,592496817	0,034312	0,882928806
8635	ENSG00000234616	0,108968061	4,023820711	0,1931401	0,664397508
8636	ENSG00000234618	-0,146844914	4,479639672	0,3362007	0,567640285
8637	ENSG00000234648	0,528497692	4,597471804	2,7292714	0,112046619
8638	ENSG00000234684	-0,042707522	3,593072118	0,0701079	0,7935267
8639	ENSG00000234741	0,541905271	5,631935531	7,0477968	0,014122127
8640	ENSG00000234742	0,128336256	3,735938984	0,2078608	0,652704043
8641	ENSG00000234745	0,23863899	7,949888379	1,3354253	0,259649136
8642	ENSG00000234797	0,274400243	7,524309492	1,1251321	0,29977504
8643	ENSG00000234851	0,107311157	4,705548532	0,134305	0,717343071
8644	ENSG00000234964	-1,029402712	4,529657094	18,518496	0,000261963
8645	ENSG00000234975	0,015912745	5,196324759	0,0038485	0,95106707
8646	ENSG00000235050	-0,502331833	5,24792578	1,5336092	0,228002643
8647	ENSG00000235109	0,404682653	4,001031939	2,2216276	0,149611412
8648	ENSG00000235160	-0,304667511	5,240256583	0,6443572	0,43031484
8649	ENSG00000235174	0,178741533	6,69770643	0,6134659	0,441438192
8650	ENSG00000235244	0,014043914	3,705462641	0,003067	0,956310933
8651	ENSG00000235257	-0,015401723	3,551136546	0,0037945	0,951411651
8652	ENSG00000235436	-0,408406427	3,658028867	1,7688353	0,196502575
8653	ENSG00000235437	0,255032846	4,56986066	1,9601115	0,174782143
8654	ENSG00000235508	-0,10203137	3,638881082	0,1187575	0,733502075
8655	ENSG00000235535	-0,058487832	6,365305464	0,011615	0,915106053
8656	ENSG00000235552	0,065380516	7,355259965	0,0736579	0,788492783
8657	ENSG00000235652	0,174792539	3,888158807	0,9115756	0,349576663
8658	ENSG00000235655	-0,061009404	5,706619477	0,2464055	0,624289305
8659	ENSG00000235703	0,322637262	3,840821836	2,9350455	0,100060289
8660	ENSG00000235750	-0,617578871	5,094689777	6,7742266	0,015876334
8661	ENSG00000235954	-0,077302321	4,057242838	0,2445574	0,625601633
8662	ENSG00000236088	0,106367084	3,483259044	0,4307056	0,609953568
8663	ENSG00000236287	-0,276843191	5,682402591	4,5582088	0,043544791
8664	ENSG00000236404	-0,085077043	4,169761961	0,1379582	0,71370248
8665	ENSG00000236438	-0,143946315	4,190850659	0,3140662	0,580587874
8666	ENSG00000236439	0,290031279	5,534839693	1,5685309	0,222945351
8667	ENSG00000236534	-0,005778229	4,007040864	0,0022571	0,999736648
8668	ENSG00000236552	0,249885287	6,440148794	1,2732934	0,270726861
8669	ENSG00000236581	-0,327231041	5,06218727	2,3594979	0,138099708
8670	ENSG00000236824	-0,213615839	4,35378845	0,2082624	0,65239223
8671	ENSG00000236876	0,567281897	4,475139998	3,5063492	0,073839355

8672	ENSG00000237094	-0,226094792	4,092511989	0,5077205	0,483259223
8673	ENSG00000237125	0,006999317	6,91808009	0,0012152	0,972490955
8674	ENSG00000237187	0,441463255	4,287619559	3,9364029	0,059250648
8675	ENSG00000237190	-0,163161264	4,113838277	1,218502	0,326824487
8676	ENSG00000237238	-0,656992271	3,872330401	6,1419753	0,020931783
8677	ENSG00000237296	-0,210892036	6,5529048	0,7734883	0,388192781
8678	ENSG00000237298	-0,381558985	7,409970927	0,635305	0,433528195
8679	ENSG00000237440	0,184528987	4,220432644	1,1424937	0,296162735
8680	ENSG00000237441	0,010283731	5,04323474	0,0039624	0,950349716
8681	ENSG00000237491	-0,325166581	3,763105198	2,9418847	0,099688313
8682	ENSG00000237506	-0,011284499	5,297566628	0,0026923	0,959064239
8683	ENSG00000237550	0,212329357	7,999192076	0,8069297	0,378292527
8684	ENSG00000237596	0,687322341	3,985227442	5,7860385	0,02455004
8685	ENSG00000237748	-0,906666771	5,95042776	7,217151	0,013144287
8686	ENSG00000237765	-0,056146928	4,596792123	0,1040205	0,749956523
8687	ENSG00000237973	-0,136134682	13,62010213	0,3163628	0,579197995
8688	ENSG00000237975	0,263672996	3,785077592	0,7340141	0,400381159
8689	ENSG00000237984	0,003390948	4,076985018	0,0003345	0,985564581
8690	ENSG00000237986	-0,183664833	4,681551571	0,4976319	0,487591862
8691	ENSG00000238083	-0,347692419	5,236478306	2,6878475	0,114658046
8692	ENSG00000238103	0,242401176	7,358055158	0,9943676	0,329003207
8693	ENSG00000238741	0,005871644	7,479871772	0,0003105	0,986092005
8694	ENSG00000239246	0,303586572	4,103964249	2,7056787	0,113525295
8695	ENSG00000239305	-0,111984363	5,441314243	0,5554756	0,463601289
8696	ENSG00000239306	-0,114389016	4,377150411	0,2785548	0,602680601
8697	ENSG00000239470	-0,038918266	5,188500377	0,0336122	0,856133045
8698	ENSG00000239474	-1,237869677	6,638725297	6,8349067	0,01546735
8699	ENSG00000239665	-0,037356917	4,513046644	0,0328988	0,857650356
8700	ENSG00000239797	-0,034749694	5,327725961	0,0193632	0,890535749
8701	ENSG00000239883	-0,157120424	5,004776168	0,5606729	0,461542007
8702	ENSG00000239899	0,463284371	6,173929288	1,0403439	0,318299775
8703	ENSG00000240036	0,025044062	4,030392962	0,01474	0,904416764
8704	ENSG00000240038	0,084879286	5,364273231	0,2332274	0,633683928
8705	ENSG00000240087	0,130926208	5,393601493	0,3204809	0,576774634
8706	ENSG00000240184	0,230733339	3,921677578	0,6573373	0,425771706
8707	ENSG00000240225	-0,049535176	4,013943937	0,1395966	0,777653223
8708	ENSG00000240342	0,117391104	6,06575915	0,3123364	0,581624972
8709	ENSG00000240344	-0,062205134	4,108066244	0,1014025	0,753012706
8710	ENSG00000240376	0,270978343	4,008652754	1,4836091	0,235499258
8711	ENSG00000240445	0,098860245	4,431540884	0,3155231	0,579717308
8712	ENSG00000240583	0,042123097	8,18768483	0,0100543	0,920993702
8713	ENSG00000240674	-0,009920372	4,186582483	0,0015028	0,969409388
8714	ENSG00000240857	-0,482514607	4,824807354	7,5594915	0,011388197
8715	ENSG00000240869	0,36422294	8,069102278	0,6328568	0,434403733
8716	ENSG00000240914	0,410321326	4,313863093	3,7625722	0,064711055
8717	ENSG00000241058	0,026551265	4,128442959	0,0527073	0,84465375
8718	ENSG00000241135	-1,145230136	5,216148104	12,281086	0,001897221
8719	ENSG00000241158	-0,634443107	4,281203482	2,1112982	0,159658278
8720	ENSG00000241258	0,041064315	4,440001332	0,1310566	0,879418174
8721	ENSG00000241316	-0,225238443	3,867992757	2,1520433	0,198349381
8722	ENSG00000241399	0,126536311	3,856320754	0,3672179	0,550430187
8723	ENSG00000241429	0,339358418	5,964619152	1,6648342	0,209711709
8724	ENSG00000241458	0,163870081	4,293361792	0,3381606	0,566521647
8725	ENSG00000241468	-0,507463046	5,461576081	7,6646799	0,010901958
8726	ENSG00000241506	-0,390456007	5,286331511	2,8777331	0,103241691
8727	ENSG00000241549	0,344436251	3,334603866	3,5748686	0,118237037
8728	ENSG00000241553	-0,09012695	4,564882014	0,277621	0,603285636
8729	ENSG00000241560	-0,070697361	3,853337926	0,1082864	0,745066795
8730	ENSG00000241612	-0,018358201	3,967211411	0,0059687	0,939083871

8731	ENSG00000241644	1,925553859	6,400936023	10,233321	0,003972267
8732	ENSG00000241684	0,30689414	4,129756834	3,0958905	0,091722289
8733	ENSG00000241685	-0,064572328	3,838192547	0,2479405	0,749808233
8734	ENSG00000241837	-0,734687927	6,593127544	10,536097	0,003549181
8735	ENSG00000241839	-0,057412244	4,492447523	0,0596348	0,809228865
8736	ENSG00000241860	-0,354383998	3,934491768	1,0796644	0,309521146
8737	ENSG00000241878	0,052153031	4,138902616	0,0690178	0,795099776
8738	ENSG00000241973	-0,047045686	5,988985551	0,0460278	0,832006919
8739	ENSG00000242071	0,355377141	5,955710528	3,4055213	0,077829196
8740	ENSG00000242086	-0,318958064	5,905610717	1,4306066	0,243792111
8741	ENSG00000242193	-0,170382964	3,803645368	0,8163915	0,375558617
8742	ENSG00000242247	-0,230382578	5,218465324	3,0632172	0,093302437
8743	ENSG00000242259	0,020117081	5,002620353	0,0303958	0,900659783
8744	ENSG00000242299	0,104758641	4,917096437	0,0993553	0,755433005
8745	ENSG00000242372	-0,134003613	3,924561913	0,376055	0,545710059
8746	ENSG00000242485	-0,379349947	5,455703475	5,1897775	0,032282714
8747	ENSG00000242498	0,286515166	4,539460217	1,7573697	0,19790719
8748	ENSG00000242571	-0,039853895	4,686112806	0,0228361	0,881195355
8749	ENSG00000242759	-0,261980959	3,789628358	1,7095348	0,203903221
8750	ENSG00000243071	0,015099117	5,252130571	0,0033771	0,954157935
8751	ENSG00000243147	-0,696899136	6,27038967	11,378529	0,002610811
8752	ENSG00000243156	0,237571608	5,6379154	0,5994544	0,44663445
8753	ENSG00000243181	0,087192208	4,464423571	0,1186735	0,733592428
8754	ENSG00000243199	0,062138943	4,19494995	0,0555809	0,815698504
8755	ENSG00000243244	0,039869617	4,350830138	0,0143791	0,9055883
8756	ENSG00000243317	-0,087848018	5,131132996	0,4120871	0,527206288
8757	ENSG00000243335	0,151201202	4,348876938	1,06333	0,373602193
8758	ENSG00000243701	-0,257769288	4,249794095	3,3696539	0,083671309
8759	ENSG00000243716	0,148700315	7,71295743	0,364988	0,55163355
8760	ENSG00000243927	-0,67583571	4,520553738	5,5567756	0,027247652
8761	ENSG00000243943	0,029764457	4,284854372	0,0912648	0,813722962
8762	ENSG00000244005	-0,094169055	4,344009879	0,4009673	0,532797406
8763	ENSG00000244021	-0,022670483	6,940618299	0,0104336	0,919522418
8764	ENSG00000244038	0,020044541	6,187924387	0,0205809	0,887166037
8765	ENSG00000244086	0,159718329	4,950826195	0,2933175	0,593285343
8766	ENSG00000244198	-0,132917043	3,867935386	0,2911678	0,594634097
8767	ENSG00000244245	0,221337448	4,1072759	0,6861188	0,415959537
8768	ENSG00000244313	0,239717683	5,403121847	1,2457505	0,27583548
8769	ENSG00000244363	-0,026896707	6,225895105	0,0078999	0,929941934
8770	ENSG00000244398	0,027397904	5,167264173	0,0129064	0,910531006
8771	ENSG00000244405	0,607249811	4,42231942	10,931955	0,003068846
8772	ENSG00000244462	0,036523248	5,930456876	0,0983862	0,756578437
8773	ENSG00000244509	0,006924468	4,197455487	0,0012297	0,9723275
8774	ENSG00000244642	0,441327972	6,715597914	1,4238672	0,244873168
8775	ENSG00000244682	-0,384745509	4,615092044	3,0473339	0,094151467
8776	ENSG00000244716	0,091565115	6,636527619	0,1305918	0,721101715
8777	ENSG00000244731	0,881298222	6,853816416	2,5299897	0,125287212
8778	ENSG00000244734	2,946441625	9,181845313	9,7062893	0,00484705
8779	ENSG00000244754	-0,112943074	8,15892071	0,2979478	0,590401955
8780	ENSG00000244879	0,230557978	5,025610072	0,6569454	0,425907788
8781	ENSG00000245205	0,436341744	5,748129523	2,8254988	0,106244217
8782	ENSG00000245532	-0,700262258	11,44192091	1,9220299	0,178862363
8783	ENSG00000245662	0,362384711	5,111285698	6,0073398	0,022219466
8784	ENSG00000245680	0,102832243	4,601783812	0,8333597	0,454466692
8785	ENSG00000245910	-0,447157672	5,079420449	4,7035853	0,040633883
8786	ENSG00000245937	-0,214208172	5,252351031	2,0107567	0,16952983
8787	ENSG00000245958	-0,11719877	4,223237926	0,632718	0,434450871
8788	ENSG00000246067	-0,378891585	4,137469483	6,0659819	0,021651421
8789	ENSG00000246695	0,047522749	4,363531994	0,0958213	0,843433366

8790	ENSG00000246705	0,062655711	4,343267727	0,074873	0,786799833
8791	ENSG00000246859	0,327444947	3,662103454	1,4443516	0,241606172
8792	ENSG00000247077	-0,125797118	4,079933702	0,6130297	0,44159851
8793	ENSG00000247516	-0,395975193	3,776723844	4,8834739	0,037289918
8794	ENSG00000247556	0,04855101	7,690874134	0,1126647	0,7401479
8795	ENSG00000247572	-0,293031426	3,727325672	1,5991579	0,218625895
8796	ENSG00000247627	0,090419929	7,062320406	0,1227959	0,729195178
8797	ENSG00000247809	-0,202260718	4,183436488	0,9272983	0,345533392
8798	ENSG00000247828	-0,408083771	6,64664981	2,4429188	0,131645976
8799	ENSG00000248019	-0,073004441	4,347814167	0,1023808	0,751865585
8800	ENSG00000248092	-0,287168068	5,663295869	1,2989894	0,266072063
8801	ENSG00000248124	0,085005173	4,203874461	0,4394597	0,592518118
8802	ENSG00000248144	0,316313056	4,181282538	0,60247	0,445507875
8803	ENSG00000248152	-0,44981252	3,925026404	1,5442724	0,226443183
8804	ENSG00000248290	0,043774652	5,376953564	0,0198003	0,889315403
8805	ENSG00000248323	-0,660461113	4,327911325	2,7668476	0,109737797
8806	ENSG00000248333	-0,078685208	4,682820315	0,2703953	0,608006697
8807	ENSG00000248334	-0,121467747	3,797865123	0,7305943	0,507354227
8808	ENSG00000248483	-0,193805813	3,512347305	1,0989086	0,305344564
8809	ENSG00000248527	-0,017545262	11,00298665	0,0062763	0,937534514
8810	ENSG00000248932	-0,074986322	3,71651688	0,1595834	0,693207871
8811	ENSG00000249242	0,216631314	4,297578135	0,9512177	0,339507565
8812	ENSG00000249264	0,338636537	6,008860531	1,1484915	0,294928394
8813	ENSG00000249353	0,125762343	4,072922473	0,3018573	0,587990113
8814	ENSG00000249437	0,092402347	5,968344168	0,2197851	0,64359549
8815	ENSG00000249669	-0,876790228	6,492675072	7,0981054	0,013823497
8816	ENSG00000249839	0,211304021	4,026710095	1,8070438	0,242124636
8817	ENSG00000249855	0,501067153	7,752562507	4,219316	0,051445237
8818	ENSG00000250007	-1,300298419	4,02233135	12,072717	0,002040601
8819	ENSG00000250159	-0,227531067	4,721289953	0,9283104	0,345275388
8820	ENSG00000250182	0,435188875	8,100416811	2,555892	0,123466044
8821	ENSG00000250303	-0,770122797	4,749385305	10,330111	0,003831288
8822	ENSG00000250305	-0,871599069	5,735568844	8,4659749	0,007868315
8823	ENSG00000250312	0,072210117	3,554763077	0,1007791	0,753746847
8824	ENSG00000250317	-0,364990865	4,542426484	3,5955651	0,07050343
8825	ENSG00000250479	-0,378888518	6,726251286	2,6397932	0,117777385
8826	ENSG00000250497	-0,602266434	3,861358489	3,8938552	0,060537043
8827	ENSG00000250722	0,268454911	6,109839033	0,8421508	0,368259837
8828	ENSG00000250891	0,777549687	5,890949543	3,1998917	0,086759111
8829	ENSG00000250903	-0,077060349	5,073662896	0,1803148	0,67502965
8830	ENSG00000251022	0,051305199	4,415328937	0,1229077	0,729070934
8831	ENSG00000251034	-0,186651166	3,337479674	1,3189633	0,38830568
8832	ENSG00000251136	0,101565775	5,252489261	0,2834013	0,599561447
8833	ENSG00000251158	0,092769062	3,782256939	0,0737206	0,78840507
8834	ENSG00000251322	0,382382892	4,97697298	2,291011	0,143680705
8835	ENSG00000251369	-0,034433549	3,811848267	0,0552596	0,830740922
8836	ENSG00000251562	-0,231012343	13,24957697	0,6857407	0,416056817
8837	ENSG00000251615	-0,21179061	3,918858456	0,6410907	0,43147006
8838	ENSG00000251634	0,148843843	4,914867267	0,767595	0,389977072
8839	ENSG00000251791	0,636274428	5,558187694	4,4451291	0,04604528
8840	ENSG00000252010	0,888313786	7,403720068	4,6598545	0,041496886
8841	ENSG00000252316	1,638927222	5,500537883	4,5455142	0,043852475
8842	ENSG00000252835	0,588559419	4,193640291	2,6836292	0,11492796
8843	ENSG00000253203	0,255703405	3,81985887	2,0829376	0,167108112
8844	ENSG00000253250	-0,579225578	3,962691644	4,1949918	0,05206845
8845	ENSG00000253276	-0,357608522	4,495518146	1,1641913	0,29172985
8846	ENSG00000253284	-0,113948891	4,682014563	0,1086858	0,744614497
8847	ENSG00000253305	-0,18487191	3,833156551	0,6304055	0,435283181
8848	ENSG00000253352	0,116557476	6,691072396	1,1061046	0,303764853

8849	ENSG00000253366	0,405917744	3,978109655	5,8356764	0,024006015
8850	ENSG00000253368	-0,584965219	4,384944648	8,0057361	0,009476423
8851	ENSG00000253710	-0,133869017	3,923636072	1,3242142	0,457249127
8852	ENSG00000253719	0,093681968	5,959189538	0,7615647	0,39178439
8853	ENSG00000253729	0,170379917	7,624392325	1,6776388	0,207982836
8854	ENSG00000253731	-0,138804903	4,076642739	0,2465502	0,62420435
8855	ENSG00000253738	-0,398459182	5,804120209	2,2418244	0,147855331
8856	ENSG00000253816	0,32990942	3,60625912	3,0370373	0,099178111
8857	ENSG00000253846	-0,231916458	3,841404198	0,7604062	0,392170104
8858	ENSG00000254004	0,091999561	4,608571276	0,5240341	0,476364525
8859	ENSG00000254087	-0,075186252	4,145508837	0,0842504	0,774206212
8860	ENSG00000254122	-0,004965043	3,525582901	0,0005504	0,981483445
8861	ENSG00000254206	0,156320923	4,607847026	0,433725	0,516674239
8862	ENSG00000254373	-0,778387129	4,437712785	6,4102603	0,01859588
8863	ENSG00000254634	-0,063489832	5,15881057	0,0772143	0,783579219
8864	ENSG00000254635	0,049925271	4,687278749	0,2339736	0,667261665
8865	ENSG00000254685	-0,05759126	4,301311126	0,0634928	0,803285392
8866	ENSG00000254701	0,478272635	3,619431742	6,2718642	0,019762511
8867	ENSG00000254838	0,362720414	3,679782156	2,2279649	0,149057717
8868	ENSG00000254852	-0,127039444	3,846877962	0,3109352	0,58246786
8869	ENSG00000254999	0,049145565	6,128370527	0,1467487	0,705150449
8870	ENSG00000255112	0,107772989	5,066088918	1,0369271	0,343749879
8871	ENSG00000255248	0,216433385	6,562021068	1,6001492	0,218487859
8872	ENSG00000255302	-0,157961967	7,962472715	0,8350743	0,370227298
8873	ENSG00000255399	-0,493869905	5,055095521	2,5312759	0,125196036
8874	ENSG00000255529	0,100479686	5,561047059	0,2803488	0,601521982
8875	ENSG00000255583	0,152010423	3,885309045	0,6035367	0,445110471
8876	ENSG00000255717	-0,185577238	4,576540933	1,7356155	0,200570203
8877	ENSG00000255823	-0,40407149	5,996302294	0,3983817	0,534118929
8878	ENSG00000256025	-0,374973438	3,954421687	0,8421188	0,368268776
8879	ENSG00000256043	0,474134264	4,903645444	7,6465339	0,010984176
8880	ENSG00000256053	-0,442555087	3,692120149	6,1061412	0,02613177
8881	ENSG00000256087	-0,056901288	4,458557082	0,2109026	0,720630986
8882	ENSG00000256223	0,025557292	4,128978682	0,0295453	0,870371355
8883	ENSG00000256235	0,024175407	4,259568026	0,0075242	0,931623737
8884	ENSG00000256338	0,112724404	5,453066255	0,255861	0,617768526
8885	ENSG00000256393	0,145701836	6,763533628	0,5267524	0,475260997
8886	ENSG00000256537	-0,328875186	4,988055136	2,8060535	0,107387894
8887	ENSG00000256683	-0,081465412	3,908450583	0,3327114	0,701279695
8888	ENSG00000256751	-0,195789474	3,789380419	1,0858947	0,308160547
8889	ENSG00000256771	-0,11953823	3,894681547	0,5460043	0,494460632
8890	ENSG00000257017	3,247766519	8,692053776	5,118745	0,033373019
8891	ENSG00000257027	-0,011865174	4,146634778	0,0033544	0,954312648
8892	ENSG00000257093	-0,011756896	5,431647234	0,0072281	0,932977161
8893	ENSG00000257103	0,18057741	6,061163659	3,7121591	0,066356607
8894	ENSG00000257176	-0,235016447	3,562036598	1,2630753	0,272607491
8895	ENSG00000257218	0,015893152	3,69109907	0,0222266	0,935588112
8896	ENSG00000257267	-0,259694483	5,943495945	2,408218	0,134286118
8897	ENSG00000257354	-0,436473841	3,718963834	3,599189	0,070371615
8898	ENSG00000257599	0,405205314	5,230321001	14,449715	0,000909901
8899	ENSG00000257621	-0,136061331	5,721912487	0,6808982	0,417713275
8900	ENSG00000257923	0,238092062	6,663989491	1,5395906	0,227126214
8901	ENSG00000258289	0,079312956	6,014646765	0,1394226	0,712258655
8902	ENSG00000258441	0,229037931	4,39549302	1,3867419	0,250940048
8903	ENSG00000258667	0,078615291	4,103291574	0,0854454	0,772657137
8904	ENSG00000258890	0,109141741	5,061567198	0,6970531	0,412293195
8905	ENSG00000259030	-0,630955912	5,09157918	1,4866026	0,23504171
8906	ENSG00000259295	0,112141756	3,936953219	0,2550679	0,61831089
8907	ENSG00000259319	-0,238198522	4,107674807	0,7780018	0,386834428

8908	ENSG00000259330	-0,309319037	5,213383996	3,2864479	0,0828637
8909	ENSG00000259781	-0,050452665	5,501964955	0,0648978	0,801168907
8910	ENSG00000259820	-0,243742319	5,061666574	1,0570148	0,314536898
8911	ENSG00000259956	0,194466653	4,614918374	1,3420847	0,258497086
8912	ENSG00000259959	-1,76839625	4,903694912	11,001334	0,002992245
8913	ENSG00000260032	0,412016718	8,962508587	10,304715	0,003856321
8914	ENSG00000260075	-0,029482463	4,203948628	0,0218355	0,883807079
8915	ENSG00000260244	0,416021559	4,973662387	3,0760076	0,092708107
8916	ENSG00000260314	-0,846745563	6,294720232	8,8235904	0,006825957
8917	ENSG00000260528	-0,271164164	3,918934994	0,6837355	0,416758744
8918	ENSG00000260565	-0,051669823	5,396114161	0,0555171	0,815802354
8919	ENSG00000260691	-0,14696391	4,490264524	0,3536856	0,557811919
8920	ENSG00000260916	-0,029137881	4,61680866	0,0274564	0,869834168
8921	ENSG00000261115	-0,32878219	6,294449441	0,7866892	0,384239607
8922	ENSG00000261326	-0,112421867	3,942599265	0,2345308	0,632741928
8923	ENSG00000261371	-0,218098879	7,582833297	1,7835044	0,194708722
8924	ENSG00000261377	-0,597151367	3,872391022	16,624717	0,00067418
8925	ENSG00000261499	-0,169269712	4,309682216	0,5290185	0,474323445
8926	ENSG00000261553	0,12476033	4,40471756	0,1231465	0,728825075
8927	ENSG00000261556	-0,138101368	4,542450634	0,4620099	0,503437566
8928	ENSG00000261609	-0,279831526	4,514478889	2,2815099	0,144476016
8929	ENSG00000261701	3,128940537	6,287022331	6,8530199	0,01534754
8930	ENSG00000261799	-0,019783277	3,550463025	0,0149774	0,904005502
8931	ENSG00000261824	-0,141220713	5,05122578	1,0019717	0,327177776
8932	ENSG00000262074	1,866575314	12,18400159	3,8964341	0,060458173
8933	ENSG00000262655	0,617880847	5,674484072	3,4687285	0,075299964
8934	ENSG00000262879	0,131251122	4,975062156	0,4549951	0,506663889
8935	ENSG00000262902	-0,375440157	3,756504089	1,6365196	0,213497945
8936	ENSG00000263001	0,19650152	7,801961007	1,9003012	0,181196943
8937	ENSG00000263002	0,013436455	3,991257342	0,0123566	0,935293032
8938	ENSG00000263006	0,262777581	4,068592949	1,9997239	0,170657497
8939	ENSG00000263155	-0,566386932	7,163826535	4,5079036	0,044659946
8940	ENSG00000263266	0,031491699	5,570791027	0,0160822	0,900183012
8941	ENSG00000263465	0,298619772	4,990325868	9,5275569	0,008495915
8942	ENSG00000263563	-0,062140196	4,132540831	0,1446035	0,7152781
8943	ENSG00000263740	0,484614535	13,60461997	1,0587792	0,314142213
8944	ENSG00000263753	-0,178688975	5,346623706	2,0914942	0,161500141
8945	ENSG00000263934	1,215028891	12,3667957	2,6411949	0,117685001
8946	ENSG00000263956	0,190072379	5,298093024	1,2325934	0,278320638
8947	ENSG00000264112	0,199545718	5,04019561	0,4565418	0,505949377
8948	ENSG00000264247	-0,304860649	3,981579423	4,0688327	0,081589198
8949	ENSG00000264281	0,147208786	6,854332791	0,4831819	0,49391429
8950	ENSG00000264343	0,236770849	4,52467842	1,2775941	0,269940401
8951	ENSG00000264364	-0,20011343	6,90858988	1,4601513	0,239124347
8952	ENSG00000264522	0,19896291	5,197324379	1,9434559	0,176527902
8953	ENSG00000264538	-0,186782811	4,092380453	1,0126759	0,324682388
8954	ENSG00000264940	1,388616741	11,42138044	2,9575402	0,098842865
8955	ENSG00000265107	-0,460745486	5,794682802	1,5745483	0,222088272
8956	ENSG00000265142	-0,90880604	6,79260668	3,785378	0,063963016
8957	ENSG00000265185	1,174934216	11,45593408	2,6398809	0,117771605
8958	ENSG00000265241	-0,059217888	5,115967728	0,3243483	0,667508206
8959	ENSG00000265354	-0,444043628	5,469535727	11,32136	0,002656042
8960	ENSG00000265491	0,274369976	7,11699669	2,3542403	0,13851889
8961	ENSG00000265735	0,500113132	13,43191651	1,166589	0,291245459
8962	ENSG00000265808	0,10880154	6,395486743	0,5211378	0,477594387
8963	ENSG00000265817	-0,063536246	3,55337215	0,1011446	0,786688985
8964	ENSG00000265972	0,180433205	10,2357891	0,76577	0,390500905
8965	ENSG00000266019	0,002956754	4,981955427	8,585E-05	0,992686634
8966	ENSG00000266028	0,189082456	5,753028572	0,6516814	0,42774206

8967	ENSG00000266094	0,242891863	3,855179065	1,7022013	0,204842291
8968	ENSG00000266173	-0,120473155	3,698756644	0,303118	0,587216712
8969	ENSG00000266338	0,237817206	5,465297128	2,1439632	0,156601437
8970	ENSG00000266412	-0,495245796	8,52555002	4,2140044	0,051580603
8971	ENSG00000266472	-0,390792217	5,561677214	5,1814487	0,032408469
8972	ENSG00000266714	0,217492	5,228970596	0,7011289	0,410979598
8973	ENSG00000267002	0,009061858	3,929567954	0,0020671	0,964126885
8974	ENSG00000267023	-0,369726865	3,571239023	4,4098839	0,05559713
8975	ENSG00000267041	0,113462322	3,545074863	0,7326782	0,554145554
8976	ENSG00000267519	0,771349067	4,738020572	3,6130217	0,069871055
8977	ENSG00000267541	-0,331877824	4,403697605	1,048319	0,31649203
8978	ENSG00000267680	-0,05900016	3,795727256	0,2560565	0,769106494
8979	ENSG00000267784	-0,06541569	6,625584058	0,0163888	0,899241428
8980	ENSG00000268043	0,186486354	6,303249046	0,7184757	0,405336769
8981	ENSG00000268205	0,15317894	5,467769371	0,7253043	0,403147608
8982	ENSG00000268350	0,247719606	4,785888115	2,4468228	0,131352812
8983	ENSG00000268362	-0,39333263	4,719368967	2,0284647	0,167738712
8984	ENSG00000269001	0,113706494	3,919595393	0,8116751	0,49401127
8985	ENSG00000269028	0,547280337	7,090462332	1,4818215	0,235773031
8986	ENSG00000269067	0,18917326	3,483265758	1,6683124	0,343971619
8987	ENSG00000269293	-0,176324284	3,874970455	1,1569059	0,314588845
8988	ENSG00000269343	-0,029077408	4,278949614	0,0298527	0,864329035
8989	ENSG00000269378	-0,018688869	6,333072	0,009083	0,924894048
8990	ENSG00000269556	0,017149243	4,977413368	0,0102405	0,920268004
8991	ENSG00000269713	0,259105855	5,578610132	2,0209191	0,168499109
8992	ENSG00000269821	-0,296181301	6,477246645	0,7510801	0,395042612
8993	ENSG00000269893	0,239156369	4,373627267	1,1648358	0,291599554
8994	ENSG00000269929	-0,120121026	3,835778269	0,5107663	0,486398614
8995	ENSG00000270025	0,104334507	4,280415048	0,2645138	0,61191881
8996	ENSG00000270231	0,240181635	5,825786408	1,6427629	0,212655796
8997	ENSG00000270629	0,328164228	6,314764973	2,3277523	0,140653935
8998	ENSG00000270647	-0,047000643	5,941238128	0,1135544	0,73916446
8999	ENSG00000270872	0,261689024	3,707950577	1,1443529	0,295779378
9000	ENSG00000271121	-0,078062733	4,533134511	0,1739966	0,680433105
9001	ENSG00000271204	-0,16315817	3,331477047	0,3243238	0,574514453
9002	ENSG00000271254	0,491783571	5,354660442	3,2945759	0,082508366
9003	ENSG00000271394	0,30976526	7,074742448	0,9110801	0,349705173
9004	ENSG00000271401	-0,344063768	4,722840737	0,7729353	0,388359703
9005	ENSG00000271425	0,319006234	6,127020979	3,3028883	0,082146787
9006	ENSG00000271533	-0,026446639	5,35495687	0,013057	0,910012638
9007	ENSG00000271601	0,399239668	4,917731469	5,76876	0,024742621
9008	ENSG00000271952	-0,068128145	4,708012584	0,0445102	0,834756255
9009	ENSG00000272047	-0,163069764	4,841707344	0,7673958	0,390037582
9010	ENSG00000272055	0,50433169	3,929490561	2,1445002	0,156551772
9011	ENSG00000272150	0,275658427	4,412944869	2,9387516	0,099858519
9012	ENSG00000272168	0,345327239	3,643932661	1,3304636	0,260511845
9013	ENSG00000272316	0,106807547	3,713380169	0,3560949	0,556483689
9014	ENSG00000272335	-0,027328307	3,63284611	0,0453208	0,906498601
9015	ENSG00000272391	0,117840604	4,636093419	0,2264545	0,638632529
9016	ENSG00000272419	0,021493439	3,815104656	0,0150099	0,903550464
9017	ENSG00000272602	-0,127578278	4,108421768	0,6802184	0,417942471
9018	ENSG00000272886	-0,085196824	5,08981325	0,737409	0,399280059
9019	ENSG00000272888	-0,198056045	5,645986187	1,1638548	0,291797917
9020	ENSG00000273015	-0,090216029	4,950818946	0,3269855	0,572959442
9021	ENSG00000273018	-0,084583162	3,560967194	0,1450883	0,706752712
9022	ENSG00000273033	0,173261724	3,843752187	0,7354083	0,399940986
9023	ENSG00000273136	0,316489458	5,892169681	1,9782993	0,172873457
9024	ENSG00000273319	-0,22326832	3,955202372	0,5759433	0,455577148
9025	ENSG00000273559	-0,067213207	3,982994764	0,3236114	0,679449776

9026	ENSG00000273590	-0,224942016	4,097750118	3,0683533	0,178116067
9027	ENSG00000273611	-0,095535431	4,043873352	0,2988396	0,642496369
9028	ENSG00000273654	-0,204788976	4,348011411	0,8804959	0,357768958
9029	ENSG00000273748	-0,077909464	3,708483259	0,0963892	0,758988975
9030	ENSG00000273749	0,18452893	5,783633598	0,9626311	0,336684127
9031	ENSG00000273768	0,625159567	10,07459488	1,1867414	0,287216218
9032	ENSG00000273841	-0,364191352	4,904781645	2,2991012	0,143007617
9033	ENSG00000274070	-0,431457878	4,740400451	1,4564137	0,239708492
9034	ENSG00000274211	0,216457308	4,545471953	1,3721491	0,253377698
9035	ENSG00000274265	-0,118636694	5,280525192	0,7304162	0,401497966
9036	ENSG00000274333	-0,471578499	4,379270082	2,1101165	0,15977021
9037	ENSG00000274349	-0,319447846	4,682707914	1,9599891	0,174795067
9038	ENSG00000274487	-0,072739834	4,674882859	0,1386039	0,713064743
9039	ENSG00000274523	0,050324153	4,898849684	0,0856846	0,772348487
9040	ENSG00000274615	-0,012740691	4,64355033	0,003935	0,950521317
9041	ENSG00000274642	-0,373581339	6,834149395	2,0610422	0,164503097
9042	ENSG00000275023	0,238392165	6,235068764	1,2248873	0,279789923
9043	ENSG00000275052	0,230429287	6,157714643	2,0601121	0,164594418
9044	ENSG00000275066	0,172575609	5,370720489	2,1804804	0,153220306
9045	ENSG00000275131	-0,142723065	9,615168558	0,2458025	0,624727779
9046	ENSG00000275325	-0,323889509	3,832381154	5,3794228	0,06586946
9047	ENSG00000275405	0,722592851	12,45320956	1,4072804	0,24756015
9048	ENSG00000275700	0,088637186	4,603129107	0,561026	0,538012544
9049	ENSG00000275832	0,007822535	5,128876779	0,0011699	0,973007955
9050	ENSG00000275835	-0,013614648	4,604197408	0,0157141	0,901322145
9051	ENSG00000275895	-0,207297685	5,524472509	1,8047927	0,19217668
9052	ENSG00000275993	1,297238427	5,012126002	11,618426	0,002396117
9053	ENSG00000276023	0,038684658	3,900294496	0,0612197	0,868662622
9054	ENSG00000276077	-0,521342199	3,774206174	2,3140466	0,141774046
9055	ENSG00000276141	0,03097387	3,998122532	0,0442406	0,837487837
9056	ENSG00000276168	0,517211164	15,11203125	0,9414528	0,341949589
9057	ENSG00000276203	-0,121135522	3,760251415	0,1683018	0,685403475
9058	ENSG00000276234	0,011974283	4,236158776	0,0177597	0,94469145
9059	ENSG00000276291	-0,158982991	4,213592873	0,8905468	0,35509002
9060	ENSG00000276293	0,197065198	6,070936716	1,8306183	0,18911686
9061	ENSG00000276386	-0,983051973	4,642279087	5,6056902	0,026645462
9062	ENSG00000276550	-0,568808816	6,925313593	4,5537965	0,043676878
9063	ENSG00000276966	0,394138528	5,139212315	1,3345558	0,259800059
9064	ENSG00000276975	0,757176559	4,201924422	9,5764297	0,005093751
9065	ENSG00000277053	0,220749813	7,606939441	2,2700478	0,145395551
9066	ENSG00000277067	-0,489490691	4,117598911	2,0914717	0,161548759
9067	ENSG00000277072	-0,157863146	4,17557766	0,5822604	0,453146177
9068	ENSG00000277157	0,177348045	3,558044779	0,2038024	0,655876502
9069	ENSG00000277406	0,171130926	4,201130249	1,1324853	0,298237939
9070	ENSG00000277443	0,427157537	5,930262108	2,992808	0,096968616
9071	ENSG00000277701	0,791819528	3,771123629	10,544521	0,003538138
9072	ENSG00000277734	-1,387849805	4,935358605	67,11875	2,74217E-08
9073	ENSG00000277778	-0,358904475	5,060843073	1,9209748	0,178977054
9074	ENSG00000277791	-0,490822779	5,600287437	8,239357	0,008619002
9075	ENSG00000277918	0,657233461	10,27196656	1,2820868	0,269122046
9076	ENSG00000277991	-0,576709382	4,043749749	3,1787317	0,087743265
9077	ENSG00000278053	-0,134943021	4,726062218	1,9027716	0,282292461
9078	ENSG00000278129	0,07215275	3,840958008	0,0989107	0,755962149
9079	ENSG00000278259	-0,2684058	4,02277712	2,302515	0,142724722
9080	ENSG00000278311	-0,031643114	5,892082055	0,1239953	0,797036859
9081	ENSG00000278318	0,064428724	3,527200159	0,2617801	0,741545415
9082	ENSG00000278540	-0,476306768	5,922638707	3,6563378	0,068329698
9083	ENSG00000278730	-0,364893677	4,446400837	5,0866024	0,03387989
9084	ENSG00000278771	0,516749489	11,3741666	1,3217219	0,262040851

9085	ENSG00000278845	-0,182054645	4,569482135	2,5908289	0,197743437
9086	ENSG00000278931	0,099659465	3,570431061	0,1878894	0,668697821
9087	ENSG00000278974	-0,542168699	3,831414808	13,424563	0,003756218
9088	ENSG00000279159	-0,162149465	4,763286323	0,4749028	0,497600688
9089	ENSG00000279166	-0,255850672	4,181106197	1,0820777	0,30899315
9090	ENSG00000279170	-0,005774152	3,862122411	0,0021843	0,979273552
9091	ENSG00000279289	-0,22225384	3,598863025	0,9363907	0,34322525
9092	ENSG00000279453	-0,088801793	3,677307342	0,3593318	0,69119521
9093	ENSG00000279457	0,046895779	4,856858943	0,0582627	0,811391781
9094	ENSG00000279483	0,111315091	7,485924189	0,2822793	0,600280437
9095	ENSG00000279598	-0,159700883	4,242344466	0,2148482	0,647329083
9096	ENSG00000279662	-0,108382179	3,392270268	0,1601377	0,692704419
9097	ENSG00000279688	-0,824818391	3,766555074	6,1456657	0,02089752
9098	ENSG00000279800	0,030235207	4,96326371	0,0366211	0,849909685
9099	ENSG00000280077	-0,452275689	3,945231341	2,692039	0,11439059
9100	ENSG00000280109	-0,081362391	3,726767937	0,0879898	0,769397732
9101	ENSG00000280128	-0,154483737	3,953485086	0,699373	0,411557432
9102	ENSG00000280138	0,092897085	4,21888037	0,064178	0,802250088
9103	ENSG00000280219	0,112061884	4,480264392	0,107933	0,745467817
9104	ENSG00000280339	0,586608566	3,873144139	4,8714744	0,037503092
9105	ENSG00000280347	-0,031411884	3,61964722	0,027134	0,87059344
9106	ENSG00000280383	-0,252094744	3,46922408	0,6915234	0,414155841
9107	ENSG00000280385	-0,202974445	3,890538877	0,6902931	0,414565388
9108	ENSG00000280433	-0,012206359	4,157331059	0,0122181	0,926561834
9109	ENSG00000280614	2,659485189	8,901405546	4,252544	0,05060749
9110	ENSG00000280739	0,10895102	3,970744931	0,276713	0,603875168
9111	ENSG00000280800	2,558184452	8,673835352	3,3460194	0,08029964
9112	ENSG00000281026	-0,241722703	4,932537614	1,0321319	0,320176048
9113	ENSG00000281181	2,145508881	11,77398385	1,6403647	0,212978791
9114	ENSG00000281183	-0,207779555	3,70926319	0,5755325	0,45573597
9115	ENSG00000281195	-0,161376478	3,770967882	0,7212917	0,404431808
9116	ENSG00000281344	0,592530057	4,129624694	1,0823104	0,308942303
9117	ENSG00000281383	2,289596039	8,442452301	2,4072452	0,134361029
9118	ENSG00000281649	0,163859291	5,595077953	1,7252864	0,201859669
9119	ENSG00000282458	-0,372407064	4,629715178	1,7200899	0,202560987
9120	ENSG00000282826	0,021649659	4,11586284	0,0172093	0,896764917
9121	ENSG00000282917	-2,596383002	5,939666868	21,475155	0,000114578
9122	ENSG00000283041	0,258147379	7,626581552	1,8429959	0,187708432
9123	ENSG00000283196	-0,055722451	3,745299858	0,0604861	0,807900253
9124	ENSG00000283498	0,341614926	5,22435852	1,0462515	0,316959335
9125	ENSG00000283674	0,204083244	4,173358268	0,6929838	0,413670528
9126	ENSG00000284024	-0,017156377	3,707710658	0,0322867	0,930044876
9127	ENSG00000284052	0,094635137	3,379700113	0,1644995	0,688777367
9128	ENSG00000284612	-0,680893109	5,181751904	4,2095487	0,051694466
9129	ENSG00000285410	-0,0932429	3,844369528	0,2908316	0,609811816
9130	ENSG00000285437	-0,418105261	4,686164346	1,6083016	0,217356752
9131	ENSG00000285756	0,401597323	4,023072089	1,5607731	0,224056491
9132	ENSG00000285967	-0,171165134	4,328623497	0,9405008	0,342188989
9133	ENSG00000286062	-0,126733026	5,528864129	0,1121298	0,740752218
9134	ENSG00000286071	-0,606720942	5,337440498	2,4174909	0,133574508
9135	ENSG00000286156	-0,012906915	3,713355099	0,0058618	0,992316825
9136	ENSG00000286219	0,156250491	5,014314625	0,5594946	0,462007533
9137	ENSG00000286619	-1,39693761	4,558728029	9,1791447	0,005938302
9138	ENSG00000287134	-0,666386286	3,914658903	7,0092562	0,014355734
9139	ENSG00000287280	-0,398794394	3,764650564	1,2942056	0,266930673
9140	ENSG00000287299	-0,024380776	3,500052529	0,0176496	0,895460329
9141	ENSG00000287315	0,112648824	3,866237235	0,0810744	0,778382318
9142	ENSG00000287891	-1,3833728	5,086343749	8,931342	0,006542433
9143	ENSG00000288066	0,246353424	3,983923778	2,3186534	0,141396368

FDR	Gene name	Gene biotype
0,7924243	DPM1	protein_coding
0,7444016	SCYL3	protein_coding
0,0606893	CFH	protein_coding
0,9045866	FUCA2	protein_coding
0,9767278	GCLC	protein_coding
0,5829929	NFYA	protein_coding
0,8617303	NIPAL3	protein_coding
0,9112805	LAS1L	protein_coding
0,5248895	ENPP4	protein_coding
0,7440219	ANKIB1	protein_coding
0,9621856	KRIT1	protein_coding
0,1800952	LAP3	protein_coding
0,9081173	CD99	protein_coding
0,4879925	LASP1	protein_coding
0,958712	SNX11	protein_coding
0,7797349	TMEM176A	protein_coding
0,9928407	M6PR	protein_coding
0,8622295	KLHL13	protein_coding
0,887319	ICA1	protein_coding
0,9544158	ALS2	protein_coding
0,9840915	CASP10	protein_coding
0,7558125	CFLAR	protein_coding
0,8930787	TFPI	protein_coding
0,4151093	NDUFAF7	protein_coding
0,8189129	RBM5	protein_coding
0,2318378	SLC7A2	protein_coding
0,9773277	ARF5	protein_coding
0,5558351	POLDIP2	protein_coding
0,6766513	PLXND1	protein_coding
0,1431468	AK2	protein_coding
0,6180429	FKBP4	protein_coding
0,7230802	KDM1A	protein_coding
0,611428	RBM6	protein_coding
0,764019	RECQL	protein_coding
0,6648483	VPS50	protein_coding
0,0473388	NDUFAB1	protein_coding
0,997931	PDK4	protein_coding
0,7200284	SLC25A13	protein_coding
0,2354584	ST7	protein_coding
0,8578793	CDC27	protein_coding
0,1974243	HCCS	protein_coding
0,4818782	UPF1	protein_coding
0,0168003	SKAP2	protein_coding
0,8255291	SLC25A5	protein_coding
0,3126419	MCUB	protein_coding
0,6463814	POLR2J	protein_coding
0,9928407	DHX33	protein_coding
0,3522293	MEOX1	protein_coding
0,9644268	THSD7A	protein_coding
0,8500846	LIG3	protein_coding
0,2866571	RPAP3	protein_coding
0,5024248	ACSM3	protein_coding
0,4045325	CIAPIN1	protein_coding
0,0639504	COPZ2	protein_coding
0,8992483	PRKAR2B	protein_coding
0,654521	MSL3	protein_coding
0,9484395	CREBBP	protein_coding

0,9908419	GCFC2	protein_coding
0,768388	CROT	protein_coding
0,933129	ABCB4	protein_coding
0,4509993	KMT2E	protein_coding
0,7309867	IBTK	protein_coding
0,2430128	ZNF195	protein_coding
0,9628671	MYCBP2	protein_coding
0,9998478	FBXL3	protein_coding
0,9233889	PDK2	protein_coding
0,2948143	ITGA3	protein_coding
0,7763012	ZFX	protein_coding
0,7390284	LAMP2	protein_coding
0,4535882	GDE1	protein_coding
0,115935	TMEM98	protein_coding
0,8525767	AP2B1	protein_coding
0,6574161	ZNF263	protein_coding
0,6624016	SPATA20	protein_coding
0,8440006	TNFRSF12A	protein_coding
0,6999614	RALA	protein_coding
0,949756	KDM7A	protein_coding
0,9899513	ETV1	protein_coding
0,8238032	AGK	protein_coding
0,7954765	PHTF2	protein_coding
0,5336185	FARP2	protein_coding
0,6947291	IFRD1	protein_coding
0,7318327	COX10	protein_coding
0,995098	PAF1	protein_coding
0,6613295	VPS41	protein_coding
0,6440403	ELAC2	protein_coding
0,8800304	PNPLA4	protein_coding
0,7639032	ADIPOR2	protein_coding
0,6865358	PROM1	protein_coding
0,9707919	PAFAH1B1	protein_coding
0,995098	KIAA0100	protein_coding
0,8851105	GAS7	protein_coding
0,6898216	ST7L	protein_coding
0,9450135	LUC7L	protein_coding
0,3202177	SELE	protein_coding
0,5364552	DNAJC11	protein_coding
0,9296784	MYLIP	protein_coding
0,6658884	PSMB1	protein_coding
0,7162623	JARID2	protein_coding
0,9925374	CDKL5	protein_coding
0,9417097	NADK	protein_coding
0,2104415	CYTH3	protein_coding
0,7571978	ADAM22	protein_coding
0,4219373	SYPL1	protein_coding
0,6159171	SPAG9	protein_coding
0,1951241	AASS	protein_coding
0,9081173	MGST1	protein_coding
0,9669517	CRY1	protein_coding
0,3872426	NFIX	protein_coding
0,8810791	ST3GAL1	protein_coding
0,9919804	IL32	protein_coding
0,3309793	PKD1	protein_coding
0,9439974	MED24	protein_coding
0,8885864	RHOBTB2	protein_coding
0,8269408	HEATR5B	protein_coding
0,7296863	SEC62	protein_coding

0,8189129	RPS20	protein_coding
0,9917279	CSDE1	protein_coding
0,8908494	UBE3C	protein_coding
0,6318488	REV3L	protein_coding
0,9645423	FAM76A	protein_coding
0,5544626	VTA1	protein_coding
0,4906652	BAZ1B	protein_coding
0,9176584	RANBP9	protein_coding
0,4160411	SPRTN	protein_coding
0,6053522	EEF1AKNMT	protein_coding
0,9296784	DYRK4	protein_coding
0,8304235	ZNF207	protein_coding
0,3607643	UQCRC1	protein_coding
0,784306	STARD3NL	protein_coding
0,0868691	CD9	protein_coding
0,5095965	HHATL	protein_coding
0,5510619	NCAPD2	protein_coding
0,9632674	SEMA3G	protein_coding
0,4307688	NISCH	protein_coding
0,696332	STAB1	protein_coding
0,472118	IDS	protein_coding
0,7230802	CD4	protein_coding
0,9463209	SCMH1	protein_coding
0,9496064	FYN	protein_coding
0,881423	HIVEP2	protein_coding
0,7201269	ELOA	protein_coding
0,9762117	CLCN6	protein_coding
0,4344705	MRC2	protein_coding
0,9925374	TSPAN9	protein_coding
0,8318664	BTBD7	protein_coding
0,9907221	ABHD5	protein_coding
0,3339605	ANOS1	protein_coding
0,738122	AKAP8L	protein_coding
0,435547	MBTD1	protein_coding
0,9781971	UTP18	protein_coding
0,2819771	RNF216	protein_coding
0,5919274	TTC19	protein_coding
0,8561515	PTBP1	protein_coding
0,3049645	SYT7	protein_coding
0,860343	LARS2	protein_coding
0,5964345	PIK3C2A	protein_coding
0,5998801	RABGAP1	protein_coding
0,9194718	DCN	protein_coding
0,7296863	PPP5C	protein_coding
0,8721281	CEP68	protein_coding
0,7984578	MAP4K3	protein_coding
0,645515	TMEM159	protein_coding
0,7444016	BRCA1	protein_coding
0,79362	ERCC1	protein_coding
0,9450135	SEMA3B	protein_coding
0,8922683	MBTPS2	protein_coding
0,9233889	EXTL3	protein_coding
0,892596	ELOVL5	protein_coding
0,8801863	KDM5D	protein_coding
0,9450135	CALCOCO1	protein_coding
0,6785825	UBR7	protein_coding
0,7942035	MAP4K5	protein_coding
0,4562339	EHD3	protein_coding
0,9142106	PSMC4	protein_coding

0,3770345	MAN2B2	protein_coding
0,904174	SLC25A39	protein_coding
0,9781971	MVP	protein_coding
0,6173428	NUB1	protein_coding
0,9170247	PGM3	protein_coding
0,9792506	CLK1	protein_coding
0,9978259	POLR3B	protein_coding
0,8255851	ANGEL1	protein_coding
0,7246609	RNF14	protein_coding
0,8463447	HEBP1	protein_coding
0,9512091	GPRC5A	protein_coding
0,9628671	UFL1	protein_coding
0,8992483	CAPN1	protein_coding
0,4412714	MDH1	protein_coding
0,6864699	SLC30A9	protein_coding
0,7452363	COX15	protein_coding
0,7230802	YAF2	protein_coding
0,6160654	ZMYND11	protein_coding
0,623981	BID	protein_coding
0,9194718	MATR3	protein_coding
0,9039795	RGPD5	protein_coding
0,9082846	NUDCD3	protein_coding
0,8023631	GLT8D1	protein_coding
0,9237476	ATP2C1	protein_coding
0,4835164	RALBP1	protein_coding
0,4467509	RUFY3	protein_coding
0,53819	WWTR1	protein_coding
0,9898099	AGPS	protein_coding
0,7382254	CXorf56	protein_coding
0,5622112	ATP1A2	protein_coding
0,9914435	TTC27	protein_coding
0,7418156	PHLDB1	protein_coding
0,4141191	PRDM11	protein_coding
0,3888096	CD74	protein_coding
0,8189129	HGF	protein_coding
0,5943942	ZRANB1	protein_coding
0,6613295	MNAT1	protein_coding
0,71754	SAMD4A	protein_coding
0,9738181	MRE11	protein_coding
0,599779	SERPINB1	protein_coding
0,7842247	SPAST	protein_coding
0,861259	AQR	protein_coding
0,8500846	FHL1	protein_coding
0,620901	RTF2	protein_coding
0,77251	SLC45A4	protein_coding
0,6607313	RNF10	protein_coding
0,8829877	ZDHHC6	protein_coding
0,6854132	RNH1	protein_coding
0,7235397	NDUFS1	protein_coding
0,7844757	RB1CC1	protein_coding
0,7936863	ERP44	protein_coding
0,3520154	ALAS1	protein_coding
0,624851	BIRC3	protein_coding
0,4413743	AKAP11	protein_coding
0,9908419	GLRX2	protein_coding
0,8847411	SNAPC1	protein_coding
0,8930787	DERA	protein_coding
0,3041449	STRAP	protein_coding
0,9768456	PLEKHO1	protein_coding

0,986868	GCLM	protein_coding
0,895571	UBR2	protein_coding
0,9778442	EHD2	protein_coding
0,5563416	CCDC28A	protein_coding
0,583756	RRAGD	protein_coding
0,0757434	HSF2	protein_coding
0,7162408	PHF20	protein_coding
0,8155634	NR1H3	protein_coding
0,626613	SEC63	protein_coding
0,6896707	KPNA6	protein_coding
0,42916	VIM	protein_coding
0,6137496	FAS	protein_coding
0,9899185	CD44	protein_coding
0,7542355	AGPAT4	protein_coding
0,1232731	BTN3A1	protein_coding
0,9277595	MIPEP	protein_coding
0,3792977	PRKCH	protein_coding
0,38667	IFNGR1	protein_coding
0,32001	VRK2	protein_coding
0,6613295	TNFRSF1B	protein_coding
0,7467854	VEZT	protein_coding
0,8992483	BRD9	protein_coding
0,3358222	SNX1	protein_coding
0,9907221	TBPL1	protein_coding
0,9533676	BCLAF1	protein_coding
0,7842247	SLC39A9	protein_coding
0,9842269	ANK1	protein_coding
0,9358535	RABEP1	protein_coding
0,8423623	NUP160	protein_coding
0,9738194	IKZF2	protein_coding
0,9194718	GRN	protein_coding
0,691951	FAM13B	protein_coding
0,7687197	ARHGAP31	protein_coding
0,5082264	SARS	protein_coding
0,9808899	RANBP3	protein_coding
0,9794449	ARID4A	protein_coding
0,5024248	IFT88	protein_coding
0,7905225	ZCCHC8	protein_coding
0,330331	CHPF2	protein_coding
0,1412577	LRRC7	protein_coding
0,8474324	FUT8	protein_coding
0,9481776	UBA6	protein_coding
0,1926389	GAB2	protein_coding
0,6475905	ATP6V0A1	protein_coding
0,6546449	PIAS1	protein_coding
0,5976843	SLC4A7	protein_coding
0,624851	MAP2K3	protein_coding
0,8003414	TMSB10	protein_coding
0,5626731	RNF19A	protein_coding
0,6977704	PEX3	protein_coding
0,9463928	GABARAPL2	protein_coding
0,1589626	MYOC	protein_coding
0,9181578	SH3YL1	protein_coding
0,6900636	FAM136A	protein_coding
0,9707377	VCL	protein_coding
0,2048374	DAPK2	protein_coding
0,8301309	NSMAF	protein_coding
0,8436853	ADSS	protein_coding
0,5263704	TIMP2	protein_coding

0,9644268	RFC1	protein_coding
0,9410289	TBC1D23	protein_coding
0,9645423	CUL3	protein_coding
0,7558125	MYOM2	protein_coding
0,9205351	AC118549.1	protein_coding
0,4826313	USP2	protein_coding
0,9054206	NSUN2	protein_coding
0,6927966	FBXO42	protein_coding
0,7968733	MFAP3	protein_coding
0,654521	PI4K2B	protein_coding
0,6720329	BOD1L1	protein_coding
0,4237568	MAT2B	protein_coding
0,4894579	TLL1	protein_coding
0,7118106	EDC4	protein_coding
0,997931	TRIO	protein_coding
0,9900815	VCAN	protein_coding
0,9953531	CLEC16A	protein_coding
0,855954	MSR1	protein_coding
0,6109628	MTREX	protein_coding
0,8992483	ZFYVE16	protein_coding
0,8131645	RIPOR1	protein_coding
0,1093101	C6	protein_coding
0,4032255	RAI14	protein_coding
0,2945575	PHLPP2	protein_coding
0,5062136	STAU2	protein_coding
0,8786805	INPP4A	protein_coding
0,6158337	PSMA4	protein_coding
0,2841212	LSG1	protein_coding
0,0639504	TNC	protein_coding
0,7805908	AIFM2	protein_coding
0,7258655	SPATA7	protein_coding
0,9699064	MED17	protein_coding
0,841851	RETSAT	protein_coding
0,6543081	AP2S1	protein_coding
0,9750933	USH2A	protein_coding
0,853092	DCUN1D1	protein_coding
0,7444016	JADE2	protein_coding
0,9282881	LCP2	protein_coding
0,3089259	TRIT1	protein_coding
0,7161818	ADRB1	protein_coding
0,2870346	CUL7	protein_coding
0,7687197	CTNNA1	protein_coding
0,9463209	PHKA2	protein_coding
0,9925374	CNTLN	protein_coding
0,0868691	EPHA3	protein_coding
0,6882013	HSPA5	protein_coding
0,8850351	DSG2	protein_coding
0,605669	OFD1	protein_coding
0,8563699	GPM6B	protein_coding
0,7431973	PREX2	protein_coding
0,702199	WDR37	protein_coding
0,749866	YTHDC2	protein_coding
0,6287358	CTPS2	protein_coding
0,9933219	ATP6V1H	protein_coding
0,6383874	POLR2B	protein_coding
0,6797506	FAM214A	protein_coding
0,4367004	ARAP2	protein_coding
0,5140003	TPR	protein_coding
0,0026674	CP	protein_coding

0,2332141	KIAA0556	protein_coding
0,6651275	XK	protein_coding
0,9693586	ANO2	protein_coding
0,633922	C12orf4	protein_coding
0,2104415	SCML1	protein_coding
0,4894579	WWC3	protein_coding
0,4008367	MAP4	protein_coding
0,5573066	GOPC	protein_coding
0,9730551	USP28	protein_coding
0,7740102	HDAC9	protein_coding
0,7318327	CC2D2A	protein_coding
0,9256429	RRM2B	protein_coding
0,9669517	ZNF800	protein_coding
0,7740102	SNX29	protein_coding
0,5707233	LMO3	protein_coding
0,4750209	MRPS10	protein_coding
0,7924243	RSF1	protein_coding
0,8500846	VPS13D	protein_coding
0,9621631	CELF2	protein_coding
0,7639764	FAM120A	protein_coding
0,8619772	R3HDM1	protein_coding
0,6335222	KITLG	protein_coding
0,8530643	ERCC8	protein_coding
0,4724079	H6PD	protein_coding
0,9808899	VAMP3	protein_coding
0,0943087	PER3	protein_coding
0,1326966	LTBP1	protein_coding
0,8481719	RCN1	protein_coding
0,7025012	ELN	protein_coding
0,9154352	ARID1B	protein_coding
0,8885864	CLPTM1L	protein_coding
0,9781971	NEDD4L	protein_coding
0,4840962	HEXB	protein_coding
0,7213265	PTCD2	protein_coding
0,7968733	JKAMP	protein_coding
0,4260926	DKK3	protein_coding
0,5268669	NFE2L3	protein_coding
0,3515446	MCUR1	protein_coding
0,5095965	LIMA1	protein_coding
0,330331	LETMD1	protein_coding
0,2945575	MAPK9	protein_coding
0,624851	FAM160A2	protein_coding
0,4161706	HERPUD1	protein_coding
0,7253738	PIK3CB	protein_coding
0,4061841	HEBP2	protein_coding
0,4775021	MPHOSPH9	protein_coding
0,9678404	PLEKHA5	protein_coding
0,9439974	SIKE1	protein_coding
0,8118149	RRP12	protein_coding
0,7126143	FNIP2	protein_coding
0,9364287	MSMO1	protein_coding
0,9700303	TTC17	protein_coding
0,7027821	FOXN3	protein_coding
0,5704217	AKR7A2	protein_coding
0,4775021	MRTO4	protein_coding
0,9248086	LAMA3	protein_coding
0,4916085	AP5M1	protein_coding
0,9182782	ANAPC4	protein_coding
0,5060008	TRAPPC3	protein_coding

0,7921707	THRAP3	protein_coding
0,9761718	PHPT1	protein_coding
0,4894579	ARID4B	protein_coding
0,7820981	SDCCAG8	protein_coding
0,998169	KIF1B	protein_coding
0,7763012	TBC1D22A	protein_coding
0,4408807	SYNE2	protein_coding
0,3094135	ATP9A	protein_coding
0,8992483	FAM168A	protein_coding
0,9327336	GALC	protein_coding
0,6887681	NOP58	protein_coding
0,9750933	SZRD1	protein_coding
0,7954765	KCNH2	protein_coding
0,6534387	CUL1	protein_coding
0,9693586	FAM114A2	protein_coding
0,7921707	CYFIP2	protein_coding
0,9860501	TAB2	protein_coding
0,9773277	GINM1	protein_coding
0,5474737	EIF2AK2	protein_coding
0,9925374	USP36	protein_coding
0,9252971	KMT2C	protein_coding
0,9967216	PUM2	protein_coding
0,9510167	MRPL43	protein_coding
0,4806087	HPF1	protein_coding
0,9127443	ZFR	protein_coding
0,9618453	RC3H2	protein_coding
0,9914435	TRAF3IP2	protein_coding
0,472118	DCBLD2	protein_coding
0,8863637	SOAT1	protein_coding
0,9082846	PKP2	protein_coding
0,8550924	GDI2	protein_coding
0,7444016	ATG5	protein_coding
0,7885692	PITHD1	protein_coding
0,9919804	MTA3	protein_coding
0,9917279	USP13	protein_coding
0,7057791	ATP11B	protein_coding
0,673546	CDK14	protein_coding
0,8463447	SEC61A1	protein_coding
0,8322139	PPP1R12A	protein_coding
0,5587702	CAMK2B	protein_coding
0,8969303	POLR3E	protein_coding
0,517991	ATP2B4	protein_coding
0,8682799	RIOK2	protein_coding
0,6762366	YIPF1	protein_coding
0,8901085	NDC1	protein_coding
0,458152	DGKG	protein_coding
0,2785577	PARP12	protein_coding
0,1900988	ALDH18A1	protein_coding
0,7081012	TARBP1	protein_coding
0,1356666	GATB	protein_coding
0,9951314	MXD1	protein_coding
0,8575378	CDK17	protein_coding
0,8992483	SLC2A3	protein_coding
0,902171	YBX3	protein_coding
0,6462349	WNK1	protein_coding
0,8422988	CCAR1	protein_coding
0,9587213	SNRNP40	protein_coding
0,5773011	QSER1	protein_coding
0,6563172	MPC1	protein_coding

0,6638064	ACAA1	protein_coding
0,9766075	BCAT1	protein_coding
0,9973946	HDAC7	protein_coding
0,8238032	NCKAP1	protein_coding
0,6476315	MRPS35	protein_coding
0,9108829	GUCY1B1	protein_coding
0,7611529	SFSWAP	protein_coding
0,8545769	MON2	protein_coding
0,8745989	GPBP1	protein_coding
0,9310151	DGAT2	protein_coding
0,7611529	ZNF112	protein_coding
0,62553	CS	protein_coding
0,9233889	ELMO2	protein_coding
0,9817008	WAPL	protein_coding
0,887319	VMP1	protein_coding
0,7894641	APPBP2	protein_coding
0,6776989	EIF4B	protein_coding
0,6160654	RPL18	protein_coding
0,7913218	U2AF2	protein_coding
0,6766513	EPN1	protein_coding
0,559444	MED29	protein_coding
0,5269672	ZNF275	protein_coding
0,6591127	MTMR1	protein_coding
0,8427405	GPC1	protein_coding
0,9464798	HAGH	protein_coding
0,9183994	RNF4	protein_coding
0,4827579	LIMCH1	protein_coding
0,4724079	INTS13	protein_coding
0,2778507	TM7SF3	protein_coding
0,8575378	CCN5	protein_coding
0,9481776	ST3GAL6	protein_coding
0,3616153	CDON	protein_coding
0,9703483	TAF2	protein_coding
0,9233889	HIPK2	protein_coding
0,7627705	TNPO3	protein_coding
0,2061005	CTSA	protein_coding
0,7961601	SUGP2	protein_coding
0,6604792	SLC12A2	protein_coding
0,902171	SNX24	protein_coding
0,7500896	CNN2	protein_coding
0,8530994	DDX20	protein_coding
0,7558125	BTBD1	protein_coding
0,1720115	FAR2	protein_coding
0,9998538	PMS1	protein_coding
0,6818653	CALCRL	protein_coding
0,4767135	TAF11	protein_coding
0,7393702	ANKS1A	protein_coding
0,7880474	AP3D1	protein_coding
0,8373584	ZNF76	protein_coding
0,6607313	UHRF1BP1	protein_coding
0,9484395	GNAI3	protein_coding
0,615864	IPO5	protein_coding
0,4835164	OAT	protein_coding
0,685088	WDR3	protein_coding
0,9997039	PKN2	protein_coding
0,9391446	TRAM2	protein_coding
0,9998478	NTN1	protein_coding
0,9946475	ANKRD44	protein_coding
0,3520154	KARS	protein_coding

0,9778442	ADAT1	protein_coding
0,150677	NDUFB4	protein_coding
0,771306	SPEN	protein_coding
0,9994264	MYLK	protein_coding
0,8021477	ZC3H15	protein_coding
0,9894339	MAP2K4	protein_coding
0,5267449	SLK	protein_coding
0,7213265	CYB5R4	protein_coding
0,9998538	SEC61A2	protein_coding
0,4684539	ASB1	protein_coding
0,1801696	FAM107B	protein_coding
0,0132445	ME1	protein_coding
0,0606893	TBC1D1	protein_coding
0,9677725	CDK13	protein_coding
0,1225394	MTHFD2	protein_coding
0,875851	SLC9A7	protein_coding
0,6473313	FOXJ2	protein_coding
0,7219856	YBX1	protein_coding
0,633922	PPP2R5A	protein_coding
0,9919804	ELAVL1	protein_coding
0,8189129	TIE1	protein_coding
0,6988495	DIP2B	protein_coding
0,9003006	SMARCD1	protein_coding
0,6879654	KDM4A	protein_coding
0,933129	NFYC	protein_coding
0,9977992	CD84	protein_coding
0,8992483	ELOVL1	protein_coding
0,9953531	ZBTB11	protein_coding
0,7420657	ATXN3	protein_coding
0,9630144	GOLGA5	protein_coding
0,4894579	LRRC40	protein_coding
0,0336588	ISOC1	protein_coding
0,9583708	EML1	protein_coding
0,7740102	TRMT11	protein_coding
0,9965259	THUMPD1	protein_coding
0,9997651	MSANTD3	protein_coding
0,8221205	ATG2B	protein_coding
0,9962215	ARFGEF1	protein_coding
0,5312481	MTFR1	protein_coding
0,7025012	FECH	protein_coding
0,9900815	MYO9A	protein_coding
0,9538485	DDX3Y	protein_coding
0,8131645	PFKP	protein_coding
0,8318664	IDI1	protein_coding
0,9127443	SP100	protein_coding
0,5622112	KLF6	protein_coding
0,8162707	PLPP1	protein_coding
0,4161706	NEO1	protein_coding
0,9074092	TRAM1	protein_coding
0,9752796	PHKA1	protein_coding
0,8440006	TNFRSF1A	protein_coding
0,4906292	EVI5	protein_coding
0,5879554	PKM	protein_coding
0,9578517	DHX29	protein_coding
0,592905	DNTTIP2	protein_coding
0,5175383	METTL22	protein_coding
0,9528743	TP53BP1	protein_coding
0,1444307	RRP15	protein_coding
0,7467854	RHOA	protein_coding

0,8425029	DHX8	protein_coding
0,9847107	ZFY	protein_coding
0,9700303	IARS2	protein_coding
0,8721867	NAV3	protein_coding
0,8318518	IDH3G	protein_coding
0,7639764	ROCK1	protein_coding
0,5880659	CBFB	protein_coding
0,4545648	PDK3	protein_coding
0,8554227	HYAL2	protein_coding
0,9374881	HDAC4	protein_coding
0,933129	HEATR6	protein_coding
0,8545769	MEF2A	protein_coding
0,4684923	OTUD5	protein_coding
0,997931	TFE3	protein_coding
0,8764046	ACSL4	protein_coding
0,8851105	INPP5A	protein_coding
0,855954	GRIPAP1	protein_coding
0,3606951	REEP1	protein_coding
0,7039013	ATP11A	protein_coding
0,9142106	POLR1A	protein_coding
0,8593223	LAPTM4A	protein_coding
0,8885864	TTC7A	protein_coding
0,5953643	IP6K2	protein_coding
0,9233889	SRBD1	protein_coding
0,9998478	KIF2A	protein_coding
0,7296863	PSME4	protein_coding
0,6173428	IFT80	protein_coding
0,2841212	SIRT2	protein_coding
0,4750209	ERLEC1	protein_coding
0,0757434	PYGM	protein_coding
0,9854182	MAST4	protein_coding
0,1763931	ADGRF5	protein_coding
0,9003006	NUP133	protein_coding
0,9781148	NUCKS1	protein_coding
0,9997039	VPS35	protein_coding
0,7965103	DNAJA2	protein_coding
0,5688668	ABCC9	protein_coding
0,8992483	CLEC2D	protein_coding
0,5180247	FUNDC1	protein_coding
0,8318518	MAOB	protein_coding
0,7500896	RORA	protein_coding
0,0349682	TGFBR3	protein_coding
0,2354584	ATP1B3	protein_coding
0,6689227	NEDD4	protein_coding
0,8992483	MAPK6	protein_coding
0,1125192	GNB5	protein_coding
0,3792977	RAB27A	protein_coding
0,7444016	HDHD5	protein_coding
0,5474737	UFD1	protein_coding
0,8472938	LRP6	protein_coding
0,8148845	PHRF1	protein_coding
0,8155634	ELP1	protein_coding
0,7613174	NUCB2	protein_coding
0,9536474	PFN2	protein_coding
0,7444016	PTPN3	protein_coding
0,7894641	SPTB	protein_coding
0,8037001	SLC44A1	protein_coding
0,7921707	TMEM260	protein_coding
0,7752284	SMG6	protein_coding

0,8274546	EXOC5	protein_coding
0,3881918	CLTCL1	protein_coding
0,8917289	FSTL3	protein_coding
0,7609122	DGCR2	protein_coding
0,9746059	ZXDC	protein_coding
0,4160411	JMJD6	protein_coding
0,6173428	POLB	protein_coding
0,9700303	WIPI1	protein_coding
0,8221205	GBA2	protein_coding
0,9880046	NDST1	protein_coding
0,2529607	AP3M2	protein_coding
0,4627775	PABPC1	protein_coding
0,7114715	CFAP20	protein_coding
0,6888533	CSNK2A2	protein_coding
0,2656432	PTPN21	protein_coding
0,3829653	EIF2B3	protein_coding
0,1712026	CAMK2A	protein_coding
0,8255851	TCOF1	protein_coding
0,8062191	CDC42	protein_coding
0,8847101	OSBPL3	protein_coding
0,3607643	ATP2B1	protein_coding
0,8971058	NCK2	protein_coding
0,9914435	MAP4K4	protein_coding
0,9248086	MGAT4A	protein_coding
0,986527	RPL31	protein_coding
0,9172092	WDR1	protein_coding
0,9282881	SNX13	protein_coding
0,8915993	ARHGAP10	protein_coding
0,7912354	RPS6KA2	protein_coding
0,6403073	ING3	protein_coding
0,2104415	VASH1	protein_coding
0,7230802	LMCD1	protein_coding
0,3653635	BUD23	protein_coding
0,90504	SEL1L	protein_coding
0,933129	ATP6AP1	protein_coding
0,4806087	TRIB2	protein_coding
0,5009178	DAZAP1	protein_coding
0,9040068	HLTF	protein_coding
0,9131236	FAM50A	protein_coding
0,4748134	CYBRD1	protein_coding
0,7762612	CDH19	protein_coding
0,4732706	PDCD2	protein_coding
0,7125689	RDH11	protein_coding
0,855954	PRKACA	protein_coding
0,9410184	ACTN1	protein_coding
0,7444016	ZFYVE26	protein_coding
0,998169	EPN2	protein_coding
0,8930787	PTPN18	protein_coding
0,6285359	LIMS2	protein_coding
0,931978	SPEG	protein_coding
0,7444016	LNX1	protein_coding
0,3910224	ALDH3A2	protein_coding
0,9410184	TFRC	protein_coding
0,5358437	SREBF1	protein_coding
0,6168923	AFF4	protein_coding
0,6858794	UBE2D1	protein_coding
0,7689375	MPP5	protein_coding
0,3316892	RHOBTB1	protein_coding
0,9312186	SMC1A	protein_coding

0,6610925	HSD17B10	protein_coding
0,8031748	MARK2	protein_coding
0,90504	CHFR	protein_coding
0,4519396	P4HA2	protein_coding
0,289067	FCGR2B	protein_coding
0,6951729	NFATC3	protein_coding
0,7552965	TRNT1	protein_coding
0,7558125	ACADVL	protein_coding
0,9485776	STK10	protein_coding
0,3798831	FBXW11	protein_coding
0,3217493	EVC	protein_coding
0,8971058	DERL2	protein_coding
0,2533818	MRVI1	protein_coding
0,8630325	AP1M1	protein_coding
0,0796818	PVR	protein_coding
0,9068107	SCARB1	protein_coding
0,962885	ALPK1	protein_coding
0,9998478	PDE8A	protein_coding
0,5511615	CLCN4	protein_coding
0,7118106	SDHA	protein_coding
0,4726081	SMARCE1	protein_coding
0,7559894	KDM5A	protein_coding
0,4799252	PPP2R3A	protein_coding
0,4697514	FERMT2	protein_coding
0,0757434	DHRS9	protein_coding
0,998169	IGF2BP2	protein_coding
0,9127443	MAP3K13	protein_coding
0,4321437	ST6GAL1	protein_coding
0,3765746	VDAC1P1	processed_pseudogene
0,9792506	FRY	protein_coding
0,992369	PICALM	protein_coding
0,8422763	NSF	protein_coding
0,933129	CLASP1	protein_coding
0,8992483	NOTCH3	protein_coding
0,6651622	CLNS1A	protein_coding
0,3316892	TEAD2	protein_coding
0,9406097	EED	protein_coding
0,7182975	TSG101	protein_coding
0,6929857	NCBP3	protein_coding
0,4221375	MGLL	protein_coding
0,8919058	NTN4	protein_coding
0,7740102	BCS1L	protein_coding
0,5180247	NUAK1	protein_coding
0,8992483	DPP8	protein_coding
0,6988495	ZNF532	protein_coding
0,9245441	LMAN1	protein_coding
0,5095965	HACD3	protein_coding
0,9771336	ZZEF1	protein_coding
0,9917279	ENO1	protein_coding
0,9808899	MYDGF	protein_coding
0,6761449	TUBE1	protein_coding
0,7352523	ARHGEF10L	protein_coding
0,7694456	ACTR6	protein_coding
0,8117718	SRI	protein_coding
0,9647765	EIF4G3	protein_coding
0,5134965	SEMA3C	protein_coding
0,8790229	TTC38	protein_coding
0,738173	ACAT1	protein_coding
0,9558655	GRAMD4	protein_coding

0,5117223	CELSR1	protein_coding
0,8868311	ZNF638	protein_coding
0,7364979	SLC25A40	protein_coding
0,1582806	TIMM21	protein_coding
0,5879554	RASAL2	protein_coding
0,7842247	ZNF37A	protein_coding
0,4835164	MARK3	protein_coding
0,2179973	SLC25A3	protein_coding
0,9484395	FNDC3B	protein_coding
0,9544158	FOSL2	protein_coding
0,6999614	FRYL	protein_coding
0,9632674	TMEM131	protein_coding
0,8373584	ACTB	protein_coding
0,5364552	PLD1	protein_coding
0,7921707	DLG1	protein_coding
0,9998478	RAB7A	protein_coding
0,5347705	BCAP29	protein_coding
0,8259599	SEC31B	protein_coding
0,4161706	SART3	protein_coding
0,90504	ARHGAP15	protein_coding
0,9644268	EXOSC7	protein_coding
0,9125449	KIFAP3	protein_coding
0,9357394	MKRN2	protein_coding
0,8114908	MCM6	protein_coding
0,5400662	REXO2	protein_coding
0,9182782	RBM7	protein_coding
0,8189129	RBMS2	protein_coding
0,8189129	BAZ2A	protein_coding
0,5688668	MLH1	protein_coding
0,4045325	UNG	protein_coding
0,6591379	KLHL20	protein_coding
0,8992483	PLXNA2	protein_coding
0,7118106	ANKRD13A	protein_coding
0,8992483	TPD52	protein_coding
0,7816267	ACACB	protein_coding
0,284683	PAG1	protein_coding
0,8221205	NT5C2	protein_coding
0,9374738	MCAM	protein_coding
0,7390284	GPC4	protein_coding
0,7736716	MBNL3	protein_coding
0,9808899	NMRK2	protein_coding
0,8882787	DGKD	protein_coding
0,3820553	CTTNBP2	protein_coding
0,4519396	RARB	protein_coding
0,9900268	TOP2B	protein_coding
0,9880046	TM9SF3	protein_coding
0,9428271	PPP1R12B	protein_coding
0,9953531	DNAJC10	protein_coding
0,8209243	GTF3C1	protein_coding
0,71754	IL4R	protein_coding
0,9616434	USP33	protein_coding
0,6896707	DYNC1I2	protein_coding
0,9998538	FAM76B	protein_coding
0,9982092	POLD3	protein_coding
0,993752	ACTN2	protein_coding
0,8992483	CAPZB	protein_coding
0,4593782	GPR137B	protein_coding
0,8229361	JADE1	protein_coding
0,7774855	UBE2A	protein_coding

0,9808899	FGFR1	protein_coding
0,9081173	FBLN1	protein_coding
0,5664861	ITGA8	protein_coding
0,62553	MAP2	protein_coding
0,804648	PIAS2	protein_coding
0,2330928	AMPH	protein_coding
0,727014	ARAF	protein_coding
0,6563172	MCCC1	protein_coding
0,8021477	NEBL	protein_coding
0,9928407	ACER3	protein_coding
0,768388	UBE2K	protein_coding
0,997931	PIK3C3	protein_coding
0,7154026	N4BP2	protein_coding
0,4160411	TULP3	protein_coding
0,330331	SYNJ2	protein_coding
0,9773277	PPP2R5C	protein_coding
0,4541226	RBFOX1	protein_coding
0,8155634	GNB1	protein_coding
0,9967216	MLLT10	protein_coding
0,8301309	ITM2A	protein_coding
0,2281984	NRDC	protein_coding
0,3653635	VDAC3	protein_coding
0,8840514	PCM1	protein_coding
0,7558125	TNRC6C	protein_coding
0,6867028	CBFA2T2	protein_coding
0,9672665	ITCH	protein_coding
0,7235397	TP53INP2	protein_coding
0,855954	SDF4	protein_coding
0,645515	MYH7B	protein_coding
0,5140003	UBE2D4	protein_coding
0,9439974	RUNX1T1	protein_coding
0,7359516	THOC1	protein_coding
0,9914435	FKBP7	protein_coding
0,6762366	OSBPL6	protein_coding
0,0727176	SLC1A3	protein_coding
0,855954	XRCC5	protein_coding
0,6184746	MKNK1	protein_coding
0,9181578	TNS1	protein_coding
0,9494633	SAR1A	protein_coding
0,7440219	CDC14A	protein_coding
0,7456311	RAPGEF3	protein_coding
0,8386162	SENP1	protein_coding
0,5462026	LIPE	protein_coding
0,7836389	FDFT1	protein_coding
0,3516664	OPHN1	protein_coding
0,2321036	CARMIL1	protein_coding
0,4314718	PGM1	protein_coding
0,5625279	DDX1	protein_coding
0,7219856	DNM2	protein_coding
0,4450378	EPB41L2	protein_coding
0,9540553	STX7	protein_coding
0,9840915	KEAP1	protein_coding
0,7558125	SLC35C2	protein_coding
0,9587213	CRYBG3	protein_coding
0,9496064	RFX3	protein_coding
0,9933219	RIF1	protein_coding
0,9766993	RAB21	protein_coding
0,4344504	SLC4A4	protein_coding
0,4818782	SMARCA2	protein_coding

0,3398681	SESN1	protein_coding
0,6491869	COL5A3	protein_coding
0,7321942	PUM3	protein_coding
0,0125259	KCNN2	protein_coding
0,9233889	CNOT4	protein_coding
0,9771336	PSEN1	protein_coding
0,4607593	CLDND1	protein_coding
0,9248086	MOK	protein_coding
0,6244656	HSP90AA1	protein_coding
0,864196	RBL1	protein_coding
0,5622112	DLGAP4	protein_coding
0,887319	IGSF9B	protein_coding
0,8951112	AP4E1	protein_coding
0,7842247	RSBN1	protein_coding
0,6463814	MAGI3	protein_coding
0,579213	CXCL2	protein_coding
0,8823913	COL4A4	protein_coding
0,5209997	OSTM1	protein_coding
0,9496064	PCNP	protein_coding
0,9917279	EXD2	protein_coding
0,9127443	MEF2C	protein_coding
0,9112805	PTPRC	protein_coding
0,59951	UBA5	protein_coding
0,5709725	STK17B	protein_coding
0,8559418	CDC14B	protein_coding
0,931978	ZNF510	protein_coding
0,9616301	ZNF506	protein_coding
0,1444307	AACS	protein_coding
0,6816598	DELE1	protein_coding
0,0317466	CADPS2	protein_coding
0,6060734	HSPB11	protein_coding
0,9781971	PHLPP1	protein_coding
0,99743	ATP8B1	protein_coding
0,4264135	SMARCD3	protein_coding
0,9894339	WDR70	protein_coding
0,8834615	FYB1	protein_coding
0,998169	STRADB	protein_coding
0,8992483	BZW1	protein_coding
0,7154506	PGR	protein_coding
0,1933793	ME2	protein_coding
0,7320618	C5orf22	protein_coding
0,8554227	CCNT2	protein_coding
0,7213265	FAM135A	protein_coding
0,1801696	EPB41L3	protein_coding
0,4818782	COBLL1	protein_coding
0,7589872	DLG3	protein_coding
0,1350446	SERTAD4	protein_coding
0,7019864	TRAF5	protein_coding
0,252949	MRPL22	protein_coding
0,993752	GEMIN5	protein_coding
0,9362475	NFE2L1	protein_coding
0,9840915	GSK3B	protein_coding
0,0959391	ITGB5	protein_coding
0,9233889	ERC1	protein_coding
0,8091803	XPO1	protein_coding
0,9173787	RNF13	protein_coding
0,9533676	PALB2	protein_coding
0,8219774	DOP1A	protein_coding
0,3881918	LYRM2	protein_coding

0,9973317	BCKDHB	protein_coding
0,9977992	KAT6A	protein_coding
0,9689977	TUT7	protein_coding
0,0385201	ULK2	protein_coding
0,9998478	TNPO1	protein_coding
0,9341044	PLOD1	protein_coding
0,887319	DIS3	protein_coding
0,9997039	PIBF1	protein_coding
0,7558125	TDRD3	protein_coding
0,9112805	PDS5B	protein_coding
0,8238202	OXCT1	protein_coding
0,7956173	RRAGB	protein_coding
0,2486339	CYLD	protein_coding
0,997931	ZNF671	protein_coding
0,9951314	ZNF586	protein_coding
0,3513961	ZNF264	protein_coding
0,827821	RPS5	protein_coding
0,7688687	FAT1	protein_coding
0,4718	YTHDC1	protein_coding
0,9738194	CHMP2B	protein_coding
0,9700303	SMAP2	protein_coding
0,5869872	PPIE	protein_coding
0,330331	ZMPSTE24	protein_coding
0,284683	STARD7	protein_coding
0,947809	NOA1	protein_coding
0,4456365	REST	protein_coding
0,7321942	SSH1	protein_coding
0,9363291	GSTP1	protein_coding
0,9900663	APLP2	protein_coding
0,6425736	WBP11	protein_coding
0,7296863	EIF3I	protein_coding
0,1856054	COL16A1	protein_coding
0,8575378	TXLNA	protein_coding
0,654521	APOB	protein_coding
0,6854132	NCOA1	protein_coding
0,8269408	AGBL5	protein_coding
0,5723744	EFR3B	protein_coding
0,4519396	RAB10	protein_coding
0,54953	HADHA	protein_coding
0,5455384	MAPRE3	protein_coding
0,5444622	CAD	protein_coding
0,0390866	CD59	protein_coding
0,8992483	ATRX	protein_coding
0,7114715	AK6	protein_coding
0,7558125	MYNN	protein_coding
0,3316892	MECOM	protein_coding
0,7558125	SCAMP1	protein_coding
0,8850069	PREP	protein_coding
0,9118315	HACE1	protein_coding
0,930757	SEH1L	protein_coding
0,9953531	WDR47	protein_coding
0,6438808	WDFY1	protein_coding
0,6511505	SLC25A24	protein_coding
0,623981	MAP3K4	protein_coding
0,0385201	AKR1B1	protein_coding
0,6689227	CPNE3	protein_coding
0,5170218	RRN3	protein_coding
0,191133	CTTN	protein_coding
0,284683	MTIF2	protein_coding

0,7118106	DDHD2	protein_coding
0,3803906	TTC39A	protein_coding
0,9768456	EPS15	protein_coding
0,5313262	MGST2	protein_coding
0,9625694	ATG16L1	protein_coding
0,9496064	USP40	protein_coding
0,8714772	POMGNT1	protein_coding
0,6319662	MAST2	protein_coding
0,933129	DNAJA1	protein_coding
0,8698319	B4GALT1	protein_coding
0,9544158	CHMP5	protein_coding
0,9233889	NFX1	protein_coding
0,9962215	DIMT1	protein_coding
0,9953531	IPO11	protein_coding
0,8417824	EIF2AK1	protein_coding
0,7571978	EPDR1	protein_coding
0,997931	SEPHS1	protein_coding
0,6303013	ITPKC	protein_coding
0,4806087	RBM22	protein_coding
0,9709611	TMED2	protein_coding
0,8992483	ERO1B	protein_coding
0,7687197	ZFAND6	protein_coding
0,9233889	TXLNG	protein_coding
0,7936863	HUWE1	protein_coding
0,692146	ZW10	protein_coding
0,9173787	ALG9	protein_coding
0,0941174	NOX4	protein_coding
0,4826313	ACOX3	protein_coding
0,7972313	MTMR2	protein_coding
0,5175383	PPP1R15A	protein_coding
0,6613295	FTL	protein_coding
0,931978	SRRT	protein_coding
0,5560269	NLK	protein_coding
0,5343413	PIGS	protein_coding
0,5011485	ADAMTS2	protein_coding
0,8422988	ATXN7L3	protein_coding
0,4894579	PGS1	protein_coding
0,6448972	PSMC5	protein_coding
0,9350074	UIMC1	protein_coding
0,5140003	MMP2	protein_coding
0,8887908	LPCAT2	protein_coding
0,7558125	GNAO1	protein_coding
0,8495694	OGFOD1	protein_coding
0,7558125	SH3BP2	protein_coding
0,9621537	NOP14	protein_coding
0,9127443	ADD1	protein_coding
0,4957019	L2HGDH	protein_coding
0,4179007	TXNDC16	protein_coding
0,3459301	RTRAF	protein_coding
0,8381234	NID2	protein_coding
0,5073158	GMCL1	protein_coding
0,7854018	SF3B2	protein_coding
0,9439974	KLHL42	protein_coding
0,9178514	GNAS	protein_coding
0,5347705	DNM1L	protein_coding
0,5675917	ERGIC2	protein_coding
0,289067	AAMDC	protein_coding
0,7748443	METTL2A	protein_coding
0,975657	PTPN4	protein_coding

0,9702772	DDX18	protein_coding
0,6277796	KHSRP	protein_coding
0,9954421	GNA11	protein_coding
0,6701082	ASAP3	protein_coding
0,9296784	EDEM2	protein_coding
0,54953	EPB41L1	protein_coding
0,8745367	DOCK9	protein_coding
0,9860605	ANKRD10	protein_coding
0,9967216	DOCK3	protein_coding
0,2419676	C3orf18	protein_coding
0,4971845	COQ9	protein_coding
0,5474737	PPP1R13B	protein_coding
0,7230802	ATRN	protein_coding
0,1709741	SIGLEC1	protein_coding
0,6254273	FKBP1A	protein_coding
0,8512635	NSFL1C	protein_coding
0,6887436	C20orf194	protein_coding
0,9962215	MAVS	protein_coding
0,5267449	XRN2	protein_coding
0,5485366	KIZ	protein_coding
0,9142106	DYNLL1	protein_coding
0,4750209	SNX5	protein_coding
0,9484395	RPL6	protein_coding
0,9174724	MAPKAPK5	protein_coding
0,9598334	ESF1	protein_coding
0,7444016	RBBP9	protein_coding
0,5590581	ANAPC5	protein_coding
0,5393446	SLC23A2	protein_coding
0,5248109	SLC8B1	protein_coding
0,827821	TMEM230	protein_coding
0,4765298	TASP1	protein_coding
0,7844757	GCN1	protein_coding
0,9678404	RPLP0	protein_coding
0,8238032	PXN	protein_coding
0,6988495	KIF16B	protein_coding
0,7122541	TRMT6	protein_coding
0,6287358	CHGB	protein_coding
0,6574161	PEBP1	protein_coding
0,8941532	TBX5	protein_coding
0,841851	BRAP	protein_coding
0,9765371	ERP29	protein_coding
0,8594992	FUS	protein_coding
0,9840915	IGBP1	protein_coding
0,7114949	ZNF302	protein_coding
0,5114885	HEPH	protein_coding
0,7154026	CDIP1	protein_coding
0,6762366	GANAB	protein_coding
0,9481776	RBM41	protein_coding
0,6383874	MLF2	protein_coding
0,9169269	DDX24	protein_coding
0,6053522	ZBTB25	protein_coding
0,9838551	NECAP1	protein_coding
0,5777776	DHX32	protein_coding
0,9998538	RCOR1	protein_coding
0,9802452	GPATCH2L	protein_coding
0,2945575	LTBP4	protein_coding
0,7611529	BLVRB	protein_coding
0,8698319	SPTLC1	protein_coding
0,7029174	PAPOLA	protein_coding

0,9589961	PCBP4	protein_coding
0,1431227	MRPS33	protein_coding
0,1559069	NDUFB2	protein_coding
0,5265966	NUDC	protein_coding
0,8992483	MAEA	protein_coding
0,887128	ICAM1	protein_coding
0,8721281	STRN4	protein_coding
0,9484395	IRAK3	protein_coding
0,9142106	LYZ	protein_coding
0,5510556	MUL1	protein_coding
0,5440543	PDCD7	protein_coding
0,8721281	SPG21	protein_coding
0,8823583	DNAJB11	protein_coding
0,2354584	RAB11FIP3	protein_coding
0,933129	ZNF268	protein_coding
0,3194617	GOLGA3	protein_coding
0,8885864	PABPC4	protein_coding
0,0247382	CD209	protein_coding
0,8698323	USP48	protein_coding
0,9900815	EFNB1	protein_coding
0,6604792	PDPR	protein_coding
0,8698319	AARS	protein_coding
0,6613295	GLG1	protein_coding
0,9997039	TNRC6A	protein_coding
0,6785825	EXOC1	protein_coding
0,8523001	RBM27	protein_coding
0,8847411	OSBPL8	protein_coding
0,7201269	LAMB1	protein_coding
0,5024248	DLD	protein_coding
0,7834554	WDR7	protein_coding
0,2506935	TXNL1	protein_coding
0,8318664	CMTM6	protein_coding
0,4115954	ITGA6	protein_coding
0,8834615	RAPGEF4	protein_coding
0,9781971	MAP3K20	protein_coding
0,2318378	SMPX	protein_coding
0,3954735	FH	protein_coding
0,9142106	TF	protein_coding
0,8436853	CDV3	protein_coding
0,7954765	ALKBH5	protein_coding
0,330331	NLRP1	protein_coding
0,5263704	SPAG7	protein_coding
0,2443691	ZFHX4	protein_coding
0,6160654	ZC3HC1	protein_coding
0,3679769	ESR1	protein_coding
0,7904545	ANGPT2	protein_coding
0,4031526	CD200	protein_coding
0,5879554	CCDC80	protein_coding
0,6607313	PSME1	protein_coding
0,738122	PPP2R3C	protein_coding
0,4151093	MYH7	protein_coding
0,6173428	OSGEP	protein_coding
0,8725749	SCFD1	protein_coding
0,9194118	G2E3	protein_coding
0,947809	HECTD1	protein_coding
0,9827285	HNRNPC	protein_coding
0,3881918	SUPT16H	protein_coding
0,9067454	TOX4	protein_coding
0,9248086	TINF2	protein_coding

0,3803906	SEMA6A	protein_coding
0,9998478	TRPM7	protein_coding
0,997931	SNAP23	protein_coding
0,307685	PHGDH	protein_coding
0,4179007	EZR	protein_coding
0,8238032	MYL6	protein_coding
0,8545769	AGO1	protein_coding
0,6184079	RFFL	protein_coding
0,4806087	MFSD11	protein_coding
0,9233889	DPYSL2	protein_coding
0,8930787	GPATCH2	protein_coding
0,5402971	NUP50	protein_coding
0,9768456	COMT	protein_coding
0,8422988	ADA2	protein_coding
0,8318519	ECHDC1	protein_coding
0,7673209	LRRFIP2	protein_coding
0,7154026	SEC22C	protein_coding
0,7318327	HDAC6	protein_coding
0,8908494	UPRT	protein_coding
0,9432348	CDC23	protein_coding
0,9296784	AAAS	protein_coding
0,6220194	CBX5	protein_coding
0,2283663	FMO2	protein_coding
0,9781971	SUCO	protein_coding
0,9724163	MSH2	protein_coding
0,9253623	MAP3K1	protein_coding
0,768388	HOOK2	protein_coding
0,4137636	ARCN1	protein_coding
0,8373584	EPB41L4B	protein_coding
0,2250724	TMEM38B	protein_coding
0,9358987	PSMD5	protein_coding
0,0845999	PTGS1	protein_coding
0,9077931	NUP188	protein_coding
0,8415978	CRAT	protein_coding
0,3694563	NANS	protein_coding
0,9978259	CWF19L1	protein_coding
0,8318664	BTAF1	protein_coding
0,8301309	IKZF5	protein_coding
0,9827285	SORBS1	protein_coding
0,2834613	BAMBI	protein_coding
0,9977992	WAC	protein_coding
0,5799008	CREM	protein_coding
0,9578517	HIVEP1	protein_coding
0,4160411	FKBP5	protein_coding
0,54642	SRPK1	protein_coding
0,997931	BRPF3	protein_coding
0,3549138	EFHC1	protein_coding
0,7243515	HSP90AB1	protein_coding
0,9481776	CDC5L	protein_coding
0,9947212	ITPR3	protein_coding
0,7352523	DSP	protein_coding
0,5972245	SIRT1	protein_coding
0,9603194	HNRNPH3	protein_coding
0,8500846	IFT74	protein_coding
0,730414	JAK2	protein_coding
0,6206631	ABL1	protein_coding
0,9647765	SH3GLB1	protein_coding
0,8287149	SYDE2	protein_coding
0,5846553	PCSK5	protein_coding

0,931978	SCD	protein_coding
0,7714588	ABLIM1	protein_coding
0,7468218	ERMP1	protein_coding
0,7743102	RAB18	protein_coding
0,8117718	NRP1	protein_coding
0,2169157	PALMD	protein_coding
0,316005	WASHC2A	protein_coding
0,9017378	MYO9B	protein_coding
0,9386808	PSMD8	protein_coding
0,8176511	CIRBP	protein_coding
0,7611852	PRKY	protein_coding
0,9484395	HNRNPM	protein_coding
0,9463928	MARCH2	protein_coding
0,809839	NDUFB7	protein_coding
0,6563172	TECR	protein_coding
0,9952606	MTAP	protein_coding
0,9424246	POLR2E	protein_coding
0,9333994	GADD45B	protein_coding
0,855954	MKNK2	protein_coding
0,8489317	RANBP1	protein_coding
0,9377639	KLHL22	protein_coding
0,9709611	MED15	protein_coding
0,8865445	SNAP29	protein_coding
0,9424246	CRKL	protein_coding
0,8285832	CECR2	protein_coding
0,730414	SMARCB1	protein_coding
0,6229784	BCL2L13	protein_coding
0,8909428	CABIN1	protein_coding
0,8422988	SF3A1	protein_coding
0,5074563	SPECC1L	protein_coding
0,887319	PPIL2	protein_coding
0,9237476	SNRPD3	protein_coding
0,997923	PES1	protein_coding
0,4835164	MAPK1	protein_coding
0,6134356	PPM1F	protein_coding
0,7175278	CYTH4	protein_coding
0,3912252	GRK3	protein_coding
0,8603095	GGA1	protein_coding
0,9824231	LGALS1	protein_coding
0,9377639	HPS4	protein_coding
0,166273	PIK3IP1	protein_coding
0,6169817	SRRD	protein_coding
0,2778507	PATZ1	protein_coding
0,9801969	TRIOBP	protein_coding
0,855954	TFIP11	protein_coding
0,5212854	EIF3L	protein_coding
0,3382498	SNU13	protein_coding
0,6766513	TTC28	protein_coding
0,397269	SLC5A1	protein_coding
0,0727176	KDELR3	protein_coding
0,9074092	DDX17	protein_coding
0,81614	TCF20	protein_coding
0,9081173	CBY1	protein_coding
0,633922	TOMM22	protein_coding
0,887319	XBP1	protein_coding
0,9316489	RTCB	protein_coding
0,4246004	JOSD1	protein_coding
0,5249547	FBXO7	protein_coding
0,6408377	GTPBP1	protein_coding

0,7835684	POLDIP3	protein_coding
0,997931	TIMP3	protein_coding
0,7437136	PPP6R2	protein_coding
0,6611168	SBF1	protein_coding
0,995098	SUN2	protein_coding
0,9450135	CYB5R3	protein_coding
0,8992483	PACSIN2	protein_coding
0,9954421	AP1B1	protein_coding
0,9215449	HMGXB4	protein_coding
0,891947	TOM1	protein_coding
0,3078991	HMOX1	protein_coding
0,3697716	THOC5	protein_coding
0,9998478	TSPO	protein_coding
0,7542355	CBX7	protein_coding
0,5723744	RPL3	protein_coding
0,9240289	RBFOX2	protein_coding
0,6776989	SYNGR1	protein_coding
0,8500846	ASCC2	protein_coding
0,4321437	MTMR3	protein_coding
0,7740102	MIEF1	protein_coding
0,9919804	APOL1	protein_coding
0,8578793	MYH9	protein_coding
0,2533818	SAMM50	protein_coding
0,5707233	TXN2	protein_coding
0,8861906	EIF3D	protein_coding
0,7956789	TNRC6B	protein_coding
0,7713242	IFT27	protein_coding
0,9298036	KIAA0930	protein_coding
0,8238032	FAM118A	protein_coding
0,7357059	ST13	protein_coding
0,5985056	RBX1	protein_coding
0,7743102	EP300	protein_coding
0,9703483	L3MBTL2	protein_coding
0,9464798	RANGAP1	protein_coding
0,5771952	ZC3H7B	protein_coding
0,6168923	PHF5A	protein_coding
0,933129	ACO2	protein_coding
0,4999985	TRMU	protein_coding
0,0904915	DESI1	protein_coding
0,6285359	CERK	protein_coding
0,7558125	BRD1	protein_coding
0,8887908	ZBED4	protein_coding
0,9941887	ABHD4	protein_coding
0,7528498	KHNYN	protein_coding
0,0796818	FKBP3	protein_coding
0,9484395	RBM23	protein_coding
0,9982092	PRMT5	protein_coding
0,8301103	AP4S1	protein_coding
0,6762366	SOS2	protein_coding
0,9917279	NIN	protein_coding
0,779252	PYGL	protein_coding
0,5212854	PSMC6	protein_coding
0,8364227	DDHD1	protein_coding
0,8992483	CNIH1	protein_coding
0,6878093	CGRRF1	protein_coding
0,5187247	ATP6V1D	protein_coding
0,0868691	PSMA3	protein_coding
0,7935149	VTI1B	protein_coding
0,9282793	KIAA0586	protein_coding

0,54642	TMED8	protein_coding
0,4246004	AHSA1	protein_coding
0,9726433	DAAM1	protein_coding
0,9953531	SPTLC2	protein_coding
0,4498511	LGMN	protein_coding
0,8824496	SNW1	protein_coding
0,8745989	ITPK1	protein_coding
0,4684923	DHRS7	protein_coding
0,7968733	PPM1A	protein_coding
0,3912252	GALNT16	protein_coding
0,3607643	ASB2	protein_coding
0,7713242	ERH	protein_coding
0,9933219	HIF1A	protein_coding
0,8155634	SUSD6	protein_coding
0,9535559	SRSF5	protein_coding
0,892596	EIF5	protein_coding
0,8436853	DICER1	protein_coding
0,4835164	ZFYVE21	protein_coding
0,6330107	MTHFD1	protein_coding
0,997931	ZC3H14	protein_coding
0,855954	PCNX1	protein_coding
0,8319167	GSKIP	protein_coding
0,9925374	VRK1	protein_coding
0,5531846	PSMC1	protein_coding
0,6238595	RPS6KA5	protein_coding
0,9583708	PPP4R3A	protein_coding
0,3089259	PSMB5	protein_coding
0,7437571	YY1	protein_coding
0,9745853	ACIN1	protein_coding
0,3459301	CCNB1IP1	protein_coding
0,9481776	TRIP11	protein_coding
0,8992483	APEX1	protein_coding
0,997931	PABPN1	protein_coding
0,7118106	ARHGAP5	protein_coding
0,6499951	CINP	protein_coding
0,8373584	SRP54	protein_coding
0,7885463	CHD8	protein_coding
0,8885864	DCAF11	protein_coding
0,4835164	NFKBIA	protein_coding
0,8269408	PSME2	protein_coding
0,6238595	BRMS1L	protein_coding
0,5851976	SEC23A	protein_coding
0,7956173	GMPR2	protein_coding
0,9113537	PNN	protein_coding
0,6146798	NFATC4	protein_coding
0,077831	PLTP	protein_coding
0,99611	GSS	protein_coding
0,8871625	TRPC4AP	protein_coding
0,5977377	PYGB	protein_coding
0,9808899	ABHD12	protein_coding
0,5881683	PROCR	protein_coding
0,5029234	UQCC1	protein_coding
0,613355	ZMYND8	protein_coding
0,3122511	IFT52	protein_coding
0,8875885	NDRG3	protein_coding
0,3961322	NFATC2	protein_coding
0,5911872	STK4	protein_coding
0,2321036	ADNP	protein_coding
0,5293665	PFDN4	protein_coding

0,1729437	DOK5	protein_coding
0,8062191	CSTF1	protein_coding
0,9406097	RAE1	protein_coding
0,4153382	TPD52L2	protein_coding
0,9282881	DNAJC5	protein_coding
0,855954	NELFCD	protein_coding
0,7122873	CTSZ	protein_coding
0,4806087	PRPF6	protein_coding
0,7313486	PRELID3B	protein_coding
0,3880092	PSMA7	protein_coding
0,7367498	DIDO1	protein_coding
0,3814605	GID8	protein_coding
0,9789623	EEF1A2	protein_coding
0,6463078	RNF24	protein_coding
0,824287	ARFRP1	protein_coding
0,2437974	NDUFAF5	protein_coding
0,8525767	RASSF2	protein_coding
0,8582079	CSNK2A1	protein_coding
0,9420326	CDS2	protein_coding
0,8939634	HM13	protein_coding
0,9120573	SEC23B	protein_coding
0,5768067	PLCB4	protein_coding
0,7558577	MYL9	protein_coding
0,8721867	TM9SF4	protein_coding
0,9914435	CRNKL1	protein_coding
0,6574161	POFUT1	protein_coding
0,2908312	SAMHD1	protein_coding
0,2250724	KIF3B	protein_coding
0,6491869	NOP56	protein_coding
0,2841212	IDH3B	protein_coding
0,624851	MAPRE1	protein_coding
0,9484395	JAG1	protein_coding
0,4835164	CDK5RAP1	protein_coding
0,5225448	SNTA1	protein_coding
0,7244173	TTI1	protein_coding
0,4965036	RPRD1B	protein_coding
0,9165105	PXMP4	protein_coding
0,6620763	CHMP4B	protein_coding
0,7213265	CST3	protein_coding
0,9817008	AHCY	protein_coding
0,2945575	PPP1R16B	protein_coding
0,9962215	DHX35	protein_coding
0,9827285	SYNDIG1	protein_coding
0,615864	APMAP	protein_coding
0,7542355	ZNF516	protein_coding
0,5402971	ADNP2	protein_coding
0,997931	USP14	protein_coding
0,8877092	VAPA	protein_coding
0,9609096	METTL4	protein_coding
0,7972313	LPIN2	protein_coding
0,9914435	SMCHD1	protein_coding
0,9082846	MYOM1	protein_coding
0,9781971	MYL12A	protein_coding
0,8992483	CEP192	protein_coding
0,8422988	RNMT	protein_coding
0,0811214	SMAD7	protein_coding
0,9544158	ANKRD12	protein_coding
0,6676625	POLI	protein_coding
0,7723854	MIB1	protein_coding

0,9508384	RBBP8	protein_coding
0,806905	RIOK3	protein_coding
0,2026798	MXRA5	protein_coding
0,9922688	PSMD10	protein_coding
0,3178493	ATG4A	protein_coding
0,472118	STS	protein_coding
0,8189129	TBL1X	protein_coding
0,8832193	PGRMC1	protein_coding
0,9666028	POLA1	protein_coding
0,5123978	MID1	protein_coding
0,9754097	NKAP	protein_coding
0,6613295	ATP1B4	protein_coding
0,6880486	ALG13	protein_coding
0,8301309	MOSPD1	protein_coding
0,613355	AMMECR1	protein_coding
0,4894579	CHRDL1	protein_coding
0,9846914	WDR13	protein_coding
0,2948143	SRPX	protein_coding
0,864026	XIAP	protein_coding
0,9793092	STAG2	protein_coding
0,9967216	ATP11C	protein_coding
0,7154026	PLP2	protein_coding
0,6491869	PLS3	protein_coding
0,284683	SMARCA1	protein_coding
0,9270127	RBBP7	protein_coding
0,4638153	FMR1	protein_coding
0,5558351	TAZ	protein_coding
0,3358222	PGK1	protein_coding
0,8345564	MAGT1	protein_coding
0,6456962	SMS	protein_coding
0,6761449	UBL4A	protein_coding
0,9709611	CD99L2	protein_coding
0,7125689	EEA1	protein_coding
0,77251	RP2	protein_coding
0,6053653	CDK16	protein_coding
0,1574812	USP11	protein_coding
0,8918196	HTATSF1	protein_coding
0,4314663	TIMP1	protein_coding
0,9998478	GABRE	protein_coding
0,5567171	PIN4	protein_coding
0,5919274	MAGED2	protein_coding
0,6835435	RBM3	protein_coding
0,9933479	KLF8	protein_coding
0,3699797	SRPX2	protein_coding
0,3030424	GLA	protein_coding
0,3513961	ARMCX3	protein_coding
0,902171	BEX4	protein_coding
0,6199383	NALCN	protein_coding
0,9970518	NDFIP2	protein_coding
0,6611168	FNDC3A	protein_coding
0,6494449	CAB39L	protein_coding
0,9700639	STK24	protein_coding
0,8500846	DNAJC3	protein_coding
0,7242009	UGGT2	protein_coding
0,7842247	ARHGEF7	protein_coding
0,2073391	FGF9	protein_coding
0,8786805	SGCG	protein_coding
0,5669789	PARP4	protein_coding
0,9142106	SUPT20H	protein_coding

0,3309973	MRPS31	protein_coding
0,6418915	KPNA3	protein_coding
0,504498	FLT1	protein_coding
0,9917279	RGCC	protein_coding
0,9794449	VWA8	protein_coding
0,7658228	DGKH	protein_coding
0,9254043	KATNAL1	protein_coding
0,933129	INTS6	protein_coding
0,9939034	MEDAG	protein_coding
0,5015091	TSC22D1	protein_coding
0,7968733	CLN5	protein_coding
0,9666864	ZNF629	protein_coding
0,7948949	MAPK3	protein_coding
0,9233889	PHKB	protein_coding
0,5318944	LYRM1	protein_coding
0,9366634	NUTF2	protein_coding
0,2945575	NUP93	protein_coding
0,8704958	NFAT5	protein_coding
0,9737459	LONP2	protein_coding
0,8683086	N4BP1	protein_coding
0,5869872	DHODH	protein_coding
0,6613295	CTCF	protein_coding
0,7560747	POLR2C	protein_coding
0,6093605	MMP15	protein_coding
0,5111636	CYB5B	protein_coding
0,806856	NDRG4	protein_coding
0,1431468	PSMD7	protein_coding
0,9997039	SLC38A7	protein_coding
0,8557332	TANGO6	protein_coding
0,7740102	COG4	protein_coding
0,7135335	SLC7A6	protein_coding
0,4344504	PLA2G15	protein_coding
0,9998538	WDR59	protein_coding
0,9908068	MON1B	protein_coding
0,105104	CMC2	protein_coding
0,8912998	MLYCD	protein_coding
0,0639504	HSDL1	protein_coding
0,5337323	USP10	protein_coding
0,0026029	CRISPLD2	protein_coding
0,5752656	TSC2	protein_coding
0,6873469	ABCC1	protein_coding
0,7968733	NOMO3	protein_coding
0,8545769	FBXO31	protein_coding
0,9237476	UBE2I	protein_coding
0,4532937	CRYM	protein_coding
0,9424246	EEF2K	protein_coding
0,9998478	PIEZO1	protein_coding
0,7062909	GSPT1	protein_coding
0,3765276	CLUAP1	protein_coding
0,9358987	UBFD1	protein_coding
0,9944531	EARS2	protein_coding
0,9081173	ELOB	protein_coding
0,997931	GGA2	protein_coding
0,4161706	CPPED1	protein_coding
0,7246609	USP31	protein_coding
0,0640162	HMOX2	protein_coding
0,747138	DNAJA3	protein_coding
0,9343701	BFAR	protein_coding
0,5485366	RBL2	protein_coding

0,5245497	QPRT	protein_coding
0,6613295	XYLT1	protein_coding
0,7912174	STX4	protein_coding
0,9773277	CDIPT	protein_coding
0,5139593	BCKDK	protein_coding
0,9296784	KAT8	protein_coding
0,8931895	NOMO1	protein_coding
0,6511285	CCP110	protein_coding
0,6781085	VPS35L	protein_coding
0,6197914	RNF40	protein_coding
0,5869872	AQP9	protein_coding
0,9508384	AAGAB	protein_coding
0,3792977	LACTB	protein_coding
0,981554	HERC1	protein_coding
0,9003006	TRIP4	protein_coding
0,8410587	MTFMT	protein_coding
0,144928	RASL12	protein_coding
0,2368601	RAB11A	protein_coding
0,6206631	CTSH	protein_coding
0,6053653	TTC23	protein_coding
0,7740102	CD276	protein_coding
0,7871131	FAH	protein_coding
0,8695843	HOMER2	protein_coding
0,953175	EHD4	protein_coding
0,9142507	TMEM87A	protein_coding
0,997931	ZNF106	protein_coding
0,7444016	CEP152	protein_coding
0,7190863	ATP8B4	protein_coding
0,9463928	DTWD1	protein_coding
0,997931	GABPB1	protein_coding
0,446175	TJP1	protein_coding
0,9536474	DMXL2	protein_coding
0,7611852	EIF3J	protein_coding
0,8575378	SPG11	protein_coding
0,808509	VPS18	protein_coding
0,5558351	SLC30A4	protein_coding
0,8545769	BLOC1S6	protein_coding
0,8118149	MYEF2	protein_coding
0,8213028	SGK3	protein_coding
0,1444307	PDGFRL	protein_coding
0,9215449	CSPP1	protein_coding
0,8881504	ZDHHC2	protein_coding
0,8422988	ZFAND1	protein_coding
0,997931	FZD3	protein_coding
0,7158866	INTS9	protein_coding
0,7611529	NBN	protein_coding
0,8459353	CPQ	protein_coding
0,2068936	DECR1	protein_coding
0,9621856	IMPAD1	protein_coding
0,5709725	SFRP1	protein_coding
0,9928407	LAPTM4B	protein_coding
0,9536474	UBE2W	protein_coding
0,5224131	NIPAL2	protein_coding
0,9524483	IKBKB	protein_coding
0,624851	PLAT	protein_coding
0,6988495	JPH1	protein_coding
0,9458749	STK3	protein_coding
0,9982092	RAB2A	protein_coding
0,9977992	EIF3E	protein_coding

0,5647201	EMC2	protein_coding
0,8575378	NDRG1	protein_coding
0,922549	ZC2HC1A	protein_coding
0,6591549	ARMC1	protein_coding
0,6462349	TRPS1	protein_coding
0,7546576	CHRAC1	protein_coding
0,1800952	NCALD	protein_coding
0,9253623	SNX16	protein_coding
0,9725101	UBR5	protein_coding
0,9889321	EEF1D	protein_coding
0,9378903	INTS10	protein_coding
0,9900268	ERI1	protein_coding
0,1801696	SLC39A14	protein_coding
0,284683	MTMR9	protein_coding
0,8301309	LEPROTL1	protein_coding
0,5505806	DCTN6	protein_coding
0,8422988	R3HCC1	protein_coding
0,9998478	GSR	protein_coding
0,9465232	PPP2CB	protein_coding
0,4894579	TUSC3	protein_coding
0,4919121	MCM4	protein_coding
0,2146641	KCTD9	protein_coding
0,8786805	ASAH1	protein_coding
0,4911389	BNIP3L	protein_coding
0,8786805	MAN2B1	protein_coding
0,9771336	NUCB1	protein_coding
0,8991816	GYS1	protein_coding
0,7968733	ECH1	protein_coding
0,9081173	HNRNPL	protein_coding
0,6663173	SNRNP70	protein_coding
0,645515	CLPTM1	protein_coding
0,8116293	FCGRT	protein_coding
0,9975676	PIH1D1	protein_coding
0,3803906	CKM	protein_coding
0,6613295	PPP1R13L	protein_coding
0,7095541	OAZ1	protein_coding
0,8255851	DMPK	protein_coding
0,5537742	TLE5	protein_coding
0,9327336	SGTA	protein_coding
0,6591549	C19orf53	protein_coding
0,3697716	TIMM44	protein_coding
0,9808899	VRK3	protein_coding
0,891947	FAM32A	protein_coding
0,7934256	AKAP8	protein_coding
0,7835684	ILVBL	protein_coding
0,9766993	ZNF419	protein_coding
0,4954628	POP4	protein_coding
0,8919058	URI1	protein_coding
0,1916186	PDCD5	protein_coding
0,6184746	ANKRD27	protein_coding
0,8790123	RPS16	protein_coding
0,645515	TIMM50	protein_coding
0,8229361	FBL	protein_coding
0,8117718	GPI	protein_coding
0,8289966	AKT2	protein_coding
0,8782347	PLD3	protein_coding
0,8500846	TBCB	protein_coding
0,9296784	SLC1A5	protein_coding
0,8161522	HNRNPUL1	protein_coding

0,6261617	DENND3	protein_coding
0,4684923	DMAC2	protein_coding
0,8221205	MYH14	protein_coding
0,6888062	RPS19	protein_coding
0,5558351	NOP53	protein_coding
0,7940988	ETFB	protein_coding
0,9738194	TYK2	protein_coding
0,6140495	CDC37	protein_coding
0,8439769	NAPA	protein_coding
0,7296863	PTPRS	protein_coding
0,7894641	MEGF8	protein_coding
0,8138179	KDELR1	protein_coding
0,5094678	CYTH2	protein_coding
0,993752	CARD8	protein_coding
0,7716654	ZNF175	protein_coding
0,505053	PLA2G4C	protein_coding
0,9752796	BCAT2	protein_coding
0,8436853	PPP2R1A	protein_coding
0,5024483	TNPO2	protein_coding
0,0677818	LILRB5	protein_coding
0,6701082	RPL18A	protein_coding
0,3327196	TMEM147	protein_coding
0,7805908	USF2	protein_coding
0,9002008	KXD1	protein_coding
0,8665655	FKBP8	protein_coding
0,8137923	ZNF14	protein_coding
0,7661133	SCN1B	protein_coding
0,9700303	AVL9	protein_coding
0,702199	CFAP69	protein_coding
0,8711676	GTPBP10	protein_coding
0,5964345	RASA4	protein_coding
0,6037819	CDK6	protein_coding
0,7444016	PMPCB	protein_coding
0,6766513	DNAJC2	protein_coding
0,9484395	BET1	protein_coding
0,6333516	NAMPT	protein_coding
0,768388	TWISTNB	protein_coding
0,6003589	PIK3CG	protein_coding
0,3089797	PON2	protein_coding
0,4307688	ITGB8	protein_coding
0,887319	HBP1	protein_coding
0,9410184	SP4	protein_coding
0,9738194	DNAH11	protein_coding
0,8500846	CBLL1	protein_coding
0,8597029	MTPN	protein_coding
0,3694563	PTN	protein_coding
0,615864	MPP6	protein_coding
0,3194617	ZC3HAV1	protein_coding
0,7639764	OGDH	protein_coding
0,6138613	H2AFV	protein_coding
0,8189129	CAV2	protein_coding
0,5647201	CAV1	protein_coding
0,5552067	MET	protein_coding
0,3004311	LMBR1	protein_coding
0,9730672	DNAJB6	protein_coding
0,8875885	IQCE	protein_coding
0,6718649	TSPAN12	protein_coding
0,1888441	SSBP1	protein_coding
0,2512796	CPED1	protein_coding

0,4638153	HIBADH	protein_coding
0,5224131	TAX1BP1	protein_coding
0,9374881	CPVL	protein_coding
0,9998538	GRB10	protein_coding
0,4835164	COBL	protein_coding
0,9994633	FKBP14	protein_coding
0,7355144	PLEKHA8	protein_coding
0,7445906	NOD1	protein_coding
0,37039	GARS	protein_coding
0,9922409	CASP2	protein_coding
0,4593782	CHCHD2	protein_coding
0,8111375	HSPB1	protein_coding
0,8702296	PDAP1	protein_coding
0,9136442	BUD31	protein_coding
0,8992483	CYP3A5	protein_coding
0,4467509	ZKSCAN1	protein_coding
0,938611	EIF3B	protein_coding
0,8578793	WASL	protein_coding
0,458152	RBM28	protein_coding
0,4894579	LSM5	protein_coding
0,0908653	SERPINE1	protein_coding
0,654521	C1GALT1	protein_coding
0,9338281	PLOD3	protein_coding
0,8553175	RPA3	protein_coding
0,8160828	ZNHIT1	protein_coding
0,9951314	PHF14	protein_coding
0,7816527	NRF1	protein_coding
0,6785825	TMEM106B	protein_coding
0,5362266	ZNF862	protein_coding
0,654521	MEST	protein_coding
0,481646	MEOX2	protein_coding
0,7027821	ANKMY2	protein_coding
0,330331	TSPAN13	protein_coding
0,4959869	RARRES2	protein_coding
0,8992483	AHR	protein_coding
0,0560697	CHCHD3	protein_coding
0,7114715	TMEM176B	protein_coding
0,3327196	MRPL32	protein_coding
0,1431468	COA1	protein_coding
0,435547	BLVRA	protein_coding
0,5531846	URGCP	protein_coding
0,3803906	TMEM248	protein_coding
0,4835164	STAG3L4	transcribed_unprocessed_pseudogene
0,4344705	RHEB	protein_coding
0,9360339	PRKAG2	protein_coding
0,2908312	AEBP1	protein_coding
0,9628671	POLD2	protein_coding
0,5879554	MYL7	protein_coding
0,6563172	BCL7B	protein_coding
0,8622295	YKT6	protein_coding
0,4551386	TBL2	protein_coding
0,8924025	EIF4H	protein_coding
0,9771336	FKTN	protein_coding
0,9925374	FSD1L	protein_coding
0,3316892	CNTNAP3	protein_coding
0,5170218	SPIN1	protein_coding
0,5249842	NMRK1	protein_coding
0,7805908	TMEM245	protein_coding
0,7558125	PRUNE2	protein_coding

0,6904615	MEGF9	protein_coding
0,7032369	TGFBR1	protein_coding
0,9771336	SEC61B	protein_coding
0,284683	OGN	protein_coding
0,0796818	ASPN	protein_coding
0,1112135	ECM2	protein_coding
0,284683	TLE4	protein_coding
0,4808891	PTGR1	protein_coding
0,949756	SUSD1	protein_coding
0,7456311	AKNA	protein_coding
0,4638153	ENG	protein_coding
0,2146641	AK1	protein_coding
0,8823913	CDC37L1	protein_coding
0,1653531	PLGRKT	protein_coding
0,9781971	TBC1D13	protein_coding
0,9464798	RIC1	protein_coding
0,8118765	KDM4C	protein_coding
0,9205351	DOCK8	protein_coding
0,4806087	KANK1	protein_coding
0,4519396	FUBP3	protein_coding
0,7740102	TYRP1	protein_coding
0,9551307	CREB3	protein_coding
0,8252207	RGP1	protein_coding
0,6238595	MPDZ	protein_coding
0,8189129	DDX58	protein_coding
0,776038	EDF1	protein_coding
0,2146641	PIP5K1B	protein_coding
0,6425736	GLIS3	protein_coding
0,4835164	BAG1	protein_coding
0,7296863	RAPGEF1	protein_coding
0,9033043	SETX	protein_coding
0,8189129	PTGDS	protein_coding
0,6785825	ABCA2	protein_coding
0,8532945	UBE2R2	protein_coding
0,8500846	ABHD17B	protein_coding
0,9804708	EXOSC3	protein_coding
0,8172545	ZFAND5	protein_coding
0,6021768	PDLIM1	protein_coding
0,9248086	CCNJ	protein_coding
0,8067508	HPS1	protein_coding
0,8721867	PHYH	protein_coding
0,855954	RASSF4	protein_coding
0,9768456	DNMBP	protein_coding
0,9450135	RAB11FIP2	protein_coding
0,4806087	CXCL12	protein_coding
0,544397	ERLIN1	protein_coding
0,6511285	EIF3A	protein_coding
0,6828036	TRDMT1	protein_coding
0,9248086	DDX50	protein_coding
0,9700557	MAPK8	protein_coding
0,8930727	SEC23IP	protein_coding
0,5558351	ATE1	protein_coding
0,8330359	NSMCE4A	protein_coding
0,8176511	PLEKHA1	protein_coding
0,2948143	UNC5B	protein_coding
0,9538818	VSIR	protein_coding
0,2347224	SPOCK2	protein_coding
0,8495694	MICU1	protein_coding
0,9112805	PPP3CB	protein_coding

0,9759101	CCSER2	protein_coding
0,9902795	BMPR1A	protein_coding
0,1112135	ACTA2	protein_coding
0,669182	LIPA	protein_coding
0,5040596	LZTS2	protein_coding
0,4788751	SFXN3	protein_coding
0,9528194	FBXW4	protein_coding
0,620901	TNKS2	protein_coding
0,7661133	GBF1	protein_coding
0,4954628	ARHGAP21	protein_coding
0,8373584	CPEB3	protein_coding
0,9291102	CUEDC2	protein_coding
0,5558351	ANKRD26	protein_coding
0,8714772	ACBD5	protein_coding
0,9621856	LARP4B	protein_coding
0,4894579	GTPBP4	protein_coding
0,8153751	EDRF1	protein_coding
0,5212854	BCCIP	protein_coding
0,6638064	MTPAP	protein_coding
0,9738194	SH3PXD2A	protein_coding
0,8274305	PITRM1	protein_coding
0,9917279	STN1	protein_coding
0,887319	MAP3K8	protein_coding
0,4746597	EBF3	protein_coding
0,2539546	GLRX3	protein_coding
0,9185201	TASOR2	protein_coding
0,6319662	XPNPEP1	protein_coding
0,9463928	SMC3	protein_coding
0,749866	SHOC2	protein_coding
0,8831146	TFAM	protein_coding
0,9928407	CCDC6	protein_coding
0,8259599	CUL2	protein_coding
0,2337466	CCNY	protein_coding
0,8117718	RPL28	protein_coding
0,8992483	ZMIZ1	protein_coding
0,3772742	PPIF	protein_coding
0,4626741	TSPAN14	protein_coding
0,5558351	TBC1D12	protein_coding
0,8041431	NUFIP2	protein_coding
0,9378237	GIT1	protein_coding
0,5474737	RPL19	protein_coding
0,4165098	FBXL20	protein_coding
0,3358222	UBTF	protein_coding
0,8930787	PSMD3	protein_coding
0,6854132	CASC3	protein_coding
0,5024483	RAD51C	protein_coding
0,9925196	SEPTIN4	protein_coding
0,8176787	MTMR4	protein_coding
0,572303	TRIM37	protein_coding
0,7393702	DHX40	protein_coding
0,7894641	KPNB1	protein_coding
0,7444016	GOSR2	protein_coding
0,9738194	PNPO	protein_coding
0,9003006	RPS6KB1	protein_coding
0,747834	CBX1	protein_coding
0,587388	INTS2	protein_coding
0,5365997	MED13	protein_coding
0,1712026	ENO3	protein_coding
0,9039795	PFN1	protein_coding

0,5793003	RNF167	protein_coding
0,7542355	SLC25A11	protein_coding
0,6887681	NUP88	protein_coding
0,2778507	C1QBP	protein_coding
0,6144866	BLMH	protein_coding
0,4835164	CPD	protein_coding
0,6927966	GOSR1	protein_coding
0,2560075	CCDC47	protein_coding
0,9922688	DRG2	protein_coding
0,5180247	FTSJ3	protein_coding
0,8698319	AKAP10	protein_coding
0,7956408	SMARCD2	protein_coding
0,7136899	SYNGR2	protein_coding
0,4971845	UTP6	protein_coding
0,8823093	DDX5	protein_coding
0,696332	C17orf75	protein_coding
0,5651785	CYTH1	protein_coding
0,4151093	PSMD11	protein_coding
0,7230802	LGALS3BP	protein_coding
0,9081173	CCL2	protein_coding
0,9385376	KAT2A	protein_coding
0,330331	MLX	protein_coding
0,50493	EZH1	protein_coding
0,6227414	COL1A1	protein_coding
0,9781971	SGCA	protein_coding
0,0606893	MRPL27	protein_coding
0,9828271	VAT1	protein_coding
0,9759101	LRRC59	protein_coding
0,5456715	HDAC5	protein_coding
0,9450135	LUC7L3	protein_coding
0,3515446	SMURF2	protein_coding
0,9206489	DUSP3	protein_coding
0,9010513	EFTUD2	protein_coding
0,182619	HLF	protein_coding
0,7190863	PRKAR1A	protein_coding
0,3607643	FAM20A	protein_coding
0,6160654	YWHAE	protein_coding
0,4835164	MMD	protein_coding
0,9535694	MAP2K6	protein_coding
0,2281984	DHRS7B	protein_coding
0,9998478	WSB1	protein_coding
0,3607643	VTN	protein_coding
0,8330359	TNFAIP1	protein_coding
0,0727176	PMP22	protein_coding
0,1712026	ALDOC	protein_coding
0,620901	SUPT6H	protein_coding
0,3030424	RAB34	protein_coding
0,887319	PHF12	protein_coding
0,9953531	TMEM33	protein_coding
0,727014	SLAIN2	protein_coding
0,4835164	OCIAD1	protein_coding
0,9312186	DCUN1D4	protein_coding
0,8829558	USP46	protein_coding
0,6761449	LAMTOR3	protein_coding
0,4835164	NFKB1	protein_coding
0,4161706	MANBA	protein_coding
0,6790658	UBE2D3	protein_coding
0,5723744	MAPK10	protein_coding
0,9778442	ELF2	protein_coding

0,1670869	NDUFC1	protein_coding
0,3194617	TBC1D9	protein_coding
0,4778798	ZNF330	protein_coding
0,9438763	INPP4B	protein_coding
0,9442712	GAB1	protein_coding
0,9669517	KLHL2	protein_coding
0,4783874	CPE	protein_coding
0,8373584	RPL34	protein_coding
0,9951314	WFS1	protein_coding
0,3155884	GRPEL1	protein_coding
0,5117223	FRG1	protein_coding
0,2870346	CLCN3	protein_coding
0,9082846	GALNT7	protein_coding
0,5679388	DHX15	protein_coding
0,9953531	SEPSECS	protein_coding
0,9645236	TRIM2	protein_coding
0,6371005	FBXW7	protein_coding
0,5313262	TBC1D19	protein_coding
0,7639764	NSD2	protein_coding
0,4835164	SH3D19	protein_coding
0,7611529	STIM2	protein_coding
0,6609893	GLRB	protein_coding
0,9578269	RAPGEF2	protein_coding
0,9680323	SNX25	protein_coding
0,645515	LRP2BP	protein_coding
0,7558125	UFSP2	protein_coding
0,8422988	KLF3	protein_coding
0,8614902	KLHL5	protein_coding
0,6140495	UGDH	protein_coding
0,7369294	PPARGC1A	protein_coding
0,8885864	CRYAB	protein_coding
0,3520154	HTATIP2	protein_coding
0,7211115	CTSC	protein_coding
0,9296784	ZBTB16	protein_coding
0,7767291	ELP4	protein_coding
0,9998478	ZPR1	protein_coding
0,1674733	MTCH2	protein_coding
0,6419483	FNBP4	protein_coding
0,9428271	SC5D	protein_coding
0,9459932	HSPA8	protein_coding
0,4809815	VWA5A	protein_coding
0,2529607	SIAE	protein_coding
0,9768456	EHD1	protein_coding
0,4475789	OSBP	protein_coding
0,692146	KMT5B	protein_coding
0,6591127	FOXRED1	protein_coding
0,6184079	PPP6R3	protein_coding
0,5647201	MS4A6A	protein_coding
0,0895372	MS4A4A	protein_coding
0,373012	ST3GAL4	protein_coding
0,6563172	CPT1A	protein_coding
0,0868691	CCND1	protein_coding
0,9925374	PRPF19	protein_coding
0,9998478	TMEM109	protein_coding
0,8908494	CHORDC1	protein_coding
0,6044343	PANX1	protein_coding
0,7211115	ARHGEF17	protein_coding
0,5011485	CEP164	protein_coding
0,7245119	RNF141	protein_coding

0,768388	CEP126	protein_coding
0,9626169	EIF4G2	protein_coding
0,9205351	IL10RA	protein_coding
0,6238595	GALNT18	protein_coding
0,9341044	BIRC2	protein_coding
0,716221	UBE4A	protein_coding
0,5296182	DDX6	protein_coding
0,7468242	CBL	protein_coding
0,5478951	HIPK3	protein_coding
0,8698319	FBXO3	protein_coding
0,3860206	PDHX	protein_coding
0,3335334	COMMD9	protein_coding
0,5393446	ACCS	protein_coding
0,7546576	AMBRA1	protein_coding
0,9855435	MADD	protein_coding
0,6137496	NAA40	protein_coding
0,4599193	CARS	protein_coding
0,7321942	CD81	protein_coding
0,1712026	C11orf21	protein_coding
0,5267449	SOX6	protein_coding
0,9808899	C11orf58	protein_coding
0,8885864	RPS13	protein_coding
0,9312186	NUP98	protein_coding
0,6662006	NDUFS8	protein_coding
0,9817008	CHKA	protein_coding
0,8118765	HPS5	protein_coding
0,9707377	GTF2H1	protein_coding
0,472118	VWF	protein_coding
0,4971178	PPFIBP1	protein_coding
0,8883426	PRDM4	protein_coding
0,9802452	CLEC2B	protein_coding
0,3358222	COQ5	protein_coding
0,7558125	CORO1C	protein_coding
0,9928407	CAPRIN2	protein_coding
0,7118106	KCTD10	protein_coding
0,2393106	SLC11A2	protein_coding
0,864196	MLEC	protein_coding
0,9064657	CSRNP2	protein_coding
0,7558125	CAMKK2	protein_coding
0,5265966	ATP5F1B	protein_coding
0,9829971	PTGES3	protein_coding
0,231981	BCL7A	protein_coding
0,6408377	RSRC2	protein_coding
0,7456311	LIN7A	protein_coding
0,9953531	ACSS3	protein_coding
0,8189129	TNS2	protein_coding
0,4879925	METAP2	protein_coding
0,8458063	LTA4H	protein_coding
0,9428271	ELK3	protein_coding
0,8917289	MAGOHB	protein_coding
0,8865445	ITFG2	protein_coding
0,9925374	PARP11	protein_coding
0,7748443	ARPC3	protein_coding
0,6462349	GPN3	protein_coding
0,3513961	VPS29	protein_coding
0,8189129	MYL2	protein_coding
0,9142106	SH2B3	protein_coding
0,284683	DUSP16	protein_coding
0,446175	CREBL2	protein_coding

0,8912641	ACAD10	protein_coding
0,931978	ALDH2	protein_coding
0,1712026	CDKN1B	protein_coding
0,3953915	NAA25	protein_coding
0,7204811	LTBR	protein_coding
0,4161706	CDK2AP1	protein_coding
0,2908312	OAS3	protein_coding
0,5677206	OAS2	protein_coding
0,3159229	MGP	protein_coding
0,8866985	ARHGDIB	protein_coding
0,8545769	GTF2H3	protein_coding
0,8997937	EIF2B1	protein_coding
0,645515	SLC38A1	protein_coding
0,7280026	C12orf49	protein_coding
0,887128	STX2	protein_coding
0,8555218	ADGRD1	protein_coding
0,6430147	COPZ1	protein_coding
0,6790658	CAND1	protein_coding
0,8888845	RAB5B	protein_coding
0,5647201	MDM1	protein_coding
0,7558125	NUP107	protein_coding
0,8118149	CNOT2	protein_coding
0,914514	CPSF6	protein_coding
0,6333516	KRR1	protein_coding
0,1606381	MRPL51	protein_coding
0,6491869	GAPDH	protein_coding
0,9535559	CHD4	protein_coding
0,6745473	UHRF1BP1L	protein_coding
0,9576401	COPS7A	protein_coding
0,721162	ING4	protein_coding
0,1080307	CHPT1	protein_coding
0,7125689	USP5	protein_coding
0,2834613	TPI1	protein_coding
0,7118106	GNPTAB	protein_coding
0,5011485	ENO2	protein_coding
0,8554227	ATN1	protein_coding
0,4806087	SUDS3	protein_coding
0,9933479	GOLT1B	protein_coding
0,4593782	LDHB	protein_coding
0,933129	PRKAB1	protein_coding
0,7748443	CMAS	protein_coding
0,9621537	HCFC2	protein_coding
0,9248086	C2CD5	protein_coding
0,9914435	RAB35	protein_coding
0,6903585	PHC1	protein_coding
0,8021601	COX6A1	protein_coding
0,9112805	RIC8B	protein_coding
0,841477	SRSF9	protein_coding
0,8875885	FGFR1OP2	protein_coding
0,5608916	COL12A1	protein_coding
0,3737635	BTN3A3	protein_coding
0,9296784	TDP2	protein_coding
0,6867028	FRK	protein_coding
0,7298242	DSE	protein_coding
0,2945575	RWDD1	protein_coding
0,9908528	TMEM14C	protein_coding
0,4911389	PAK1IP1	protein_coding
0,0868691	GCNT2	protein_coding
0,7743102	SMIM8	protein_coding

0,7318327	NEDD9	protein_coding
0,2088169	CEP85L	protein_coding
0,4779616	ASF1A	protein_coding
0,8153751	MCM9	protein_coding
0,8511804	RNGTT	protein_coding
0,7558125	MAN1A1	protein_coding
0,7118106	SERINC1	protein_coding
0,1225394	HDDC2	protein_coding
0,1067405	TPD52L1	protein_coding
0,7558125	HINT3	protein_coding
0,4835164	NCOA7	protein_coding
0,9494633	SASH1	protein_coding
0,0561363	MTRF1L	protein_coding
0,9700303	MAPK14	protein_coding
0,9887908	KCTD20	protein_coding
0,7010516	STK38	protein_coding
0,7956408	SRSF3	protein_coding
0,9933219	SOD2	protein_coding
0,2834613	MRPL18	protein_coding
0,998169	MCM3	protein_coding
0,7197731	RNF8	protein_coding
0,6285359	PHACTR1	protein_coding
0,3811175	ICK	protein_coding
0,7087026	FBXO9	protein_coding
0,9962215	MDN1	protein_coding
0,7634906	RBM24	protein_coding
0,7203736	CAP2	protein_coding
0,8992483	ZNF451	protein_coding
0,4593782	BAG2	protein_coding
0,34092	RAB23	protein_coding
0,9778442	FBXL4	protein_coding
0,9669517	CCNC	protein_coding
0,9496064	E2F3	protein_coding
0,9928407	ASCC3	protein_coding
0,8062191	BVES	protein_coding
0,9998478	MED23	protein_coding
0,5456715	ALDH5A1	protein_coding
0,9678404	CRYBG1	protein_coding
0,0839464	ACOT13	protein_coding
0,8695843	SMAP1	protein_coding
0,7987424	RPS12	protein_coding
0,4958925	C6orf62	protein_coding
0,7940001	EYA4	protein_coding
0,9977992	SOBP	protein_coding
0,5768837	SNX3	protein_coding
0,5052075	HBS1L	protein_coding
0,749866	TRIM38	protein_coding
0,9583561	FIG4	protein_coding
0,5985056	PERP	protein_coding
0,4219373	ARFGEF3	protein_coding
0,645515	HECA	protein_coding
0,3256015	ADGRG6	protein_coding
0,5953643	PHACTR2	protein_coding
0,7464528	EPM2A	protein_coding
0,9792506	SLC39A7	protein_coding
0,9156816	PHF1	protein_coding
0,904174	CUTA	protein_coding
0,8588806	QKI	protein_coding
0,6442182	PDE10A	protein_coding

0,6279799	SMOC2	protein_coding
0,8114908	CCND3	protein_coding
0,8461755	FAM120B	protein_coding
0,4834958	BICRAL	protein_coding
0,9410289	PPP2R5D	protein_coding
0,8847411	SRF	protein_coding
0,6999614	CUL9	protein_coding
0,1067405	DUSP22	protein_coding
0,8992483	EXOC2	protein_coding
0,4298619	COX7A2	protein_coding
0,9081173	TMEM30A	protein_coding
0,9151358	SENP6	protein_coding
0,7956408	VEGFA	protein_coding
0,8885864	PRPF4B	protein_coding
0,9465232	SLC29A1	protein_coding
0,7431973	BTN2A1	protein_coding
0,4020941	LAMA4	protein_coding
0,9755908	TENT5A	protein_coding
0,9535559	CLIC5	protein_coding
0,0998266	TBX18	protein_coding
0,9847107	ERBIN	protein_coding
0,9998538	HARS2	protein_coding
0,4521055	NUDT12	protein_coding
0,8346156	MAN2A1	protein_coding
0,9802452	SEMA5A	protein_coding
0,441257	C7	protein_coding
0,7118106	TENT4A	protein_coding
0,9481776	GHR	protein_coding
0,713782	HMGCS1	protein_coding
0,4105939	DAP	protein_coding
0,6459868	BRD8	protein_coding
0,7431559	NNT	protein_coding
0,7122873	MRPS30	protein_coding
0,6762366	HSPA9	protein_coding
0,8725749	MRPS27	protein_coding
0,6184746	PFDN1	protein_coding
0,4585504	HBEGF	protein_coding
0,3792977	LOX	protein_coding
0,2629376	SPARC	protein_coding
0,9865636	IK	protein_coding
0,9768456	HMGCR	protein_coding
0,7444016	CERT1	protein_coding
0,8818298	FAF2	protein_coding
0,4933888	PDE8B	protein_coding
0,7006869	CLK4	protein_coding
0,8422988	RNF130	protein_coding
0,3907697	ARSB	protein_coding
0,4835164	CLINT1	protein_coding
0,71754	THBS4	protein_coding
0,9481776	CNOT6	protein_coding
0,2971924	TTC1	protein_coding
0,3424108	MSH3	protein_coding
0,2737133	RASGRF2	protein_coding
0,7467854	CCNG1	protein_coding
0,7968733	DROSHA	protein_coding
0,9951314	ARRDC3	protein_coding
0,9731251	GOLPH3	protein_coding
0,7816527	SUB1	protein_coding
0,0088417	NPR3	protein_coding

0,998169	FAM172A	protein_coding
0,0088417	SLC27A6	protein_coding
0,7607016	TARS	protein_coding
0,99108	LNPEP	protein_coding
0,1986027	PDE4D	protein_coding
0,0149003	RAD1	protein_coding
0,5356307	BRIX1	protein_coding
0,9954421	SLC12A7	protein_coding
0,9768456	ST8SIA4	protein_coding
0,9484395	GNPDA1	protein_coding
0,9781971	PCDH12	protein_coding
0,8003414	SKP1	protein_coding
0,9579484	NUP155	protein_coding
0,9998478	PPP2CA	protein_coding
0,5268876	FGF1	protein_coding
0,492696	NR3C1	protein_coding
0,8238032	C5orf15	protein_coding
0,7740102	PPWD1	protein_coding
0,7743102	LIFR	protein_coding
0,8229361	TRIM23	protein_coding
0,4894579	TRAPPC13	protein_coding
0,4532937	SEC24A	protein_coding
0,2506935	TXNDC15	protein_coding
0,8545769	TTC33	protein_coding
0,8721281	RARS	protein_coding
0,3830886	H2AFY	protein_coding
0,4954628	TCERG1	protein_coding
0,8281575	DPYSL3	protein_coding
0,9765371	SMAD5	protein_coding
0,645515	CSNK1A1	protein_coding
0,9974735	HMGXB3	protein_coding
0,9621537	ERGIC1	protein_coding
0,9840915	PDGFRB	protein_coding
0,620901	ATP6V0E1	protein_coding
0,0375734	STC2	protein_coding
0,9450135	CPEB4	protein_coding
0,2318378	HRH2	protein_coding
0,3803906	DBN1	protein_coding
0,8381234	EHHADH	protein_coding
0,3694563	SMC4	protein_coding
0,4919121	SELENOK	protein_coding
0,7154026	ACTR8	protein_coding
0,2440137	TIMMDC1	protein_coding
0,8422988	CRBN	protein_coding
0,1528801	BCL6	protein_coding
0,8275847	NPHP3	protein_coding
0,4599193	AMOTL2	protein_coding
0,2068936	NIT2	protein_coding
0,2731548	FAM162A	protein_coding
0,8561022	KPNA1	protein_coding
0,9465232	PCCB	protein_coding
0,9450135	UBE3A	protein_coding
0,3513961	ARMC8	protein_coding
0,7611529	CEP70	protein_coding
0,6766513	SLC25A36	protein_coding
0,9998478	RNF7	protein_coding
0,645515	TFDP2	protein_coding
0,9997554	XRN1	protein_coding
0,5953643	KAT2B	protein_coding

0,7397205	BCHE	protein_coding
0,5510556	PDCD10	protein_coding
0,6664224	FGF12	protein_coding
0,9717962	PRKAR2A	protein_coding
0,0903439	HES1	protein_coding
0,9727051	USP4	protein_coding
0,8422988	ACAP2	protein_coding
0,5029234	ECT2	protein_coding
0,9176029	GNAI2	protein_coding
0,9478428	TFG	protein_coding
0,9619323	USP9Y	protein_coding
0,2073391	HYAL1	protein_coding
0,4626741	TUSC2	protein_coding
0,9341044	RPL24	protein_coding
0,6613295	FXR1	protein_coding
0,1852138	CBLB	protein_coding
0,6184746	BBX	protein_coding
0,5117223	IFT57	protein_coding
0,90504	GNB4	protein_coding
0,9361547	GBE1	protein_coding
0,8062191	UMPS	protein_coding
0,7506449	NCBP2	protein_coding
0,8930787	SNX4	protein_coding
0,8247964	FRMD4B	protein_coding
0,9233889	SLC41A3	protein_coding
0,7740102	PLXNA1	protein_coding
0,9721598	ATP6V1A	protein_coding
0,7088772	KLHL18	protein_coding
0,9242864	SCAP	protein_coding
0,5265966	MRPL3	protein_coding
0,3049645	PLSCR4	protein_coding
0,9771336	HEMK1	protein_coding
0,5786535	MAPKAPK3	protein_coding
0,6461001	ACVR2B	protein_coding
0,8213028	WDR48	protein_coding
0,9839612	COMMD2	protein_coding
0,933129	GORASP1	protein_coding
0,5268669	ABCC5	protein_coding
0,3942661	ABHD14B	protein_coding
0,6602321	EIF1B	protein_coding
0,601474	ARHGEF26	protein_coding
0,9997039	KLHL24	protein_coding
0,7894641	DNAH1	protein_coding
0,8091803	SSR3	protein_coding
0,284683	ZBTB47	protein_coding
0,8285832	TNNC1	protein_coding
0,4894579	NKTR	protein_coding
0,8946561	FOXP1	protein_coding
0,9064657	EIF4G1	protein_coding
0,8062191	SPCS1	protein_coding
0,4608748	NEK4	protein_coding
0,8578793	SLC4A3	protein_coding
0,943695	INO80D	protein_coding
0,7511084	EEF1B2	protein_coding
0,7321942	ADAM23	protein_coding
0,9118275	MOB1A	protein_coding
0,7382254	KANSL3	protein_coding
0,8992483	LMAN2L	protein_coding
0,3797364	TTL	protein_coding

0,9370061	PIKFYVE	protein_coding
0,8590905	FAHD2A	protein_coding
0,6128477	NCL	protein_coding
0,7307715	ACTR1B	protein_coding
0,5847346	SLC35F5	protein_coding
0,9925374	ACTR3	protein_coding
0,8474324	EPB41L5	protein_coding
0,769458	SF3B6	protein_coding
0,1703144	DNAJC27	protein_coding
0,7921707	STAM2	protein_coding
0,7968733	GPD2	protein_coding
0,8345589	ACVR1	protein_coding
0,7085673	TANC1	protein_coding
0,9481776	MPV17	protein_coding
0,9185201	GTF3C2	protein_coding
0,5140003	EIF2B4	protein_coding
0,8062191	NRBP1	protein_coding
0,3451145	PSMD14	protein_coding
0,5765517	SNX17	protein_coding
0,9998478	ASB3	protein_coding
0,6220194	PPM1G	protein_coding
0,2318378	PDE1A	protein_coding
0,5511615	IFIH1	protein_coding
0,8721281	RPS15	protein_coding
0,9125449	GCA	protein_coding
0,7972313	MOGS	protein_coding
0,9194018	CLIP4	protein_coding
0,5267449	SPTBN1	protein_coding
0,7219051	AUP1	protein_coding
0,271425	RTN4	protein_coding
0,8550924	CCDC88A	protein_coding
0,8992483	MRPL19	protein_coding
0,8067508	LANCL1	protein_coding
0,54642	WDR75	protein_coding
0,1131434	EFEMP1	protein_coding
0,8946561	FANCL	protein_coding
0,1124027	FN1	protein_coding
0,2984194	STAT1	protein_coding
0,4498511	GLS	protein_coding
0,997931	PAPOLG	protein_coding
0,8823913	UNC50	protein_coding
0,4032255	IGFBP2	protein_coding
0,4670954	ELMOD3	protein_coding
0,8846329	IGFBP5	protein_coding
0,9074092	USP34	protein_coding
0,8875885	CCT4	protein_coding
0,887319	GGCX	protein_coding
0,993752	EHBP1	protein_coding
0,6818653	TXNDC9	protein_coding
0,8221205	COQ10B	protein_coding
0,7952422	SF3B1	protein_coding
0,9977992	ST3GAL5	protein_coding
0,8255851	CHST10	protein_coding
0,645515	PDCL3	protein_coding
0,4011774	MOB4	protein_coding
0,3797364	HSPE1	protein_coding
0,9438763	KDM3A	protein_coding
0,995098	CHMP3	protein_coding
0,3268775	SMYD1	protein_coding

0,6383874	IL1R1	protein_coding
0,2347224	FHL2	protein_coding
0,9406097	MLPH	protein_coding
0,9374881	CNPPD1	protein_coding
0,8593223	UXS1	protein_coding
0,7025012	HDLBP	protein_coding
0,9290744	PPP1R7	protein_coding
0,902171	STK25	protein_coding
0,176447	ID2	protein_coding
0,9628671	TAF1B	protein_coding
0,1773766	ODC1	protein_coding
0,9884769	BIRC6	protein_coding
0,738122	NOL10	protein_coding
0,4070535	PLEKHB2	protein_coding
0,8568617	GORASP2	protein_coding
0,5397488	STRN	protein_coding
0,8555218	CEBPZ	protein_coding
0,7645994	PRKD3	protein_coding
0,7569416	DCAF17	protein_coding
0,997931	RAB3GAP1	protein_coding
0,4311333	SLC25A12	protein_coding
0,736331	DARS	protein_coding
0,7296863	SRSF7	protein_coding
0,5193296	PLCL1	protein_coding
0,7403796	SOS1	protein_coding
0,8796725	WIPF1	protein_coding
0,8955637	ORC2	protein_coding
0,621558	COX7A2L	protein_coding
0,623981	PNO1	protein_coding
0,5347705	ORC4	protein_coding
0,4498511	PLEK	protein_coding
0,855954	RND3	protein_coding
0,997931	ATF2	protein_coding
0,9082846	THADA	protein_coding
0,8874269	AAK1	protein_coding
0,7912354	TRAK2	protein_coding
0,8318664	TIA1	protein_coding
0,9296784	PCYOX1	protein_coding
0,7954765	EPAS1	protein_coding
0,5558351	SUMO1	protein_coding
0,3881918	NFE2L2	protein_coding
0,9914435	MSH6	protein_coding
0,6269745	PLEKHA3	protein_coding
0,9533508	SPR	protein_coding
0,998169	EPHA4	protein_coding
0,5140003	PARD3B	protein_coding
0,2568349	FARSB	protein_coding
0,6633625	ALMS1	protein_coding
0,8481719	BCL9	protein_coding
0,2231549	PRRX1	protein_coding
0,9112805	DHCR24	protein_coding
0,8095229	DNAJC16	protein_coding
0,7309156	MARK1	protein_coding
0,191133	CACYBP	protein_coding
0,8373546	SCP2	protein_coding
0,0895372	RALGPS2	protein_coding
0,5558351	ANGPTL1	protein_coding
0,5017354	CEP104	protein_coding
0,955797	FAM20B	protein_coding

0,673546	TCEANC2	protein_coding
0,4344504	TMEM59	protein_coding
0,692146	LRRC42	protein_coding
0,1738303	MRPL37	protein_coding
0,2182656	ICMT	protein_coding
0,8229361	RPL22	protein_coding
0,505053	QSOX1	protein_coding
0,824287	STXBP3	protein_coding
0,7318327	ERRFI1	protein_coding
0,435547	PARK7	protein_coding
0,630319	SRSF4	protein_coding
0,7154026	MECR	protein_coding
0,5140003	KCNC4	protein_coding
0,8721867	EDEM3	protein_coding
0,6378198	WDR77	protein_coding
0,252949	ATP5PB	protein_coding
0,5245497	RAP1A	protein_coding
0,993752	HDAC1	protein_coding
0,9628671	CAPZA1	protein_coding
0,8373584	S100PBP	protein_coding
0,5647201	RNF19B	protein_coding
0,7062909	SCAMP3	protein_coding
0,6285359	ASH1L	protein_coding
0,855954	SFPQ	protein_coding
0,9603194	RHOU	protein_coding
0,7611529	GON4L	protein_coding
0,6244656	ARHGEF2	protein_coding
0,8117718	MEF2D	protein_coding
0,927176	DOCK7	protein_coding
0,83716	C1orf21	protein_coding
0,7639726	SWT1	protein_coding
0,7984578	DNAJC6	protein_coding
0,1773741	LEPR	protein_coding
0,8721867	IVNS1ABP	protein_coding
0,997931	KIAA2013	protein_coding
0,7677769	MFN2	protein_coding
0,9946475	SMG7	protein_coding
0,7118106	NCF2	protein_coding
0,3792977	SLC35D1	protein_coding
0,2223198	GADD45A	protein_coding
0,8647372	WLS	protein_coding
0,9998538	PRDM2	protein_coding
0,5139593	RGS2	protein_coding
0,8992483	RO60	protein_coding
0,3803906	UCHL5	protein_coding
0,5890453	BCAS2	protein_coding
0,7820981	SRSF11	protein_coding
0,54642	OLFML3	protein_coding
0,9603194	PLEKHM2	protein_coding
0,4971845	CRYZ	protein_coding
0,6438613	PHTF1	protein_coding
0,6137496	CD58	protein_coding
0,808509	TTF2	protein_coding
0,290302	TMEM9	protein_coding
0,673546	MAP7D1	protein_coding
0,9678404	WARS2	protein_coding
0,1052063	MRPS15	protein_coding
0,7987982	EXOC8	protein_coding
0,3850967	GNPAT	protein_coding

0,5776471	TSNAX	protein_coding
0,8851892	RRAGC	protein_coding
0,1131434	NID1	protein_coding
0,5402971	LGALS8	protein_coding
0,7641298	MTR	protein_coding
0,7462938	SIPA1L2	protein_coding
0,9729242	RLF	protein_coding
0,6027003	AKT3	protein_coding
0,2026798	ETV3	protein_coding
0,6463814	ACADM	protein_coding
0,654521	ADGRL2	protein_coding
0,1431468	SDHB	protein_coding
0,5337323	RPF1	protein_coding
0,445647	KDM5B	protein_coding
0,195587	UAP1	protein_coding
0,9792506	CTBS	protein_coding
0,9298036	KLHL12	protein_coding
0,9540553	SSX2IP	protein_coding
0,7572071	ZNHIT6	protein_coding
0,9358487	RBBP5	protein_coding
0,3371364	GBP3	protein_coding
0,6925346	GBP1	protein_coding
0,3881918	GPR89A	protein_coding
0,829234	CDK18	protein_coding
0,8229361	RAB29	protein_coding
0,7237365	ECE1	protein_coding
0,7558125	HMGCL	protein_coding
0,1856054	ID3	protein_coding
0,8163405	CD46	protein_coding
0,7984312	PRPF3	protein_coding
0,5296182	APH1A	protein_coding
0,427478	SLC2A1	protein_coding
0,7291201	EBNA1BP2	protein_coding
0,6818653	IPO13	protein_coding
0,8682799	ATP6V0B	protein_coding
0,9984673	B4GALT2	protein_coding
0,9975676	ERI3	protein_coding
0,9303646	AKR1A1	protein_coding
0,0938658	PRDX1	protein_coding
0,5537742	PIK3R3	protein_coding
0,7313486	BLZF1	protein_coding
0,6468834	SLC19A2	protein_coding
0,7611529	NSUN4	protein_coding
0,7909474	TMED5	protein_coding
0,7972313	DR1	protein_coding
0,933129	CNN3	protein_coding
0,8622295	PRRC2C	protein_coding
0,284683	F3	protein_coding
0,5879554	ABCD3	protein_coding
0,9496064	VAMP4	protein_coding
0,749866	DPH5	protein_coding
0,2318378	PTBP2	protein_coding
0,1284788	PRDX6	protein_coding
0,9953531	DARS2	protein_coding
0,4246801	IRF6	protein_coding
0,5656126	UTP25	protein_coding
0,9541981	SYF2	protein_coding
0,3194617	RSRP1	protein_coding
0,9778442	SLC35A3	protein_coding

0,8992483	RCOR3	protein_coding
0,3194617	STMN1	protein_coding
0,6543081	MTFR1L	protein_coding
0,400727	MAN1C1	protein_coding
0,7740102	DHDDS	protein_coding
0,9464798	NENF	protein_coding
0,6156953	NSL1	protein_coding
0,8858562	PROX1	protein_coding
0,9910925	ARID1A	protein_coding
0,6583765	RPA2	protein_coding
0,993752	PPP1R8	protein_coding
0,770735	STX12	protein_coding
0,1326966	MARC2	protein_coding
0,7956173	OSBPL9	protein_coding
0,9630144	ESYT2	protein_coding
0,6870873	MESD	protein_coding
0,9781971	RCN2	protein_coding
0,9233889	CTSD	protein_coding
0,8447416	STAG1	protein_coding
0,9840915	STK11	protein_coding
0,4848227	KMT2A	protein_coding
0,8155634	IFT46	protein_coding
0,9925374	RPS25	protein_coding
0,8578622	TNNT2	protein_coding
0,8812706	DDX59	protein_coding
0,9002008	CAMSAP2	protein_coding
0,878778	ATF6	protein_coding
0,4954628	FASTKD2	protein_coding
0,3803906	NRP2	protein_coding
0,9998478	CREB1	protein_coding
0,9450135	KLF7	protein_coding
0,1738303	B4GALT6	protein_coding
0,8318664	SPCS2	protein_coding
0,891947	FILIP1	protein_coding
0,6870873	CASP8AP2	protein_coding
0,3503253	HMGN3	protein_coding
0,8481719	ANKRD13C	protein_coding
0,3943278	SGIP1	protein_coding
0,8992483	PHF3	protein_coding
0,6564675	PLAGL1	protein_coding
0,9564455	FBXO30	protein_coding
0,284683	TNFAIP3	protein_coding
0,9296784	AKAP7	protein_coding
0,5558351	SGK1	protein_coding
0,895571	RNF146	protein_coding
0,3034549	CCN2	protein_coding
0,7558125	FBXL5	protein_coding
0,8373584	MED28	protein_coding
0,9428271	SLC16A7	protein_coding
0,8841902	ZNF430	protein_coding
0,8847411	MYL12B	protein_coding
0,8131645	FOXO3	protein_coding
0,9919804	ARMC2	protein_coding
0,5537742	RPN2	protein_coding
0,7126808	CASQ2	protein_coding
0,4498511	PKD2	protein_coding
0,3697716	SPP1	protein_coding
0,762448	CCNI	protein_coding
0,8885864	RARRES1	protein_coding

0,8500846	MFSD1	protein_coding
0,997931	RAB3GAP2	protein_coding
0,5456715	PPL	protein_coding
0,9962215	UBN1	protein_coding
0,9946475	KLF12	protein_coding
0,6543081	PCDH17	protein_coding
0,7506449	HS1BP3	protein_coding
0,9995727	LDAH	protein_coding
0,5647201	WDR35	protein_coding
0,6132228	CCND2	protein_coding
0,7836389	ELL2	protein_coding
0,5537742	CYP20A1	protein_coding
0,1674733	NDUFB3	protein_coding
0,6244656	GTF3C3	protein_coding
0,7596217	UBE2B	protein_coding
0,7606144	KLF9	protein_coding
0,8721867	ITGB1BP1	protein_coding
0,7572724	CPSF3	protein_coding
0,9715104	SENP5	protein_coding
0,4806087	CCDC92	protein_coding
0,3358601	C1orf198	protein_coding
0,9840915	HEATR1	protein_coding
0,7956173	PTBP3	protein_coding
0,8888699	RAD23B	protein_coding
0,5265966	FKBP15	protein_coding
0,6206631	CTNNAL1	protein_coding
0,8870441	ABITRAM	protein_coding
0,9528744	SET	protein_coding
0,7062909	PTPA	protein_coding
0,8775933	GLE1	protein_coding
0,6438613	RAB14	protein_coding
0,5011485	CNTRL	protein_coding
0,9481776	TRIM32	protein_coding
0,9709611	FBXW2	protein_coding
0,861259	PHF19	protein_coding
0,4886289	NEK6	protein_coding
0,8885783	PPP6C	protein_coding
0,2687129	NDUFA8	protein_coding
0,6613295	RBM18	protein_coding
0,9481776	HSDL2	protein_coding
0,615864	MAPKAP1	protein_coding
0,7114949	NR4A3	protein_coding
0,8099847	INVS	protein_coding
0,671498	DENND1A	protein_coding
0,8682129	ALG2	protein_coding
0,7558125	KDSR	protein_coding
0,6673583	VPS4B	protein_coding
0,7803562	YLPM1	protein_coding
0,9578517	FCF1	protein_coding
0,7385573	NEK9	protein_coding
0,5140003	IFT43	protein_coding
0,8318664	NPC2	protein_coding
0,1881669	DNAL1	protein_coding
0,933129	IRF2BPL	protein_coding
0,7980625	ACOT2	protein_coding
0,1284788	LTBP2	protein_coding
0,6795819	AREL1	protein_coding
0,3776155	MLH3	protein_coding
0,9953531	TTLL5	protein_coding

0,9411751	ABCD4	protein_coding
0,6048439	DLST	protein_coding
0,6563172	TGFB3	protein_coding
0,2150608	SLIRP	protein_coding
0,8522628	RBM25	protein_coding
0,3801685	ALDH6A1	protein_coding
0,5445815	EIF2B2	protein_coding
0,7321942	NRDE2	protein_coding
0,5117223	COQ6	protein_coding
0,7607016	RHOQ	protein_coding
0,9700303	SUPT7L	protein_coding
0,5180247	DNMT3A	protein_coding
0,9376695	TMEM214	protein_coding
0,9562513	ATAD2B	protein_coding
0,855954	ATL2	protein_coding
0,8062191	YPEL5	protein_coding
0,855954	FAM98A	protein_coding
0,9312186	YIPF4	protein_coding
0,8373584	AFTPH	protein_coding
0,5879554	LGALSL	protein_coding
0,445647	CNRIP1	protein_coding
0,4954628	CRIPT	protein_coding
0,4096736	SLC17A5	protein_coding
0,6776989	OGFRL1	protein_coding
0,7596217	SLF2	protein_coding
0,5474737	IDE	protein_coding
0,6425583	IFIT3	protein_coding
0,310557	IFIT2	protein_coding
0,983457	GPAM	protein_coding
0,2443691	CUTC	protein_coding
0,3178501	PPP1R3C	protein_coding
0,9792506	MXI1	protein_coding
0,997931	SMNDC1	protein_coding
0,3049645	TCTN3	protein_coding
0,6491869	DENND10	protein_coding
0,9484395	WDR11	protein_coding
0,4657211	ARMH3	protein_coding
0,091723	KCNIP2	protein_coding
0,4070535	GOT1	protein_coding
0,9781148	GNA13	protein_coding
0,5943942	KANSL1	protein_coding
0,1740802	DUSP1	protein_coding
0,9767278	PANK3	protein_coding
0,0895372	TEK	protein_coding
0,7996567	CAAP1	protein_coding
0,8881504	CD274	protein_coding
0,5537742	NUP43	protein_coding
0,2737133	PCMT1	protein_coding
0,2984194	PLEKHG1	protein_coding
0,4732706	MYCT1	protein_coding
0,505053	CYSTM1	protein_coding
0,9900815	WDR55	protein_coding
0,6988495	MRPS14	protein_coding
0,9587213	GORAB	protein_coding
0,3089259	TCP1	protein_coding
0,8221205	SNX19	protein_coding
0,4602818	KCNJ5	protein_coding
0,6023346	PDZD11	protein_coding
0,9465232	SLC10A7	protein_coding

0,855954	NUDCD1	protein_coding
0,4521055	ENY2	protein_coding
0,3829653	KIAA1217	protein_coding
0,1352102	PLXDC2	protein_coding
0,7984578	EPC1	protein_coding
0,721162	TAF12	protein_coding
0,6304019	MTRF1	protein_coding
0,2440137	DNAJC15	protein_coding
0,9954421	PROSER1	protein_coding
0,8648632	UFM1	protein_coding
0,2407669	WBP4	protein_coding
0,8189129	ELF1	protein_coding
0,195587	SMAD9	protein_coding
0,8885864	HSPH1	protein_coding
0,6128477	KBTBD7	protein_coding
0,9002008	ALG5	protein_coding
0,5180247	EXOSC8	protein_coding
0,7940001	ETF1	protein_coding
0,1916186	TGFBI	protein_coding
0,796666	FAM53C	protein_coding
0,8796725	SIL1	protein_coding
0,8930787	PAIP2	protein_coding
0,9528194	MYOT	protein_coding
0,3667541	KDM3B	protein_coding
0,5029234	EGR1	protein_coding
0,4859932	SERP1	protein_coding
0,9761152	ZFP30	protein_coding
0,8229361	NR2C1	protein_coding
0,9761152	UTP20	protein_coding
0,7352523	TMPO	protein_coding
0,8545769	ARL1	protein_coding
0,2337466	GLT8D2	protein_coding
0,7924243	MTERF2	protein_coding
0,9666028	SOCS2	protein_coding
0,9735049	NFYB	protein_coding
0,3447404	WASHC3	protein_coding
0,2834613	APAF1	protein_coding
0,8430111	CLU	protein_coding
0,624851	TNFRSF10B	protein_coding
0,7817948	PTK2B	protein_coding
0,6173428	PPP3CC	protein_coding
0,8489317	EPHX2	protein_coding
0,809839	RNF170	protein_coding
0,3078991	NPPB	protein_coding
0,9084344	TARDBP	protein_coding
0,9150264	ZNF706	protein_coding
0,5583085	LYPLA1	protein_coding
0,1642927	COPS5	protein_coding
0,9680053	RDH10	protein_coding
0,8575378	AKAP1	protein_coding
0,8373584	COIL	protein_coding
0,9538485	TRIM25	protein_coding
0,9226832	SCPEP1	protein_coding
0,8870441	SPOP	protein_coding
0,2281984	SLC35B1	protein_coding
0,5985056	TMEM131L	protein_coding
0,6425583	TENT4B	protein_coding
0,5442603	CEP89	protein_coding
0,8578793	ECHDC2	protein_coding

0,7237365	PLBD1	protein_coding
0,749866	PYROXD1	protein_coding
0,9142106	KCNJ8	protein_coding
0,4684539	PSPC1	protein_coding
0,9089634	ZNF549	protein_coding
0,5024248	ZSCAN18	protein_coding
0,9312186	ZNF211	protein_coding
0,520749	PDZRN3	protein_coding
0,9840915	RNF2	protein_coding
0,9998478	TRMT1L	protein_coding
0,9277595	SEC22A	protein_coding
0,7645994	POPDC2	protein_coding
0,5485366	B4GALT4	protein_coding
0,8901085	NAA50	protein_coding
0,9484395	DESI2	protein_coding
0,7670701	CRY2	protein_coding
0,5953122	CAT	protein_coding
0,8114908	ZMYM2	protein_coding
0,6254273	GJA3	protein_coding
0,9618453	TBC1D15	protein_coding
0,8575378	ZCCHC17	protein_coding
0,7168957	FABP3	protein_coding
0,7301898	KHDRBS1	protein_coding
0,855954	POLR3GL	protein_coding
0,9007353	TNFSF10	protein_coding
0,6591549	ZNF639	protein_coding
0,9894339	PIK3CA	protein_coding
0,9854182	PDS5A	protein_coding
0,5752656	LIAS	protein_coding
0,3004311	CPXM2	protein_coding
0,5557127	LRIF1	protein_coding
0,8786805	CLCC1	protein_coding
0,0953127	GPSM2	protein_coding
0,9840915	GTDC1	protein_coding
0,9484395	ZRANB3	protein_coding
0,8367243	ACVR2A	protein_coding
0,8131645	POLK	protein_coding
0,9840915	RPL21	protein_coding
0,0197802	MTIF3	protein_coding
0,0132445	GTF3A	protein_coding
0,855954	UBL3	protein_coding
0,9366634	FYTTD1	protein_coding
0,838064	MTERF4	protein_coding
0,3339605	OCRL	protein_coding
0,0197802	FMOD	protein_coding
0,9994264	KIAA1191	protein_coding
0,1800952	COPA	protein_coding
0,8212084	RBBP6	protein_coding
0,8373546	ZC3H7A	protein_coding
0,8871625	SERAC1	protein_coding
0,7467854	ANXA11	protein_coding
0,7774855	LDB3	protein_coding
0,5879554	SHLD2	protein_coding
0,2945575	PRXL2A	protein_coding
0,7956173	RPL5	protein_coding
0,7528871	ODF2L	protein_coding
0,1131434	PTGFR	protein_coding
0,8930787	TRMT13	protein_coding
0,5029234	LRRC39	protein_coding

0,6867028	ZNF644	protein_coding
0,7049962	CCDC18	protein_coding
0,9439974	RPAP2	protein_coding
0,4161706	BBS9	protein_coding
0,864196	PMS2	protein_coding
0,9998478	SEPTIN7	protein_coding
0,4175635	KLHL7	protein_coding
0,3792977	HERPUD2	protein_coding
0,7238799	CBX3	protein_coding
0,3748795	HNRNPA2B1	protein_coding
0,8863508	FAM126A	protein_coding
0,8117718	FKBP9	protein_coding
0,8373584	NT5C3A	protein_coding
0,8258095	ARL4A	protein_coding
0,692146	CCZ1	protein_coding
0,7385573	RAMP3	protein_coding
0,8545769	SMU1	protein_coding
0,9359033	SLC25A51	protein_coding
0,9296784	CLTA	protein_coding
0,6337897	RECK	protein_coding
0,9781971	ACO1	protein_coding
0,9067454	DCAF10	protein_coding
0,8189129	TRIM24	protein_coding
0,7065438	CALD1	protein_coding
0,1336905	PLAU	protein_coding
0,7154026	SRGN	protein_coding
0,6604792	CHST3	protein_coding
0,5847346	BICC1	protein_coding
0,2948143	CISD1	protein_coding
0,8238032	ECD	protein_coding
0,7135335	P4HA1	protein_coding
0,5709725	SLC25A16	protein_coding
0,9998538	VPS26A	protein_coding
0,8641364	RBM19	protein_coding
0,8992483	IFT81	protein_coding
0,9952606	MED13L	protein_coding
0,8578622	CDKN2C	protein_coding
0,9476644	RNF11	protein_coding
0,9312186	RASSF8	protein_coding
0,0185612	BHLHE41	protein_coding
0,9296784	SSPN	protein_coding
0,8868311	ITPR2	protein_coding
0,9825766	CCDC91	protein_coding
0,6852472	NECAB1	protein_coding
0,4311333	WWP1	protein_coding
0,7118106	ACOT9	protein_coding
0,6491869	PRDX4	protein_coding
0,9174724	PKN1	protein_coding
0,8578793	TRIR	protein_coding
0,9781971	ADGRE5	protein_coding
0,9803555	GIPC1	protein_coding
0,6776989	SPRYD7	protein_coding
0,0943087	ATP7B	protein_coding
0,3883821	ZC3H13	protein_coding
0,8578622	NLN	protein_coding
0,8311093	OPTN	protein_coding
0,4775021	ITIH5	protein_coding
0,6084958	ATF1	protein_coding
0,7912354	NCKAP1L	protein_coding

0,8565534	PFDN5	protein_coding
0,6644356	SPATS2	protein_coding
0,3943278	NR4A1	protein_coding
0,9465232	LRP1	protein_coding
0,8259599	TUBA1B	protein_coding
0,8209243	ATPAF1	protein_coding
0,3078991	AMD1	protein_coding
0,4562339	NDUFAF4	protein_coding
0,7444016	USP45	protein_coding
0,0736295	PLP1	protein_coding
0,8682799	MORF4L2	protein_coding
0,8259599	RAB9B	protein_coding
0,9614756	FAM199X	protein_coding
0,9953531	RAB9A	protein_coding
0,690748	METTL8	protein_coding
0,685088	TTC21B	protein_coding
0,9410289	BAZ2B	protein_coding
0,9080545	LPGAT1	protein_coding
0,8219707	G0S2	protein_coding
0,764019	RAP2C	protein_coding
0,6961817	EXOSC9	protein_coding
0,8218459	PLA2G12A	protein_coding
0,8868311	PFKFB2	protein_coding
0,9944531	AGO2	protein_coding
0,3218826	MXD4	protein_coding
0,9074092	ACSL3	protein_coding
0,3316892	CHPF	protein_coding
0,8847411	DNPEP	protein_coding
0,3697716	OBSL1	protein_coding
0,6003589	SLC12A4	protein_coding
0,9615223	FAM210B	protein_coding
0,5998801	TTPAL	protein_coding
0,7638819	PREX1	protein_coding
0,3894807	SDC4	protein_coding
0,7506449	NCOA3	protein_coding
0,9481776	PIGT	protein_coding
0,9703483	NCOA5	protein_coding
0,9998478	VAPB	protein_coding
0,2915153	ATP5F1E	protein_coding
0,7467854	CHD6	protein_coding
0,5164856	PLCG1	protein_coding
0,9792506	SRSF6	protein_coding
0,998169	ARFGEF2	protein_coding
0,4521055	ZNFX1	protein_coding
0,5647201	CSE1L	protein_coding
0,5994444	RAB22A	protein_coding
0,4847278	PTGIS	protein_coding
0,6285359	STAU1	protein_coding
0,5964345	STX16	protein_coding
0,9738194	PMEPA1	protein_coding
0,9368504	RNF114	protein_coding
0,9804708	DDX27	protein_coding
0,59951	MTRR	protein_coding
0,6383874	PEPD	protein_coding
0,9738194	VAMP7	protein_coding
0,7104968	STAMBP	protein_coding
0,9792506	NAGK	protein_coding
0,7237365	MCEE	protein_coding
0,8062191	PAIP2B	protein_coding

0,6564675	SNRNP27	protein_coding
0,9528194	MPHOSPH10	protein_coding
0,9312186	ATP8A1	protein_coding
0,441827	USP22	protein_coding
0,8930787	POF1B	protein_coding
0,9894339	HIF3A	protein_coding
0,9781971	ZNF45	protein_coding
0,931978	USP9X	protein_coding
0,0015119	F13A1	protein_coding
0,6333516	SIRT5	protein_coding
0,624851	MRS2	protein_coding
0,8603658	WRNIP1	protein_coding
0,4344705	RRP36	protein_coding
0,2217718	SNRPC	protein_coding
0,0730772	SERPINB6	protein_coding
0,8854446	XPO5	protein_coding
0,3459301	NQO2	protein_coding
0,9450135	OARD1	protein_coding
0,9465232	RPS10	protein_coding
0,6225174	MOCS1	protein_coding
0,4237568	MED20	protein_coding
0,4761054	MAD2L1BP	protein_coding
0,3212727	APOBEC2	protein_coding
0,9618453	KLHDC3	protein_coding
0,9628671	MEA1	protein_coding
0,6212456	KLHL31	protein_coding
0,0868691	COL21A1	protein_coding
0,95402	CDKN1A	protein_coding
0,9602183	SOX4	protein_coding
0,4608748	GLO1	protein_coding
0,7490465	CPNE5	protein_coding
0,9798738	RREB1	protein_coding
0,7444016	SSR1	protein_coding
0,9962215	RIOK1	protein_coding
0,7352523	NRN1	protein_coding
0,8086025	SLC35B3	protein_coding
0,4638153	ATXN1	protein_coding
0,8547943	NUP153	protein_coding
0,4724079	DEK	protein_coding
0,4601677	LRRFIP1	protein_coding
0,5046858	AHNAK	protein_coding
0,1712026	EMC3	protein_coding
0,7546576	CNOT1	protein_coding
0,9007353	BBS2	protein_coding
0,645515	MT2A	protein_coding
0,6604792	GOT2	protein_coding
0,9628671	RAP2A	protein_coding
0,9893144	ABCC4	protein_coding
0,3881918	EFNB2	protein_coding
0,8834615	TM9SF2	protein_coding
0,9033043	IRF1	protein_coding
0,5180247	SEPTIN6	protein_coding
0,1326966	NDUFA1	protein_coding
0,8377134	DMAC2L	protein_coding
0,9925374	FAM193A	protein_coding
0,2841212	SOX9	protein_coding
0,2560075	MRPS7	protein_coding
0,9450135	GGA3	protein_coding
0,6305527	NUP85	protein_coding

0,9900815	MSTO1	protein_coding
0,4835164	TTF1	protein_coding
0,7477503	GTF3C4	protein_coding
0,9484395	PPP1R12C	protein_coding
0,9768456	INSIG2	protein_coding
0,9572126	POLR1B	protein_coding
0,9039795	CCDC93	protein_coding
0,9086664	SLC25A23	protein_coding
0,8221205	GTF2F1	protein_coding
0,7412353	THOC2	protein_coding
0,9356716	MED1	protein_coding
0,9533676	RPL23	protein_coding
0,9700303	ATG4C	protein_coding
0,5558351	C3	protein_coding
0,7122873	TRIP10	protein_coding
0,5062136	GPR108	protein_coding
0,6567534	OPA3	protein_coding
0,7740102	SNRPD2	protein_coding
0,3989779	VASP	protein_coding
0,8908494	SYMPK	protein_coding
0,9899185	GPCPD1	protein_coding
0,9628671	PANK2	protein_coding
0,8695843	CD93	protein_coding
0,9410184	GZF1	protein_coding
0,989732	NAPB	protein_coding
0,9081173	CENPB	protein_coding
0,6914529	PSMF1	protein_coding
0,7576959	RBCK1	protein_coding
0,2690459	TMX4	protein_coding
0,8665655	STK35	protein_coding
0,9828457	SNRPB	protein_coding
0,9485776	RRBP1	protein_coding
0,8623038	ZNF133	protein_coding
0,1501348	MKKS	protein_coding
0,5688668	DSTN	protein_coding
0,623981	SNRPB2	protein_coding
0,8721281	MGME1	protein_coding
0,6653209	TBC1D20	protein_coding
0,943695	HNRNPR	protein_coding
0,5474737	ZNF436	protein_coding
0,8727034	MAX	protein_coding
0,3943278	ID1	protein_coding
0,997923	RALY	protein_coding
0,445647	EIF2S2	protein_coding
0,8875885	ERGIC3	protein_coding
0,8593223	ROMO1	protein_coding
0,4378091	CEP250	protein_coding
0,9893144	KDM5C	protein_coding
0,3715255	AMOT	protein_coding
0,4835164	PSMB2	protein_coding
0,9540553	AGO3	protein_coding
0,9156816	UROD	protein_coding
0,8647372	HECTD3	protein_coding
0,9975676	KLC1	protein_coding
0,9956922	TUBGCP3	protein_coding
0,7095541	MCF2L	protein_coding
0,6613295	PCID2	protein_coding
0,8206562	CAPNS1	protein_coding
0,8688662	UBA2	protein_coding

0,1735916	COX6B1	protein_coding
0,4593782	THRA	protein_coding
0,5925149	PRDX5	protein_coding
0,7713242	PRMT1	protein_coding
0,8992483	RRAS	protein_coding
0,9610275	SBDS	protein_coding
0,9771336	STAT5A	protein_coding
0,6023346	BECN1	protein_coding
0,9594887	TRAP1	protein_coding
0,9645423	NSRP1	protein_coding
0,7065438	DNAJC8	protein_coding
0,1064872	IFI6	protein_coding
0,5175488	EMG1	protein_coding
0,7165662	UXT	protein_coding
0,8269408	PCNX4	protein_coding
0,7118106	ATG14	protein_coding
0,6088362	KTN1	protein_coding
0,8238032	RHOJ	protein_coding
0,8941532	L3HYPDH	protein_coding
0,1232731	HSPA2	protein_coding
0,5895248	ZBTB1	protein_coding
0,998169	TRMT5	protein_coding
0,9296784	SGPP1	protein_coding
0,5416343	PLEKHG3	protein_coding
0,6838951	RHOT1	protein_coding
0,6316303	WDR60	protein_coding
0,09831	AIF1L	protein_coding
0,6184079	FAM78A	protein_coding
0,9374881	NUP214	protein_coding
0,7740102	MAP2K2	protein_coding
0,8148845	HNRNPH2	protein_coding
0,624851	ARMCX1	protein_coding
0,7321942	CANX	protein_coding
0,933129	INTS11	protein_coding
0,6591549	ZNF484	protein_coding
0,0908653	OMD	protein_coding
0,9359827	PPCS	protein_coding
0,2205761	COX7C	protein_coding
0,3867348	MASP1	protein_coding
0,9296784	HELB	protein_coding
0,8786805	RAP1B	protein_coding
0,2506935	RAB3IP	protein_coding
0,4835164	PTPRB	protein_coding
0,9536474	DYRK2	protein_coding
0,8545769	PIN1	protein_coding
0,9769756	EMC1	protein_coding
0,554525	PLA2G5	protein_coding
0,9928407	UBR4	protein_coding
0,5269672	HP1BP3	protein_coding
0,6988495	SIN3B	protein_coding
0,933129	SLC35E1	protein_coding
0,8996732	EPS15L1	protein_coding
0,4246004	UQCR11	protein_coding
0,964785	MACF1	protein_coding
0,9781971	SMARCA4	protein_coding
0,9998478	KDM4B	protein_coding
0,7230802	METTL25	protein_coding
0,7452363	METTL16	protein_coding
0,4321437	TUBA4A	protein_coding

0,5249547	AAMP	protein_coding
0,6613295	PNKD	protein_coding
0,0222168	TNFRSF19	protein_coding
0,9582115	RNF6	protein_coding
0,9391446	ECHS1	protein_coding
0,8194018	AKAP9	protein_coding
0,3943278	GNG11	protein_coding
0,2644142	SEM1	protein_coding
0,9700303	HIP1	protein_coding
0,9998478	PTPN12	protein_coding
0,9628671	POR	protein_coding
0,4593782	FGL2	protein_coding
0,1067405	STEAP4	protein_coding
0,8117718	GNAI1	protein_coding
0,7467854	PEX1	protein_coding
0,8441592	MTERF1	protein_coding
0,8474324	SGCE	protein_coding
0,9918615	RBM48	protein_coding
0,7114715	CASD1	protein_coding
0,7972313	ZNF780B	protein_coding
0,9997651	ZFP36	protein_coding
0,6093605	PAICS	protein_coding
0,445647	KDR	protein_coding
0,615864	TUBGCP6	protein_coding
0,9551108	DGCR6L	protein_coding
0,9998538	DGCR8	protein_coding
0,8812706	YWHAH	protein_coding
0,4532937	ATF4	protein_coding
0,592905	APOL3	protein_coding
0,3358601	TPST2	protein_coding
0,4761054	MPST	protein_coding
0,5060008	APOL2	protein_coding
0,5754389	EMC4	protein_coding
0,3167153	SPECC1	protein_coding
0,8695843	DOCK4	protein_coding
0,6383874	POT1	protein_coding
0,4628071	ATP6V1F	protein_coding
0,4562339	LSM8	protein_coding
0,5413406	PODXL	protein_coding
0,8240849	STRIP2	protein_coding
0,9379013	IFT22	protein_coding
0,6188889	MKLN1	protein_coding
0,8829558	DNAJB9	protein_coding
0,9108829	FLNC	protein_coding
0,5456715	CALU	protein_coding
0,6238595	KLHDC10	protein_coding
0,2807832	NDUFA5	protein_coding
0,9454316	MYO1B	protein_coding
0,1856054	MTX2	protein_coding
0,6568293	EIF2S2P4	processed_pseudogene
0,6613817	OSGEPL1	protein_coding
0,9463928	ORMDL1	protein_coding
0,3358222	HAT1	protein_coding
0,8281575	HERC2	protein_coding
0,9007686	SNRPN	protein_coding
0,9731251	PSMG2	protein_coding
0,5367932	TWSG1	protein_coding
0,8283953	EIF2AK4	protein_coding
0,1232731	MYO5C	protein_coding

0,2945575	CGNL1	protein_coding
0,8238032	TMOD2	protein_coding
0,8189129	TTBK2	protein_coding
0,9368504	INO80	protein_coding
0,9358987	ICE2	protein_coding
0,0286685	ALDH1A2	protein_coding
0,9678404	MINDY2	protein_coding
0,7362379	IVD	protein_coding
0,9966756	DUT	protein_coding
0,9142106	ARPP19	protein_coding
0,8545769	VPS13C	protein_coding
0,3358222	ISLR	protein_coding
0,7892243	ANAPC13	protein_coding
0,9644268	MBD4	protein_coding
0,9962215	COPB1	protein_coding
0,6383874	PSMA1	protein_coding
0,5675917	SUMF2	protein_coding
0,6120803	PALLD	protein_coding
0,9282881	SPCS3	protein_coding
0,2915153	CSRP3	protein_coding
0,115935	DCTD	protein_coding
0,7109532	RPAIN	protein_coding
0,4886257	TXNDC17	protein_coding
0,6750523	FXR2	protein_coding
0,8492407	KIF1C	protein_coding
0,3089259	MPDU1	protein_coding
0,8269408	PHF20L1	protein_coding
0,8825313	CCNT1	protein_coding
0,9975676	PUS7L	protein_coding
0,9738194	ILF3	protein_coding
0,9998478	SLC44A2	protein_coding
0,7362379	MTUS1	protein_coding
0,6184746	NGDN	protein_coding
0,5328077	ADCY4	protein_coding
0,6613295	RAB2B	protein_coding
0,4602818	BCL2L2	protein_coding
0,9536474	PARP2	protein_coding
0,9976739	HEATR5A	protein_coding
0,8050685	SNX6	protein_coding
0,8545769	EAPP	protein_coding
0,9761718	EGLN3	protein_coding
0,8623038	MIS18BP1	protein_coding
0,2443691	RNASE1	protein_coding
0,4638153	NEDD8	protein_coding
0,624851	DAD1	protein_coding
0,9894339	TEP1	protein_coding
0,5559368	EPB41L4A	protein_coding
0,8318664	CDO1	protein_coding
0,5224131	REEP5	protein_coding
0,8117718	ITFG1	protein_coding
0,8971058	SEC14L1	protein_coding
0,8219774	ARHGEF6	protein_coding
0,7302504	MAP7D3	protein_coding
0,8373584	ASH2L	protein_coding
0,914514	CDKN1C	protein_coding
0,7885463	RPS4Y1	protein_coding
0,5879554	MAU2	protein_coding
0,9998538	TNNI3	protein_coding
0,2164941	PUDP	protein_coding

0,993752	ERMARD	protein_coding
0,8721281	PHF10	protein_coding
0,6254273	KCNA5	protein_coding
0,6462207	STARD8	protein_coding
0,2945575	FAM155B	protein_coding
0,7977187	SAT1	protein_coding
0,7230802	GNL3L	protein_coding
0,5453655	SH3BP4	protein_coding
0,6173428	MOSPD2	protein_coding
0,7894641	DOCK6	protein_coding
0,5400662	ECSIT	protein_coding
0,6613295	LDLR	protein_coding
0,8117718	PRKCSH	protein_coding
0,7506449	CNN1	protein_coding
0,8176511	CDC16	protein_coding
0,8212084	NECTIN2	protein_coding
0,6098976	APOE	protein_coding
0,7357059	LRCH2	protein_coding
0,5777776	XPO7	protein_coding
0,7242009	SAFB2	protein_coding
0,6527079	RPL36	protein_coding
0,9699064	KIF1A	protein_coding
0,9792506	PLVAP	protein_coding
0,7433064	SLC27A1	protein_coding
0,6287358	COLGALT1	protein_coding
0,330331	MRPL34	protein_coding
0,9183994	TULP4	protein_coding
0,8941532	SNX9	protein_coding
0,1326966	RTN4IP1	protein_coding
0,1482066	QRSL1	protein_coding
0,290302	MTRES1	protein_coding
0,3765746	RSPH3	protein_coding
0,8810791	AFDN	protein_coding
0,8930787	ACTN4	protein_coding
0,8555218	STK33	protein_coding
0,5647201	NDUFA10	protein_coding
0,7349798	ZSWIM6	protein_coding
0,8908494	PXDN	protein_coding
0,4724079	PGPEP1	protein_coding
0,8318664	LSM4	protein_coding
0,7296863	JUND	protein_coding
0,4157638	HRC	protein_coding
0,7876407	ZNF557	protein_coding
0,3105619	CAMSAP1	protein_coding
0,3830886	UBAC1	protein_coding
0,3435348	H19	lncRNA
0,7213265	COL5A1	protein_coding
0,9312186	ATXN10	protein_coding
0,965142	TUBGCP2	protein_coding
0,9954421	CEP85	protein_coding
0,9884562	LAMA5	protein_coding
0,5847346	OSBPL2	protein_coding
0,9773277	ASS1	protein_coding
0,8698319	EXOSC2	protein_coding
0,6491869	POMT1	protein_coding
0,7713242	PRRC2B	protein_coding
0,7213265	CHMP2A	protein_coding
0,4450378	UBE2M	protein_coding
0,768388	TRIM28	protein_coding

0,6999614	EIF2S3	protein_coding
0,4911389	ZC3H4	protein_coding
0,9998538	LRRC47	protein_coding
0,6613295	ATP5IF1	protein_coding
0,9062598	CLIP1	protein_coding
0,9183247	HIP1R	protein_coding
0,9998478	ZNF317	protein_coding
0,8269408	EIF3G	protein_coding
0,5622598	DNMT1	protein_coding
0,4835164	ZNF426	protein_coding
0,9481776	SLC6A8	protein_coding
0,6559545	DKC1	protein_coding
0,103738	MPP1	protein_coding
0,2281984	ZNF331	protein_coding
0,997931	ZNF236	protein_coding
0,517991	NOL11	protein_coding
0,7823793	UBE4B	protein_coding
0,9166492	CASZ1	protein_coding
0,9960707	HABP4	protein_coding
0,6591549	PRRG1	protein_coding
0,8105839	UBA1	protein_coding
0,6203109	RGN	protein_coding
0,9998478	TXLNGY	transcribed_unprocessed_pseudogene
0,7805908	PPIL4	protein_coding
0,8850351	AKAP12	protein_coding
0,5456715	SYNE1	protein_coding
0,817424	LATS1	protein_coding
0,8992483	AAR2	protein_coding
0,8833312	RBM39	protein_coding
0,4360201	ACSS2	protein_coding
0,4810647	ARHGEF9	protein_coding
0,3218826	ATP6V1E1	protein_coding
0,9669517	ZNF227	protein_coding
0,7258655	ZNF141	protein_coding
0,3779824	COX4I1	protein_coding
0,6184079	EMC8	protein_coding
0,8017181	GSE1	protein_coding
0,8996562	CHMP1A	protein_coding
0,931978	SH3BGRL	protein_coding
0,1129731	COX7B	protein_coding
0,8575378	CAP1	protein_coding
0,9368504	PPT1	protein_coding
0,9544158	RLIM	protein_coding
0,5175488	ABCB7	protein_coding
0,3386759	TRAF3	protein_coding
0,0286685	MRPS25	protein_coding
0,8704958	HACL1	protein_coding
0,6594711	TBC1D5	protein_coding
0,9406097	CAPN7	protein_coding
0,7915374	RFTN1	protein_coding
0,5474737	RBSN	protein_coding
0,2393106	GALNT15	protein_coding
0,2281984	SLC6A6	protein_coding
0,9997651	NR1H2	protein_coding
0,9982092	KIF3A	protein_coding
0,8870682	MGAT1	protein_coding
0,7132753	GFPT2	protein_coding
0,5011485	PSME3	protein_coding
0,9951314	RPL27	protein_coding

0,6557182	AOC3	protein_coding
0,284683	ACLY	protein_coding
0,66699	VPS25	protein_coding
0,6403073	NDUFA2	protein_coding
0,9729242	DIAPH1	protein_coding
0,7027821	NDFIP1	protein_coding
0,6448972	UBE2D2	protein_coding
0,6287358	EXOC4	protein_coding
0,3903182	ANO1	protein_coding
0,7444016	PPFIA1	protein_coding
0,4602818	MAP1B	protein_coding
0,5317229	IL13RA1	protein_coding
0,54642	WDR44	protein_coding
0,452318	CKMT2	protein_coding
0,8885864	ZCCHC9	protein_coding
0,9781148	PEX11B	protein_coding
0,8545769	PRKAB2	protein_coding
0,176447	PDHA1	protein_coding
0,6383874	MCCC2	protein_coding
0,7558125	ZNF304	protein_coding
0,9709611	ZNF132	protein_coding
0,6903585	SELENOS	protein_coding
0,7027821	CHSY1	protein_coding
0,3194617	SNRPA1	protein_coding
0,7490465	THAP1	protein_coding
0,3801685	C19orf12	protein_coding
0,4675238	ACTR10	protein_coding
0,9007686	LGALS3	protein_coding
0,0546553	DNAJB1	protein_coding
0,9789395	SPATA6	protein_coding
0,7816267	LRRC41	protein_coding
0,9802452	DHX30	protein_coding
0,3358222	RAF1	protein_coding
0,8851892	PPARG	protein_coding
0,582571	ENOSF1	protein_coding
0,2663408	EMILIN2	protein_coding
0,7924243	ARFIP2	protein_coding
0,7190863	TRIM22	protein_coding
0,8189129	RRP8	protein_coding
0,9702772	TIMM10B	protein_coding
0,8930787	EFR3A	protein_coding
0,9363291	PTCD3	protein_coding
0,6238595	IMMT	protein_coding
0,5943287	MRPL35	protein_coding
0,985846	ILKAP	protein_coding
0,0465925	PER2	protein_coding
0,5020851	RAMP1	protein_coding
0,6137496	PTPRE	protein_coding
0,6383874	RAN	protein_coding
0,6659037	PRKAA1	protein_coding
0,9439974	CARD6	protein_coding
0,1412577	RAP1GAP2	protein_coding
0,9768456	CLUH	protein_coding
0,8934536	INPP5K	protein_coding
0,940196	RPA1	protein_coding
0,5777334	SERPINF1	protein_coding
0,9738194	UBE2G1	protein_coding
0,9660499	TBC1D14	protein_coding
0,9209064	TMEM128	protein_coding

0,1356666	COQ3	protein_coding
0,9481776	PNISR	protein_coding
0,2281984	POPDC3	protein_coding
0,4835164	SEC61G	protein_coding
0,9839612	LANCL2	protein_coding
0,8131645	FIGNL1	protein_coding
0,2492279	GRSF1	protein_coding
0,931978	ANKRD17	protein_coding
0,9206489	UTP3	protein_coding
0,7174713	WBP2	protein_coding
0,9528743	H3F3B	protein_coding
0,8553465	UNK	protein_coding
0,7671658	ZRANB2	protein_coding
0,6446908	EIF5A	protein_coding
0,5318944	XAF1	protein_coding
0,624851	RIDA	protein_coding
0,8565534	VPS13B	protein_coding
0,4045325	MATN2	protein_coding
0,9488066	PCBD2	protein_coding
0,5248895	SDF2	protein_coding
0,7506449	FLOT2	protein_coding
0,7673893	PRMT7	protein_coding
0,7558125	TERF2	protein_coding
0,8653179	VPS4A	protein_coding
0,7609122	BTBD3	protein_coding
0,6624016	PCNA	protein_coding
0,9919804	POLR3F	protein_coding
0,9700303	RIN2	protein_coding
0,1712026	PTPRA	protein_coding
0,5847346	DAP3	protein_coding
0,8661642	KHDC4	protein_coding
0,5402971	NES	protein_coding
0,9169269	ARHGEF11	protein_coding
0,8318538	DCAF8	protein_coding
0,8318519	SYT11	protein_coding
0,5847346	MMACHC	protein_coding
0,7972313	NASP	protein_coding
0,5347705	CTNNBL1	protein_coding
0,4073279	RBM38	protein_coding
0,4971178	VSTM2L	protein_coding
0,9998478	OSER1	protein_coding
0,7921707	SERINC3	protein_coding
0,2184644	BHMT2	protein_coding
0,6873469	AP3B1	protein_coding
0,4422012	PATJ	protein_coding
0,8721867	DCTN4	protein_coding
0,7054786	MTUS2	protein_coding
0,9492532	ZMYM5	protein_coding
0,9444553	USPL1	protein_coding
0,9528743	XPO4	protein_coding
0,2148035	POMP	protein_coding
0,54642	CDK8	protein_coding
0,9439974	HMGB1P5	transcribed_processed_pseudogene
0,7146386	WASF3	protein_coding
0,0469777	MYH10	protein_coding
0,6986378	SCO1	protein_coding
0,7010516	MPRIP	protein_coding
0,9450135	PIK3C2B	protein_coding
0,8538999	DSTYK	protein_coding

0,6563172	SLC41A1	protein_coding
0,1367817	DCLK1	protein_coding
0,997931	COG6	protein_coding
0,8416675	SPART	protein_coding
0,1667775	POSTN	protein_coding
0,9341044	TPT1	protein_coding
0,8812706	GPALPP1	protein_coding
0,1524221	STARD13	protein_coding
0,2687129	MORC4	protein_coding
0,4888513	TBC1D8B	protein_coding
0,7393702	TCEAL4	protein_coding
0,7444016	SRRM1	protein_coding
0,7431973	SLF1	protein_coding
0,6689227	CNDP2	protein_coding
0,9946475	RTN3	protein_coding
0,9994264	MYH11	protein_coding
0,8474324	FOPNL	protein_coding
0,9907221	PDZD2	protein_coding
0,9348712	MORC2	protein_coding
0,9551108	LARGE1	protein_coding
0,7611852	MYO18B	protein_coding
0,6988495	SLC2A11	protein_coding
0,6787299	GIMAP6	protein_coding
0,7894641	GIMAP4	protein_coding
0,7126808	MKRN1	protein_coding
0,6776791	ZNF767P	transcribed_unprocessed_pseudogene
0,5675917	BTG1	protein_coding
0,9439974	C12orf29	protein_coding
0,5980078	ATP13A3	protein_coding
0,1232731	TMTC1	protein_coding
0,7743102	KRAS	protein_coding
0,6527079	IPO8	protein_coding
0,7820244	LARS	protein_coding
0,4787761	IMPA1	protein_coding
0,6620763	LRRCC1	protein_coding
0,8877092	CCDC59	protein_coding
0,8062191	SWAP70	protein_coding
0,0088417	ARNTL	protein_coding
0,0247233	LYVE1	protein_coding
0,8885864	SBF2	protein_coding
0,0336588	MICAL2	protein_coding
0,7431973	RRAS2	protein_coding
0,5040596	HSD17B4	protein_coding
0,7687197	ZFC3H1	protein_coding
0,5415916	SARAF	protein_coding
0,9296784	DUSP26	protein_coding
0,8992483	DPF2	protein_coding
0,9438763	DGLUCY	protein_coding
0,9424246	NUMB	protein_coding
0,9848567	MED6	protein_coding
0,4672216	EIF2S1	protein_coding
0,9496064	ELP3	protein_coding
0,5679388	PEBP4	protein_coding
0,7576959	CTIF	protein_coding
0,9362082	MBD2	protein_coding
0,3994613	IER3IP1	protein_coding
0,284683	MRPS36	protein_coding
0,4684923	THUMPD3	protein_coding
0,747138	BHLHE40	protein_coding

0,9975676	ARL8B	protein_coding
0,3929033	EDEM1	protein_coding
0,0473067	CHL1	protein_coding
0,4765298	MEIS2	protein_coding
0,6613295	DPH6	protein_coding
0,9772493	KATNBL1	protein_coding
0,1520655	EMC7	protein_coding
0,9776997	PRPF38B	protein_coding
0,5015091	GSTM3	protein_coding
0,9583561	SORT1	protein_coding
0,8823913	WNT2B	protein_coding
0,792656	PTGFRN	protein_coding
0,2205761	LAMTOR5	protein_coding
0,4222683	NOTCH2	protein_coding
0,7820981	TRIM45	protein_coding
0,9142106	CEPT1	protein_coding
0,9277595	AP4B1	protein_coding
0,8632902	NAPG	protein_coding
0,4835164	SPIRE1	protein_coding
0,8270848	PPHLN1	protein_coding
0,5474737	ARF3	protein_coding
0,6664224	TMEM106C	protein_coding
0,824287	SLC38A2	protein_coding
0,1140347	YWHAQ	protein_coding
0,3803906	KIDINS220	protein_coding
0,9387004	ROCK2	protein_coding
0,9463928	RSAD2	protein_coding
0,6867028	LPIN1	protein_coding
0,7820244	IAH1	protein_coding
0,2535482	LDHA	protein_coding
0,9625694	IL6ST	protein_coding
0,9977992	NAV1	protein_coding
0,9118315	CDC73	protein_coding
0,0488716	TIMM17A	protein_coding
0,768388	RPS15A	protein_coding
0,9377639	NARS	protein_coding
0,845681	RELCH	protein_coding
0,922549	FBH1	protein_coding
0,2533818	RBM17	protein_coding
0,9953531	ECHDC3	protein_coding
0,5506008	CCNH	protein_coding
0,8597029	KCTD1	protein_coding
0,887319	DOCK2	protein_coding
0,9802452	EMP1	protein_coding
0,5647201	SOX5	protein_coding
0,9484395	LRP4	protein_coding
0,9854182	MYBPC3	protein_coding
0,8166471	ACP2	protein_coding
0,9166492	RTL8C	protein_coding
0,9925374	RBMX2	protein_coding
0,6059615	PUM1	protein_coding
0,1869543	YARS	protein_coding
0,4311333	PHC2	protein_coding
0,7368645	GNL2	protein_coding
0,9997554	AGO4	protein_coding
0,8831297	HOOK1	protein_coding
0,8882787	CYP2J2	protein_coding
0,8674207	BTF3L4	protein_coding
0,9533676	TUT4	protein_coding

0,9215449	PRPF38A	protein_coding
0,4562339	DSC2	protein_coding
0,931978	RNF138	protein_coding
0,6591549	ELP2	protein_coding
0,9237476	DSC1	protein_coding
0,2945575	DTNA	protein_coding
0,9792506	FHOD3	protein_coding
0,9364287	TPGS2	protein_coding
0,330331	SLC43A3	protein_coding
0,8786805	APLNR	protein_coding
0,4175635	FADS2	protein_coding
0,8198317	TMEM258	protein_coding
0,7320389	TMEM165	protein_coding
0,7968733	CLOCK	protein_coding
0,673546	PDGFRA	protein_coding
0,8992483	COL4A2	protein_coding
0,5060296	DZIP1	protein_coding
0,8422988	UBAC2	protein_coding
0,8076343	ARGLU1	protein_coding
0,7190863	BIVM	protein_coding
0,7095541	TPP2	protein_coding
0,8189129	CARS2	protein_coding
0,6337897	ARHGAP32	protein_coding
0,9170247	STT3A	protein_coding
0,8255851	ETS1	protein_coding
0,8189556	TMED7	protein_coding
0,916788	APC	protein_coding
0,7444016	NREP	protein_coding
0,8626487	WDR36	protein_coding
0,9131236	OSTF1	protein_coding
0,6727601	RFK	protein_coding
0,8487116	UBQLN1	protein_coding
0,90504	NAA35	protein_coding
0,7740102	ANXA1	protein_coding
0,0336588	CTSL	protein_coding
0,7546576	CEMIP2	protein_coding
0,3706065	AGTPBP1	protein_coding
0,3435348	GOLM1	protein_coding
0,9670362	FAM189A2	protein_coding
0,6305527	ISCA1	protein_coding
0,7558125	ADAM19	protein_coding
0,8076343	TAOK3	protein_coding
0,5768837	USP30	protein_coding
0,9312186	FBXO21	protein_coding
0,0473388	TBX3	protein_coding
0,4612267	TRAFD1	protein_coding
0,8440006	DMTF1	protein_coding
0,8901085	CCDC146	protein_coding
0,6662301	CD36	protein_coding
0,6475905	PNPLA8	protein_coding
0,8865445	RINT1	protein_coding
0,583756	SRPK2	protein_coding
0,3089259	TES	protein_coding
0,4495398	MDFIC	protein_coding
0,8561022	MTO1	protein_coding
0,8670918	ANKRD6	protein_coding
0,6157478	CEP162	protein_coding
0,8941532	SYNCRIP	protein_coding
0,8289966	SNX14	protein_coding

0,3202177	NT5E	protein_coding
0,9406097	EPHA7	protein_coding
0,9804708	AKIRIN2	protein_coding
0,7740102	ORC3	protein_coding
0,7763012	LCA5	protein_coding
0,9803555	MAP3K7	protein_coding
0,933129	PHF21A	protein_coding
0,9064117	NAT10	protein_coding
0,6776791	CAPRIN1	protein_coding
0,3803906	ATP5MC2	protein_coding
0,7095541	CD63	protein_coding
0,305334	GDF11	protein_coding
0,8495694	ITGA7	protein_coding
0,7558125	BLOC1S1	protein_coding
0,4957019	CDK4	protein_coding
0,2774991	PPP1R1A	protein_coding
0,9112805	TSPAN31	protein_coding
0,7956173	TFCP2	protein_coding
0,1779442	COQ10A	protein_coding
0,949756	PAN2	protein_coding
0,3480863	HNRNPA1	protein_coding
0,7956408	ACVR1B	protein_coding
0,7253153	OS9	protein_coding
0,5117223	LTV1	protein_coding
0,9925374	CD164	protein_coding
0,7352523	AFG1L	protein_coding
0,290302	NHSL1	protein_coding
0,8711676	AHI1	protein_coding
0,5879554	MICAL1	protein_coding
0,6621874	REPS1	protein_coding
0,3264373	PRADC1	protein_coding
0,3830886	CCT7	protein_coding
0,9998538	RAB11FIP5	protein_coding
0,9998478	SMYD5	protein_coding
0,7244173	DYSF	protein_coding
0,7213265	USP15	protein_coding
0,5801848	GNS	protein_coding
0,692146	CPM	protein_coding
0,9142106	MDM2	protein_coding
0,7318327	KLHL36	protein_coding
0,7010516	KIAA0513	protein_coding
0,2332141	DYNC1LI2	protein_coding
0,993752	AGT	protein_coding
0,3587016	KCNK1	protein_coding
0,6416356	COG2	protein_coding
0,654521	ABCB10	protein_coding
0,4103148	NTPCR	protein_coding
0,997931	TAF5L	protein_coding
0,1674733	GLUL	protein_coding
0,2945575	STX6	protein_coding
0,8619772	RNASEL	protein_coding
0,922549	DHX9	protein_coding
0,9136442	CEP350	protein_coding
0,8992483	NIBAN1	protein_coding
0,9652509	PIGC	protein_coding
0,9700303	LAMC1	protein_coding
0,8622295	RC3H1	protein_coding
0,9397602	SP110	protein_coding
0,6244656	MRPL44	protein_coding

0,4141191	DOCK10	protein_coding
0,953175	USP37	protein_coding
0,9352317	ITM2C	protein_coding
0,4638153	SERPINE2	protein_coding
0,6673583	DNAJB2	protein_coding
0,9477062	TMBIM1	protein_coding
0,0829375	CYP27A1	protein_coding
0,8882787	EIF4E2	protein_coding
0,6232447	CAB39	protein_coding
0,3803906	COX5B	protein_coding
0,9789395	REV1	protein_coding
0,922549	TMEM127	protein_coding
0,5164856	TGFBRAP1	protein_coding
0,7118106	GCC2	protein_coding
0,2012728	MRPS9	protein_coding
0,7426221	C2orf49	protein_coding
0,7639764	ANKRD36	protein_coding
0,6371005	EPC2	protein_coding
0,7892243	ISCU	protein_coding
0,9933219	ALDH1L2	protein_coding
0,764019	SCYL2	protein_coding
0,5844756	CKAP4	protein_coding
0,9039795	PLXNC1	protein_coding
0,9182782	APPL2	protein_coding
0,9781148	PWP1	protein_coding
0,5474737	DRAM1	protein_coding
0,9731251	WASHC4	protein_coding
0,2354584	SLC41A2	protein_coding
0,9490174	FLNB	protein_coding
0,7385573	NEK3	protein_coding
0,5511615	VPS36	protein_coding
0,4724079	RNASEH2B	protein_coding
0,9986615	CKAP2	protein_coding
0,9738194	TBC1D4	protein_coding
0,8930787	THSD1	protein_coding
0,902171	LRCH1	protein_coding
0,4045325	SUCLA2	protein_coding
0,998169	RCBTB1	protein_coding
0,7211115	MED4	protein_coding
0,9731251	PHF11	protein_coding
0,654521	RPL13AP25	processed_pseudogene
0,7235397	COG3	protein_coding
0,8360601	LMO7	protein_coding
0,7842247	ITM2B	protein_coding
0,466638	SPRY2	protein_coding
0,4872295	NUDT15	protein_coding
0,3406074	EDNRB	protein_coding
0,6653209	RCBTB2	protein_coding
0,7972313	LCP1	protein_coding
0,8213028	SETDB2	protein_coding
0,2529607	SCRN1	protein_coding
0,4835164	TNS3	protein_coding
0,8919058	GPNMB	protein_coding
0,7611529	RAPGEF5	protein_coding
0,9406097	RAC1	protein_coding
0,8869707	KDELR2	protein_coding
0,7639764	NUP42	protein_coding
0,6760314	ZDHHC4	protein_coding
0,4535149	BZW2	protein_coding

0,7805908	TBRG4	protein_coding
0,9296784	DDX56	protein_coding
0,9997039	HUS1	protein_coding
0,983457	DBNL	protein_coding
0,8930787	CCM2	protein_coding
0,4737234	TTC5	protein_coding
0,5964345	ADAMTS7	protein_coding
0,8861906	IREB2	protein_coding
0,4835164	ALPK3	protein_coding
0,0396848	CALCOCO2	protein_coding
0,6160654	RSAD1	protein_coding
0,9828457	NMT1	protein_coding
0,9792506	SRSF1	protein_coding
0,9074092	VEZF1	protein_coding
0,3178501	TACO1	protein_coding
0,7196031	TEX2	protein_coding
0,9709611	DCAF7	protein_coding
0,9731416	KAT7	protein_coding
0,8500846	ACTL6A	protein_coding
0,5765517	NDUFB5	protein_coding
0,3177457	MRPL47	protein_coding
0,9884562	TRA2B	protein_coding
0,7921707	MARCH7	protein_coding
0,579213	SCN7A	protein_coding
0,8609959	TANK	protein_coding
0,9738194	GATA4	protein_coding
0,3358222	SKIL	protein_coding
0,7713242	EPRS	protein_coding
0,9538485	VPS45	protein_coding
0,9312186	KCTD3	protein_coding
0,9956922	RPS6KC1	protein_coding
0,3776155	CBWD2	protein_coding
0,931978	SMPD4	protein_coding
0,7444016	WDR33	protein_coding
0,4260926	CCDC115	protein_coding
0,933129	SAP130	protein_coding
0,8118149	IMP4	protein_coding
0,9802452	UGGT1	protein_coding
0,3435112	GYPC	protein_coding
0,2948143	STAM	protein_coding
0,3919684	ABI1	protein_coding
0,9424246	YME1L1	protein_coding
0,9792506	DNAJC1	protein_coding
0,796666	NIPSNAP3A	protein_coding
0,8578793	LRRC8A	protein_coding
0,7309037	CDK9	protein_coding
0,7576959	TXN	protein_coding
0,3524133	ODF2	protein_coding
0,3651489	ECPAS	protein_coding
0,9792506	TOR1B	protein_coding
0,7558125	C9orf78	protein_coding
0,6254273	SMC2	protein_coding
0,3943278	KLF4	protein_coding
0,9102206	TOR1A	protein_coding
0,9700303	RALGPS1	protein_coding
0,997931	NIBAN2	protein_coding
0,9854182	TMOD1	protein_coding
0,9497298	DAB2IP	protein_coding
0,9463928	STXBP1	protein_coding

0,2712333	ANGPTL2	protein_coding
0,7467854	CDK5RAP2	protein_coding
0,6184746	SLC31A1	protein_coding
0,8736463	TLR4	protein_coding
0,3792977	ZNF189	protein_coding
0,8881504	STX17	protein_coding
0,8903094	PRPF4	protein_coding
0,4835164	FPGS	protein_coding
0,6604792	ATP6V1G1	protein_coding
0,8131645	TEX10	protein_coding
0,6898216	GARNL3	protein_coding
0,4806087	MRPL50	protein_coding
0,9067454	TSTD2	protein_coding
0,1500522	PSMB7	protein_coding
0,9465232	GOLGA1	protein_coding
0,9925374	XPA	protein_coding
0,9583708	NCBP1	protein_coding
0,7639764	ANP32B	protein_coding
0,8868311	PDCL	protein_coding
0,8545769	RPL35	protein_coding
0,8116293	ARPC5L	protein_coding
0,9955768	ENPP2	protein_coding
0,8189129	DERL1	protein_coding
0,9305033	MYC	protein_coding
0,9233889	IL33	protein_coding
0,8890564	DMAC1	protein_coding
0,4344504	RANBP6	protein_coding
0,4835164	POLR1E	protein_coding
0,6676251	PLAA	protein_coding
0,9427242	IL11RA	protein_coding
0,90504	UBAP2	protein_coding
0,4160411	APTX	protein_coding
0,2945575	RNF38	protein_coding
0,7049962	TLN1	protein_coding
0,6662301	DNAJB5	protein_coding
0,9233889	DCTN3	protein_coding
0,3953915	TMEM8B	protein_coding
0,8721867	GRHPR	protein_coding
0,8023631	ALDH1B1	protein_coding
0,9934576	DENND4C	protein_coding
0,633922	RPS6	protein_coding
0,0225992	PPIL1	protein_coding
0,7114715	KLC4	protein_coding
0,1067405	KIF13A	protein_coding
0,4740961	ZSCAN9	protein_coding
0,3000115	PIM1	protein_coding
0,3803906	GMPR	protein_coding
0,9194718	CMTR1	protein_coding
0,824287	YIPF3	protein_coding
0,6322951	TMEM14B	protein_coding
0,4466289	TMEM63B	protein_coding
0,4929726	TUBB2A	protein_coding
0,8930787	RIPK1	protein_coding
0,517991	UQCC2	protein_coding
0,445647	FLOT1	protein_coding
0,8670918	MDC1	protein_coding
0,5578794	TPMT	protein_coding
0,1067405	RNF144B	protein_coding
0,6584077	MTCH1	protein_coding

0,6023346	TAF8	protein_coding
0,6673583	FAM8A1	protein_coding
0,9359783	CPEB2	protein_coding
0,8454927	TLR2	protein_coding
0,696332	FCHSD2	protein_coding
0,7558125	ARRB1	protein_coding
0,654521	SLCO2B1	protein_coding
0,4806087	THAP12	protein_coding
0,4408807	NUMA1	protein_coding
0,9995727	CCDC90B	protein_coding
0,4954628	SYTL2	protein_coding
0,8185595	RAB30	protein_coding
0,8117718	CREBZF	protein_coding
0,8597029	LRRC32	protein_coding
0,8703454	PRCP	protein_coding
0,9771336	NARS2	protein_coding
0,7965978	RNF121	protein_coding
0,0845999	MRPL15	protein_coding
0,4835164	SLCO5A1	protein_coding
0,1916186	SULF1	protein_coding
0,9789623	TGS1	protein_coding
0,2748973	SDCBP	protein_coding
0,8221205	NEK1	protein_coding
0,3447404	DDX60	protein_coding
0,8114908	SORL1	protein_coding
0,5747726	DCUN1D5	protein_coding
0,3187319	YAP1	protein_coding
0,855954	RDX	protein_coding
0,7639764	PPP2R1B	protein_coding
0,2440137	FDX1	protein_coding
0,6160654	CASP1	protein_coding
0,9463928	ALKBH8	protein_coding
0,939763	MAP2K5	protein_coding
0,784306	CTDSPL2	protein_coding
0,922549	SLTM	protein_coding
0,2174016	THBS1	protein_coding
0,7956789	MAPKBP1	protein_coding
0,2337466	NDUFAF1	protein_coding
0,4806087	ITGA11	protein_coding
0,9998538	HAUS2	protein_coding
0,8038845	RTF1	protein_coding
0,7122541	PARP6	protein_coding
0,8934536	RPLP1	protein_coding
0,6229784	LRRC49	protein_coding
0,7764724	TUBGCP4	protein_coding
0,8908494	RMDN3	protein_coding
0,6516554	UACA	protein_coding
0,1131434	SMAD6	protein_coding
0,5020851	TMEM62	protein_coding
0,8930787	ADAM10	protein_coding
0,8992483	ZNF280D	protein_coding
0,6140495	SEMA6D	protein_coding
0,8682129	RSL24D1	protein_coding
0,1712026	GCOM1	protein_coding
0,9700303	BCAR3	protein_coding
0,4721853	TTLL7	protein_coding
0,9997554	FNBP1L	protein_coding
0,8554227	KYAT3	protein_coding
0,9625751	GTF2B	protein_coding

0,6611168	RABGGTB	protein_coding
0,9803555	IFI44L	protein_coding
0,4039226	ARHGAP29	protein_coding
0,9484395	IFI44	protein_coding
0,997931	RPL7P9	processed_pseudogene
0,7932064	DBT	protein_coding
0,6025953	RTCA	protein_coding
0,7291201	IFT172	protein_coding
0,5308826	SELENOI	protein_coding
0,3496454	HADHB	protein_coding
0,2971924	ADCY3	protein_coding
0,3803906	PPM1B	protein_coding
0,2841212	PNPT1	protein_coding
0,4894579	DYNC2LI1	protein_coding
0,6059615	THUMPD2	protein_coding
0,8301309	CYP1B1	protein_coding
0,645515	RAB1A	protein_coding
0,9738194	ACTR2	protein_coding
0,9998478	PREB	protein_coding
0,9781971	PREPL	protein_coding
0,8295224	FBXO11	protein_coding
0,7688687	ATRAID	protein_coding
0,8578793	LRPPRC	protein_coding
0,7178359	TRIM54	protein_coding
0,5021482	ACTR1A	protein_coding
0,578003	MYOF	protein_coding
0,5187247	STAMBPL1	protein_coding
0,4649064	ATAD1	protein_coding
0,8161522	TACC2	protein_coding
0,654521	DUSP5	protein_coding
0,0727176	ARL3	protein_coding
0,9205351	KIF20B	protein_coding
0,5537742	ENTPD1	protein_coding
0,7137584	EXOC6	protein_coding
0,2533191	PLCE1	protein_coding
0,817424	DNAJC13	protein_coding
0,9296784	ANXA7	protein_coding
0,9142106	FAM149B1	protein_coding
0,2560075	ASCC1	protein_coding
0,933129	RPS24	protein_coding
0,8921822	TET1	protein_coding
0,5677206	MYPN	protein_coding
0,7972313	AOX1	protein_coding
0,6128477	ATIC	protein_coding
0,8238032	SMARCAL1	protein_coding
0,8492407	BARD1	protein_coding
0,5953122	STAT4	protein_coding
0,9967216	CARF	protein_coding
0,3894807	ASNSD1	protein_coding
0,4684923	METTL5	protein_coding
0,8554227	SSB	protein_coding
0,620901	NAB1	protein_coding
0,887319	PPIG	protein_coding
0,4638153	FASTKD1	protein_coding
0,9082846	HECW2	protein_coding
0,9465232	IDH1	protein_coding
0,4521055	OLA1	protein_coding
0,9839612	CIR1	protein_coding
0,8877092	ITPRID2	protein_coding

0,0727176	WDR12	protein_coding
0,895571	ABI2	protein_coding
0,696332	ITGAV	protein_coding
0,5890453	SLC40A1	protein_coding
0,9808899	SLC35A5	protein_coding
0,9533676	SLC49A4	protein_coding
0,7763012	SENP7	protein_coding
0,0465925	COX17	protein_coding
0,7419278	PARP9	protein_coding
0,6158337	USP8	protein_coding
0,6925926	SECISBP2L	protein_coding
0,5647201	TMOD3	protein_coding
0,7576959	SPPL2A	protein_coding
0,8695843	GLCE	protein_coding
0,7444016	APH1B	protein_coding
0,9112805	INTS14	protein_coding
0,1444307	CILP	protein_coding
0,7558125	UBL7	protein_coding
0,9021789	ARHGAP24	protein_coding
0,7122873	FAM13A	protein_coding
0,8653179	HERC6	protein_coding
0,4521055	ZGRF1	protein_coding
0,7154026	AP1AR	protein_coding
0,4835164	COPS4	protein_coding
0,993752	HNRNPD	protein_coding
0,7687197	PRKG2	protein_coding
0,5265966	RASGEF1B	protein_coding
0,0349682	SEC31A	protein_coding
0,9185201	GPAT3	protein_coding
0,5943942	FGF2	protein_coding
0,6367477	BBS7	protein_coding
0,9854182	KIAA1109	protein_coding
0,8575378	RAP1GDS1	protein_coding
0,9366634	LARP1B	protein_coding
0,7196574	PDE5A	protein_coding
0,290302	PRDM5	protein_coding
0,5563416	NAAA	protein_coding
0,9954421	NUP54	protein_coding
0,8117718	BMP2K	protein_coding
0,933129	G3BP2	protein_coding
0,4151093	SEPTIN11	protein_coding
0,1154353	FRAS1	protein_coding
0,9615223	SCARB2	protein_coding
0,5164856	CCNG2	protein_coding
0,9182782	CNOT6L	protein_coding
0,8875885	USO1	protein_coding
0,5183663	SHROOM3	protein_coding
0,3480863	ANXA3	protein_coding
0,4497301	PPA2	protein_coding
0,4933085	INTS12	protein_coding
0,0897312	ENPEP	protein_coding
0,6811841	HADH	protein_coding
0,1970133	EGF	protein_coding
0,4456276	PAPSS1	protein_coding
0,8788135	SEC24B	protein_coding
0,8901085	PPP3CA	protein_coding
0,1895063	SLC39A8	protein_coding
0,0895372	FBN2	protein_coding
0,6613295	MAPK8IP3	protein_coding

0,9423015	RGS3	protein_coding
0,633922	GUCD1	protein_coding
0,9745853	RNF185	protein_coding
0,3013021	B4GALNT3	protein_coding
0,4954628	ETV6	protein_coding
0,8148845	GABARAPL1	protein_coding
0,4875875	KIF21A	protein_coding
0,9296784	CPNE8	protein_coding
0,654521	FGD4	protein_coding
0,9154352	SINHCAF	protein_coding
0,8640757	AEBP2	protein_coding
0,9997039	ETNK1	protein_coding
0,5677206	ZCRB1	protein_coding
0,7987424	TMEM117	protein_coding
0,0464426	PRICKLE1	protein_coding
0,9781971	C1RL	protein_coding
0,4179007	NDUFA9	protein_coding
0,8703454	PEX5	protein_coding
0,9229915	SCAF11	protein_coding
0,9789395	LLPH	protein_coding
0,8912998	RPL14P1	processed_pseudogene
0,2778507	LRIG3	protein_coding
0,9894339	GLIPR1	protein_coding
0,1721544	PHLDA1	protein_coding
0,4806087	TMEM19	protein_coding
0,472118	PTPRQ	protein_coding
0,0757434	DUSP6	protein_coding
0,8698323	POC1B	protein_coding
0,9625751	TMTC3	protein_coding
0,1326966	LUM	protein_coding
0,813314	NEDD1	protein_coding
0,9951314	SLC15A4	protein_coding
0,6232447	TDG	protein_coding
0,6316303	MMAB	protein_coding
0,9168915	GLTP	protein_coding
0,9524609	GIT2	protein_coding
0,9210173	TCHP	protein_coding
0,7713242	NUP58	protein_coding
0,6757338	MTMR6	protein_coding
0,9112805	SLC46A3	protein_coding
0,6909761	SLC7A1	protein_coding
0,2690459	LNX2	protein_coding
0,8500846	SUOX	protein_coding
0,796666	ACVRL1	protein_coding
0,5245497	SMARCC2	protein_coding
0,3830886	KANSL2	protein_coding
0,9237476	CERS5	protein_coding
0,8185595	CSAD	protein_coding
0,9933219	LMBR1L	protein_coding
0,4957019	ESYT1	protein_coding
0,8099847	TMBIM6	protein_coding
0,7984578	ANKRD52	protein_coding
0,4748134	ZNF740	protein_coding
0,6835435	WDFY2	protein_coding
0,4826313	HNRNPA1L2	protein_coding
0,7291201	LPAR6	protein_coding
0,5180247	ESD	protein_coding
0,8386162	RB1	protein_coding
0,9182782	SBNO1	protein_coding

0,9914435	DENR	protein_coding
0,8619772	RBM26	protein_coding
0,692146	MBNL2	protein_coding
0,9450135	ABHD13	protein_coding
0,8877092	CUL4A	protein_coding
0,4721853	FITM1	protein_coding
0,993752	TMX1	protein_coding
0,7118106	FRMD6	protein_coding
0,8966801	PELI2	protein_coding
0,8930787	ARMH4	protein_coding
0,998169	SLC38A6	protein_coding
0,7929944	NAA30	protein_coding
0,9998478	DCAF5	protein_coding
0,349702	RAB15	protein_coding
0,900863	STON2	protein_coding
0,9233889	JDP2	protein_coding
0,7892243	FBLN5	protein_coding
0,2506935	WARS	protein_coding
0,9524483	WDR20	protein_coding
0,5245497	NIPA2	protein_coding
0,9410184	SLC12A6	protein_coding
0,9789623	MFAP1	protein_coding
0,561634	TCF12	protein_coding
0,9854069	SERF2	protein_coding
0,8722977	ZSCAN29	protein_coding
0,284683	FGF7	protein_coding
0,998169	BNIP2	protein_coding
0,5869872	GTF2A2	protein_coding
0,5062136	SRP14	protein_coding
0,8219774	TLE3	protein_coding
0,9808899	ANP32A	protein_coding
0,2965462	UBE2Q2	protein_coding
0,7627705	ETFA	protein_coding
0,8721867	HMG20A	protein_coding
0,3880092	SCAPER	protein_coding
0,6285359	TSPAN3	protein_coding
0,9925374	WDR61	protein_coding
0,903318	NCOA2	protein_coding
0,8017097	MAN2C1	protein_coding
0,8850531	DNAJA4	protein_coding
0,4413743	TLNRD1	protein_coding
0,7057791	TPM1	protein_coding
0,887128	IGF1R	protein_coding
0,3799704	ARRDC4	protein_coding
0,8287149	USP3	protein_coding
0,4089968	BBS4	protein_coding
0,7639764	PML	protein_coding
0,7921707	LINS1	protein_coding
0,6098976	ULK3	protein_coding
0,4894579	PCSK6	protein_coding
0,8992483	SCAMP2	protein_coding
0,8882787	POLG	protein_coding
0,9954421	ABHD2	protein_coding
0,9922658	MFGE8	protein_coding
0,6800165	UNC45A	protein_coding
0,8992483	FURIN	protein_coding
0,8672415	IQGAP1	protein_coding
0,9700303	CRTC3	protein_coding
0,9157791	EFL1	protein_coding

0,9108751	SEC11A	protein_coding
0,8918638	GLYR1	protein_coding
0,8287149	PMM2	protein_coding
0,7291201	PARN	protein_coding
0,5879554	FTO	protein_coding
0,4750209	UQCRC2	protein_coding
0,1232731	CDR2	protein_coding
0,9464798	ARHGAP17	protein_coding
0,8147279	MYLK3	protein_coding
0,6638064	DHX38	protein_coding
0,5537742	ZFHX3	protein_coding
0,5134965	CPNE2	protein_coding
0,9666028	NLRC5	protein_coding
0,5580971	KIFC3	protein_coding
0,8100875	GCSH	protein_coding
0,1284788	CDH11	protein_coding
0,860343	NOL3	protein_coding
0,8882787	MAP1LC3B	protein_coding
0,6951729	MBTPS1	protein_coding
0,6615319	CDH13	protein_coding
0,4599964	ZCCHC14	protein_coding
0,3765746	RPL3L	protein_coding
0,8919058	RPS2	protein_coding
0,146655	NDUFB10	protein_coding
0,9366634	PDPK1	protein_coding
0,9618453	DEF8	protein_coding
0,615864	TCF25	protein_coding
0,3607643	MED9	protein_coding
0,9481776	NCOR1	protein_coding
0,2190274	COPS3	protein_coding
0,9625751	GID4	protein_coding
0,8545769	ZNF287	protein_coding
0,9142106	MYOCD	protein_coding
0,8457624	UTP4	protein_coding
0,3435112	RANBP10	protein_coding
0,9165105	PRPSAP2	protein_coding
0,9638699	UNC45B	protein_coding
0,8500846	PCTP	protein_coding
0,8992483	TOM1L1	protein_coding
0,8882787	C17orf80	protein_coding
0,3244086	TOB1	protein_coding
0,9484395	VPS53	protein_coding
0,6613817	SGSM2	protein_coding
0,9476644	NPEPPS	protein_coding
0,6390934	SCRN2	protein_coding
0,4750209	SSH2	protein_coding
0,5365997	ABCA8	protein_coding
0,9450135	CLTC	protein_coding
0,621558	BCAS3	protein_coding
0,1799031	PTRH2	protein_coding
0,9998538	SS18	protein_coding
0,9925374	AFG3L2	protein_coding
0,9894339	SLC39A6	protein_coding
0,4835164	RPRD1A	protein_coding
0,9533676	GALNT1	protein_coding
0,8309028	ASXL3	protein_coding
0,7242009	GAREM1	protein_coding
0,7118106	ESCO1	protein_coding
0,2098554	OSBPL1A	protein_coding

0,3814605	GATA6	protein_coding
0,5456715	GREB1L	protein_coding
0,9544158	RMC1	protein_coding
0,8992483	NPC1	protein_coding
0,9792506	SLC14A1	protein_coding
0,7125689	ARRB2	protein_coding
0,8130747	MINK1	protein_coding
0,5042626	SAT2	protein_coding
0,6433147	TTYH2	protein_coding
0,9997554	EIF4A3	protein_coding
0,9251589	CSNK1D	protein_coding
0,7118106	ANAPC11	protein_coding
0,8885864	TBCD	protein_coding
0,4954628	NARF	protein_coding
0,7321942	RPTOR	protein_coding
0,6574161	FOXK2	protein_coding
0,921365	WDR45B	protein_coding
0,9367129	DYM	protein_coding
0,7972313	MAPK4	protein_coding
0,6305527	MBD1	protein_coding
0,7214394	SMAD4	protein_coding
0,7291201	ZCCHC2	protein_coding
0,401929	RETREG3	protein_coding
0,8647372	ERBB2	protein_coding
0,6128477	IGFBP4	protein_coding
0,3466656	FKBP10	protein_coding
0,4732706	TXNL4A	protein_coding
0,9356716	BRD4	protein_coding
0,9840915	NFIC	protein_coding
0,633922	PFKL	protein_coding
0,9081173	DPP9	protein_coding
0,5985056	ZFP14	protein_coding
0,5420087	SIRT3	protein_coding
0,7972313	IFITM3	protein_coding
0,7673893	COL6A1	protein_coding
0,7104968	IFNAR1	protein_coding
0,3030424	SOD1	protein_coding
0,9178514	COL6A2	protein_coding
0,0941174	SIK1	protein_coding
0,8670918	SCYL1	protein_coding
0,3660091	TMEM50B	protein_coding
0,7929944	APP	protein_coding
0,9169269	DOP1B	protein_coding
0,6613295	URB1	protein_coding
0,8575378	AKT1	protein_coding
0,4845515	EMP3	protein_coding
0,8670918	SAE1	protein_coding
0,9709611	RNPEPL1	protein_coding
0,32001	PSMB6	protein_coding
0,7910551	RPS11	protein_coding
0,6662006	RPL13A	protein_coding
0,9900815	ZNF614	protein_coding
0,887319	RERE	protein_coding
0,6985177	PGD	protein_coding
0,9804708	MYOM3	protein_coding
0,9248086	RPL11	protein_coding
0,9636851	C1orf216	protein_coding
0,9670362	KIAA0319L	protein_coding
0,4799252	MAP3K6	protein_coding

0,8100875	GPN2	protein_coding
0,8255851	WDTC1	protein_coding
0,4835164	HSPG2	protein_coding
0,3930297	ITGB3BP	protein_coding
0,9619323	SERBP1	protein_coding
0,8992483	BCL10	protein_coding
0,7558125	CCN1	protein_coding
0,9512671	PRKACB	protein_coding
0,4583602	PIGK	protein_coding
0,3697716	TINAGL1	protein_coding
0,721162	RPS8	protein_coding
0,4848227	PTPRF	protein_coding
0,9977992	MOB3C	protein_coding
0,7252511	LMO4	protein_coding
0,9998538	MTF2	protein_coding
0,6591549	IGSF3	protein_coding
0,3212727	ZNF697	protein_coding
0,9802452	CTTNBP2NL	protein_coding
0,8500846	STRIP1	protein_coding
0,5911872	PSMA5	protein_coding
0,7166966	CD53	protein_coding
0,887128	CELSR2	protein_coding
0,855954	GPR161	protein_coding
0,4894579	ALDH9A1	protein_coding
0,2337466	ATP1B1	protein_coding
0,8303367	TIPRL	protein_coding
0,9168619	POGK	protein_coding
0,2318378	MPC2	protein_coding
0,7942035	CREG1	protein_coding
0,5622598	DCAF6	protein_coding
0,7687197	TMCO1	protein_coding
0,9171648	POU2F1	protein_coding
0,5267449	ILDR2	protein_coding
0,0959391	DPT	protein_coding
0,8166471	MGST3	protein_coding
0,9428271	COP1	protein_coding
0,8969303	UFC1	protein_coding
0,4806087	FCGR2A	protein_coding
0,4954628	RGS5	protein_coding
0,5622214	SDHC	protein_coding
0,7740102	PRCC	protein_coding
0,4835164	MRPL24	protein_coding
0,9316489	PIGM	protein_coding
0,4793848	CASQ1	protein_coding
0,5397488	HDGF	protein_coding
0,9792506	ABL2	protein_coding
0,4835164	XPR1	protein_coding
0,2908312	TOR1AIP1	protein_coding
0,4911389	HMCN1	protein_coding
0,9652509	RGL1	protein_coding
0,9906886	LYPLAL1	protein_coding
0,8594992	PRUNE1	protein_coding
0,9127443	ECM1	protein_coding
0,9406097	TARS2	protein_coding
0,8672415	SNX27	protein_coding
0,9496064	SETDB1	protein_coding
0,2104415	ADAMTSL4	protein_coding
0,4688629	MCL1	protein_coding
0,492696	CTSK	protein_coding

0,8976077	RFX5	protein_coding
0,7213265	PI4KB	protein_coding
0,8500846	PIP5K1A	protein_coding
0,8930787	ANP32E	protein_coding
0,6970327	MINDY1	protein_coding
0,9477707	SELENBP1	protein_coding
0,6624016	CERS2	protein_coding
0,9907221	ENSA	protein_coding
0,9312186	LSP1P4	transcribed_unprocessed_pseudogene
0,9358987	SEMA6C	protein_coding
0,7740102	MRPL9	protein_coding
0,6903585	ARNT	protein_coding
0,5341139	POGZ	protein_coding
0,6476315	GOLPH3L	protein_coding
0,3953915	GABPB2	protein_coding
0,9583287	DYRK3	protein_coding
0,4593782	EIF2D	protein_coding
0,4954483	INTS7	protein_coding
0,0088417	SMYD2	protein_coding
0,9481776	TP53BP2	protein_coding
0,4765978	ATP8B2	protein_coding
0,5501237	ADAM15	protein_coding
0,9840915	JTB	protein_coding
0,645515	S100A8	protein_coding
0,5557127	TPM3	protein_coding
0,613355	SNAPIN	protein_coding
0,8922683	UBAP2L	protein_coding
0,3113413	HAX1	protein_coding
0,1767096	C1orf43	protein_coding
0,8076343	GATAD2B	protein_coding
0,9998478	ILF2	protein_coding
0,8219707	RIT1	protein_coding
0,5140003	INTS3	protein_coding
0,9682998	ACTA1	protein_coding
0,5360859	GALNT2	protein_coding
0,9378831	TTC13	protein_coding
0,8545769	SCCPDH	protein_coding
0,7681066	LYST	protein_coding
0,8019639	CEP170	protein_coding
0,7763012	ACP1	protein_coding
0,5164856	SNAP47	protein_coding
0,5625279	SRP9	protein_coding
0,9893136	NVL	protein_coding
0,9967216	SDE2	protein_coding
0,8160828	DEGS1	protein_coding
0,9700303	FBXO28	protein_coding
0,8500846	ARF1	protein_coding
0,8512635	CNIH4	protein_coding
0,7435926	ITPKB	protein_coding
0,8118149	GUK1	protein_coding
0,5558351	CDC42BPA	protein_coding
0,9411338	MBOAT2	protein_coding
0,9917279	PARP1	protein_coding
0,806905	PSEN2	protein_coding
0,9954421	PYCR2	protein_coding
0,8500846	LBR	protein_coding
0,9345529	EPHX1	protein_coding
0,6676442	PPFIA4	protein_coding
0,0731855	PLEKHA6	protein_coding

0,9994264	ARL8A	protein_coding
0,0286685	OSR1	protein_coding
0,9142507	PDIA6	protein_coding
0,6676251	RHOB	protein_coding
0,806905	HNRNPLL	protein_coding
0,8381234	GALM	protein_coding
0,7444016	EML4	protein_coding
0,6475905	CALM2	protein_coding
0,7628544	RPS27A	protein_coding
0,6473313	WDPCP	protein_coding
0,9479329	VPS54	protein_coding
0,7436469	ASXL2	protein_coding
0,9356153	ETAA1	protein_coding
0,4195381	SNRPG	protein_coding
0,0796818	MEIS1	protein_coding
0,9781971	CIAO1	protein_coding
0,4835164	ZNF514	protein_coding
0,7010516	SNRNP200	protein_coding
0,1149889	MRPS5	protein_coding
0,2164941	TPRKB	protein_coding
0,8117718	EXOC6B	protein_coding
0,8992483	TEX261	protein_coding
0,9074092	DUSP11	protein_coding
0,9572126	NPHP1	protein_coding
0,4409169	MALL	protein_coding
0,7081667	THNSL2	protein_coding
0,7842247	RALB	protein_coding
0,3883821	SLC20A1	protein_coding
0,9536474	ZC3H8	protein_coding
0,8229361	FAHD2B	protein_coding
0,9925374	AFF3	protein_coding
0,9967216	UBXN4	protein_coding
0,9794449	SPOPL	protein_coding
0,8578793	POLR2D	protein_coding
0,7440219	AMMECR1L	protein_coding
0,8885864	PKP4	protein_coding
0,7072223	SCRN3	protein_coding
0,6044343	LNPK	protein_coding
0,0941174	ZNF385B	protein_coding
0,6776989	UBR3	protein_coding
0,6947291	GULP1	protein_coding
0,6766513	FAM171B	protein_coding
0,784306	HSPD1	protein_coding
0,8992483	NBEAL1	protein_coding
0,9768456	KANSL1L	protein_coding
0,9975676	SPAG16	protein_coding
0,6076071	SUMF1	protein_coding
0,8189129	RHBDD1	protein_coding
0,2231549	ACKR3	protein_coding
0,9933219	COPS7B	protein_coding
0,9734904	DIS3L2	protein_coding
0,2551699	VGLL4	protein_coding
0,993752	RAB5A	protein_coding
0,3680294	RETREG2	protein_coding
0,7154026	CTDSP1	protein_coding
0,7968733	CNOT9	protein_coding
0,4835164	GRIP2	protein_coding
0,6254273	EAF1	protein_coding
0,6137496	DYNC1LI1	protein_coding

0,3049645	RBMS3	protein_coding
0,1622398	CSRNP1	protein_coding
0,9954421	SLC25A38	protein_coding
0,8833312	ITGA9	protein_coding
0,887128	GOLGA4	protein_coding
0,5209123	CTDSPL	protein_coding
0,9865636	IQSEC1	protein_coding
0,9484395	CAND2	protein_coding
0,9127443	RPL32	protein_coding
0,2046145	PTPRG	protein_coding
0,9057527	IL17RD	protein_coding
0,5622112	SHQ1	protein_coding
0,0286685	SLC25A26	protein_coding
0,445647	UBA3	protein_coding
0,5265966	ARL6IP5	protein_coding
0,624851	TMF1	protein_coding
0,8545115	LRIG1	protein_coding
0,7558125	LIMD1	protein_coding
0,997931	ZNF660	protein_coding
0,6527079	NFKBIZ	protein_coding
0,0286685	COL8A1	protein_coding
0,7149564	NXPE3	protein_coding
0,3126419	PHLDB2	protein_coding
0,841477	ABHD10	protein_coding
0,943695	RABL3	protein_coding
0,7558125	ATG3	protein_coding
0,981857	BOC	protein_coding
0,9424246	SRPRB	protein_coding
0,7013708	EIF2A	protein_coding
0,9540553	ALDH1L1	protein_coding
0,4593782	OSBPL11	protein_coding
0,8118149	TRPC1	protein_coding
0,472118	NCEH1	protein_coding
0,935463	LPP	protein_coding
0,4919121	TCTA	protein_coding
0,9997039	DCAF1	protein_coding
0,6906735	MANF	protein_coding
0,3881918	SLIT2	protein_coding
0,7698372	EIF2B5	protein_coding
0,8721867	FIP1L1	protein_coding
0,5773893	LYAR	protein_coding
0,7816527	CENPC	protein_coding
0,922549	CORIN	protein_coding
0,3087684	ATP10D	protein_coding
0,9484259	ENOPH1	protein_coding
0,962885	KLHL8	protein_coding
0,8553175	PYURF	protein_coding
0,6383874	TBCK	protein_coding
0,9670595	CAMK2D	protein_coding
0,6254273	CISD2	protein_coding
0,8801593	ANK2	protein_coding
0,9081173	TIFA	protein_coding
0,9792506	SPATA5	protein_coding
0,9998478	METTL14	protein_coding
0,8158016	USP53	protein_coding
0,2190274	SETD7	protein_coding
0,6116816	NAF1	protein_coding
0,7905225	MARCH1	protein_coding
0,7230802	RPS3A	protein_coding

0,2308926	PDGFC	protein_coding
0,4740961	CBR4	protein_coding
0,8492407	CYP4V2	protein_coding
0,3126419	NDUFS6	protein_coding
0,7968733	MARCH6	protein_coding
0,6753466	MYO10	protein_coding
0,7740102	RPL37	protein_coding
0,284683	OSMR	protein_coding
0,5641983	PLK2	protein_coding
0,9700639	PIK3R1	protein_coding
0,998169	LHFPL2	protein_coding
0,5455384	SSBP2	protein_coding
0,5511615	IQGAP2	protein_coding
0,9552698	RASA1	protein_coding
0,5567171	GIN1	protein_coding
0,6662301	PPIP5K2	protein_coding
0,8682799	PAM	protein_coding
0,9112805	BDP1	protein_coding
0,9368504	GTF2H2	protein_coding
0,4750209	SLC30A5	protein_coding
0,9408651	BTF3	protein_coding
0,6818653	FBXL17	protein_coding
0,601474	FEM1C	protein_coding
0,5265966	COMMD10	protein_coding
0,8698319	ATG12	protein_coding
0,8847715	YIPF5	protein_coding
0,4165098	ARHGAP26	protein_coding
0,7357059	DDX46	protein_coding
0,9543278	RNF145	protein_coding
0,6613295	FBXO38	protein_coding
0,4593782	TNIP1	protein_coding
0,7738482	G3BP1	protein_coding
0,7733348	RMND5B	protein_coding
0,993752	BOD1	protein_coding
0,4519396	TENM2	protein_coding
0,0941174	TBC1D7	protein_coding
0,1974243	FARS2	protein_coding
0,1712026	GFOD1	protein_coding
0,6053653	CDKAL1	protein_coding
0,817424	LRRTM2	protein_coding
0,8163405	ZMAT2	protein_coding
0,3648373	KLHL3	protein_coding
0,5641983	HIGD2A	protein_coding
0,9810626	FAM193B	protein_coding
0,6873469	TNFRSF21	protein_coding
0,5544626	MMUT	protein_coding
0,8463447	PPP1R18	protein_coding
0,8117718	DAAM2	protein_coding
0,5511615	MLIP	protein_coding
0,6543081	RPL7L1	protein_coding
0,8930787	IRAK1BP1	protein_coding
0,979961	PHIP	protein_coding
0,9700303	MMS22L	protein_coding
0,5848767	PNRC1	protein_coding
0,9833697	PM20D2	protein_coding
0,9997554	RARS2	protein_coding
0,8189129	TBC1D32	protein_coding
0,7816372	RNF217	protein_coding
0,5710875	ARHGAP18	protein_coding

0,7907944	SLC2A12	protein_coding
0,7823793	SHPRH	protein_coding
0,7214887	AIG1	protein_coding
0,6604792	DYNLT1	protein_coding
0,7081667	TIAM2	protein_coding
0,8786615	TMEM181	protein_coding
0,9169269	WTAP	protein_coding
0,2778507	ZMYM4	protein_coding
0,6104614	ARMT1	protein_coding
0,4600881	VWDE	protein_coding
0,8062191	GNA12	protein_coding
0,8619772	WASH2P	transcribed_unprocessed_pseudogene
0,8786805	CCZ1B	protein_coding
0,8847715	RBAK	protein_coding
0,887128	CREB5	protein_coding
0,5347705	EGFR	protein_coding
0,146655	IGFBP3	protein_coding
0,6468834	PURB	protein_coding
0,3459301	MDH2	protein_coding
0,5998801	NIPSNAP2	protein_coding
0,5920116	CCT6A	protein_coding
0,9828271	ZNF92	protein_coding
0,8212084	TMEM168	protein_coding
0,7135515	ASB15	protein_coding
0,4413743	GIGYF1	protein_coding
0,2410408	TRIM4	protein_coding
0,3150284	MEPCE	protein_coding
0,6225102	TMEM209	protein_coding
0,2681506	TMEM140	protein_coding
0,3814605	TLK2	protein_coding
0,8698319	NOM1	protein_coding
0,8582779	LUC7L2	protein_coding
0,8915993	DENND2A	protein_coding
0,1140347	SH3KBP1	protein_coding
0,4307688	TMEM47	protein_coding
0,2966679	CASK	protein_coding
0,9411751	KDM6A	protein_coding
0,6785825	MSN	protein_coding
0,8997937	HDAC8	protein_coding
0,3951339	SLC16A2	protein_coding
0,1149889	DIPK2B	protein_coding
0,8238032	KRBOX4	protein_coding
0,2354584	NDUFB11	protein_coding
0,7477503	ZNF41	protein_coding
0,4318183	ZMYM3	protein_coding
0,5269672	TAF1	protein_coding
0,6140495	NONO	protein_coding
0,4157638	OGT	protein_coding
0,8992483	SNX12	protein_coding
0,1890424	ITGB1BP2	protein_coding
0,9951314	ZNF711	protein_coding
0,9670595	DIAPH2	protein_coding
0,8472938	PRPS1	protein_coding
0,8289129	DOCK11	protein_coding
0,7136494	RBMX	protein_coding
0,7362379	MCPH1	protein_coding
0,4151093	MFHAS1	protein_coding
0,7816527	FBXO25	protein_coding
0,9194718	CETN2	protein_coding

0,4324321	RPL10	protein_coding
0,9666028	CSGALNACT1	protein_coding
0,6673583	ATP6V1B2	protein_coding
0,628921	CCDC25	protein_coding
0,8626487	HMBOX1	protein_coding
0,7948949	SLC25A37	protein_coding
0,7087026	CHMP7	protein_coding
0,7114715	DOCK5	protein_coding
0,5780824	PLPBP	protein_coding
0,5869808	ERLIN2	protein_coding
0,8575378	PXDNL	protein_coding
0,8301309	TACC1	protein_coding
0,7530415	PLPP5	protein_coding
0,613355	NSD3	protein_coding
0,8189129	TRIM55	protein_coding
0,6785825	LACTB2	protein_coding
0,9465232	TERF1	protein_coding
0,993752	RPL7	protein_coding
0,8813224	MTDH	protein_coding
0,5265966	LRP12	protein_coding
0,6600808	EBAG9	protein_coding
0,554581	POLR2K	protein_coding
0,943695	EIF3H	protein_coding
0,9900663	UTP23	protein_coding
0,0757434	NDUFB9	protein_coding
0,6835435	TATDN1	protein_coding
0,9377639	ZNF7	protein_coding
0,5046858	VLDLR	protein_coding
0,6053653	AK3	protein_coding
0,9745853	UHRF2	protein_coding
0,9406097	NFIB	protein_coding
0,7558125	PLIN2	protein_coding
0,969384	HAUS6	protein_coding
0,5623252	C9orf72	protein_coding
0,6988495	ZCCHC7	protein_coding
0,4246004	CBWD5	protein_coding
0,578003	CEP78	protein_coding
0,9982092	NTRK2	protein_coding
0,7213265	AUH	protein_coding
0,7558125	MFSD14B	protein_coding
0,645515	AOPEP	protein_coding
0,7114949	ZNF462	protein_coding
0,9781148	INIP	protein_coding
0,4887663	UGCG	protein_coding
0,3803906	SNX30	protein_coding
0,2073391	STOM	protein_coding
0,5688668	GSN	protein_coding
0,512885	MRRF	protein_coding
0,6828036	ALAD	protein_coding
0,5402971	POLE3	protein_coding
0,7010516	SURF4	protein_coding
0,3466656	SURF1	protein_coding
0,9334172	SURF6	protein_coding
0,8847715	MED22	protein_coding
0,5723744	RPL7A	protein_coding
0,5347705	GTF3C5	protein_coding
0,4477996	PTGES2	protein_coding
0,8375248	CIZ1	protein_coding
0,645515	SLC25A25	protein_coding

0,7836389	SH3GLB2	protein_coding
0,8721867	MIGA2	protein_coding
0,5140003	GPR107	protein_coding
0,7213265	SEC16A	protein_coding
0,8661642	DPH7	protein_coding
0,5358437	NOTCH1	protein_coding
0,7542355	NACC2	protein_coding
0,6676251	USP6NL	protein_coding
0,4521055	COMMD3	protein_coding
0,5647201	MSRB2	protein_coding
0,9406097	FAM171A1	protein_coding
0,849909	MINDY3	protein_coding
0,7318327	RSU1	protein_coding
0,3640941	ST8SIA6	protein_coding
0,9233889	PARD3	protein_coding
0,9352317	ZEB1	protein_coding
0,9865636	NRBF2	protein_coding
0,9738194	POLR3A	protein_coding
0,860343	HERC4	protein_coding
0,0088417	LRMDA	protein_coding
0,86551	CAMK2G	protein_coding
0,8846329	GLUD1	protein_coding
0,8992483	ANKRD1	protein_coding
0,5415117	RPP30	protein_coding
0,6890498	FRA10AC1	protein_coding
0,7126808	ADD3	protein_coding
0,9946475	DNAJB12	protein_coding
0,2819431	EIF4EBP2	protein_coding
0,5245497	TCF7L2	protein_coding
0,9334172	GSTO1	protein_coding
0,5891921	PPRC1	protein_coding
0,9802452	ITPRIP	protein_coding
0,2945575	CNNM2	protein_coding
0,8839518	PDCD11	protein_coding
0,9491167	BTBD10	protein_coding
0,1119378	ADM	protein_coding
0,7942035	LIN7C	protein_coding
0,5686994	HSD17B12	protein_coding
0,2061005	APIP	protein_coding
0,9245441	PAMR1	protein_coding
0,7558125	DGKZ	protein_coding
0,7561341	EIF3M	protein_coding
0,8930787	TNKS1BP1	protein_coding
0,7444016	SERPING1	protein_coding
0,9221401	SSRP1	protein_coding
0,5675917	PTPRJ	protein_coding
0,621682	ARFGAP2	protein_coding
0,7500896	CELF1	protein_coding
0,3787575	HIKESHI	protein_coding
0,3459301	SESN3	protein_coding
0,5164856	ENDOD1	protein_coding
0,6867028	CCDC82	protein_coding
0,9387004	SERPINH1	protein_coding
0,7490465	INTS4	protein_coding
0,8745367	PAK1	protein_coding
0,6383874	RPS3	protein_coding
0,7558125	ZC3H12C	protein_coding
0,7359516	TTC12	protein_coding
0,9997651	NCAM1	protein_coding

0,9463928	NPAT	protein_coding
0,9169269	ATM	protein_coding
0,8221205	AASDHPPT	protein_coding
0,6719615	SLX4IP	protein_coding
0,8921625	LAMTOR1	protein_coding
0,6961817	HYOU1	protein_coding
0,7885692	KAT14	protein_coding
0,9928407	MTA2	protein_coding
0,9054206	TMEM138	protein_coding
0,5557127	FADS1	protein_coding
0,8578793	EML3	protein_coding
0,8500846	CPSF7	protein_coding
0,1712026	EI24	protein_coding
0,9406097	FEZ1	protein_coding
0,4464427	ESAM	protein_coding
0,7558125	MPZL2	protein_coding
0,9781971	SIDT2	protein_coding
0,6999614	TAGLN	protein_coding
0,771306	JPH2	protein_coding
0,3150284	COMMD7	protein_coding
0,9377639	SOGA1	protein_coding
0,983457	LSM14B	protein_coding
0,9702772	YTHDF1	protein_coding
0,3792977	TRPT1	protein_coding
0,8268614	MRPL49	protein_coding
0,5777334	FAU	protein_coding
0,2318378	TM7SF2	protein_coding
0,4894579	VPS51	protein_coding
0,621558	TAOK2	protein_coding
0,8330359	TMEM219	protein_coding
0,7571978	MPP7	protein_coding
0,8189129	ITGB1	protein_coding
0,330331	CWC15	protein_coding
0,8498626	ARID5B	protein_coding
0,8447416	DCUN1D2	protein_coding
0,0132445	TMCO3	protein_coding
0,6184746	LATS2	protein_coding
0,7518452	SAP18	protein_coding
0,7431559	ADGRL3	protein_coding
0,654521	KIAA1328	protein_coding
0,933129	MIA2	protein_coding
0,5111636	HNMT	protein_coding
0,3830886	PDCD4	protein_coding
0,7238093	DLG2	protein_coding
0,1738303	PRSS23	protein_coding
0,3943278	MTMR12	protein_coding
0,7611529	PPP1R1C	protein_coding
0,3019122	CCT5	protein_coding
0,5187247	ATPSCKMT	protein_coding
0,1692705	DOCK1	protein_coding
0,2190274	DIXDC1	protein_coding
0,3514523	DLAT	protein_coding
0,4498511	NKAPD1	protein_coding
0,4151093	TIMM8B	protein_coding
0,654521	PTS	protein_coding
0,5688668	PIP4K2A	protein_coding
0,9660874	FOXO1	protein_coding
0,2506935	CRIM1	protein_coding
0,7987424	SEC24D	protein_coding

0,7010516	UBC	protein_coding
0,9408651	ITPR1	protein_coding
0,8330359	DCP1B	protein_coding
0,8992483	CACNA1C	protein_coding
0,6249095	THRB	protein_coding
0,3194617	NGLY1	protein_coding
0,330331	OXSM	protein_coding
0,579213	UEVLD	protein_coding
0,7352523	C12orf45	protein_coding
0,7057791	TMEM263	protein_coding
0,9463209	UBE3B	protein_coding
0,290432	ANK3	protein_coding
0,9745679	IPMK	protein_coding
0,9312186	PLBD2	protein_coding
0,5998801	DLG5	protein_coding
0,9808899	SLC2A13	protein_coding
0,5776471	GXYLT1	protein_coding
0,9738194	TWF1	protein_coding
0,9738194	DIP2C	protein_coding
0,7805908	EIF4E	protein_coding
0,654521	MAGI1	protein_coding
0,5412806	CSNK1G3	protein_coding
0,9771336	SRFBP1	protein_coding
0,9754097	AKAP6	protein_coding
0,435547	FAM177A1	protein_coding
0,7972313	MBIP	protein_coding
0,7910551	MIPOL1	protein_coding
0,8373584	EXT2	protein_coding
0,6305527	TMEM18	protein_coding
0,2073391	NDUFC2	protein_coding
0,5707477	ME3	protein_coding
0,9977992	NUBPL	protein_coding
0,7791166	NEK7	protein_coding
0,8831297	FER	protein_coding
0,855954	VIPAS39	protein_coding
0,2394158	ANKRD50	protein_coding
0,9998478	UPF2	protein_coding
0,4887663	CDC123	protein_coding
0,799134	SCLT1	protein_coding
0,5964345	CCDC3	protein_coding
0,9954421	FRMD4A	protein_coding
0,8620615	EPS8	protein_coding
0,7228815	ACAD8	protein_coding
0,624851	THYN1	protein_coding
0,9484395	VPS26B	protein_coding
0,8062191	NCAPD3	protein_coding
0,7589066	VTI1A	protein_coding
0,9919804	QDPR	protein_coding
0,6701082	FAM160B1	protein_coding
0,7980625	QTRT2	protein_coding
0,654521	ZNF827	protein_coding
0,621682	EDNRA	protein_coding
0,6254273	NR3C2	protein_coding
0,9476094	AKR1C2	protein_coding
0,6563172	KIN	protein_coding
0,9033043	PIGF	protein_coding
0,4309527	ANKAR	protein_coding
0,5245497	INPP1	protein_coding
0,3640941	MFSD6	protein_coding

0,9621537	RNF144A	protein_coding
0,7029174	ASAP2	protein_coding
0,9439974	ADAM17	protein_coding
0,8971058	FLI1	protein_coding
0,8386162	WWC2	protein_coding
0,9765371	ACSL1	protein_coding
0,271425	SLC25A4	protein_coding
0,2179973	BICD1	protein_coding
0,9533676	SAV1	protein_coding
0,855954	NBAS	protein_coding
0,305334	ZNF385D	protein_coding
0,6749061	GUF1	protein_coding
0,9087161	SACS	protein_coding
0,9536474	CENPJ	protein_coding
0,4477996	PARP8	protein_coding
0,571249	GFRA1	protein_coding
0,427478	CACUL1	protein_coding
0,9296784	DST	protein_coding
0,8017181	TIAL1	protein_coding
0,7190863	BAG3	protein_coding
0,7611529	SCHIP1	protein_coding
0,9385376	RABGAP1L	protein_coding
0,81614	TLCD4	protein_coding
0,8244461	FAM168B	protein_coding
0,2332141	PTPN14	protein_coding
0,9709611	AC073869.1	transcribed_processed_pseudogene
0,913228	MGAT5	protein_coding
0,9621537	GPATCH11	protein_coding
0,7340252	HSPB8	protein_coding
0,6613295	OBI1	protein_coding
0,4932632	SETBP1	protein_coding
0,7688687	ARL14EP	protein_coding
0,7542355	EPG5	protein_coding
0,4806087	ATP5F1A	protein_coding
0,56634	C18orf25	protein_coding
0,8885864	PDK1	protein_coding
0,7546576	PDE3B	protein_coding
0,8522739	TGOLN2	protein_coding
0,673546	UHMK1	protein_coding
0,391095	SPOCK1	protein_coding
0,4020941	TADA1	protein_coding
0,7905225	GUCY1A2	protein_coding
0,9583708	CWF19L2	protein_coding
0,6313749	JMY	protein_coding
0,9233889	HOMER1	protein_coding
0,9771336	ZNF776	protein_coding
0,9142106	ZNF256	protein_coding
0,7558125	DCLRE1C	protein_coding
0,7842706	NMT2	protein_coding
0,445647	USP12	protein_coding
0,5440543	CCDC50	protein_coding
0,6244656	ZFP36L2	protein_coding
0,7291201	PAN3	protein_coding
0,3640941	PLEKHH2	protein_coding
0,7836389	PFKM	protein_coding
0,8887908	TMEM123	protein_coding
0,6516554	IGSF10	protein_coding
0,4089968	SPEF2	protein_coding
0,1107554	SPARCL1	protein_coding

0,8219774	MBNL1	protein_coding
0,3695527	NADK2	protein_coding
0,730414	GPD1L	protein_coding
0,8387664	GJA1	protein_coding
0,7673893	SLC30A6	protein_coding
0,7546576	PELO	protein_coding
0,7240635	RASGRP3	protein_coding
0,5776471	SAR1B	protein_coding
0,9768456	GPR180	protein_coding
0,4740961	WDR78	protein_coding
0,9680323	FARP1	protein_coding
0,9424246	IFIT5	protein_coding
0,1895063	SLC16A12	protein_coding
0,9364287	PANK1	protein_coding
0,9928407	HNRNPDL	protein_coding
0,9463209	UTRN	protein_coding
0,5510556	PTPRK	protein_coding
0,6199488	GGPS1	protein_coding
0,9142106	ZNF117	protein_coding
0,6718649	RAD17	protein_coding
0,671498	MED21	protein_coding
0,9115427	PLOD2	protein_coding
0,4750209	ADGRA3	protein_coding
0,8801863	SREK1IP1	protein_coding
0,7291201	CWC27	protein_coding
0,1642927	MR1	protein_coding
0,6333516	SRP19	protein_coding
0,9792506	CDYL	protein_coding
0,993752	CARHSP1	protein_coding
0,7118106	TXNDC11	protein_coding
0,8996732	DAB2	protein_coding
0,1382998	BCL2L11	protein_coding
0,8823583	ANAPC1	protein_coding
0,330331	CAST	protein_coding
0,6287358	SCOC	protein_coding
0,4726081	CLGN	protein_coding
0,7940001	CETN3	protein_coding
0,7154026	SMARCA5	protein_coding
0,9883187	RGPD3	protein_coding
0,9310151	RASSF3	protein_coding
0,9248086	HNRNPU	protein_coding
0,8189129	RANBP2	protein_coding
0,9746059	AHCTF1	protein_coding
0,5641983	MERTK	protein_coding
0,7104968	TMEM87B	protein_coding
0,1431227	NR4A2	protein_coding
0,349409	PLA2R1	protein_coding
0,7010516	RBMS1	protein_coding
0,5848767	SLC25A27	protein_coding
0,7472131	FAM49B	protein_coding
0,947809	ASAP1	protein_coding
0,8575378	TRAPPC8	protein_coding
0,784306	LPCAT1	protein_coding
0,5625279	ADPRHL1	protein_coding
0,9548854	UBP1	protein_coding
0,7072223	RMND5A	protein_coding
0,624851	PTPRD	protein_coding
0,6844359	CNKSR3	protein_coding
0,6513411	CFDP1	protein_coding

0,8099847	ZDHHC7	protein_coding
0,9925374	JAZF1	protein_coding
0,7125689	CMIP	protein_coding
0,8436853	SPHKAP	protein_coding
0,6563172	PID1	protein_coding
0,8941532	TRIP12	protein_coding
0,3850967	CEBPG	protein_coding
0,3697716	DDAH1	protein_coding
0,7558125	SREK1	protein_coding
0,9761152	CHD1	protein_coding
0,887128	DGKE	protein_coding
0,702199	HS2ST1	protein_coding
0,7444016	MSI2	protein_coding
0,8695843	CACNA2D1	protein_coding
0,9603194	NUS1	protein_coding
0,8722519	PPP2R5E	protein_coding
0,7213265	IMPACT	protein_coding
0,8746152	TBCEL	protein_coding
0,5456715	ANKH	protein_coding
0,9486004	OTULIN	protein_coding
0,9784835	ROBO4	protein_coding
0,9709611	TBRG1	protein_coding
0,160824	RETREG1	protein_coding
0,5869808	TOMM70	protein_coding
0,8834615	ABI3BP	protein_coding
0,7314802	ANGPT1	protein_coding
0,472118	PITPNC1	protein_coding
0,8255291	CC2D1B	protein_coding
0,1872555	PRKCA	protein_coding
0,9237476	CEP112	protein_coding
0,2419676	ABCA9	protein_coding
0,7163823	ABCA6	protein_coding
0,2644142	ABCA10	protein_coding
0,6785825	ABCA5	protein_coding
0,0486965	UCHL1	protein_coding
0,7980597	MIA3	protein_coding
0,5998801	DISP1	protein_coding
0,90504	TNIK	protein_coding
0,5347705	NEIL2	protein_coding
0,7682033	PGM5	protein_coding
0,9934576	OBSCN	protein_coding
0,8383977	LONRF1	protein_coding
0,1100299	ENAH	protein_coding
0,4971845	PPP1R3A	protein_coding
0,9233889	CCSAP	protein_coding
0,5964345	SH3RF1	protein_coding
0,9944531	BUB3	protein_coding
0,7762876	DIPK1A	protein_coding
0,097271	ATP5MC3	protein_coding
0,2984194	CNTNAP3B	protein_coding
0,9341044	PDLIM3	protein_coding
0,6527079	SORBS2	protein_coding
0,2205761	ELOC	protein_coding
0,8992483	CEP170P1	transcribed_unprocessed_pseudogene
0,3558016	CXADR	protein_coding
0,9917279	C21orf91	protein_coding
0,6591379	L3MBTL4	protein_coding
0,806856	PDE1C	protein_coding
0,2560075	MRPL39	protein_coding

0,713782	JAM2	protein_coding
0,1277681	ATP5PF	protein_coding
0,9671548	GABPA	protein_coding
0,5768067	ADAMTS1	protein_coding
0,7744962	ADAMTS5	protein_coding
0,4602818	TSEN2	protein_coding
0,0606893	XPC	protein_coding
0,90504	CCDC174	protein_coding
0,9928407	FGD5	protein_coding
0,9792506	FLCN	protein_coding
0,3830653	DPH3	protein_coding
0,4835164	OXNAD1	protein_coding
0,437327	PLCL2	protein_coding
0,6591127	PPP4R1	protein_coding
0,5600133	APCDD1	protein_coding
0,9536474	CCDC144B	transcribed_unprocessed_pseudogene
0,6087679	MPPE1	protein_coding
0,9081173	CCDC144CP	transcribed_processed_pseudogene
0,5207698	RAB6B	protein_coding
0,7444016	ACSS1	protein_coding
0,9450135	ANKRD40	protein_coding
0,4008367	VOPP1	protein_coding
0,7258802	APOOL	protein_coding
0,2072047	CYP2U1	protein_coding
0,4033416	AK9	protein_coding
0,7912354	KLF10	protein_coding
0,7271508	AZIN1	protein_coding
0,997931	ATP6V1C1	protein_coding
0,4312749	PIP4P2	protein_coding
0,7816527	OTUD6B	protein_coding
0,54642	CDK19	protein_coding
0,1129731	GTF3C6	protein_coding
0,3691605	TTC39B	protein_coding
0,9411751	AGPAT5	protein_coding
0,936881	MMS19	protein_coding
0,8118149	PI4K2A	protein_coding
0,623981	ZFYVE27	protein_coding
0,5909829	SLC25A28	protein_coding
0,8422988	HSPA13	protein_coding
0,9756408	USP25	protein_coding
0,7824563	GRAMD2B	protein_coding
0,7125689	ZCCHC10	protein_coding
0,5773589	C16orf87	protein_coding
0,817424	MOV10	protein_coding
0,7956173	RHOC	protein_coding
0,7842247	DBI	protein_coding
0,9508384	SLC16A1	protein_coding
0,4076876	HEATR3	protein_coding
0,9917279	NIFK	protein_coding
0,8500846	OXA1L	protein_coding
0,8799382	LARP1	protein_coding
0,8790123	CNOT8	protein_coding
0,9933219	MIER3	protein_coding
0,4835164	NUP205	protein_coding
0,8500846	ZKSCAN2	protein_coding
0,5433886	PIK3AP1	protein_coding
0,8117718	RBM45	protein_coding
0,7940001	TTN	protein_coding
0,0286685	VSIG4	protein_coding

0,6634961	PDIA4	protein_coding
0,5995805	KCTD18	protein_coding
0,8712074	FAM126B	protein_coding
0,8993494	C2CD6	protein_coding
0,6662301	TMEM237	protein_coding
0,0015119	FZD7	protein_coding
0,9082846	RNF20	protein_coding
0,8500846	PPARGC1B	protein_coding
0,9156816	ELMO1	protein_coding
0,9644268	SLC26A2	protein_coding
0,5768067	MED7	protein_coding
0,9166492	RRAGA	protein_coding
0,7713242	PXYLP1	protein_coding
0,8992483	RASA2	protein_coding
0,5474737	RMND1	protein_coding
0,3371364	SLA	protein_coding
0,9142106	TMBIM4	protein_coding
0,4063431	VBP1	protein_coding
0,5622225	CLIC2	protein_coding
0,9264426	MICU3	protein_coding
0,7311739	VPS37A	protein_coding
0,645515	PSD3	protein_coding
0,8062191	CARNMT1	protein_coding
0,9496064	MCU	protein_coding
0,3776155	ELMSAN1	protein_coding
0,5664111	CFAP70	protein_coding
0,6653209	GNAQ	protein_coding
0,3280381	UGT2B4	protein_coding
0,4600881	MMP16	protein_coding
0,0839464	ADK	protein_coding
0,8330359	DCK	protein_coding
0,9854182	DPY19L4	protein_coding
0,5347705	NDUFAF6	protein_coding
0,5028151	DRAM2	protein_coding
0,7743102	ADAMTSL3	protein_coding
0,3880092	ART3	protein_coding
0,8117718	WHAMM	protein_coding
0,7546576	N6AMT1	protein_coding
0,2499691	RWDD2B	protein_coding
0,6500583	USP16	protein_coding
0,479851	CCT8	protein_coding
0,8919058	BACH1	protein_coding
0,458152	TSPAN7	protein_coding
0,9998478	SCAF4	protein_coding
0,4835164	RPGR	protein_coding
0,6903585	ANKRD9	protein_coding
0,1412577	ATP5MPL	protein_coding
0,4847278	PCDH1	protein_coding
0,9997039	SH3RF2	protein_coding
0,1773741	UQCRB	protein_coding
0,0286685	MTERF3	protein_coding
0,0075305	PTDSS1	protein_coding
0,692146	RPL30	protein_coding
0,4748134	SUPV3L1	protein_coding
0,7418156	FAM122B	protein_coding
0,5245497	EEF1A1	protein_coding
0,8117718	HK1	protein_coding
0,9578269	PHF6	protein_coding
0,7915374	CD109	protein_coding

0,4835164	UBE2L6	protein_coding
0,9645685	ZDHHC5	protein_coding
0,9894339	ZFAND3	protein_coding
0,4112262	NPTN	protein_coding
0,9391828	KAT6B	protein_coding
0,9761718	SAMD8	protein_coding
0,0811214	RAB11FIP1	protein_coding
0,8189129	UTP14A	protein_coding
0,330331	AIFM1	protein_coding
0,9914435	BAG4	protein_coding
0,9533049	TBC1D31	protein_coding
0,3801685	WDYHV1	protein_coding
0,9759101	ATAD2	protein_coding
0,9965961	FBXO32	protein_coding
0,5777334	NSMCE2	protein_coding
0,874444	COX6A2	protein_coding
0,1536666	MALSU1	protein_coding
0,4311333	VPS8	protein_coding
0,6951729	GALK2	protein_coding
0,9781148	EIF4A2	protein_coding
0,3280381	RPUSD3	protein_coding
0,922549	SEC13	protein_coding
0,9792506	EXOG	protein_coding
0,8836774	NTAN1	protein_coding
0,6867028	NMNAT2	protein_coding
0,7956173	ZFYVE9	protein_coding
0,9928407	SMG1	protein_coding
0,6999614	FCHO2	protein_coding
0,7763012	RBPMS	protein_coding
0,9781971	TIMP4	protein_coding
0,7940001	ODR4	protein_coding
0,5336185	CPT2	protein_coding
0,9248086	NECAP2	protein_coding
0,90504	PAXIP1	protein_coding
0,9463928	STEAP2	protein_coding
0,8552255	SSBP3	protein_coding
0,3558016	CLDN12	protein_coding
0,9982092	MMP14	protein_coding
0,4957019	FZD1	protein_coding
0,6527079	GATAD1	protein_coding
0,7663988	DHRS4	protein_coding
0,9998478	DDX19B	protein_coding
0,565522	ST3GAL2	protein_coding
0,9210173	AASDH	protein_coding
0,5111636	CACNA2D3	protein_coding
0,8888699	RNF111	protein_coding
0,933129	MYO1E	protein_coding
0,895571	APPL1	protein_coding
0,4524087	AFAP1L1	protein_coding
0,4732706	TSC22D3	protein_coding
0,8436853	VPS26C	protein_coding
0,9928407	DYRK1A	protein_coding
0,7972313	ERG	protein_coding
0,4767674	ETS2	protein_coding
0,9700303	TSPAN18	protein_coding
0,9171648	SLC35B2	protein_coding
0,9496064	TMEM164	protein_coding
0,2834613	MX1	protein_coding
0,4806087	C2CD2	protein_coding

0,8575378	TAB3	protein_coding
0,4032255	SLC38A10	protein_coding
0,9919069	PALM2-AKAP2	protein_coding
0,0486965	DGKI	protein_coding
0,0413597	SNX22	protein_coding
0,4498511	UBN2	protein_coding
0,7318327	BRAF	protein_coding
0,8117718	WDR19	protein_coding
0,8183781	SLC37A3	protein_coding
0,8939634	AP3S2	protein_coding
0,9082846	FMNL2	protein_coding
0,4040485	SPPL3	protein_coding
0,9840915	RAB28	protein_coding
0,8695843	PANK4	protein_coding
0,3881918	RER1	protein_coding
0,7662231	SKI	protein_coding
0,9781971	WIPI2	protein_coding
0,8394189	AGAP1	protein_coding
0,8397121	BABAM2	protein_coding
0,4906292	TRIM63	protein_coding
0,4032255	MRPL17	protein_coding
0,5953122	PTPDC1	protein_coding
0,9998478	NCK1	protein_coding
0,6505186	TPRG1L	protein_coding
0,3316892	PRXL2C	protein_coding
0,9233889	CNNM4	protein_coding
0,8197846	EYA3	protein_coding
0,7984578	FANCC	protein_coding
0,6440403	MRAS	protein_coding
0,4750209	WASF2	protein_coding
0,9464798	CLSTN2	protein_coding
0,7137584	COLEC12	protein_coding
0,6610925	RNF207	protein_coding
0,5276954	CUL4B	protein_coding
0,4311333	GPRASP2	protein_coding
0,4161706	AUTS2	protein_coding
0,7740102	SHROOM4	protein_coding
0,9953531	MITD1	protein_coding
0,9481776	EIF5B	protein_coding
0,9406097	CNOT11	protein_coding
0,3881918	AHCYL2	protein_coding
0,4521055	B4GALT5	protein_coding
0,4585504	TSR2	protein_coding
0,8941532	PPP1R9A	protein_coding
0,4477996	DYNC1I1	protein_coding
0,5011485	TMED4	protein_coding
0,4954628	PPP1R15B	protein_coding
0,943695	COPG2	protein_coding
0,8062191	EMSY	protein_coding
0,8425029	GPAT4	protein_coding
0,9364287	ZSCAN12	protein_coding
0,9792506	TAGLN2	protein_coding
0,9481776	ELK4	protein_coding
0,7171927	NBL1	protein_coding
0,7842706	F11R	protein_coding
0,9781971	USF1	protein_coding
0,6462233	NIT1	protein_coding
0,7291201	DEDD	protein_coding
0,1712026	EDA	protein_coding

0,8863508	PINK1	protein_coding
0,7558125	DMTN	protein_coding
0,0385201	ADAMTS4	protein_coding
0,8575378	FAM160B2	protein_coding
0,2737133	NDUFS2	protein_coding
0,1574812	FCER1G	protein_coding
0,138108	TOMM40L	protein_coding
0,8930787	CCAR2	protein_coding
0,5073762	CACHD1	protein_coding
0,9410289	CDC42SE2	protein_coding
0,7940988	RAPGEF6	protein_coding
0,6101378	EPB41	protein_coding
0,8062191	ALG8	protein_coding
0,9142106	FBXW5	protein_coding
0,9112805	SYNJ1	protein_coding
0,9270004	PAXBP1	protein_coding
0,9944531	MRPL10	protein_coding
0,9998478	IFNGR2	protein_coding
0,4831898	GART	protein_coding
0,9928407	SON	protein_coding
0,5510556	CSRP1	protein_coding
0,8255851	C1QC	protein_coding
0,0998266	ATP5MC1	protein_coding
0,3212727	RCAN1	protein_coding
0,47585	UBE2Z	protein_coding
0,4835164	SNF8	protein_coding
0,9450135	RUNX1	protein_coding
0,284683	ACTC1	protein_coding
0,9182782	MORC3	protein_coding
0,9424246	HLCS	protein_coding
0,8318519	ARHGAP27	protein_coding
0,9745853	ADPGK	protein_coding
0,9112805	PTMS	protein_coding
0,9463209	ADIPOR1	protein_coding
0,4806087	CYB5R1	protein_coding
0,4835164	PSMD4	protein_coding
0,8062191	PSMB4	protein_coding
0,9142106	BTG2	protein_coding
0,6285359	HK2	protein_coding
0,9804708	C1R	protein_coding
0,6867028	ALDH4A1	protein_coding
0,7956408	STARD9	protein_coding
0,7444016	THEM4	protein_coding
0,8859349	UBR1	protein_coding
0,9853651	AMFR	protein_coding
0,7431973	MED8	protein_coding
0,7747743	RSPRY1	protein_coding
0,8060644	GPBP1L1	protein_coding
0,5972873	NAE1	protein_coding
0,7954765	TMEM69	protein_coding
0,5062136	EFCAB14	protein_coding
0,9917279	CTBP1	protein_coding
0,9002008	ATP6V0D1	protein_coding
0,8130747	RGS12	protein_coding
0,931978	PSKH1	protein_coding
0,7589066	ZYX	protein_coding
0,7291201	ABR	protein_coding
0,3049645	CCDC117	protein_coding
0,7132753	NPR2	protein_coding

0,9781148	ZNF235	protein_coding
0,9810229	GNE	protein_coding
0,9379013	ARHGAP35	protein_coding
0,611428	CALM3	protein_coding
0,9463928	DFFA	protein_coding
0,6371005	BSDC1	protein_coding
0,4799252	SSU72	protein_coding
0,7154026	ZNF362	protein_coding
0,3150284	FNDC5	protein_coding
0,796666	CPAMD8	protein_coding
0,8698319	VMA21	protein_coding
0,7397205	KALRN	protein_coding
0,3803906	ABCG1	protein_coding
0,2073391	NDUFV3	protein_coding
0,5909829	U2AF1	protein_coding
0,931978	RRP1B	protein_coding
0,7634906	PDXK	protein_coding
0,7558125	CSTB	protein_coding
0,7921707	AGPAT3	protein_coding
0,9702772	TRAPPC10	protein_coding
0,9307184	GAB3	protein_coding
0,8481719	GATD3A	protein_coding
0,7611529	ITGB2	protein_coding
0,9933479	LSS	protein_coding
0,9481776	MCM3AP	protein_coding
0,2656432	PCNT	protein_coding
0,7785565	DIP2A	protein_coding
0,5768067	PRMT2	protein_coding
0,2068936	ZNF208	protein_coding
0,9804708	ZNF761	protein_coding
0,7135515	C19orf47	protein_coding
0,9011545	RDH13	protein_coding
0,5245497	ZER1	protein_coding
0,6168923	PLPP7	protein_coding
0,855954	TAOK1	protein_coding
0,9169269	SIK3	protein_coding
0,5117223	JAML	protein_coding
0,9583561	PCSK7	protein_coding
0,7611529	SAFB	protein_coding
0,5666553	S100A1	protein_coding
0,726381	CHTOP	protein_coding
0,8015756	ZBTB7B	protein_coding
0,4894579	FLAD1	protein_coding
0,9003006	SHC1	protein_coding
0,747138	VPS11	protein_coding
0,5166442	ADAR	protein_coding
0,6383874	IL6R	protein_coding
0,9210173	UBE2Q1	protein_coding
0,9804708	ANO10	protein_coding
0,4684539	FDPS	protein_coding
0,7763012	SLC25A44	protein_coding
0,8992483	LMNA	protein_coding
0,9296784	NBEAL2	protein_coding
0,9333994	UBQLN4	protein_coding
0,998169	MYL3	protein_coding
0,3520154	GPATCH4	protein_coding
0,4433415	AZGP1	protein_coding
0,9781971	ZNF394	protein_coding
0,9767278	VPS28	protein_coding

0,5453247	PWWP3A	protein_coding
0,9998478	ZNF333	protein_coding
0,3358222	MRNIP	protein_coding
0,3765746	SQSTM1	protein_coding
0,3792977	MGAT4B	protein_coding
0,8914719	RPL8	protein_coding
0,997931	MAML1	protein_coding
0,6588074	NAPEPLD	protein_coding
0,3894807	PSMC2	protein_coding
0,6563172	DVL3	protein_coding
0,9260532	AP2M1	protein_coding
0,9463209	ABCF3	protein_coding
0,997931	PCYT1A	protein_coding
0,3358601	BDH1	protein_coding
0,4765298	COX7A1	protein_coding
0,931978	ZNF382	protein_coding
0,3831225	PLXDC1	protein_coding
0,5024483	SAP30BP	protein_coding
0,8035834	ACOX1	protein_coding
0,458152	PRPSAP1	protein_coding
0,9700303	SRSF2	protein_coding
0,2933095	ZNF577	protein_coding
0,9270004	ITGA5	protein_coding
0,2329611	MPP3	protein_coding
0,2971924	LSM12	protein_coding
0,426935	EMC10	protein_coding
0,1482066	FMNL3	protein_coding
0,7226369	LARP4	protein_coding
0,6371005	IP6K3	protein_coding
0,8578793	LEMD2	protein_coding
0,8992483	BCL6B	protein_coding
0,9316489	RPL26	protein_coding
0,1951241	SNRNP25	protein_coding
0,4422012	ZNF75A	protein_coding
0,9248086	ADCY9	protein_coding
0,8189129	CLPB	protein_coding
0,6227164	NEU3	protein_coding
0,995098	UBXN1	protein_coding
0,9956943	NXF1	protein_coding
0,6988495	STX5	protein_coding
0,7662231	RPL29	protein_coding
0,7114715	SYVN1	protein_coding
0,1100299	LRP5	protein_coding
0,6098976	CMPK1	protein_coding
0,7639764	COA7	protein_coding
0,9484395	ZYG11B	protein_coding
0,565522	CZIB	protein_coding
0,9484259	ACOT11	protein_coding
0,8295097	USP24	protein_coding
0,0895372	PLPP3	protein_coding
0,9917279	NOL9	protein_coding
0,9917279	PRKAA2	protein_coding
0,8031748	KLHL21	protein_coding
0,9081173	SELENON	protein_coding
0,8309678	AK4	protein_coding
0,9548854	JAK1	protein_coding
0,4105939	RAVER2	protein_coding
0,6867028	LZIC	protein_coding
0,2440137	FBLIM1	protein_coding

0,5347705	DHRS3	protein_coding
0,887128	LAPTM5	protein_coding
0,3358222	SDC3	protein_coding
0,9998478	PEF1	protein_coding
0,615864	RBBP4	protein_coding
0,1431468	MXRA8	protein_coding
0,9312186	NFIA	protein_coding
0,9925374	OMA1	protein_coding
0,645515	MYSM1	protein_coding
0,8721867	TM2D1	protein_coding
0,8956453	USP1	protein_coding
0,916788	FUBP1	protein_coding
0,9210173	NEXN	protein_coding
0,9781971	DNAJB4	protein_coding
0,4321437	ADGRL4	protein_coding
0,9669517	TYW3	protein_coding
0,5880659	SNX7	protein_coding
0,3572155	B3GALT2	protein_coding
0,3316892	FAM102B	protein_coding
0,8259599	C1orf52	protein_coding
0,9998478	GBP2	protein_coding
0,933129	GBP4	protein_coding
0,9033043	ZNF326	protein_coding
0,8885864	HFM1	protein_coding
0,1712026	KCNT2	protein_coding
0,6373029	AGL	protein_coding
0,9341044	VCAM1	protein_coding
0,9450135	EXTL2	protein_coding
0,6785825	SLC30A7	protein_coding
0,9551108	ZNF281	protein_coding
0,8441592	ARPC5	protein_coding
0,4894579	ZNF496	protein_coding
0,9406097	DDR2	protein_coding
0,9481776	PEA15	protein_coding
0,9792506	PEX19	protein_coding
0,8622295	NCSTN	protein_coding
0,6254273	ATF3	protein_coding
0,9953531	RBM15	protein_coding
0,5511615	IER5	protein_coding
0,8829558	SNED1	protein_coding
0,9374881	BPNT1	protein_coding
0,3514523	C1orf115	protein_coding
0,7858129	BROX	protein_coding
0,5768067	NBPF20	protein_coding
0,4141191	ACP6	protein_coding
0,8265935	TFB2M	protein_coding
0,7397205	CNST	protein_coding
0,2337466	PPP1R21	protein_coding
0,3094135	PKDCC	protein_coding
0,2354584	B3GALNT2	protein_coding
0,7190863	MAPKAPK2	protein_coding
0,7154026	CAPN2	protein_coding
0,768388	WDR26	protein_coding
0,595282	REL	protein_coding
0,8189129	PUS10	protein_coding
0,764019	PEX13	protein_coding
0,9734904	KIAA1841	protein_coding
0,4828854	RFTN2	protein_coding
0,997931	DISC1	protein_coding

0,7104968	DPY30	protein_coding
0,953175	TYW5	protein_coding
0,330331	SLC66A3	protein_coding
0,9900815	ARL5A	protein_coding
0,2403842	KCNJ3	protein_coding
0,9432348	DUSP19	protein_coding
0,7506449	CFAP36	protein_coding
0,9406097	NUP35	protein_coding
0,8941532	SMC6	protein_coding
0,8454927	VSNL1	protein_coding
0,8153751	H3F3A	protein_coding
0,3113413	COQ8A	protein_coding
0,8176511	SGCB	protein_coding
0,7569416	NOSTRIN	protein_coding
0,8653179	XIRP2	protein_coding
0,9625751	SMARCAD1	protein_coding
0,8050685	PDLIM5	protein_coding
0,6316303	RPRD2	protein_coding
0,9998478	CTSS	protein_coding
0,9253623	PACRGL	protein_coding
0,9127443	VPS72	protein_coding
0,9917279	ERCC3	protein_coding
0,5225448	RNF149	protein_coding
0,3049645	IWS1	protein_coding
0,8992483	CDC42EP3	protein_coding
0,8833312	S100A11	protein_coding
0,7894641	DHX57	protein_coding
0,54642	S100A9	protein_coding
0,3772742	CCNYL1	protein_coding
0,6604792	DCAF16	protein_coding
0,7258802	GNPDA2	protein_coding
0,9802452	PAQR3	protein_coding
0,8481719	ANTXR2	protein_coding
0,9700639	HELQ	protein_coding
0,2737133	MRPS18C	protein_coding
0,9538485	CGGBP1	protein_coding
0,4372888	GPR155	protein_coding
0,4847278	PMVK	protein_coding
0,9551108	PBXIP1	protein_coding
0,6999614	PYGO2	protein_coding
0,8992483	HIPK1	protein_coding
0,8716837	COL6A3	protein_coding
0,9544158	YY1AP1	protein_coding
0,8318664	KBTBD8	protein_coding
0,5647201	EOGT	protein_coding
0,6564675	LMOD3	protein_coding
0,5848767	NAXE	protein_coding
0,9312186	POGLUT1	protein_coding
0,6225174	ATP1A1	protein_coding
0,6574161	EIF4E3	protein_coding
0,8406321	LRRC58	protein_coding
0,7774855	FSTL1	protein_coding
0,4627775	LMOD1	protein_coding
0,7661133	TMEM183A	protein_coding
0,146655	IGFBP7	protein_coding
0,7972313	ARPC2	protein_coding
0,2533818	CCT3	protein_coding
0,8161522	SSR2	protein_coding
0,702199	NEK10	protein_coding

0,9205351	CCDC141	protein_coding
0,9705617	CWC22	protein_coding
0,6168923	AZI2	protein_coding
0,3387057	TGFBR2	protein_coding
0,768388	ANKZF1	protein_coding
0,9127443	HDAC11	protein_coding
0,645515	FBLN2	protein_coding
0,9925374	STT3B	protein_coding
0,0941174	CHCHD4	protein_coding
0,2506935	NFASC	protein_coding
0,8422763	CLASP2	protein_coding
0,202023	SUCLG1	protein_coding
0,4498511	PRKCI	protein_coding
0,7921707	MNDA	protein_coding
0,4463195	IFI16	protein_coding
0,9245441	EIF5A2	protein_coding
0,0336588	PPM1L	protein_coding
0,9997651	ICA1L	protein_coding
0,7444016	SNHG16	lncRNA
0,641691	RYBP	protein_coding
0,8189129	PPP4R2	protein_coding
0,2842973	GTPBP8	protein_coding
0,8185595	NEPRO	protein_coding
0,6563172	CADPS	protein_coding
0,9452386	WDFY3	protein_coding
0,8131645	COX18	protein_coding
0,3269911	PTPN13	protein_coding
0,445647	THOC7	protein_coding
0,4141191	PSMD6	protein_coding
0,9789395	PRICKLE2	protein_coding
0,2474819	ADAMTS9	protein_coding
0,887128	PPM1K	protein_coding
0,656867	GMPS	protein_coding
0,9316489	TIPARP	protein_coding
0,849692	CCNL1	protein_coding
0,9731251	SLMAP	protein_coding
0,5346782	RPL9	protein_coding
0,645515	SMIM14	protein_coding
0,9629003	RPP14	protein_coding
0,7550845	APBB2	protein_coding
0,9309593	CRELD1	protein_coding
0,6855507	PCOLCE2	protein_coding
0,7552965	U2SURP	protein_coding
0,9817457	MTMR14	protein_coding
0,9874145	TTC14	protein_coding
0,9357407	MTHFD2L	protein_coding
0,8269408	RCHY1	protein_coding
0,4806087	GYG1	protein_coding
0,9379013	HPS3	protein_coding
0,4740961	TM4SF18	protein_coding
0,8355525	TOPBP1	protein_coding
0,9724163	RYK	protein_coding
0,5190186	SNRK	protein_coding
0,9767278	SLC4A1AP	protein_coding
0,9828271	KIAA1143	protein_coding
0,9484395	WDR43	protein_coding
0,654521	ZDHHC3	protein_coding
0,3829653	LZTFL1	protein_coding
0,8847411	FYCO1	protein_coding

0,9233889	LRRC2	protein_coding
0,993752	FBXO40	protein_coding
0,7611529	DTX3L	protein_coding
0,8992483	ZNF148	protein_coding
0,5622112	NMNAT3	protein_coding
0,0197802	SMIM12	protein_coding
0,855954	ZMYM6	protein_coding
0,692146	YEATS2	protein_coding
0,6116281	MEAF6	protein_coding
0,8099847	SNIP1	protein_coding
0,9956922	TMEM41A	protein_coding
0,9077931	RPN1	protein_coding
0,860343	SENP2	protein_coding
0,6999614	HEYL	protein_coding
0,5029234	IFT122	protein_coding
0,8683086	BAP1	protein_coding
0,8052193	TKT	protein_coding
0,9166492	PRKCD	protein_coding
0,9828271	SFMBT1	protein_coding
0,554581	GNL3	protein_coding
0,9781971	PBRM1	protein_coding
0,7842706	TASOR	protein_coding
0,8992483	ARHGEF3	protein_coding
0,7716654	SLBP	protein_coding
0,931978	LRPAP1	protein_coding
0,5869872	UBXN7	protein_coding
0,8875885	RNF168	protein_coding
0,7182975	PIGX	protein_coding
0,7446613	AIMP1	protein_coding
0,62584	SGMS2	protein_coding
0,3482281	METAP1	protein_coding
0,9803555	DNAJB14	protein_coding
0,2026798	H2AFZ	protein_coding
0,5265966	EMCN	protein_coding
0,9802452	SLC9B2	protein_coding
0,6140495	BDH2	protein_coding
0,9481776	PGRMC2	protein_coding
0,5402971	ZNF589	protein_coding
0,8269408	PLXNB1	protein_coding
0,8058953	SHISA5	protein_coding
0,9705617	SPRY1	protein_coding
0,806905	APEH	protein_coding
0,9178514	INTU	protein_coding
0,9645838	RNF123	protein_coding
0,8318664	HSPA4L	protein_coding
0,9484395	MFSD8	protein_coding
0,7054107	ABHD18	protein_coding
0,8554227	RAD54L2	protein_coding
0,6462349	TEX264	protein_coding
0,8735371	WDR82	protein_coding
0,0640162	PITX2	protein_coding
0,9496064	C4orf3	protein_coding
0,9603194	HMGB2	protein_coding
0,8950245	HAND2	protein_coding
0,7355147	ANXA5	protein_coding
0,9917279	MAP9	protein_coding
0,4513092	GUCY1A1	protein_coding
0,8885864	FBXO8	protein_coding
0,8578622	CEP44	protein_coding

0,9145234	TMEM144	protein_coding
0,624851	GASK1B	protein_coding
0,5159574	NAA15	protein_coding
0,3706065	FAM160A1	protein_coding
0,9194718	ARFIP1	protein_coding
0,9233889	ICE1	protein_coding
0,8238032	ABCE1	protein_coding
0,8935967	OTUD4	protein_coding
0,8624018	TMEM184C	protein_coding
0,641691	PRMT9	protein_coding
0,4141191	MOCS2	protein_coding
0,9057039	EDIL3	protein_coding
0,7171006	TMEM161B	protein_coding
0,4599964	NDUFAF2	protein_coding
0,6658691	LMBRD2	protein_coding
0,7182975	NIPBL	protein_coding
0,6607313	RNF180	protein_coding
0,7118106	SLC25A46	protein_coding
0,9768456	STARD4	protein_coding
0,7956173	PGGT1B	protein_coding
0,8992483	ANKRD33B	protein_coding
0,7687197	CMBL	protein_coding
0,6778187	PRRC1	protein_coding
0,9998538	AGGF1	protein_coding
0,887319	WDR41	protein_coding
0,3816085	NDUFS4	protein_coding
0,0093815	HTR4	protein_coding
0,9477953	GRPEL2	protein_coding
0,9089634	RHOBTB3	protein_coding
0,1611222	GPX8	protein_coding
0,8019639	SERINC5	protein_coding
0,9998478	CASP3	protein_coding
0,9077931	PRIMPOL	protein_coding
0,6903585	ERAP1	protein_coding
0,9759101	CMYA5	protein_coding
0,1412577	EGFLAM	protein_coding
0,9068107	CFAP97	protein_coding
0,9463928	RICTOR	protein_coding
0,9751519	TENT2	protein_coding
0,8992483	EBF1	protein_coding
0,9998478	ANKRA2	protein_coding
0,8851892	UBLCP1	protein_coding
0,3327196	UTP15	protein_coding
0,9496064	NSA2	protein_coding
0,6053653	GFM2	protein_coding
0,9484395	CCDC127	protein_coding
0,3706065	SEPTIN8	protein_coding
0,5879554	UQCRQ	protein_coding
0,9802452	GRIK2	protein_coding
0,993752	TXLNB	protein_coding
0,8500846	CITED2	protein_coding
0,998169	CREBRF	protein_coding
0,7213265	SFXN1	protein_coding
0,7820981	PDSS2	protein_coding
0,9439974	STXBP5	protein_coding
0,9583708	PI16	protein_coding
0,6613817	TBX20	protein_coding
0,6807165	STK17A	protein_coding
0,7687197	TRA2A	protein_coding

0,7784325	GALNT10	protein_coding
0,5964345	SAP30L	protein_coding
0,6337897	RPS14	protein_coding
0,8209243	HCN1	protein_coding
0,330331	COG5	protein_coding
0,9616301	BMT2	protein_coding
0,8500092	SLU7	protein_coding
0,7136899	RP9	protein_coding
0,654521	CAMLG	protein_coding
0,9281076	ZNF12	protein_coding
0,9766993	CDCA7L	protein_coding
0,7154026	MIOS	protein_coding
0,6613295	KIAA1324L	protein_coding
0,1528801	ZNF704	protein_coding
0,0283162	FABP5	protein_coding
0,520999	COL1A2	protein_coding
0,7309156	BRI3	protein_coding
0,6254273	LMTK2	protein_coding
0,1799031	CTSB	protein_coding
0,3194617	DLC1	protein_coding
0,5267449	ADCY1	protein_coding
0,7798822	PEX2	protein_coding
0,9705617	RAD21	protein_coding
0,9898099	SPIDR	protein_coding
0,8745367	ORC5	protein_coding
0,2318378	OSGIN2	protein_coding
0,9377639	SUN1	protein_coding
0,8528913	OXR1	protein_coding
0,6549292	INTS1	protein_coding
0,7029174	SLC4A2	protein_coding
0,8996732	FASTK	protein_coding
0,7981341	PHAX	protein_coding
0,9804708	ALDH7A1	protein_coding
0,6446908	FOXK1	protein_coding
0,1523131	COX6C	protein_coding
0,9621856	YWHAZ	protein_coding
0,8545769	FZD6	protein_coding
0,5686994	DCAF13	protein_coding
0,7252511	TP53INP1	protein_coding
0,8131645	INTS8	protein_coding
0,7716654	VIRMA	protein_coding
0,5688668	FREM1	protein_coding
0,4875875	GEM	protein_coding
0,9450135	PDP1	protein_coding
0,3089259	TMEM67	protein_coding
0,7854018	WASHC5	protein_coding
0,9481776	FAM219A	protein_coding
0,6491869	SNAPC3	protein_coding
0,8183781	NUDT2	protein_coding
0,8257564	TMEM65	protein_coding
0,6745473	PSIP1	protein_coding
0,47585	CCDC171	protein_coding
0,5531846	UBAP1	protein_coding
0,8850351	SYK	protein_coding
0,284683	NIPSNAP3B	protein_coding
0,5558351	ABCA1	protein_coding
0,3640941	NFIL3	protein_coding
0,8887908	METTL2B	protein_coding
0,3399876	FXN	protein_coding

0,4179007	TMEM71	protein_coding
0,2809994	MAMDC2	protein_coding
0,933129	ALDH1A1	protein_coding
0,5622112	KDM1B	protein_coding
0,5510556	HGSNAT	protein_coding
0,6696502	GKAP1	protein_coding
0,8019639	KIF27	protein_coding
0,7271508	HNRNPK	protein_coding
0,9702772	AL353743.1	transcribed_unprocessed_pseudogene
0,5953643	SVEP1	protein_coding
0,9364287	TMEM246	protein_coding
0,9334172	ZHX1	protein_coding
0,6988495	CYBB	protein_coding
0,9995727	DYNLT3	protein_coding
0,964785	SHOC1	protein_coding
0,6613295	ASB11	protein_coding
0,3801685	PIGA	protein_coding
0,5964345	STRBP	protein_coding
0,9481776	GAPVD1	protein_coding
0,9917279	WNK2	protein_coding
0,5073158	ATP7A	protein_coding
0,1225394	NDUFB6	protein_coding
0,8375248	AQP7	protein_coding
0,9081173	NOL6	protein_coding
0,9705617	VCP	protein_coding
0,8318519	PIGO	protein_coding
0,3435112	STOML2	protein_coding
0,8834615	BRWD3	protein_coding
0,9928407	OTUD1	protein_coding
0,931978	ARHGAP12	protein_coding
0,7010516	HECTD2	protein_coding
0,6316303	FBXO33	protein_coding
0,6475905	INTS6L	protein_coding
0,1800952	SPTSSA	protein_coding
0,887319	WRN	protein_coding
0,9975676	MARCH8	protein_coding
0,749866	CFL2	protein_coding
0,9296784	SUGT1	protein_coding
0,7161818	GTF2A1	protein_coding
0,4906292	ZCCHC24	protein_coding
0,7321942	PGM2L1	protein_coding
0,6543081	FOLR2	protein_coding
0,860343	INPPL1	protein_coding
0,7687197	CRYL1	protein_coding
0,8330359	REEP3	protein_coding
0,2231549	MICU2	protein_coding
0,5777776	PCF11	protein_coding
0,8155634	RPL36AL	protein_coding
0,2127154	DEPP1	protein_coding
0,4806087	ZNF22	protein_coding
0,5768067	KLHDC2	protein_coding
0,9275549	NEMF	protein_coding
0,536593	RPUSD4	protein_coding
0,9406097	ARF6	protein_coding
0,4779341	TTC8	protein_coding
0,9477707	KBTBD6	protein_coding
0,9792506	NUDT5	protein_coding
0,9839612	BEND7	protein_coding
0,1531167	ATP5F1C	protein_coding

0,6591549	VSTM4	protein_coding
0,240225	VDAC2	protein_coding
0,9933219	PDZD8	protein_coding
0,5675917	ABRAXAS2	protein_coding
0,8500846	FAM204A	protein_coding
0,9484395	NSD1	protein_coding
0,5060008	PRDX3	protein_coding
0,8622295	ENOX2	protein_coding
0,5122163	GHITM	protein_coding
0,9516698	PMPCA	protein_coding
0,9074092	TSC1	protein_coding
0,9332245	HPRT1	protein_coding
0,9081173	BORCS5	protein_coding
0,5343413	DDX21	protein_coding
0,6613295	BMS1	protein_coding
0,7985809	JCAD	protein_coding
0,1431468	FUNDC2	protein_coding
0,624851	METTL17	protein_coding
0,6425736	NDRG2	protein_coding
0,5185243	ARHGEF40	protein_coding
0,6776791	CASP7	protein_coding
0,8563699	BTNL9	protein_coding
0,9731251	CCDC186	protein_coding
0,9176584	METTL3	protein_coding
0,9528743	TRUB1	protein_coding
0,6516554	ZFYVE1	protein_coding
0,8440006	HSPA12A	protein_coding
0,4638153	SHLD2P1	transcribed_unprocessed_pseudogene
0,8530643	ARHGAP42	protein_coding
0,9562513	ISCA2	protein_coding
0,9463928	OTOGL	protein_coding
0,8274546	PACSIN3	protein_coding
0,9839612	TTC7B	protein_coding
0,6313749	PSMC3	protein_coding
0,6999614	TC2N	protein_coding
0,9137326	CPSF2	protein_coding
0,2971084	MOAP1	protein_coding
0,7816267	IFI27	protein_coding
0,520749	CLMN	protein_coding
0,3749466	PTER	protein_coding
0,3640941	CACNB2	protein_coding
0,4724079	HACD1	protein_coding
0,9136442	ARL5B	protein_coding
0,7842706	CEP295	protein_coding
0,9345529	TAF1D	protein_coding
0,895571	ABTB2	protein_coding
0,9880319	R3HCC1L	protein_coding
0,7858129	AMOTL1	protein_coding
0,0606893	HTRA1	protein_coding
0,8386162	CEP57	protein_coding
0,9033043	TCP11L2	protein_coding
0,7611529	SPRED1	protein_coding
0,7125689	JAM3	protein_coding
0,1284788	CMTM5	protein_coding
0,4806087	ADAMTS15	protein_coding
0,3803906	GPT2	protein_coding
0,9282881	RAB8B	protein_coding
0,913228	HIF1AN	protein_coding
0,3944312	NDUFB8	protein_coding

0,9364287	FBN1	protein_coding
0,887319	BRD7	protein_coding
0,6315698	CKB	protein_coding
0,6238595	BTRC	protein_coding
0,8997937	BAG5	protein_coding
0,2354584	LARP6	protein_coding
0,9336853	API5	protein_coding
0,9423015	NOLC1	protein_coding
0,692146	ALKBH3	protein_coding
0,6961817	COPS2	protein_coding
0,0920491	GABRB3	protein_coding
0,9792506	SGPL1	protein_coding
0,8930787	FRS2	protein_coding
0,4847278	CCT2	protein_coding
0,7162408	PCBD1	protein_coding
0,9802452	ARIH1	protein_coding
0,7395968	CLMP	protein_coding
0,7915526	COX11	protein_coding
0,817424	STXBP4	protein_coding
0,2104415	CYYR1	protein_coding
0,6999614	CUL5	protein_coding
0,7182975	WBP1L	protein_coding
0,9551108	BORCS7	protein_coding
0,4161706	ANAPC16	protein_coding
0,7211115	SMPD1	protein_coding
0,7558125	APBB1	protein_coding
0,7842247	SYNPO2L	protein_coding
0,7213265	TRIM44	protein_coding
0,645515	TPP1	protein_coding
0,611428	DCHS1	protein_coding
0,7506449	CYB5A	protein_coding
0,8575378	USP54	protein_coding
0,3113413	C11orf74	protein_coding
0,6757338	ATP9B	protein_coding
0,8890235	PPFIBP2	protein_coding
0,9334172	KIAA0355	protein_coding
0,8850351	TUB	protein_coding
0,7395905	RIC3	protein_coding
0,4998506	IDH3A	protein_coding
0,3472571	ZMAT1	protein_coding
0,624851	XRRA1	protein_coding
0,7010516	RNF169	protein_coding
0,8062191	RPL27A	protein_coding
0,8373584	ST5	protein_coding
0,5062136	ATMIN	protein_coding
0,9603194	TMEM41B	protein_coding
0,7968733	PKD1L2	polymorphic_pseudogene
0,8711676	LEO1	protein_coding
0,4750209	ZNF143	protein_coding
0,8189129	TMX3	protein_coding
0,5675917	MFAP4	protein_coding
0,7607016	WEE1	protein_coding
0,9729242	HDGFL3	protein_coding
0,7213265	MCM7	protein_coding
0,8578793	CCDC68	protein_coding
0,868018	ZNF3	protein_coding
0,8373584	RIMKLB	protein_coding
0,2833432	TK2	protein_coding
0,9248086	TMED3	protein_coding

0,7235397	SEC11C	protein_coding
0,7816267	TMEM135	protein_coding
0,9917279	NDEL1	protein_coding
0,903318	RRAD	protein_coding
0,5558351	CIAO2B	protein_coding
0,7842247	HSP90B1	protein_coding
0,583239	BLCAP	protein_coding
0,9087161	ATF7IP2	protein_coding
0,396339	BEX3	protein_coding
0,8519625	COG1	protein_coding
0,8198317	PLEKHA7	protein_coding
0,9759101	ZNF606	protein_coding
0,9358987	B2M	protein_coding
0,7464528	ZNF592	protein_coding
0,3765746	CASC4	protein_coding
0,7744911	NNMT	protein_coding
0,9973317	AP1G1	protein_coding
0,6796756	SLFN5	protein_coding
0,7444016	ZNF667-AS1	lncRNA
0,8003414	BMERB1	protein_coding
0,64053	MARF1	protein_coding
0,8930787	PPIB	protein_coding
0,3089259	CIAO2A	protein_coding
0,9484395	FAM111A	protein_coding
0,9350074	LDHD	protein_coding
0,5735514	PLIN1	protein_coding
0,5565876	TMEM170A	protein_coding
0,5029234	RBPMS2	protein_coding
0,5375314	NAV2	protein_coding
0,9759101	DCTN5	protein_coding
0,0731855	TERF2IP	protein_coding
0,8019639	CLPX	protein_coding
0,391877	NEMP1	protein_coding
0,654521	VPS39	protein_coding
0,7296863	STAT6	protein_coding
0,933129	PATL1	protein_coding
0,7611529	STX3	protein_coding
0,3872426	MRPL16	protein_coding
0,8996732	PIP4K2C	protein_coding
0,6563172	MTMR10	protein_coding
0,8721867	YWHAB	protein_coding
0,9484395	MS4A7	protein_coding
0,997931	DIS3L	protein_coding
0,4638153	CCNDBP1	protein_coding
0,5051303	SMAD3	protein_coding
0,9609096	MAP1A	protein_coding
0,0386353	AKTIP	protein_coding
0,7558125	MAPRE2	protein_coding
0,4165098	EVA1C	protein_coding
0,6591549	MARS	protein_coding
0,9738194	PDIA3	protein_coding
0,8801863	NUDT21	protein_coding
0,855954	SGSM1	protein_coding
0,0385201	TEF	protein_coding
0,9925374	PBX3	protein_coding
0,3653941	PHB	protein_coding
0,3078991	SNRPD1	protein_coding
0,8447416	ACSF2	protein_coding
0,487229	GOLGA2	protein_coding

0,8788037	TRUB2	protein_coding
0,8034097	COQ4	protein_coding
0,7880474	URM1	protein_coding
0,8258095	COQ7	protein_coding
0,928336	GPRC5B	protein_coding
0,6613295	CRK	protein_coding
0,7085673	FBXO22	protein_coding
0,3830886	TBC1D2B	protein_coding
0,9908068	ZNF91	protein_coding
0,8091803	RNF214	protein_coding
0,9694578	CDK12	protein_coding
0,9998538	POP5	protein_coding
0,4806087	ATP5MG	protein_coding
0,9205351	TBC1D16	protein_coding
0,2440238	MYO5B	protein_coding
0,7154026	ACAA2	protein_coding
0,9925374	STIM1	protein_coding
0,8318538	RRM1	protein_coding
0,8969303	IRGQ	protein_coding
0,9528743	ZNF226	protein_coding
0,9804708	ZNF180	protein_coding
0,7984578	PPP2R3B	protein_coding
0,9496064	SMG8	protein_coding
0,7320389	TPM4	protein_coding
0,8930787	GPX4	protein_coding
0,9356153	MIDN	protein_coding
0,9912461	GATAD2A	protein_coding
0,6854132	TRAPPC2L	protein_coding
0,8992483	ANKRD11	protein_coding
0,5474737	RPL13	protein_coding
0,7980625	ZNF641	protein_coding
0,8622295	KMT2D	protein_coding
0,9817008	CORO6	protein_coding
0,7160987	TUBA1A	protein_coding
0,5293665	TUBA1C	protein_coding
0,9450135	ZNF528	protein_coding
0,9933219	ZNF701	protein_coding
0,7854018	SERTAD3	protein_coding
0,6824068	GPD1	protein_coding
0,999806	AXL	protein_coding
0,9693586	LAIR1	protein_coding
0,4894579	LENG8	protein_coding
0,9922688	TRAPPC9	protein_coding
0,809839	ZNF146	protein_coding
0,9441647	ZNF283	protein_coding
0,6776989	DAPK3	protein_coding
0,2841212	EEF2	protein_coding
0,8330359	UBXN6	protein_coding
0,5062136	PLIN4	protein_coding
0,9270004	GLOD4	protein_coding
0,9450135	TSR1	protein_coding
0,3803906	ZNF83	protein_coding
0,8682129	OTUB1	protein_coding
0,5249842	SPRYD3	protein_coding
0,6238595	IGFBP6	protein_coding
0,9998478	ZNF558	protein_coding
0,8823913	NDUFV1	protein_coding
0,7045706	PRDX2	protein_coding
0,7876407	MIS12	protein_coding

0,1872671	MRPL58	protein_coding
0,3803906	ATP5PD	protein_coding
0,878778	SRP68	protein_coding
0,8373584	TMEM68	protein_coding
0,5558351	FAM234A	protein_coding
0,6383874	ECI1	protein_coding
0,9357394	SRRM2	protein_coding
0,5812017	DDB1	protein_coding
0,8888699	FTH1	protein_coding
0,749866	POLR2G	protein_coding
0,9998538	SLC3A2	protein_coding
0,9725101	C2CD3	protein_coding
0,1974243	TRANK1	protein_coding
0,7984578	RPSA	protein_coding
0,8108219	CTNNB1	protein_coding
0,0419898	ULK4	protein_coding
0,271425	LTBP3	protein_coding
0,9628671	SF1	protein_coding
0,2506935	SCARA5	protein_coding
0,5537635	COPS6	protein_coding
0,9069111	PAFAH1B2	protein_coding
0,9366634	KIAA1586	protein_coding
0,3953518	RAB4A	protein_coding
0,9967216	SETD5	protein_coding
0,8373584	THAP9	protein_coding
0,9998478	RNF187	protein_coding
0,3466656	HOOK3	protein_coding
0,9296784	MAPK1IP1L	protein_coding
0,3881918	RBPJ	protein_coding
0,9253623	LMBRD1	protein_coding
0,8578622	ZCCHC4	protein_coding
0,4835164	TTC39C	protein_coding
0,624851	UBTD2	protein_coding
0,6929857	NKIRAS2	protein_coding
0,3881918	DNAJC7	protein_coding
0,3517736	IRF2BP2	protein_coding
0,195587	NT5DC2	protein_coding
0,4160411	SMIM4	protein_coding
0,7823793	BMI1	protein_coding
0,7490465	THAP11	protein_coding
0,5245497	MMADHC	protein_coding
0,3177457	PDHB	protein_coding
0,2945575	PXK	protein_coding
0,9363291	HIST1H1E	protein_coding
0,8992483	PCMTD1	protein_coding
0,8882787	MPLKIP	protein_coding
0,997931	ACOX2	protein_coding
0,1356666	FAM107A	protein_coding
0,7871131	IRF2	protein_coding
0,9233889	XIRP1	protein_coding
0,5011485	ARF4	protein_coding
0,3508112	SEPTIN2	protein_coding
0,3715255	FILIP1L	protein_coding
0,8463447	TAP1	protein_coding
0,8992483	ING5	protein_coding
0,9572126	ATG4B	protein_coding
0,9248086	CMAHP	transcribed_unitary_pseudogene
0,5011485	RFWD3	protein_coding
0,4848639	COG7	protein_coding

0,853092	CDC40	protein_coding
0,3202177	STIP1	protein_coding
0,2945575	RAB31	protein_coding
0,8099847	TNXB	protein_coding
0,8836774	ATXN2L	protein_coding
0,9033043	POLR3D	protein_coding
0,9894339	CAVIN2	protein_coding
0,5011485	HJV	protein_coding
0,696332	FNTA	protein_coding
0,9933219	TRAPPC11	protein_coding
0,2945575	COL3A1	protein_coding
0,8373584	CDKN2AIP	protein_coding
0,8118765	SNRNP48	protein_coding
0,887319	SLC20A2	protein_coding
0,981857	TMUB2	protein_coding
0,5879554	STAT3	protein_coding
0,864196	ADAM9	protein_coding
0,1933793	NDUFS5	protein_coding
0,3881918	LRATD2	protein_coding
0,9954421	LDLRAD4	protein_coding
0,5558351	SLC16A4	protein_coding
0,1501348	LRP1B	protein_coding
0,6613295	AHCYL1	protein_coding
0,1129731	DNAJC21	protein_coding
0,7968733	PKIG	protein_coding
0,4835164	SEMA4C	protein_coding
0,5964345	CNNM3	protein_coding
0,2318378	GSTM4	protein_coding
0,4467509	TET2	protein_coding
0,5879554	PPIP5K1	protein_coding
0,6232451	ZBTB5	protein_coding
0,5660793	ADAL	protein_coding
0,504498	SNTB2	protein_coding
0,7444016	ZNF507	protein_coding
0,8318519	STX18	protein_coding
0,5890453	GFM1	protein_coding
0,4761054	ATOH8	protein_coding
0,9535559	USP39	protein_coding
0,9572126	C2orf68	protein_coding
0,8869707	RNF181	protein_coding
0,7467854	LRRC28	protein_coding
0,7213265	MAT2A	protein_coding
0,7558125	ZNF608	protein_coding
0,6613295	INPP5D	protein_coding
0,9248086	LETM1	protein_coding
0,8232467	TMEM129	protein_coding
0,1821534	PPIC	protein_coding
0,5420087	CEP120	protein_coding
0,1528801	STXBP6	protein_coding
0,2841212	MFF	protein_coding
0,9182782	PXDC1	protein_coding
0,9484395	E2F6	protein_coding
0,5558351	FEM1B	protein_coding
0,9670595	COMMD8	protein_coding
0,2819771	ATP5ME	protein_coding
0,1382998	UQCRFS1	protein_coding
0,933129	COL4A3	protein_coding
0,8623038	MAP2K1	protein_coding
0,9127443	HNRNPH1	protein_coding

0,7087026	IRS1	protein_coding
0,8930787	MECP2	protein_coding
0,5879554	UPF3A	protein_coding
0,8818298	AR	protein_coding
0,4986683	DHRSX	protein_coding
0,9068107	HSPBAP1	protein_coding
0,4775021	SLC25A6	protein_coding
0,7834554	PARM1	protein_coding
0,5675917	CSNK1G1	protein_coding
0,7774855	FAM110B	protein_coding
0,8238032	UBE2V2	protein_coding
0,7712396	ZBTB43	protein_coding
0,9233889	XPO6	protein_coding
0,9533676	MN1	protein_coding
0,5590581	NSMCE1	protein_coding
0,9678404	CCDC126	protein_coding
0,9709611	NPIPB12	protein_coding
0,7321942	CD2BP2	protein_coding
0,9998538	LMAN2	protein_coding
0,3358601	THBS3	protein_coding
0,8976077	CA5B	protein_coding
0,290302	EFNA1	protein_coding
0,9808899	NPIPB3	protein_coding
0,7558125	SH3TC2	protein_coding
0,7834471	NMD3	protein_coding
0,620901	B3GALNT1	protein_coding
0,3398681	HSPB3	protein_coding
0,9233889	KCNAB1	protein_coding
0,3697716	MRPL1	protein_coding
0,7805908	SHE	protein_coding
0,7296863	PGM2	protein_coding
0,8862542	SLC33A1	protein_coding
0,505053	CRADD	protein_coding
0,6734328	SIN3A	protein_coding
0,6613295	ARL13B	protein_coding
0,9148394	PTK2	protein_coding
0,6729908	PTPN9	protein_coding
0,8281575	NPR1	protein_coding
0,140122	SCN9A	protein_coding
0,9082846	SDC2	protein_coding
0,8919058	MMGT1	protein_coding
0,6160654	TM2D2	protein_coding
0,9666028	PLEKHA2	protein_coding
0,4835164	CLIC4	protein_coding
0,8422988	GPR183	protein_coding
0,4933085	CCDC8	protein_coding
0,9082846	METTL15	protein_coding
0,9360637	ZEB2	protein_coding
0,1763931	HINT1	protein_coding
0,9074092	DTWD2	protein_coding
0,2841212	NFU1	protein_coding
0,1712026	ANTXR1	protein_coding
0,9644268	C15orf40	protein_coding
0,615864	RAMAC	protein_coding
0,4806087	APLF	protein_coding
0,9126716	RGPD8	protein_coding
0,6003589	LUZP1	protein_coding
0,9874145	AGPAT2	protein_coding
0,6059512	FASN	protein_coding

0,6613817	CNBP	protein_coding
0,9998478	MT1E	protein_coding
0,9879323	GPS1	protein_coding
0,7296863	DCXR	protein_coding
0,3269911	ZNF32	protein_coding
0,8885864	LDB2	protein_coding
0,7321942	LIMS1	protein_coding
0,7558125	TAPT1	protein_coding
0,4835164	UGP2	protein_coding
0,8930787	HNRNPF	protein_coding
0,6776791	BTD	protein_coding
0,8238032	CSGALNACT2	protein_coding
0,0998266	ROBO1	protein_coding
0,2499691	AVEN	protein_coding
0,9933219	CTNND2	protein_coding
0,6574161	TRIM56	protein_coding
0,060478	REPS2	protein_coding
0,99743	SYAP1	protein_coding
0,3606951	ITGAM	protein_coding
0,645515	TPST1	protein_coding
0,6244656	TOR1AIP2	protein_coding
0,4799252	TM4SF1	protein_coding
0,9670362	OTUD3	protein_coding
0,8500846	GUSB	protein_coding
0,5558351	BRD3	protein_coding
0,9251589	KLF13	protein_coding
0,3607643	ZFPM2	protein_coding
0,6828036	MAP3K2	protein_coding
0,5799813	SF3B5	protein_coding
0,5222808	ZNF35	protein_coding
0,855954	CHD3	protein_coding
0,9729242	ALCAM	protein_coding
0,9975676	YWHAG	protein_coding
0,3847557	UBE2E3	protein_coding
0,8869707	TMEM192	protein_coding
0,6607313	AC106795.1	transcribed_unprocessed_pseudogene
0,9112805	ZNF778	protein_coding
0,8269408	NIPA1	protein_coding
0,730165	UBE2E1	protein_coding
0,949756	HNRNPA3	protein_coding
0,8786805	SIK2	protein_coding
0,7954765	RNF150	protein_coding
0,4818782	USP38	protein_coding
0,7785964	PWWP2A	protein_coding
0,8683086	USP47	protein_coding
0,9928407	PDCD6IP	protein_coding
0,9908528	GLB1	protein_coding
0,2104415	FAXDC2	protein_coding
0,2146641	CRTAP	protein_coding
0,9296784	SLN	protein_coding
0,8324496	ELP5	protein_coding
0,5688668	STX8	protein_coding
0,9951314	UBB	protein_coding
0,489407	NFRKB	protein_coding
0,9976097	FABP4	protein_coding
0,7985809	B3GNT2	protein_coding
0,3358222	FOS	protein_coding
0,9933219	TMED10	protein_coding
0,7531077	SETMAR	protein_coding

0,9998538	SMAD1	protein_coding
0,4260926	SLC30A1	protein_coding
0,9245441	DCLK2	protein_coding
0,7546576	TMEM182	protein_coding
0,9956922	KRT8	protein_coding
0,0727176	METTL7B	protein_coding
0,7968733	HARS	protein_coding
0,0390866	NFXL1	protein_coding
0,9700639	DENND5B	protein_coding
0,5879554	CD14	protein_coding
0,6591127	DNAJC18	protein_coding
0,9998478	RALGAPB	protein_coding
0,4344705	NPAS2	protein_coding
0,6903497	LONRF2	protein_coding
0,6244656	NUDT9	protein_coding
0,6888062	PA2G4	protein_coding
0,9868416	PFKFB3	protein_coding
0,6371005	ARL6IP1	protein_coding
0,4467509	SERPINB9	protein_coding
0,6098976	CDH2	protein_coding
0,7258655	EMB	protein_coding
0,4151093	STAT2	protein_coding
0,9572126	NUDCD2	protein_coding
0,9925374	HSPA4	protein_coding
0,9378903	SGCD	protein_coding
0,8176511	DPY19L2P2	transcribed_unprocessed_pseudogene
0,427478	ARMC10	protein_coding
0,7823793	RNF34	protein_coding
0,8373584	ACYP2	protein_coding
0,8992483	ATF7	protein_coding
0,6088362	RASA4B	protein_coding
0,6217281	SOCS6	protein_coding
0,8455757	CAVIN4	protein_coding
0,9581117	KIF5B	protein_coding
0,9540553	AKAP13	protein_coding
0,5911872	CHCHD7	protein_coding
0,9481776	HTRA3	protein_coding
0,6383874	FOXN2	protein_coding
0,8992483	LMOD2	protein_coding
0,8189129	USP32	protein_coding
0,904174	PPM1D	protein_coding
0,8318664	KBTBD2	protein_coding
0,5175488	RIOX2	protein_coding
0,5485366	LSM3	protein_coding
0,645515	KIAA0232	protein_coding
0,5445815	MTSS1	protein_coding
0,4464427	TMEM43	protein_coding
0,9641192	RNF139	protein_coding
0,6347101	RPS9	protein_coding
0,7740102	GSTA4	protein_coding
0,6160654	MSANTD4	protein_coding
0,7211115	NDUFA3	protein_coding
0,749866	TPT1-AS1	lncRNA
0,6604792	TANC2	protein_coding
0,9528743	DNAJC24	protein_coding
0,7724125	ZNF160	protein_coding
0,6988495	ZNF415	protein_coding
0,2809994	HAS2	protein_coding
0,124191	PDGFD	protein_coding

0,5456715	S1PR1	protein_coding
0,8295224	PYGO1	protein_coding
0,9860501	PKIA	protein_coding
0,5265966	FEZ2	protein_coding
0,8825313	MTM1	protein_coding
0,9210173	INSR	protein_coding
0,4032255	MFN1	protein_coding
0,9277595	GIMAP8	protein_coding
0,9860501	ATP6V0E2	protein_coding
0,5140003	PRKCE	protein_coding
0,6835119	JAGN1	protein_coding
0,9154352	TADA3	protein_coding
0,645515	SOCS5	protein_coding
0,5029234	C1GALT1C1	protein_coding
0,6157478	ZNF672	protein_coding
0,2080886	TMEM126A	protein_coding
0,3697716	TMEM126B	protein_coding
0,922549	TRIM8	protein_coding
0,6835435	JUNB	protein_coding
0,5880659	FAM98B	protein_coding
0,8219774	ZNF439	protein_coding
0,9528743	ZNF440	protein_coding
0,9928407	CANT1	protein_coding
0,2169157	KCNK3	protein_coding
0,4810647	ZDHHC16	protein_coding
0,9625751	CHST11	protein_coding
0,7740102	EXOSC1	protein_coding
0,99108	PGAM1	protein_coding
0,646339	CHD7	protein_coding
0,9074092	CLCN5	protein_coding
0,4954628	KCND3	protein_coding
0,654521	PDE7B	protein_coding
0,6203109	MCC	protein_coding
0,8274546	ZBTB26	protein_coding
0,7713242	DSEL	protein_coding
0,9914435	ASXL1	protein_coding
0,9840915	ZNF562	protein_coding
0,8185595	ZNF318	protein_coding
0,7740102	ZNF561	protein_coding
0,5848767	WIPF2	protein_coding
0,7936863	LRRC8C	protein_coding
0,8189129	RSL1D1	protein_coding
0,654521	LRRC8D	protein_coding
0,6383874	PPID	protein_coding
0,4765298	ETFDH	protein_coding
0,3679769	LPAR3	protein_coding
0,7558125	TBCA	protein_coding
0,9767278	BCL2L1	protein_coding
0,8117718	PLRG1	protein_coding
0,7558125	CLSTN1	protein_coding
0,6662761	CXXC5	protein_coding
0,8875885	ZNF274	protein_coding
0,8062191	SLC25A33	protein_coding
0,9484395	ENC1	protein_coding
0,1067405	SPSB1	protein_coding
0,7558125	BPTF	protein_coding
0,7456366	GPR34	protein_coding
0,6310261	ATF7IP	protein_coding
0,9769756	ANO5	protein_coding

0,8258095	HDAC3	protein_coding
0,8381556	GPHN	protein_coding
0,115935	VAT1L	protein_coding
0,5313262	CAMTA1	protein_coding
0,1712026	GATM	protein_coding
0,9182782	BCL2	protein_coding
0,7915374	CTPS1	protein_coding
0,9781971	ZNF540	protein_coding
0,9998478	EXOSC10	protein_coding
0,7576959	ZNF570	protein_coding
0,8111745	MLLT3	protein_coding
0,624851	TRAPPC12	protein_coding
0,4894579	RPS21	protein_coding
0,6285359	C3AR1	protein_coding
0,9774667	PTEN	protein_coding
0,997931	RPS7	protein_coding
0,7545035	RNASEH1	protein_coding
0,654521	PRNP	protein_coding
0,8466046	FRMD5	protein_coding
0,9074092	TLN2	protein_coding
0,9248086	TVP23B	protein_coding
0,8087971	ZNF217	protein_coding
0,6199446	SRGAP2C	protein_coding
0,934719	JMJD1C	protein_coding
0,9700303	SYNPO	protein_coding
0,6785825	RAB33B	protein_coding
0,7385573	ANKRD20A4	protein_coding
0,5355129	LAMB2	protein_coding
0,6254273	USP19	protein_coding
0,6903585	QARS	protein_coding
0,2073391	ORMDL3	protein_coding
0,5166442	SERF1A	protein_coding
0,3316892	KLF11	protein_coding
0,4999985	SMN1	protein_coding
0,6867028	EIF2AK3	protein_coding
0,7518452	KRCC1	protein_coding
0,6867028	NME6	protein_coding
0,3792977	CYCS	protein_coding
0,7913684	FRMD3	protein_coding
0,7885463	SNTB1	protein_coding
0,2945575	MRPL13	protein_coding
0,8912998	MALT1	protein_coding
0,9296784	MBOAT1	protein_coding
0,0088417	ID4	protein_coding
0,9839612	GPR22	protein_coding
0,6543081	CEBPB	protein_coding
0,7355144	PAIP1	protein_coding
0,7253153	NEGR1	protein_coding
0,9670362	ZNF131	protein_coding
0,9709611	BSG	protein_coding
0,6762366	CERS6	protein_coding
0,9450135	SPTLC3	protein_coding
0,7834471	COPRS	protein_coding
0,8330596	TP53RK	protein_coding
0,4362639	BPGM	protein_coding
0,8545769	SUCLG2	protein_coding
0,5402971	CSDC2	protein_coding
0,2073391	RCAN2	protein_coding
0,4466289	IL16	protein_coding

0,9767278	GNB2	protein_coding
0,4740961	GNG12	protein_coding
0,4246004	MYOZ2	protein_coding
0,3792977	SYNPO2	protein_coding
0,633922	COPS9	protein_coding
0,9914435	GTPBP2	protein_coding
0,7444016	FGGY	protein_coding
0,4957019	TCEAL1	protein_coding
0,8545769	ZNF24	protein_coding
0,8427176	MANEA	protein_coding
0,7542355	AFF1	protein_coding
0,992369	FIBP	protein_coding
0,9828271	PPP1CA	protein_coding
0,9374881	HCFC1	protein_coding
0,7321942	PDE3A	protein_coding
0,9618453	RASGRP1	protein_coding
0,1667775	CHCHD1	protein_coding
0,7187791	MRPL52	protein_coding
0,5166442	EFEMP2	protein_coding
0,5879554	WASHC2C	protein_coding
0,5180247	ZMAT3	protein_coding
0,6425583	ZFAND4	protein_coding
0,872444	SLFN11	protein_coding
0,3451145	FUT10	protein_coding
0,4409678	LRRC20	protein_coding
0,8875885	CFL1	protein_coding
0,8330359	TMCC1	protein_coding
0,9781971	NAA16	protein_coding
0,445647	FAM192A	protein_coding
0,3724099	CBWD1	protein_coding
0,7419278	DCP2	protein_coding
0,8062191	RPL38	protein_coding
0,5245497	CYP7B1	protein_coding
0,4498511	CES2	protein_coding
0,9315593	SP3	protein_coding
0,902171	DMXL1	protein_coding
0,7912354	ZNF621	protein_coding
0,8131645	NADSYN1	protein_coding
0,9540553	DHCR7	protein_coding
0,8721867	NBEA	protein_coding
0,7639764	OXSR1	protein_coding
0,6607313	PHF8	protein_coding
0,7774855	LCLAT1	protein_coding
0,3030424	MIR4435-2HG	lncRNA
0,2337466	AC007318.1	processed_pseudogene
0,2908312	GXYLT2	protein_coding
0,5510556	DCAKD	protein_coding
0,855954	TADA2B	protein_coding
0,9481776	GRK2	protein_coding
0,9771336	RELA	protein_coding
0,784306	ZNF680	protein_coding
0,9081173	HECTD4	protein_coding
0,9112805	BNC2	protein_coding
0,4663698	COQ2	protein_coding
0,5140003	TRMT112	protein_coding
0,0385201	LRRN3	protein_coding
0,8369686	KDM2A	protein_coding
0,5245497	MRPL57	protein_coding
0,9491167	NOC3L	protein_coding

0,6906735	ESRRA	protein_coding
0,6285359	COMMD1	protein_coding
0,9998478	RAPH1	protein_coding
0,8917504	ADCY5	protein_coding
0,9894339	PARP14	protein_coding
0,9484395	CKS1B	protein_coding
0,8522628	ABCD2	protein_coding
0,7024923	AHSA2P	transcribed_unitary_pseudogene
0,8823913	ABLIM3	protein_coding
0,9998478	MFSD4B	protein_coding
0,7247675	VANGL1	protein_coding
0,3893986	GLRX	protein_coding
0,6128477	IQCB1	protein_coding
0,8745367	GOLGB1	protein_coding
0,284683	ZNF483	protein_coding
0,8221205	MMRN2	protein_coding
0,7385573	TNKS	protein_coding
0,3803906	ZNF449	protein_coding
0,9290837	ZBTB21	protein_coding
0,4513092	PPP1R3B	protein_coding
0,073095	STOX2	protein_coding
0,9410184	MAP3K11	protein_coding
0,654521	TRIB1	protein_coding
0,7972313	C1QB	protein_coding
0,933129	C1QA	protein_coding
0,943695	DAG1	protein_coding
0,4514702	ARV1	protein_coding
0,9270127	NAA20	protein_coding
0,0379118	MICOS10	protein_coding
0,633922	EHBP1L1	protein_coding
0,7378932	THAP2	protein_coding
0,5313262	PPP1R14B	protein_coding
0,330331	SMARCC1	protein_coding
0,5768067	ZNF417	protein_coding
0,9933219	PTPRM	protein_coding
0,9544158	VEGFB	protein_coding
0,5365997	PEAK1	protein_coding
0,9461357	MOB1B	protein_coding
0,8326738	ZNF622	protein_coding
0,9238011	CSPG4	protein_coding
0,3004311	SNX33	protein_coding
0,8823093	NABP1	protein_coding
0,7135515	CHD2	protein_coding
0,4148453	CEP83	protein_coding
0,6281588	NUDT4	protein_coding
0,4165098	SCAI	protein_coding
0,784306	NMNAT1	protein_coding
0,7740102	HSPB7	protein_coding
0,2179973	UQCRH	protein_coding
0,9953531	EIF1AX	protein_coding
0,8823913	BCLAF3	protein_coding
0,5510619	PSMD1	protein_coding
0,7743102	HEG1	protein_coding
0,633922	TOMM20	protein_coding
0,4835164	AGFG1	protein_coding
0,5073762	STAT5B	protein_coding
0,9112805	CNP	protein_coding
0,7820981	JUP	protein_coding
0,9820421	EIF1	protein_coding

0,8255851	RNF213	protein_coding
0,8941532	NET1	protein_coding
0,7125689	DPY19L1	protein_coding
0,7444016	ZNF791	protein_coding
0,7431559	PHC3	protein_coding
0,6736287	GOLIM4	protein_coding
0,8189129	RBM4B	protein_coding
0,3271904	ATP5MD	protein_coding
0,673546	C1QTNF1	protein_coding
0,9416952	MARCH3	protein_coding
0,4292102	RBM4	protein_coding
0,7433064	UBXN2A	protein_coding
0,4806087	TCAP	protein_coding
0,8221205	KLHL15	protein_coding
0,6254273	FBXO45	protein_coding
0,9700303	SLC25A30	protein_coding
0,633922	CD34	protein_coding
0,4464427	CTSF	protein_coding
0,620901	MSRB3	protein_coding
0,8875885	LEMD3	protein_coding
0,7558125	TLR1	protein_coding
0,9998478	FAM174A	protein_coding
0,4222683	TRMT10C	protein_coding
0,2150608	SELP	protein_coding
0,8373584	MGA	protein_coding
0,8805902	PIGG	protein_coding
0,8373584	PRPF8	protein_coding
0,2073391	ADCY6	protein_coding
0,9802452	PITPNA	protein_coding
0,7671445	DDX23	protein_coding
0,5801848	ZBTB4	protein_coding
0,9481776	ZHX3	protein_coding
0,7242009	PODN	protein_coding
0,9944531	STAG3L3	transcribed_unprocessed_pseudogene
0,997931	RALGAPA1	protein_coding
0,6988495	LIG4	protein_coding
0,9953531	GTF2IRD2B	protein_coding
0,5265966	ABRA	protein_coding
0,1888441	ATP2A2	protein_coding
0,8289129	RPL4	protein_coding
0,4697514	SNAPC5	protein_coding
0,9115427	DENND4A	protein_coding
0,9484395	ANKRD36C	protein_coding
0,8161522	SLC26A9	protein_coding
0,4894579	MRPL11	protein_coding
0,5817753	AKIRIN1	protein_coding
0,736331	MSL2	protein_coding
0,6285359	CMKLR1	protein_coding
0,998169	ANGEL2	protein_coding
0,5060008	SLCO2A1	protein_coding
0,7558125	ZNF266	protein_coding
0,7945147	B4GAT1	protein_coding
0,9233889	TMEM167A	protein_coding
0,2778507	SH3PXD2B	protein_coding
0,8908494	RESF1	protein_coding
0,5408562	LARP7	protein_coding
0,5245497	NR1D2	protein_coding
0,4684923	RPL15	protein_coding
0,9702125	FAM241A	protein_coding

0,8500846	SRP72	protein_coding
0,9142106	THAP6	protein_coding
0,5869808	FZD4	protein_coding
0,9408651	DENND6A	protein_coding
0,9840915	PDE12	protein_coding
0,7611529	GLMN	protein_coding
0,0413597	RSRC1	protein_coding
0,9985172	RAB1B	protein_coding
0,6658884	MICOS13	protein_coding
0,9953531	DHX36	protein_coding
0,8373546	AC026271.1	processed_pseudogene
0,6168923	CTBP2	protein_coding
0,4740961	ZDHHC14	protein_coding
0,6475905	ATR	protein_coding
0,1970133	SNHG29	lncRNA
0,7854018	GK5	protein_coding
0,9975676	VCPIP1	protein_coding
0,8575378	DES	protein_coding
0,8792761	PDIK1L	protein_coding
0,9589397	TRAF6	protein_coding
0,9705617	ZNF654	protein_coding
0,6543081	TVP23C	protein_coding
0,2841212	MRPS22	protein_coding
0,4834958	PACS1	protein_coding
0,8474324	SH3BP5L	protein_coding
0,4409678	YPEL2	protein_coding
0,366478	CADM2	protein_coding
0,8148845	PSMD2	protein_coding
0,3218826	CSRP2	protein_coding
0,8023631	DDIT3	protein_coding
0,6591549	PCCA	protein_coding
0,8829558	DCTN2	protein_coding
0,6000916	NPPA	protein_coding
0,7395639	CTDSP2	protein_coding
0,8578793	CKAP5	protein_coding
0,2568349	ARHGAP1	protein_coding
0,5511615	ATG13	protein_coding
0,9484395	GOLGA8A	protein_coding
0,9840915	TP53I11	protein_coding
0,7201269	PHYKPL	protein_coding
0,9738194	ZNF519	protein_coding
0,554581	LSM1	protein_coding
0,6190365	BANF1	protein_coding
0,624851	TMEM9B	protein_coding
0,7444016	PTPN2	protein_coding
0,8769229	SMAD2	protein_coding
0,4405523	EIF3F	protein_coding
0,4673249	ZNF25	protein_coding
0,9496064	ARL10	protein_coding
0,6790658	CLTB	protein_coding
0,7767291	LPL	protein_coding
0,8698319	CCDC14	protein_coding
0,9761718	SART1	protein_coding
0,7934256	PPP2R2D	protein_coding
0,328725	MCTP1	protein_coding
0,9039795	ALG10B	protein_coding
0,9182782	PAAF1	protein_coding
0,2492279	MRPL48	protein_coding
0,9998478	RAB6A	protein_coding

0,9998478	ERCC4	protein_coding
0,2329611	TMEM70	protein_coding
0,6613295	TOM1L2	protein_coding
0,4089968	MTLN	protein_coding
0,8578622	MLXIP	protein_coding
0,5664861	AURKAIP1	protein_coding
0,7956173	SLC35E3	protein_coding
0,284683	RUVBL1	protein_coding
0,1712026	MSRA	protein_coding
0,7583175	CTDNEP1	protein_coding
0,3578629	RPL7AP66	processed_pseudogene
0,5998801	ZDHHC21	protein_coding
0,7154026	PLEKHF2	protein_coding
0,5296182	A2M	protein_coding
0,8844081	UBE2O	protein_coding
0,8861906	KLHL38	protein_coding
0,623981	UNC119B	protein_coding
0,9628671	DENND2C	protein_coding
0,1444307	TUBB6	protein_coding
0,9127443	LYSMD3	protein_coding
0,9481776	NUPR1	protein_coding
0,7918194	SLC35A4	protein_coding
0,6425736	IP6K1	protein_coding
0,9481776	CSTF3	protein_coding
0,8155634	YES1	protein_coding
0,676881	DLEU1	lncRNA
0,8992483	TMEM39A	protein_coding
0,9358535	TCP11L1	protein_coding
0,7102188	BNIP3	protein_coding
0,8422988	SMIM19	protein_coding
0,887319	RTTN	protein_coding
0,440767	ZBTB8OS	protein_coding
0,2809994	COX8A	protein_coding
0,7213265	CDC26	protein_coding
0,922549	CRLF3	protein_coding
0,7611529	RNPEP	protein_coding
0,3137083	KCMF1	protein_coding
0,7954765	SPRYD4	protein_coding
0,4740961	CLEC14A	protein_coding
0,9808899	SYNE3	protein_coding
0,9709611	CLK2	protein_coding
0,3578629	SLCO3A1	protein_coding
0,6867028	PLAAT3	protein_coding
0,5343413	DIRAS1	protein_coding
0,8019639	GNG7	protein_coding
0,9761718	USF3	protein_coding
0,5754389	RMDN1	protein_coding
0,7026925	MEX3C	protein_coding
0,4252265	RNF152	protein_coding
0,0908653	MYO1D	protein_coding
0,54642	LRRC37A	protein_coding
0,8786805	TTTY14	lncRNA
0,3678372	RBIS	protein_coding
0,8397654	NCKAP5	protein_coding
0,9112805	RUFY1	protein_coding
0,4840962	LRRC37A3	protein_coding
0,8189129	FKBP9P1	transcribed_unprocessed_pseudogene
0,8373584	VSIG10	protein_coding
0,9781971	FAM91A1	protein_coding

0,6903585	WSB2	protein_coding
0,5318944	PNMA1	protein_coding
0,8373584	TCIM	protein_coding
0,8105839	ANKLE2	protein_coding
0,6219816	THAP4	protein_coding
0,8674075	NFATC2IP	protein_coding
0,2797024	FIBIN	protein_coding
0,8831297	DPP7	protein_coding
0,8422988	SEC24C	protein_coding
0,855954	SMCR8	protein_coding
0,4532937	MTHFR	protein_coding
0,9510059	MTX3	protein_coding
0,3680294	ZDHHC13	protein_coding
0,8721867	SLC38A9	protein_coding
0,8099847	POLE	protein_coding
0,702199	SCN4B	protein_coding
0,7258655	ANO6	protein_coding
0,4919121	ZBTB34	protein_coding
0,9233889	NUDT4B	protein_coding
0,4673249	FAM210A	protein_coding
0,7558125	TALDO1	protein_coding
0,481646	RIMKLA	protein_coding
0,8698319	RPS6KA3	protein_coding
0,9994264	CHD9	protein_coding
0,6602321	GATD1	protein_coding
0,3578629	MAN1B1	protein_coding
0,7467854	TOP3A	protein_coding
0,9540553	ZBTB38	protein_coding
0,5245497	C10orf71	protein_coding
0,7235397	TIMM22	protein_coding
0,9912461	MAGEF1	protein_coding
0,689194	SAMD9L	protein_coding
0,692146	PAWR	protein_coding
0,4835164	TGIF1	protein_coding
0,8258095	NR2C2	protein_coding
0,5140003	CAVIN1	protein_coding
0,54953	ARIH2	protein_coding
0,9215449	ZBTB33	protein_coding
0,9768456	ATOX1	protein_coding
0,9154815	TBL1XR1	protein_coding
0,5336185	SAMD12	protein_coding
0,0390866	CD163	protein_coding
0,8373584	RPLP2	protein_coding
0,5123161	JUN	protein_coding
0,9800121	CSTF2T	protein_coding
0,9253623	GBA	protein_coding
0,9829583	ACAD9	protein_coding
0,4599964	IL17RA	protein_coding
0,9725101	PNPLA2	protein_coding
0,9248086	THAP5	protein_coding
0,3943278	NAALADL2	protein_coding
0,9738181	CD151	protein_coding
0,7296863	POLR2L	protein_coding
0,6160654	NECTIN3	protein_coding
0,6721745	TMEM94	protein_coding
0,8743212	FLII	protein_coding
0,7197887	HNRNPA0	protein_coding
0,7954765	CHID1	protein_coding
0,8885864	ZNF518A	protein_coding

0,7230802	AP3S1	protein_coding
0,645515	GRB2	protein_coding
0,9917279	ZBTB41	protein_coding
0,4835164	UBE2N	protein_coding
0,9680323	ZNF354C	protein_coding
0,5984498	BET1L	protein_coding
0,4726129	RPS27	protein_coding
0,5267449	RIC8A	protein_coding
0,8387664	IMP3	protein_coding
0,1900988	ASB8	protein_coding
0,9840915	DPY19L2	protein_coding
0,8745367	DMAP1	protein_coding
0,6662301	CALHM5	protein_coding
0,6776989	IMPDH2	protein_coding
0,9450135	ALS2CL	protein_coding
0,2407669	MLF1	protein_coding
0,9033043	NDUFAF3	protein_coding
0,7842247	C2orf69	protein_coding
0,887319	PDE4DIP	protein_coding
0,7558125	DDX10	protein_coding
0,9450135	GOLGA8Q	protein_coding
0,5567171	ZNF518B	protein_coding
0,8322139	LCORL	protein_coding
0,6229326	SH2B1	protein_coding
0,7763012	POGLUT3	protein_coding
0,62553	PLEC	protein_coding
0,8301309	ZNF543	protein_coding
0,8682799	GALNT11	protein_coding
0,645515	WDR6	protein_coding
0,4151093	GEN1	protein_coding
0,9356153	ZNF354B	protein_coding
0,5879554	PLEKHM3	protein_coding
0,659093	NT5DC1	protein_coding
0,6604792	RPS3AP5	processed_pseudogene
0,1131434	COX14	protein_coding
0,9450135	H3F3AP6	processed_pseudogene
0,4740961	RPL10P16	processed_pseudogene
0,4151093	P4HTM	protein_coding
0,9282793	DTX3	protein_coding
0,4341808	KLHL11	protein_coding
0,5511615	SLC25A20	protein_coding
0,7550915	CA8	protein_coding
0,993752	EPM2AIP1	protein_coding
0,9725101	ERBB4	protein_coding
0,3558016	MAF	protein_coding
0,8847715	CTNNBIP1	protein_coding
0,3150284	ERN1	protein_coding
0,7007521	CSRNP3	protein_coding
0,9768456	SUZ12	protein_coding
0,9067454	NSUN3	protein_coding
0,9854982	KCTD12	protein_coding
0,9312186	GRINA	protein_coding
0,5400662	THBD	protein_coding
0,2164941	COX5A	protein_coding
0,8791066	FAM219B	protein_coding
0,9481776	ZHX2	protein_coding
0,435547	MPI	protein_coding
0,8086025	EFCAB13	protein_coding
0,9802452	APOLD1	protein_coding

0,6230544	DPY19L3	protein_coding
0,8877092	TAF7	protein_coding
0,6160654	CYBC1	protein_coding
0,7673893	GAK	protein_coding
0,6999614	TUFM	protein_coding
0,9428271	CTC1	protein_coding
0,7431559	FBXO34	protein_coding
0,1767146	SELENOW	protein_coding
0,4422012	EIF3K	protein_coding
0,9385376	MRFAP1L1	protein_coding
0,4011774	SNX18	protein_coding
0,8267398	MRFAP1	protein_coding
0,9729242	C3orf38	protein_coding
0,645515	RCC2	protein_coding
0,5117223	CYC1	protein_coding
0,437327	PER1	protein_coding
0,5347705	TMTC2	protein_coding
0,5869872	SPTY2D1	protein_coding
0,73747	SAMD4B	protein_coding
0,3194617	GIMAP7	protein_coding
0,8255851	TCAIM	protein_coding
0,5073762	FUCA1	protein_coding
0,567026	ZNF664	protein_coding
0,9248086	CALR	protein_coding
0,3881918	MAGED1	protein_coding
0,7444016	RAD23A	protein_coding
0,7213265	PTPN11	protein_coding
0,5029234	FAM156B	protein_coding
0,887319	CLK3	protein_coding
0,8373584	PACS2	protein_coding
0,9804708	ELMOD2	protein_coding
0,59271	LINC00174	lncRNA
0,7558125	GEMIN4	protein_coding
0,8019639	KLHL28	protein_coding
0,8784701	ZBTB18	protein_coding
0,9678404	C14orf28	protein_coding
0,9081173	DNHD1	protein_coding
0,4835164	GCC1	protein_coding
0,6383874	CIITA	protein_coding
0,3830653	CDC42EP4	protein_coding
0,9572126	LACC1	protein_coding
0,7094756	MAF1	protein_coding
0,8528913	CDH5	protein_coding
0,4019188	FAM216B	protein_coding
0,9907221	PCBP1-AS1	lncRNA
0,933129	MYADM	protein_coding
0,4598598	SERTAD2	protein_coding
0,8592084	PDXDC1	protein_coding
0,887128	ZNF154	protein_coding
0,7972653	R3HDM2	protein_coding
0,2061005	ITLN1	protein_coding
0,3327196	NRXN1	protein_coding
0,284683	SEPHS2	protein_coding
0,3803906	C14orf119	protein_coding
0,997931	BBS10	protein_coding
0,9336853	PUF60	protein_coding
0,7235397	AC140134.1	unprocessed_pseudogene
0,6762366	TSHZ1	protein_coding
0,9941501	SOCS4	protein_coding

0,252949	ZADH2	protein_coding
0,8189129	TRNAU1AP	protein_coding
0,9994264	EXOC3	protein_coding
0,9406097	LYNX1	protein_coding
0,7500896	MED14	protein_coding
0,2395631	FAHD1	protein_coding
0,7154026	PRKRA	protein_coding
0,4498511	HERC2P3	transcribed_unprocessed_pseudogene
0,8274546	FGD6	protein_coding
0,6254273	OAZ2	protein_coding
0,4987955	CCDC43	protein_coding
0,6785825	HCLS1	protein_coding
0,0283162	MTURN	protein_coding
0,8868311	ZNF609	protein_coding
0,9538485	PAK2	protein_coding
0,9621537	CCDC66	protein_coding
0,6762366	MCFD2	protein_coding
0,2354584	GAS1	protein_coding
0,8992483	MIGA1	protein_coding
0,9738194	NRIP1	protein_coding
0,8033177	TSPYL5	protein_coding
0,4806087	HIST1H2AC	protein_coding
0,5675917	SRP9P1	processed_pseudogene
0,9358487	ZNF594	protein_coding
0,9163227	PCGF5	protein_coding
0,7318327	YOD1	protein_coding
0,8117718	TMEM64	protein_coding
0,7296863	SMG1P3	transcribed_unprocessed_pseudogene
0,8992483	GPR157	protein_coding
0,1517576	SLC36A4	protein_coding
0,9951314	ZDHHC20	protein_coding
0,8921625	ZFP3	protein_coding
0,2733926	PPA1	protein_coding
0,9336246	PDIA3P1	transcribed_processed_pseudogene
0,5453247	SSR4	protein_coding
0,504498	CAPS2	protein_coding
0,8019639	CUEDC1	protein_coding
0,933129	KCTD2	protein_coding
0,9248086	CMTR2	protein_coding
0,8898615	PITPNB	protein_coding
0,6928629	TCEAL8	protein_coding
0,7952381	LRRC57	protein_coding
0,3195926	MRPL14	protein_coding
0,9808899	NQO1	protein_coding
0,3680294	HIGD1A	protein_coding
0,692146	CHRM2	protein_coding
0,9899185	EHMT1	protein_coding
0,8078279	ADIPOQ	protein_coding
0,0197802	F2R	protein_coding
0,9248086	NPM1	protein_coding
0,5154843	PJA1	protein_coding
0,6835435	DHTKD1	protein_coding
0,5219726	POLR2A	protein_coding
0,9680053	TLCD5	protein_coding
0,9738194	ZNF322	protein_coding
0,8221205	DDX60L	protein_coding
0,9917279	ZNF678	protein_coding
0,7972313	TMEM45A	protein_coding
0,669182	RAP2B	protein_coding

0,9773277	RPL24P4	processed_pseudogene
0,633922	SETD2	protein_coding
0,3640941	MRPS23	protein_coding
0,9169269	GPR135	protein_coding
0,9728506	ZNF875	protein_coding
0,692146	PLAG1	protein_coding
0,7924243	YIPF6	protein_coding
0,8532945	ZBTB20	protein_coding
0,7062909	DIPK2A	protein_coding
0,9954421	AMIGO1	protein_coding
0,993752	SIAH2	protein_coding
0,2945575	COPG1	protein_coding
0,928336	SLC9A9	protein_coding
0,9357394	RELL1	protein_coding
0,3488985	RFX7	protein_coding
0,8847411	RNF41	protein_coding
0,4246004	SLC2A4	protein_coding
0,7639764	IBA57	protein_coding
0,9792506	ZNF329	protein_coding
0,9536474	C5orf24	protein_coding
0,5176282	ADO	protein_coding
0,4475789	COA4	protein_coding
0,5879554	PRKAG1	protein_coding
0,7894641	CCDC149	protein_coding
0,4164572	MRPS11	protein_coding
0,673546	SNRPE	protein_coding
0,1284788	PNMA8A	protein_coding
0,8500846	CHST15	protein_coding
0,1100299	IDH2	protein_coding
0,5416343	GET1	protein_coding
0,4810647	TNRC18	protein_coding
0,2948143	NOP10	protein_coding
0,6232447	ZNF708	protein_coding
0,8992483	IST1	protein_coding
0,9406097	ERCC6L2	protein_coding
0,6591549	MRPL41	protein_coding
0,979961	CREB3L2	protein_coding
0,7258405	UNC5C	protein_coding
0,1067405	UBA7	protein_coding
0,284683	MRPS16	protein_coding
0,0868691	EXT1	protein_coding
0,9156816	SHMT2	protein_coding
0,7025012	ATP6AP2	protein_coding
0,6604792	BACE2	protein_coding
0,7748443	UBE2E2	protein_coding
0,3150284	SYNM	protein_coding
0,9463209	FIGN	protein_coding
0,3881918	AP1S2	protein_coding
0,7081012	C8orf33	protein_coding
0,9142106	C1S	protein_coding
0,8153751	KBTBD3	protein_coding
0,9707919	CLN8	protein_coding
0,9625751	NPLOC4	protein_coding
0,6590532	TSHZ2	protein_coding
0,9374738	EXOC7	protein_coding
0,1428674	KPNA2	protein_coding
0,1773766	BGN	protein_coding
0,9977601	CEP97	protein_coding
0,2945575	GLRX5	protein_coding

0,7187791	MXRA7	protein_coding
0,5956025	LIMK2	protein_coding
0,997931	ADI1	protein_coding
0,7238799	RWDD4	protein_coding
0,8318664	SATB1	protein_coding
0,4684539	CSF1R	protein_coding
0,9233889	TRAK1	protein_coding
0,5600133	PLCB1	protein_coding
0,7546576	SKA2	protein_coding
0,6084958	NDN	protein_coding
0,2850896	TTC3	protein_coding
0,1174245	IGIP	protein_coding
0,9296784	TSKU	protein_coding
0,8670918	ANXA2	protein_coding
0,077831	RGS6	protein_coding
0,9463928	PAPPA	protein_coding
0,578003	NGRN	protein_coding
0,8062191	CRIP2	protein_coding
0,6621874	ACBD3	protein_coding
0,8423712	C16orf72	protein_coding
0,5594718	PLCXD3	protein_coding
0,3451145	COL18A1	protein_coding
0,8555218	RBM10	protein_coding
0,6543081	RPL35A	protein_coding
0,8373584	ZNF721	protein_coding
0,9975676	C11orf54	protein_coding
0,271425	CEP63	protein_coding
0,9284741	SRPRA	protein_coding
0,9496064	EWSR1	protein_coding
0,8387664	HMGN4	protein_coding
0,6383874	SPATA13	protein_coding
0,8786805	GJC1	protein_coding
0,8255851	CNOT10	protein_coding
0,9439974	ZNF662	protein_coding
0,8682799	CADM1	protein_coding
0,5866982	NAA38	protein_coding
0,7352523	AP2A2	protein_coding
0,9496064	SLC8A1	protein_coding
0,1934594	ABAT	protein_coding
0,7940001	CAMK1D	protein_coding
0,9127443	RGPD6	protein_coding
0,5964871	LYSMD4	protein_coding
0,9424246	GAS6	protein_coding
0,6659037	NEB	protein_coding
0,5400662	GPC6	protein_coding
0,8721867	ARHGEF37	protein_coding
0,7940988	CEP57L1	protein_coding
0,6613295	SMDT1	protein_coding
0,621558	HSP90AB3P	processed_pseudogene
0,9112805	RUVBL2	protein_coding
0,947809	CTNNA3	protein_coding
0,7816527	PTTG1IP	protein_coding
0,998169	DDX41	protein_coding
0,767127	DAZAP2	protein_coding
0,8381234	SELENOF	protein_coding
0,860343	RPSAP19	processed_pseudogene
0,4835164	ZNF623	protein_coding
0,692146	BCOR	protein_coding
0,6395564	JRKL	protein_coding

0,3801685	KIAA2026	protein_coding
0,8500846	NPIPA1	protein_coding
0,9410289	SF3A3	protein_coding
0,195587	GRIN2A	protein_coding
0,9731251	GTF2H2C	protein_coding
0,7820981	ASB7	protein_coding
0,9210173	MX2	protein_coding
0,9253623	EP400	protein_coding
0,9925374	PI4KAP2	transcribed_unitary_pseudogene
0,4999985	TENT5C	protein_coding
0,9734904	COA5	protein_coding
0,115935	UTP11	protein_coding
0,7576959	PSMG1	protein_coding
0,6613295	PRR14L	protein_coding
0,9377639	SETD3	protein_coding
0,8163405	FBXL7	protein_coding
0,7302504	TANGO2	protein_coding
0,7611529	SMG1P5	transcribed_unprocessed_pseudogene
0,290432	SFXN4	protein_coding
0,1800952	ZNF438	protein_coding
0,3049645	HMCES	protein_coding
0,1560841	NDUFB1	protein_coding
0,7190863	GUSBP1	transcribed_unprocessed_pseudogene
0,7743102	RFLNB	protein_coding
0,9860501	UPP1	protein_coding
0,9865636	TRIM52	protein_coding
0,8330359	LHFPL6	protein_coding
0,450002	CMTM4	protein_coding
0,6219816	TMEM50A	protein_coding
0,6408377	TBK1	protein_coding
0,7500896	CBX6	protein_coding
0,9406097	KREMEN1	protein_coding
0,9533676	NPIPA5	protein_coding
0,3792977	OLFML1	protein_coding
0,9766993	RBM12B	protein_coding
0,4919121	LIN9	protein_coding
0,7805908	BTBD9	protein_coding
0,6184746	KIRREL1	protein_coding
0,50493	TOB2	protein_coding
0,6317974	SCN5A	protein_coding
0,9427242	UTY	protein_coding
0,9700639	AC138969.1	protein_coding
0,9484395	KMT5A	protein_coding
0,6808534	SMTN	protein_coding
0,4521055	COA3	protein_coding
0,4894579	PTP4A2	protein_coding
0,7956408	ACTG1	protein_coding
0,88341	DENND5A	protein_coding
0,3030424	UQCR10	protein_coding
0,7095541	FAM120C	protein_coding
0,7154026	EIF3C	protein_coding
0,5486193	NIPSNAP1	protein_coding
0,9734904	SCFD2	protein_coding
0,1140347	UBE2F	protein_coding
0,7987424	PPP1R2	protein_coding
0,5985056	TSPYL2	protein_coding
0,9998538	GOLGA6L4	protein_coding
0,4990941	C22orf46	transcribed_unitary_pseudogene
0,8210379	IRAK1	protein_coding

0,2329611	CMSS1	protein_coding
0,4314663	PCDH9	protein_coding
0,6762366	ACOT1	protein_coding
0,9248086	OAF	protein_coding
0,8698319	HIST2H2AC	protein_coding
0,6999614	TM2D3	protein_coding
0,4070535	PRKD1	protein_coding
0,8148845	CCSER1	protein_coding
0,9484259	RPL23AP82	transcribed_unprocessed_pseudogene
0,2250724	SLIT3	protein_coding
0,4724079	EFNA5	protein_coding
0,8847715	CSF1	protein_coding
0,5895248	MAML2	protein_coding
0,9678404	SS18L1	protein_coding
0,998169	COPB2	protein_coding
0,8991816	KNTC1	protein_coding
0,7137584	WDR27	protein_coding
0,8623405	FOXO4	protein_coding
0,4635653	PTP4A3	protein_coding
0,0015119	PROS1	protein_coding
0,8545769	ZFP1	protein_coding
0,6604792	DHRS7C	protein_coding
0,5688668	SOCS3	protein_coding
0,9879323	XPOT	protein_coding
0,9253623	TMEM173	protein_coding
0,9296784	PDE4B	protein_coding
0,8751323	C14orf180	protein_coding
0,4344504	SNN	protein_coding
0,5318944	MED12	protein_coding
0,6442182	SEPTIN9	protein_coding
0,9494054	ZBTB40	protein_coding
0,993752	HIST2H2BE	protein_coding
0,887319	EIF4ENIF1	protein_coding
0,9182782	RNLS	protein_coding
0,7058095	ATL3	protein_coding
0,0830387	NDUFA12	protein_coding
0,6462207	UBE2G2	protein_coding
0,0895372	APOO	protein_coding
0,9572126	TMED9	protein_coding
0,9894339	RBM33	protein_coding
0,1326966	ARMCX2	protein_coding
0,7842706	BTBD6	protein_coding
0,9215449	SUMO3	protein_coding
0,9166492	IMMP2L	protein_coding
0,4430845	ZFP90	protein_coding
0,1763931	NDUFA6	protein_coding
0,654521	AP3M1	protein_coding
0,2329611	F8	protein_coding
0,781311	CA13	protein_coding
0,9535559	CIB1	protein_coding
0,2318378	FLRT2	protein_coding
0,9738194	RPS27L	protein_coding
0,3691605	ADSSL1	protein_coding
0,6787299	FAF1	protein_coding
0,9540553	NSMCE3	protein_coding
0,9925196	HSF1	protein_coding
0,9533676	C6orf120	protein_coding
0,5647201	PURA	protein_coding
0,841477	NOMO2	protein_coding

0,7444016	IFITM2	protein_coding
0,8440006	TNFAIP2	protein_coding
0,8930787	ZNF445	protein_coding
0,9700303	TCEAL9	protein_coding
0,9792506	PRMT3	protein_coding
0,7039013	PRPF39	protein_coding
0,7156356	KIAA0825	protein_coding
0,7118106	ZBTB37	protein_coding
0,6444859	RGPD2	protein_coding
0,9836846	ARL15	protein_coding
0,3715255	TCN2	protein_coding
0,7029174	ATP6V0A2	protein_coding
0,9450135	PRKN	protein_coding
0,6238595	HGS	protein_coding
0,673546	MRPL30	protein_coding
0,9865636	TARSL2	protein_coding
0,7816267	METTL7A	protein_coding
0,7490465	GPRIN3	protein_coding
0,0197802	ROR1	protein_coding
0,7912354	SDHAP1	transcribed_unprocessed_pseudogene
0,9112805	AC138393.1	transcribed_unprocessed_pseudogene
0,9954421	BRCC3	protein_coding
0,4533439	PRKG1	protein_coding
0,7671445	NR2F2	protein_coding
0,9107956	AHNAK2	protein_coding
0,9729242	OLFML2A	protein_coding
0,7397205	SP1	protein_coding
0,9590601	WASH3P	transcribed_unprocessed_pseudogene
0,6190365	MRPL40	protein_coding
0,8210922	PCGF3	protein_coding
0,6254273	P4HB	protein_coding
0,3245535	PSMD13	protein_coding
0,6511285	PBX1	protein_coding
0,997931	AC034236.1	processed_pseudogene
0,5964345	ZFP36L1	protein_coding
0,1712026	UBE2L3	protein_coding
0,9709611	BRWD1	protein_coding
0,8922683	EP400P1	transcribed_unprocessed_pseudogene
0,7213265	SMG1P4	transcribed_unprocessed_pseudogene
0,9846914	MOSMO	protein_coding
0,4593896	DRG1	protein_coding
0,6462349	ANKFY1	protein_coding
0,9544158	YTHDF3	protein_coding
0,578003	SRL	protein_coding
0,9928407	IFIT1	protein_coding
0,7611529	MORF4L1	protein_coding
0,533016	PIGP	protein_coding
0,8301309	BCAP31	protein_coding
0,4826313	ARL17A	protein_coding
0,7431973	RPL12P4	processed_pseudogene
0,8422988	DNAH14	protein_coding
0,9762117	NPIPB4	protein_coding
0,1112135	TRIM69	protein_coding
0,6563172	IFITM1	protein_coding
0,9891373	LAMP1	protein_coding
0,9463928	KLHDC8B	protein_coding
0,9773277	SETD4	protein_coding
0,654521	RNPC3	protein_coding
0,9781971	ZNF267	protein_coding

0,8545769	IRS2	protein_coding
0,9925374	BICD2	protein_coding
0,4498511	TMLHE	protein_coding
0,7154026	SDHAP3	transcribed_unprocessed_pseudogene
0,8578622	RASA3	protein_coding
0,6574161	LRCH3	protein_coding
0,9968111	ZNF566	protein_coding
0,9636851	ZNF529	protein_coding
0,9680053	AIDA	protein_coding
0,9609096	C15orf41	protein_coding
0,8723583	AC012085.1	processed_pseudogene
0,9463928	GSAP	protein_coding
0,4672216	CYP2R1	protein_coding
0,6446908	ANKRD46	protein_coding
0,8788135	ZBTB6	protein_coding
0,6475905	POLR3C	protein_coding
0,8850351	CCDC84	protein_coding
0,9583561	POLR1D	protein_coding
0,8918731	MRTFB	protein_coding
0,9484395	ZNF17	protein_coding
0,7230802	TOR3A	protein_coding
0,0015119	PPP1CC	protein_coding
0,8919058	ZNF555	protein_coding
0,2737133	NAP1L3	protein_coding
0,3218826	PRELID2	protein_coding
0,2318378	BACE1	protein_coding
0,4919058	THBS2	protein_coding
0,8722519	RXRA	protein_coding
0,8875885	ZNF75D	protein_coding
0,90504	GOLGA8R	protein_coding
0,8409875	KPNA4	protein_coding
0,472118	TRDN	protein_coding
0,6903585	ZNF197	protein_coding
0,9538485	NAP1L2	protein_coding
0,8562264	RPS23	protein_coding
0,6475905	GNG2	protein_coding
0,2354584	BTN3A2	protein_coding
0,7118106	PCLO	protein_coding
0,8682799	INSIG1	protein_coding
0,7062909	SEPTIN10	protein_coding
0,7698372	SMYD4	protein_coding
0,8922683	GPATCH8	protein_coding
0,9508384	NF2	protein_coding
0,2737133	UBE2H	protein_coding
0,6128477	MIR22HG	lncRNA
0,8949542	KATNA1	protein_coding
0,8966801	FSD2	protein_coding
0,6297108	ARAP1	protein_coding
0,6999614	PDE2A	protein_coding
0,6042348	ZFP91	protein_coding
0,4888499	LYRM7	protein_coding
0,7291201	BCR	protein_coding
0,7054786	ZNF397	protein_coding
0,7154026	ZSCAN30	protein_coding
0,8373546	TPCN1	protein_coding
0,9465232	HEXIM1	protein_coding
0,7984578	MAPT	protein_coding
0,6604792	ZDHHC17	protein_coding
0,3358172	ZNF395	protein_coding

0,9781971	PPARA	protein_coding
0,6304019	RPS19BP1	protein_coding
0,4311333	TEAD1	protein_coding
0,9077931	ENTPD5	protein_coding
0,8992483	MITF	protein_coding
0,9861848	NAP1L1	protein_coding
0,2443691	CMC1	protein_coding
0,9535559	AKR1C1	protein_coding
0,7242009	RNF220	protein_coding
0,427478	SHTN1	protein_coding
0,9678404	ZNF546	protein_coding
0,7918194	TSPYL4	protein_coding
0,7912354	AC100800.1	lncRNA
0,6840329	SESTD1	protein_coding
0,5504165	FNBP1	protein_coding
0,5622225	DYNC2H1	protein_coding
0,9234217	RSBN1L	protein_coding
0,6824068	TAF9B	protein_coding
0,9933219	MAGI2	protein_coding
0,6662301	CHP1	protein_coding
0,5327661	FPR3	protein_coding
0,8672415	COL4A1	protein_coding
0,6473313	PTMA	protein_coding
0,7433064	HSPA14	protein_coding
0,9933219	USP7	protein_coding
0,7972313	MAGEH1	protein_coding
0,6658691	RGPD1	protein_coding
0,5558351	PERM1	protein_coding
0,3792977	TMSB4XP8	processed_pseudogene
0,578739	ERC2	protein_coding
0,6762366	B3GLCT	protein_coding
0,1916186	SPRY4	protein_coding
0,1356256	C2orf88	protein_coding
0,7118106	TMEM203	protein_coding
0,9794449	THSD4	protein_coding
0,9282881	TCEA1	protein_coding
0,9194718	SECISBP2	protein_coding
0,5777334	SEMA4D	protein_coding
0,8831146	MCRS1	protein_coding
0,808959	FANCM	protein_coding
0,887128	HIST1H1C	protein_coding
0,7362379	FAM122A	protein_coding
0,2283663	COL14A1	protein_coding
0,8117718	UBQLN2	protein_coding
0,7291254	RILPL1	protein_coding
0,5180247	C11orf95	protein_coding
0,3316892	GPR89B	protein_coding
0,9952606	COL4A5	protein_coding
0,7154506	ZNF626	protein_coding
0,9516698	ZC3H6	protein_coding
0,9768456	LAMTOR4	protein_coding
0,7154026	DCUN1D3	protein_coding
0,6383874	ZNF793	protein_coding
0,4318356	TUBB4B	protein_coding
0,9485776	AGAP4	protein_coding
0,7337852	COMMD6	protein_coding
0,8155634	PLA2G2A	protein_coding
0,9463928	ZNF383	protein_coding
0,2908312	PLSCR1	protein_coding

0,9781148	ZNF559	protein_coding
0,3520154	GTF2F2	protein_coding
0,9925374	FAM92A	protein_coding
0,9954421	FOCAD	protein_coding
0,9738194	CHM	protein_coding
0,9925374	BLOC1S5	protein_coding
0,9127443	SRSF10	protein_coding
0,0640162	HBA2	protein_coding
0,7102291	CCDC9B	protein_coding
0,4344504	NBR1	protein_coding
0,9670362	RALGAPA2	protein_coding
0,9951314	SUMO2	protein_coding
0,9802452	RTL6	protein_coding
0,7704778	DPYD	protein_coding
0,6543081	S100A16	protein_coding
0,8545769	PTAR1	protein_coding
0,3222894	PARVB	protein_coding
0,5869872	UROS	protein_coding
0,3435112	ZDHHC9	protein_coding
0,9583561	SMIM15	protein_coding
0,4521055	VWC2	protein_coding
0,9367988	FSIP2	protein_coding
0,6563172	PRELP	protein_coding
0,9296784	ZNF548	protein_coding
0,9484395	MTF1	protein_coding
0,992369	ZNF322P1	processed_pseudogene
0,9709611	NHLRC3	protein_coding
0,9177329	ZDHHC11	protein_coding
0,9628588	RPL14	protein_coding
0,6383874	RPSAP47	processed_pseudogene
0,9081173	MSL1	protein_coding
0,8371307	LRRK2	protein_coding
0,8476238	TRMT2B	protein_coding
0,8219774	HACD4	protein_coding
0,7118106	FAM120AOS	protein_coding
0,9771336	NOC2L	protein_coding
0,861259	ZNF292	protein_coding
0,504498	SHLD2P3	unprocessed_pseudogene
0,888621	ZNF567	protein_coding
0,1729437	NDUFA4	protein_coding
0,9112805	RELN	protein_coding
0,0132445	APOD	protein_coding
0,4845704	H1F0	protein_coding
0,426935	LITAF	protein_coding
0,7247675	TMEM120A	protein_coding
0,7499102	ARID2	protein_coding
0,6624016	SF3B3	protein_coding
0,9997554	UBE2Q2P1	transcribed_unprocessed_pseudogene
0,887319	ZNF573	protein_coding
0,9194718	ZNF527	protein_coding
0,5474737	S100A13	protein_coding
0,8895276	ZNF33A	protein_coding
0,7639764	PCDH18	protein_coding
0,7203672	BTBD8	protein_coding
0,8801863	DPY19L2P1	transcribed_unprocessed_pseudogene
0,8255851	MAOA	protein_coding
0,9183994	PAX8-AS1	lncRNA
0,4883835	TSPYL1	protein_coding
0,9997554	PNRC2	protein_coding

0,9194118	RRP7A	protein_coding
0,7954765	LIN54	protein_coding
0,7630684	FAM53B	protein_coding
0,5879554	SLC35E2B	protein_coding
0,8622656	RPS2P46	processed_pseudogene
0,1382998	TOGARAM2	protein_coding
0,9082846	NEMP2	protein_coding
0,9854182	GSPT2	protein_coding
0,8206562	HMGB1	protein_coding
0,8561515	USP32P3	transcribed_unprocessed_pseudogene
0,9362082	BLOC1S2	protein_coding
0,7740102	IL1RAP	protein_coding
0,1131434	ZNF676	protein_coding
0,4344705	ZNF699	protein_coding
0,8528913	TDRD7	protein_coding
0,1412577	HLA-DRB1	protein_coding
0,7842706	AKR1C3	protein_coding
0,9450135	SPATS2L	protein_coding
0,993752	ZNF250	protein_coding
0,7956408	WDSUB1	protein_coding
0,6999614	S100A4	protein_coding
0,4848227	FAT4	protein_coding
0,8875885	ACADSB	protein_coding
0,5062136	STK40	protein_coding
0,8500846	TMEM63A	protein_coding
0,3087684	MPHOSPH8	protein_coding
0,5017459	EEF1A1P5	processed_pseudogene
0,9761718	ZNF766	protein_coding
0,997931	FAM217B	protein_coding
0,6765217	TUBB	protein_coding
0,9315593	LCOR	protein_coding
0,6084958	SUPT5H	protein_coding
0,9540553	XPNPEP3	protein_coding
0,8108219	ZNF107	protein_coding
0,8427405	PPIA	protein_coding
0,4835164	ZNF471	protein_coding
0,8575378	ZNF493	protein_coding
0,9572126	GTF2IRD2	protein_coding
0,5768067	NIF3L1	protein_coding
0,8847715	GARS-DT	lncRNA
0,9536474	IARS	protein_coding
0,9738194	MFSD14C	transcribed_unprocessed_pseudogene
0,9768456	POM121	protein_coding
0,9287437	ZBTB44	protein_coding
0,9755908	CD55	protein_coding
0,8422988	WDR5	protein_coding
0,9312186	LONP1	protein_coding
0,8318664	TRRAP	protein_coding
0,6620763	SRGAP2B	protein_coding
0,8721867	ZNF140	protein_coding
0,9127443	PTPN1	protein_coding
0,3178501	EVL	protein_coding
0,993752	EPHB4	protein_coding
0,922549	ZNF765	protein_coding
0,6199488	ZNF124	protein_coding
0,6254273	XRCC6	protein_coding
0,6140495	TSC22D2	protein_coding
0,9956922	ZNF569	protein_coding
0,7952381	ARMCX4	protein_coding

0,9759014	PIK3R4	protein_coding
0,8176511	ZNF605	protein_coding
0,9998538	TRAPPC2	protein_coding
0,8318664	SIAH1	protein_coding
0,7175299	ESRRG	protein_coding
0,9756408	NCOR2	protein_coding
0,9021812	SULT1A1	protein_coding
0,8438938	PRPF40A	protein_coding
0,9406097	GDAP2	protein_coding
0,9738194	TCEAL3	protein_coding
0,2533818	ANAPC7	protein_coding
0,642899	TPK1	protein_coding
0,7104968	AFAP1	protein_coding
0,6613295	NACA	protein_coding
0,6229784	MYO18A	protein_coding
0,6588074	MAN2A2	protein_coding
0,9998538	MME	protein_coding
0,5723744	SULF2	protein_coding
0,8930787	LAMA2	protein_coding
0,1517576	PLXNB2	protein_coding
0,9238608	MYO6	protein_coding
0,9290744	MRTFA	protein_coding
0,5511615	HDAC2	protein_coding
0,9908419	ZNF782	protein_coding
0,806905	ZNF846	protein_coding
0,9112805	ADH1B	protein_coding
0,8578793	TCF4	protein_coding
0,784187	RABL6	protein_coding
0,9170247	ZNF136	protein_coding
0,7740102	ZKSCAN5	protein_coding
0,9464798	TRAPPC4	protein_coding
0,9379013	AC004057.1	transcribed_processed_pseudogene
0,7213265	TECPR2	protein_coding
0,9887908	ZFP62	protein_coding
0,855954	ERI2	protein_coding
0,9699064	TOMM7	protein_coding
0,7010516	ZNF33B	protein_coding
0,9618453	AMZ2	protein_coding
0,7552965	ZNF431	protein_coding
0,928336	NF1	protein_coding
0,654521	VKORC1L1	protein_coding
0,9644268	ZNF418	protein_coding
0,2945575	DAPK1	protein_coding
0,0390866	HLA-DQA1	protein_coding
0,6731385	GM2A	protein_coding
0,9891373	ZNF700	protein_coding
0,9953531	CD47	protein_coding
0,5276954	TLE1	protein_coding
0,8834615	STRN3	protein_coding
0,2506935	ILRUN	protein_coding
0,290302	PPTC7	protein_coding
0,8992483	RGPD4	protein_coding
0,9803555	NHLRC2	protein_coding
0,7744962	ZFP28	protein_coding
0,1933793	CBWD3	protein_coding
0,8877092	KPNA5	protein_coding
0,8745367	ANKRD36B	protein_coding
0,8447416	ARHGEF12	protein_coding
0,1100299	ZNF252P	transcribed_unprocessed_pseudogene

0,9074092	FLNA	protein_coding
0,8875885	SRGAP1	protein_coding
0,5647201	FAM3C	protein_coding
0,5210286	NOP9	protein_coding
0,4498511	SLC39A10	protein_coding
0,7816267	CASP4	protein_coding
0,90504	AP2A1	protein_coding
0,8425029	ZNF585A	protein_coding
0,7444016	FUT11	protein_coding
0,3844531	ANXA4	protein_coding
0,9277595	WDR45	protein_coding
0,8622656	METTL9	protein_coding
0,9700303	ZNF138	protein_coding
0,711869	ZNF429	protein_coding
0,7258655	ZNF470	protein_coding
0,9463928	ZNF100	protein_coding
0,9087161	ZNF398	protein_coding
0,9439974	ZSCAN25	protein_coding
0,8619772	ANXA6	protein_coding
0,9828271	ZNF441	protein_coding
0,8782347	GMFB	protein_coding
0,998169	ZNF420	protein_coding
0,7713242	ZMYM1	protein_coding
0,8318664	HIST1H4C	protein_coding
0,8992483	ZSCAN26	protein_coding
0,933129	MAFG	protein_coding
0,9082846	KIAA1671	protein_coding
0,7942035	IGF2R	protein_coding
0,7613174	DYNC1H1	protein_coding
0,7467854	PCBP2	protein_coding
0,4675238	PGAP1	protein_coding
0,7237365	ZNF772	protein_coding
0,5985056	ADAM32	protein_coding
0,8087971	ACSL5	protein_coding
0,9781971	LRRC8B	protein_coding
0,6999614	SND1	protein_coding
0,6664224	PSMD12	protein_coding
0,9700639	NOL4L	protein_coding
0,5511615	ENTPD4	protein_coding
0,5011485	C1D	protein_coding
0,6238595	TBC1D9B	protein_coding
0,504498	KANK2	protein_coding
0,4894579	EIF4BP6	processed_pseudogene
0,9367988	GTF2E2	protein_coding
0,8851105	FITM2	protein_coding
0,8114908	ZNF720	protein_coding
0,9296784	DDI2	protein_coding
0,5393446	SVIL	protein_coding
0,864196	TRIM33	protein_coding
0,8301309	LRP10	protein_coding
0,8885864	PELI1	protein_coding
0,9581517	ZNF655	protein_coding
0,2281984	MRPL21	protein_coding
0,7805908	SLC22A5	protein_coding
0,730414	ADARB1	protein_coding
0,9919804	HTT	protein_coding
0,3792977	IPP	protein_coding
0,5688668	MAP3K5	protein_coding
0,7506449	GSTK1	protein_coding

0,4724079	HNRNPAB	protein_coding
0,7228252	PDGFA	protein_coding
0,3222894	RPF2	protein_coding
0,9428271	MYO5A	protein_coding
0,9998478	C5orf56	lncRNA
0,9998538	ATG7	protein_coding
0,3177457	SIPA1L1	protein_coding
0,933129	TTC30A	protein_coding
0,7611529	PIGN	protein_coding
0,6088362	COL4A6	protein_coding
0,9538485	TOPORS	protein_coding
0,654521	BCO2	protein_coding
0,8722519	ENTPD6	protein_coding
0,7931208	ENPP1	protein_coding
0,8163405	FAR1	protein_coding
0,9327336	CPLANE1	protein_coding
0,6543081	ZNF841	protein_coding
0,0895372	MFAP5	protein_coding
0,4835164	MYH6	protein_coding
0,7118106	ZNF615	protein_coding
0,7481835	CXorf40A	protein_coding
0,8225056	CDC42SE1	protein_coding
0,9363291	MPEG1	protein_coding
0,5267449	SPTAN1	protein_coding
0,7291201	PARVA	protein_coding
0,1801696	FAM114A1	protein_coding
0,3316892	RPE	protein_coding
0,6574161	ZNF460	protein_coding
0,8238032	PHF2	protein_coding
0,7936863	RPS26	protein_coding
0,8221205	PSAP	protein_coding
0,9115427	S100A10	protein_coding
0,965142	RPL37A	protein_coding
0,8890235	TXNRD3	protein_coding
0,6986866	CFD	protein_coding
0,9998478	MCMBP	protein_coding
0,7443726	KLHDC1	protein_coding
0,799869	ZNF81	protein_coding
0,5709725	TAF13	protein_coding
0,8966801	ZNF780A	protein_coding
0,6254273	FAM118B	protein_coding
0,7456311	SLC9A8	protein_coding
0,6476315	ZNF181	protein_coding
0,8155634	INKA2	protein_coding
0,7611529	ZNF44	protein_coding
0,5265966	SGTB	protein_coding
0,9142106	ZNF790	protein_coding
0,933129	FAM49A	protein_coding
0,6682217	MYO1C	protein_coding
0,4237568	NKIRAS1	protein_coding
0,3881918	KIF13B	protein_coding
0,7352491	NRAP	protein_coding
0,9364548	ADH5	protein_coding
0,9998478	SPG7	protein_coding
0,97835	HRNR	protein_coding
0,8653179	ZNF677	protein_coding
0,9484395	ERO1A	protein_coding
0,9463928	ZNF347	protein_coding
0,5323751	PLCG2	protein_coding

0,9463928	S100A6	protein_coding
0,561634	RPL12	protein_coding
0,671498	DNM3	protein_coding
0,9700303	ZNF121	protein_coding
0,7542355	MPZL1	protein_coding
0,6098976	VPS13A	protein_coding
0,6277796	MBP	protein_coding
0,8825313	GOLGA6L9	protein_coding
0,4957019	C1orf122	protein_coding
0,9998478	NOL8	protein_coding
0,8324551	IRAK4	protein_coding
0,6516554	MRPL42	protein_coding
0,7987982	ENTPD7	protein_coding
0,7546576	RPS4X	protein_coding
0,8863508	ZNF84	protein_coding
0,9057527	MAK16	protein_coding
0,7088772	ZNF667	protein_coding
0,8640757	SIRPA	protein_coding
0,6604792	MARCH5	protein_coding
0,9544158	NPIPB13	protein_coding
0,9842675	CD2AP	protein_coding
0,8682799	ZNF649	protein_coding
0,6285359	ZNF248	protein_coding
0,8847715	LPAR1	protein_coding
0,6473313	MB	protein_coding
0,6371005	HIBCH	protein_coding
0,9831527	ZNF544	protein_coding
0,8355525	ZNF770	protein_coding
0,9386808	MIER1	protein_coding
0,8189129	MAN1A2	protein_coding
0,7558125	SVIP	protein_coding
0,7558125	ZNF251	protein_coding
0,9264586	DDRGK1	protein_coding
0,9287437	TFDP1	protein_coding
0,9767278	HSD17B11	protein_coding
0,9406097	SZT2	protein_coding
0,9296784	QRICH1	protein_coding
0,8871625	FKBP1C	protein_coding
0,3327196	DDX42	protein_coding
0,3600539	AC131392.1	unprocessed_pseudogene
0,8833614	RPL23A	protein_coding
0,8238032	STYX	protein_coding
0,5544626	UBL5	protein_coding
0,624851	HELZ	protein_coding
0,284683	TMEM116	protein_coding
0,7558125	PEG3	protein_coding
0,9792506	SDAD1	protein_coding
0,6899599	ZKSCAN8	protein_coding
0,141284	MYL4	protein_coding
0,8295224	GET3	protein_coding
0,9828457	ASPH	protein_coding
0,5660793	SPRED2	protein_coding
0,3514523	WWP2	protein_coding
0,2329611	GFPT1	protein_coding
0,6254273	UVRAG	protein_coding
0,9364287	ZNF26	protein_coding
0,8847411	ITSN2	protein_coding
0,7692025	BZW1P2	processed_pseudogene
0,445647	OGA	protein_coding

0,5879554	ZNF658B	transcribed_unprocessed_pseudogene
0,9033043	TCAF1	protein_coding
0,9792506	TXNRD1	protein_coding
0,7118106	ZNF568	protein_coding
0,4806087	ZXDB	protein_coding
0,4943386	ZNF480	protein_coding
0,9082846	ZNF587	protein_coding
0,5558351	TPM2	protein_coding
0,7880474	SH3BGRL2	protein_coding
0,993752	ZNF808	protein_coding
0,997931	YTHDF2	protein_coding
0,8851105	TMA16	protein_coding
0,2313926	HLA-DRB5	protein_coding
0,8259599	ZNF43	protein_coding
0,7357059	GPN1	protein_coding
0,7118106	PLN	protein_coding
0,7894641	ZNF28	protein_coding
0,6322951	ZNF627	protein_coding
0,6638064	CTNND1	protein_coding
0,7740102	DDX39B	protein_coding
0,9074092	NUDT16	protein_coding
0,7559894	TLK1	protein_coding
0,9125449	LRBA	protein_coding
0,962885	BAZ1A	protein_coding
0,6244656	COPS8	protein_coding
0,8269408	PPIAP22	processed_pseudogene
0,2506935	CCDC69	protein_coding
0,9962215	MDM4	protein_coding
0,8565825	RYR2	protein_coding
0,8949542	KLHL9	protein_coding
0,9299044	NCOA6	protein_coding
0,7114715	STK39	protein_coding
0,5111636	C6orf89	protein_coding
0,6371005	CALM1	protein_coding
0,7842247	TTC37	protein_coding
0,3358222	PAPSS2	protein_coding
0,4513092	SLC9A6	protein_coding
0,7135352	FAN1	protein_coding
0,9003006	EIF1AY	protein_coding
0,9422708	MT-ND6	protein_coding
0,9928407	IPO9	protein_coding
0,7740102	CEP290	protein_coding
0,7639764	MT-CO2	protein_coding
0,8992483	TOGARAM1	protein_coding
0,4684539	ECI2	protein_coding
0,8625711	UNC13B	protein_coding
0,8825313	MT-CYB	protein_coding
0,3616153	LDB1	protein_coding
0,4422012	PPP1R14C	protein_coding
0,5180247	CTR9	protein_coding
0,5440543	F5	protein_coding
0,6660548	LRRTM3	protein_coding
0,7249384	ZNF652	protein_coding
0,8641364	SMURF1	protein_coding
0,7968733	SLC5A3	protein_coding
0,7924243	MTCO3P12	unprocessed_pseudogene
0,6673583	CDC42BPB	protein_coding
0,6951729	RPL10A	protein_coding
0,3480863	COLGALT2	protein_coding

0,949756	MT-ND2	protein_coding
0,5011485	RCSD1	protein_coding
0,7558125	FAM169A	protein_coding
0,8463447	MT-ND5	protein_coding
0,8825313	CNOT7	protein_coding
0,9998538	TMEM184B	protein_coding
0,9575231	MTOR	protein_coding
0,9998538	ZNF521	protein_coding
0,4521055	ALPK2	protein_coding
0,9680053	LRIG2	protein_coding
0,8545769	MT-CO1	protein_coding
0,1528801	LRRC10	protein_coding
0,9205351	GK	protein_coding
0,7816267	FOXJ3	protein_coding
0,9458749	SFT2D1	protein_coding
0,8130999	CHAMP1	protein_coding
0,253927	INPP5F	protein_coding
0,1064872	HMGN2	protein_coding
0,9376695	SELENOM	protein_coding
0,7318327	UBE2J1	protein_coding
0,7178359	OPA1	protein_coding
0,8500846	DENND4B	protein_coding
0,9457094	ZNF277	protein_coding
0,6023346	MT-ND3	protein_coding
0,9296784	DUSP27	protein_coding
0,6689227	SELENOT	protein_coding
0,6658691	CES1	protein_coding
0,9082846	RUSC2	protein_coding
0,9544158	OSTC	protein_coding
0,4408807	TSEN15	protein_coding
0,6383874	LTN1	protein_coding
0,9738194	RUNDC1	protein_coding
0,9700303	GRK5	protein_coding
0,3942661	TYW1	protein_coding
0,3616153	DCAF12	protein_coding
0,7188119	SFMBT2	protein_coding
0,9792506	MT-ND4	protein_coding
0,9647765	SMC5	protein_coding
0,9450135	MT-ND1	protein_coding
0,3358222	SHISA4	protein_coding
0,721162	CIPC	protein_coding
0,54953	CAPZA2	protein_coding
0,9338281	MT-ATP6	protein_coding
0,9928407	TOP1	protein_coding
0,4919121	MAP3K3	protein_coding
0,5346782	SREBF2	protein_coding
0,7401378	RPL39	protein_coding
0,8653179	DZIP3	protein_coding
0,445647	KIAA0753	protein_coding
0,8015756	DCLRE1A	protein_coding
0,9142106	ATG9A	protein_coding
0,2337466	GPRASP1	protein_coding
0,7673893	MT-CO3	protein_coding
0,7965978	DMD	protein_coding
0,5679388	NAGA	protein_coding
0,9894339	SMG5	protein_coding
0,7352523	KIF1BP	protein_coding
0,9647765	TGM2	protein_coding
0,6818653	ARMCX6	protein_coding

0,8578793	PJA2	protein_coding
0,9725364	SGMS1	protein_coding
0,6867028	RNA5SP370	rRNA_pseudogene
0,6796078	RNA5SP389	rRNA_pseudogene
0,6676251	RNA5SP226	rRNA_pseudogene
0,4344705	RNU5A-1	snRNA
0,5296182	Y_RNA	misc_RNA
0,7740102	RNA5SP145	rRNA_pseudogene
0,5245497	SNORA73B	snoRNA
0,6634961	RNU5B-1	snRNA
0,5817753	RN7SKP255	misc_RNA
0,5445815	Y_RNA	misc_RNA
0,6044319	RN7SKP203	misc_RNA
0,5183663	Y_RNA	misc_RNA
0,9628671	RNA5SP429	rRNA_pseudogene
0,9725364	RNU4-1	snRNA
0,4466289	SNORA74A	snoRNA
0,5573066	RNY1	misc_RNA
0,5777334	RNA5SP202	rRNA_pseudogene
0,7500896	RN7SKP71	misc_RNA
0,624851	RNU6-7	snRNA
0,5115453	RNY3P1	misc_RNA
0,5847346	SNORA23	snoRNA
0,824287	RN7SKP80	misc_RNA
0,5265966	RNY3	misc_RNA
0,7968733	RN7SKP230	misc_RNA
0,7984578	RNU4-2	snRNA
0,7385573	ZNF525	protein_coding
0,5276954	INF2	protein_coding
0,9917279	RBMS3-AS2	lncRNA
0,5558351	EFCAB2	protein_coding
0,8702296	CHML	protein_coding
0,7558125	TATDN3	protein_coding
0,8176511	MIR29B2CHG	lncRNA
0,0648859	CR1	protein_coding
0,8553175	FCGR3A	protein_coding
0,4747639	FAM229B	protein_coding
0,9642558	EEF1AKMT2	protein_coding
0,5082264	DDO	protein_coding
0,9112805	RBM20	protein_coding
0,8786805	SNHG5	lncRNA
0,9808899	GDI1	protein_coding
0,88513	PCMTD2	protein_coding
0,9233889	LINC00632	lncRNA
0,9309593	EFCAB7	protein_coding
0,6657649	LINC00963	lncRNA
0,8930787	SYS1	protein_coding
0,9967216	INPP5B	protein_coding
0,7823793	MAFB	protein_coding
0,483594	TRAF3IP1	protein_coding
0,6574161	CHIC1	protein_coding
0,864196	GIGYF2	protein_coding
0,8238032	RUFY2	protein_coding
0,8545769	NHSL2	protein_coding
0,391877	GGTA1P	protein_coding
0,8996267	PHACTR4	protein_coding
0,7137814	ASAH2B	protein_coding
0,0727176	TMEM273	protein_coding
0,9170247	BMS1P1	transcribed_unprocessed_pseudogene

0,8898615	MACO1	protein_coding
0,9039014	PTPN20	protein_coding
0,6129557	ZDBF2	protein_coding
0,611428	DAXX	protein_coding
0,7956173	BMPR2	protein_coding
0,8834615	TCEA3	protein_coding
0,3942661	RXRB	protein_coding
0,995098	HNRNPCP2	processed_pseudogene
0,7685768	BRD2	protein_coding
0,4721364	HLA-DMA	protein_coding
0,4626741	COL5A2	protein_coding
0,8375248	PSMB8	protein_coding
0,9537852	SPIN3	protein_coding
0,284683	HLA-DRA	protein_coding
0,445647	COL15A1	protein_coding
0,7558125	NOTCH4	protein_coding
0,7182975	PBX2	protein_coding
0,1712026	RNF5	protein_coding
0,998169	SKIV2L	protein_coding
0,9131236	NELFE	protein_coding
0,5393446	SDHD	protein_coding
0,8863637	NEU1	protein_coding
0,9528743	HSPA1B	protein_coding
0,9377639	HSPA1A	protein_coding
0,9828271	VARS	protein_coding
0,9874145	MBD5	protein_coding
0,9850681	C6orf47	protein_coding
0,9768456	BAG6	protein_coding
0,8500846	PRRC2A	protein_coding
0,887319	AIF1	protein_coding
0,9803555	ZNF814	protein_coding
0,8971447	ZNF551	protein_coding
0,4779341	ZNF805	protein_coding
0,4835164	HLA-C	protein_coding
0,7444016	DHX16	protein_coding
0,2437974	MRPS18B	protein_coding
0,3616153	PPP1R10	protein_coding
0,9802452	ABCF1	protein_coding
0,1601158	DDR1	protein_coding
0,5276954	GNL1	protein_coding
0,2337466	HLA-E	protein_coding
0,9609142	ZNF468	protein_coding
0,5511615	ZNF616	protein_coding
0,3706065	PPP1R11	protein_coding
0,9625751	ZNRD1ASP	transcribed_unitary_pseudogene
0,8695843	RACK1	protein_coding
0,6023346	TBC1D8	protein_coding
0,290432	HLA-F	protein_coding
0,684772	LINC02210	transcribed_unitary_pseudogene
0,9450135	RPS26P8	processed_pseudogene
0,7395639	GABBR1	protein_coding
0,284683	TRIM27	protein_coding
0,9463928	AC083899.1	unprocessed_pseudogene
0,3792977	RANBP17	protein_coding
0,5890453	CBWD4P	unprocessed_pseudogene
0,6462349	ZNF204P	processed_pseudogene
0,8992483	ATXN2	protein_coding
0,7607016	DCTN1	protein_coding
0,9284465	TCTN1	protein_coding

0,9729242	MZT1	protein_coding
0,7081667	TRIM13	protein_coding
0,3513961	PKHD1L1	protein_coding
0,8992483	SLC35B4	protein_coding
0,8438109	TRIQK	protein_coding
0,9962215	ZBTB10	protein_coding
0,8229361	LGR4	protein_coding
0,1232731	VIT	protein_coding
0,7892243	E2F4	protein_coding
0,5170218	PDE7A	protein_coding
0,90504	TMEM170B	protein_coding
0,913228	SNX2	protein_coding
0,9484395	IPO7	protein_coding
0,8176511	PRR13	protein_coding
0,166273	INSYN1	protein_coding
0,6305527	SAMD9	protein_coding
0,620901	CNEP1R1	protein_coding
0,645515	CCDC85C	protein_coding
0,9894339	NAP1L4	protein_coding
0,7359516	SMG1P2	transcribed_unprocessed_pseudogene
0,4806087	TMSB4X	protein_coding
0,6084958	SMN2	protein_coding
0,5117223	SERF1B	protein_coding
0,2354584	HMGN1	protein_coding
0,9841237	STAG3L1	transcribed_unprocessed_pseudogene
0,7065438	EIF3CL	protein_coding
0,9928407	LCMT1	protein_coding
0,9668896	LIN52	protein_coding
0,5964345	SMIM11A	protein_coding
0,5336185	TECRL	protein_coding
0,7985804	DPF3	protein_coding
0,4336027	ETFRF1	protein_coding
0,9033043	ITSN1	protein_coding
0,2908312	ITPRIPL2	protein_coding
0,6527079	CRYZL1	protein_coding
0,8969303	C5orf51	protein_coding
0,5402971	RPS3AP47	processed_pseudogene
0,9481776	RNPS1	protein_coding
0,3087684	NYNRIN	protein_coding
0,284683	DNAJC19	protein_coding
0,8868311	DOK6	protein_coding
0,7395639	JPT2	protein_coding
0,7025012	ZDHHC11B	protein_coding
0,7154026	GOLGA8O	protein_coding
0,5140003	HERC2P9	transcribed_unprocessed_pseudogene
0,073095	HBA1	protein_coding
0,462729	HCP5	lncRNA
0,4886257	HLA-H	unprocessed_pseudogene
0,7397205	COL6A6	protein_coding
0,4999985	RAB12	protein_coding
0,2330928	HLA-A	protein_coding
0,5916421	HACD2	protein_coding
0,7639764	CFAP44	protein_coding
0,6184079	VGLL3	protein_coding
0,9699064	ANKRD28	protein_coding
0,2354584	COLQ	protein_coding
0,7774855	THUMPD3-AS1	lncRNA
0,6632698	RNVU1-7	snRNA
0,7467854	RNU1-28P	snRNA

0,7010516	RNU1-27P	snRNA
0,5026144	RNU6-1	snRNA
0,6173428	Y_RNA	misc_RNA
0,6662301	RNU1-1	snRNA
0,7736716	RNVU1-18	snRNA
0,6591379	RNU6-36P	snRNA
0,6462349	RNU6-4P	snRNA
0,5445815	RNU6-5P	snRNA
0,6887436	RNU1-2	snRNA
0,8099847	SNORA54	snoRNA
0,5688668	RNU6-3P	snRNA
0,522489	Y_RNA	misc_RNA
0,6337897	RNU6-2	snRNA
0,7010516	RNU1-4	snRNA
0,4954628	SNORD15B	snoRNA
0,620901	RNU6-9	snRNA
0,6563172	RNU1-3	snRNA
0,5647201	RNU6-33P	snRNA
0,6785825	SNORD94	snoRNA
0,9998478	SNORA49	snoRNA
0,6658884	MT-RNR2	Mt_rRNA
0,9410289	MT-TA	Mt_tRNA
0,9341044	MT-TN	Mt_tRNA
0,9982092	MT-TC	Mt_tRNA
0,7984578	MT-TY	Mt_tRNA
0,6607313	MT-TP	Mt_tRNA
0,4222223	GPX3	protein_coding
0,8875885	DIO2	protein_coding
0,8375248	STK38L	protein_coding
0,8619772	SACM1L	protein_coding
0,6835435	MT-RNR1	Mt_rRNA
0,9412812	TSN	protein_coding
0,7054786	SLC48A1	protein_coding
0,3943278	SNORD17	snoRNA
0,3305341	SNORA74B	snoRNA
0,5245497	SNORA53	snoRNA
0,6903585	SNORA12	snoRNA
0,984386	AC064799.1	processed_pseudogene
0,9781971	LINC02693	lncRNA
0,4302368	RPL15P3	processed_pseudogene
0,9953531	MT-ND4L	protein_coding
0,887128	RPS26P6	processed_pseudogene
0,9479329	ZNF611	protein_coding
0,997931	NUP62	protein_coding
0,9781971	DENND1B	protein_coding
0,7321942	SFT2D2	protein_coding
0,7972313	FGFR1OP	protein_coding
0,9082846	SCAF8	protein_coding
0,2543569	ACKR1	protein_coding
0,8888699	ZNF254	protein_coding
0,2046145	KLHL23	protein_coding
0,8323828	RPL22P1	processed_pseudogene
0,7682033	MLLT11	protein_coding
0,9998538	NRAS	protein_coding
0,9789623	HNRNPA3P6	processed_pseudogene
0,9925374	RPS7P11	processed_pseudogene
0,7542355	CHUK	protein_coding
0,7213265	GSTM2	protein_coding
0,7558125	HSPD1P1	processed_pseudogene

0,623981	RPL18AP3	processed_pseudogene
0,9377639	ERV3-1	protein_coding
0,8369686	SYNJ2BP	protein_coding
0,9840915	RBMXL1	protein_coding
0,8111745	SRA1	protein_coding
0,9682998	RPLP0P6	processed_pseudogene
0,4954628	VDAC1	protein_coding
0,077831	TMX2	protein_coding
0,9528194	HEXA	protein_coding
0,2506935	NDUFS3	protein_coding
0,9984673	RPSAP54	processed_pseudogene
0,9994264	LEPROT	protein_coding
0,0286685	LBH	protein_coding
0,9625751	PPP1CB	protein_coding
0,2778507	NCKIPSD	protein_coding
0,5318944	ATF6B	protein_coding
0,0093815	S1PR3	protein_coding
0,9248086	SLC35F6	protein_coding
0,993752	HMGB1P10	processed_pseudogene
0,9946475	CLIC1	protein_coding
0,438012	DDAH2	protein_coding
0,7397205	RPS29	protein_coding
0,998169	CENPBD1P1	transcribed_processed_pseudogene
0,7743102	AC020898.1	processed_pseudogene
0,9479329	ZNF134	protein_coding
0,893888	ZNF845	protein_coding
0,7645994	EMP2	protein_coding
0,9771336	RPL21P75	processed_pseudogene
0,8318519	YBX1P10	processed_pseudogene
0,730414	RPL13AP7	transcribed_processed_pseudogene
0,8882787	CSNK1E	protein_coding
0,997931	GALT	protein_coding
0,8076343	ITGA1	protein_coding
0,2318378	NUDT19	protein_coding
0,9225269	NAXD	protein_coding
0,6591127	GANC	protein_coding
0,5485366	TTLL3	protein_coding
0,9907221	REPIN1	protein_coding
0,7576959	ZNF891	protein_coding
0,9233889	SMIM7	protein_coding
0,8847715	CPNE1	protein_coding
0,2337466	SMCO1	protein_coding
0,7095364	PAXIP1-AS2	lncRNA
0,8500846	LYRM4	protein_coding
0,5879554	AC132008.2	transcribed_unprocessed_pseudogene
0,9998538	AMZ2P1	transcribed_unprocessed_pseudogene
0,6467114	SMIM30	protein_coding
0,6319662	EEF1A1P12	processed_pseudogene
0,3286712	HNRNPA1P10	processed_pseudogene
0,9484259	FIS1	protein_coding
0,9840915	AC009053.1	transcribed_unprocessed_pseudogene
0,6653209	NEURL1B	protein_coding
0,9028298	HAUS3	protein_coding
0,7249384	RPS3AP26	processed_pseudogene
0,685088	TUBAP2	processed_pseudogene
0,7118106	BBIP1	protein_coding
0,7268431	LRRC37A4P	transcribed_unprocessed_pseudogene
0,8117718	PLIN5	protein_coding
0,9982092	RPL7P1	processed_pseudogene

0,9953531	PPME1	protein_coding
0,993752	GTF2IRD2P1	transcribed_unprocessed_pseudogene
0,4315528	MEG3	lncRNA
0,5393446	ZNF727	protein_coding
0,6999614	ZSWIM8	protein_coding
0,9359033	ZBED1	protein_coding
0,9544158	HNRNPUL2	protein_coding
0,913228	SEPTIN7P2	transcribed_unprocessed_pseudogene
0,654521	AC010468.1	processed_pseudogene
0,3616153	CYCSP55	processed_pseudogene
0,9379013	ZSWIM7	protein_coding
0,8319167	CRPPA	protein_coding
0,8638437	NPIPA7	protein_coding
0,8847715	PPIAP29	processed_pseudogene
0,9952606	PHB2	protein_coding
0,4246004	RPL13P12	processed_pseudogene
0,685088	EEF1A1P29	processed_pseudogene
0,3607643	TTC3P1	processed_pseudogene
0,520643	UBXN2B	protein_coding
0,2068936	CBWD6	protein_coding
0,8061393	AC138409.2	transcribed_unprocessed_pseudogene
0,8847411	PEX26	protein_coding
0,8801614	AC116351.1	lncRNA
0,1140347	FASTKD5	protein_coding
0,8257564	GOLGA8B	protein_coding
0,7542355	DDX3X	protein_coding
0,771306	AC127502.1	transcribed_unprocessed_pseudogene
0,9998538	VPS16	protein_coding
0,4806087	MIR99AHG	lncRNA
0,284683	ZNF407	protein_coding
0,4153382	HNRNPA1P7	processed_pseudogene
0,2644142	TMEM242	protein_coding
0,7883677	TMEM167B	protein_coding
0,8375248	SLC35E2A	transcribed_unprocessed_pseudogene
0,9840915	ZC3H11B	protein_coding
0,5453655	AC245297.1	transcribed_unprocessed_pseudogene
0,9112805	AC078819.1	processed_pseudogene
0,998169	RPS2P55	processed_pseudogene
0,5777776	CCDC7	protein_coding
0,8247964	FNIP1	protein_coding
0,565522	RPS10P3	processed_pseudogene
0,721162	MORF4L1P1	processed_pseudogene
0,3814605	TENM3	protein_coding
0,5245497	AL591135.1	processed_pseudogene
0,7542355	AL590867.2	processed_pseudogene
0,3943278	CEBPZOS	protein_coding
0,7954765	AL033519.3	processed_pseudogene
0,9523288	AL592114.1	processed_pseudogene
0,9412812	AC138392.1	processed_pseudogene
0,5982405	NBPF1	protein_coding
0,9359783	FTH1P8	processed_pseudogene
0,997931	UMAD1	protein_coding
0,9998478	FAM228B	protein_coding
0,7385573	VAMP2	protein_coding
0,992936	RPL21P28	processed_pseudogene
0,9755908	RPL21P119	processed_pseudogene
0,9781971	RPL21P16	processed_pseudogene
0,0908653	EBF2	protein_coding
0,7956173	PPP3R1	protein_coding

0,4835164	PLXNA4	protein_coding
0,8992483	CEBPD	protein_coding
0,7213265	NPTXR	protein_coding
0,7869275	PPP2R2A	protein_coding
0,7892243	ZNF880	protein_coding
0,7662231	TMEM14EP	transcribed_processed_pseudogene
0,7571978	APOL6	protein_coding
0,5825918	FADS3	protein_coding
0,8189129	CCNL2	protein_coding
0,7834554	UBA52	protein_coding
0,0473388	MYBPHL	protein_coding
0,9074092	FAM185A	protein_coding
0,5994444	CYTOR	lncRNA
0,9374881	SNORA79B	snoRNA
0,5558351	RNU2-64P	snRNA
0,855954	FTH1P10	transcribed_processed_pseudogene
0,9925374	RPS26P15	processed_pseudogene
0,7385573	TCAF1P1	unprocessed_pseudogene
0,6815498	NUTM2A-AS1	lncRNA
0,8917504	EXOSC6	protein_coding
0,6854132	VPS52	protein_coding
0,9237476	AC135983.2	transcribed_processed_pseudogene
0,6371005	EEF1A1P8	processed_pseudogene
0,8721867	LINC00630	lncRNA
0,6494315	ZNF844	protein_coding
0,5357584	TMSB4XP4	processed_pseudogene
0,9670362	NSUN5P1	transcribed_unprocessed_pseudogene
0,9379013	CCDC18-AS1	lncRNA
0,0317466	HLA-DPB1	protein_coding
0,77251	OSER1-DT	lncRNA
0,141284	AC117453.1	lncRNA
0,4954628	AFG3L1P	transcribed_unitary_pseudogene
0,9781971	CHROMR	lncRNA
0,8721867	SNHG14	lncRNA
0,9838551	RPS24P8	processed_pseudogene
0,5111636	C4B	protein_coding
0,9077931	HSP90AA2P	processed_pseudogene
0,5647215	ATXN1L	protein_coding
0,8417824	SMIM13	protein_coding
0,4684539	EIF4BP3	processed_pseudogene
0,2337466	HNRNPA1P48	protein_coding
0,5400662	SVIL-AS1	transcribed_unprocessed_pseudogene
0,7528871	RPS27AP16	transcribed_processed_pseudogene
0,9618453	SH3BP5-AS1	lncRNA
0,9901318	NPIPA3	protein_coding
0,7171927	AC093843.1	lncRNA
0,7154026	RPL29P11	processed_pseudogene
0,8500846	YBX1P1	processed_pseudogene
0,8093741	NDUFAF8	protein_coding
0,9082846	LINC00863	lncRNA
0,8745367	LINC01266	lncRNA
0,4899954	EIF4BP7	processed_pseudogene
0,9618453	AC004552.1	processed_pseudogene
0,8847411	DYNC1I2P1	processed_pseudogene
0,7062909	GOLGA2P7	transcribed_unprocessed_pseudogene
0,8992483	RPSAP58	processed_pseudogene
0,9709611	PLEKHM1	protein_coding
0,7740102	RPS15AP17	processed_pseudogene
0,7980625	JPX	lncRNA

0,9068107	NUTM2B-AS1	lncRNA
0,9496064	MTND2P28	unprocessed_pseudogene
0,6564675	SBDSP1	transcribed_unprocessed_pseudogene
0,5140003	HNRNPA1P35	processed_pseudogene
0,284683	FGD5-AS1	lncRNA
0,1951217	MEG8	lncRNA
0,9312186	AL080250.1	lncRNA
0,6491869	ERCC6	protein_coding
0,4799252	NOL7	protein_coding
0,9700303	MTND1P23	unprocessed_pseudogene
0,9692156	AC113935.1	processed_pseudogene
0,166375	UQCRFS1P1	processed_pseudogene
0,8554227	AC009487.2	processed_pseudogene
0,73747	AL356124.1	lncRNA
0,9481776	RPL26P19	processed_pseudogene
0,993752	GTF2H2B	transcribed_unprocessed_pseudogene
0,6613295	NPY6R	transcribed_processed_pseudogene
0,5943942	CFLAR-AS1	lncRNA
0,9771336	AC016831.1	lncRNA
0,3089259	TPI1P1	processed_pseudogene
0,7611529	SLC16A1-AS1	lncRNA
0,8422763	TMEM185B	protein_coding
0,9358535	FTH1P20	processed_pseudogene
0,7235397	ENTPD1-AS1	lncRNA
0,276112	CUTALP	transcribed_unitary_pseudogene
0,9773277	PGAM4	protein_coding
0,9481776	LINC01359	lncRNA
0,6785825	DANCR	lncRNA
0,5062136	RHEBP2	processed_pseudogene
0,9766993	AL009174.1	processed_pseudogene
0,9991742	SLC8A1-AS1	lncRNA
0,9544158	WDR46	protein_coding
0,8647372	RPL41P1	processed_pseudogene
0,5245497	AC005912.1	processed_pseudogene
0,4894579	RPS28P7	processed_pseudogene
0,8185595	ZNF717	protein_coding
0,9424246	WASH7P	unprocessed_pseudogene
0,9166492	PARG	protein_coding
0,9551108	TP73-AS1	transcribed_unitary_pseudogene
0,7836389	AP001324.1	processed_pseudogene
0,8212084	AL390728.4	transcribed_unprocessed_pseudogene
0,3799892	RD3L	protein_coding
0,3355997	AC239809.3	lncRNA
0,3801685	AC138969.2	unprocessed_pseudogene
0,2332141	HCG11	lncRNA
0,8348267	MT-ATP8	protein_coding
0,9551148	AL669831.1	transcribed_unprocessed_pseudogene
0,9700303	AP006222.1	transcribed_processed_pseudogene
0,8436853	OST4	protein_coding
0,5445815	EEF1A1P11	processed_pseudogene
0,9998478	AC005000.1	processed_pseudogene
0,8183781	SPCS2P4	processed_pseudogene
0,7558125	CES1P1	transcribed_unprocessed_pseudogene
0,4521055	ARL17B	protein_coding
0,9010513	DHFR	protein_coding
0,7296863	LINC01128	lncRNA
0,9705617	RPS13P2	processed_pseudogene
0,8155634	RPL41	protein_coding
0,9874145	AC026403.1	processed_pseudogene

0,8495694	AC006001.3	transcribed_unprocessed_pseudogene
0,993752	TTTY10	lncRNA
0,8597029	LINC00710	lncRNA
0,7086114	MTCO2P12	unprocessed_pseudogene
0,8992483	DPY19L1P1	unprocessed_pseudogene
0,8185595	RALGAPA1P1	processed_pseudogene
0,9994264	AC092155.2	processed_pseudogene
0,997931	RPL21P93	processed_pseudogene
0,9794449	MBNL1-AS1	lncRNA
0,9086283	RPL4P4	processed_pseudogene
0,6371005	NAMPTP1	processed_pseudogene
0,7149965	AL589880.1	processed_pseudogene
0,8025062	EIF4EP2	processed_pseudogene
0,6762366	RPL10P6	processed_pseudogene
0,8698319	AC006059.1	lncRNA
0,6903585	ACBD6	protein_coding
0,9678404	AL450405.1	processed_pseudogene
0,9839612	FTH1P5	processed_pseudogene
0,855954	RPL4P5	processed_pseudogene
0,8197846	AC078817.1	processed_pseudogene
0,7740102	GOLGA6L5P	transcribed_unprocessed_pseudogene
0,9730551	AC009245.1	processed_pseudogene
0,9900815	TCEA1P2	processed_pseudogene
0,9921186	AC021078.1	lncRNA
0,9731251	FTX	lncRNA
0,2737133	AC092683.1	lncRNA
0,7984578	RPS23P8	processed_pseudogene
0,9666028	DNM3OS	lncRNA
0,0125259	LINC02541	lncRNA
0,3450085	HSBP1	protein_coding
0,998169	RPL7P32	processed_pseudogene
0,9700303	HCG18	lncRNA
0,3428257	VDAC1P6	processed_pseudogene
0,931978	WARS2-AS1	lncRNA
0,077831	HLA-DPA1	protein_coding
0,8221205	RPS18	protein_coding
0,8387664	DLEU2	lncRNA
0,7177328	LINC-PINT	lncRNA
0,8213028	AC079922.1	processed_pseudogene
0,7607016	AL159166.1	lncRNA
0,7820981	TAPBP	protein_coding
0,7740102	ANXA2P2	processed_pseudogene
0,673546	TMA7	protein_coding
0,3435112	MCTS1	protein_coding
0,9998478	FTH1P7	processed_pseudogene
0,3278973	AL078590.2	lncRNA
0,7451695	RPS3AP25	processed_pseudogene
0,4887663	SMIM26	protein_coding
0,5679388	RPL3P4	processed_pseudogene
0,7968733	KANTR	protein_coding
0,6383874	GOLGA8N	protein_coding
0,5996432	LINC00342	lncRNA
0,8575378	NPIPA9	protein_coding
0,4954628	AC097523.1	processed_pseudogene
0,6244656	EEF1A1P14	processed_pseudogene
0,466638	LINC00702	lncRNA
0,6475905	AL161457.2	lncRNA
0,7546576	LINC00472	lncRNA
0,9848567	RPL21P134	processed_pseudogene

0,7972313	GPX1	protein_coding
0,5117223	GTF2IP4	transcribed_processed_pseudogene
0,9067454	LINC01719	lncRNA
0,4835164	EIF3FP3	processed_pseudogene
0,4919058	EEF1A1P6	processed_pseudogene
0,6613295	LINC00513	lncRNA
0,8364227	AC098617.1	lncRNA
0,6244656	RPL10P9	processed_pseudogene
0,997931	RPS28	protein_coding
0,3503253	UQCRHL	protein_coding
0,8373546	TRIM26	protein_coding
0,633922	AP000936.3	processed_pseudogene
0,5777776	AC099560.2	processed_pseudogene
0,7467854	AC009264.1	lncRNA
0,9538485	RPS26P47	processed_pseudogene
0,8133591	ZNF37BP	transcribed_processed_pseudogene
0,9333994	ZNF736	protein_coding
0,9374738	MAGI2-AS3	lncRNA
0,8176511	RPL13AP20	processed_pseudogene
0,3098192	AC018464.1	lncRNA
0,8500846	FAM133B	protein_coding
0,9771336	CCT6P3	transcribed_unprocessed_pseudogene
0,90504	JRK	protein_coding
0,8640757	RPSAP9	processed_pseudogene
0,5074563	AL162151.2	processed_pseudogene
0,9484395	SDCBP2-AS1	lncRNA
0,2337466	GAS5	lncRNA
0,8992483	AC144530.1	processed_pseudogene
0,673546	HLA-B	protein_coding
0,7102291	RPS3AP6	processed_pseudogene
0,9248086	RPL23AP42	processed_pseudogene
0,0386353	FABP5P7	processed_pseudogene
0,9934381	FTH1P2	processed_pseudogene
0,6475905	MLIP-AS1	lncRNA
0,5600133	ZSCAN31	protein_coding
0,7934662	LINC02248	lncRNA
0,7972313	RPL39P3	processed_pseudogene
0,9944531	DANT2	lncRNA
0,9934576	ITGA9-AS1	lncRNA
0,6206631	DPY19L2P4	transcribed_unprocessed_pseudogene
0,5943942	LINC01278	lncRNA
0,9312186	RPS2P7	processed_pseudogene
0,9840915	TRDN-AS1	lncRNA
0,9479329	RPL6P27	transcribed_processed_pseudogene
0,7433064	AL356599.1	lncRNA
0,888621	H3F3AP4	processed_pseudogene
0,4894579	LINC00894	lncRNA
0,2443993	KIAA0040	protein_coding
0,8889668	TTC28-AS1	lncRNA
0,8854446	COX10-AS1	lncRNA
0,3694563	ZBED5	protein_coding
0,9238608	VLDLR-AS1	lncRNA
0,8711676	FAM157A	transcribed_unprocessed_pseudogene
0,645515	AC099336.2	processed_pseudogene
0,9998538	H3F3BP1	processed_pseudogene
0,6835435	RPL13AP5	processed_pseudogene
0,5462026	STARD13-AS	lncRNA
0,8992483	BCYRN1	scRNA
0,4498511	TMSB4XP1	processed_pseudogene

0,8212084	AL732372.2	transcribed_unprocessed_pseudogene
0,9976952	HAND2-AS1	lncRNA
0,4148453	NR2F1-AS1	lncRNA
0,7258405	CDKN2AIPNL	protein_coding
0,2814709	BMS1P10	transcribed_unprocessed_pseudogene
0,7673209	SMG1P1	transcribed_unprocessed_pseudogene
0,7952381	TTN-AS1	lncRNA
0,7065438	ZNF737	protein_coding
0,9933219	RGL2	protein_coding
0,4894579	LINC01409	lncRNA
0,9951314	RPSAP15	processed_pseudogene
0,7609122	AC243919.1	transcribed_processed_pseudogene
0,2945575	AL138828.1	lncRNA
0,2318378	UQCRBP1	processed_pseudogene
0,9360637	FAM200B	protein_coding
0,8703454	MTCO1P12	unprocessed_pseudogene
0,7740102	FLG-AS1	lncRNA
0,9994264	PTENP1	transcribed_processed_pseudogene
0,8229361	CELF2-AS2	lncRNA
0,5123978	LRRC37A2	protein_coding
0,7278987	RPL9P7	processed_pseudogene
0,9994264	SCARNA7	scaRNA
0,5111636	AC008026.1	processed_pseudogene
0,8117718	RNF103	protein_coding
0,8833312	RBM14	protein_coding
0,9700639	AC011979.1	processed_pseudogene
0,2430128	KLHL41	protein_coding
0,9703483	AL157392.3	lncRNA
0,9781971	RPL21P39	processed_pseudogene
0,81098	PARGP1	transcribed_unprocessed_pseudogene
0,721162	RN7SL674P	misc_RNA
0,9808899	AC104563.1	processed_pseudogene
0,8928206	AMY2B	protein_coding
0,8695843	RPSAP12	processed_pseudogene
0,7912354	PCDHGC3	protein_coding
0,9450135	ZNF542P	transcribed_unprocessed_pseudogene
0,8720083	RPS2P5	processed_pseudogene
0,9367988	PPIL3	protein_coding
0,654521	AC010343.1	processed_pseudogene
0,8704958	FOXO3B	protein_coding
0,9865636	AQP1	protein_coding
0,9968825	AC106872.2	processed_pseudogene
0,2164941	RDH14	protein_coding
0,7954765	RN7SL128P	misc_RNA
0,42916	RPL15P2	processed_pseudogene
0,9668896	NSUN6	protein_coding
0,0998266	LINC00881	lncRNA
0,5740917	ADAMTS9-AS1	lncRNA
0,9767278	CRCP	protein_coding
0,6219816	SUCLG2-AS1	lncRNA
0,855954	CD302	protein_coding
0,6305527	EEF1A1P25	processed_pseudogene
0,8632351	RPL7P19	processed_pseudogene
0,2127154	ATP5MF	protein_coding
0,4954628	PSMC1P1	processed_pseudogene
0,5175488	GUSBP2	transcribed_unprocessed_pseudogene
0,8834615	ARPC4	protein_coding
0,9345529	ZBTB20-AS1	lncRNA
0,9917279	AC114728.1	processed_pseudogene

0,138108	INMT	protein_coding
0,4810647	ADAMTS9-AS2	lncRNA
0,9360637	ARPC1A	protein_coding
0,1326966	ATP5PO	protein_coding
0,9533676	PLEKHO2	protein_coding
0,7154026	AL627309.5	lncRNA
0,9484395	PISD	protein_coding
0,9621537	PI4KA	protein_coding
0,4585504	RPL7AP6	processed_pseudogene
0,6604792	MUC20-OT1	lncRNA
0,7576959	CRYZL2P	transcribed_unitary_pseudogene
0,4835164	ARFGAP3	protein_coding
0,9803555	C22orf39	protein_coding
0,9374881	AC073861.1	processed_pseudogene
0,8545769	EIF6	protein_coding
0,3280381	MRPL20	protein_coding
0,6216638	ARPIN	protein_coding
0,9768456	RPL21P11	processed_pseudogene
0,624851	LINC00882	lncRNA
0,993752	AC107032.1	processed_pseudogene
0,1131434	MRPL33	protein_coding
0,8003414	MICAL3	protein_coding
0,9312186	AC087343.1	processed_pseudogene
0,9544158	AC115223.1	processed_pseudogene
0,9813581	STON1	protein_coding
0,8441592	STMP1	protein_coding
0,7563025	KCTD7	protein_coding
0,4697514	DUBR	lncRNA
0,855954	NPIPB5	protein_coding
0,305334	MRPS6	protein_coding
0,9540553	ZNF512	protein_coding
0,8481719	NFS1	protein_coding
0,9860501	AC093591.1	processed_pseudogene
0,9781148	DDOST	protein_coding
0,8786805	AC110749.1	processed_pseudogene
0,8791163	AC004889.1	lncRNA
0,7842247	AC133134.1	processed_pseudogene
0,6883842	AC024293.1	processed_pseudogene
0,9900268	RPL7P23	processed_pseudogene
0,9832186	AC116533.1	processed_pseudogene
0,1225394	ETV5	protein_coding
0,9377639	RBM12	protein_coding
0,9976739	APOBEC3C	protein_coding
0,6610925	RN7SL396P	misc_RNA
0,4835164	FCGR2C	polymorphic_pseudogene
0,9270004	BX679664.3	processed_pseudogene
0,5267449	C4A	protein_coding
0,1500522	HBB	protein_coding
0,8769713	N4BP2L2	protein_coding
0,7912354	GABPB1-AS1	lncRNA
0,4999985	EEF1A1P4	processed_pseudogene
0,59951	NEAT1	lncRNA
0,284683	LINC02211	lncRNA
0,8062191	ZNF585B	protein_coding
0,358645	SNHG6	lncRNA
0,5879554	LINC01184	lncRNA
0,7954765	AC093752.1	transcribed_unprocessed_pseudogene
0,2841212	RAB30-DT	lncRNA
0,9665816	RASSF8-AS1	lncRNA

0,9476644	H2AFJ	protein_coding
0,6591549	STARD4-AS1	lncRNA
0,7972313	PGAM5	protein_coding
0,3454701	MIR4458HG	lncRNA
0,933129	OIP5-AS1	lncRNA
0,6403073	CKMT2-AS1	lncRNA
0,9296784	MTND4P12	processed_pseudogene
0,7395968	NR2F2-AS1	lncRNA
0,5364552	TMEM161B-AS1	lncRNA
0,9364287	FAM13A-AS1	lncRNA
0,6785825	NNT-AS1	lncRNA
0,8786805	RRN3P1	transcribed_unprocessed_pseudogene
0,7992396	ADH1C	protein_coding
0,6463814	AC093879.1	lncRNA
0,9781971	TNXA	unprocessed_pseudogene
0,504498	LUCAT1	lncRNA
0,8850069	CDK11B	protein_coding
0,8326738	WHAMMP2	transcribed_unprocessed_pseudogene
0,7125689	POU5F2	protein_coding
0,9917279	MTATP6P1	unprocessed_pseudogene
0,9165105	AC046134.2	lncRNA
0,7355147	TMEM150C	protein_coding
0,7057791	EEF1A1P9	processed_pseudogene
0,8745367	NPM1P27	processed_pseudogene
0,8971058	NAIP	protein_coding
0,2332141	CARMN	lncRNA
0,6593174	AC011330.1	unprocessed_pseudogene
0,3881918	EEF1A1P19	processed_pseudogene
0,1052063	AC087457.1	lncRNA
0,7395639	AC106791.1	lncRNA
0,5248895	EEF1A1P13	processed_pseudogene
0,1356666	LINC02762	lncRNA
0,1835405	TRMT9B	protein_coding
0,9368504	ZNF718	protein_coding
0,4430813	SMIM20	protein_coding
0,5170218	CHCHD10	protein_coding
0,4161706	AC007126.1	lncRNA
0,7558125	SELENOP	protein_coding
0,4748134	LINC02208	lncRNA
0,9082846	GMDS-DT	lncRNA
0,9296784	THAP9-AS1	lncRNA
0,7673209	AC087854.1	lncRNA
0,8823913	AF117829.1	lncRNA
0,9479329	AC131392.2	unprocessed_pseudogene
0,5531846	SHANK3	protein_coding
0,9618453	ZNF550	protein_coding
0,7842247	MALAT1	lncRNA
0,7940001	AC104825.1	lncRNA
0,768388	AC145138.1	unprocessed_pseudogene
0,3772742	SCARNA6	scaRNA
0,3616153	SCARNA5	scaRNA
0,3697716	RNY4	misc_RNA
0,512885	SCARNA21	scaRNA
0,5847346	GUSBP3	transcribed_unprocessed_pseudogene
0,3894807	C8orf88	protein_coding
0,7025012	CCDC71L	protein_coding
0,9343701	AC092828.1	lncRNA
0,7956173	PCDHGB6	protein_coding
0,7118106	TUG1	protein_coding

0,2915153	AC139272.1	unprocessed_pseudogene
0,1996599	TRNP1	protein_coding
0,8079393	ALG11	protein_coding
0,7687718	ATXN7L3B	protein_coding
0,6285359	PRKDC	protein_coding
0,888621	PCDHGA6	protein_coding
0,5567171	OTUD6B-AS1	lncRNA
0,4894579	AC138866.1	unprocessed_pseudogene
0,7688687	PCDHGA10	protein_coding
0,8185595	ZNF260	protein_coding
0,9439974	LYN	protein_coding
0,998169	PCDHGB7	protein_coding
0,8381234	NPIPB11	protein_coding
0,2656432	AC112191.2	processed_pseudogene
0,9464798	SMG1P6	unprocessed_pseudogene
0,9066034	WAC-AS1	lncRNA
0,9510167	FPGT	protein_coding
0,2737133	AC138866.2	unprocessed_pseudogene
0,5590581	GVINP1	transcribed_unprocessed_pseudogene
0,8721281	NPIPA2	protein_coding
0,921128	BRK1	protein_coding
0,7385573	CHMP1B	protein_coding
0,6403073	MIR100HG	lncRNA
0,7558125	EID1	protein_coding
0,5267449	TBX5-AS1	lncRNA
0,8829558	POLR2M	protein_coding
0,7988401	AC084357.3	transcribed_unprocessed_pseudogene
0,6238595	SNHG1	lncRNA
0,8492407	MTRNR2L8	protein_coding
0,7558125	CACNA1C-AS4	lncRNA
0,2137007	CTSO	protein_coding
0,3004311	COA8	protein_coding
0,9269165	ZNF432	protein_coding
0,9738194	ZNF10	protein_coding
0,9902795	SMIM3	protein_coding
0,8873508	RPL41P2	processed_pseudogene
0,8176511	RPL41P5	processed_pseudogene
0,5015091	SMIM10L1	protein_coding
0,9194118	ZNF350	protein_coding
0,7146386	PLBD1-AS1	lncRNA
0,8259599	ZNF253	protein_coding
0,3309793	HP	protein_coding
0,993752	AC010186.3	lncRNA
0,9907262	DENND11	protein_coding
0,4321437	LSM14A	protein_coding
0,6854132	AC009318.1	lncRNA
0,9914435	GATC	protein_coding
0,5402971	ZNF271P	transcribed_unitary_pseudogene
0,4425571	AC048341.1	lncRNA
0,0736295	OVCH1-AS1	lncRNA
0,784306	PSMA3-AS1	lncRNA
0,6473313	CUX1	protein_coding
0,9234217	CHURC1	protein_coding
0,6651002	LINC00641	lncRNA
0,9438763	HIF1A-AS3	lncRNA
0,7821595	CEP95	protein_coding
0,654521	FPGT-TNNI3K	protein_coding
0,8875885	CSPG4P12	transcribed_unprocessed_pseudogene
0,7662231	AF111167.2	lncRNA

0,4684539	INAFM2	protein_coding
0,949756	HMGB1P6	processed_pseudogene
0,7182975	AC083843.3	lncRNA
0,6727601	RBM15B	protein_coding
0,1216048	AC107068.1	lncRNA
0,1356666	NORAD	lncRNA
0,9772493	NSFP1	unprocessed_pseudogene
0,4827579	AC104083.1	lncRNA
0,1729437	MRC1	protein_coding
0,7842706	FAM157C	lncRNA
0,9544158	ERVK13-1	lncRNA
0,8578793	ANKRD20A1	protein_coding
0,9738194	CCPG1	protein_coding
0,7639764	TMEM178B	protein_coding
0,8922683	LINC01355	lncRNA
0,6184746	PECAM1	protein_coding
0,0640162	PDCD6IPP2	transcribed_unprocessed_pseudogene
0,817424	AC233699.1	unprocessed_pseudogene
0,9296784	AL137782.1	lncRNA
0,8316952	SMG1P7	transcribed_unprocessed_pseudogene
0,5537742	GAN	protein_coding
0,2419676	HPR	protein_coding
0,9808899	AC007406.5	lncRNA
0,7258655	LINC00662	lncRNA
0,4161706	SNORD3B-2	snoRNA
0,4521055	SPON1	protein_coding
0,8322139	AC005670.3	lncRNA
0,633922	MTCO1P40	processed_pseudogene
0,6023346	GTF2I	protein_coding
0,9914435	ZNF234	protein_coding
0,5879554	ROCK1P1	transcribed_unprocessed_pseudogene
0,3715255	MYZAP	protein_coding
0,9802452	RPS7P1	processed_pseudogene
0,1904085	SRSF8	protein_coding
0,9248086	UBBP4	transcribed_unprocessed_pseudogene
0,7182975	RN7SL4P	misc_RNA
0,5768067	LINC00667	lncRNA
0,5170218	SNORD3A	snoRNA
0,6903585	NBPF11	protein_coding
0,8318664	AC015813.1	lncRNA
0,4662823	LINC00909	lncRNA
0,8258095	AC016596.3	processed_pseudogene
0,6828036	NOTCH2NLA	protein_coding
0,6574161	DYNLL2	protein_coding
0,5964345	OTUD7B	protein_coding
0,7242009	SUZ12P1	transcribed_unprocessed_pseudogene
0,4890796	SNORD3C	snoRNA
0,6444859	GJA5	protein_coding
0,4266067	MIR133A1HG	lncRNA
0,5170218	SNORD3B-1	snoRNA
0,9067454	RBM8A	protein_coding
0,1140347	TIMM23	protein_coding
0,54642	RNF115	protein_coding
0,7024923	RN7SL5P	misc_RNA
0,8189129	SEC22B	protein_coding
0,9476644	FSBP	protein_coding
0,768388	TXNIP	protein_coding
0,9998478	MIR3609	miRNA
0,7921707	SRGAP2	protein_coding

0,6254273	RASSF5	protein_coding
0,874513	STRADA	protein_coding
0,5688668	NBPF15	protein_coding
0,3881918	NCOA4	protein_coding
0,3286712	MRPS21	protein_coding
0,7816527	MYO15B	protein_coding
0,9954421	AC060780.1	lncRNA
0,4018815	LRRC37A16P	transcribed_unprocessed_pseudogene
0,8575378	ZNF850	protein_coding
0,4413743	AC020916.1	lncRNA
0,7199013	MTCO2P2	processed_pseudogene
0,9424246	ZNF224	protein_coding
0,9802452	AC010680.1	lncRNA
0,7771859	NBPF12	protein_coding
0,7755905	AC005261.1	lncRNA
0,5360859	FAM156A	protein_coding
0,5851976	AC092279.1	lncRNA
0,8258207	AC092070.2	transcribed_unprocessed_pseudogene
0,654521	MTRNR2L12	protein_coding
0,7385573	ZNF728	protein_coding
0,7182975	ZSCAN16-AS1	lncRNA
0,9729242	ZNF587B	protein_coding
0,9884562	AC022149.1	processed_pseudogene
0,9861632	TMEM185A	protein_coding
0,5869808	NBPF9	protein_coding
0,7713242	KCNQ1OT1	lncRNA
0,7025012	SNHG8	lncRNA
0,8222645	AL158152.1	lncRNA
0,8865445	BMS1P7	transcribed_unprocessed_pseudogene
0,6337897	NBPF8	transcribed_unprocessed_pseudogene
0,5491629	NBPF14	protein_coding
0,933129	TAF15	protein_coding
0,7062909	SRGAP2D	unprocessed_pseudogene
0,9112805	NUDT4P2	processed_pseudogene
0,8682799	AC016831.5	lncRNA
0,4680251	AC240274.1	protein_coding
0,7433064	7SK	misc_RNA
0,7673209	AC010680.5	lncRNA
0,4673249	NBPF10	protein_coding
0,9829971	Z83843.1	lncRNA
0,2945575	LIX1L	protein_coding
0,9628671	LINC01954	lncRNA
0,768388	GTF2H5	protein_coding
0,5688668	RNU6-6P	snRNA
0,4894579	NBPF25P	transcribed_unprocessed_pseudogene
0,6745473	CASC15	lncRNA
0,8578793	AL021368.2	lncRNA
0,9817008	AC093297.2	lncRNA
0,8941532	POM121C	protein_coding
0,9808899	LINC01145	transcribed_unprocessed_pseudogene
0,7844757	ZNF595	protein_coding
0,7740102	DCP1A	protein_coding
0,7025012	LINC01578	lncRNA
0,8674075	AC008124.1	lncRNA
0,9221671	FAM106A	lncRNA
0,7740102	LINC02035	lncRNA
0,5911872	NBPF26	protein_coding
0,806856	AC058791.1	lncRNA
0,9112805	CWC25	protein_coding

0,5985056	SMIM11B	protein_coding
0,8966801	ZNHIT3	protein_coding
0,7472452	AC020904.2	transcribed_processed_pseudogene
0,9385376	AL592183.1	protein_coding
0,7321942	CYFIP1	protein_coding
0,6999614	U1	snRNA
0,5511615	TAF9	protein_coding
0,658029	CASTOR2	protein_coding
0,6663173	SOCS7	protein_coding
0,7743102	AC245297.3	lncRNA
0,5742684	CU633967.1	lncRNA
0,5943942	ZNF658	protein_coding
0,9237476	AC244154.1	transcribed_unprocessed_pseudogene
0,943695	RCC1L	protein_coding
0,9933219	AC233968.1	unprocessed_pseudogene
0,5799813	AC244669.2	unprocessed_pseudogene
0,6918332	MLLT6	protein_coding
0,5799813	PPP4R3B	protein_coding
0,5647201	SYNRG	protein_coding
0,8887908	AC241952.1	transcribed_unprocessed_pseudogene
0,4314663	PDCD6IPP1	transcribed_unprocessed_pseudogene
0,6613295	U1	snRNA
0,8500846	AATF	protein_coding
0,9977992	ARHGAP23	protein_coding
0,9803555	TUBGCP5	protein_coding
0,6160654	U2AF1L5	protein_coding
0,1112135	SIK1B	protein_coding
0,9738194	DUSP14	protein_coding
0,5510556	CU633904.1	lncRNA
0,9635114	WHAMMP3	transcribed_unprocessed_pseudogene
0,73747	RN7SL1	misc_RNA
0,9132055	ANKRD20A3	protein_coding
0,9925374	TADA2A	protein_coding
0,7456311	FRG1HP	transcribed_unprocessed_pseudogene
0,6137496	PIP4K2B	protein_coding
0,3030424	CNTNAP3P2	unprocessed_pseudogene
0,3694563	HERC2P2	transcribed_unprocessed_pseudogene
0,673546	HIST1H4E	protein_coding
0,1527123	HYDIN2	transcribed_unprocessed_pseudogene
0,5552067	GTF2IP1	transcribed_unprocessed_pseudogene
0,5768067	CU634019.1	lncRNA
0,8062191	STAG3L2	transcribed_unprocessed_pseudogene
0,9003006	HIST1H4D	protein_coding
0,7087026	SEC22B4P	transcribed_unprocessed_pseudogene
0,4848227	MARCKS	protein_coding
0,1326966	AC159540.2	lncRNA
0,0002507	TRAC	TR_C_gene
0,59951	PGM5P2	transcribed_unprocessed_pseudogene
0,1916186	PSMB3	protein_coding
0,6818653	U1	snRNA
0,4761054	FP236241.1	lncRNA
0,6938931	DDX52	protein_coding
0,9376695	ZNF8	protein_coding
0,5511615	MYO19	protein_coding
0,9486004	GGNBP2	protein_coding
0,9332245	ZNF229	protein_coding
0,4372336	ACACA	protein_coding
0,3316892	AC005332.6	lncRNA
0,6762366	RN7SL3	misc_RNA

0,621558	MRPL45	protein_coding
0,9069375	CR381670.1	unprocessed_pseudogene
0,1352238	AC093909.6	TEC
0,8275847	AC003681.1	lncRNA
0,7154026	AC009951.4	TEC
0,997931	TSTD3	protein_coding
0,7384592	AL136164.3	TEC
0,9154352	Z99129.4	TEC
0,9536474	WASH9P	unprocessed_pseudogene
0,8824701	AC090498.1	processed_pseudogene
0,8992483	AC009948.4	TEC
0,9162577	AC131649.2	TEC
0,281424	AQP7P3	unprocessed_pseudogene
0,9682998	BCLAF1P2	processed_pseudogene
0,5119861	AL353763.2	TEC
0,9424246	PLAC4	lncRNA
0,7820618	AL662795.2	TEC
0,9505993	AC027290.2	TEC
0,9347995	AC093908.1	TEC
0,3459301	AP001528.2	TEC
0,9738194	AC000123.3	TEC
0,7835684	Z95331.1	lncRNA
0,7836389	AP000648.4	TEC
0,9893136	FP565260.6	protein_coding
0,3872426	FP236383.4	lncRNA
0,8834615	EIF1B-AS1	lncRNA
0,4628071	FP671120.6	lncRNA
0,7213265	N4BP2L2-IT2	lncRNA
0,633922	FP236383.5	lncRNA
0,806905	NPTN-IT1	lncRNA
0,7763012	AC007878.1	lncRNA
0,7154026	HELLPAR	lncRNA
0,5402971	FP671120.7	lncRNA
0,6244656	EBLN3P	lncRNA
0,624851	WASH5P	transcribed_processed_pseudogene
0,9794449	FRG1CP	transcribed_unprocessed_pseudogene
0,0283162	AC107068.2	lncRNA
0,6128477	AC008038.1	processed_pseudogene
0,9533049	AC006453.2	transcribed_unprocessed_pseudogene
0,7201269	MIR1244-2	miRNA
0,7834471	AC068587.4	lncRNA
0,9900268	HSPA14	protein_coding
0,9142106	AC006460.2	lncRNA
0,3883821	AL591543.1	lncRNA
0,8854446	GABPB1-IT1	lncRNA
0,6383874	POLR2J3	protein_coding
0,645515	BX890604.2	lncRNA
0,7376181	NIPBL-DT	lncRNA
0,933129	AC092353.2	lncRNA
0,5400662	AL136171.2	lncRNA
0,9998478	AC026273.1	transcribed_unprocessed_pseudogene
0,8111745	NOTCH2NLC	protein_coding
0,166273	AC119673.2	lncRNA
0,2354584	AC106845.1	lncRNA
0,6793248	AC009878.2	lncRNA
0,9792506	AC012459.1	lncRNA
0,9450135	AL670729.3	lncRNA
0,1712026	Z85996.3	lncRNA
0,5504165	AC097448.1	lncRNA

Supplementary Table S3. MED subunits alteration in DCM patients *vs* HS

Gene name	logFC	FC	p value
**MED1**	0.044	1.031	0.747
**MED4**	-0.155	-1.113	0.318
**MED6**	-0.023	-1.016	0.917
**MED7**	-0.254	-1.193	0.161
**MED8**	-0.164	-1.120	0.349
**MED9**	**-0.333**	**-1.260**	**0.031**
**MED12**	0.357	1.280	0.128
**MED13**	0.224	1.168	0.132
**MED13L**	0.008	1.006	0.960
**MED14**	0.103	1.074	0.361
**MED15**	0.033	1.023	0.860
**MED17**	0.044	1.031	0.852
**MED20**	-0.341	-1.267	0.063
**MED21**	-0.216	-1.161	0.258
**MED22**	0.107	1.077	0.608
**MED23**	0.001	1.001	0.994
**MED24**	0.050	1.035	0.774
**MED28**	0.097	1.070	0.515
**MED29**	0.234	1.176	0.149
**CDK8**	0.253	1.192	0.139

Supplementary Table S4. MED subunit organization

Module localization	Subunit
**Head**	MED6; MED8; MED11; MED17; MED18; MED19; MED20; MED22; MED28; MED29; MED30.
**Middle**	MED1; MED4; MED7; MED9; MED10; MED14; MED21; MED26; MED31.
**Tail**	MED2; MED3; MED5; MED15; MED16; MED23; MED24; MED25.
**CDK8-kinase**	MED12/MED12L*; MED13/MED13L*; CDK8/19; CYCC.

* These subunits appear to assemble in a mutually exclusive manner.

Tomtom

Motif Comparison Tool

For further information on how to interpret these results please access https://meme-suite.org/meme/doc/tomtom-output-format.html.
To get a copy of the MEME software please access https://meme-suite.org.

If you use Tomtom in your research, please cite the following paper:
Shobhit Gupta, JA Stamatoyannopolous, Timothy Bailey and William Stafford Noble, "Quantifying similarity between motifs", *Genome Biology*, **8**(2):R24, 2007. [full text]

Query Motifs | Target Databases | Matches | Settings | Program information | Results in TSV Format NEW | Results in XML Format

Query Motifs

Next Top

Database	ID	Alt. ID	Preview	Matches	List
query_motifs	1		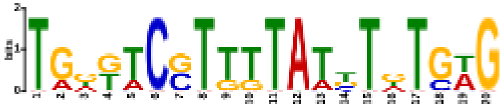	30	TCF7_HUMAN.H11MO.0.A, ZFP28_HUMAN.H11MO.0.C, CDX2_HUMAN.H11MO.0.A, GATA4_HUMAN.H11MO.0.A,

Target Databases

Previous Next Top

Database	Used	Matched
HOCOMOCOv11_core_HUMAN_mono_meme_format	401	30

Matches to 1

Previous Next Top

Summary | **Optimal Alignment**

**Name**	TCF7_HUMAN.H11MO.0.A
**Database**	HOCOMOCOv11_core_HUMAN_mono_meme_format
***p*-value**	1.03e-03
***E*-value**	4.12e-01
***q*-value**	4.75e-01

**Overlap**	16
**Offset**	-3
**Orientation**	Reverse Complement
	Show logo download options

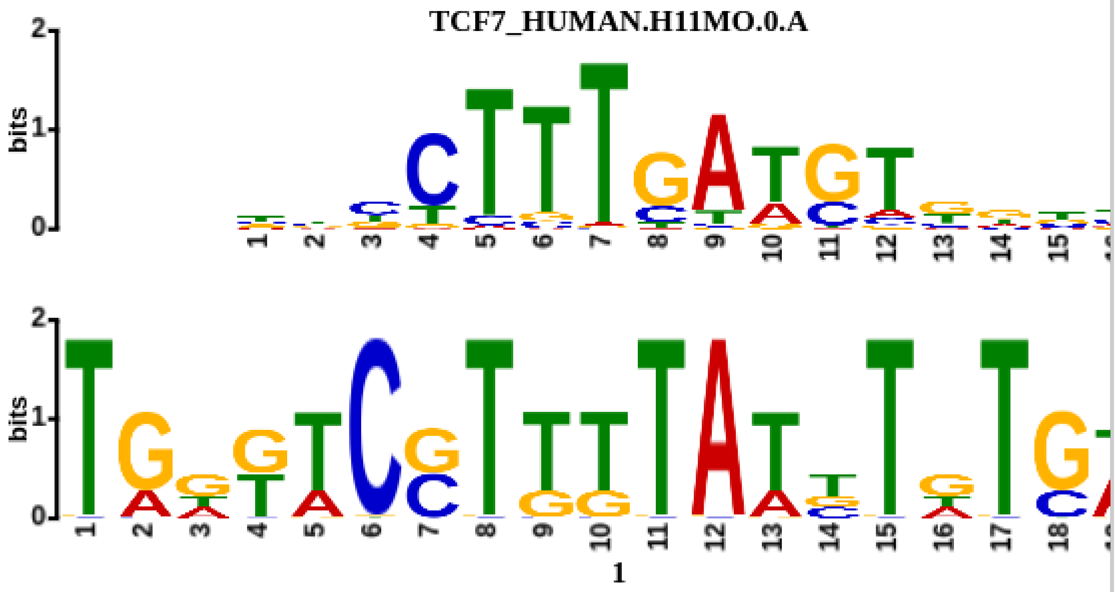

Summary

Optimal Alignment

**Name**	ZFP28_HUMAN.H11MO.0.C
**Database**	HOCOMOCOv11_core_HUMAN_mono_meme_format
***p*-value**	2.95e-03
***E*-value**	1.18e+00
***q*-value**	4.75e-01
**Overlap**	17
**Offset**	-3
**Orientation**	Normal
	Show logo download options

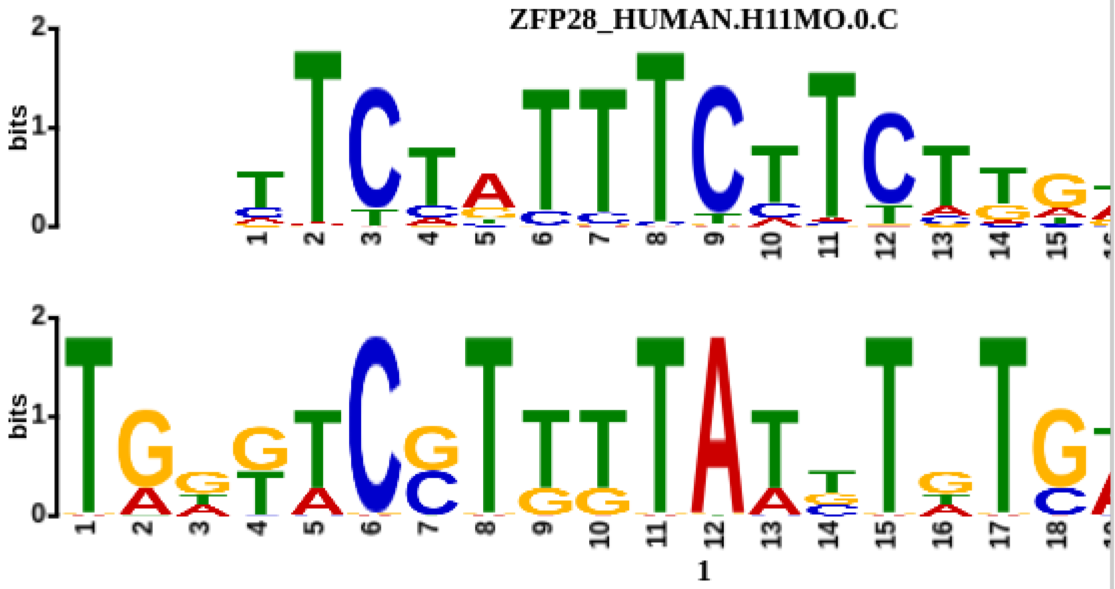

Summary

Optimal Alignment

**Name**	CDX2_HUMAN.H11MO.0.A

**Database**	HOCOMOCOv11_core_HUMAN_mono_meme_format
***p*-value**	3.48e-03
***E*-value**	1.40e+00
***q*-value**	4.75e-01
**Overlap**	12
**Offset**	-7
**Orientation**	Normal
	Show logo download options

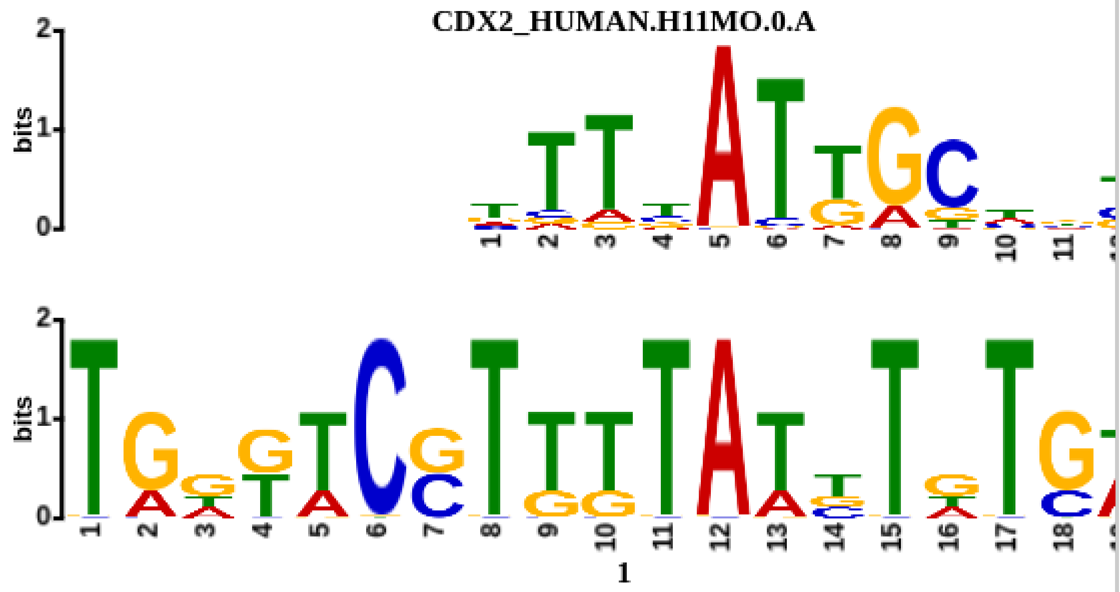

Summary

Optimal Alignment

**Name**	GATA4_HUMAN.H11MO.0.A
**Database**	HOCOMOCOv11_core_HUMAN_mono_meme_format
***p*-value**	3.51e-03
***E*-value**	1.41e+00
***q*-value**	4.75e-01
**Overlap**	10
**Offset**	-7
**Orientation**	Reverse Complement
	Show logo download options

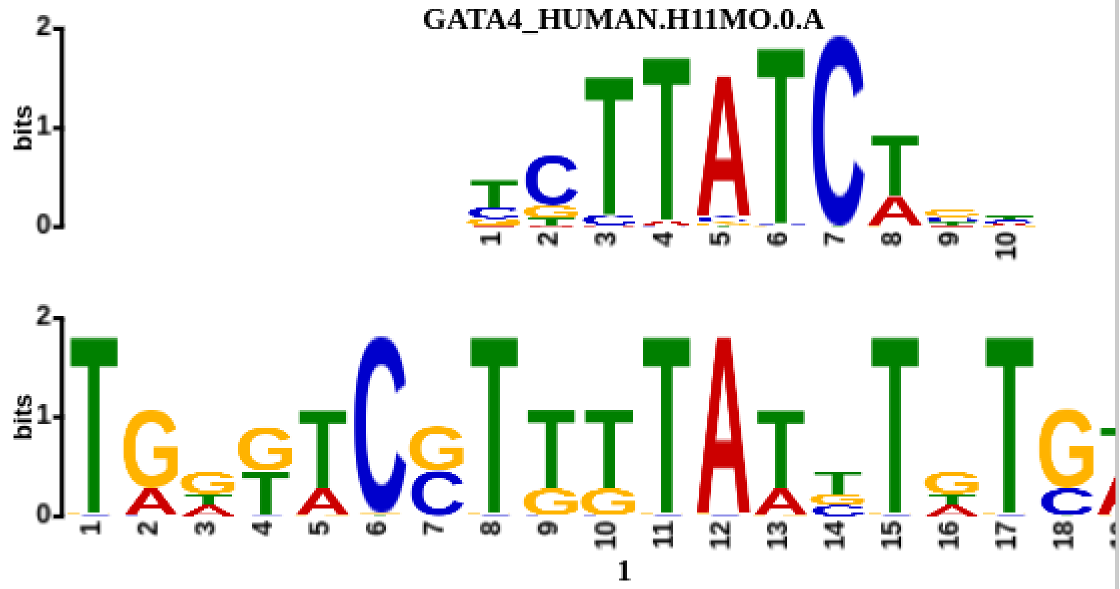

Summary

Optimal Alignment

**Name**	TF7L2_HUMAN.H11MO.0.A

**Database**	HOCOMOCOv11_core_HUMAN_mono_meme_format
***p*-value**	4.08e-03
***E*-value**	1.63e+00
***q*-value**	4.75e-01
**Overlap**	13
**Offset**	-4
**Orientation**	Normal

[Show logo download options]

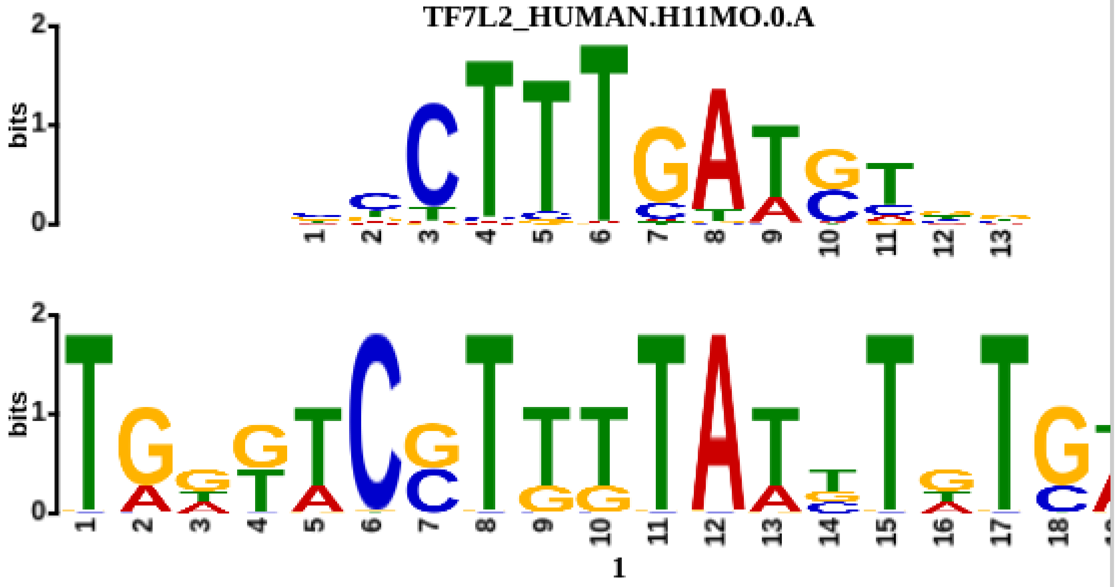

Summary

Optimal Alignment

**Name**	[ZIM3_HUMAN.H11MO.0.C]
**Database**	HOCOMOCOv11_core_HUMAN_mono_meme_format
***p*-value**	4.54e-03
***E*-value**	1.82e+00
***q*-value**	4.75e-01
**Overlap**	17
**Offset**	2
**Orientation**	Normal

[Show logo download options]

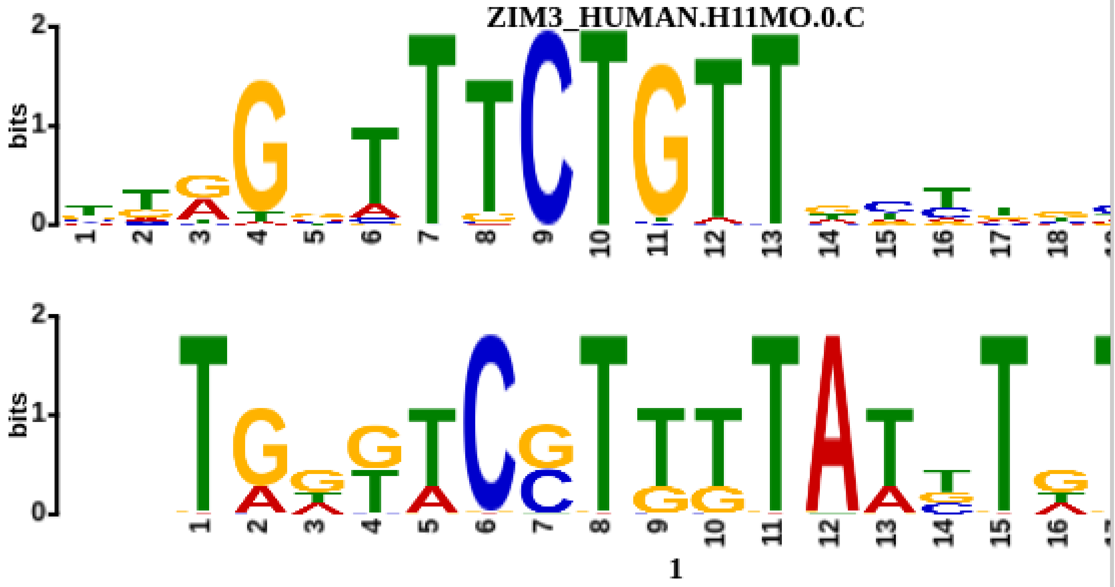

Summary

Optimal Alignment

**Name**	[ANDR_HUMAN.H11MO.0.A]

**Database**	HOCOMOCOv11_core_HUMAN_mono_meme_format
***p*-value**	4.88e-03
***E*-value**	1.96e+00
***q*-value**	4.75e-01
**Overlap**	18
**Offset**	0
**Orientation**	Normal

Show logo download options

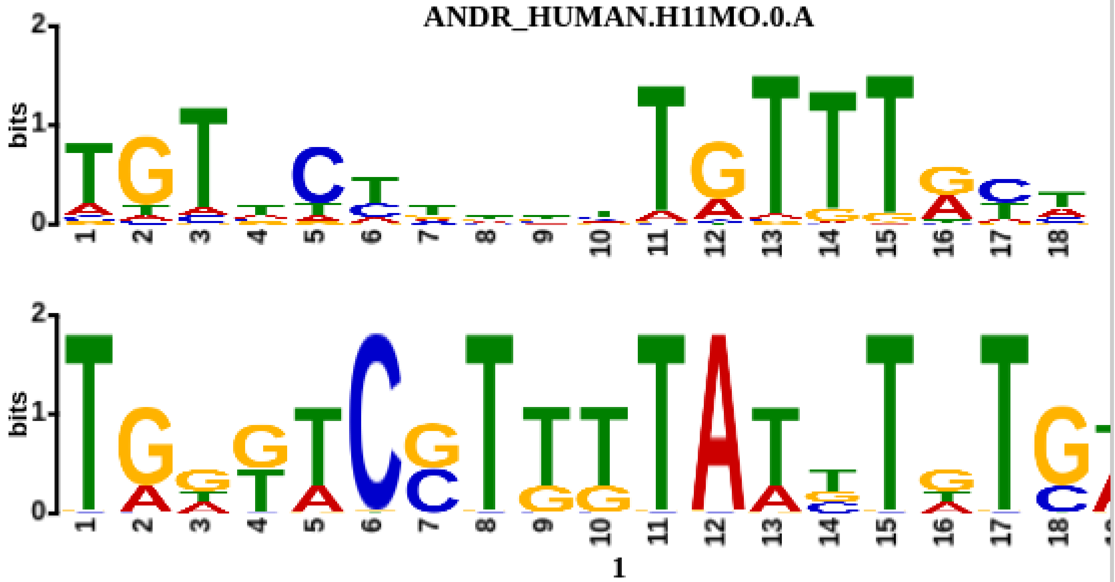

Summary

Optimal Alignment

**Name**	TF7L1_HUMAN.H11MO.0.B
**Database**	HOCOMOCOv11_core_HUMAN_mono_meme_format
***p*-value**	6.24e-03
***E*-value**	2.50e+00
***q*-value**	4.75e-01
**Overlap**	13
**Offset**	-3
**Orientation**	Normal

Show logo download options

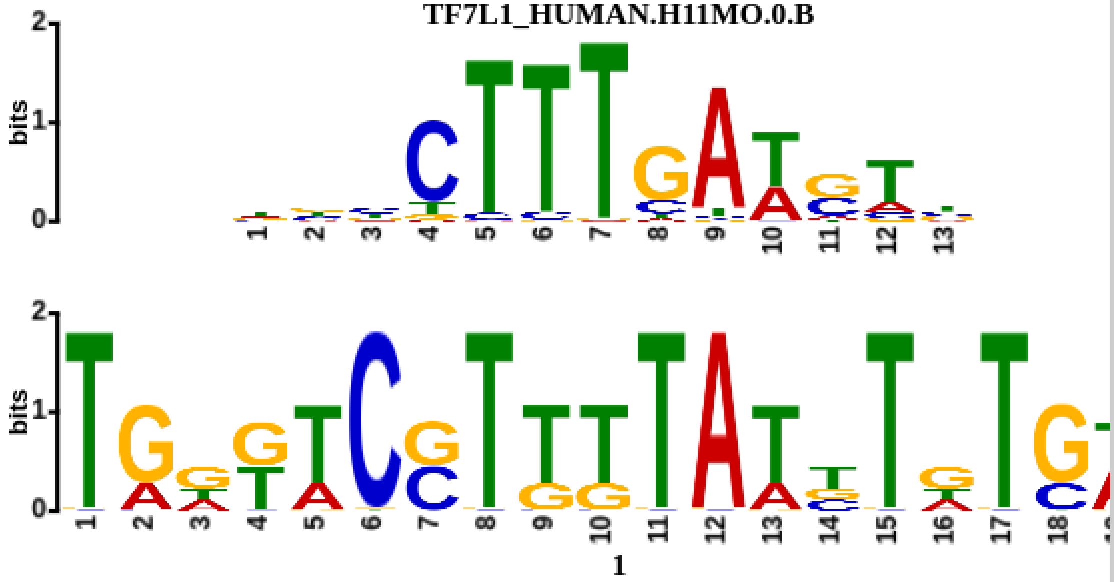

Summary

Optimal Alignment

**Name**	HXB13_HUMAN.H11MO.0.A

**Database**	HOCOMOCOv11_core_HUMAN_mono_meme_format
***p*-value**	6.25e-03
***E*-value**	2.51e+00
***q*-value**	4.75e-01
**Overlap**	10
**Offset**	-7
**Orientation**	Normal

Show logo download options

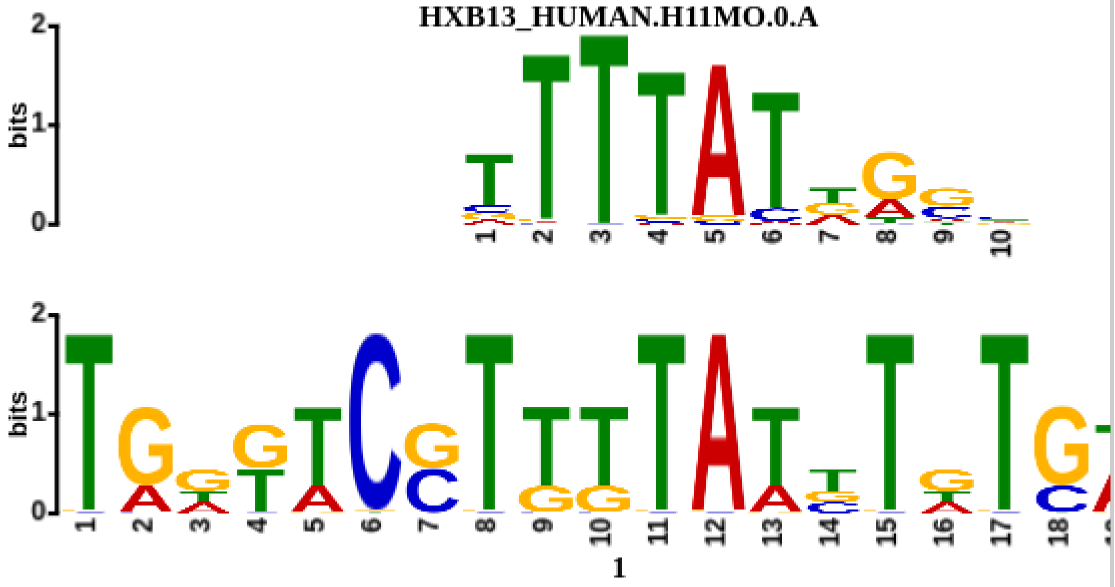

Summary

Optimal Alignment

**Name**	GATA3_HUMAN.H11MO.0.A
**Database**	HOCOMOCOv11_core_HUMAN_mono_meme_format
***p*-value**	6.83e-03
***E*-value**	2.74e+00
***q*-value**	4.75e-01
**Overlap**	11
**Offset**	-4
**Orientation**	Reverse Complement

Show logo download options

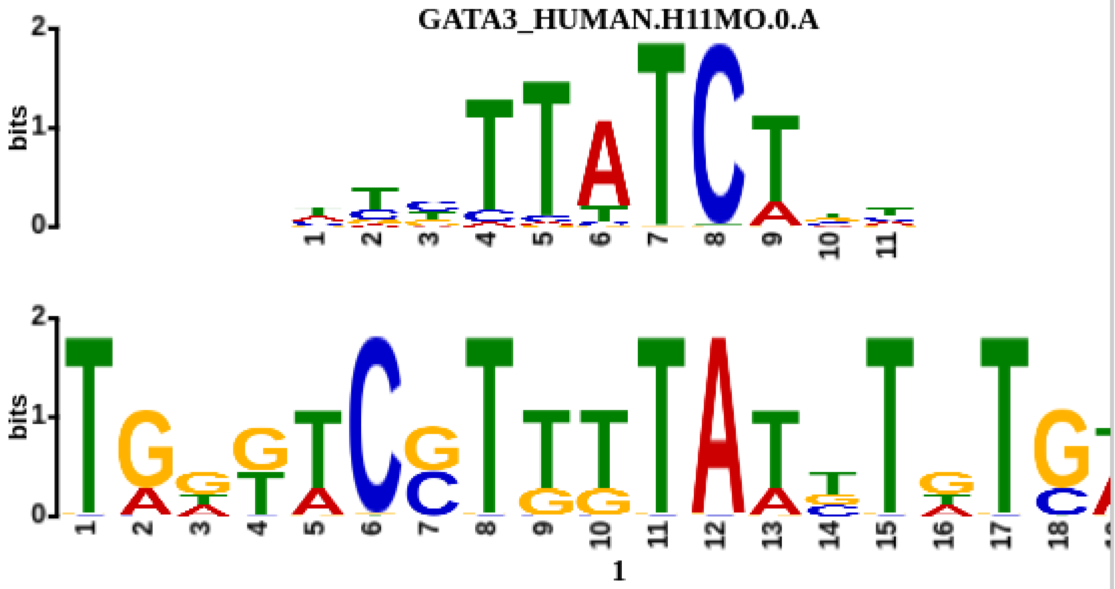

Summary

Optimal Alignment

**Name**	CDX1_HUMAN.H11MO.0.C

**Database**	HOCOMOCOv11_core_HUMAN_mono_meme_format
***p*-value**	7.11e-03
***E*-value**	2.85e+00
***q*-value**	4.75e-01
**Overlap**	8
**Offset**	-11
**Orientation**	Normal

Show logo download options

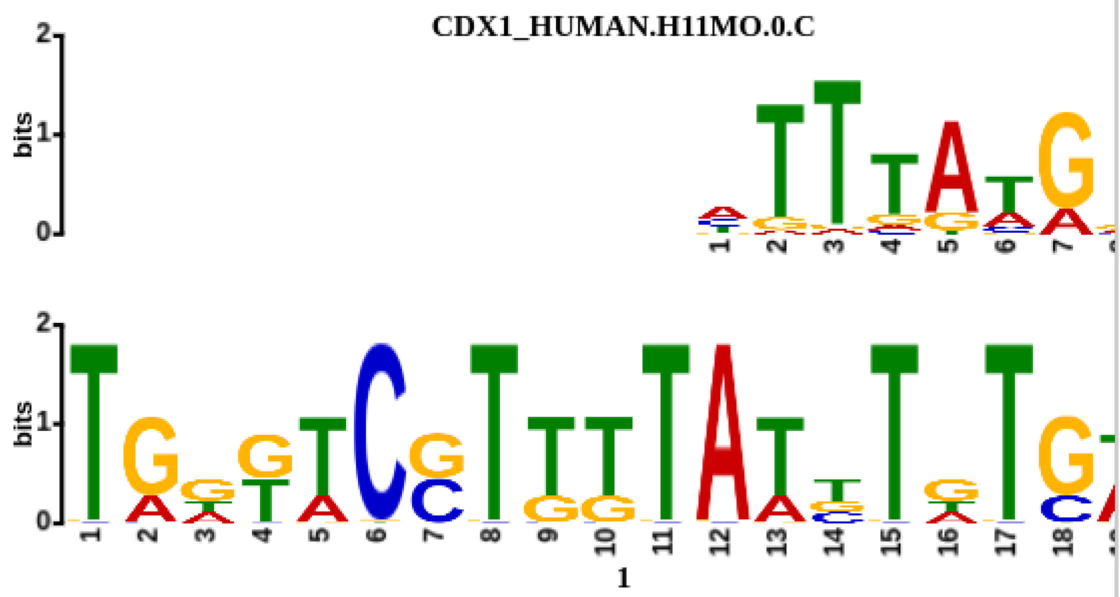

Summary

Optimal Alignment

**Name**	ZNF41_HUMAN.H11MO.0.C
**Database**	HOCOMOCOv11_core_HUMAN_mono_meme_format
***p*-value**	7.17e-03
***E*-value**	2.88e+00
***q*-value**	4.75e-01
**Overlap**	20
**Offset**	4
**Orientation**	Reverse Complement

Show logo download options

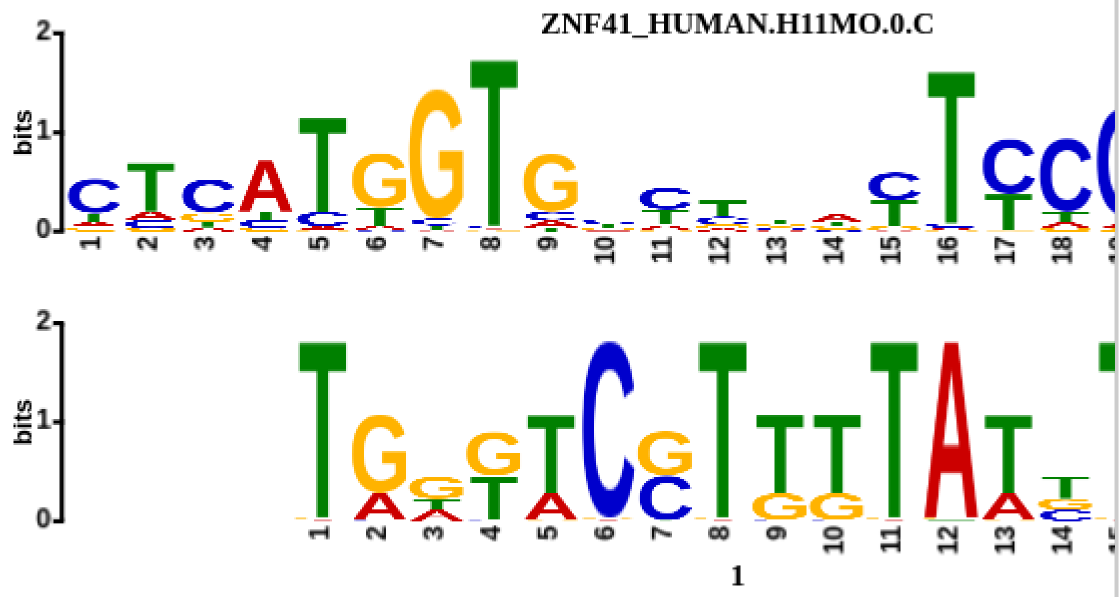

Summary

Optimal Alignment

**Name**	HXA10_HUMAN.H11MO.0.C

**Database**	HOCOMOCOv11_core_HUMAN_mono_meme_format
***p*-value**	9.57e-03
***E*-value**	3.84e+00
***q*-value**	5.84e-01
**Overlap**	12
**Offset**	-7
**Orientation**	Normal

[Show logo download options]

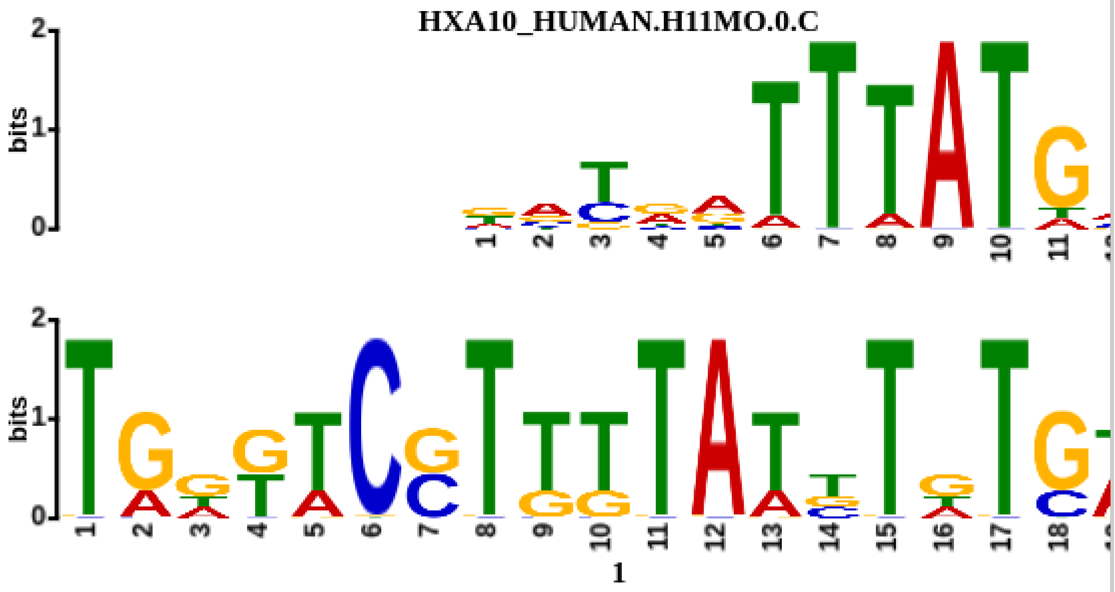

Summary

Optimal Alignment

**Name**	[HXA13_HUMAN.H11MO.0.C]
**Database**	HOCOMOCOv11_core_HUMAN_mono_meme_format
***p*-value**	1.07e-02
***E*-value**	4.29e+00
***q*-value**	5.85e-01
**Overlap**	11
**Offset**	-5
**Orientation**	Normal

[Show logo download options]

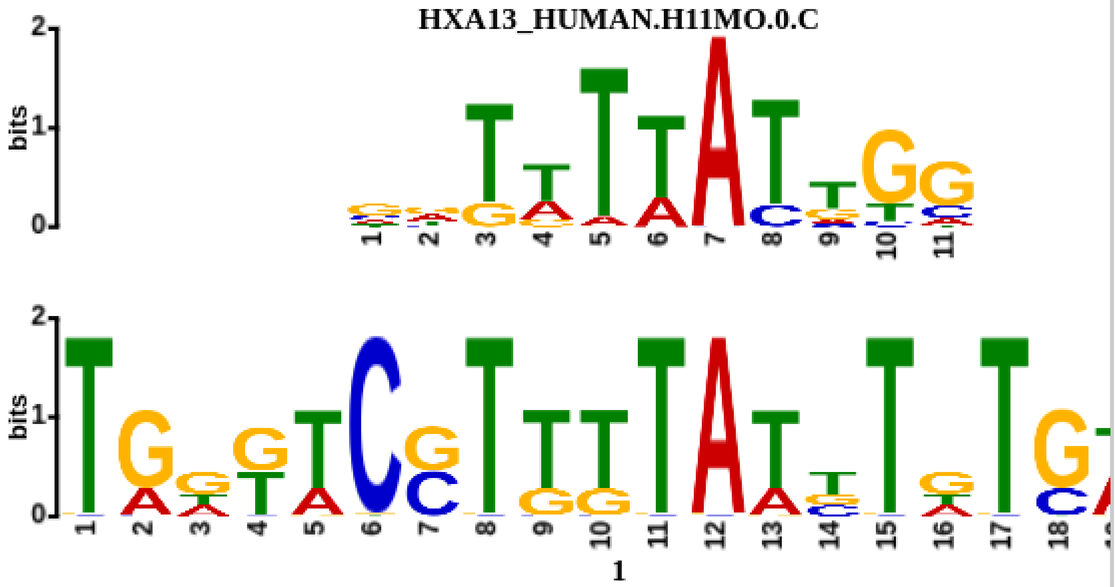

Summary

Optimal Alignment

**Name**	[ZN274_HUMAN.H11MO.0.A]

**Database**	HOCOMOCOv11_core_HUMAN_mono_meme_format
***p*-value**	1.14e-02
***E*-value**	4.58e+00
***q*-value**	5.85e-01
**Overlap**	18
**Offset**	2
**Orientation**	Reverse Complement

Show logo download options

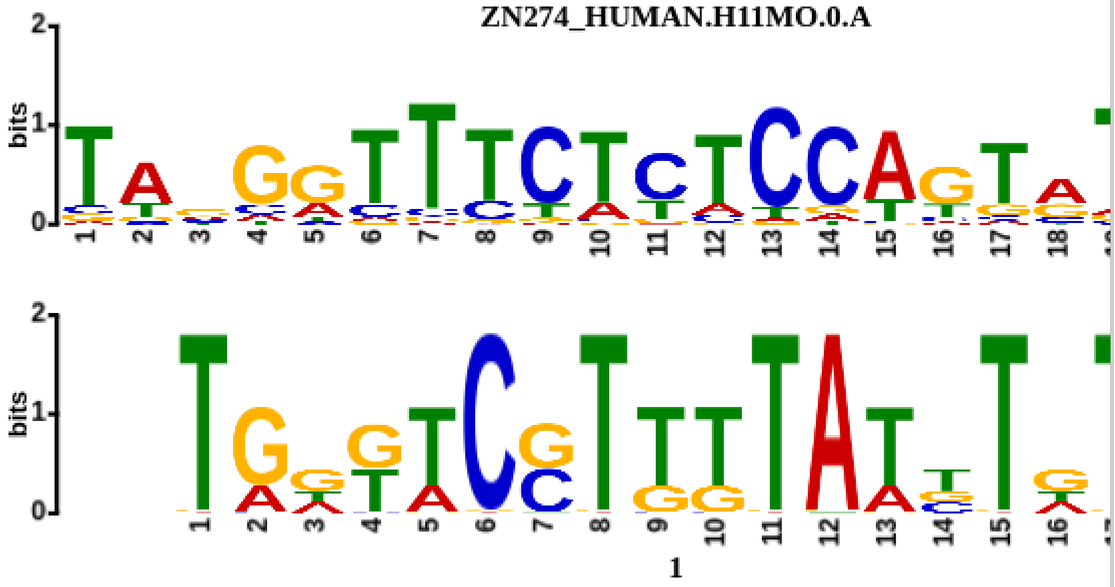

Summary

Optimal Alignment

**Name**	FOXO1_HUMAN.H11MO.0.A
**Database**	HOCOMOCOv11_core_HUMAN_mono_meme_format
***p*-value**	1.29e-02
***E*-value**	5.17e+00
***q*-value**	5.85e-01
**Overlap**	12
**Offset**	-3
**Orientation**	Normal

Show logo download options

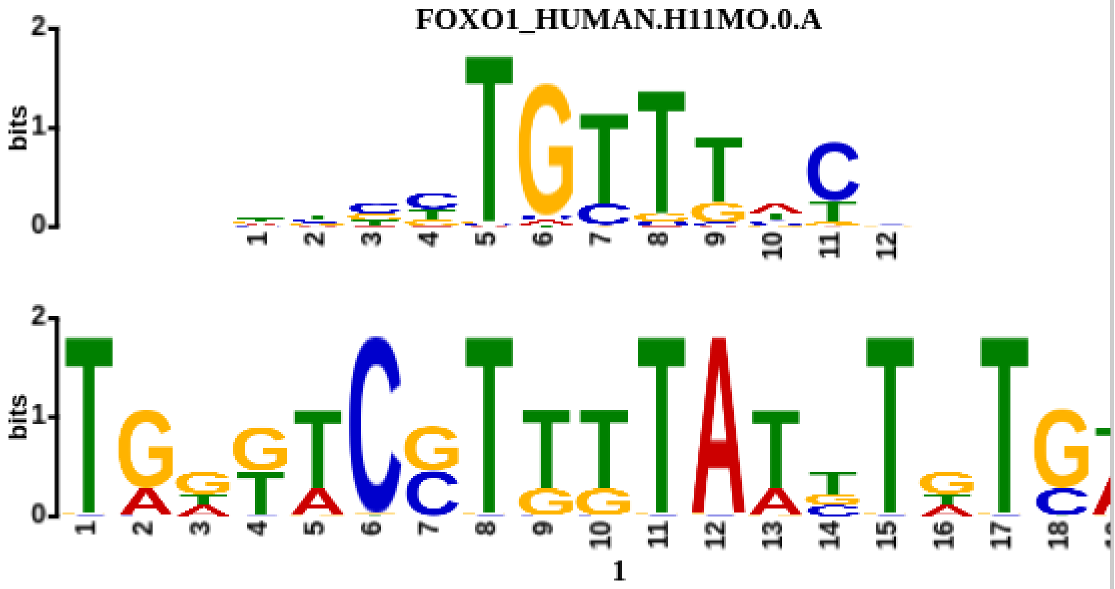

Summary

Optimal Alignment

**Name**	PRRX2_HUMAN.H11MO.0.C

**Database**	HOCOMOCOv11_core_HUMAN_mono_meme_format
***p*-value**	1.36e-02
***E*-value**	5.44e+00
***q*-value**	5.85e-01
**Overlap**	7
**Offset**	-9
**Orientation**	Normal

Show logo download options

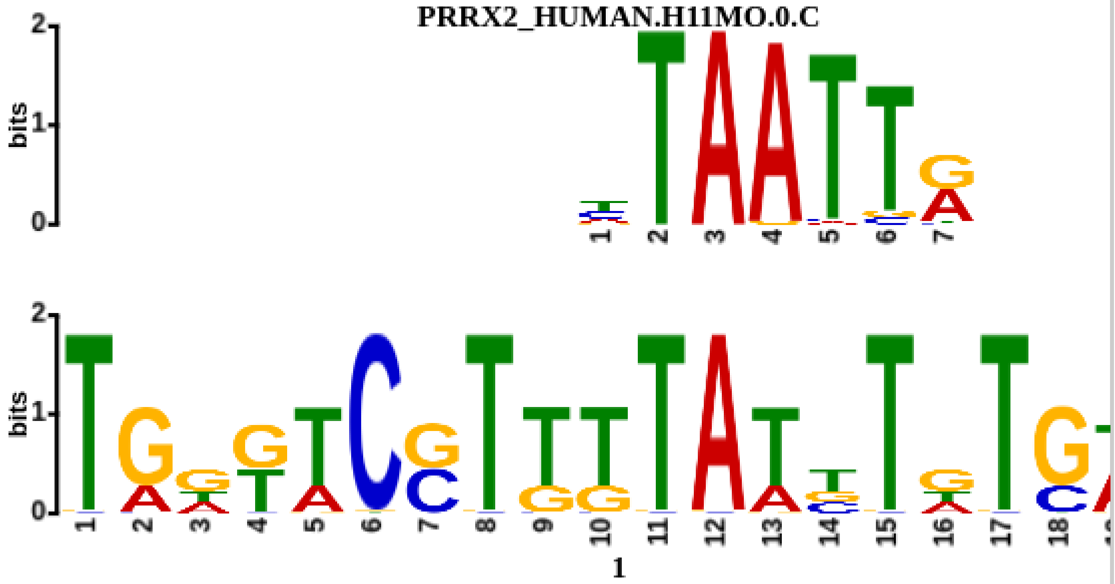

Summary

Optimal Alignment

**Name**	FOXJ3_HUMAN.H11MO.0.A
**Database**	HOCOMOCOv11_core_HUMAN_mono_meme_format
***p*-value**	1.40e-02
***E*-value**	5.61e+00
***q*-value**	5.85e-01
**Overlap**	13
**Offset**	-4
**Orientation**	Normal

Show logo download options

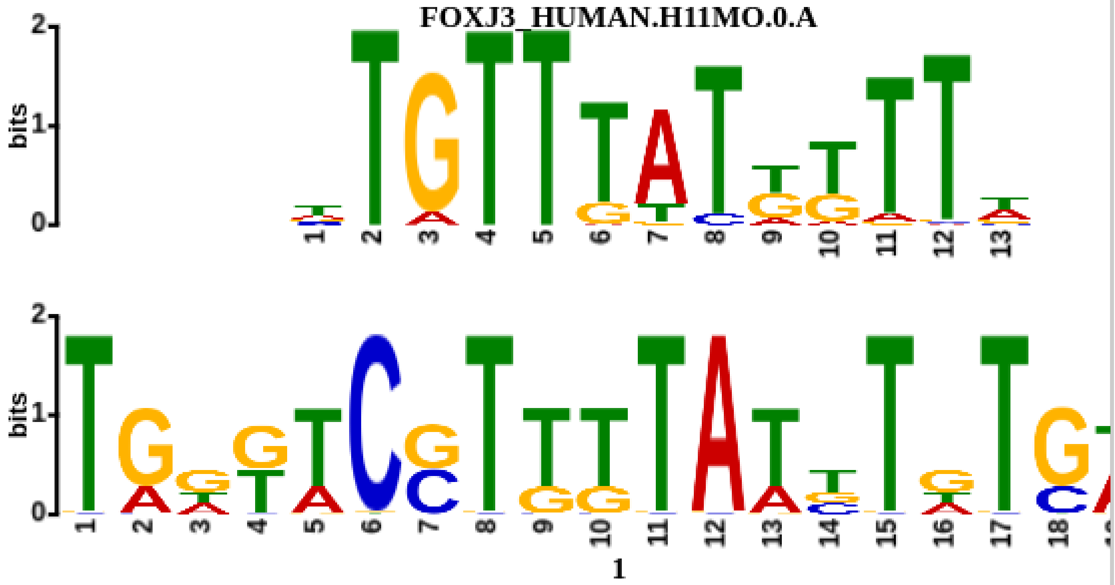

Summary

Optimal Alignment

**Name**	PIT1_HUMAN.H11MO.0.C

**Database**	HOCOMOCOv11_core_HUMAN_mono_meme_format
***p*-value**	1.40e-02
***E*-value**	5.61e+00
***q*-value**	5.85e-01
**Overlap**	13
**Offset**	-7
**Orientation**	Reverse Complement

Show logo download options

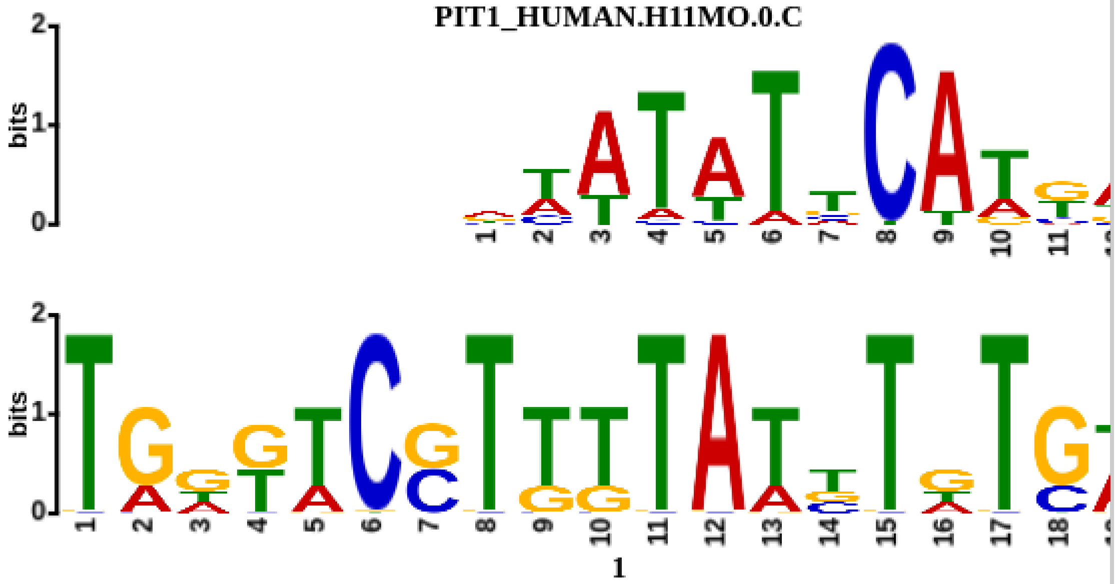

Summary

Optimal Alignment

**Name**	TBP_HUMAN.H11MO.0.A
**Database**	HOCOMOCOv11_core_HUMAN_mono_meme_format
***p*-value**	1.50e-02
***E*-value**	6.00e+00
***q*-value**	5.94e-01
**Overlap**	10
**Offset**	-5
**Orientation**	Reverse Complement

Show logo download options

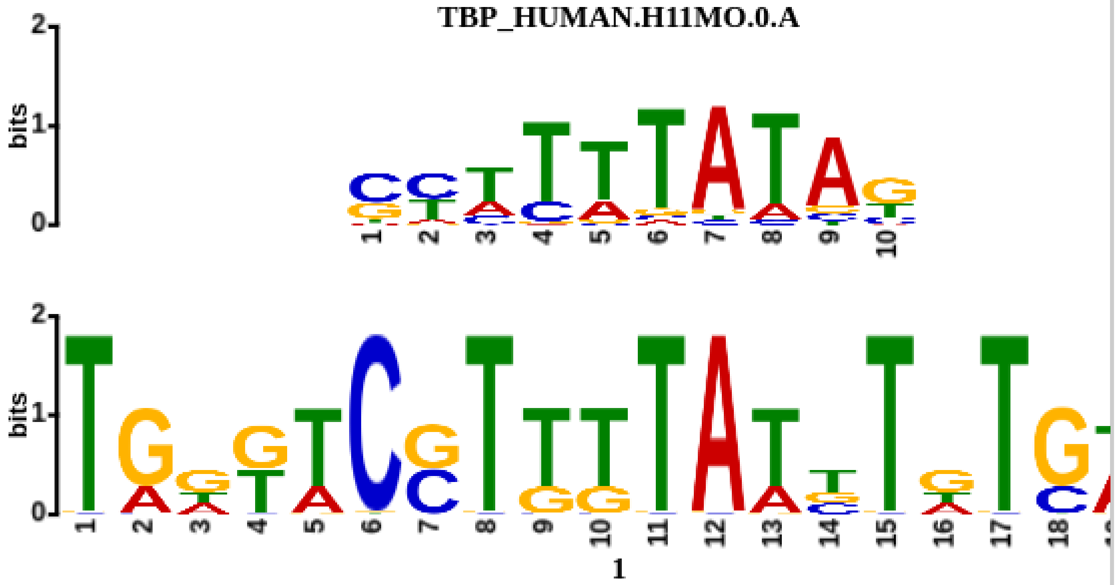

Summary

Optimal Alignment

**Name**	FOXK1_HUMAN.H11MO.0.A

Database HOCOMOCOv11_core_HUMAN_mono_meme_format

***p*-value** 1.60e-02
***E*-value** 6.40e+00
***q*-value** 6.03e-01

Overlap 10
Offset 0
Orientation Normal

[Show logo download options]

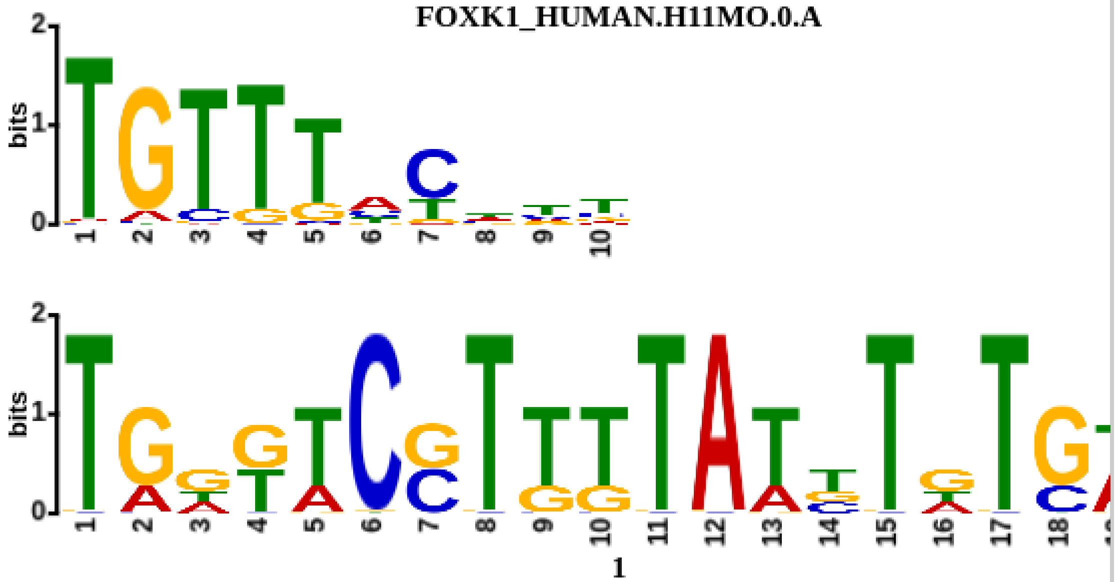

Summary

Optimal Alignment

Name [PO2F1_HUMAN.H11MO.0.C]
Database HOCOMOCOv11_core_HUMAN_mono_meme_format

***p*-value** 1.69e-02
***E*-value** 6.78e+00
***q*-value** 6.03e-01

Overlap 15
Offset 1
Orientation Reverse Complement

[Show logo download options]

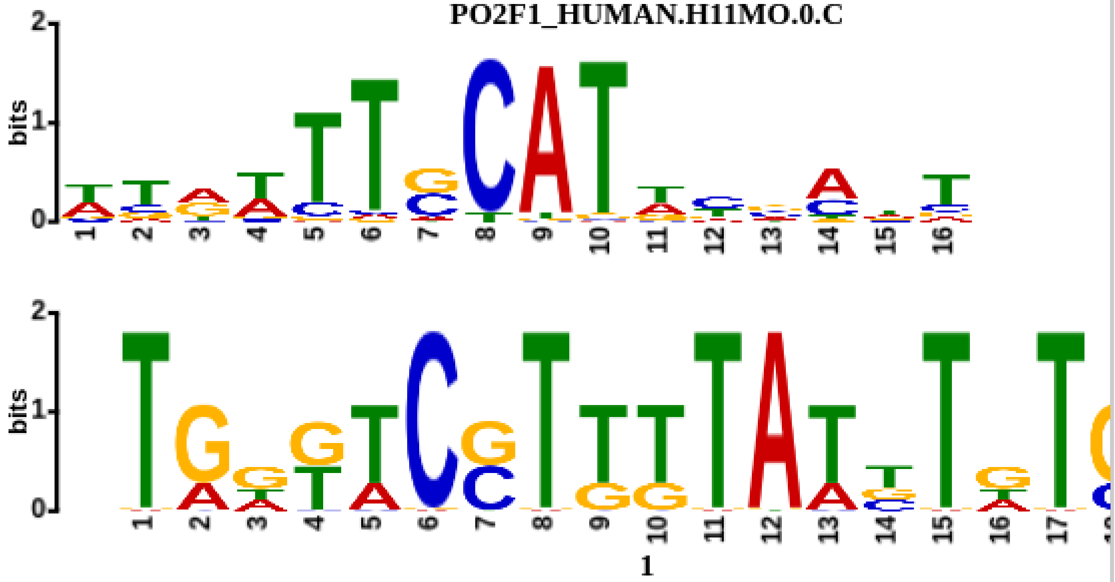

Summary

Optimal Alignment

Name [AIRE_HUMAN.H11MO.0.C]

**Database**	HOCOMOCOv11_core_HUMAN_mono_meme_format
***p*-value**	1.75e-02
***E*-value**	7.01e+00
***q*-value**	6.03e-01
**Overlap**	18
**Offset**	-2
**Orientation**	Normal

Show logo download options

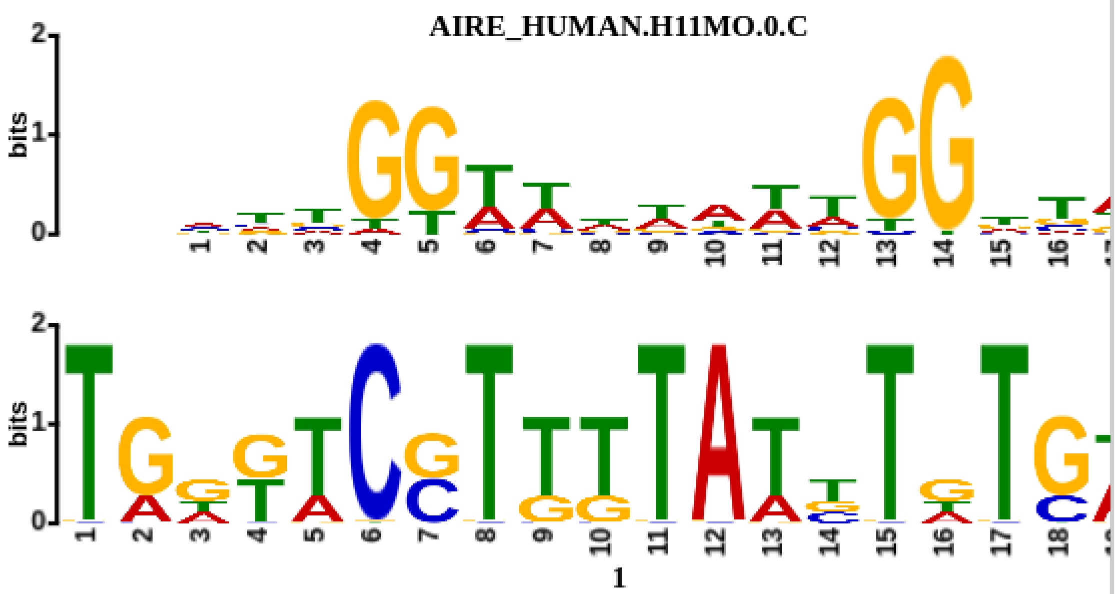

Summary

Optimal Alignment

**Name**	Z354A_HUMAN.H11MO.0.C
**Database**	HOCOMOCOv11_core_HUMAN_mono_meme_format
***p*-value**	2.12e-02
***E*-value**	8.51e+00
***q*-value**	6.44e-01
**Overlap**	20
**Offset**	2
**Orientation**	Normal

Show logo download options

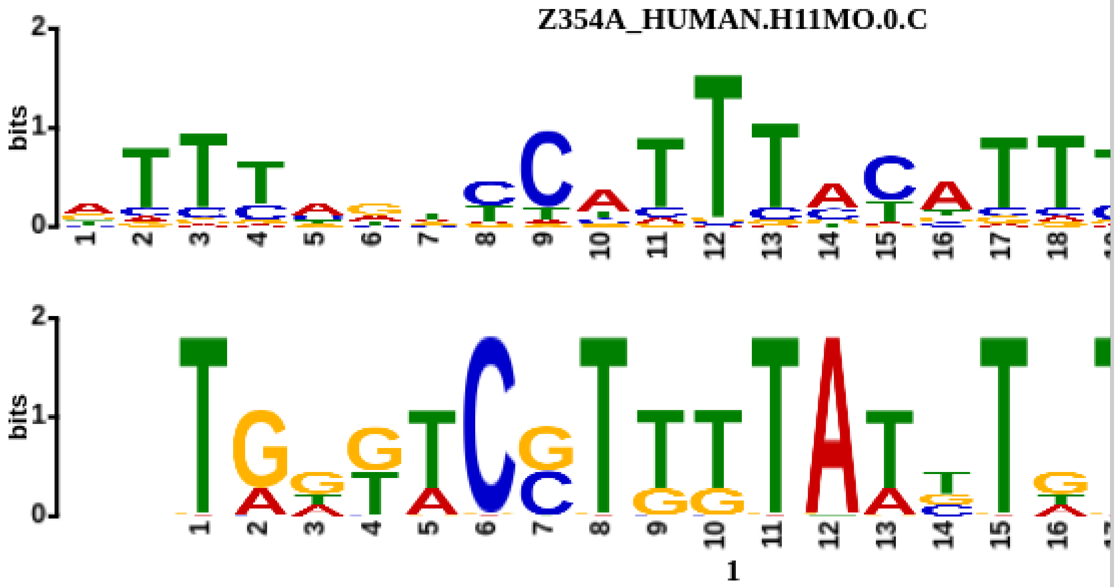

Summary

Optimal Alignment

**Name**	LEF1_HUMAN.H11MO.0.A

**Database**	HOCOMOCOv11_core_HUMAN_mono_meme_format
***p*-value**	2.23e-02
***E*-value**	8.96e+00
***q*-value**	6.44e-01
**Overlap**	14
**Offset**	-4
**Orientation**	Normal

Show logo download options

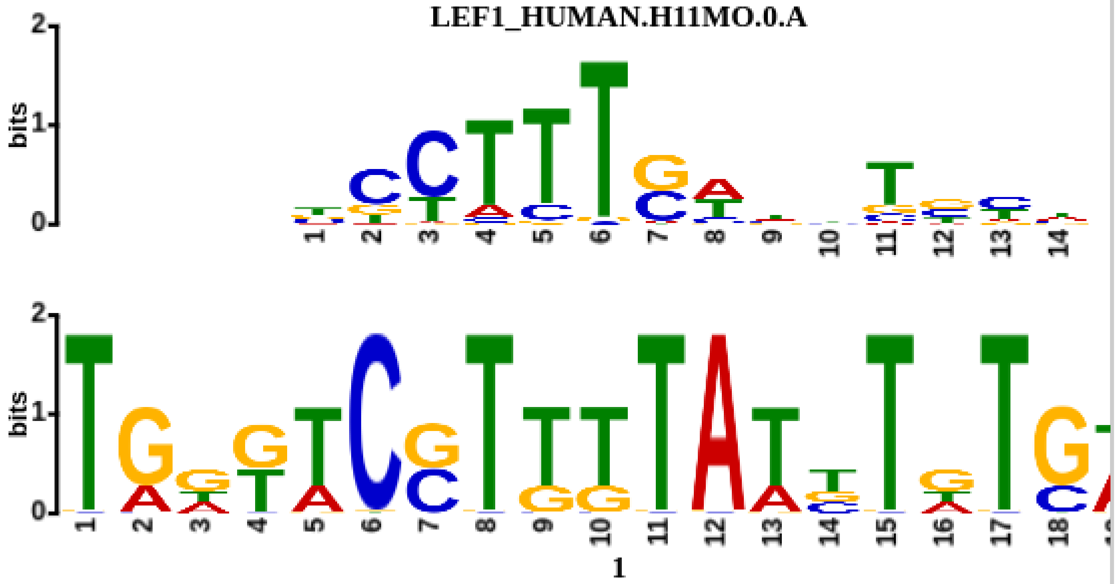

Summary

Optimal Alignment

**Name**	MEF2A_HUMAN.H11MO.0.A
**Database**	HOCOMOCOv11_core_HUMAN_mono_meme_format
***p*-value**	2.28e-02
***E*-value**	9.16e+00
***q*-value**	6.44e-01
**Overlap**	13
**Offset**	-7
**Orientation**	Normal

Show logo download options

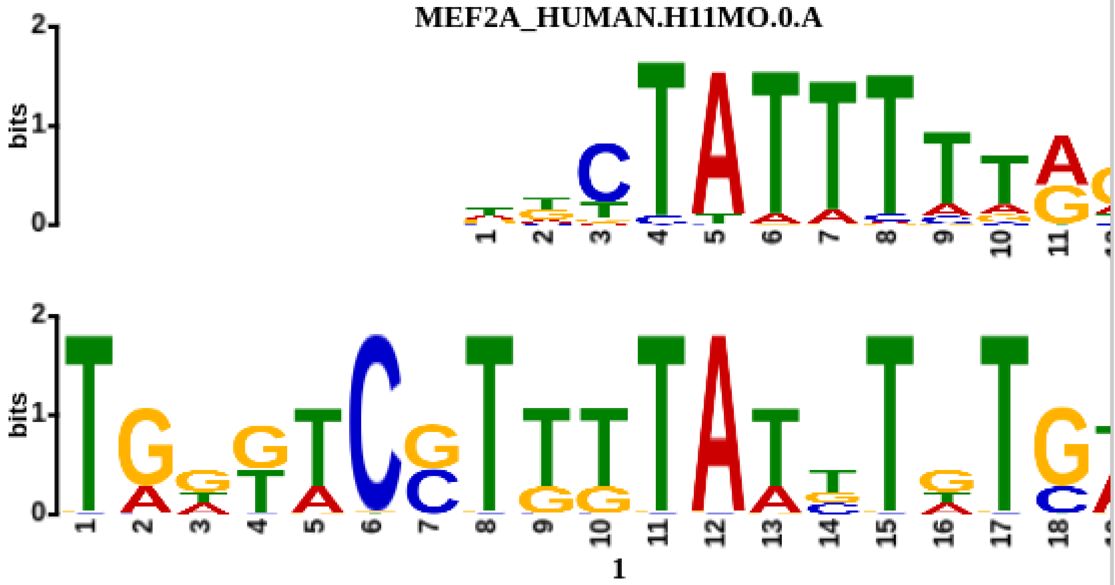

Summary

Optimal Alignment

**Name**	OVOL1_HUMAN.H11MO.0.C

**Database**	HOCOMOCOv11_core_HUMAN_mono_meme_format
***p*-value**	2.29e-02
***E*-value**	9.18e+00
***q*-value**	6.44e-01
**Overlap**	9
**Offset**	-8
**Orientation**	Normal

Show logo download options

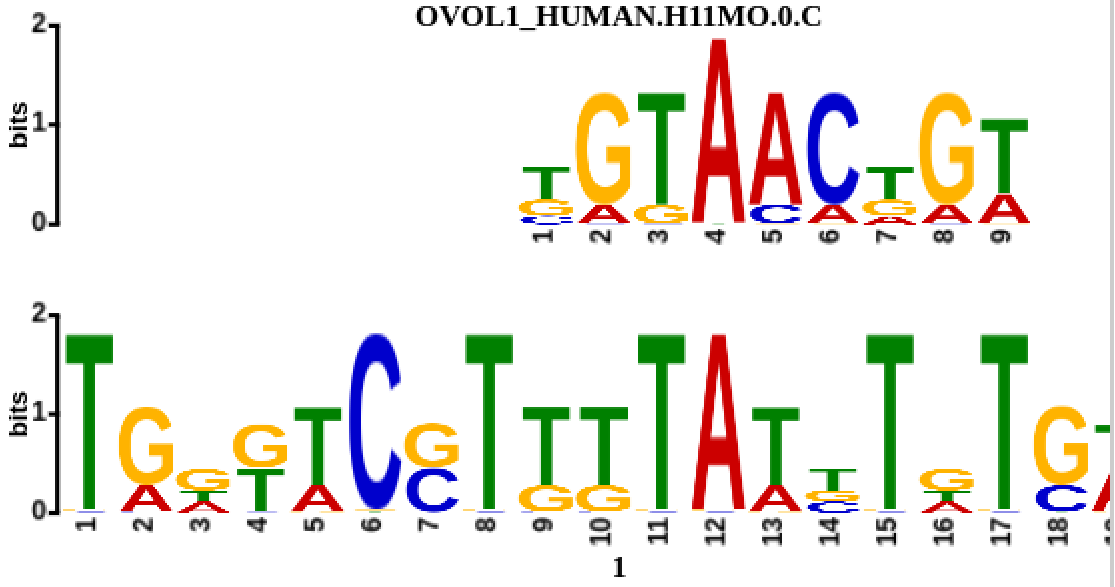

Summary

Optimal Alignment

**Name**	HXC9_HUMAN.H11MO.0.C
**Database**	HOCOMOCOv11_core_HUMAN_mono_meme_format
***p*-value**	2.32e-02
***E*-value**	9.32e+00
***q*-value**	6.44e-01
**Overlap**	10
**Offset**	-7
**Orientation**	Normal

Show logo download options

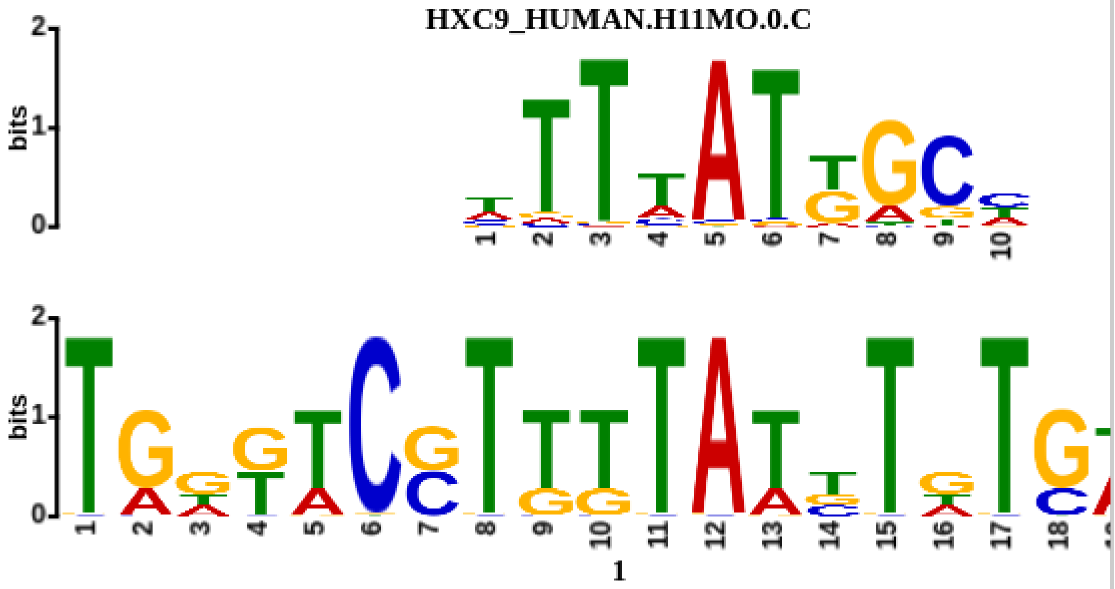

Summary

Optimal Alignment

**Name**	HNF6_HUMAN.H11MO.0.B

**Database**	HOCOMOCOv11_core_HUMAN_mono_meme_format
***p*-value**	2.35e-02
***E*-value**	9.44e+00
***q*-value**	6.44e-01
**Overlap**	11
**Offset**	-8
**Orientation**	Normal

Show logo download options

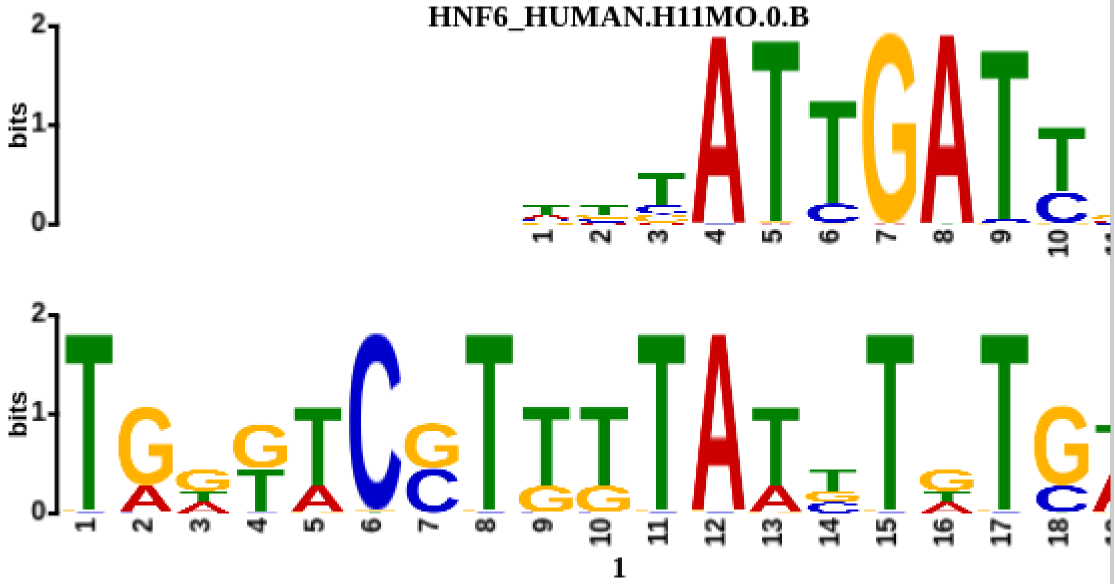

Summary

**Name**	HXB7_HUMAN.H11MO.0.C
**Database**	HOCOMOCOv11_core_HUMAN_mono_meme_format
***p*-value**	2.47e-02
***E*-value**	9.91e+00
***q*-value**	6.54e-01
**Overlap**	10
**Offset**	-8
**Orientation**	Normal

Show logo download options

Optimal Alignment

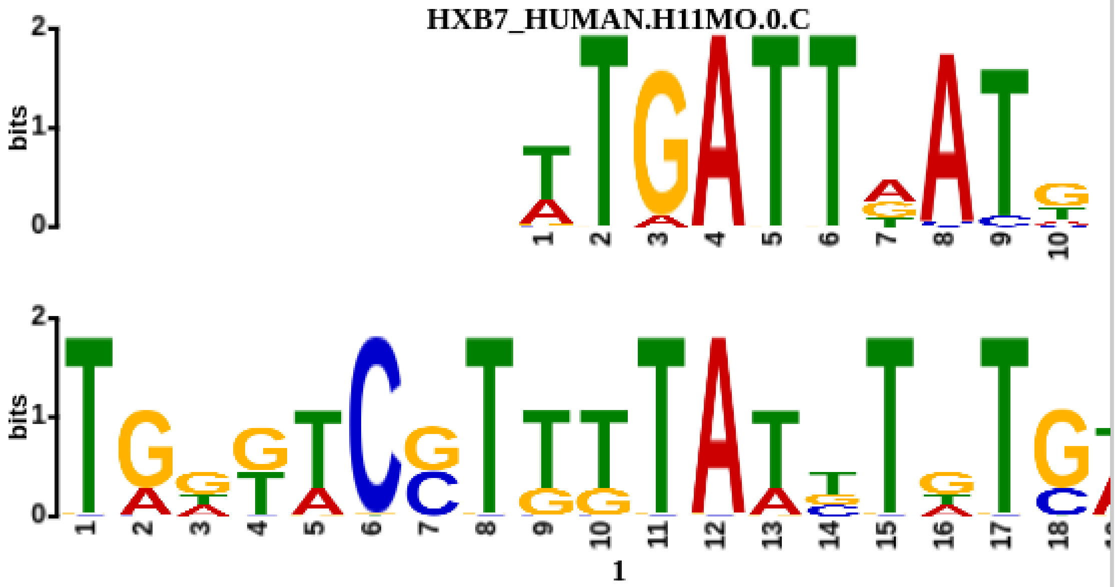

Settings

Previous Next Top

Alphabet

Source: the query file

Name	Bg.				Bg.	Name
Adenine	0.222	A	~	T	0.222	Thymine
Cytosine	0.278	C	~	G	0.278	Guanine

Other Settings

**Strand Handling**	Motifs may be reverse complemented before comparison to find a better match.
**Distance Measure**	Pearson correlation coefficient
**Match Threshold**	Matches must have a *E*-value of 10 or smaller.

Previous Top

Tomtom version
5.3.2 (Release date: Sat Feb 6 16:38:33 2021 -0800)

Reference
Shobhit Gupta, JA Stamatoyannopolous, Timothy Bailey and William Stafford Noble, "Quantifying similarity between motifs", *Genome Biology*, **8**(2):R24, 2007.

Command line
```
tomtom -no-ssc -oc . -verbosity 1 -min-overlap 5 -mi 1 -dist pearson -evalue -thresh 10.0 -time 300 query_motifs
```

Result calculation took 1.759 seconds

We certify that the following article

Familial dilated cardiomyopathy: a novel MED9 short isoform identification

Monica Franzese, Mario Zanfardino, Andrea Soricelli, Annapaola Coppola, Ciro Maiello, Marco Salvatore, Concetta Schiano *, Claudio Napoli

has undergone English language editing by MDPI. The text has been checked for correct use of grammar and common technical terms, and edited to a level suitable for reporting research in a scholarly journal.
MDPI uses experienced, native English speaking editors. Full details of the editing service can be found at
► https://www.mdpi.com/authors/english.

MDPI Multidisciplinary Digital Publishing Institute

Basel, Switzerland
February 2024

AUTHORservices

english-77574
